# A large-scale population based organelle pan-genomes construction and phylogeny analysis reveal the genetic diversity and the evolutionary origins of chloroplast and mitochondrion in *Brassica napus* L.

**DOI:** 10.1186/s12864-022-08573-x

**Published:** 2022-04-30

**Authors:** Hongfang Liu, Wei Zhao, Wei Hua, Jing Liu

**Affiliations:** 1 Oil Crops Research Institute of the Chinese Academy of Agricultural Sciences, Key Laboratory of Biology and Genetic Improvement of Oil Crops, Ministry of Agriculture and Rural Affairs, Wuhan, 430062 China; 2 Hubei Hongshan Laboratory, Wuhan, 430070 China

**Keywords:** *Brassica*, Rapeseed, Organelle pan-genome, Mitotype, Cytoplasm haplotype, Maternal ancestor

## Abstract

**Background:**

Allotetraploid oilseed rape (*Brassica napus* L.) is an important worldwide oil-producing crop. The origin of rapeseed is still undetermined due to the lack of wild resources. Despite certain genetic architecture and phylogenetic studies have been done focus on large group of *Brassica* nuclear genomes, the organelle genomes information under global pattern is largely unknown, which provide unique material for phylogenetic studies of *B. napus*. Here, based on de novo assemblies of 1,579 *B. napus* accessions collected globally, we constructed the chloroplast and mitochondrial pan-genomes of *B. napus*, and investigated the genetic diversity, phylogenetic relationships of *B. napus*, *B. rapa* and *B. oleracea*.

**Results:**

Based on mitotype-specific markers and mitotype-variant ORFs, four main cytoplasmic haplotypes were identified in our groups corresponding the *nap*, *pol*, *ole*, and *cam* mitotypes, among which the structure of chloroplast genomes was more conserved without any rearrangement than mitochondrial genomes. A total of 2,092 variants were detected in chloroplast genomes, whereas only 326 in mitochondrial genomes, indicating that chloroplast genomes exhibited a higher level of single-base polymorphism than mitochondrial genomes. Based on whole-genome variants diversity analysis, eleven genetic difference regions among different cytoplasmic haplotypes were identified on chloroplast genomes. The phylogenetic tree incorporating accessions of the *B. rapa*, *B. oleracea*, natural and synthetic populations of *B. napus* revealed multiple origins of *B. napus* cytoplasm*.* The *cam*-type and *pol*-type were both derived from *B. rapa,* while the *ole*-type was originated from *B. oleracea.* Notably, the *nap*-type cytoplasm was identified in both the *B. rapa* population and the synthetic *B. napus*, suggesting that *B. rapa* might be the maternal ancestor of *nap*-type *B. napus*.

**Conclusions:**

The phylogenetic results provide novel insights into the organelle genomic evolution of *Brassica* species. The natural rapeseeds contained at least four cytoplastic haplotypes, of which the predominant *nap*-type might be originated from *B. rapa*. Besides, the organelle pan-genomes and the overall variation data offered useful resources for analysis of cytoplasmic inheritance related agronomical important traits of rapeseed, which can substantially facilitate the cultivation and improvement of rapeseed varieties.

**Supplementary Information:**

The online version contains supplementary material available at 10.1186/s12864-022-08573-x.

## Introduction

Rapeseed is one of the most important oilseed crops in the world, which is also utilized as protein feed and a source of industrial raw materials [1]. It is an allopolyploid species (AACC, 2n = 38) that originated from the hybridization of *Brassica rapa* (AA, 2n = 20) and *Brassica oleracea* (CC, 2n = 18) approximately 7,500 years ago [2–4] and was first cultivated in Europe [5]. Despite its relatively shorter domestication history compared to those of other crop plants, such as rice (*Oryza sativa*) [6] and soybean (*Glycine max*) [7], rapeseed has adapted to different eco-environmental conditions and was domesticated into three distinct ecotype groups, namely winter, semi-winter, and spring types, based on growth and flowering characteristics [8–10]. The vernalization times of winter and semi-winter rapeseed are more than one month and 15–20 days, respectively, whereas spring rapeseed does not require vernalization.

Large-scale genome sequencing projects have been performed to better understand the evolutionary and mechanism of rapeseed phenotypic diversity formation, since high-quality *B. napus* genomes facilitate the genome-wide sequences comparison among a wide range of diverse materials [3, 11–14]. By resequencing 991 germplasm resources collected from worldwide 39 countries, the global pattern of genetic polymorphism in rapeseed was determined which ulteriorly revealed the pathways of population splits and mixtures and uncovered the genetic basis of ecotype divergence [15]. Another resequencing project of 588 *B. napus* accessions revealed that winter oilseed may be the original form of *B. napus*, and identified genetic loci associated with stress tolerance, oil content, seed quality, and ecotype improvement by integrating genome-wide association studies, selection signals, and transcriptome analysis [4]*.* Based on a collection of 1,688 rapeseed resequencing data, a genomic platform composed of multi-omics data and common bioinformatics tools, the BnPIR database was constructed, which contains gene information, phylogenetic relationship, expression data and presence/absence variations (PAVs) information [16]. Population resequencing strategy is especially efficient for delving phylogenetic, phylogeography, and population genetics information.

Extensive resequencing studies have been conducted on the rapeseed genome. However, scarce research has been focused on organelle genomes, which are also important components of genetic information. The chloroplast (cp) and mitochondrial (mt) genomes of land plants contain a circular molecule of DNA with a relatively small genome size respectively: the size of the chloroplast genomes ranges between 115 and 165 kb [17], and the mitochondrial genomes have fluctuant size of approximately 200 kb–2 Mb [18]. Cytoplasmic inheritance was found to significantly contribute to the formation of most agronomical important traits of crops, such as yield, low-temperature tolerance, grain weight, filled-grain ratio, and milling quality traits in *indica* rice [19–21], plant height in maize [22], seed protein content in soybean [23, 24], and oil content in rapeseed [25–27]. Up to now, bits of organellar genes have been proved to be phenotypically important. For instance, the mitochondria-encoded *orf188* was identified as a potential rapeseed oil content determinated gene in our previous study [28], but the genetic mechanisms of cytoplasmic activities determining agronomic traits have not been fully explored. In addition, the key structural and functional component-encoding genes [29–31] are crucial to a better understanding of the mechanisms of evolutionary divergence [32–36]. Evolutionary studies have been conducted on mt and cp genomes using contemporary, highly effective extraction and assembly methods to obtain plastid and mt genomes [37], which have provided deeper research insights into the genetics of *B. napus* [38–42]. However, most investigations have been focused on establishing the relationships among different species or varieties based on the collection of a small number of germplasms. Recently, population-based organelle genome studies with a large number of accessions have been conducted in crops. Based on 412 rice cp and mt genomes analysis, *indica* and *japonica* were found to have experienced different domestication processes [43, 44]. The organellar phylogenies were combined with nuclear for *B. napus* and its progenitors, contributing to reveal varying patterns of inheritance and post-formation introgression [41]. However, the genetic diversity, population structure and genetic basis of agronomic characters for global rapeseed organellar genomes have not been deeply analyzed.

In this study, we developed an organellar genome dataset and performed a comprehensive study of a large number of diverse accessions: 1,579 natural [4, 15] and 31 synthetic *B. napus* [45]*,* 199 *B*. *rapa*, and 119 *B*. *Oleracea* [46]. The organelle genome sequencing reads were extracted out and assembled for each accession. All cyclic assemblies were next merged to construct the organellar pan-genome and the allelic variant dataset. Our analysis was focused on the phylogenetic relationships, population structure, and genetic diversity, especially concerning the divergence of different cytoplasmic groups, which provided new organelle genomic evidence on rapeseed origin and evolution. Additionally, these allelic variations supplied comprehensive information that could serve as the basis for follow-up studies on cytoplasmic effect-affected crop agronomic traits. Moreover, our findings facilitate the acceleration of the process of organelle genome-assisted breeding in the near future.

## Results

### Rapeseed organellar genomes (cp and mt) assembly and annotation

The genome sequencing data of 1,579 rapeseed accessions from all major production countries were obtained from the NCBI database under SRP155312 [15] and PRJNA358784 [4]. After quality checking and trimming for low-quality regions from two ends of reads, we first mapped clean data to a mitochondrial genome sequence cluster consisting of 35 mt genomes, and a chloroplast genome sequence cluster consisting of 42 cp genomes respectively. Both of the two data sets contained six *Brassica* species (Table S1). The mapped paired-end reads were extracted and de novo assembled for the cp and mt genomes by NOVOPlasty [47] and ARC software (http://ibest.github.io/ARC/), respectively. The extracted data sizes of the samples differed (from 20 to 400 Mb in mt and from 11 Mb to 1.8 Gb in cp), but all reached high average coverage (> 100 ×).

**Fig. 1:**
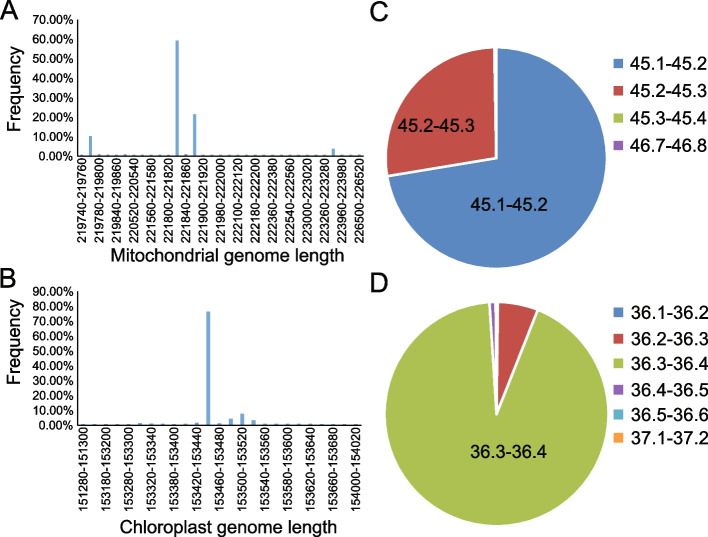
Sequence feature of assembled chloroplast and mitochondrion genomes. **A** Length distribution of mitochondrial genomes and **B** chloroplast genomes. **C** G + C content of mitochondrial genomes and **D** chloroplast genomes

The organellar genomes (cpDNA and mtDNA) were subsequently assembled individually for each accession. A total of 1,327 cpDNA and 1,456 mtDNA were assembled into single circular-mapping molecules and were considered in the downstream analysis. The relatively concentrated size of the cp genomes was 153 kb, whereas the sizes of the mt genomes ranged from 219 to 226 kb (Fig. 1A,B). The main G + C content of the cp and mt genomes was approximately 36.3% and 45.2%, correspondingly (Fig. 1C,D). Of all assembled sequences, 47 cpDNAs and 225 mtDNAs contained gap filling base “N”, whose lengths ranged from 1 to 770 bp.

The assembled genomes were annotated with GeSeq [48] using a reference consisting of gene sets obtained from known *Brassica* species, genomes of which were also used as the reference in the aforementioned mapping. After removing the duplications, the cp reference gene set contained 79 protein-coding genes, 2 function-unknown open reading frame (ORFs), 4 rRNAs, and 21 tRNAs. The mt reference gene set included 35 protein-coding genes, 3 rRNAs, 17 tRNAs, and 80 ORFs, 33 of which corresponded eight homologous groups were not possessed consistently by different mitotypes [49].

Reference genes were all detected in each of the 1,327 assembled rapeseed cpDNAs. Due to the existence of multiple copies of several genes, there were 87 locus corresponding 79 protein-coding genes were identified in 99% of the accessions, 8 rRNAs locus (double copies for all 4 rRNAs) were identified in all samples and 37 tRNAs were identified in 99% of the accessions. Of all predicted chloroplast genes, 95% genes had more than 98% similarity with the reference genes.

Similar to the cp, the mt genes were also highly conserved. Each of the assembled genome was predicted to contained all the reference genes except for mitotype-specific ORFs (35 protein-coding genes, 3 rRNAs, 17 tRNAs, and 47 ORFs). And the gene sequences were in high degree of similarity with the reference gene sequence. Among all predictions, 99% genes showed 98% coverage, and 93% genes showed 98% identity. In the subsequent analysis, we excluded the abnormal accessions whose 10% of the genes aligned less than 80% bases with reference, which was far lower than other samples.

As described above, all accessions were predicted to contain all the reference genes, indicated the completeness of the assemblies in terms of gene content. To evaluate the quality of the genome assembly further, we first conducted a comparison analysis of organelle genomes with the public *Brassica* species. The assembled organelle genomes were mapped by BLASTN [50] with a filter criteria of identity > 0.9, and only the subject with optimal alignment for each sample was retained. The homology sequences between the assemblies and the corresponding mapped subjects were more than 97.5% (all queries and subjects had > 97.5% coverage), suggesting the current assembly was completed. Additionally, we mapped reads used for assembly back to the assembled single circular sequence for each accession. The coverage and depth were measured based on exactly matched reads, which meant that reads with mismatch, deletion, insertion, and soft or hard clipping were all filtered out. 97.7% of the assemblies were covered by at least 150 × reads along the whole genome, and the curve graph of the mapping depth along genome body was roughly steady (Figure S1, S2), which guaranteed the high level of accuracy of the assembly at the single-nucleotide level. Moreover, to evaluate whether there were structural errors in our assemblies, we detected the continuity of reads mapping start sites on genome. Gap was defined here as >  = 150 bp without any read mapping start site. If any gap is detected, there may be breakpoints because most reads have a length of 150 bp and the sequence depth is greater than 100x. We found that no gap was detected in 80% of the assemblies (without sequence with ‘N’). The results showed that more than 80% of the accessions were assembled with high quality at the structural level for unique genome regions, which further confirmed the high-quality assembly of the *Brassica napus* organelle genome sequences.

### Construction of *B.napus* organelle pan-genome

We constructed the organellar pan-genome of *B. napus* using a reference-based assembly approach (Additional File 1). Organelle genomes of R4834 were taken as mt and cp draft genomes because of their largest number of alignments with other accessions. Variants calling was performed using local Perl script based on the whole genome alignments of assembled *B. napus* cp and mt with the reference. The draft genome was edited by inserting the insertion fragments (> 10 bp) and was supplemented by adding unanchored fragments (> 100 bp) which were supported by at least two accessions.

**Fig. 2:**
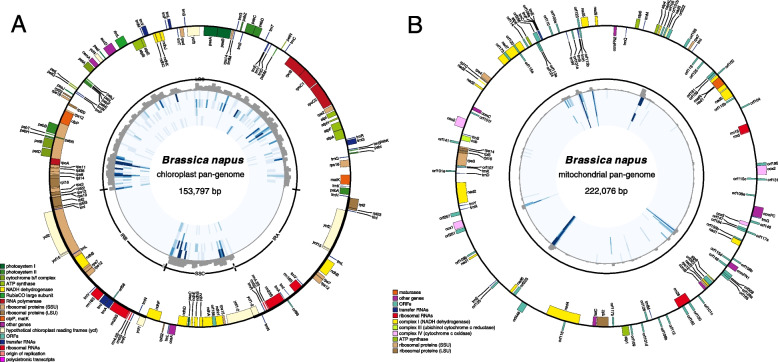
Circular genome maps and distribution of variants of the **A** Chloroplast and **B** mitochondrial Genome of *Brassica napus*. The outer circle is the organellar genome map displaying genes in different functional categories (listed in the legend) distinguished by colors. Genes marked inside the circle are transcribed clockwise, while outside the circle are counter clockwise. The inner circle displays the distribution of variants in different groups, the dark gray bars representing all assembled accessions, and blue highlights corresponding to four different cytoplasm (*nap*, *cam*, *pol* and *ole*), the higher the bar or the darker the color, the greater the density. The length of each bar denotes the total number of variants in a 500-bp window

The alignment results showed high structural stability of the cp genome with nearly no recombination sequences. The cp pan-genome was a 153,797 bp circular molecule, which was composed of a large and a small single-copy (LSC and SSC) region between two reverse repeats. We identified a total of 87 proteins, 3 ORFs with unknown functions, 8 ribosomal RNAs, and 37 tRNA sequences (Fig. 2A), accounting for approximately 49% of the genome.

The total size of the mt pan-genome was 231,901 bp, which was composed of a 222,076-bp circular molecule and 5 dispensable scaffolds with an average length of 1,965 bp (Fig. 2B). A total of 35 protein-coding genes, 3 rRNAs, 18 tRNAs, and 62 ORFs were annotated, accounting for 28% of the genome sequence. Of them, 15 ORFs were mitosis-specific, including the CMS-related gene *orf224* [52].

The presence-absence variation (PAV) of organellar genes is an important genetic factor affecting organelle inheritance agronomic traits. Here, the whole-genome assembly and gene annotation provided information for the PAV analysis of whole-worldwide *B. napus* organellar genomes. We defined the criteria of gene presence-absence as a 60% cut-off threshold value for coverage and 85% for identity with the reference. All the protein-encoding cp genes were core, in which only one gene *ycf2* was discordant for an additional copy in only one accession (Table S2). All four species of the ribosome genes appeared twice in 1,327 accessions, but 3 of the 25 species of tRNAs had different copy numbers in the population, and all were different in a few samples (< 1%). In general, all cp genes were found in 1,327 accessions, but only four of them had different copies in a small number of accessions.

Without regarding to the mitotype-specific ORFs in the mt genomes, none was missing in any assembled accessions, and only 3 ORFs (*orf108a*, *orf115c* and *orf131*) and 1 tRNA (*trmH*) were found to have different copy numbers in less than 1% accessions (Table S3). As a summary of the above information, all protein-coding genes of the mt genome were core, and 4 were differed in the copy number.

### Identification of the cytoplasm haplotype

The cp genomes are known to be highly conserved, whereas extensive recombination and sequence gain and loss, induced by rearrangement, are observed in mt genomes [49, 53, 54]. Therefore, the sequence variants in mt genomes account for the majority of cytoplasm-specific sequences. To distinguish the cytoplasmic types of all assembled accessions, 12 mitotype-specific markers (genome-specific sequences, > 100 bp) developed by Heng et al. [55] and 34 homologous ORFs [49] which is not consistent in six *Brassica* mitotypes were used for searching in each mt genome. We defined the cytoplasmic type for the sample that completely conformed to the relationship between mitochondrial type and MSSs/ORFs. Samples that met the corresponding conditions of mixed MSSs and ORFs were defined as the "like" type based on structural comparison.

Four major types of mitotypes were detected in the 1,456 assembled accessions. There were 1,215 cases of *nap* cytoplasm, 170 cases of *cam* cytoplasm, 53 cases of *pol* cytoplasm, and the remaining 18 cases of *ole* cytoplasm (Table 1, Supplementary Table S4). The *nap*-type accessions accounted for 83% of the group, indicating that *nap* mitotype was predominant in natural *B. napus* [39]. Accessions with same cytoplasm type exhibited consistent organelle genome structure supported by Mugsy [56] alignments. To check the structural characterization of organelle genomes, four cp and four mt genomes from different cytoplasms were selected to construct multiple genome alignments and identify large-scale rearrangements by Mauve (http://darlinglab.org/mauve/mauve.html) (Figure S3). The cp genomes from different cytoplasms showed collinearity for the whole genome. However, the four mt genome mitotypes had rearrangements. The *cam* and *pol* were found to be completely collinear as they shared all same collinear blocks in a consistent order and direction, but at least three recombination events had occurred between any other two mt genomes.

**Table 1:** Classification and distribution of mitotypes among 1,456 assembled *B.napus* accessions

Cytoplasm type	Number	Percentage(%)
*nap*	1,215	83.45
*cam*	170	11.68
*pol*	53	3.64
*ole*	18	1.24

To explore whether a certain correlation or bias existed between ecotypes and mitotypes, we separately established the distribution of different cytoplasms in three ecotypes and that of different ecotypes in four cytoplasms (Figure S4). No significant differences were observed in the distribution of cytoplasms among the three ecotypes, which was similar to the overall distribution, with the exception of 28% of the *cam* type in the spring group, which was less than 10% in the other three groups. However, the ratio of the three ecotypes in different cytoplasmic types showed inconformity. The *pol*-type accessions were mainly semi-winter (83.63%), and only two ecotypes (61.11% of the winter and 35% of the semi-winter ecotype) were detected in the *ole* accessions. The numbers of three ecotypes in *cam* were approximate. The ratio of the semi-winter, spring, and winter ecotypes in the *nap* accessions was 2:1:4.

### Genome-wide cytoplasmic variations in *Brassica*

A total of 4,115 and 450 primary variants were detected in assembled cp and mt genomes, respectively. After removing single sample-specific variants, 2,092 high-quality variants were obtained from the cp genomes, including 1,660 SNPs (79.35%) and 433 small InDels (20.65%). Additionally, 254 SNPs (77.91%) and 72 small InDels (22.09%) were identified in the mt genomes, reaching a density of 13 and 1.5 variants per kb in the cp and mt genomes, correspondingly (Table 2). In the whole population, the number of SNPs and small InDels detected in cpDNAs were much higher than that detected in mtDNAs, but the number in each subgroup was lower in cpDNAs, indicating that SNP/small InDels between different cytoplasm were more abundant in cp than in mt, whereas no structural variations were detected in cpDNAs.

**Table 2:** Summary of the total and subgroup variants (SNPs and InDels)

Groups	Cp Variant			Mt Variant		
	**SNPs**	**InDels**	**Ts/Tv **^**a**^	**SNPs**	**InDels**	**Ts/Tv**
subgroups						
*nap*	131	64	0.5	213	49	1.01
*cam*	104	75	0.62	90	24	0.92
*pol*	9	17	0.29	138	36	0.97
*ole*	31	17	0.58	64	8	1
All	1,660	433	0.78	254	72	0.95

^a^ Ts/Tv is the proportion of transition/transversion

The cp variants were evenly distributed along the reference genome, except for two inverted-repeat regions, whose reads were skipped due to multiple alignments (Fig. 2). Of the overall 1,327 cp variants, 53% were located in upstream/downstream regions, 11% were in introns, and 31% of the variants were found in coding regions, of which 43% were predicted to be non-synonymous or frameshift (Table 3, Table S5), resulting in a potentially different functional protein encoding. The distribution of the variants along the genome was consistent with the overall distribution of the four cytoplasm types.

**Table 3:** Genome distribution of variants (SNPs and InDels)

Region	Cp Variant	Mt Variant
downstream/upstream	1,279	147
exonic		
synonymous / nonframeshift	427	64
nonsynonymous / frameshift	322	76
stopgain	5	1
stoploss	1	1
unknown	0	10
intergenic	0	26
intronic	369	8
splicing	0	1
ncRNA_exonic	4	1
Total ^a^	2,407	335

^a^ Total number here was larger than described above because several locus had multiple variants

The mt variants were scattered throughout the whole reference genome but were enriched (*P-*value < 0.0001, phyper test) in the coding region. Of note, 45% of the variants were located in the coding region, which constituted a much higher frequency than the 28% of the genome sequences in all coding areas. Of the variants in the coding region, 58% were predicted to be functionally affected (Table S5). The ratio of cp variants detected in four cytoplasm types were not similar to that of mt genomes. Additionally, inconsistent transition/transversion (Ts/Tv) ratio was also observed (Table 2). The proportion of four mitotypes was approximate 1 in the mt genomes, whereas range from 0.29 to 0.78 in the cp genomes, suggestint the Ts/Tv proportion in cpDNA was lower than that in mtDNA. There were four common cp SNPs and 57 common mt SNPs in the four subgroups (*nap*, *pol*, *cam*, and *ole* mitotypes). Except for *pol*, most the cp variants of each genotype were specific, whereas only a small proportion of the mt variants were specific (Figure S5).

### Diversity analysis of cytoplasmic genomes

**Fig. 3:**
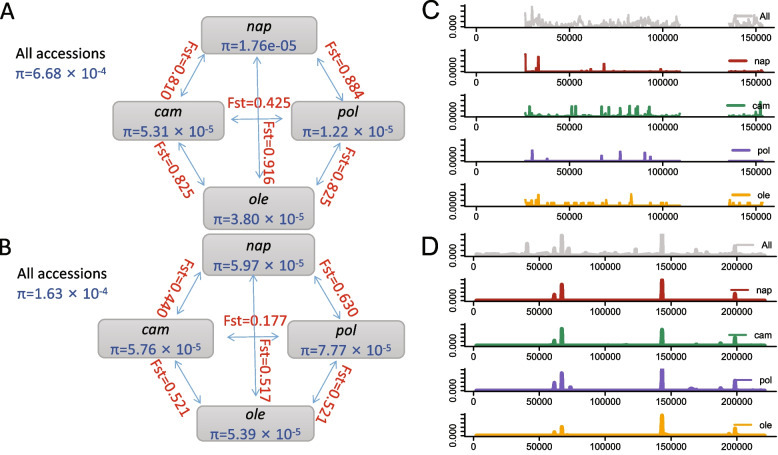
Organelle genome- and subgenome-wide comparison of nucleotide diversity and FST during the four main cytoplasm ypes of *B. napus*. **A** Nucleotide diversity and Fst between four group based on cpDNA. **B** Nucleotide diversity and Fst between four group based on mtDNA. **C** Nucleotide diversity along cp genome except the two inverted repeat region. **D** Nucleotide diversity along mt genome. π values were estimated for 500-bp sliding windows with 100-bp step size along organellar genomes across different cytoplasmic groups

**Fig. 4:**
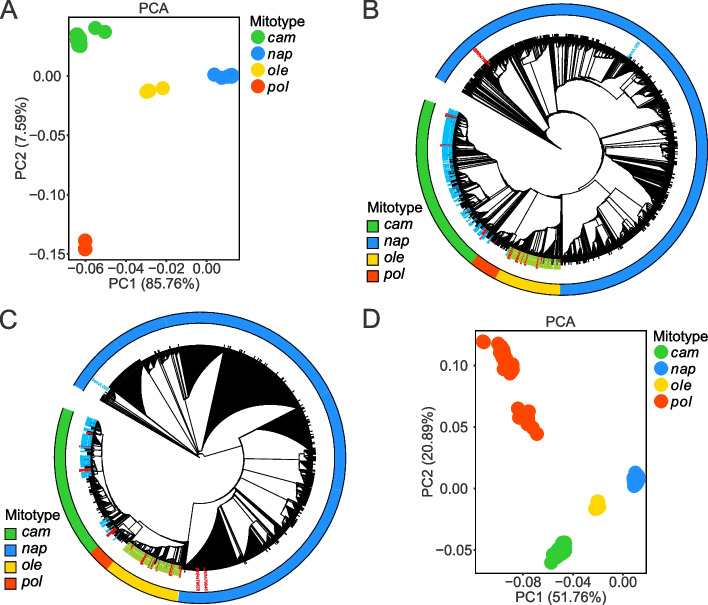
Population structure of *B. napus* accessions and its two progenitor species. **A** PCA plot of assembled cp genomes*.* **B** Chloroplast phylogeny of assembled *B. napus* combined together with 199 *B. rape*, 119 *B. oleoracea* and 31 synthetic *B. napus* accessions. **C** Mitochondria phylogeny of *B. napus* and its two progenitor species. **D** PCA plot of assembled mt genomes. ML phylogenetic tree were constructed using SNPs after filtering loci with minor allele frequencies (MAFs) < 0.005 and missing calls > 20%. In PCA plots, samples of different cytoplasmic types were highlighted as dots in different colors. In phylogenetic tree, the cytoplasmic type of each sample was annotated at outer circle as a vetical bar highlighted in different colors. Labels for each sample in phylogenetic tree were also highlighted with four other colors corresponding to groups as follows: black, natural *B. napus*; red, synthetic *B. napus*; yellowgreen, *B. olracea*; cyan, *B. rapa*

Nucleotide diversity (π) and fixation index (Fst) analyses were conducted for both the cp and mt genomes based on whole-genome variants. We found that each of the subgroups in the cp and mt genomes had similar and small mean nucleotide diversity. The highest diversity was 7.77 × 10^–5^ in *pol* of the mt genome, the lowest was 1.76 × 10^–5^ in *nap* of the cp genome (Fig. 3A,B). However, it was higher among all accessions, as the nucleotide diversity of the whole assembled cpDNA and mtDNA was 6.68 × 10^–4^ and 1.63 × 10^–4^, respectively. On the other hand, except group *cam* and *pol*, the genetic distance (Fst) between each two populations in the cp genome ranged from 0.810 to 0.916 and in the mt genome from 0.440 to 0.630 (Fig. 3A,B), showing a high degree of differentiation among these four cytoplasmic groups, which was more intuitively displayed in the PCA plot (Fig. 4A,D). The differentiation degree between *cam* and *pol* was lower than those between other groups in both mt and cp, indicating a close evolutionary origin.

The cp genomes exhibited smaller intra-group nucleotide diversity than those of the mt ones but had greater inter-group differences, implying that the differences in the cp genomes among different cytoplasmic types were reflected in single-base polymorphism, while a conservative genome structure was maintained, which was different from the mitochondrial genome.

The diversity along the organellar genomes in the four cytoplasm types (*nap*, *cam*, *pol,* and *ole*) was estimated based on 500-bp sliding windows and a 100-bp step size. Consistent results (*P* > 0.05 in *t*-test) between the subgroups were observed in the mt genomes, but significant differences existed in the cp genomes (*P* < 0.01 in *t*-test) (Fig. 3C,D). Several regions had higher polymorphism between two of the four subgroups. To detect those genomic regions, we calculated the reduction of diversity (ROD) [4] values based on the nucleotide diversity ratio of the whole group to *nap*, *cam*, *pol*, and *ole* using 500-bp windows (Supplementary Table S6). The regions with the top 1% ROD values were excluded. Continuous windows were then merged into 11 regions (Table 4).

**Table 4:** Significant difference genome regions of **c**hloroplast among different cytoplasm

Start	End	N_Variants	π (All samples)	π (*nap*)	π (*cam*)	π (*pol*)	π (*ole*)	Genes
26,001	26,800	16	3.27e-03	1.03e-03	1.95e-04	na ^a^	3.44e-04	*trnH, psbA*
30,001	30,700	38	4.77e-03	1.36e-05	7.09e-05	7.04e-04	na	*matK* (upstrean)
33,301	34,200	29	4.10e-03	7.75e-04	1.46e-04	na	2.82e-04	*psbI, trnS*
55,401	56,100	32	3.34e-03	na	na	na	na	*trnD*
81,501	82,100	19	3.79e-03	na	1.32e-04	na	na	*rbcL*
88,301	88,800	38	3.54e-03	na	2.64e-05	na	na	*petA* (upstream)
92,301	92,900	29	3.14e-03	9.50e-06	4.19e-04	na	na	*psaJ, rpl33*
107,301	107,800	15	3.94e-03	2.61e-05	7.76e-05	na	na	*rpl16*
138,901	139,600	34	3.25e-03	2.36e-05	na	na	na	*rpl32*
148,901	149,600	18	3.26e-03	2.71e-06	na	na	na	*rps15*
150,401	150,900	20	3.60e-03	na	na	na	na	*ycf1-2*

^a^ ‘na’ indicates no SNPs among the subgroup samples but several variants between two subgroups

### Genetic structure and phylogenetic relationship

To investigate the genetic structure and phylogenetic relationship between the *B. napus,* their diploid progenitors, and synthetic rapeseed organellar genomes, SNPs for 199 *B. rapa* and 119 *B. oleracea* accessions [46], together with 31 synthetic *B. napus* accessions [45] (11 accessions were from unpublished sequencing data) were detected by mapping reads to *B. napus* organellar pan-genomes. Next, we performed principal component analysis (PCA) as well as phylogenetic tree and population structure analyses after filtering sites with a minor allele frequency (MAF) < 0.02 in *B. napus*, but with a MAF < 0.005 in three species for preservation of the species-specific loci. Based on the PCA and maximum likelihood (ML) tree results, we founded that *Brassica* accessions with same cytoplasm types organelle genomes were clustered together and possessed nearly identical evolutionary positions in both the cpDNA- and mtDNA-derived trees but were not related to the ecotypes that were affected mainly by the differences in the climate zones and latitudes (Supplementary Figure S6). The correlation coefficient of cpDNA- and mtDNA-derived PC1 was 0.96 (Supplementary Figure S7), indicating that the cp and mt genomes in natural populations of *B. napus* evolved concurrently, as the PC1 variance proportion was 85.76% for cpDNA, and 51.76% for mtDNA.

The optimal number of population clusters of *B. napus* accessions was identified as *K* = 2 based on the results of its population structure which distinguish the *nap* and the other mitotype clusters (Supplementary Figure S8). However, in the PCA plot, the *B. napus* accessions further clustered into four groups by PC1 and PC2 (Fig. 4A,D), which exactly corresponded to the *nap*, *cam*, *pol*, and *ole* mitotypes. The *nap* and *cam* subgroups were in proximity at PC1 level in the cp PCA plot.

To investigate the domestication history of different cytoplasm types both within and between the three *Brassica* species, we constructed a tree incorporating accessions of the *B. rapa*, *B. oleracea*, synthetic and natural populations of *B. napus*. The mt and the cp trees showed similar population structures. The *B. napus* accessions diverged into three clades, revealing the multiply origin of *B. napus* (Fig. 4B,C). In a previous study, a few *B. napus* accessions were grouped with the majority of *B. rapa* [41]. Consistently, here, almost all *B. rapa* accessions were grouped closest to *cam* and followed by *pol* of *B. napus*, suggesting that the *cam* and *pol* cytoplasms of *B. napus* might be evolved from the ancestor of *B. rapa*. In addition, all the *B. oleracea* accessions were clustered in the *ole* clade, suggesting another independent cytoplasm originated from *B. oleracea* (Fig. 4B,C). The materials of the *nap* mitotype, predominant in natural *B. napus*, were not grouped together with the cluster of *B. rapa* or *B. oleracea* accessions. However, there was one *B. rapa* accession (SamA_055, ssp. *pekinensis*) and two synthetic *B. napus* accessions (ERR479628 and ERR479646), whose female parent were from *B. rapa* ssp. oleifera 4 × and *B. rapa* ssp*. rapa*, respectively, were clustered into the *nap* clade, while the other synthetic accessions, whose female parent was *B. rapa*, were clustered into the *cam* clade and the accessions, whose female parent was from *B. oleracea*, were clustered into the *ole* clade. Meanwhile, these three accessions were detected as *nap* cytoplasm based on MSS mapping, which supported the cluster assessment results. The aforementioned information indicated that the *nap* cytoplasm might have evolved from that of *B. rapa*, which had been considered controversial and ambiguous in past studies. To investigate the phylogenetic relationship more clear among different types of cytoplasm, we selected a small amount of representative accessions from each clade and constructed the phylogenetic tree (Figure S9).

## Discussion

MSS markers and mitotype-specific homologous ORFs detection was effective means for identification of cytoplasm type. In this study. we identified four cytoplasm types in 1,456 world-wide *B. napus* accessions, in which 18 accessions were classified as *ole,* whose genomes contained identical MSSs and ORFs but lacking double copies for genes specified in *ole*-type [49]. The *ole-*type (JF920286) mt genome was reported to be the largest in *Brassicaceae* species due to the duplication of a 141.8-kb segment [49], which resulted in different gene numbers of 18 ORFs. However, *B. oleracea* mt genomes were different due to structure variable and the 141.8 kb segment was not necessarily repetitive just like *B. oleracea* var. *Botrytis* [57]. As repeats were more susceptible to errors during the assembly, we used read mapping analysis to examine whether the absence of the 141.8-kb duplication was caused by an assembly defect in the 18 *ole*-type accessions. Reads used for assembly in each accession were mapped to the corresponding assemblies and the depth along reference was detected after removing the duplicates. The genomic depth curve of 18 *ole* accessions had uniform distribution along the whole genome, except for several small regions (< 3 kb) exhibiting significant higher depth, which were validated as homologous sequences of the cp genome, and two mitotype-specific regions were uncovered. Consistently with the other three groups (Supplementary Figure S1, S2), no long segment (> 10 kb) had a double coverage depth, revealing that mt genomes of *ole*-type in *B. napus* does not contain the repetitive 141.8-kb sequence.

We also detected the mitotypes for the accessions that were not assembled into a single and circular molecule by mapping MSSs to contigs. A few accessions were identified to possess infrequent mitotypes. For example, R4699 [15] had the *hau* cytoplasm, and other two genomes (R4580, R5025 [4, 15]) had the *ogu* cytoplasm. The identification of the cytoplasm type can be valuable for extensive application of CMS lines in heterosis-promoting hybrid breeding.

The origin and improvement processes of rapeseed remain unclear and confused despite extensive research has been done as no truly wild *B. napus* populations are known. *B. napus* was formed by hybridization of *B. rapa* and *B. oleracea*, in which the A subgenome was presumed to originated from European turnip, and C subgenome was still undetermined [4]. Recently, organellar resequnencing and analysis have been taken to investigate the original maternal progenitors of *B. napus*. In previous studies of organellar genomes of *B. napus*, the origin of the *cam*-type and *pol*-type were consistently considered to directly inherited from *B. rapa*, but *nap*-type was controversial and unascertained. It was supposed to have originated from *B. oleracea* as the *nap*-type is tightly clustered with a botrytis-type cauliflower *B. oleracea* in the phylogenetic tree [58]. Similarly, certain sparse C-genome wild species were speculated to have primarily contributed the *nap*-type cytoplasm and be the corresponding C subgenome to *B. napus* as the cluster of *nap* is inserted in the middle of a C-genome clade [27]. It was also supposed in our study that the *nap*-type was closest to *ole*-type in mtDNA-based phylogeny (Figure S9). Moreover, the *nap*-type *B. napus* was further considered to have been derived from an unidentified or lost mitotype of *B. rapa* because of its rich germplasm and mt genome evolution [49]. Based on a combination of chloroplast and nuclear genetic markers, the most prevalent chloroplast haplotype was found at low frequencies in *B. rapa* but was not present within the C genome accessions (wild and cultivated *B. oleracea* L. and related species) [1], which was the consistent discovery in our organelle resequncing project. In this study, we collected samples of large populations of *B. napus* and its two progenitor species (*B. rapa* and *B. oleracea*). Using MSS sequences detection and phylogenetic analysis, the predominant *nap*-type was detected in a small number of *B. rapa* and the synthetic *B. napus*, whose female parent was *B. rapa* for the first time. Thus, we speculated that the *nap*-type *B. napus* had been derived from an infrequent mitotype of *B. rapa* with direct evidence. Additionally, the presence of 53 *ole*-types in the *B. napus* population indicated that there also were germplasms at low frequencies whose maternal ancestor was C genome species in natural rapeseed.

## Conclusions

The organelle genomes are important for formation of cytoplasmic inheritance related agronomical traits and are effective means to investigate the maternal origins of rapeseed. In this study, the chloroplast and mitochondrial pan-genomes of rapeseed based on *B. napus* accessions collected globally was constructed and the genome-wide variances and diversity were identified, which provide materials for *Brassica* breeding by studying of cytoplasmic inheritance related traits and provide information to understanding the overall polymorphism of rapeseed organelle genomes. The phylogenetic tree derived of the *B. rapa*, *B. oleracea*, natural and synthetic populations of *B. napus* revealed the origins of different *B. napus* cytoplasm haplotypes*.* The identification of the *nap*-type in both the *B. rapa* population and the synthetic *B. napus* whose female parent was *B. rapa*, offered novel argument suggesting that *B. rapa* might be the maternal ancestor of *nap*-type *B. napus*. The cytoplasmic haplotypes identification and phylogeny provide novel insights into the organelle genomic evolution of *B. napus*.

## Materials and methods

### Samples and resequencing

A total of 1,579 rapeseed accessions originated from two published studies were used for constructing organelle pan-genomes and genetic diversity analysis in our research, of which a worldwide set of 991 germplasm accessions including 3 ecotypes (658 winter, 145 semi-winter, and 188 spring) from 39 countries were collected by the Leibniz Institute of Plant Genetics and Crop Plant Research (https://gbis.ipk-gatersleben.de/gbis2i/faces/index.jsf) in Gatersleben, Germany, and the Provincial Key Laboratory of Crop Gene Resources of Zhejiang University [15]. Another diversity panel comprised by 588 *B. napus* accessions (74 winter, 428 semi-winter, and 86 spring) includes 466 from Asia, 102 from Europe, 13 from North America, and 7 from Australia [4]. In addition, 31 synthetic *B. napus,* 199 *B*. *rapa*, and 119 *B*. *oleracea* accessions were also taken into the investigation of the phylogenetic relationships of *B. napus*, *B. rapa* and *B. oleracea.* The 199 *B. rapa* and *119 B. oleracea* accessions were from Plant breeding germplasm, Company and Genebank [46]. The synthetic accessions were developed by interspecific hybridizations between highly diverse parental origins, in which 20 accessions were conducted by Schmutzer T, et al. [45] and another 11 accession were sequenced by Key Laboratory of Biology and Genetic Improvement of Oil Crops, Ministry of Agriculture and Rural Affairs. Whole-genome DNA of all accessions was extracted from leaves and sequenced using next generation sequencing (NGS) technologies by abovementioned public studies. Detailed information of all the accessions is listed in Supplementary Table 7–9.

### Pan-genome assembly and annotation

The quality of the raw reads was checked and the low-quality regions were trimmed from leading and trailing side of reads by Trimmomatic (version 0.36, LEADING:3 TRAILING:3 SLIDINGWINDOW:4:15 MINLEN:120) [59]. Then, clean reads were mapped to a mitochondrial genome sequence cluster and a chloroplast genome sequence cluster respectively using BWA [60] of the Sentieon DNASeq software [61]. Both of the two data set contained the six *Brassica* species that comprising the Triangle of U [2] (Table S1). The alignment files were used for mapped paired-end reads extraction and de novo assembly. The cp genomes were assembled using the NOVOPlasty version3.3 [47] with a kmer value of 39 for the cp genomes and the mt genomes were assembled using ARC v1.1.4-beta (http://ibest.github.io/ARC/). The contigs assembled by ARC were first filtered with coverage > 30 and connected to single molecules by a local Perl script based on BLAST alignments.

The quality of the organelle genome assembly were evaluated based on gene and genome level, genes annotation revealed the completeness of the assemblies in terms of gene content. we then performed genome comparative analysis and pair-end reads mapping assessment. The assembled genomes were mapped to the aforementioned reference (public *Brassica* species organelle genome sequences) using BLASTN. Alignment with identity less than 90% was filtered and only subject with optimal alignment for each sample was retained. The high similarity between assembled genomes and corresponding reference genome revealed the completeness of the assembly. Pair-end reads used for assembly were mapped back to the assembled genomes using BWA [60] of the Sentieon DNASeq software [61]. After filtered reads with mismatch, deletion, insertion, and soft or hard clipping, we assessed the accuracy of the assembly by measuring the coverage and depth and detected whether there were breakpoints across whole genome.

The cp and mt pan-genomes were constructed based on the whole-genome alignment of all samples. First, all assembled genomes were aligned with each other by BLASTN (version 2.7.1 +) [50] (-E 1e-30), and the genome that had the largest number of high-quality alignments with other samples was chosen as a reference. All other assemblies were mapped to the chosen reference using Mugsy v1r2.3 [56]. Based on the Mugsy alignments, we detected the insertion fragments (> 10 bp) and the unanchored fragments (> 100 bp) using a local Perl script. The draft genome was edited by inserting the insertion fragments and was supplemented by unanchored fragments which were detected by at least two accessions.

To explore the gene and PAV information of the *B. napus* accessions, GeSeq [48] (search identity 85) was employed to annotate the organellar pan-genome and the genomes of all assembled samples using a gene set from the aforementioned *Brassica* species as a reference. The predicted fragmented genes with coverage or identity less than 60 were excluded. The genome maps were drawn using OGDRAW [62] and Circos v0.69–9 (http://circos.ca/).

### Identification of mitotypes

Twelve MSS markers developed by Heng et al. [55] and 34 homologous ORFs [49] inconsistent in six *Brassica* mitotypes were selected to differentiate the mitotypes of all assembled accessions. We searched the sequence of each of the MSS markers against genome assemblies through BLASTN [50]. Meanwhile, we searched the sequences against published genomes whose mitotypes were known and determined the filter threshold for each MSS, resulted a similarity threshold of 90% for MSS4, 85% for MSS9 and 80% for others. Along with the PAV information of the 34 homologous ORFs, data of associations between the accessions and the cytoplasmic fragments/ORFs were generated, and the mitotypes of the accessions with consistent corresponding relation were determined.

### Variant calling

The reads used for assembling were initially mapped to the assembled genomes for each sample, reads without any mismatch were selected and mapped to the organellar pan-genome. For accessions whose mt genomes were not assembled into single molecules, the reads were filtered by mapping to assembled contigs that were linked to the genomes of *Brassicaceae*. Bam files after removing the duplicates were taken into variant calling process using Haplotyper from Sentieon DNASeq [61] with options –emit_conf = 20, –call_conf = 20. Variants from *B. rapa*, *B. oleracea, B. napus* synthetic and natural groups were merged using bcftools v1.3.1 [63]. To decide the genotypes of all undetected loci for each sample, we performed haplotype-aware consequence calling using samtools v1.3.1 [51] and bcftools, and the genotype was set as N for loci in samples with low depth (depth <  = 30) and quality (Q < 20).

### Phylogenetic inference and population structure

SNPs with a minor allele frequency greater than 5% and less than 20% missing data were screened out for population structure and phylogenetic inference study. The maximum likelihood (ML) trees were constructed using IQ-TREE v1.6.12 [64]. A best best-fit model determined by ModelFinder [65] and specifying 1000 replicates was selected for the ultrafast bootstrap. The trees were displayed by an online tool Interactive tree of life (iTOL) v3 (https://itol.embl.de) [66]. Principal component analysis (PCA) among *B. napus* accessions and estimation of Fst were conducted by GCTA v1.25 [67]. The population structure was inferred by STRUCTURE v2.3.4 [68] which implements a model-based clustering method. Plots were generated with R package ggplot2 (https://cran.r-project.org/web/packages/ggplot2/index.html).

### Nucleotide diversity

Nucleotide diversity (π) and population fixation statistics (Fst) across three genetic clusters were calculated by vcftools v0.1.13 (https://vcftools.github.io) using a 500-bp sliding window with a 100-bp step. The reduction of diversity (ROD) [4] values based on nucleotide diversity ratio between nap, cam and pol were calculated to detect the differentiation regions of pairwise genomes.

### Supplementary Information

**Additional file 1.** Organelle pan-genome sequences of *Brassica napus.*

**Additional file 2.** Supplemetary figures.

**Additional file 3.** Supplemetary tables.

## Data Availability

The raw genome sequences of 1,579 natural *Brassica napus* can be found in GenBank under SRP155312, PRJNA358784 and PRJNA430009. The raw sequencing data of 20 synthetic *B. napus* accessions can be found in European Nucleotide Archive (https://www.ebi.ac.uk/ena/browser/home) under the project numbers PRJEB5974 and PRJEB6069. The raw sequences of *B. rapa* and *B. oleracea* can be found in the NCBI database under BioProject accession PRJNA312457. The obtained published cp and mt genomes of six *Brassica* species used for organellar genomes reads extracting were listed in Supplementary Table S1. The datasets of *Brassica* chloroplast and mitochondrial genome sequences generated during the current study are available at Mendeley Data (https://doi.org/10.17632/9g7kxvgnyr.1).

## Abbreviations

cp: Chloroplast
mt: Mitochondrial
PCA: Principal component analysis
MAFs: Minor allele frequencies
MSS: Mitotype-specific sequence
PAV: Presence-absence variation
