# Correction: A large-scale population based organelle pan-genomes construction and phylogeny analysis reveal the genetic diversity and the evolutionary origins of chloroplast and mitochondrion in *Brassica napus L*

**DOI:** 10.1186/s12864-023-09812-5

**Published:** 2023-11-27

**Authors:** Hongfang Liu, Wei Zhao, Wei Hua, Jing Liu

**Affiliations:** 1 Key Laboratory of Biology and Genetic Improvement of Oil Crops, Ministry of Agriculture and Rural Afairs, Oil Crops Research Institute of the Chinese Academy of Agricultural Sciences, Wuhan, 430062 China; 2 Hubei Hongshan Laboratory, Wuhan, 430070 China

**Correction**: *BMC Genomics***23**, 339 (2022)

10.1186/s12864-022-08573-x

Following publication of the original article [1], it was reported that there was a typographical error in the Given name of the first author, Hongfang Liu.

Hongfang Liu’s name was incorrectly published as Honfang Liu.

The original article has been updated.
