# Supplementary material for: A large-scale population based organelle pan-genomes construction and phylogeny analysis reveal the genetic diversity and the evolutionary origins of chloroplast and mitochondrion in Brassica napus L
Source: BMC Genomics. 2022 Apr 30;23:339. doi: 10.1186/s12864-022-08573-x (PMC9063048; doi:10.1186/s12864-022-08573-x)
Supplement: Supplementary file 1 — Additional file 1. Organelle pan-genome sequences of Brassica napus. [file 12864_2022_8573_MOESM1_ESM.docx]

**Additional file 1.** The chloroplast and [mitochondrial](F:/install%20files/Youdao/Dict/8.9.9.0/resultui/html/index.html#/javascript:;) pan-genome sequences of *Brassica napus*

>chloroplast genome

TTATTTCCATCTCGATTTTCAGAAACTGTTTTATAGTTATAGTATGTTCGAACGTGGAATTCATCATCCTTATTAATTTTGTCTTTTCCATTCACTCCGATTTCTTCCATTTCATCGATTTTGTCCAGATCCTCCCTTTCTTCCGAAAAAAGAGAAGGAAAAGGAGCTTCTTCGGTGGATACCTCTTGGTCCTGTTTAGTCCCACCCGTTTCTGAGGTTCCTTTTAGTTTCTTAGTAAAAATGGGTGATGGGATTCTGCCTAAATAGTAGACACAGGTAATAAATAAGATAATACTAAAGATTCGAGTCATAGAATTTCTCAATTCTGACACAAGGAACTTATACTTATTAGATCTAATAAGTACATTAGACCTAATAGAATTATTTTGCTGTATCCAGACTAATACCAATCCAACCCATTTCATGAATAAAATGTGACCAATTAACCAACCAACAAAACTACTTGTTACAAATAACATCTTGTTGTTGCATCGAAACATATAAATGTTGACTAATCTGGCTAACATTGAACTTGGTAAAATGAAATGGTTGAATAATTGAAAAATGAAATTATTCAGGAATACACATTGAATGCGAAGATTACGCATTTCATTTCTGGTAGTAGATCCATAATCAAAAAAGTGTTTGTGATTGTTCCAGAAGAAATGAAACAAAAGATACGGTAGAGCTAGGACAGTTATTGTATGAGGTCTACCCAATGCTAAATGCAGAGGCGCATAATAGATCGATATGAACATCATGAGCTGTCCCGCAATAAAACCAGTTGTTGCTGATACTTTCTTCTCGGTTCCTTCTTCTCCTTCGTCCATAACCCGAGCTCGGAGAAGGAAGAGATAAGAGGGCCCTATGGAGAATGTGGTCAGAAATCCATAATAGAGTCCGACCACAACGACCGAATTGATTATCTTCATGCATAAGGATACTAGATTACCTAGTATAAAAGATTGAAAAACCATCACAAACCTCCCTTTTTTCTTTTCTATTTCAATTTCTGGATTATTATATGATGATTTTGCAACTTTCCATATATAGAAATAGAAAGAGATAGACTAGAAACGACATCTCTTATGTCAATGACACCAAAGGGATATTAAATGAATGGAATTGGGATATGGATGGAATATAATGAAATAGAGCCGCTTTGAGGTTCCCTATGAAATGAGGCATGGAACGGAGCCACTACGAAGAAGTTCCGGGGGTTACGAAGGAAACTTCGAGTTCATATTGGTCATGGGTTGAGAACGGGAATTGAACTCTAGGAGATCTAATCTCCCGTTGTTCCTCAGTAGCTCAGTGGTAGAGCGGTCGGCTGTTAACTGATTGGTCGTAGGTTCGAATCCTACTTGGGGAGATTTGATTCATTCCGAATTCAAGAATTCAGAATGTAAGAGTAGGTAACCCGTTCCCTGTGTCTTTGTTTCTATTGCATTCTATCTCATCGTATCACATTCTGTTCTGTGATATTTGAGAATCACCGTCAATACCTCGGTGTAGGTCCGGGATAATCCTTTATTCCATAGTCCTGGGGCTATTTACAACTAGCCAATTCAGAATTTGCAGGTGTACTAACAAGTGCATCTTTGATGCAGTCATCGATTCTCCCGAGAGTTCACAATTACCGCGCGCAAACATATTCATTTAATGACTTAATGATGAGGAACGCATTTTTTCTATGCTACTAATACTTGTACTTGCTCTGCTATTCTGCCCAAGCCTGGCTGAGGAAGAGTTACGGGGCTTCGAAAAATATGCTGATTCGGCCGGTAATCATACTATATGTAAAAAAAAAGCGATAGATATAATAGATATATATATCTAAATTCAAATAAAAAAGAAGGCCACTCCACTCTATTTCGACAAAAGACCCATCCCCAACCAAGTTCCATAGCTTTGAGTCCGCTATCCCGAGCATGATTTTCCTACCCCCCGGAGGGAAAGGTCCTTCCCTTTTGGGCCGGTTGTGGGCGAGGAGGGATTCGAACCCCCGACACCGTGGTTCGTAGCCACGTGCTCTAATCCTCTGAGCTACAAGCCCCACCCCGTCTCCACTGGATCTGTTCCCAGGAGTACCCTACAAAAAAAGGAACCTTTCCTCTCCCCAGCCATTTCGGGTTAAGAAGATGTGAAAGTGCCTTTCTCTCTATAAGAACGGTGCGTTCCGGGGTGTGAAGTGGGATAGAAGGGATTTCATAATTGGGGTTTTGAATAAGACAACCTTTTCATTTTTCATTTTTTTTCAATATGAAAAAGTAATAAGAATGAGAGGTGTTAAGCTTTTCATCATCCTGGCGTCGAGCTATTTTTCCGCAGGACCTCCCCTACAGTATCGTCACCGCAGTAGAGTTTAACCACCAAGTTCGGGATGGATTGGTGTTGTTCCTCTACGCCTAGGACACCAGAATATCGAACCATGAACGAAGAAAGGCATGCGAGAAAAGCATATTGGCTAGTGATTGTGAGGCTCCAATTCTTGACTGGAGTGGACACCAAAGGCCTCCGCCCCTCCATCCTTTGGATAGAAGGGCAGAATTTTTGGTTTTTTCATGTTGTCAAAGAGTTGAACAATGGTTTTTTCGTGTTGTCAAAGAGTTGAACTATGAAAATAGATGGCGAGTGCCTGATCGAATTGATCAGGTCATGTAGGAACAAGGTTCAAGTCTACCGGTCTGTTAGGATGCCTCAGCTGCATACATCACTGCACTTCCACTTGACACCTATCGTAATGATAAACGGCTCGTCTCGCCGTGACCTTCTCTTGAATTCTCAAAACTTCTGTCACTCCATCCCCGCAGGGGCGGAGAACCCGTTGCTGTCTCGGCTGTGCTACCGGAGGCTCTGGGGAAGTCGGAATAGGAGAGCACTCATCTTGGGGTGGGCTTACTACTTAGATGCTTTCAGCAGTTATCCGCTCCGCACTTGGCTACCCAGCGTTTACCGTGGGCACGATAACTGGTACACCAGAGGTGCGTCCTTCCCGGTCCTCTCGTACTAGGGAAAGGTCCTCTCAATGCTCTAACGCCCACACCGGATATGGACCGAACTGTCTCACGACGTTCTGAACCCAGCTCACGTACCGCTTTAATGGGCGAACAGCCCAACCCTTGGAACATACTACAGCCCCAGGTGGCGAAGAGCCGACATCGAGGTGCCAAACCTTCCCGTCGATGTGAGCTCTTGGGGAAGATCAGCCTGTTATCCCTAGAGTAACTTTTATCCGTTGAGCGACGGCCCTTCCACTCGGCACCGTCGGATCACTAAGGCCGACTTTCGTCCCTGCTCGACGGGTGGGTCTTGCAGTCAAGCTCCCTTCTGCCTTTGCACTCGAGGGCCAATCTCCGTCCGGCCCGAGGAAACCTTTGCACGCCTCCGTTACCTTTTGGGAGGCCTACGCCCCATAGAAACTGTCTACCTGAGACTGTCCCTTGGCCCGTAGGTCCTGACACAAGGTTAGAATTCTAGCTCTTCCAGAGTGGTATCTCACTGATGGCTCGGGCCCCCCCGGAAGGAGGCCTTCTTCGCCTTCCACCTAAGCTGCGCAGGAAAAGCCCAAAGCCAATCCCAGGGAACAGTGAAGCTTCATAGGGTCTTTCTGTCCAGGTGCAGGTAGTCCGCATCTTCACAGACATGTCTATTTCACCGAGCCTCTCTCCGAGACAGTGCCCAGATCGTTACGCCTTTCGTGCGGGTCGGAACTTACCCGACAAGGAATTTCGCTACCTTAGGACCGTTATAGTTACGGCCGCCGTTCACCGGGGCTTCGGTCGCCGGCTCCCCTGTCATCAGGTCACCAACTTCCTTGACCTTCCGGCACTGGGCAGGCGTCAGCCCCCATACATGGTCTTACGACTTTGCGGAGACCTGTGTTTTTGGTAAACAGTCGCCCGGGCCTGGTCACTGCGACCCCCTTTGTGAGGAGGCACCCCTTCTCCCGAAGTTACGGGGCTATTTTGCCGAGTTCCTTAGAGAGAGTTGTCTCGCGCCCCTAGGTATTCTCTACCTACCCACCTGTGTCGGTTTCGGGTACAGGTACCCTTTTGTTGAAGGTCGTTCGAGCTTTTCCTGGGAGTATAGCATGGGTTACTTCAGCGCCGTAGCGCCTGGTACTCGAACATTGGCTCGGGGCATTTTCTCTACCCCTTCTTACCCTGAAAAAACAGGGACACCTTGCGTTCTTGAACCGATAACCATCTTTCGGCTAACCTAGCCTCCTCCGTCCCTCGGGACCAACAAGGGGTAGTACAGGAATATTCACCTGTTGTCCATCGACTACGCCTTTCGGCCTGATCTTAGGCCCTGACTCACCCTCCGTGGACGAACCTTGCGGAGGAACCCTTGGGTTTTCGGGGCATTGGATTCTCACCAATGTTTGCGTTACTCAAGCCGACATTCTCGCTTCCGCTTCGTCCACCGCCGCTCGCGCGGTTGCTTCCCCCTAAGGCGGAACGCTCCCCTACCGATGCATTTTGACATCCCACAGCTTCGGCAGATCGCTTAGCCCCGTTCATCTTCGGCGCAAGAGCGCTCGATCAGTGAGCTATTACGCACTCTTTCAAGGGTGGCTGCTTCTAGGCAAACCTCCTGGCTGTCTCTGCACCCCTACCTCCTTTATCACTGAGCGGTCATTTAGGGGCCTTAGCTGGTGATCCGGGCTGTTTCCCTCTCGACGATGAAGCTTATCCCCCACCGTCTCACTGGCCGACCTTGACCCCAGTTATTTTGAGGTCATATCTAGTATTCAGAGTTTGCCTCGATTTGGTACCGCTCTCGCGGCCCGCACCGAAACAGTGCTTTACCCCTAGATGTCCAGTCAACTGCTGCGCCTCAACGCATTTCGGGGAGAACCAGCTAGCTCTGGGTTCGAGTGGCATTTCACCCCTAACCACAACTCATCCGCTGATTTTTCAACATCAGTCGGTTCGGACCTCCACTTAGTTTCACCCAAGCTTCATCCTGGTCATAGATAGATCACCCAGGTTCGGGTCCATAAGCAGTGACAATTGCCCTATGAAGACTCGCTTTCGCTACGGCTCCGGTGGGTTCCCTTAACCAAGCCACTGCCTATGAGTCGCCGGCTCATTCTTCAACAGGCACGCGGTCAGAGCCCAGGGCTCCTCCCACTGCTTGGGAGCTTACGGTTTCATGTTCTATTTCACTCCCCGATGGGGGTTCTTTTCACCCTTCCCTCACGGTACTACTTCGCTATCGGTCACCCAGGAGTATTTAGCCTTGCAAGGTGGTCCTTGCTGATTCACACGGGATTCCACGTGCCCCATGCTACTCGGGTCAGAGCATAAGCTAGTGATGCTTTCGGCTACTGGACTCTCGCCATCTAGGGTGCGGCACTCCACCGCTTCGCCTAGCAGCACGACGCTTTTATTGCTCTCCCACAACCCCGTTTTCACGGTTTAGGCTGCTCCCATTTCGCTCGCCGCTACTACGGGAATCGCTTTTGCTTTCTTTTCCTCTGGCTACTAAGATGTTTCAGTTCGCCAGGTTGTCTCTTGCCTGCCCATGGATTCAGCAGCAGTTCAAAAGGTTAACCTATTCGGGAATCTCCGGATCTACGCTTATTTTCAACTCCCCGAAGCATTTCGTCGCTTACTACGCCCTTCCTCGTCTCTGGGTGCCTAGGTATCCACCGTAAGCCTTTCCTCGTTTGAACCTCGCTCTTAACTTTAAGGCTATGCCATCCTAAGGTGCTGCTAAATGGAAAGATCTTATCAACGTCCATGAATGAAAAATAATAGATCGAACTGCCGAATCGGAAAAATTGGGTGCTATCATATAGCTTTGTATCGGCTAAGTTCACGAGTTGGAGATAAGCGGACTCGAACCGCTGACATCCGCCACAGGGTAAACCACCGCCTATCAGGCCCCTGACTGATTCTACCATAGAGGCCAACGATAGACAAGAACTCCCCCCCGAACACAGCTTACAACTTTCATCGTACTGTGCTCTCCAAAGAGCAACTCTTCTCAAAATCTCAAAAGGTGATGAGTTGGAATCCCATTCCAACTCAGGATTCTTGTGGTTCGAGAGGATCCAGCTACAGGAGAACCAGGAACGGAGAGCTTTCCCCCCTTTTCCGCCCCAACTCTTTCGAATGCTGGTTTTAAGAATGAGTGATTGCCCTTCTCCGACCCTTACTGCCCAACCTGCGAGCGGACAGCTAATGCATTCCACTTATTGAACAGGGTTCTATGGTCGGTCCGCGACCCCTGGATACCGAAGGCGTCCTTGGGGTGATCTCGTAGTTCCTACGGGGTGGAGACGATGGGGTCGGTCCATGGATTTTCCTTCCTTTTGCCGCATTTCGCTCAAAGGGTTGAAGGGAGATAGTGCATCAAGCTGTTCGCAAGGGCCAACTTGATCCTCTTCCCCAGGGATCAATCCCAGACGAGGGAACCCTAGGAGAGCCGCCGACTCCAACTACCGTCCATGTACGATCCATACTAGATCTGACCAACTGACCATCCTACCTCCTCTACGTTCTTGACAGCCCATCTTTGTCTCAGTAGAGTCTTTCAGTGGCATGTTTCGGTCCTTTTCCCCATTACTTAGAAAAAGTGAGCCACCGGTTCAGGTACAAGATACTATCATTACCGCCTGGACAATTAGACACCCAACCCGTAATCGCAACGACCCAATTGCAAGAGCGGAGCTCTACCAACTGAGCTATATCCCCCCGAGCCAAGTGGAGCATGCATGAAGGAGTCAGATCCTTCTTATATTCTTTTCCTTGGCGCAGCTGGGCCATCCTGGATTTGAACCAGAGACCTCGCCCGTGAAGTAAATCATCGCACCTATGGTCCAACCAATTGGGAGAGAATCAATAGATTCCTTTTCGGGAGCGATTCATCCTTCCCGAACGCAGCATACAACTCTCCGTTGTACTGCGCTCTCCAAGTGTGCTTGTTCCCCCCCTCTTCCTTACCATAGGAAGTATTTGTGAAATAACTTCGATGAGAAGAAAAAAGAGGGCGTTAAGAGACCCTACTGGCCTAACCCTAGACACTCTAAGATCCTTTTTCAAACCTGCTCCCATTTCGAGGCGGAAAGGAAAAAGAATTTCACGTTCTTCCTTTCGGGAAGGGAGGATTAGGAAAATCCTATTGATTGCAGCTTTCTCCAGACCCCTGGGAAAAGCATGAAAAAAAGGCTCGAACGGTACGATCCCTCCGTCACCCCAGAATGAAAGGGGTGATCTCGTAGTTCTTGGTCTGTGAAGATACGTTGTTAGGTGCTCCATTTTATTTTCCCATTGAGGCCGAACCTAAACCTGCGCTCGAGAGATAGCTGTCCATACACTGATAAGGGATGTATGGATTCTCGAGAAGAGAGGAGCCGTGGTGGTCCCCTCCGGACCGCCCGGATCCCACGAGTGAATAGAAAGTTGGATCTACATTGGATCTCACCTGAATCGCCCCATCTATCCTCCTGAGGAGAAGTTTGGTTTCAAACCCCGGTTCGAACAGGAGGAGTACGCCATGCTAATGTGCCTTGGATGATCCACATCTCAGGGTCAGGCGCTGATGAGCACATTGAACTATCCATGTGGCTGAGAGCCCTCACAGCCCAGGCACAACGACGCAATTATCAGGGGCGCGCTCTACCACTGAGCTAATAGCCCGTCGTGCGGGCCTCCTGCTGGGGGCCCGCTATGCCAAGCCAAAAGCGAGAGAAACCCCATCCCTCTCTTTCCTTTTTACGCCCCCCTGCCGCCACACGAGAGGGACATGGGGGCGTAAAAGGGGATCCTATCAACTTGTTCCGACCTAGGATAATAAGCTCATGGGCTTTGGGTTTGAAGCTGTGTCAAACCTAAATACCCAAGAAGCATTAGCTCTCCCTGAAAAGGAGGTGATCCAGCCGCACCTTCCAGTACGGCTACCTTGTTACGACTTCACTCCAGTCACTAGCCCTGCCTTCGGCACCCCCCTCCTTGCGGTTAAGGTAACGACTTCGGGCATGGCCAGCTCCCATAGTGTGACGGGCGGTGTGTACAAGGCCCGGGAACGAATTCACCGCCGTATGGCTGACCGGCGATTACTAGCGATTCCGGCTTCATGCAGGCGAGTTGCAGCCTGCAATCCGAACTGAGGACGGGTTTTTGGAGTTAGCTCACCCTCGCGGGATCGCGACCCTTTGTCCCGGCCATTGTAGCACGTGTGTCGCCCAGGGCATAAGGGGCATGATGACTTGACGTCATCCTCACCTTCCTCCGGCTTATCACCGGCAGTCTGTTCAGGGTTCCAAACTCAACGGTGGCAACTAAACACGAGGGTTGCGCTCGTTGCGGGACTTAACCCAACACCTTACGGCACGAGCTGACGACAGCCATGCACCACCTGTGTCCGCGTTCCCGAAGGCACCCCTCTCTTTCAAGAGGATTCGCGGCATGTCAAGCCCTGGTAAGGTTCTTCGCTTTGCATCGAATTAAACCACATGCTCCACCGCTTGTGCGGGCCCCCGTCAATTCCTTTGAGTTTCATTCTTGCGAACGTACTCCCCAGGCGGGATACTTAACGCGTTAGCTACAGCACTGCACGGGTCGATACGCACAGCGCCTAGTATCCATCGTTTACGGCTAGGACTACTGGGGTATCTAATCCCATTCGCTCCCCTAGCTTTCGTCTCTCAGTGTCAGTGTCGGCCCAGCAGAGTGCTTTCGCCGTTGGTGTTCTTTCCGATCTCTACGCATTTCACCGCTCCACCGGAAATTCCCTCTGCCCCTACCGTACTCAAGCTTGGTAGTTTCCACCGCCTGTCCAGGGTTGAGCCCTGGGATTTGACGGCGGACTTAAAAAGCCACCTACAGACGCTTTACGCCCAATCATTCCGGATAACGCTTGCATCCTCTGTATTACCGCGGCTGCTGGCACAGAGTTAGCCGATGCTTATTCCCCAGATACCGTCATTGCTTCTTCTCTGGGAAAAGAAGTTCAGGACCCGTAGGCCTTCTACCTCCACGCGGCATTGCTCCGTCAGGCTTTCGCCCATTGCGGAAAATTCCCCACTGCTGCCTCCCGTAGGAGTCTGGGCCGTGTCTCAGTCCCAGTGTGGCTGATCATCCTCTCGGACCAGCTACTGATCATCGCCTTGGTAAGCTATTGCCTCACCAACTAGCTAATCAGACGCGAGCCCCTCCTCGGGCGGATTCCTCCTTTTGCTCCTCAGCCTACGGGGTATTAGCAGCCGTTTCCAGCTGTTGTTCCCCTCCCAAGGGCAGGTTCTTACGCGTTACTCACCCGTCCGCCACTGGAAACACCACTTCCCGTCCGACTTGCATGTGTTAAGCATGCCGCCAGCGTTCATCCTGAGCCAGGATCGAACTCTCCATGAGATTCATAGTTGCATTACTTATAGCTTCCTTCTTCGTAGACAAAGCTGATTCGGAATTGTCTTTCATTCCAAGTCATAACTTGTATCCATGCGCTTCATATTCGCATGGAGTTCGCTCCCAGAAATATAGCTACCCCTACCCCCTCACGTCAATCCCACGAGCCTCTTATCCATTCTTATTCGATCACAGCGAGGGAGCAAGTCAAAATAGAAAAACTCACATTCATTGGGTTTAGGGATAATCAGGCTCGAACTGATGACTTCCACCACGTCAAGGTGACACTCTACCGCTGAGTTATATCCCTTCCCCCATCAAGAAATAGAACTGACTAATCCTAAGTCAAAGGGTCGAGAAACTCAAGGCCACTATTCTTGAACAACTTGGATTGGAGCCGGGCTTTCCTTTCGCACTATTACGGGTATGAAATGAAAATAATGGAAAAAGTTGGATTCAATTGTCAACTACTCCTATCGGAAATAGGATTGACTACGGATTCGAGCCATAGCACATGGTTTCATAAAACCGTACGATTCTCCCGATCTAAATCAAGCCGGTTTTACATGAAGAAGATTTTACTCAGCATGTTCTATTCGATACGGGTAGGAGAAACGGTATTCTTTTCTTAAACTTCAAAAAATAGAGAAATCAGAACCAAGTCAAGATGATACGGATTAATCCTTTATTCTTGCGCCAAAGATCTTCCTATTTCCAAAGGAACTGGAGTTACATCTCTTTTCCATTTCCATTCAAGAGTTCTTATGTGTTTCCACGCCCCTTTAAGACCCCGAAAAATTAACAAATTCCCTTTTCTTAGGAACACGTGCGAGATAAAAAAAAAAAGAGAGAATGGTAACCCCACGATTAACTATTTCATTTATGAATTTCATAGTAATAGAAATACATGTCCTACCGAAACAGAATTTGTAACTTGCTATCCTATAATCTTGCCTAGCAGGCAAAGATTTCACTCCGCGAAAAAGATGATTCATTCGGATCAACATGAAAGCCCAACTACATTGCATTGCCAGAATTCATGTTATCTATTGGAAAGAGGTTGACCTCCTTGCTTCTATGGTACAATCCTCTTCCCGCTGAGCCTCCTTTCTTCCGTGATTAACTGTTGGCACCAGTCCTACATTTTGTCTCTGTGGACCGAGAAGAAAGGACTCACTGCGCCAAGATCACTAACTAACACTAATCTAATAGAATAGAAAATCCTAATATAATAGAAAAGAACTGTCTTTTCTGTATACTTATGTATACTTTCCCCGGTTCCGTTGCTACTGCGGGCTTTACGCAATCGATCGGATCATCTAGATATCCCTTCAACACAACATAGGTCGTCGAAAGGATCTCGGAGACCCGCCAAAGCACGAAAGCCAGGATCTTTCAGAAAATGAATTCCTATTCGAAGAGTGCATAACCGCATGGATAAGCTCACACTAACCCGTCAATTTGGGATCCAATTCGGGATTTTCCTTGAGGGATATTGGTAAGGAATTGGAATGTAATAATATCGATTCATAATGGATTCATATCGATACAGAAGAAAAGGTTCTCTATCGATTCAACAAGTGCTGTACTTATGGGAAAGCGATAGAGAAAGAGAAAAAAAAAAACGAAGATTTCACATAGTGATTTTTTTTTGATCAAAAAAAAATATGATTGAATTTATTTCGTACCCTTCGCTCAATGAGAACATGGGTCAGATTCTATAGGATCAAACCTATGGGACTTAAGAATGATGGAAGGGAATAAAATCAAAAAAGAAATCAAATAAAGAAAAGAGAGGGAAAATAAAGAAATAATAAGTAAATAAAAATGAAGTAGAAGAACCCAGATTACAAATGAACAAATTCAAACTTGAAAAAGTCTCTTTCTGATTCTCGAAGAATGAGGGGCAAAGAGATTGATCGAGAAAGATCTCTTGTTCTTATTATAAGATCGTGTGATTGGACCCGCAGATGTTTGGTAAAAAGAATAATCTTATCCTTTGAGAATAATCAAAAATAGAAAGTGTTCAATTGGAACATGAAAACGTGACCGAGTTTATCCTAGTTACTCTTCGGGACGGAGGAGATTCGCGAACGAGGAAAGGGACCCAATGACTTCGAAAGAATTGAACGAGGAGCCGTATGAGGTGAAAATCTCATGTCCGGTTCTGTAGAGTGGCAGTAAGGGTGACTTATCTGTCAACTTTTCCACTATCACCCCCAAAAAACCAAACTCTGCCTTACGTAAAGTTGCCAGAGTACGATTAACCTCGGGATTTGAAATCACTGCTTATATACCTGGTATTGGCCATAATTTACAAGAACATTCTGTAGTCTTAGTAAGAGGGGGAAGGGTTAAGGATTTACCCGGTGTGAGATATCACATTGTTCGAGGAACCCTAGATGCTGTCGGAGTAAAGGATCGTCAACAAGGGCGTTCTAGTGCGTTGTAGATTCTTATCCAAGACTTGTATCATTTGATGATGCCATGTGAATCGCTAGAAACATGTGAAGTGTATGGCTAACCCAATAACGAAAGTTTCGTAAGGGGACTGAAGCAGGCTACCATGAGACAAAAGATCTTCTTTCAAAAGAGATTCAATTCGGAACTCTTATATGTCCAAGGTTCAATATTGAAATAATTTCAGAGGTTTTCCCTGACTTTGTCCGTGTCAACAAACAATTCGAAATGCCTCGACTTTTTTAGAACAGGTCCGGGTCAAATAGCAATGATTCGAAGCACTTATTTTTACACTATTTCGGAAACCCAAGGACTCAATCGTATGGATATGTAAAATACAGGATTTCCAATCCTAGCAGGAAAAGGAGGGAAACGGATACTCAATTTAAAAGTGAGTAAACAGAATTCCATACTCGATTTCATAGATACATATAGAATTCTGTGGAAAGCCGTATTCGATGAAAGTCGTATGTACGGTTTGGAGGGAGATCTTTCATATCTTTCGAGATCCACCCTACAATATGGGGTCAAAAAGCCAAAATAAAAGATTTGAGCCCTTATAAAAAGAAAACAGATTCTTGAACCCCTTTCACGCTCATGTCACGTCGAGGTACTGCAGAAGAAAAAACTGCAAAATCCGATCCAATTTATCGTAATCGATTAGTTAACATGTTGGTTAACCGTATTCTGAAACACGGAAAAAAATCATTGGCTTATCAAATTATCTATCGAGCCTTGAAAAAGATTCAACAAAAGACAGAAACAAATCCACTATCTGTTTTACGTCAAGCAATACGTGGAGTAACTCCCGATATAGCAGTAAAAGCAAGACGTGTAGGCGGATCAACTCATCAAGTTCCCATTGAAATAGGATCCACGCAAGGAAAAGCACTTGCCATTCGTTGGTTATTAGGGGCATCCCGAAAACGTCCGGGTCGAAATATGGCTTTCAAATTAAGTTCCGAATTAGTGGATGCTGCCAAAGGGAGTGGCGATGCCATACGCAAAAAGGAAGAGACTCATAGAATGGCAGAGGCAAATAGAGCGTTTGCACATTTTCGTTAATCCATGAACAGGATCTATATAGACACATAGATCCGTGGATCCATACATCTCGATCCGAAAAGAATCAATAGAAAAAGAAAAAATCGGAATTGATCGATCTCTTTCTCGAAACAAACGAAAAGGAAAGAAAAGACGAAACATAAATCATGGATCAACTAAGCCCTCTCGGGGACTTGCTTAAGAATAAGAAAGAGCAATCTCATGTAAATACCATGGAATAAGGTTTTAACCTATTCATGGGGATTCCGTAAATATTCCATTCAAAAAAAAAAAAATTGGTTTTTTTTTGGAGATTGGATGCAGTTACTAATTCATGATCTGGCATGTACAGAATGAAAATTTCATTCTCGATTCTACGAGAATTTTTATGAAAGCCTTTCATTTGCTTCTCTTCGATGGAAGTTTTATTTTCCCAGAATGTATCCTAATTTTTGGCCTAATCCTTCTTCTGATGATCGATTCAACCTCTGATCAAAAAGATATACCTTGGTTATATTTCATCTCGTCAACAAGTTTCGTAATGAGCATAACGGCCCTATTGTTCCGATGGAGAGAAGAACCTATGATTAGCTTTTCAGGAAATTTCCAAACGAACAATTTCAACGAAATCTTTCAATTTCTTATTTTACTATGTTCAACTCTCTGTATTCCTCTATCCGTAGAGTACATTGAATGTACAGAAATGGCTATAACAGAGTTTCTGTTATTCGTATTAACAGCTACTCTAGGAGGAATGTTTTTATGTGGTGCTAACGATTTAATAACTATCTTTGTAGCTCCAGAATGTTTCAGTTTATGCTCCTACCTATTATCTGGATATACCAAGAAAGATGTACGATCTAATGAAGCTACTATGAAATATTTACTCATGGGTGGGGCAAGCTCTTCTATTCTGGTTCATGGTTTCTCTTGGCTATATGGTTCATCCGGGGGAGAGATTGAGCTTCAAGAAATAGTGAATGGTCTTATCAATACACAAATGTATAACTCCCCAGGAATTTCAATTGCGCTTATATTCATCACTGTAGGAATTGGGTTCAAGCTTTCCCTAGCCCCTTCTCATCAATGGACTCCTGACGTATACGAAGGAGTGCGGTTCGTTTGAGAAATTCCTACCTCTCTATCTATCTCTGAGATGTTTGGATTTTTCAAAACTCCATGGACATGCAGAAGAGAAATGCTATCCCCACGCAGACCAAGACAGAACTTTGACTTGTTCAAATAACAATTAATGTGAAGCAGGGTCAGGAACAACGAATCTCTTTATGATAAACGGATCCATTTTGCAAGTTTGTTATTACGGGTAGTTCCTACAAAGGATCGGACTAATGACGTATACAAGAAAGACTTGAATTCTCGATGTAGATGCTACATAGTTGGTTCTCATCCTTCAGAGACTACGAGTGTAATAGGAGCATCCGTCGACAAAAGGATCACCCTAAGATGATCATCTCATGGCTATTGAGAACGAATCAAATCAGATGGTTCCATTTCTCAATCTTTCGGACGTGCTCCTACGGAACCAAGGTCGAAACGATTGAGAAAAATCAGTCATTCACAACCACTGATGAAGGATTCCTCGAAAAGTTAAGGATTAGTAATCCGTTTTAGAAAGGATTCGATCTTATACATACGCGAGGAAAGTAATCAAAAAAGAAAGAAGATGAGTTCTTCTTTACTTTTATCACTTAGGAGCCGTGCGAGATGAAAGTCTCATGCACGGTTTTGAATGAGAGAAAGAAGTGAGGAATCCTCTTTTCGACTCTGACTCTCCCACTCCAGTCGTTGCTTTTCTTTCTGTTACTTCGAAAGTAGCTGCTTCAGCTTTAGCCACTCGAATTTTCGATATTCCTTTTTATTTCTCATCAAATGAATGGCATCTTCTTCTGGAAATCCTAGCTATTCTTAGCATGATATTGGGGAATCTCATTGCTATTACTCAAACAAGCATGAAACGTATGCTTGCATATTCGTCCATAGGTCAAATCGGATATGTAATTATTGGAATAATTGTTGGAGACTCAAATGGTGGATATGCGAGCATGATAACTTATATGCTGTTCTATATCTCCATGAATCTAGGAACTTTTGCTTGCATTATATTATTTGGTCTACGTACCGGAACTGATAACATTCGAGATTATGCAGGATTATACACAAAAGATCCTTTTTTGGCTCTCTCTTTAGCTCTATGTCTCTTATCCCTAGGAGGTCTTCCTCCACTAGCAGGTTTTTTTGGAAAACTCCATTTATTCTGGTGTGGATGGCGGGCAGGCCTATATTTCTTGGTTTCAATAGGACTCCTTACGAGCGTTCTTTCTATCTACTATTATCTAAAAATAATCAAGTTATTAATGACTGGACGAAACCAAGAAATAACCCCTCACGTGCGAAATTATAGAATATCCCCTTTAAGATCAACCAATTCCATCGAATTGAGTATGATTGTATGTGTGATAGCATCTACTATACCAGGAATATCAATGAACCCGATTATTGCGATTGCTCAGGATACCCTTTTTAGCTTCTAGAATCTATTTCTTAGTTCAAGATCCCTCTTACTAACTGGAATCAAAGAATTAGTAGATCGGTTCCGCCCAAAATGGGAATGGACTAAGGTTATGAACTTATAATCTATAATCTGATGATCGAGTCGATTCCATGATTATAAGTTCATTCCATACCGGACCAGACCGGAATAAGGTTATATACATTCTCATTATGAGAAGGGGTCATTCGAGCGTATCTAAATAGATACTATGTTTACATAGGGATCCCTACGTCGTTACATTCCATTTAGGATTAGGAATAGGCGAAATCTGACCTACTTTTTACATATCTCTCGTTATTTGGGACCCTATTCACCTCTTTGGTTGGACTTCTATTGAATCGAGAAATAGGTTTGATTGTCCATCTTTTTGATATAATATTAATATATATATAAGGCATCCTCCGGATAAGGATAATTCAAATCTAAGCAATTAGATGTCCGACTCGGGCCTATATGACATGACCGATCAATAGAAATACTTCAACACTCCACCTTTGTCATATATTCAATACACCGTACTAGATAGATATCATATTTATGGAATACGATTCACTTTCAAGATGCCTTGGTGGTGAAATGGTAGACACGCGAGACTCAAAATCTCGTGCTAAAAAGCGTGGAGGTTCGAGTCCTCTTCAAGGCATAATATTGAAATGGAATAAGTTCGGCAGCGGATCGCGAAATCTTGGCGATCTTCTCTATCTAATGAATGGGGAGGGGGAGTCCGCTTTGAAATCGTCCGCCCTGCGCCCCGCAGTATATGATTCAACAGGAATCACACAAGGGTAGATTGATACAATCTAAACCTCTGGTAAAATGCCCCCGTAACCCAGCAGATAAAGTACATAGTCCGTTTTAGGGATTGGTGACTTACCCATTCAGTGACTTTGGCACTGGATGGACGTTACCAAAATTGGTACTATCGGGTCGGGTGAATTCAATAATAGACGCCTGGCGGCATTCCAGCCTTCCTTCTCCTTTCAGGACCTATCCTAAACAGAATCCAGTACTTCTTGGTCGTGAATATCTGAATAGGGCGAACCACTCCGTGGATATCTTTACTTCGGAACAAAACAATTAGAATTAGGCTCGGTCAACTGGAATGTGTATTATCCATATAGGGGATCTTCCAATTGAGAAGATCTATCGACCTGAGACGAAGAGAAAGGTCTATCTATTTTATTTAGTTATTCAGTTGATTCGTTATTGGAACAGATAGCAACAACAATTTCATCCGACATGCGTATTTTTGATTTTCCAATGGATTTCCATCCTTCATTAATGGAAATTTTTTTGATGTAGTGAGTAATAGCTCTGGTTGTTCGCTGTTCAAGAATTCTTGTTTAGGCAGTTCGTACCATCCATACATAGTGTTTTGATCTAAGATTTCAATTCTTCCATGTTTCCGTCGTAGCATATTGTTCCATGGAGCTAAGTGGAAGAAACAGGTGTTTCTACAACTCTACCACCCAGTCAATTCCGTTCCACTTAATCCCTATTTCATGGACACATATCTTTCCGGCTAAGTAATGGGAAACCTTTCTCCTGTTACATTACATGAATCCTATTTTCATTTCATCCGGAAAAAGCCATCTTTTTTTCAACAATGTCTTTGTCATTCGATCCACTAGCGTTCCGTTAGATAGGAACAGATTTGATAAATACTGATAACTCTCGGATAGAGTATTAGAACGGAAAAATCCATTAGATAATGAACTATTGGTTCTAAGCCATCTCTGGCGCTGAATCAACAATTCGAAGTGCTTTTCTTGCGTATTCTTGATAAACCAGCGTTTATATATAGATGTAGGAGGATCTGTTTGGGAAGTAAGAAGCCCCTTTGACATCTCTTCATCTGCAAAGAATTCTCGATGTGAAAACACAGAGACAAAGGGCTGATCTTTGAATAGGAAAAAGAGTGGATCTGCGGGGTCCCAAATGAATTGGCTTATTCTAAAAAAGCCTTGTTCTTTGGAAGACCTATCTCGTCTCTGGTACTGCATGGTTCCGCTCTGCAAGAACTCCGAATCATTCTCTTGAAGCTCATACTTTTCATCATAAATGATCCGCTTGCCCCGAAATGACCCGGCCAAATAGGGAAATCCCAATTCATTAGGCCTTTCGATACAATCAAATAGAAAGCCCCGAGGGCGCCATATTCTAGGAGCCCAAACTATGTGATTGAATAAATCCTCCTCTATCTGTTCCGGGTCGAGGACTGCTTCTCCTTCCCCTTCTTCAAACTCCGATTCGTATTTTTCATAGAGAAATCTCTGATCAACGATAGAACAAGATCCATCTTGCATCATATATAAGGGATCCCTTGGTTCGGAGCGAAAAAGCAATGTCACTCGATCATTATCAAACTGACTGCAATCTTTTTCTGTCCGTGAGGATCCCACCAAAGCGCCTTGCACTTCTAATAGGCCATGAAATAGATCCGAATCATTCTCAATGAATCCATAAGAAGTGATCCTATTTTTTTCATCGGGTCCGGGTAGAGACCAAAGGTCTTGAGCGACCGATCCGGCAGAACAACTCAAAAGATAAAGAAGTATCGTGAATTTCTTCATGCTCGTTCCAAGTTCGAAGTACCATTTGTACAAATAAGAATCCCCTTCGTTACATGATTTCTTCTTCATATAGATAGATATAGGATCTATGGGGCAATTACTTATAAGTACATTTTGTGCAACAACCCTTCCTATCTGATAGAAAAGGATCCCATGATCCTGAACCGATCTTACCTGGGATCGCAAATCCCAAGTTTGTCTATGAAGAGCAGATCTAATTGTATTAGTGTCTATAATTGATTTCTTCTGTGTAATACTAATTGATAAGGCCTCATTGGTAAGTGCTACAAGATCTCGTGCACTGGAACCCATGGTTATGGACTCGAATCCATTAGTATGGAACATTTTCTTTTCCAAGTGAAATCCCCTAGTATAGGAAAGAGTGAAAAAGTGCTTTCGTTGTTGTGGAATAAGAAGCCTTCTTATTTTAATGCATGTATTTAATTTATTCGGGGCTATTAGAGCGGGATCCACTTTTTGGGGAATATGAGTCGAAGCAATAACAAGACTATTTCTAGTCGAACATCTTTCACAATCCCTGGAGAGAGAGTTCACCAAGAGACCGAGGGCTAAGTAATTCGACTCATTCACATCAAGATCATGAATGTTTGGAATCCATATTATGCAAGGAGACATTGCTTTTGCTAATTCGAATTGAAGGGTGATATAAAATCGGTCTATTTCCGACATCATATCCATAGTTAGCGCATTCATCATAGTTAGAAGCTCCAGCTCCGTATCAAGTTCACGATCAATATCGTTACTAGCATCAATATCGTCACTATCATCAATATCGATATCATCAAGAAAAAAACCTTTCGGCTTGTTATCCAGGAACTTGTTCAGACATACTGTAATGAAAGGAACATAGGAGTTTGTCGCTAGGTATTTGACCAAATAGGATCGTCCGGTTCCTATAGAACCTATCACTAAAATACTCCTAGAGGGGGATAGGGCTAAGCGGAGCGAAAAGGGTTTTCCATGAGACGGGAAATGAAAACTATTAGCCCCACACGAAGTTTGTGAATAAGTGATTGTCTGATAATGAGCAAGGAATATCCGTCTTTCTGCTAAACAGGATGTATTGAACTCATAATTCATTAGATACTTTTTATGAATGTCAACTAAGTATCGTAAGTAAATTGTTCCCGGTTGTTCAATCATTTGATAACCAGAGTCATTCTTTGATAAATGATCACTATGAGTCAGACTCAATAGAATTTGATCAATCCTTTTTTCTGCCCTTAAGGTGGAGAACTGAACCAAGAATTCTCTTTCTTTATCATCAATCGAATCACTGTTCGCGACCCAGGATTCTATTTTATCATCAATCCAATCACCGCTCACGTTTTTTCTTTTTCTTATCAATGAATAGATGTCTTTACTTGTATGACTTAGATGTCTCGTATTTCTCGAAAAAGTGATTCGATTGATGGGATTTGGTATGATACTTATGAGATCGATGATATCGATGAAGTTTATTTTCAAATCTGTCTTCTTAGAACGTATTGATTTGACCCCATAAGCGGGATCACCACCCCATAGCATGTTGCCGCCAGAACCCCGTATTTCTTCTAGACAATCTCCTAATTGTTCCAGAGCAACTAGAAAAAGATTCTTTAACCAGAAAGAATTCTGTTCAGATGTAGGATACCTATCCAGAAGTTTTCGCAACTCAATCATGTATGATGGAATCATCAAAGATTTGATCTTTTCGAACTCTGTCTGTAACTCACTATAGGCTCGGGAAACAAAGAGAAGATGTGTACGAACGATATATCCAGCAACAAGAAGAAGGAAAAGGATTGAATAGAGGACCTCACGAACATTTGGCGATCTCAGATGTGTCGATATCAACGATGACTCATTATTTCGATGAATCATTTCTTCGGACAGAAGAAGATTATGTAAAGACTTACTCGAAATCTCACTTATCAGATTCCTTTGTGGAAGACACAATTTTTTCTGAAGAATTCGCCATGATATATCTAATCCATACATAATATCATGAAAAATGGATACAAATTTTTGACTGCTACTTAGTATCCGCAATAGGTCTGAAAAAATATCTAAAAATATCAAATTTAGATATTTGTACCCTGTCGAAGTAAAGAACCATGGCATATATGTTTGGAATAGATTCCATTTTGAGAGAGTTGAAAAAGCACTATCTCGTTGAAAGGTTCTATCCATCTGCCCTTTGTCAACGCATTTTTTTAGGCAAAGACTCCGTTTTTTCCTCTGTAAATATTTCTCAGAACATGGAGTGTGAATCAAACCCACGTTTGAATTGAAATTGAGATACTGATGCAAGCTCTTCTCTTCTGAATCGGATAGATTCATATCTGAAAGAGTTTGACAATACGTTCTTTCCAAATTTACTCTTTGTCCCTCTATTAGAGGTGTTCCAGAAATGTCTGCAATCGAGTAAATAGCTCTACGAACTAATGGATCGGATCGAATTGGAAAATGGAAAGATTTGTACAAGTTATACCTTTCGTCACCACTTTGTGGAAAATCGTTAGATATGAATATGTTAGATACCTGTGACTCGATTGACGAAGGTGAAATAGTATCTCTCTCCAAAAAAGCATGTTTTTTTTTACCACCACACGAAGAAAATATTTTGTTGTGAATGAACAAGATAGTGAGGAATTGTCCATACGTAAAATCAGAATTATTGAGACGGGCCTTTTCCACATAAAAAGGGAATCTTTTGTTACAATAGAAGCAGAAGTGATGTGGATTATTCAAGAATCGAAGTCGATTTGCTTTAGAAAAAGAAGATATCAATGAACTTCTCTGAAATGGTTTCACGGGATTCAGCCAATTGTCTTGATCGTGGGATACGATTGAGAAATAGGAATCCGTGTTATCAAAAGATTTCCTGCGATTCTTTCTAGTATGGAATGAGTCAATCATCCACTTTGGTATCTTATTGAACAAAAATGGTGATATTGTTCCTCCATTGATCAAGAATTTCGATTTTTGAGAAGTATTATGATCATCCAATAAAAAGGGTTTCAATTTTTTAAAATGAACGATTTGAAGACCTATTGATTCTAACAACTGATTGCAGGGTTGATCGTTCGGACCTTTCAATTCATAGATGTGGATCTCAGACCTATGAATGGGGATATTCTCGAAACTCACAAAGAAAAAAGGAAGTGAGTTAGACAAAAAGAGAAGTAACTTGGACAAAAAACGAAGTAACTTGGACAAAAAGAAACGAAGTGACTTAGACAAATCTTTTTTATCAATAACCTCAGACCAATCAATCGAATATTGATTAATACATAATCGATCGAACACTACTTGAAAACGGCTCTTCCGCTCAGAAACGAAATGTTTCAAATGCTCCTGGAAATTCTTGCTCCCATTGGACCATTTGTATCTATATGCATTAGGATCCCGATTTATGGATCTCTCGGTTCGAGAAAGAAAAATAAGAGGATCGAACCATTTCTTCTGACTCTTTTTCAAATTCGATAAATGTTGGTTGATCGTATCTTTCATTATAGTTCTATGATTCAGAGTATCATTTCCTATTAGATCCCTTTGAATTCCATATTCGAAGTTGCGATCAGGTCTCTTCATTAAAAAGAATCGATTCAATACATTTCTTATGTACCCATAGGGACTATATTGGAATTGGATTTGAATCAGATTTCGGATCAATCTATATTGATTGACTGCCTCCATTATGTTGTTGCTAGCAAATACCACTCTTTTTGGTTTTGGATCTTCAAAAAAATTCCCGCAGGAGATCCGGACCCAATTTTTTCTGATCCTTCGATAAAAAGATTCATTTTCTTCATAAAAAATAGGAGGTAGAACCAATAAAGATTTCTTTTTCAATTCATCCCTGGAGTTGAAAACCTCCTTCAAGAATTGTCTTTGATCCAATCCGTAGGAATCAATAGAAAAGGCAAATCCCTTATGATACACCAGATCCGGCTCGGTTATTGATAGAGTGAATAGATCTGCCATTTCTTGAAATCTCTCTTCTGACTCAAAATCGTGGCGTAACGTGTATCCCCCCCTCTTCCGTTCATGGAATAGATGAAATAAATAAAAAAATGGATTTTTGTTCAAGAATGAAATCTTATTGGAACTGTCCATATCCAGTTCATCCTTCGGAACCGTATCACATCCCAGATCTGATGAAATAGGATGAATTGAGACGGTATTTTGTAAATACGTAATTATCTTGAATATATTAACTATTTCTTTATTTTCCGATCGCCTGGAAGGGACAAAAGAAACATCTTGTTCTTTCTTCAACAATTTCTGATCCCTAGTGGACCTCTCAGTAGGATTCGAACCCAGATGAAGTTCTGACCATCTGTCAGAGAAAAAAGAACGAATGGCTCTTGTAGAATTCCAAAAAAATTCTTCGCTTTCTTCCGGAAGCAGATGATTATTCATTCGCTTTTCACGTTCCGTGAATAGCCGGGGCATTGAGGAATATCCAGAAAGGTATTTAGGGAATCGGTCTGATTCTATCTCTCTTCCTTCCGTTTGAATAAAGGAAGGATCCCAAAGAATCGATCTTTCTTTTAGTTGTTGAATCTCTCTTTGATTGATCAATGTGTGATAGTGATATTCCGAATCCTCATTACTAATGGAATCGAAAGGATCTATGAATTGATCAGAAGATCCGTTCAATTGGCTAGAATCCGTTACTTGAACGAAACTAGATCTTGTAGAATCATATTGAATATTTGACGATACATTTCGTACCTTGCTAAAAAATCTATCCTTGTTTACCAACCACACATTGTCTAACCAAATCCAATTCTCTCTCGATATTTTCCTCAAAAAATCCGATTCGTGCGGATTCTTCCCCCAACTAACGAAGAGATCTTGGTGGAATTGCCACATATGAAATTGAGCACAATTTTGCAAAGAAATAGCCCGCTTGTTTCTCGAGAAGAGATGGGAAACATGCTCAATATCATTTGATTGAATAGTTGACCCAGCTCCTTGTTGTTTGAAGAAACCCTCCACTTCAATTGGTATTTTTTCACGAAAAGCAAACATGAGATAACAAATCCAGTCTTTCACTAAGATTTCGAATAGCTGTCCCGAATTCAAGTTGATTATGTTTCGCCTCTTATTCGGAGAAAGACGATCAAACAATTCCCAATCATGGCCCTTGCGGATCGGATCATCCATATAATATACAAAAAGAAACTCCAGATATTTGATATCTTTCTCTTTAAATGAGATATCAATTCCAGCGACGGTTTCATTAGATATCTTACAACAAAAATCCCTCTTTTTTCCGATCCAGTTCCTCCACCACCGCGAACTCCAGTTAGATTCAGGCATGATACACTTTTTAGTTATTGGGAGAACCCGAGTACTCTCTTTCGGATCCCGGAAACAGCTCTCAGAGATCTTTTTTCCTTTTGTAAAATACAGGAGCGAAACAATCAACCTATTGATATTGGAAGACCCAAAAGATTCTTCCGATGTATCATTTCTGGGTCCAATGGAATTCATAGGTATAGGAAGAAGCCCTTTCAAATAGAGATTTTTGCTTTCGACCATATTTCGATTGTTAATACGATATAGAAGGGCCGCTACTACAAATAGTACTACACCCTTGATCGTGAAATATCGATTGCTTGTTGAACCCTGTGAATTGCGCAAAAGTAGGATACTAAAAATTCGAGGGTCCAAGAGTTTTCTAAAACGTTCTTGGTGGAAAAAAATATGAATGAAAGATCCCACTGAATTGATTTGGGTCCATGAATCTAAGAAATAGTGAGAATTCTTGATCTCTCTCACTATTTCTCTCAATTCGAAAATCCAGGATTTGAATTGATGTCCTTTCATTGATTCCTCCTAAATTGCATTGATTTATCCTAAAGATTTCATTTCAATTGGAATTTGGTTATTCACCATGTACGAGGATCCCCACTAAGCATCCATGGCTGAATGGTTAAAGCGCCCAACTCATAATTGGCGAATTCGTAGGTTCAATTCCTACTGGATGCACGCCAATGGGACCCTCCAATAAGTCTATTGGAATTGGCTCTGTATCAATGGAATCTTCTCATCATCTATACATAACGAATTGGTGTGGTATATTCATATCATAACATAACATATGAACAGTAAGAACTAGCATTCTTATTGAGACTAGAACTCATAGGGAAGAAAATCGATTTATGGATGGAATCAAATATGCAGTATTTACAGACAAAAGTATTCGGTTATTGGGGAAAAATCAATATACTTTTAATGTCGAATCAGGATCAACTAGGACAGAAATAAAGCATTGGGTCGAACTCTTCTTTGGTGTCAAGGTAATAGCTATGAATAGTCATCGACTCCCCGGAAAGGTTAAAAGAATGGGACCTATTCTGGGACATACAATGCATTACAGACGTATGATCATTACGCTTCAACCGGGTTATTCTATTCCACCTCTTAGAAAGAAAAGAACTTAAATCAAAATACTTAATAGCATGGCGATACATTTATACAAAACTTCTACCCCGAGCACACGCAATGGAGCCGTAGACAGTCAAGTGAAATCCAATCCACGAAATAATTTGATCTATGGGCAGCATCATTGTGGTAAAGGTCGTAATGCCAGAGGAATCATTACCGTAAGGCATAGAGGGGGAGGTCATAAGCGTCTATACCGTAAAATAGATTTTCGACGAAATACAAAAGACATATATGGTAGAATCGTAACCATAGAATACGACCCTAATCGAAATGCATACATTTGTCTCATACACTATGGGGATGGTGAGAAGAGATATATTTTACATCCCAGAGGGGCTATAATTGGAGATACCATTGTTTCTGGTACAGAAGTTCCTATAAAAATGGGAAATGCCCTACCTTTGAGTGCGGTTTGAACTATTTGATTTACGTAATTGGAAGTAACCAATTAGGTTTACGACAAAACCTAGAAATCGATCACTGATCCAATTTGAGTACCTCTGCAGGATAGACCTCAACAGAAAACTGAAGAGTAACGGCAGCAAGTGATTGAGTTCAGTAGTTCCTCATATAAAATTATTGACTCTAGAGATATAGTAATATGGAGAAGACAAAATTGTTTCAAGCACCGACAGAACCATAAGCGCCCCTTGTTTCAAAGAGAGGAGGACGGGTTATTCACATTTCATTTGATGGTCAGAGGCGAATTGAAAGCTAAGCAGTGGTAATTCTAAAGATTCCCCCGGGGAAAAATAGAGATGTCTCCTACGTTACCCATAATATGTGGAAGTATCGACGTAATTTCATAGAGTCATTCGGTCTGAATGCTACATGAAGAACATAAGCCAGATGACGGAACGGGAAGACCTAGGATGTAGAAGATCATAACATAAGTTATTCGGCAGATTTTGATTCCTATATATCCACTCGTGTGGTACTTCTACCATATATAGAAGAATTCTACGATATATATAAGATAAGATCCATCCGTATAGATATCATCATCTACATTCAGAAAGCCGTATGCTTTGGAAGAAGCTTGTACAGTTTGGGAAGGGGTTTTGATTGATCAAAAAGAAGAATCTACTTCAACCGATATGCCCTTAGGCACGGCCATACATAATATAGAAATCACACTTGGAAAGGGTGGACAATTAGCTAGAGCAGCGGGTGCTGTAGCGAAACTGATTGCAAAAGAGGGGAAATCGGCCACATTAAAATTACCTTCTGGAGAGGTCCGTTTGATATCCAAAAACTGCTCAGCAACAGTCGGACAAGTGGGAAATGTTGGGGTAAACCAGAAAAGTTTGGGTAGAGCCGGATCGAAATGTTGGCTAGGTAAACGTCCTGTAGTAAGAGGAGTAGTTATGAACCCTGTCGACCACCCCCATGGAGGTGGTGAAGGGAGGGCTCCAATTGGTAGAAAAAAACCCGTAACCCCCTGGGGTTATCCTGCGCTTGGAAGAAGAACTAGAAAAAGGAAAAAATATAGTGAGACTTTGATTCTTCGTCGCCGTAGTAAATAGGAGAGAAAATCGAATTTCTTTCTTCGTCTTAAAAAAAATAGGAGTTAATTAACTGTGACACGTTCACTAAAAAAAAATCCTTTTGTAGCAAAGCATTTATTAAGAAAAATAGAGAAGCTTAATACAAAGGCGGAAAAAGAAATCATAATAACTTGGTCCCGGGCATCACGGGCGAACGACGGGAATTGAACCCGCGATGGTGAATTCACAATCCACTGCCTTAATCCACTTGGCTACATCCGCCCCTACTCTACTATACATCATATCTTGTTTGTATTGTCTAAAATAAACACGCAGCAATATTTTTTTTATCAAAAAAAAATTATAAATTATAATAGTATTTTTTTCTATTTTATTTTTATATATAATAGAAAAAATTATATAAAAAAAATGATTTGTTCCGTTTTATAGAAAAAAACGAGCGATATAAGCCTTCAAAAGAAGGCTTATATCGCTCGTTTTTAATATTACTAAACTAGGTCTAGACTAACACTAAAGAATTATCCATTTATAGATGGAGCCTCAACAGCAGCTAGGTCTAGAGGGAAGTTGTGAGCATTACGTTCATGCATAACTTCCATACCAAGGTTAGCACGGTTAATAATATCAGCCCAAGTATTAATAACACGTCCTTGACTATCAACTACTGATTGGTTGAAATTGAAACCATTTAGGTTGAAAGCCATAGTACTAATACCTAAAGCAGTAAACCAAATACCTACTACCGGCCAAGCCGCTAAGAAGAAATGTAAAGAACGAGAATTGTTGAAACTAGCATATTGGAAGATCAATCGGCCAAAATAACCGTGAGCAGCTACAATGTTGTAAGTTTCTTCTTCTTGACCGAATCTGTAACCTTCATTAGCAGATTCATTTTCTGTGGTTTCCCTGATCAAACTAGAAGTTACCAAAGAACCATGCATAGCACTAAATAGGGAGCCGCCGAATACACCAGCTACACCTAACATGTGAAATGGGTGCATAAGAATGTTGTGCTCAGCCTGGAATACAATCATAAAGTTGAAAGTACCAGAGATTCCTAGAGGCATACCATCAGAAAAACTTCCTTGACCAATTGGGTAGATCAAGAAAACAGCAGTAGCAGCTGCAACAGGAGCTGAATATGCAACAGCAATCCAAGGACGCATACCCAGACGGAAACTAAGTTCCCACTCACGACCCATATAACAAGCTACACCAAGTAAAAAGTGTAGAACAATTAGTTCATAAGGACCACCGTTGTATAGCCATTCATCAACGGATGCAGCTTCCCAGATCGGGTAAAAATGCAAACCAATAGCTGCAGAAGTAGGAATAATGGCACCTGAAATAATATTGTTTCCGTAAAGAAGAGATCCAGAAACAGGTTCACGAATACCATCAATATCTACTGGAGGAGCAGCAATGAATGCGATAATAAAAACGGAAGTTGCGGTCAATAAGGTAGGGATCATCAAAACACCAAACCATCCAATGTAAAGACGGTTTTCAGTACTAGTTATCCAGTTACAGAAGCGACCCCATAGGCTTTCGCTTTCGCGTCTCTCTAAAATTGCAGTCATGGTAAAATCCTTGGTTTATTTAATCATCAGGGACTCCCAAGCACACAAATTCTCTAAAACTATAAGTAGATAATTGAGAGCTTGTTATTGAACAGTATAACATGACTTATATAGCCATGTCAACCAATGTAAAACGGCTAAGATCCTTTTAGTTTAGATTCATAATAATTTTTTTATCGAGGAGAGAAATTATAAACGAATCTATATACATAGAAATATAATTTCTCTATGAATATTATTTCAAAATCATATGAATATGATCCATAGTGGGTTGCCCGGGACTCGAACCCGGAACTAGTCGGATGGAGTAGATAATTTCCTTGTTAAAATGAAAAAAAAGTAAAAAACCCCTCCCCAAACCGTGCTTGCATTTTTCATTGCACACAGCTTTCTCTATGTATACATAGAAAACTCAGTTTCTTTGTTTCCTTATAAATAGGACTGCGAATTCAATACTCAGTAAATTTCATCTTAGTCTTACTGTATGAACATTTAATAATAGAAATAAATGACTTTTGATAATACAAAATAATTTATTTTTTTGTTATCTCCGCATTCCGTTTACGTTCTGATAAATTTTGATTTAATTTATGGAGCCTCAGAACCCCATTATTCATGATTGACTAAATCATTAAGATAAAGAATATCCAAATACCAAACCCGCACTCGATATAATCTTTTAGAAGCATAATCACTTCTTGGGAAGATTAAAGAAAGAACTTGGTCTTCCCCCGTAAGGAATTCTTCTAATAAACCCGAGCCCAACCTTTTTAAAAAAGCGCGTACAGTACTTTTGTGTTTACGAGCCAAAGTTTTAACACAACAAAGACGAAGTATATATTTTATTCGATACAAATTCTTTTTGTTTGAAGATCCGCTGTAATAATGCGAAATATTTCTGCATATACGCACAAATCGGTTGAGAATATCAGAATCTGATGAATCCGTCCAGGTCGCTTTACTAATCGGATGCCCTAATACATTACAAAATTTATCTTTAGCCAACGACCCAATAATAGAAGAAATTGGAATGTTGCTATCCAATTTTATTCGAACATTATCTATTAGAAATGAGTTTTCTAGCATTTGACTACGTACCACTAAAGGGTTTAGTCGCAAACTTGATAGATAACCCAGAAATTCTAAATTATCTTTAGATAATTGATTTATATTAACCTTTTGCGATTGAAACCATACGGAAAAATAACATTGCCATAAATTAACAAAATAATATTTCCATTTATTCATCAGAAGTGGCGTATCCTTTGTTGCCAGAATGTATTTTCCATGATATCTAACATAATGTAGGAAAGGATCCTTGAGCAACCCTAAGAGCGCCGAAAAATTATTAACAAAGACTTTTAAAAAATGTTGTATTTTTCCATAGAATAAAATTCGCTCAAAAAAGACGTCATAAGATGTCGATCGTAAATGAGAAGACTGCTTGCGTAGAAAAAAAAAGATGGATTCGTATTCACATACATGAGAATTATATAAGAACAATAAAAATCTTGGATTCAAAATTGATTTTTTTTTACTATCAAAATTCTTCCAATTGCAATACTCGTATAGACAGAACCGAAAAAAATGCAAAGAAGAGGCATCTTTTACCCGGTAACGTAGGGTTTGAACCAAGATTTCTAGATGGATGGGGTAAGGTATTAGTACATCTAATACATAATTAAAATGTGAGAGTTTGTCTTCTAAAAAGGGAAATATTGAATGAAGTGATTGTAAATTGTAAGATTTTTTTACATTTTTTCCTTCGATAGAGGATCCCAACCTTAGGGAAAATGGAATTTCTACAATCACTGCAAATAAAACAGATATCATTTGATAATAGAAATTATTGGTATGCCCCAGATTTTTGTTCAAATCCTTAGTGGGAATAATCAAACGATTCTGTTCGTACATTCGCAAAATTAAGCGTTTCACAATTAGTGAACTATATTTTTTGTCATAATCCGCATTTTCCAAGAAAATAGGGCGGTTTCTATTTAATCTATTTAAACCGTGATCATAAGCAAGTACATAAATATAGTCCCGAAAAAAAAGTGGATATAGAAAACTCTGTTGACGAGCCCCATCGAACTCTAAATATCCTTGAAATTTCTCCATTTGGATTAAAATTCGATTTGAACTAAAAGTAAAGTCTTTATTTTCTTGAGTTCTGAAATGACACATAGTGCGATACAGTCAAAATAAGGTATTAGATTACGAAAGCACTAAATACCTCATAAACAGGTAGACTGCTAACTGGATTCTCTATCTTTAAGAGGTTTCTGTTCGTTATATTATAAAATAACAAAACAAGATGATTAGAAATCCTTTATTTTTTTAACCTAATCGCTCTTTTGATTTTGGAAATATATATATATATATTTTTTTATCAATATACTGCTTCTTTTACACATCCATCTACAACCTAACCCAAACGGACTAGGTAAAAAATAATTAGGACTCACGAAAAAATTGATAATAACACGCAAGAAAAAAATTCCTTCCCATACCCGTATTAGGTACTAATCTATTTTTAACATTTAATTAGATCGGGTAATTTTTCAAATTACGAATGGAAGCTCGTTTCTTTTTTTTTTCTTAGAATAAGGAAAACTGGTTTTTTATCCATCCATTTATATTTATTCACTCGACCCAAATTGGAATTCTTCTTTTTTTTTTTATTTTCTAATCGGTACAATCTTGTCGAAAATAAAAAAAAATGTTATCTGAATTCTCCCTTGATACGACATGCTATTTTTTCCGTTCATTCCTTTCAGGATCAGTCGTGGTCTTACAAACTCTACCGCGGATCTGGACGAATCCTTTTCTTCATACAAATGTGTAAAAGATGCTAGTCGCACTTAAAAGCCGAGTACTCTACCGTTGAGTTAGCAACCCCCCCCACAAAAAAAAGCAAGTACTGCAAATATGTAGATACAACCAGAATAAAGAAAAAAAGAAAAATCCAGTCATGTGTGCGTCAGGGAGAAATAGATCTATTTCTCTATGAGAGAATTATATTTGGTCCATACACTGTTGTCAATATGATTGTAAATTTTTAATATAGCGAAAAGAATAGAAAAAATAAAAAAGTTTAACCCCCTGGTTTGTGAGTTCATACAAGGAATGAAAACTAAGCCGAATAGGATAATCTCAAATCTTTCTATTTCTATATATATTATGATTCAGAATTAATATATTATTTATTATATTAATATATTATTTATTATACAGTATGATTTTATATATTTTCTATACAATAATTCTATACAATAAAATTTTGTATTTATACAAAATTTAGAATTTCTATAAATCCAAAAATTTTTTAATAAATTTGTTTTTTATTATAAAACATGGTAGTTTTTAGCAGGATATTTGTTAGTTTTCATACCTTTAGGAAGAATACTAATAATAAATGGAAATTCTAATAAATCAAAATAAATATGATGGAAACGAAAGAGGAGGAAAGAAAAGAGTAGATCAAATTTTATACCAAGCTATATATGAGTCTTTAACATCCTCTTTTTTATAGTTCATTAATTCAATTTCGTTTTATTAAGACTTAATTCCGTAAAAATCCCTGCCTTCTTTGAAATATCATGAACTGTTCTTGTTGGTTGAGCGCCCTTTTTAAGGAAATCGAGAATAGCAGGAAGATTTAAATAAGTTTGATTAGTTATCGGATCATAAAACCCCACCTTCCGAAGATCTCTTCCTTCTCTTCGGGATCGAACATCAATTGCAACGATTCGATAAATGGCTCATTGGGATAGATGTATATGAATAATACCCCCCCCCGAGAAACGTATACGAGGCTTTGGCCTCATACGGCTCGAGAAAAAAATGCAATGAGTATAAGTTAACTCTCTTTATAAAATTCAAACATTAAATTAATTAGCCAGTATTGAAACCCTAACTATTTTTTTTCATAAAAAGCGTTCGTAACATTCGTACTCTCGTAACTCAAGTTAAATAACTCTCAAATATCTCACCAGAGACTCCTTAAGTACTCTTTTTATTGAGTAGTCTCTAACCTTTTTTTGTTTGTCTCATTTTTTCGAATCAATTTTGATTCTTCATTCTGATCTAGTTGTTCAAACAATTGAAAAAGGGATTTCCTTGTTTCAGGATTCTTTATCCTTACTTTGAATCTTTGGGTTTAGACATTACTTCGGTGATCTTGATCGTTTTATTAAAAAAGGGCAGCAACAAGCCCCTTATTTTGTTTATGATTTCTTTTCTTTCTATCAAAGAATCATACAAACGCTTGATTCACGCATGATAGACTTTTAATTCAAAGAATTTTACACTTTTACGAAAATTTCCTTTTCCATTGTAAAATTACTTGAAAGGGCTTTTTTTCAATATAAAAATAAAAAGACTTACGAAGTTGTTCCAACTTATTGATTCGCACTAACCCTAGATCCTTACTCCTGCGAAAGGAATAAAAACTTTCTATTCTCCTCGAGCTCCATCCTGTACTCTTTTTTATATTCAAAAAGGTGTAGGACTCTCGTAAAATAGAACACAAAATGTCGAGCCAAGAGCACCTATATTCCTAATATAAAAGGTGGCGGATCAAAACATCCACAGCAGATCATGTCCTTCAATTCAAGTCGCACGTTGCTTTCTACCACATCGTTTTAAACGAAGTTTTACCATAACATTCCTTTAGTTTGTGTAATTGATTCAATTATGGAATCATGAATAGTCATAGTTCAGTCAGTATATCGTAATCTATACTTTTTCTTTCTCTATGAATGGAATAGTGAATCTACGCGTAAAAGGTTCAGTCAGAATTCAAATGAATTCCACATTAAATTCTATATATGTAAAAATATGTAAAATCGAAATTTGAATAGAAATCTATATTTATATATATAAATATATATATAAATATATTTTTTTTTATTAAAACTCGTAGAATCTACGGTTCTACCTTACTTACCTACATCACACACAACTAAAAAAAGCAAATAGATTTTTTGTAATTTCGGGTGAAAAAGAAGTTTATTCTTCTTTTCATTTCAATATTTTATTCTTAAAAAATATTGTTTTTTTAAACAGAAAGAAAAAGATGGTGTACAAAGGCAATAGAAGATTTATTTCGTAATGACTGGACTCTGGGACGGAAGGATTCGAACCTCCGAATAGCGGGACCAAAACCCGTTGCCTTACCGCTTGGCTACGCCCCATTTTTATTTTTATTCAAGACTACTAAAAGAGTAATATTGCTATTGGTTGTTCGTCAATTCAATTTCAGCCCAAATGAAATATAGATTACATTGGTGCTATAGTTTTGACACGTGTAGATAGCAAATCAAACTTACTTTATTGATCATTACATAGAATTCAATTAAGATATTGTATGAAAATATTATTTCTTTCATTCTCATAAGAGAATGAAAGGATTTTTGATTGAGTAAGTTCAACAAAGTCTTTTTAGACTATCTTTCTTTATTTATTTATTTTTTTCCTTATATAAAAAATATATTAATAACTCAATCAAAATTAAATTATCCACAAGAACACCAATTTTTGTTATGCTTAATATATTTAATTTGATCTGTATTTGTTTTAATTCGGCCCTTTTTTCAAGCACTTTTTTAGTCGCCAAATTGCCGGAGGCCTACGCCTTTTTGAATCCAATCGTAGATGTTATGCCCGTAATACCTCTTTTCTTTCTTCTCTTAGCCTTTGTTTGGCAAGCAGCTGTAAGTTTTCGATGAAATTATTAATACTGTCTTAGAAAAATTCACGATTTTGATTCTTCCAACAATTCAAATCAAAAGATCAAAAAATCTTGACGTAGGAAGGAACTCTCAATTCAAACATTGAATTTTTTTGGTAGCCATACTAAATCTGGATCATTTGATTTCCTCAGTTTTATCCTCTTTTCTCTAAATGAAAGAACTTAATTAGATTCGAGTTCACTCACAAAAAAAGTATCTAGATATTTAGTATAAAAATAGAGAATCTATTCTCTTTTTTTTTTTTTTTAAAAAAAGTAAGATCTTGGAGATTGTGTAATGCTTACTCTCAAACTTTTTGTATACACTGTAGTTATATTCTTTGTTTCTCTCTTCATATTTGGATTCCTATCTAATGATCCAGGACGTAATCCGGGACGTGAAGAATAAAAAAGAAAGGTTTTTTATTACTTTATTTAATATTTAAATAGTGGAAATATTTAAATAGTGGAAATGTGCGAATTTTATTAGGATTTTATCTATTTCACATCATCAAAAAGGGGAAGGGAAAGAGAGGGATTCGAACCCTCGGTACGATTAACTCGTACAATGGATTAGCAATCCAACGCTTTAGTCCACTCAGCCATCTCTCCTAATCGAAAAGGAATACTTTTTAGGTTCCATTAGACAAAAAAACGGCTTAAAAAAAAACTTTCTCCACTTTATTCTTAAAAAAGTTTTTTTTTTTAGATTATTCTTTATCTTTAATAATACTTTAATTATATATATATTTTCATTTTCTATATTTTATTATATATATTATTTTATTATATATAAAAAATATAATATTATTTTTTCTTTTTTATTATATAAATAATATTTATATAATATTTATATTTATATATAATTAATATATATATATATATTACTATTATATAACCTTTTTTATAGAACTTTCTCAGTAATTCTATTTACATAAAAACTGTAAATAAAGATTCAATAAAGAAAAGGCTCGAAAGAGAAATAAATAAAATCACAAAAATAGAAATAGAGAATCCTTTTTGATTTTGTCTCGTCCAAACACAAATAAAAGATCTTTTTTATTTTAATAGCCTGGCCTGGTCAGTCCCCAGCCGGGCCTTTTTTTGTTAAAGTTAAAAAGACCCATCCGATGGGTTTTTAGACAAAAAAGATCTGAAATAAAAAAAAGGAATCCTGCTTTGCCTAATTTTATTAAGTCTACGCTAGAATTTTCTCATTTTTTTTTCAGATTTTTTTTCTCCCGATTACTTTGTTCGACAAAAAGTAAATTTATATACAATAATTGGATTGTAGCGGGTATAGTTTAGTGGTAAAAGTGTGATTCGTTCCTTTAACCCCTTTAATAGTTAAAGGGTCTCTCGGTTTGATTAATCTTCCGATCAAAAACTTTATTTCTGAAAAGGATTTAGTCCTTTACCTTTCAATGAAAAATTCAAGGAAGATTATAGATTCTCGTAATTTGTATCCAAAGACTCTAATTAATTGTCAATTTGGATTATGAAATTTCGAAACATAATTTTTGAATTGGATGACTATTTACAATTCAATAAGTATAACAAGAGGATCCATGGATAAAGCCAGAAAAGTTTCTTTCTAATCGTAACTAAATCTTCAGTTCTATTTTTTGTTTGGTATAGAAAAAATTGAAGCAAAATAGCTATTAAACGAGAACTTTGGTTTACTAAAGACATCGACATATTATATTGTTTTAGCTCGGTGGAAACAAAATACTTTTCCTAAGGATTCCGTTAAATAGAAATAAAGAACGAAGTAACTAGAAAGATTTTTTGAGTTCGCCTTTTCTATCTTCTAGAAGGATCATCTATAAAGCAAAATTTTTCTGGGAAAGCCTCCAAACGGGAAAAAAGCTAACATAGATGTTATGAGTCAAATTTTGATTTAGTTCCCATCTTATTTTATTTGGGAATTTCGCCATCCATCATAAAGGAGCCGAATGAAACCAAAGTTTCATGTTCGGTTTTGAATTAGAGACGTTAAAAATATAAAACTGATCGATCGACGTCGACTAAAACCCTTAGCCTTCCAAGCTAACGATGCGGGTTCGATTCCCGCTACCCGCTCTAAATTCTAAATTGTCCCCTTTTTATTAGAGACAATTTTCTCTATTAGAATTGTCTAATAGCAATTGTGTAGTGAATTCACTACACAATTGCTAAAAAGATTTCGCACATTTAACAAATGGGAAGTTAAAAAAAGCGAAAAGCGTCCATTGTCTAATGGATAGGACATAGGTCTTCTAAACCTTTGGTATAGGTTCAAATCCTATTGGACGCAATATCAATATAGATATAAATATATTGATATTTATCTATTATTTCCATTTCTATATATTATAAGAATATTTTTATTCTGTTTAGAAAATTTATAAAAAATAAATATAATAAGAAATAAATTCTGAATGCTTTAAGATTTTATATTAAACATATACAGATATTAGTTCTATACTTATATATATTATATATAGACTTAACTTAAATATATTTCTATTTTTTATAATTTCTCCTGAAGTAGAAAACGTTCCAGTTGCTCTTTAATACCTTCTTTCAAAAAGCTTTCTGCTTCAGCGGTTAATGTCTTGGTAGAGGCTATTATTTCTTGGAACTCAGGTTTATTTGTTTTTAAATAAGTGCGTAGCTGAACGAGAAATTTTCTTACTTGTCCAATTTCTAATCCATCCAGATAACCATTTGTTCCGGTATAAATGGTCATTATCTGTTCTTCCACTGTGAGAGGGGCTGATTGGGATTGTTTCAGTAACTCACGCAATCGTTGACCTCTTGCCAATTGATTCTGAGTAGCTTTATCGAGATCAGAAGAAAATTGGGAAAAGGCTTCTAATTCCGCGAATTGAGCCAATTCCAATTTTAATTTTCCAGCTACCTGTTTCATAGCTTTAATTTGAGCGGCAGATCCTACTCTCGAGACAGAAATCCCTACATTAATAGCAGGTCTAATTCCAGCATTAAAAAGATCAGCGGATAAGAATATTTGTCCATCTGTAATGGAAATTACATTAGTAGGAATATAAGCTGAAACATCTCCTGACTGGGTCTCGACGATTGGTAAGGCAGTCATACTTCCTTCACCTAATTCAGAGCTTAATTTAGCGGCTCTTTCTAAAAGACGTGAATGTAAATAAAAAACATCTCCTGGATAAGCTTCACGCCCGGGCGGTCTTCGTAATAGAAGAGACATTTGTCGATAAGCTTGTGCTTGTTTGGAAAGATCATCATAAATGATTAAAGTGTGTTGTTCACGGTACATAAAATATTCAGCCAAGGCGGCTCCTGTATAAGGCGCGAGGTATTGTAACGTAGCTGGGGAATCAGCCGTTTCAGCTACCACAATAGTGTAGTCCATTGCCCCTCGTTCCTGTAAACTAGTCACTACCTGAGCCACGGAAGAAGCTTTTTGACCAATAGCCACATAAACACATATTACATTTTGACCTTGTTGATTGAGAATTGTATCTGTGGCTACTGCTGTTTTACCGGTCTGTCTGTCACCAATAATTAATTCCCGCTGGCCGCGTCCTATAGGGATCATGGAATCAATAGCAATAAGTCCTGTTTGAAGAGGCTCATATACAGAACGTCTCGAAATAATACCTGGGGCAGGAGATTCAATTAACCGAGATTCAGAAGCTGAAATCTTACCTCGACCATCAATAGGGTTAGCCAAGGCGTTTATAACACGCCCCAAATAAGCCTCACTCACGGGTATCTGAGCAATTTTTCCCGTAGCTTTGACTGAACTTCCTTCTTGGATCATCAAACCGTCACCCATTAATACAACACCAACATTATTTGATTCTAAATTAAGGGCAATACCTATAGTACCCTCCTCAAATTCTACTAATTCACCTGCCATTACTTCATCAAGACCATAAATCCGAGCGATGCCGTCGCCCACTTGAAGTACGGTACCGGTATTTACAATCGTCACTTCTCTATTATATTGCTCAATACGTTCACGGATAATATTACTAATTTCATCGGCTTTAATGGTTACCATGAGTATTGTCCTAATTCTTTTTTGGAAGAAAAAAAAAAATAATGCCTATCATAATCGTAAGGAAAGAGCTAATCAGTAATTTCTTTCATCGTACCAAACATACCAATATTTGCATTAATAGTACGTAAATGTAACTCATTACTCAAACAACTATTTAGGGTTCCTATAGCTCCCTGTAAAGCTTGTTGGAAAACCCGTTCACGGACTTGATTAATTGTTCTTTGTTGCTCAAAAAGAATGGTTTCGTTTTTGTAATTTTCTAATTGTTTCAAAGTCCTAGAAGTTGAATTAATCAAATTTACTTTTTCTCGTTCGATTTCAGAGTATCCATTTACGCGAAACTGATCCGCCTCCATTTCTACTTTACGCAGGCGAGCTCGGGCGTTTTCTAATTGTTGAATAGCTCCTTCACGTAGTTCTTCTGAATTTCGAATAGTATTTAATATCCTCTGCTTTCGGTTATCTAATAAATCATTTAATGAAAGTAGATTATTCAGTAAAAAAAAAAGTTCTATGATCCCTTCCCGAACCAAACATGAATCTTTCGATTCATTTGGCTCTCATGCTCACGTATTCCAATCATTTATCAATTATGTATGAGACTTTCATTCCCATATTTTTCATGTAATGAGCCTATCCTCTCCCAATTTTGTTGTATTCAATTCATATTCAATATATATTTCTATCGAAAAAGATCACCAATCCAAGACAAAACTATTTGGAGGATTCTTCTGACCAATAAAAAATTGATAATTGTCAGCAAAGTTGTTTCTTTTTTTCTTGAAATCCAAAGAATTTTTATTACTTTATACGTAGGTTATCAATTCTGCATTATACAAAAAGACTCAAAAATTTTTATCGACATGAGTGTTTTATATCGAAAAAAGCCGAACTATTCTTTTTGAAAATCTTATTCATTTTTTAATTAGACTACATATGGTAGAAAGAGTACCATGTTGCATCTGAACTTCAAACGGTTTAGTTTTAACCATGTTAATTAATGGTCCCAAATTTTTGGTTGATAGAGAATCAAAGTCAAGTAGACTTACCAAAGAATAACGAAATGCTATGGTTCTAAAATATGATTTTTTATTGAATTTTGTATTCAGAAGTAATTCGCGGGATTAGGCACTCTTTGCTAGTTATAGTGCCACTGGACGAATCCAGCCTATTCTTGAAATGAACAACTCACACACACTCCCTTTCCAAAAAAGATCAATACACCGAAGACTACACTTAGATTTATTGGATTTGTTGCTAAAATATCGGTATTAAACCCGAAACTCCCGGCGGATGGCCAGTGACCCAAGTAAACGAAAGAATCGGTTAAATTTTTCATATAATCTCCTCTTCTAGCTAAACTATAAAAAAAAAGAACTCTGTCCTTTTTTTTTTTTATTCTTTGTTTTTTTGAATAAAAAGAAAATTTCGTTTAATAATTTATAATTTAATTTACCTATTTGGATATTTATAAACAGAATCAAAAACCTATTCTATTTACAAATTTATTTTCCAAAAATTTTTAATTTTCAATAATAATAATGAGACTTAATTAAAATTAAGCTAGAATTTGAGACCAAGTTTTATATCAATTTTAAAAAACCTAAACCTCCTTTTTGCGCAACACTCCTTAAAAAAAAATTTCCATTAAACTAAAAAGAATAAGGGGAAGGAAGAAAGCGAATCGATGTGTTAATTCCCCATCCTCAAATTAGTCCTTCCCAAGGGTTGTTGTCTCAATGAATAATTGTAGGAGTGAAATCTTGATAGAATAAAAAAAACTACGAAAAAAAAAATTCCTAATTTTATTATTTCTAGGATTAAACAAAAGGATTCGCAAATAAAAGCGCTAATGCTACAACTAGGCCATAAATTGTTAAAGCTTCCATAAAAGCCAAACTAAGCAATAAAGTACCTCGTATTTTTCCTTCTGCCTCAGGTTGTCTCGCGATACCTTCGACAGCTTGACCCGCAGCTGTACCTTGACCAACCCCAGGTCCAATAGAAGCAAGCCCAACAGCCAACCCAGCAGCAATAACCGAAGCAGCAGAAACCAGTGGATTCATGATAAGTTCCTCACACCAAAATAAAGAAATAGTTAATGATACAATCATCCAACGACTTAGGACTTAATTATAATTAAGTCATCGCTAAGATTCATCCAGCCAAAATAACCAAAAACTTGATAAGAATTACTTTGATATTAGTTCCTATCCACGGGATTTTGAAAAATGCATAATATATATATATACGACTTTTTTATGCCGTTTCTTTTTTGTGAACCATTCTTTTCTTTTAATTCTTCGTTCTTTTTTTGATCGTTTTTTTCAGCCAATTCACAGATAAAAAGTAAGAACTTATAATCGAATCGTTATCTAAATAGAAATTCACAAAAATAGTGGGGCAGATTATATAGATCTTTAACTTATATATACCTAGTCAATATCAAATATGACATATACAAGTGTTTCTTACATAACGTAAACCAACTATTCGATAATTGGGCTAACCTAAATTTGAAAAAAAAAAATAGTTAATGATGACCCTCCATGGATTCACCTATATAAGCCGCAGCTAAAGTGGCAAAAATGAGAGCTTGAATCCCGCTTGTAAATAATCCAAGGAACATGACAGGTATAGGAACCACTAAAGGTACTAAAGAAACAAGAACAACAACTACTAATTCATCGGCTAATATATTTCCGAAAAGTCGAAAACTCAGTGATAGGGGTTTTGTAAAATCTTCTAAGATGTTAATGGGTAAAAGAATTGGAGTTGGTTGAATGTATTTACTGAAATACCCTAATCCTTTTTTGCTAAGACCCGCATAAAAATATGCTACTGATGTGAGTAAAGCTAAAGCAACCGTCGTATTTATATCATTCGTTGGTGCTGCTAACTCCCCTTGAGGTAACTGGATAATTTTCCACGGTAAAAGGGCTCCTGACCAGTTAGAAACAAAAATAAATAAAAACAGGGTTCCAATAAAGGGAACCCATGGACCGTATTCTTCTCCAATCTGGGTTTGACTCACATCTCGAATGAATTCAAGGACAAATTCAAAGAAGTTTTGGCCGCCAGTTGGAATGGTTTGTGGATTGCGAACCGCTAGAGCTGCGGAACCTAATAAGATAGCAATTACAACCCAAGAAGTAATAAGGACTTGCGCATGGACTTGGAACCCCCCTATTTGCCAATAGAAATGTTGGCCTACTTCTACACCAGATATCTCATATAACCCTTCTTTTATTAGTGTATTGATGGAACATGATAAAACATTCATATTGCCCTCTGACAGAAATAAGAACTTTAAATTATTTTGATTCAAGACCCCCCCTTTTTTTTTACTTATTTACTTGAATTTTTATTTTAGTTTTGGATACCAACTAAACGAATCACACAATATACCCAGTTTTTTATCTCTTTTTCTTTTGTATGATTCAGGAATAGTAACCGATTTTATAAATCGAAATACAGGGAGCCCCTCCCTCAAAAAAAATTGATTTATTTATCTTATTATTAATCAAGAATTTTGTATATAGCTAGAACGACCCTCACAAATTGCGAATACTAATTTGTTAAGAATGAATCGAATTGAAGCTATAGCGTCATCATTTGCTGGAATAGAAATATCCGCGAGATCGGGATTACAATTTGTATCGATTAAAGAAATGGTTGGAATTCCCAAAGTTATACATTCTCGAAGAGCCGTATATTCTTCTTGCTGATCGATGATGATTACAATATCAGGCAATCCCGTCATATATTTAATCCCGCCTAGATATGTTTCCAAGCGAGATAATTGTCTCTTCAACACAGCTGCATCCCTTTTCGGAAGACGGTTGAATCCCTCTGTCTTTTGTTCAGTTCTCAAGTCCCTAAACTTATGAAGTCTTTTTTCTGTAGTAGACCAATTTGTTAACATGCCGCCGAGCCACTTTTTATTAACATAATGACACCGAGCCCTTATTGCAGCCCGCGACACTAAATCAGCTGCTTTATTTTTTGTCCCAACAATTAAGAATTGTTTTCCCCTACTTGCTGCATCAAAAACTAAATCACAAGCTTCTGATAAAAAACGAGCAGTTCTAGTCAGATTTATAATATGAATACCTTTACGCTTTGCAGAAATATAAGGTGCCATTCTAGGATTCCATTTCCTAGTACCATGCCCAAAATGAACTCCTGCTCTCATCATCTCTTCCAAATCGATGTTCCAATATCTTTTTGTCATTTCTTTTCACACTTAAAAGGGGGGTACCCAAAACTAAAATAAAAATTTGTTCCAATGGAACCTTCTCTTGTCCGTTTATGCACGAGCCGAGCCATTATTTTGTATTCATTATTATCTTTATTAGTGTTAACAAATTATTAAAGCAAATGACTACAGCAAACAATAAAACATGAAATTCAAAACAGGAATCTGCTATTAGGAATTATTCAATTCTAGAAAAGGCAGATTTGTAAATAGAAGAGTCACAAAATTCCCTGTGATAAAATAAAATATCTCTCATATCTCCCTCTAATAAAGATAAATTCTTTGTTTTTTTTTCAAAAAGAATATTGGTATGTTGCCGTGAACAATGCACCAATCCTTTGTTGAACCCGGTCCCGGCGGGGATCACACCCCCTAGAACAACATTTTCTTTCAGGCCTTTCAACCAATCGATACGACCCCGAAGAGCAGCTTTTGCTAAAACTCTAGCAGTTTCTTGAAAACTTGCTTCGGATATAAAACTTTGAGTATTCAAAGATGCTCGAGTTATTCCTAATAAAACGGCTCGATAACAGATTGCTTCTTCTAAAGCACGCCCCGTGCGTTCTGCTCGTAACAATCCAATCAATTCTCCAGGTAAAAAAACATTAGACATTCCCTCTTCTGAAACCAAAACTTTTGATGTTATTTGACGTACAATAATTTCGATATGCCTATTATGAATCTGCACCCCCTGGGATCGATAAACCTTTTGAATCTTATTAACCAAAGAAATACGACTTTGCACTATAGTTAGCTCAGCACCAATCAAGAATCCCCAAGGAATTCCAAGAATTCTTGTTATACACCTGTTCCAACCCTTAATCCGCTTTTCTAAGTTCAGTGATATTGAATCAATCGAGCGGACTTCTAACACCTGTTCTACTTTTGGAAGACCTTGGGTTATATCACCGGATCTCGATTTTTCATATATAAATGTAACTAATGTATCCCCTTCGTAAAGAATTTCTCTATAATGCCCGTGAACTTTTGCTCCCGGAGTAGCCAAATAGGGCTTAGCGGATCTTATTACTACAGAATCCCTTTGAACAATTAAAACTTGACCCGATTTTAGGTATGGTTCTTTTTTGGCTATACATAGATTTTCACAAAAAAATTGTCCAAGACTTATTATTGTGGACGTTTCCTCACAATAATAATTATTATAATTTTGATGAAGAAAATACCAATTCAATTTGAATGGATTCAAAACAAGGTTACTGTATGGATCTAGATTAAAAATTCTTCCGTTTTCATCTATTAAATAAGAGTGAATTATTTGAAAAATATATTTGAAGTTATCAAGTTGCAAATATTTAATTACAGAGATCTGATTATAAGTTAGTAAAGGCAAAAATGAATAAAAATTCGAAATTTGAATGGCTGTTCCTAAGGGGCCCGACGAATTTTGAATTGTAATTAGAGGTTTTTTTTTTATTGATTGGTTTATAACATTGTGATATTTTACATGATTAAATGGACCGATTCTAAAACAATTAGAGGATGATAAAATTAACAAAGATTGGGATTCCTTATTTCTGAACATACGAATAGTTCCATGATTTTGTCTAAGCGATTGTTGAAGAATGCCAGCCTTGGGAGAAAGCGAATAAAACGGATTCACGGAATCTGCAGAGATCAATCCCGAATCCGGCGGATTATTTCTTTTTCTTATATACGAAATATGGGATTTCACTAAGCCAATTCTTATGAAATCTCGAATCAAACCCTTTGTACTTACTTCAACAACGAAAGCGCGGACCTCCTCGAGGGAAGAATTTTTGTTGTCTTGGTCCCAATTCAAGACTAAACAAGTGCGAACCAATTGAATACTTGTGTCAGAAATTCCTCGAGTTGGTTTACCATTTCCATAAAGGATATAGTTGAAAACTCGAAGTTGAATATTATCCTTTTCCCGAAAGAGATCTTGTGGGAAGAGTGTTGCTAAATTTATACTGTCCATTATCTCATAGGTAGCTACGGGCCGCACCAAAACAAAAAACTTTTTCTTGGTTGGTGTGATCCGTTGGGCATAAATCCAATTTTTTAAATTTTTTGATTCTTTAGAGTTTGTTTTTCCCCTTCCTGGCGGTATCAAGATGCCACTATGTCGGGATATCTTATCTGTCTTGTCCGGAAAATGGATATCCCCCGAAAATATTTTGAGTTCAATCCTTTTTTTTTTTCTCTCCACTCGGATCAACCCGCCGACTTGGCTTCTTATATTTAAAGTGATTCGTGTATCGACTCCAATGATACTATAGTTCTGTACCATTATGGCGGAGGATTCGGGTAAAATATGCACTTCCTCAGGAATGAAAAAAAAGCGATCTACTTTCATTTCGTATTTTGTCTTAAATTTTTGGACTCCTCGATACTCAATCATATCCTCTTTTTGGACGATTGAGTCCGCCTTTAGAGTTCCATATTTAAGAATTCCGGAACTCTTTCTTCTGTATCTAGGATCATCAAAAAAAGCAAAAATACTGTTTCTACGGAAAATACCATTTATGGGTATTTCAATCGAGATACCTGAATGTGGTATGAACTCTTTCGCTTGCTCTTGAATCGATTGGAATGGAATGAGAAATCTATTTCTTCGCCTTTTTGCTAATAAATCCGAATTCTCATGAAAAATAGCAGAATATATGAAATTATAATGACTAGTACCTACGATTCCATTCAATTCTGAATAATTGGGAATCCCAGATTTTTTTTTATCAGAAAAATCTGAACTGAAAAATTTTTTGCTCACTTGATCATTATTCACTGAGAGGCTAGAAATAGATTTTCTTTCGACGGAAAGAAAGGGTATGTTCATTTGATCTTGATCTTTGTGGATCGAAAAAAGAATTAGACTAGATCCACAAGAACCTCCTGATAATATCCATAAATGACTTGTTTTTGGTAAAAGATGGACATTACTATATGTAAATTCGGGTGCATGGGATACATCAGTACTCCAATGCATTTCGCCCTCGGAGTCAGAATAAATATATTTTCTAACCCTCTCTTTAAAATGAAAAGTGGATGTTCCCTCGCGAATCTCAGCAATCACTTGTTCTGATTCCACATATTGATCATTTTGAACTAAAAGAAAACTTTTTGGTGGAATAGTCACGCTATGTATAATATCTTCGCTCTCAATAATTACAGACAAGTCTATATAACATAGAAAGGCAGGATGCCCGTGACGTGTACGTGTAGGATGAACCAAATCCTCATTAAATTTGATTTTTCCATTATAAGGGGCTCGTACATGTTCGGCAGTACCTCCTGTAAATACTCCACCGGTATGAAAAGTTCTTAATGTTAGTTGAGTCCCCGGTTCGCCAATAGATTGACCCGCAATAATACCTACAGCTTCCCCCAATTCAACTAGGTCACCATGAGTGGGACTCCGGCCATAACATAATCGACAGATCCAAGATGTACTCCGACAAGTAAAGGGAGTTCGAATAGATATTGATTGTGTTCCAAAGGTTATGAATCGATTGACAAGTCCAATCCCAAGATCTTGATTTCGAAAGGCGACACATCGGGAACCTATATATATATCGTCTGCTAAGACACGACCAATTAATGTTTGGATAAAAATTCTTTCTGACATCATCCGACTTTTATTTCGAGGACTCACAGAAATCCCTCGGATAGTGCCACAATCCGTTCGACGTACAACAATATGTTGAACTACTTCAACAAGTCGACGCGTAAGATATCCAGCATCTGATGTGCGGACCGCAGTATCTACAACTCCTTTACGGGCTCCATAGCAAGAAATAATATATTCTGTTAAAGACAGTCCTTCGCGTAAATTGCTTTGAATAGGTAAATCAATCATTTGTCCTTGGGGATCCGACATTAATCCTCTCATACCTACTAATTGATGTACTTGAGATGCATTTCCTCTAGCTCCCGAAAAAGACATCATATGGACTGGATTGAAAGGGTCCGTCATCCTAAAATTAGGATTCATTTCCTGTCGCAAATATTCACTTGTAGCATACCATATCTCAATAGATTGGCGTAATTTTTCTACCGCATGTACATTCCCATAATGATGGTGTTTTTCCAAAATCAAACTTTGTTGTTCAGCATCTTGGACAAGCCAGCCCTTAGAAGGTATCGTTAAAAGATCATCAATTCCTAATGAAATGGATGTAGCAGTTGCTTGCTGGAAACCCAGAGTCTTTACTTGATCTAGGATGTGTGATGTATATGCCATCCCGAAGTGATCTATTAATCGGCTAATAAGTCGTTTAATAGCAGTTCCATCTATCACTTTATTGTGAAATACCAGATTGGCCCGTTCCGCCATAAGTACCTCCATATTCTGCTGAATGGGATTCGACAATGAGTTTGAGTCAATGATTGCAAAACTTCCTTTTCTCGATCTTGATTTTTTAGGTCAGGAACTATGTCCGAGTTGACTCGGAGAGGTCCGAATTCACACGGGTGTCCTATAATTCTTTTTTTATGAATACCATATTATTAGGTATCATATGAACAAGCTTGAGAAAAACCTTGTATAGCTTCCTCGATTTCTCGATAAAAAGAAATATGACCAACTGTGGTTCGAATATATATACAAAAAGTTTGTTTTTTTACACTTCTTACTATCAGATAGTGTGCATAAATCTCATGATAGTTACCAAAAGATTCATAGTGAACTTCGATAGGAACTTCTTTTGAAGCAATAACGCGTTGATCTAATTGCCACCGAAGCCACAAAGGACTATCTAAATTGATTCTTTTCTGCCGATAAGCTCCAATTGCATCATAGGAATTGCAAAAAAAGGGTTCTTTCATATACTTATAGTTTGTTTCGTAAATTCTTTCATTTTGATAGTTTTTTCGATTACATGGATTATATCTGTTTGCACAAATACCTCGACGAGTGCCGCTCGTTAATACATAGAGTCCAATCAGCATATCTTGAGTCGGTACAGAAATGGGATCTCCAATAGCTGGAGATAAGAGATTCATATGAGAAAACATAAGTAAACGAGCCTCTGCTTGAGCTTCTAAAGATAAAGGCACATGAACAGCCATTTGATCCCCATCAAAGTCTGCATTGAACCCCTTACAAACTAATGGATGTAAACAAATAGTGCGTCCTTCCACTAAAATGGGTTGGAATGACTGTATGCCTAATCTATGTAGAGTAGGTGCTCTATTCAGTAATACGGGATGCCCCTGCATAACTTCTTGAAGGATTTCCCAGACAATCGGCTTTTTTTCACGAATTTGACTCTTAGCAACTCCTATGTTCGAAGCCAGATGTTGTCTAATTAGACCACGAATTACAAATGTCTGGAAGAGCTCTATTGCTATTTCGCGAGGCAATCCACAGCGATGTAATGAAAGTGAGGGTCCAACGACAATCACCGAACGCCCCGAATAATCGACCCGTTTGCCAAGCAGAGTCTCGCGAAATCTTCCCTCTTTTCCTTCAATTACATCTGAAAATGACTTGTAAACCTTATTATGACCATCCCTCATGGGTTGTCCACGGATTCCATTATCAAGAAGTGTATCCACGGCTTCTTGTACCAATTTTTCCTGACACATTACTAATTCCCCTGGTGTAGATCTACTTGTTGTTAATAGATCAGTAAGAGTATTGTTCCGATAGATAACTCTTCTATAGAGTTCATTAATATCTGAACTCATCAGTTTACCCCCTTCTATCTGAATGATGGGTCTCAACTCGGGAGGCAGAACCGGTAAGAGACATAAAATCATCCATTCCGGTTCTATATTTGTTCGAATAAAATGCTTAGCTAATTCCATACGTCTAACTAAAAAATCTTTTCTTCTTACAATTTTTCGATCTTCCCATTCATTCCCCGTGGGACCTTCTTCTCCTAATTGTTTCCATTCTACCAACGAATTTTCTATAATAATTCGCAAATCTAAATCGGCTAATTGTTCTCGGATAGCACCCGCCCCAGTAGAAATTTCTCGATTTCTAAATATATCGAAACCTTGAGTAGTAAAAAAAAGTGGGATGCTGTATTTCCAGGATTGAATTTCATATTCAAATGAACCTCGTAATCGTAAGAAAGTAGGTTTTTTCGTTATGGGCCTAGCAAAAGAAAAATTGGGATAGGGTCCACTATATGATCTCCCCCCCTCAAAACCGGACATGAAAGTTTCCTCTCATCCGGCTCAAGTAGTTATATCAAATAAAGATAAAGAAAGGGGTCGCACTTTCCAATTGTATTTTATAAAATCAAGTGAAAACCCAAAAAGAATCTACGCCTTACTCAAGTTCTCAGTGCAAACCAACCACCATTTCATTGATTCAATTAATTCTTCTTTGATTTCTATTTAGATTCTTTAGTGAATTCAAAATTACGACAGAAAAAAATGTCAAATTCTTGAGTAGTCTACTTCCCTTCGAATGCCGGAATACTTTTTACCTTAAGTGAAAGGAATGCCTTAGAATTCATACGGGATTTATTTGTCTATGTATTGTTCCATTCGATCTTTTAGGTCCTGCGTTACCTCGATGGTTATGCCACAATATTCTTAAAGCTTATATGCGATGTATAGACTTCTCCAACCATGACATATTTGTTTACTTCAATATAAAAAACCAAATTTCTTTTCGTTTAGAAAGATAAGGGAATGCTTAATTCGACAAAAAAAAGGTCTTCTTTTCACGAGGTACGACTATCAATTTGAAGTACTTTTTTTTTACTGAATCGACCATAGACCAATCGCCCTTGTTATTTGGGAGTATTGAATACACCCACAAGTCTGAGCTTCATGTTACTCTTTTCAAGAGACATGTCAGATCGAGGGCATCCCAAATTGATTGAAGGGGATGAGAGTTTATCATTCTTAAAAATAAAAATTTCGATCAAATCACACATCGCAGTATACTAGACCTTCTAATTCTTTAAGAGGTTTATCTAAAAGATTCGCAATATAACTAGGAAGACGTTTCAAATACCATACATGAGTTACAGGACATGTCAGTTTTATGTATCCCATTTGATATCTTCGTATCCGAGAATCAACAAATTCAACTCCACATTGTTCACAAAATTGCGAGTCTTCTTTTTCATCTCCGATCACTCGATAATTTCCACAAGCGCAAATTCCACTCTTTATAGGCCCAAAAATCCTTTCACAAAATAATCCATCTTTTTCCGGTTTATTGGTTTTGTAATGAAAAGTATAGGGTTTTGTCACCTCTCCAACTATCTCTCCATTAGGTATTTTTTTAGTGGCCCAAGCACTTATTTGCTGAGGAGAAACTAATCCAATTCGGAGTTGTTGATGTTTATACCGATCGATCATATAAGAAATTTTGTGATTCATTCCGATTAAACTTCCTTCCTATTAATCTGGAAATTCTTCTCAGATACAAGGAAATGATTCAGTTCCAGAGCCAAAGATCGTAGTTCTCGAACAAGTAATCGAAAAGATTCTGGAGCATCTTCTGGTTTAGGTATTGTTCCTCCAATGATAGTGGTACCAAGTACTTCTTGGCGAGCTCTAATATGATCAGATTTATAAGTAAGCATCTCTTGTAAAATATGAGCAACACCAAACCCCTCTAGAGCCCAAACCTCCATTTCGCCTACCCGCTGCCCCCCCTGCTTAGAACGGCCTCTAAGGGGTTGTTGTGTAACAAGTGCATAATGTCCACTAGAACGTCCGTGTATTTTATCATCAACCTGATGAATTAATTTCAAGATATAGGGCTTTCCTATTATCACAGGCTGTTCAAAAGGATCTCCCGTTCTTCCATCAAAAATGCGGCTTTTTCCTGGATACTCGGGTTCAAATACCCATGGATTGGCTGTTTGCTTACTAGCTTCATATAATTCAGAAAATACGAGTTTTCTCGAAGCCTCTTGTTCATATCTCTCATCAAAAGGGGCTATTCGATAATGTCTATCTAGCAAACTTCCCGCTAACCCAAGCGAGCATTCAAATATCTGTCCTACATTCATGCGTGAGGGTACTCCTAATGGGTTGAAGACCATATCCACGGGTCTCCCGTCTTGCAAATAAGGCATATCCTGTCTAGGCAAAATTTTGGAAATGATACCTTTATTTCCATGTCTTCCGGCTACTTTATCACCTACTTTGATTTCACGTTTCTGTGAAATATATACACGAATTATTTCGGGGTTATAACTTGAACCCCCCTTTTTCTGAACCCATCTCACATCAATAACTCGACCTCTACCACCTATGGGCAATTTTAAACAAGTTTCTTTTGAAGTCGATACCTGAATGCCAAGTATGGCCCGTAATAATCTATCTTCCGGAGCATACGAGGATTCTTTCGCCATCTGAGGCGTTAATTTACCTACTAAAATATCACCCGTTTCAACCCACGATCCTAGCATCACAATTCCATTTTTGTCTAAATTTCGGAGTAAACGGCCCTCTAGATGCGGTATTTCCTTAGTGATCCTTTCAGGACCTTGGGTTGTCACATGCGTCTGAATTTCATATTTCCGTATGTGGAAAGAAGTATAAATATCACCATATACTAGACACTCACTAATGAGTACCGCATCTTCAAAATTGTATCCTTCCCATGGCATATAAGCCACTAATATATTTTTCCCCAAGGCGAGTTCCCCACCAACTGTAGCAGCACCATCTGCTAAAATCTGTCCCTTTTTAATACATTTACCCCGGCGAACCTGAGGTTTTTGATGCATACAAGTATTTTTGTTTGAGCGTTGATACATAATTAATGGAATACTTAAAGTATTCTCATTTCCCGATAAAATTATCTTCTCAGTGTCAGTATAAAGGATTTTTCCCTCGTGTTCGGCTATAGCGGGAACCCCCGAATCTAAAGCCACTTGGCGTTCCAATCCAGTTCCAACAATGCACTTTTCGGACCGAGAAAGTGGAACTGCTTGACGTTGCATATTAGAACTCATTAAAGCTCGATTCGCATCATTATGTTCGATAAAAGGAATTAGGGAAGCTCCAATGGAAAAATATTGGAAAGGAAAAATGCTTCGAAGATGAACCTCTTCCCATGCGATAGTCAAAAATTCTTGGCGGTATCGAGCTGGTACAGCCTGTTCTTCTTGAATGCCCCGATTAAGAGCCAAAGAATTTCCTGCCGCTATCATATAATATTCATCTTGACTTGGTGATAAAAAAAGCATCCGTATCCGCGCCTTTTTTGATTTCTCAACGAGTTCATAAAACGGACTTTCTAACGACCCCCAATCACCAATCCTGGCATGAATTGATAAAGATCCAATAAGTCCCACATTGATTCCTTCAGACGTGTCAATGGGGCAAATACGCCCGTAGTGACTAGGATGGATATCTCGTATTCGAAAATTAGCAGTTCGCCCTGTTAATCCGCCAGGGCCCAAATAACTCAACTTTCTCCCATGAACGATTTGTGTCAATGGATTAGTGCGATCCAAAACTTGAGATAATGGATGTAATCCGAAAAAGGATTCATAAGTAGTTGTTAACGGAGTTGAAGTTACCAAATTCTGAGGAGTAGGTATCAATTTATGCCTAATTGCTCCGCCTATAGTTCCCTTAACTACATTTTCTAAACGAGCCAGAGCCAACCCGAGCTGGTCTTGTAAAAGATCCGCTACAGAGCGAATACGTTTATTTTTCAAATGATTCATATCATCAAGTGTACCCATTCCAAATTTCATCCCAATCAAATGATCGGCAGCTGCTAATATATCTCGTGGTAACAAAAATATATTGTTCTGAGGTATATTAAGATTAAGTCTCCAGTTAATATTTCGGCGACCAATCCTCCCCAATTCACACCTTTGGTGAAAGAATTTTTTTTGTAATTCCTTACATAAGGATTCAGAAAATATTGGATCCCCACCTACACAAGAAAATTGTTGATAAAACTCCAAAATAGCATTTTCTTTTGACCCAATTTTTTTTTTCTCCTTATCGGTTAAGAAAGATAAGAAAATTTCAGGGTAGCAAACATTCTCTAGAATTTCTCTTAGATTCGAACCCATAGCTGATGATAGAACTAGAATAGATATTTTCTGTTTCCTACTCACACGAGCCCATATTCTTGCTTTTTTATCAATCTCTAATTCTAGCCTGCCCCCCCAATCTGATATTATGGTGCCGGTATAGACCGAAATCCCGTTATGATCCAATTCTGACTGGTAATAGATACCAGGACTTTGTAATATTTGATTGATCACAACTCGGTATATTCCGTTTACTATAGAAGTTCCAAGGGAATTCATTAAAGGAATGTTTCCAATAAAAATTCTTTGTTCTTGCATATTCCTATTGGTTTTCCAAATTAATCCCGCGGATACATATAATTCAGAAGAATATGTAAGTAATTCATAGACAGCATCTCGTTCTTTTATCAGAGGTTCTACCAATTGATATGTTTCCACAAATAATTGAAATTCAATTTCGTGATCTATATCTTCAATTTTTGGAAATTGCGAAAGTTCTTCTATTAAACCCTGATCAATAAACCGATAAAACCCTTCAAATTGTATCTGATTAAATCCGGGTATTGTAGATGTTCCCTCTTTTCCATCCCCGAGCATCTTTTTTGAATTTATCATTTATCCGTTTATTTTAAAAATCCCATATCTCATTCTTCACCGAATCATATAGATAGAATTCGATCTAGCAATAATGGAATTTCTATTCTGTTTACTGAATCACATGAAATTTTATCCAACTCCAAGATATATGGAATGTATGAAATCCGTATGAACGGAGACTAGATTCAATTGGAATTTTTTTTATAAGAAAGAGATCCGAATGGAACAGAATTTAGAAATACCGCTGGAACTTATGGAGTTTTGTAACGACTAGAAAAAAAGTAATTTCATTTTCACCTATGATATTACATATTCCAATTCGATCGCATACCATAAAAAACGGTATTCATGATAGGATCTGTTCGAGCAGATAAACATATAAGAAATAGAAAACTTTTTTTTAAACACTTTACTTTTTCATGTATTTGTATTTCATTGTTCAAAAAAATAGTTGCAGAAAAAAGATGGATTTTTACCTATTTTGAATAGAATATTTAGAATATCATTGAATTGAAGTAGGTAAGAAAACGAATTGAAGTAGGTAAGAAAACGTATGTTTTTTTATTTATTAATTTTAATTATTATTAAAATAAAAAAGAATGCACAGGTATATATATATGTCTCTTTTTCTTCTTTTATTGTGGTACAGTTCTATTTGGAACAGCACATGCTGTGCTCTACCAAAAATTAAAAATTTTTTTCAATGTATTCAATGAAAAATTTCAATACAAAAATTTATTGAGAATTACTCCTCAAAAGCATCCCTAGAGAGATAAAATACCCCATTATAGAGCTATACAAGGTAACGTATGTTCTGATTCTGGGGTTTACATATACTCATTATTAGTGTTATAATTCAAATGGAAGAAGATTTCTTTTTAATTGAAAAAACTCAAAATAGATTAGTTATAAATCTATTTCTAATGATTTTCTTATCTTATATTATTAGAAATAAAAAATGTAAATTTGAATTCAAAAAAGGTCATGAATTTACAGTCAATAGTTAATGGTTCTGATTTGTACTAGATTCTATATTTTGTGACTGAAAATCTATATTTTTTTCGGAGTTGAAAAAAAAACAAGAGAAAATTTGAATCTAGTACAAATCATTTTGGCGGCATGGCCGAGTGGTAAGGCGGGGGACTGCAAATCCTTTTTCCCCAGTTCAAATCCGGGTGCCGCCTCAACAGGAGACTTGAAATCTCCTGTTATAAAACTATAACAAACGTAGGAAAAGACTCTTGATACTTTCTTTTCGTGATTCTAAGCCCCTGGCTCTCGAGGTTCTATTCTCTAACCTAAAGTTTTACCTATCAGATTAGAGGAAAACTAAAACGAGTGGAGGGAAATCCATTAGATTGGATAGGCAGAGAGGGAATTAAATTAATAGTTTTGGAAAGGATCTAAGATACTTTGGATATAGACTCATGAAAGTGGATATAGACTCATGAAAGTGGATATAGACTCATGAAAGTGGATATAGACTCATGAAAGTGTCGGAATGCTCAGACATTCAATCAATATTAGATTAGATGAAGAATTGCCTTTCGTTTTACTTCAAATAAAAATAAAAAACGATAAAAGAAAGAAAAAAGATTATTCTTTCTACATATGAGTCAGATTTTTTGGATACTTCGAAAAGTATCTGTTTACTTGTGTTTACATCTTGTCGATTCTACTAGAAATTCTATAATTAAGAATAACTCAGTATAAGATAAGTGGATTTTTTGGAGTAGTTCATCAATGGTGACCAAATATCTCTCCCTTTTTTTGACTCTGCACCAGTGATTTCACTATTATTAGTGAACAATAATGGAAAAGTTTCTTCATATTCATAGGGGACAGAATTCACATGGATATAGTAAGTCTCGCATGGGCTGGTTTAATGGTAGTTTTTACATTTTCCCTCTCTCTCGTAGTGTGGGGAAGAAGTGGACTCTAGAAGTACTCCTAATTGCGATAATAATCAAACTCTATCAACCTGTATCAATTGTTTTAGTTTTCTAGACCGGCCGGCAATTTTTTTTAAGATCTTTTTTTAGAAATTGGATTTATGTTTTGTTTTATTGACTCATTTTATTTTTTGATATCAGAGTTTATACCGTTAACCATTCATGGGATAACCCCCTTTCGAAATCTCAAGAGGTTTCCATCGAATTCGGATTATCCGTATTAAATGGATCAAACAAACAAATGAAATTGAGAAAGTATGTACATAGATTTCATATTCTATATTAATTTATATTTACATTTATAAAGAAAAAGAGAGATATGGGTGGATTCCTTTATATTAAGATATTTCACTTGTATCTTTATACATTACAATAACCATAATGGCTAGTATGGTAGAAAGAGATCTCTTTCTACCATACTAGCGGGCCCCTTAGGATACTACTGAATCTAATGCATTCCTTTCATTTAAGACGAGAAATTGACATCCTTTTTTGTCATTGATAGTCAAATTGTATTCAAATTAATTATTTTGACTAACCGTTTTTACGTAAATTATAAGCAAAAAAGCAGTAGGAACGAGAATGAAGAGTGCAGTAGCAATAAATGCAAGAATATTGACTTCCATAATTAAATCGTTTATTATTTATTTTTTTTTCTTTGGAATATCTCGGGATTTAATCCCATAGAGATGAGAAATCTTTCGCTTGTAAACTCACTCAGATGAATTAGATTTCGATGATATCGAATGAAAGAAATATCATGAATAACAATATCGGAGCTATAAAATCGATTCATCGTCAAGAATTTAATAGTATAACATAGGAAGATCTTTTATCCACACCGAATACATAATGAGATTCCTGATCCAATAAAAAACTATTTATTTATGATTCTTTTTCACCGCTTTCTTTTCTACAACCTAGTACTTTCCTTGTACAATCATCTGATGAAATATCATAAAAAACCTTTTATACTTCGATTGTTTATAAAAAGAGTTTCTAAAGAACCTTAAATAAACAATAGAAATCAAATAGAGAAAACAAGTACGAAATTTCAATTTGAAATTTTCAAAATTTTGTTTTTGTAAGGGTCTATGATCTTTTTGTAAAACAAAGGAAATGTGATAAAGACGAGTCCCGAAAAAAAAAAACGAAAATATTCCAAAAAATTAACTATATTAAATTTTCTTACGAGTTTTTCTTCGACATCGACTCTAATCTTTAAAAAGAGCATATTCATTAGGGAAGACTAATTTGATCTTTTTTTTTGAAACATCCTCTTTACTTGGTTGGATTCGAACTATTTTCACTTCCTTGACTTCATAGAAACAAAAGTATATATAGGTACTCTTGGCAAACGTATTATACGCTATCCTATTTTATTTTCCTACACGAGTTAATGGGAGATTAATTGACAAAAAGAGGAAACCCCATACAGTATCTCGTTCTTGAAGTGGTGAATGCTCTCAATAATTATAATTATACTAATTTACATATGTCTTTAAATTGGTGCAAAAGAAATACCCCTTTCTTTTGCTTGATGAAAAAAGAAAAATAAAACAAAAAGATAACCGAAACCATTTTGATCCCCTTGCCCAGAAACAAAAAGGGGAGTTTATGTCTTTTTTTTTAATTGAATCCGCCGGGACTGACGGGGCTCGAACCCGCAGCTTCCGCCTTGACAGGGCGGTGCTCTGACCAATTGAACTACAATCCCATGGAAATAAAGCGGGTAGCTTACATATTCCTTCTTATGATTTCATCATAATCATTTCAATTTTAGATTCAAATTAGTGTTTTGTAACAAAGAAAATCACAAGTAATATATTGATATCTATATGGATATCACTAAAGTGATATCAAGGCCGATTACTAGTAATCCTTGCATTATTCTAAAATCGATTGATAATCTATTTTTTATTGTAATTTTTTATGGAAACAAAAAGTAACGAGCCACAAGAAATAAAGAAAAAAGTAAAGTCGAAATATACCCAGATATTTGACTTTTTTCTTACCCTTCTCTGTCAATTATGCAAAACAAAAAAGGTTATGTAGACAGCGAATTATTGGGCCGAGCTGGATTTGAACCAGCGTAGACATATTGCCAACGAATTTACAGTCCGTCCCCATTAACCGCTCGGGCATCGACCCAGGAAGAATCTATTCGAACTTTATGGATAATCCATGATCAACTTCCTTTCGTAGTACCCTACCCCCAGGGGAAGTCGAATCCCCGCTGCCTCCTTGAAAGAGAGATGTCCTGAACCACTAGACGATGGGGGCATACTTGCTCAACCGCCATCATACTATGATCATAGTATGATCAGTTTTTTAAAATTGTCAATATAATCAAATGGTATGACTAGCTTATAAGATTTTTTATTTTTTTCTATAGCATTCTATATCATTTTTTTATTTTACATTTATATTCATATTCTAATCACAATTCTATAAAAAAAATCGATATATTTTCTTTTTATATTTGAAGTGGAAATATGAAAAAAAAAAAAAAATCGAAAAAAAGTCTAGTATAAAATCTTTTAAAGAAGTGATTGGTCTGACAGAAAAAAAATAAAAAAGAGGGTTAAGTTTCGTTTTTTTTACTTTACTTAATAGATTGCCTCATCTCATTGTTAAGAAATAGTAGTGTCCCTATCTAACACTAACCCAAGAAAGTCAGACAGAATCCATCTTTCTTCCCTAATTAGACGATGGATTGATAAGTTAAGTTATCGATTCTCGCTTCTAGTTGCGAAATGAGCTACTAACCACTATGCGTCTATTGTATATATATTTAATATATATATATATTTGATTTACCTATCGACTCAGTCAGGAATTAAATCAAGACGGCCCTTTTAACTCAGTGGTAGAGTAACGCCATGGTAAGGCGTAAGTCATCGGTTCAAATCCGATAAGGGGCTTTTACTTTCTTTAACTTTCTATTACTTTCTTTAACTTTCTATATAGGAAAAATTTCATTCGAAAGTCTATAATTTCGAATTTTTTGAATTTCATTCTAATGAATTTCATTTTAATAATAACTAATAATAAAGTGAGAGAGTTTAATAATAACTAATAATAAAGTGAGAGAGTAATTTAGAAAATCAAATTGAACATTTTTATATTATAATACAATGAATAATAATAAGTCGGCTTTTGAATCGCCAAATAGATATTCGTTGTTTCCCTTTTTCGATAGATTAGAAATCAACAAATCCAAAAGAAAAAGTAAGTGGACCTAACCCGTCGAATCATGACTATATCCACTATTCTGATATTCAAATTCGATAGAGATAAAATTGAAACAGTAGATTTGTTTTATTTCATATTTTTTTATTCGGAAATCTGTCGATATCTCTTATTTAATCTTCTTGTTTCTATATTTCATAGGAAATATATTGCGTTCCTGCCTAGAGAAAGAAAGTCTTATTCCAAATTTTTTAATACCTAAAGGGTATTTCAATATCTTGTTTTGATTCCAGAACATAACAAGAGCCTAAATTCTAGTTGTATAAGAATCAAATTGTATTAAGAATCAAAAAATCGAATCATAAAGAATGGCTTCAGATATCAATCAAATATTTCCATATTGATGCTTACAAGATGACAATGTAATGGGATTGAAGGTGTATGTGAGAAAGAAACTCTCATTTACAGTTTGCTATTATTTTATTTAAATATTGTATTGAATTAGATATAAATAATAAATTTTCCCTTTTTTTACCGGCATGGACATGTAGATATCAAATAAAATAGAAAAAAAGATTTCTTTATCTGAGTAATGAGTCATCTGACAATTCATGATTTAGATTCAACTACTTATTAAGAAACTAATAGCAAGGAAGAAACAATTTGAGTTGATGCGTTTACCTAAGTAAGGACCAATAAAATCAAATATTTTGATCTTCGAAACCAATTAAATGAAATTCTAAAGGTTAAATTTTATGGGGCAGTGCGCGAGAAATCAAATCATAAATAAATGATAGAATTTTGAGCGTCCTGAACATAATATATAACATTAAGATATATAAAGGTGTTCGGAAATGGTTGAAGTAGATGAATAGGAGGATCGCTATGACTATAGCCCTTGGTAAATTTACCAAAGACGAAAAAGATTTATTTGATATTATGGATGACTGGTTACGGAGGGACCGCTTCGTTTTTGTAGGTTGGTCTGGTCTATTGCTCTTTCCTTGTGCCTATTTCGCTTTGGGGGGTTGGTTCACAGGTACAACCTTTGTAACTTCATGGTATACTCATGGATTGGCTAGTTCCTATTTAGAAGGTTGCAATTTTTTAACCGCTGCAGTTTCTACTCCTGCTAATAGTTTAGCGCATTCTTTGTTGTTACTGTGGGGTCCTGAAGCACAAGGAGATTTTACTCGTTGGTGTCAATTAGGCGGTCTGTGGGCTTTTGTTGCTCTCCACGGTGCTTTCGCATTAATAGGTTTTATGTTACGTCAATTTGAACTTGCTCGATCTGTTCAATTGCGACCTTATAATGCAATCGCATTCTCTGGTCCAATTGCTGTTTTTGTTTCTGTCTTTCTAATTTATCCACTAGGTCAATCTGGTTGGTTCTTTGCGCCTAGTTTTGGTGTAGCGGCTATATTTCGATTCATCCTCTTTTTCCAAGGGTTTCATAATTGGACATTGAACCCATTTCATATGATGGGAGTCGCTGGTGTACTGGGCGCGGCTCTGTTATGCGCTATTCATGGTGCTACTGTAGAAAATACTTTATTTGAAGATGGTGATGGTGCAAATACATTCCGTGCTTTTAACCCAACTCAAGCCGAAGAAACTTATTCAATGGTCACCGCTAACCGCTTTTGGTCACAAATCTTTGGGGTTGCTTTTTCCAATAAACGTTGGTTACATTTCTTTATGTTATTTGTACCAGTAACTGGTTTATGGATGAGTGCTCTTGGAGTAGTCGGTCTAGCTTTGAACCTACGTGCCTATGACTTCGTTTCCCAGGAAATCCGTGCAGCGGAAGATCCGGAATTTGAGACTTTCTATACTAAAAATATTCTTTTAAACGAAGGTATTCGCGCTTGGATGGCGGCTCAAGATCAGCCTCATGAAAACCTTATATTCCCTGAGGAGGTTCTACCACGTGGAAACGCTCTTTAATGGAACTTTAGCTTTAGCTGGTCGTGACCAAGAAACCACTGGTTTCGCTTGGTGGGCCGGGAATGCCCGACTTATCAATTTATCTGGTAAACTATTGGGAGCTCATGTAGCCCATGCCGGATTAATCGTATTCTGGGCCGGAGCAATGAACTTATTTGAAGTGGCTCATTTTGTACCTGAAAAGCCCATGTATGAACAAGGATTGATTTTACTTCCCCACCTAGCCACTTTAGGCTGGGGGGTAGGTCCTGGGGGAGAAGTTATAGACACCTTTCCATACTTTGTATCTGGAGTACTTCACTTAATTTCTTCTGCAGTTTTGGGCTTTGGCGGTATTTATCATGCACTTCTGGGACCCGAAACTCTTGAAGAATCTTTTCCATTTTTCGGTTATGTATGGAAAGATAGAAATAAAATGACCACCATTTTGGGTATTCACTTAATTTTGTTAGGTGTAGGTGCTTTTCTTCTAGTATTCAAGGCTCTCTATTTTGGGGGCGTATATGATACCTGGGCTCCAGGAGGGGGGGATGTAAGAAAAATTACAAACTTGACTCTTAGCCCAAGTGTTATATTTGGTTATTTACTAAAATCTCCCTTTGGGGGAGAAGGATGGATTGTTAGTGTGGACGATTTGGAAGATATAATTGGAGGGCATGTATGGTTAGGTTCCATTTGTATATTTGGTGGAATCTGGCATATCTTAACCAAACCTTTTGCATGGGCTCGCCGCGCACTTGTATGGTCTGGGGAGGCTTACTTGTCTTATAGTTTAGCTGCTTTATCTGTTTGTGGTTTCATTGCTTGTTGTTTTGTCTGGTTTAATAATACTGCTTACCCTAGTGAGTTTTACGGACCTACAGGGCCAGAAGCTTCTCAAGCTCAAGCATTTACTTTTCTAGTTAGAGACCAACGTCTTGGAGCTAACGTGGGGTCTGCTCAAGGACCTACAGGTTTAGGTAAATACTTAATGCGTTCCCCGACTGGAGAAGTTATTTTTGGAGGAGAAACAATGCGTTTTTGGGATCTGCGTGCTCCCTGGTTAGAACCTTTAAGGGGTCCTAATGGTTTGGACTTAAGTAGGTTGAAAAAAGACATACAACCTTGGCAAGAACGACGTTCTGCAGAATATATGACTCATGCTCCTTTAGGTTCCTTAAATTCTGTAGGGGGCGTAGCTACTGAGATCAATGCAGTCAATTACGTCTCTCCGAGAAGTTGGTTATCTACCTCTCATTTTGTTCTAGGATTCTTCCTATTCGTGGGTCATTTATGGCACGCGGGAAGAGCTCGGGCAGCGGCAGCAGGATTTGAAAAAGGAATTGATCGTGATTTTGAACCTGTTCTTTCTATGACTCCTCTTAACTAAAGTAGTAGTTAAAATAGGAAAGTAAAAATCGGGTCATATTAAAAAGTCTTCTTTCTTTCAATTCAATCTCGTTTTTTCTGGCTCGGCTGGATAGTATAGCCGAGCCATTCTCCTTTTTTATGATGCTAAGAAGTAAAAAAAGCCAATAAAGAAAAAAATCTATTCATCCAACAAAAGGAGAGAGAGGGATTCGAACCCTCGATAGTTATTTTTTATGAACTATACCGGTTTTCAAGACCGGAGCCATCAACCACTCGGCCATCTCTCCAAAAGATAATTTCTATTTTATCTTTTTTTTCGCCAAATAGAACATAGCTCGATGAGTTAATACGATCACTATGTAGAAAAAGATATAGGGTGTGACTTTCTTTATAAGTCTATCAATTGGTCTATATAAATGAGATACATGATCCAGTCTACCCATTTGTGAAGTAAAAAAGAACCTTTAACTTCATGTCCGAATAGAATAAAAGTGGTAAAAAGAAGTTGGAAATAAGGCATCTCGAATAAACGGATTCATGATAAAATCCCTTTATTTATTAAAATTTTTTTAGTGGGTAAGAGGATTAAATGGTGTATATTGTTAATAGCTTGGAGGATTAAAAACATGACTATTGCTTTTCAATTGGCTGTTTTTGCATTAATTATTACTTCATCAATCTTACTGATTAGTGTACCCGTTGTATTTGCGTCTCCTGATGGGTGGTCGAGTAACAAAAATGTTGTATTTTCTGGTACATCTTTATGGATTGGATTAGTCTTCTTGGTGGGTATCCTTAATTCTCTTATCTCTTGAATTCATTCGTTGCAGATCAAAAAATGAGATGACCCCTCCCATTCCACGAATTACACATTCAAATTCAATATAAGTCCATAAAATGCAAATAAAGAAAACAAAAAAATTAGAGGGGGGGTCGAACTTCTGTAACTTGAGTGAAATATGAATCAAATATTAATAAATAGCAATTTACTAAATATAACTATGAAATAGTAATAACTAATTAAATAAAAAAAAAACGAATCAAAAATTGATATCTGATATCAATATAGAAAATAATATTTTATGGAAATAGAGAATAATATATTATTGAATATGGAATTCTATATATAGATATAGAATAAATATATTATTAATATATAATAAATATATATATATTTATATATATTAATAGAATTGTTAATTGAACTTTTTTGTTTTTTGGTAGTAGAGTTTTATCAAATGACCCCAAACCAAAGAGTGTATCTCGTATAGCTTTGAACAAATATTATCCATAAATTTCTTATCAAGAAGGCAAAAAAATGCGGATATAGTCGAATGGTAAAATTTCTCTTTGCCAAGGAGAAGACGCGGGTTCGATTCCCGCTATCCGCCCAAATAGAAATGGATTCAAAAAGATCAAAGATTCGGTATAGTTGACCGGGAAATATAGTAATTTTTGCCTCGCGTCCCAAAAGATAAGTATTAATTATAGTTAATAGAATCAAACTTACATTTGTTGAAAAAAAAATGTTGCGGAGACAGGATTTGAACCCGTGACCTCAAGGTTATGAGCCTTGCGAGCTACCAAACTGCTCTACCCCGCGATGAAACAAAAAAACTTGGACTAAACTCTAATAAACAAAGACGAATTGAATGCGCCCCTATTCCATATCTGTACAAATAGAATAGCCTATTTAGACAGAATGGTAAAGGGGCCTCGTCGAGCATAGAAAAAATAGAAAAATTAAAGGATACTTAAATCTTTACCAGCTTGATCTTGTTGCCCCTGGCAATAAACATGCCTGAACCATTTCCCGAAGGATGTGTCCAGATAGTCCAAAGTCTCGATAGTTAGCTCTCGGTCTTCCGGTCGAGAAGCAACGTCGATGAAGACGTGTAGGTGCACTATTACGCGGTGGGGATTGTAATTTTCCATGAATTTTCCACTTCTCACTTAGCGACGGAATCTCACTTATTTCCTTTTTTAAGGATCGACGAATCAAATGATATTTTTGTTCTAATTTTTGCCTCTTCTTCTCCCTATAAATCAAACTTTTCTTTGCCATAATGCTTAAGTTCCTCTTATTATCAATGATAATGATACAAATCGGATCCTAGATGTAGAAATAAATATAAGAGTGCATACCTATATTTTTATTATTTTAATAAAATTAATAAAAAAAAATATATTATTGCGGATAGAATAATTAAATAATTAACCGAATTTGCCCGACGTGGAGGCAATCAAGAAAGCCGCATAAGTGAATATATAACCTACAGAAAAGTGAGCTAATCCAACCAATCTTGCTTGCACAATTGAAAGAGCTACTGGTTTATCTTTCCATCGAATCAAATTTGCCAAAGGTGTACGTTCATGAGCCCATGCTAAAGTTTCAATCAATTCCTGCCAATAACCACGCCAGGAAATTAAGAACATAAATCCAGTAGCCCAAACAAGATGCCCAAATAAGAACATCCATGCCCAGACTGATAAACTATTCATACCAAACGGGTTATATCCATTGATAAGTTGTGAAGAGTTTAACCATAGATAATCTCTTAACCATCCCATCAAATAAGTGGAAGATTCATTAAACTGTGAAACGTTACCTTGCCATAATGTGATGTGTTTCCAATGCCAATAAAAAGTAACCCATCCAATAGTATTTAACATCCAAAAAACTGCCAAATAAAATGCGTCCCAAGCCGAAATATCACAAGTACCACCTCGTCCCGGACCATCGCAAGGAAAACTATACCCGAAATCCTTTTTATCTGGCATTAACTTGGAACCACGTGCATCTAAAGCACCTTTTACTAAGATCAATGTAGTTGTATGTAAACCTAAAGCAATAGCATGATGAACCAAGAAATCTCCAGGACCTATTGTTAAGAATAATGAATTACTATTCTCATTAATAGCATTTAACCAGCCGGGCAACCATATGCTTCGACCCGCATTAAATGCTGGGCCATTTGTCGAAGATAAAAGTACATCAAATCCATATGAAGTTTTCCCATGAGCGGATTGTATCCATTGGGCAAATATGGGTTCGATCAAGATTTGTTTTTCGGGAGTACCAAAAGCAAGCATGACGTCATTATGAACATAAAGTCCCAAAGTATGGAACCCTAGAAAGAGGCTGGCCCAACTTAAATGGGATATGATAGCTTCTTTATGGTCTAACATTCTTGCCAATACGTTATCCTCATTCTGTTCTGGATTGTAATCTCTAATAAAAAATATAGCTCCATGAGCAAAAGCTCCTGTCATGATGAATCCTGCAATGTATTGGTGATGGGTATATAACGCAGCTTGAGTCGTAAAATCTTGCGCTATGAACGCATAAGCAGGTAAAGAGTACATGTGTTGAGCTACCAAGGAAGTAATAACTCCTAAGGAGGCTAGAGCAAGGCCTAATTGAAAATGAATCGAATTATTGATTGTGTCATAAAGACCCTTATGCCCACGCCCCAACCGTCCTCCCGGAGGAATATGTGCTTCTAAAAGATCTTTTATACTGTGTCCGATTCCAAAGTTAGTTCTATACATATGACCCGCAATGAGGAAAAGAATTGCGATAGCTAGATGATGATGTGCCATATCGGTTAGCCATAAACTTTGCGTTTGTGGATGGAATCCCCCAAGAAGGGTTAGAATGGCAGTTCCTGATCCTTGGGAGGTACCAAATAAATGACTACTTGAATCGGGGTTTTGAGCATACAGATTCCACTGACCCGTAAAAAGTGGGCCTAACCCTTGGGGATGCGGTAATACACTTAAGAAATTATTCCATCGAACATATTCCCCCCTGGATGCAGGAATAGCGACATGTACTAAATGACCTGTCCAAGCCAAGGAGCTTACCCCGAATAGTCCTGACAAATGATGATTCAGACGAGATTCAGCATTTTTGAACCATGAAACTCTTGGTTTCCATTTTGGTTGTAGGTGTAACCAACCCCCTATTAAGGATAGGGCAGAAAGAAATAATAGAAAAAGAGCTCCAGTATAAAGATCTTCATTAGTACGTAAACCGATTGTATACCACCACTGATAAACACCAGAATAAGCTATATTCACCGGGCCAAGAGCACCTCCTCGAGTAAATGCTTCCACAGCCGGTTGACCAAAATGAGGATCCCAAATAGCATGAGCAATCGGTCTTACATGTAAAGGGTCTTGTATCCATGTCTCAAAATTTCCTTGCCAAGCTACATGAAACAAATTTCCGGAAGTCCACAGAAAAATTATTGCTAATTGCCCGAAATGAGAAGCAAAAATATTCTGATAAAGACGTTCTTCAGTAATATCATCATGACTCTCGAAGTCATGTGCGGTAGCAATACCAAACCAAATACGACGAGTAGTGGGGTCCTGAGCTAAGCCTTGGCTAAACCTTGGAAATCTTAATGCCATAATGCCTTTCAAATCCTCCTAGCCATTATCCTACTGCAATAATTCTTGCTAAGAAGAACGCCCATGTTGTGGCAATTCCACCCAGAAGGTAATGGGTTACTCCTACAGCACGTCCTTGTACAATGCTCAAGGCTCTAGGCTGAGTAGCAGGAGCAACTTTTAATTTATTATGAGCCCAAACAATGGATTCAATAAGTTCTTGCCAATAACCACGCCCGCTGAATAGAAACATTAAACTGAAAGCCCATACAAAATGAGCACCTAGGAAAAAAAGACCATATGCAGATAACGAAGAACCATAAGATTGAATTACCTGAGATGCTTGTGCCCATAAGAAATCGCGGAGCCACCCATTAATAGTAATGGAACTCTGTGCAAAGTTTCCTCCGGTAATATGAGTTACCACCCCTTGATCGCTTATACTACCCCAAACATCTGACTGCATTTTCCAACTGAAATGGAATATTACTACCGAAATAGAATTGTACATCCAGAATAGTCCTAAGAAGACATGATCCCAAGCAGATACTTGACACGTTCCTCCTCTTCCAGGCCCATCACAAGGGAAACGAAAACCAAGATTTGCTTTATCTGGTATTAACCGCGAGCTACGAGCAAATAAAACACCTTTCAACAGTATCAATACCGTCACATGAATTGTAAATGCATGAATATGATGTACCAAAAAGTCGGCCGTTCCTAATGGAATAGGTAGCAAAGCTACTTTGCCACCCACTGCTACTAACTCACCGCCCCCCCAAGTCAAACTGGTGCTCGCTGTTTCACCAGGGGCTGTTACACCAGGTGCTAAAGCATGGGTATTTTGTATCCATTGAGCAAAGACTGGTTGTAATTGTATAGCAGTATCTGAAAACATATCTTGTGGACGCCCTAAAGCACTCATGGTATCATTATGAATATACAAACCAAAACTGTGGAAGCCTAGAAATATACATACCCAGTTGAGGTGTGATATGATTGCATCGCGATGCCTCAGGACACGATCTAATAAATCGTTGTATCGATTAGTTGGATCATAGTCTCTTACCATAAAAATGGCTGCATGCGCAGCAGCACCAACTATGAGAAATCCACCAATCCACATGTGATGTGTGAACAATGATAGTTGTGTAGCATAGTCAGTAGCTAGATATGGATAAGGGGGCATGGAATACATATGGTGAGCTACAACAATAGTTAAAGAGCCTAACATAGCCAGGTTAAGAGATAATTGAGCATGCCATGATGTTGTTAGAATTTCATATAGACCTTTATGGCCTTGGCCTGTAAATGGACCTTTATGAGCCTCTAAAATATCTTTTAGACCATGACCAATACCCCAGTTGGTCCTATACATATGACCTGCTATTAGGAAAAGAATTGCGATAGCTAAATGATGATGTGCTGTATCGGTTAACCATAGACCCCCGGTCACTGGATCTAATCCACCACGAAAAGTAAGAAAGTCTGAGTATTTTGACCAATTCAAGGTAAAAAAGGGGGTTGCTCCTTCAGCAAAACTTGGATAAAGTTGAGCCAAAAGATCCCGATTCAAGATAAATTCATGAGGAAGTGGTATTTCTTTAGGATCTACTCCAGCATTTAGAAATTGGTTAATCGGTAAAGATACATGTACTTGATGTCCTGCCCACGAAAGGGACCCAAGTCCTAGTAGCCCTGCTAAATGGTGATTCAACATAGATTCTACATCTTGGAACCAAGCCAATTTTGGAGCTGCTTTGTGATAATGGAACCAACCAGCAAAAAGCATTAAGGCTGCGAAGACCAATGCGCCAATTGCGGTACAATAAAGTTGTAATTCACTAGTTATTCCAGATGCTCGCCAAAGCTGAAAAAAGCCAGAGGTTATTTGTATTCCTCGGAAGCCTCCGCCCACATCTCCATTCAGGATTTCTTGGCCCACTATTGGCCAAACCACCTGAGCACTAGGTCCAATGTGAGTAGGATCACTCAGCCATGCTTCATAATTGGAAAAACGAGCACCGTGGAAATACATGCCACTCAGCCAAAGAAAGATGATAGAGAGTTGGCCAAAATGGGCACTAAATACTTTTCGAGAGATTTCCTCCAAATCACTGGTATGACTATCAAAATCGTGAGCATCAGCATGTAGGTTCCAGATCCAAGTGGTAGTATCAGGTCCCTTAGCTATTGTTCTTGAGAAATGACCGGGTTTAGCCCATTCCTCGAAAGAAGTTTTTATGGGATCCCTATCTACCAAAATTTTGACTTCTGGTTCCGGCGAACGAATAATCATTGAGTCCTCCTCTTTCCGGACAACACATACAAAGAAACCCGCCAACAGTCACTCAAATAATTAGTGAACCGATGATAGATGCTTAGAATTTTGTTCTTTCTCTTCTATCTCCCATCTATTCATCCATTTTCTTTAGTTATTCACTAGAGCAATTATGATCTGGAAGTCGATCTGGGGCAAGTGTTCGGATCTATTATGACATATCCATAGGGTGCTCAACGGACCCCCCCCTTTTTTTTTTTTATTAAAAAGCGTTTTCGCACCTTTACATTAGTATTGGTACACAAATAATTTTTTTTTATAACCTAATCTAGTGTATTCATATTTCAATTATAAGTTCCGAAATATAGCCTATTTTTTATGTTTTAAATAGAGGATATTATCCTATTTCAATAACCGCTTATTAGTCATTACTAAGAAACATTCTAGTATTGATATTTAGTCATTTTCAAATCCCTTTTATTCGTTTTAATAGTCGAAAAGAAAAAAATAGAAAAAACTAGATATAGATATTATAGATATTCTCATATTCATGTACTACTTATCCCTAGAGAATACCAGATTAAATAGAACGATTTGAGAAAAGGATATAATGAAATTTTTTTCTGGGATTGGTTCTTCTGATAGAAAAAAGAATCTGGTTTATTTGACCGAGAGGGCCAAGAAACTAAAAAACAATTAATTGTAAAAACAAATAAAGATATATTATAGAATAAAAAAAAAAGAAACAAAGAAAAAGTTCTTATTCGAAGCGCCTCGTGATCGTCAACCAATTCTGTGCTTCAATATAATTACCAGGAGTAAGCGTTATAGCCTGTTTCCAATACTCGGCGGCTTGAGCAAACCAAGCCTCCGCCATTTCAGAATCTCCTTGTTGAATGGCCTGTTCTCCACGGTCGGAATAGGCGGGTCAATTCCCTCCCTGAGAACCGTACTTGAGAGTTTCCTACCTCATACGGCTCGACAACCAACTCTTTTGTTTTGGTGTACCAGTTTTTTTCACTTTAACCTACTTTAACTTTATATCTAATTGAATGAGATTTCTTATAGATATTCATTCGGTTTTTCTTGGATTAAACAAAAGAGAGTAATTACATGAGTTTCAAACTTTCGTTTTGATTTAATTAATATATTAATTAATCTAATAATAAGTTTTATCTTTTCTCCTACCTTCAGAAAAAAAAAGGCATGTCCACTGTTATTAGATATTAGAATTTTCTGAAAGGTAACTATCCCGCTTTCATATATAAATTTATATAGAATCGTTGAAAAAGACTTTTTTTCATACTTCATATCATAAAAAAGAAAAAGACTTACTGTCTTTAGGATCTGATGCTACACCGCTGCTCAATACCTTAGGGGATCCACTCTATTACATAAGTAGATTTCTAAGATTTATCTCATATTATGATATAAATAAACAGCTCTTGTTGTATCGGTCCAAAACCTTTCCAGTTGATCTTTACGGTGCTTCCTCTATCAATTAAATCTTTTTTTATCCATAGAAATAAAGTATTTAGGCATATCTAGTCTTCACTTCATATTTCGATCCATGAAGTTTATTTATTTGCTACAGCTGATAAAAAATCGTTTTGGACGATGCTTATGTAGAAAGCCTTTTTTTTTTTCTAGTATTTCATTGACTAGCTGTTCGTTCTTTTTTTCTATAGTGGAGATAGTCGCACGTAATGACAGATCACAGCCATATTATTAAAAGCTTGTGGTAAAAAGGGGTTTCGTTCTAATGCCCGAAAATAATATTCTAAAGCTTTGGTATGTTCCCCATTACTTGTGTGGATAAGGCCTATATTATAGAGTATATAACTTCGATCATAGGGGTCAATTTCTAGTCGCATAGCTTCATAATAATTCTGTAATGCTTCCGCATAATTTCCTTCAGATTGAGCCGACATCCGTTACGGTCGTCATTCGCTTTAACGAATTCTCCGTTTCAGAACCGTATGTGAGATTTTCATCTCATACGGCTCCTCCTTTAGGTGCATAATGAAAATAATAAATATATGGATATGGAAAAATTTGATGTCATTATGAACTAAGCGGGGCTAATGTTTTTACAAGAAATCCCTAGCCAACCTTCTTGTAAAAGATCTTTTCTTACTACCAAGTGGATTCATATTCATACTAGATAAAAATAAAAAAGGAAACTCTAACAATTTCTTTGTTCTCAACGCCCCTAAATTTCCAGGAATTAGTCACTTCAACAGTCTTCAATGGTTATACGGGTATCCAAAGTACGGACGAGATGGATGTTTATTGTTCCAACCATTTTAATTAGTCCCAATCCCAAAGAAGAAGAAAGAAAAGGAATCTTTTTGAAGAAAGTTTTCGTGTTGTTGATTTCTCGGCGTAGTGCTTCTTCCCCTGTGCCTCCTATTCGTATATTGTATTAGTCTAGTAGGATTGATCTGTAATACGGGAACCGTAGGTAAAAAACCTTTTGCTCAATACTAGAATTCATAATTGAAGCATCTAAGGCTGCACTAATCGTGGATACATGACAGAAGGGATTGCTTTTTTATATTATAAACTTCACCTTCAAAAGCGTAGATTTTTTTCAATACTCATTTTTTTCTATTCCAAATCGGTGAGAAATAGAAAAAAATGATAATGATAATCAAATCGCACCATCTCTGTAATAAGTAAATGCCTCTTTTTCTCCGGAAGTTGTCGGAATGACTCGTAATAAGATATCGGCTACAATTGTAAAGGTTTTATCAATAAAATTTCCATTTATACGCGATCTTGGCATAGGTAGTAATCCATTCTATAACTCTTTTTATTTCCTTTAACTTTTCTTTTGTGAGAAAATTTTCTCACAAACAAAGGAATTTTATAGTACGAACTAACATAAAAGCGGACTCGTTTTTTATAAAAAAATATTCTATCTACTTCCAATTTTTCTGATCAAAAAAGGTATCTATTAACCATAATCTAAAAAACGATGAATAACTCGCTATTCACCCAGGTACTCAGTCATAATCCTGATGTCGGAGAGATGGCCGAGTGGTTGAAGGCGTAACATTGGAACTGTTATGTAGACTTTTGTTTACCGAGGGTTCGAATCCCTCTCTTTCCGTACTTTCAACTAAATAACCAATCTTACGTGATTGACCACAACGTATCAAATCAAATAAAAAAAATAGATATAAAATCTACATTTCTTTGCTATGGAAAATGCTGGGAAGAGCAAACAAGGGATCCAAACCTCCCTACCAATCTATGATACATGAATAGGAAAAAGATTCCCCGACAAAATCCCTTACCTTGTCCCTTTTTAGTTTTAATCAAAAAAGCGGGCAAAGGGGAGTTGTCCGAACTCTTGTTTTTAGTGATTTTTTTTTACTTCACTTAGGTTTTTTAAGTCTGGCGAGAGTAATATTCTACGACAAGCAATTCATTTATTTTCAAACCGACGCATTTCCTATCTATTATTTGATTGACTAACCCTTCATATTGGAATGTGTGAAGAGTCAGATGGTTTGGCAATTCCTCGGGGGCAGATGACTCAAGAAGATTTTGAACCAAAGTTCTAGAGTTTTGTTCATCCTTCACTGTAATAATATCTCGGGGTTTGCATCGATAACTTGGTATATCAACTATACGACCATTAACTAAAATATGCCCATGGTTAACTAATTGGCGCGCTTGAGGAATAGTCAAAGCCATACCCAACCGAAAAAGGATGTTATCCAAACGCATTTCAAGTAATTGTAATAAAACTTGACCCGTTGACCCCTTAGCTTTTCCGGCGATACGAACATATTTAAGTAATTGGCGTTCTGTAAGACCATAATGAAAACGCAATTTTTGTTTTTCTTCTAAACGAATACGATATTGAGATTTTTTTCCGGAGCGTGATTGGTTTCTAAGATCGCTTCCTGCCCTAGGCCTTTTACTAGTTAGTCCCGGTAAAGCCCCCAGACGGCGTATTTTTTTAAAACGAGGCCCTCGGTAACGTGACATAAAGACTCCTTTTTTTATTGAAATTGTACAAAAACTAAACAAAATTAAAACTGAACTAAATGATAATGATAAATAACGTAAAATCCACTCCAATAAACTATTGGAATACAAAGAGTCAGAAGATATATTCTCTCAATATACAGATTTTTTTTATTGTATATACAATATATATAAATCAATAAATCACAAAAATTTTCCTTTATTTTCTTCATTTATTTTGCCAAGATCTAACCCTTTTACCCCAATATATATTCCTATATGGAAGTTTATATGACATAATATAAATGGCGTGGTAACTCTTGGAAAAAGGTGAAAGAAGTCTTTTCAATCTTATTTTTTTTTGAAAGTACATTAAAAATCATGTAAAAAAAATGAAAAACTATGTAAAAGCCGGCTATCGGAATCGAACCGATGACCATCGCATTACAAATGCGATGCTCTAACCTCTGAGCTAAGCGGGCTCAACTAAAATAGTGTATACAAATTCACTAAACTACTAGATCGTATTAATTAACTATTCTATTCATATTTTTCCTTATCTATTTAGAATTCATCATATTTTCGATATTCTAGAACAGAATATAGCTCAAATAAATAGTGACTATCATTAAATAAATAAAACAAAACCTTAATGAATTAATATAATATAGCAATATATCGACTTTCTAATTTTGATTTCATGAGTTTCTAAATAAGAAAATTTTAATTAGACCGGAAAGCTTTTTTTTTAAAGTTAAATGATATCTGATTTGAAATTCTTGGTTTTTTTGTTCTAACCTCATGCAATTATTATTATTTGATACTTTTTCTCTTTTTATATTCTTTATTATTTTATAGAATTAAATTATTAGAATGAATATTCGAATATTCATTTCGAATATACTTTTTTAGAATTATTCGAATTTCAAATCTACGAAGTAGACTTATAATCTTTTTACATTGCACATTGTAGAATTCTAAGTTTCAATAATGATCATAAATTTCTTTTCATGGAAGTAAAAAAAACGAATCGACCGTTCGACTATTTCTTAAAATTGAAGACAACGATGAGAAAAGGAAGAACATATATATGTTCTCTAATATATAACCATATTGAATTGCAAATACAAAAATGATAGAATCTTTGTTGATTAAACTAAATCAATATGGATGGGGCTAAAAAAAATGCAAGAAGATACCAAAGAAATAAAATAAGTATCTGTATGTAATGAATTCCAAGGTTTCGTCATAAGAAAAAGTGGAAAGACATCATAATGAGATCCTAATCTCAAAGCAAAAAGGGGGATATGGCGGAATTGGTAGACGCTACGGACTTAATTGGATTGAGCCTTGGTATGGAAACCTACTAAGTGATAACTTTCAAATTCAGAGAAACCCTGGAATTAACAATGGGCAATCCTGAGCCAAATCCTGGGTTACGCGAACAAACCAGAGTTTAGAAAGCGGGATAGGTGCAGAGACTCAATGGAAGCTGTTCTAACAAATGGAGTTCAATCCCTTGTGTTGAATCAAACGATTCACTTCATAGTCTGATAGATCCTTGGTGGAACTTATTAATCGGACGAGAATAAAGATAGAGTCCCATTCTACATGTCAATACTGACAACAATGAAATTTATAGTAAGATGAAAATCCGTTGACTTTTAAAATCGTGAGGGTTCAAGTCCCTCTATCCCCAACCCTACTCCCTAAAAAAGTCTGTTTGACACCTTACCCTTTTTTTAGTTATTCAAGAATTCATTGATCTTTTTTCATTCATCCGACACTTTTACAAACTCGAATTTCTTTTCTTATTATATACAAGTCTTGTGGGATATATCATACATATACAAATGAGAAAGAACTATCGATTTGAATTATTTCGAATCTAAATAATTTTTCATTCTAAAACTTAGAAAGTCTTCTTTTCGAAGATCCAATAAATTCCCGGTCCAAAACTTTTTTCATTTACTACTTTTGCGTTTCTTTTAATTGACATAGACCTAAGTCATCTCATAAAATGAGAATGATACTTCGGTAATGGCCGGGATAGCTCAGTTGGTAGAGCAGAGGACTGAAAATCCTCGTGTCACCAGTTCAAATCTGGTTCTTGGCATAGGGCAGAGGACTGAAAATCCTCGTGTCACCAGTTCAAATCTGGTTCTTGGCATAGGGCAGAGGACTGAAAATCCTCGTGCCACCAGTTCAAATCTGGTTCTTGGCATAGGATTGATTAATTTTGATAAGTTTATAGTCTTCAAATTAAACGTATCTTTAGTAAAAAAAGTGCAATAATCCTTTATCCCCCTCTCTTTTTTGTTCATGTTGTGGATCCATCCGTTCAAAAAAAATGTATAAGACTTTATACCTAATACATATTCGAAAGGAAAGTTCTGGTTGAAAGAATAAAAAAAAGTAAAAAAAAAAAGATCTATATCTATCTATCTATATCTATCTATAGTATCTATCGTTGAAGGGCAGAAATACCCCCAAGATTCATTAGATTAGATACAATAGAAATAGAATTTTAACCCCCCCATTTATTGTATTGCTTTCCAATCTTATTTATTCATTCCCAGTTATGTGACTAAAGTTGACTAAGTTATGTGCGCGATACAAAGTTCATAATGCAGAACTCTTTTTTTTTTTTTTAGTTCATCCTATTGGCTCGGCTTTTAGGAAAAAAGTATCTTTCAAATTGGAGATTAAGCTATCTATAATAATATGAATAAGACCTTAATTCTTCTGTTTGTTTGATCTAAAAACGACTCGAATTCAAAATATTCCGCGAAGGTCCGTAGTTGTAGAAACTAAGACTCATTTTTATCATTCAAATTTTTTATCATTCAATAAGCATCTTGTATTTCATAAAAATTGGGGGCAATATAATCCTTACGTAAAGGCCACCCTATCCAACTTTCGGGCATTAAGATCCGTTTCAGCCGCGGATGGCTATCATAAGTGATTCCTAACATATCATAAGATTCCCGTTCTTGAAAATCCGTACTTTTCCAAACCCAGAAAACAGATGGAATTCTGGGATTACTCCTGTGAGTAAATACTTTTATGCAAACTTCTTCCGCTTGATTGACACCATATTCTATTCTCGTAAGATGATACACACTGGCTAAGAGGCCACCTGGTGCCACATCATAGGCACATTGGGAACGTAAATAATTGTAACCATATACATATAAAATTACAGCAATAGAATGCCAATCTTCGGGCTTTATTTGTAAAGTCTCTATTCCTTGGTAATCGAAGCCCAACGATCTATGAACCAGCCCGCGTTTGGCTAGCCAAACGGACAAAGTGCCCTGCATCTTTTTTATTTCCCCCACACCTTTTTTATATAAATTTAAGTATTTCACATTTACCATGAGTTCTAATTTATGAAGATTTTTTTCTTATTCTCTCAAATCCTCCCTAATTCACTAATTCGTGGGAAGATACTGGGCTTTTGTATTTAAAAAATGTTTCAGTAGAGATCTCTGAAGTAGATGATGGTGGATAGAGTAATTCTTGATCATAATTTCCAGTCTGTGTACTGCGTACAACAAAAAACTTGTGATTGGTAGTAAAACACCGATTACCCCGTTGAGGTCTAATTCGATCCTTATAGATTTCTCTAGCTATTTTCTTACGAAGCTTTGTTATAGCGTCTATAACAGCCTCTGGTTTAGGTGGACAACCCGGCAAATAGACATCTACAGGAATTAGCTTATCAACCCCTCGAACAGTACTATAAGAATCGGTACTGAACATCCCCCCTGTAATTGTACACGCTCCCATAGCAATAACATACTTTGGTTCAGGCATTTGTTCATATAATCTCACTAAAGAAGGAGCCATTTTCATTGTTACTGTACCTGCTGTTAAAATAAGGTCCGCCTGTCTAGGACTTGATCTTGGTACTAGCCCATAACGATCAAAGTCAAATCGGGAGCCTATTAATGAGGCAAATTCAATAAAACAACAACTGGTACCATAAAGAAGCGGCCATAGGCTGGAAAGTCTTGACCAATTTGAAAGATCATTTAACGTAGTTGAAATAACTGAGTTTTTTGTTGTTCGATCAAGTACGGGAAACTTAATGGAATTCATAATTGTTTCAATGGTTTTTTTTTACTTTTTTTTTATTGTTATTGTACAAGTATTCAGGAAACGAACTAAGACCATTCCAACGCTCCTTTTCGCCATGCATAAACTAAACCAAGAATTAGGATAAGCACGAAAATGAAAGCTTCTATAAAAGCGGATACCCCTAGTACATCGAAACTCATTGCCCACGGATACAGAAAAACGGTTTCAACATCAAAAACAACAAAAACTAGAGCAAACATATAATAACGGATTCTAAATTGTAACCAAGCATCCCCGATCGGTTCTATACCTGATTCATAACTAGAAAGTTTCTCCGGCCCCTTCGTAATTGGAGATAAAACCCCGGAAATTAGAAATGCCAAAACAGGAATAGCACTTGATATTATTAAAAATGCCCAGAAAATATCATATTCGTAAAGCAGAAACATAGACGAACTCCTATGAATGTGGAAAAAATACCCGCTTAGTCAATTCCAATCGGAGTGGATTGGGCAAGGTATATATAACTCTTGCGTCAAAACAAAAATTCAGGTTAATCGAATCATTTATTTTCGTTTGGTTGCTGTGGTAGACGTCTCCTTTTAAGATTTATTGATTGTAATCTTATTTTCAGTACACTTATTACTTAATATTTCCATGTTTCTATTACTAATAGTTTCTCATATTAATAATATAATATTAATATGATTAATAACTAGTAATTTTTTTTATTTCTGTTTCTTAAATTTGCTTTATGTTTTATTTAAAAATAAAACAAATTGATAAAAATATCTTCGTTTTTAAAATTATGACGTATCAAAAAATCCACTTACGACTATGAAAATGAATGAATAAAAAACGTTTATTCTAAATTATAAGTATCTATCTAGATATATCTATAGATAGTGATTGGATCCACTGAAATCAAATGAAATCAAATTTGGTTTTCCGTTTTATTCTGAACGACCCCCAGGACTTATGGTTTAGGGTCTGGGAGTTTTTTTTATGAACCAACAAATTGAAAGTAACCAGTTAGAAATAAAGAATACAATAAAAAGTCAAAAATTATCCAATTATTTGGATTTGAATGTCATTTATTAGAATAAATTTATTAGTTAGGGCTATACGGATTCGAACCGTAGACCTGCTCGGTAAAAGAGCTCGAACTTATTATTATCAAAATGATTCGAACTCTTTCAAAGACCCAACATGCATTTTTTTTGCATTGGGCTCTTTCATTAACTGATAGAAAGATCAGTTAGTCTACCATATTTTTTCTTAAAAAAAAAAGATAAGAAATGGTTCCAAGTACTCTGATTGATTATTTTTTAATTCTAATACAATACAGAATAACTACCAAAGTGTTTCAAAGAAGGGTTCTCTTGACGTAGGTTTGCTTTTGGTCTAGATCAACTTAAGTTAAATATAGTCTCTAACATCCTGATTAAAAAATCAAATATGAAACTTGCTACACCTTAAGGTTCATAGGACGAAAAGATCATTTTTGAGTTCCTTATACTCATTCTGCCTAGCATTAAGTAGACTGGGTATTCACCCTATCAATATCTCAAATCAATGATGGGTTCTATTAATTCCCTACCGAAATGGGGTACTTTAATAGGACCTAATGTCAGGCTATTGTTCTCCTCTTTTTCCTAAAAAAAAGTCATGGAGTAAGACATCGATTTATTAATAAGATCAATCAATTGGTTTGATTGCGTGATGGACTCCTCTGAAAAACTTTGGCGCACGTGTAAACGAGGTGCTCTACCTAACTGAGCTATAGCCCTTGTGTTTATGATCCACATTTTATCTTATCTTATCATGTAGATAATTTCTTGTCAAGATTAATATTATATGATCGAACATTATATCTCTTTCATCTCGTTGTTTATTGGTATTGCTTAGAAATAATATTGGATTTATAATCCTATCGATGTGATAAGTATCCCCGTGCCTTCTCTTTACGATGATAAATAACCTACTTAACTCAGTGGTTAGAGTATTGCTTTCATACGGCAGGAGTCATTGGTTCAAATCCAATAGTAGGTATAACTTATTAGACACCATGATCAATGGTGTCTAATAAGTTTTTGTAGCCAGCTTTTTTTTTTTTTTTTCTCGCTTTTGGATCCTATTTTTTTATACGTCAGCTAGTTACAAAATCAAATCGTATTGAGAGCCTCGACGCGTGTCCGAGCTCGTCTGAGAGCTAGATTAGCCTCAATTGTTTGTCTCTTGCCTTCAGCTTTTCTCAAGTTCGCCTCTGCTATTTCAAGAGTTTGCTGAGCTTCTTGTGGATCAATGTCACTATTCTTCTCTGCATCATTTACTAAAATAGTAATTTCATTATTGCCTATTCTAGCAAAACCGCCCATCAGAGCCATTGTTAACCATTGGTTATTAAGGCGTATTTTCAAAATACCTATATCAACAGCTGTGGCAATCGGCGCGTGATTTGGTAATACGCCAATTTGTCCACTATTAGTAGATAAAATGATTTCTTTTACTTCTGAATCCCAAACAATTCGATTCGGAGTCAGTACACAAAGATTTAAGGTCATTTCTTCAATTTACTCTCCATTTCTAAGTTCGTAGCCTTCGCAGTAGCTTCATCGATGTTACCCACTAAGTAAAAGGCCTGTTCAGGAAGAGAATCAAATTCTCCGGAAAGGATCAAATTAAACCCTCTAATTGTTTCCGCTAGCCCAACATATTTTCCCGGAGAACCTGTAAATACTTCTGCTACGAAAAAAGGTTGTGATAAGAAACGCTCAATCTTTCGTGCTCTTGCGACGGTTAAGCGATCCTCTTCGGATAATTCGTCCAACCCCAGGATAGCTATAATGTCCTGAAGCTCCTTGTAACGTTGTAAAGTTTGCTTTACTTGTTGCGCAGTTTCATAATGTTCCTCGCCAACGATTCGAGGTTGTAGCATAGTTGACGTTGAATCTAAAGGATCTACCGCTGGATAGATACCTTTAGCAGCTAATCCTCTTGATAGTACGGTAGTCGCATCTAAATGTGCAAATGTGGTGGCAGGAGCAGGGTCAGTCAAATCGTCTGCAGGTACATAAACTGCTTGAATAGAGGTTATGGACCCTTTTTTCGTAGAAGTAATTCTTTCTTGTAAAGAACCCATTTCGGTACTAAGGGTGGGTTGGTAACCCACAGCAGAAGGCATTCTACCCAATAAAGCGGATACCTCGGATCCTGCTTGTACAAAACGGAAGATATTGTCGATAAATAGAAGTACGTCTTGCTCATTAACATCTCGGAAATATTCTGCCATAGTTAAGGCAGTCAGACCAACTCTCATACGAGCTCCCGGCGGTTCATTCATCTGACCGTAGACTAGGGCTACTTTGGAGTCCGCAAGGTTTAGTTCATTAATGACTCCAGATTCTTTCATTTCCATGTAAAGATCATTTCCTTCACGAGTTCGTTCGCCTACTCCACCAAATACGGATACACCACCATGAGCTTTGGCAATGTTGTTGATCAATTCCATAATTAGTACTGTTTTACCCACGCCAGCCCCACCGAATAGTCCGATTTTTCCCCCACGACGATAAGGGGCCAAAAGATCTACTACTTTAATTCCTGTTTCAAAAATAGATAAGGTTGTATCTAAGTCTATAAAAGCAGGCGCGGATTTATGGATAGGAGATGTTGTGAGAGTATCGACAGGACCTAAATTATCAACAGGTTCCCCAAGTACATTGAAAATTCGTCCTAGAGTCGCTCCGCCGACTGGAACACTTAGAGGATTTCCCATATCAACCACGTCCATCCCTCTCTTTAAACCCTCGGTCGCGCTCATAGCTACAGCTCTAACTCGGTTGTTTCCTAATAATTGCTGTACTTCACAAGTCACATTAATTTCTTGACCAAGCGTATCTCGACCCTTAACCACCAGAGCATTGTAAATATTAGGCATCTTGCCCGGGGGAAAGGCTACATCCAGTACCGGACCAATGATTTGGGCAATACGTCCCAGGTTGTTTTTTTCACGTATTGAAACCGCTGGATCCGAAGTAGTAGGATTTATTCTCATAATAAAAAATATGTTCAATTTTGTTGCGAAATTTTTCGAATACAGAAAAAATCTTCGATAGTAAATTCATTGGTTAATTCAATAATAAATGGGAGTAAGCACTCGATTTCATTGGTACCACCCAAGCGAATATGCAATTCAATTTTTTACTTAATTAAATTTCAATGAAGGAATAGTCGTTTTCAAGCTCAACTAACCAAAACCTAGTTTTAAAATAAAAAATATATGAATAAAAAAAATTTTTGTGGAAAGTCTTTGATTTATTTGTCATAATAGGCAAGACTTTGTTTTATCTAGCCAATTCCGAAATGGAACTCTATTTATGATTCATTATTTCGATCTCATTAGCCTTTTTTTTTTCATATTTTCATTTTAGCATATCCGGTTATGCGTCCCATCGATATCAACCCCCCCTTGTTTTTCATTTTCATGGATGAATTCCGCATATTGTCATATCTAGGATTTACATATACAACATATATTACTGTCAAGAGTGATTTTATTATTATTTTAATATTAAATATTTCGATTTATAAAAAGTCAAAGATTCAAAACTGGAAAAACAAGTATTAGGTTGCGCTATACATATGAAAGAATATACAATAATGATGTATTTGGCGAATCAAATATCATGGTCTAATAAAGAATCATTCTGATTAGTTGATAATTTTGTGAAAGATTCCTGTGAAAAAGGTTAATTAAATCTATTCCTAATTTATGTCGAGTAGACCTTGTTGTTTTGTTTTATTGCAAGAATTCTAAATTCATGACTTGTAGGGAGGGACTTATGTCACCACAAACAGAGACTAAAGCAAGTGTTGGATTCAAAGCTGGTGTTAAAGAGTATAAATTGAATTATTATACTCCTGAATATGAAACCAAGGATACTGATATCTTGGCAGCATTCCGAGTAACTCCTCAACCCGGAGTTCCACCTGAAGAAGCAGGGGCTGCGGTAGCTGCTGAATCTTCTACTGGTACATGGACAACTGTGTGGACCGATGGGCTTACCAGCCTTGACCGTTACAAAGGACGATGCTACCACATCGAGCCCGTTCCAGGAGAAGAAACTCAATTTATTGCGTATGTAGCTTACCCATTAGACCTTTTTGAAGAAGGGTCTGTTACTAACATGTTTACCTCAATTGTGGGTAACGTATTTGGGTTCAAAGCCCTGGCTGCTCTACGTCTAGAGGATCTGCGAATCCCTCCGGCTTATACTAAAACTTTCCAGGGACCACCTCATGGTATCCAAGTTGAAAGAGATAAATTGAACAAGTATGGACGTCCCCTATTAGGATGTACTATTAAACCTAAGTTGGGGTTATCCGCGAAGAACTATGGTAGAGCAGTTTATGAATGTCTACGTGGTGGACTTGATTTTACCAAAGATGATGAGAATGTGAACTCTCAACCATTTATGCGTTGGAGAGACCGTTTCTTATTTTGTGCCGAAGCTATTTATAAATCACAGGCTGAAACAGGTGAAATCAAAGGACATTATTTGAATGCTACTGCGGGTACATGCGAAGAAATGATGAAAAGAGCTATATTTGCCAGAGAATTGGGAGTTCCTATCGTAATGCATGACTACTTAACAGGGGGATTCACCGCAAATACTAGTTTGGCTCATTATTGCCGAGATAATGGCCTACTTCTTCACATCCACCGTGCAATGCACGCTGTTATTGATAGACAGAAGAATCATGGTATGCACTTCCGTGTACTAGCTAAAGCTTTACGTCTATCGGGTGGAGATCATGTTCACGCGGGTACAGTAGTAGGTAAACTTGAAGGAGACAGGGAGTCAACTTTGGGCTTTGTTGATTTACTGCGCGATGATTATGTTGAAAAAGACCGAAGTCGTGGTATCTTTTTCACTCAAGATTGGGTCTCACTACCAGGTGTTCTACCTGTGGCTTCAGGGGGTATTCACGTTTGGCATATGCCTGCTTTGACCGAGATCTTTGGAGATGATTCCGTACTACAATTTGGTGGCGGAACTTTAGGCCACCCTTGGGGAAATGCACCGGGTGCCGTAGCTAACCGAGTAGCTCTAGAAGCATGTGTACAAGCTCGTAATGAGGGACGTGATCTTGCAGTCGAGGGTAATGAAATTATCCGTGAGGCTTGCAAATGGAGTCCTGAACTAGCTGCTGCTTGTGAAGTATGGAAGGAGATCACATTTAACTTCCCAACCATCGATAAATTAGATGGCCAAGACTAGAAATTAGATTAGTAATTCACGTCCGTTTTATTAGTTTAATTGCAATTAAACTCGGCTCAATCCTTTTAGTAAAAAAAAGATTGAGCCGAGTTTATCTAGTGTATATACTGTTTTTGATAGATACATACTTAATCTAGATATACAAAATCTGAAAAAAAAAGAAGATTAAACACAACTACACTTTTGTATTGTAGTGTCCACAAGAAATTCTATACGAAATATGGATTCTTAGGATTTTTTTATTCTTTTTTTAAGTTTCGTGTCAGGGCTTGAACCAAGTATCCCCACTTCTTCTACCCATTCTGCATGTTGTCCTTTTCTTTTCATTCCGTATTGGAATAAAAACTTTTTTTTTATATTAGTATACGAGATTTTACTAAAAAAGTTCTTCATATCGCTTATATTCATAAGCGAAGAACAAATATTTCTTTTTTTTAATGAGAATTTTACACAATATAAGAAAATCCTTATTTTCATTTAGAATTGAAATTTATTAATTTCAATTGCTTTTACTTAATAATCTTAGCAATTAGCAATTGCATTGACATGCTTTGCTTACTCTGAATAGAAAATGAACTATTCAAATTTTTTTTTTGCATTTTTCAATTTTTTCATTGAATGACTATTCATCTATTGTTATTTTATTTTCATGTAAATAGAGGCCAGAAGCTCTATGGAAAAATCGTGGTTCAATTTGATGTTTTCTAAGGGAGAATTGGAATACAGAGGCGAGCTAAGTAAAGCAATGGATAGTTTTGCTCCTATTGAAAAGACTACTATAAGTAAAGACCGGTTTATATATGATATGGATAAAAACTTTTATGGTTGGGGTGAGCGTTCTAGTTATTACAATAATGTTGATCTTTTAGTTAACTCCAAGGACATTCGGAATTTCATATCGGATGACACCTTTTTTGTTAGGGATAGTAATAAAAATAGTTATTCTATATATTTTGATATAAAAAAGAAAAAATTTGAGATTAACAATGATTTGAGTGACCTAGAAATTTTTTTTTATAGTTATTGTAGTTCTAGTTATCTGAATAATAGATCTAAAGGTGACAACGATCTGCACTATGATCCTTACATTAAGGATACTAAATATAATTGTAATAATCACATTAATAGTTGCATTGACTCTTATTTTCGTTCTCACATCTGTATTAATAGTCACTTTTTAAGCGATAGTAATAATTCCAATGAAAGTTACATTTATAATTTCATTTGTAGTGAAAGTGGAAGTGGAAAGATTCGTGAAAGCAAAAATGACAAGATAAGAACTAATAGTAATCGTAATAATTTAATGAGTTCTAAGGATTTCGATATAACTAAAAACTACAATCAATTGTGGATTCAATGCGACAATTGTTATGGATTAATGTATAAGAAAGTCGAAATGAATGTTTGTGAAGAATGTGGACATTATTTGAAAATGACCAGTTCAGAGAGAATTGAGCTTTCGATTGATCCGGGTACTTGGAATCCTATGGATGAAGACATGGTCTCTGCGGATCCCATTAAATTTCATTCGAGGGAGGAACCTTATAAAAAGCGTATTGCCTCTGCTCAAAAAAAGACAGGGTTGACTGACGCTATTCAAACAGGTACAGGTCAATTAAACGGTATTCCGGTAGCTCTTGGGGTTATGGATTTTCAGTTTATGGGGGGTAGTATGGGATCCGTAGTAGGCGAAAAAATAACTCGTTTGATCGAGTATGCTACCAATCAATGTTTACCTCTTATTTTAGTGTGTTCTTCCGGAGGAGCACGAATGCAAGAAGGAAGTTTAAGTTTGATGCAAATGGCTAAAATTTCTTCGGTTTTATGTGATTATCAATCAAGTAAAAAGTTATTCTATATATCAATTCTTACATCTCCTACTACCGGTGGGGTGACAGCAAGTTTTGGTATGTTGGGGGATATCATTATTGCCGAACCCTATGCCTATATTGCATTTGCGGGTAAAAGAGTAATTGAACAAACATTGAAAAAAGCCGTGCCTGAAGGTTCACAAGCAGCTGAATCTTTATTACGTAAGGGCTTATTGGATGCAATTGTACCACGTAATCCTTTAAAAGGTGTTGTGAGTGAGTTATTTCAGCTCCATGCTTTTTTTCCTTTGAACAAAAATGAAATCAAATAAAACAGTTAGTTTATCATAATTAAACGAAAACCCTGAAAAATTCATTTTTCTTTAGAATCATTTTTTTATCGATATTCTTGTTTACTACTCAGTAAACCTTTATCAACAAGATAAAAAGTGAATTTTTGCTTTCGGGAAGTTCAAATTCGACTAGAAAAATAAAACAAAGTTTTTTTCCTCTCTTGCTTGCATATGGATAGATAATTCAAATAGAGATATAGATCTATAGAGAGTCTTGCATCGTTTTGCATTTCCCGAAAATTCCCTGTTGGTGGATCAGATTCCAATCAATTTTGTATAAAATTTTAATGGAATAAAATTTTTTCTTTATTAATGACTATTAGAAGACAAAAAGAACAAAAAGAATAATAAATCTAACAGGGAGATTATGATAATACATCTATTTTATTTTGAAAGATTAATAAGTCCATTTATTTAGTTTGGCATTTCTTGTACCTATTTTTTTATTCTATTTCTAGTAGGTTCTATTATTTCTATTAGGTTGTATATTAGTATTCGATATATATTTACTTAAAGATACTTAGTATAATTATATAATATATATAATAGAAATAATAAAACTACAAGATATTCTAAGATATCTTTAGAATTCAGAATATAACAATAACAGGTACAAATATTAAATTGAGGTACCCCATTTTATGACAACTTTCAACAACTTACCCTCTATTTTTGTGCCTTTAGTAGGCCTAGTCTTTCCGGCACTTGCAATGGCTTCTTTATTTCTTCATATTCAAAAAAATAAGATTTTTTAGATCGGATGAGACCGAATCGTATAACTCCCCTTTTTATTTTAAAAACTTCGATTTGATAAGACCCATTTGGTAGAATATTGTATAACACATAGATTCCTACAAACATAACTAAAAAAAGTTTTTATGCATGTGTAAACGTATTATATGGGGTAACTCAATTTGCGCTCTTTTGAAAAAATGGATCATCGTCGGACCGCTGGATGAAATTCAAGTCAATGTATTTATTTGTATGTATATAGTTATAGGGGATCATATAAAGGAAGGAGATTTTATTATTTTAGATATAAACAATTATATAAATTATTCCTAAAGTAAAGGTTCACAACAAAATAGTTATAGTTGATGAGAGTTACTTTGAAAACAAAAAAAGGAAAGTCATATTTTCTCAATTCCAAAAAATTGTATAACTGGATCTAATATATATGAGTTGGCGATCAGAATCTCTATGGATAGAATTTATAACGGGGTCTCGAAAAACAAGTAATTTCTGCTGGGCCTTTATCCTATTTTTAGGTTCATTGGGATTCTTATTGGTTGGAACTTCCAGTTATCTTGGTAAAAATTTTATATCGTTAGTTGCATCTCAGGAAATCCTTTTTTTTCCACAAGGGATTGTGATGTCTTTCTATGGGATCGCGGGTCTCTTTATTAGTTGCTATTTGTGGTGCACTATTTTGTGGAATGTGGGTAGTGGTTATGATCTTTTCGACCGAAAAGAAGGGATAGTACGGATTTTTCGTTGGGGATTTCCTGGAAAAAGCCGTCGCATCTTTTTACGATTCCTTATGAAAGATATTCAGTCGATCAGAATCGAAGTTAAAGAGGGTGTTTCTGCCCGGCGTGTCCTTTATATGGAAATTAGAGGTCAAGGGGCTATTCCTTTAATTCGTACTGATGAGAATTTTACTACACGAGAAATTGAGCAAAAAGCTGCTGAATTGGCTTACTTCTTGCGTGTACCAATTGAAGTATTTTGAAATGAATTCATTTTTAAAGTTTAAAGACTAAATCCTTTGGCAGTAGGAAGAAAAAACGAAAGAATTGCTTTCTTTTTTTTCAATTGAACATTCATCTATTCTTTTATGCTCGTTTTTTTTTATATATTTGATAGAAAAGAAAGGGAGTTTATTCGTCTCGAAAATAGAATCATATTTTTTATTTTAAAAATTCAAAAAAGTTCTTTTAGTATTGATCGAAAAAAGGGGGAATAACATCCTGGAAATACAATTTTTTCTTTATTCAAATTGTAAGTGTATTCTGAGTCTATTTCTGTATTCTTTCTAGATTCAAAGCAAAGACTAAGTATTGAATCAAAAGAAAAAGAGAAAAGGGATTATAGGCTCAATACATTCTATTTGAATTAGAATAGAAACTCATGCTCGATAGAAATAGTAGATCTAATAGAATCCACAAATGCGGTAGGTTCATTAACAATTCACAGATTCAAAATGGCAAAAAAGAAAGCATTCATTCCTTTTTTTTATTTTACATCTATAGTCTTTTTGCCCTGGTTGATCTCTCTCTGCTGTAATAAAAGTTTGAAAACTTGGATTACTAATTGGTGGAATACTAGACAATGCGAAACTTTCTTGAATGATATTCAAGAAAAAAGTGTTCTAGAAAAATTCATACAATTAGAGGAACTATTCCAGCTGGATGAAATGATAAAGGAATACCCAGAAACCGATTTACAACAATTTCGTCTAGGAATCCACAAAGAAACGATCCAATTCATCAAAATACACAATGAGTATCGTATCCATACAATCTTGCACTTCTCGACAAATCTAATATCTTTCGTTATTCTAAGTGGTTATTCCTTTTGGGGTAAGGAAAAGCTTTTTATTCTCAATTCTTGGGTTCAAGAATTCCTATATAATTTAAGTGATACAATTAAAGCTTTTTCGATTCTTTTATTAACTGATTTATGTATCGGATTCCATTCGCCTCACGGTTGGGAACTAATGATTGGTTATATTTACAAAGATTTTGGGTTTGCTCATTATGAGCAAATTTTATCTGGTCTAGTTTCTACCTTTCCAGTCATTCTTGATACAATTTTTAAATATTGGATCTTTCGTTATTTAAATCGTGTATCTCCGTCACTTGTAGTGATTTATCATGCAATAAATGACTAAAAAACGATTCACTGATCCAATTCTACTCTTTCTTACTTTATACATCATAACCAAATCAAAGTCGTATTTACTTTACTCTTTTTTACCCACGAGGGATTCCTTGTATATTTCAAAAAAATTTATTTTTTCAGTAAATGTAAATAGCAGAATTGTGGCTAGGGAAGTATATTATCGACCTACCTAACTTTATTGTAGAAATTTTCGGGATAAACGATTGGACCATGCAAACTAGAAATACCTTTTCTTGGATAAGGGAAGAGATTACTCGCTCCATATCTGTCTCACTCATGATATATATAATAACTTGGGCATCCATTTCAAGTGCATATCCAATTTTTGCCCAGCAGAATTATGAAAATCCACGAGAGGCAACTGGGCGTATTGTATGTGCCAATTGCCATTTAGCTAGTAAGCCCGTGGATATTGAGGTTCCACAAGCGGTACTTCCTGATACTGTATTTGAAGCAGTTGTTAAAATTCCTTATGATATGCAGCTAAAACAAGTTCTAGCTAATGGTAAAAAAGGAGCTTTGAATGTGGGAGCTGTTCTTATTTTACCGGAGGGGTTTGAATTAGCCCCCCCCGATCGTATTTCACCCGAGATGAAAGAAAAGATAGGAAATCTGTCTTTTCAGAATTATCGCCCCAATAAAAAAAATATTCTTGTGATAGGTCCTGTTCCTGGTCAAAAATATAGTGAAATAACCTTTCCTATTCTTGCCCCAGACCCTGCTACTAATAAAGATGTTCACTTCTTAAAATATCCTATATACGTAGGTGGAAATAGGGGAAGGGGTCAGATTTATCCTGATGGTAGCAAAAGTAACAATACAGTTTATAATGCTACGGCAGGAGGGATAATAAGTAAAATTTTACGAAAAGAAAAAGGGGGATACGAAATAACCATAGTGGATGCATCGAATGAACGCCAAGTAATTGATATTATCCCTCGAGGCCTAGAACTTCTTGTTTCAGAGGGCGAATCCATTAAACTCGATCAACCATTAACAAGCAATCCTAATGTGGGTGGGTTTGGTCAGGGGGATGCGGAAATAGTACTTCAAGATCCATTACGTGTCCAAGGCCTTTTGTTCTTCTTAGGATCTGTTGTTTTGGCACAAATCTTTTTGGTTCTTAAAAAGAAACAGTTTGAGAAGGTTCAATTATCCGAAATGAATTTTTAGATCTGTCTATTTCGCCTTATCAAATTCGTAAAAAAGAAAGAACAAAAAAAATTATCAAAAGCCTTTTTGCCTCTCTTTAGACTTTCGATTTCGACCAGGTGTCAGGAATTACTTGTCTGATAGTCCTAATCCTAGTATGTATATTAAGAAGAATTCACTTTACCCCCCTTTTCTTTATTTTTCAATACAAATTTGTATTGAAAAATGGGAGGGGGTGTGATGTAACTCTGTCGTTAGTGACCAATTGAAATTGATAGAATGTATCAATAATCAAGAGTTTTTTTCTAATTGAAATTGATAGAATGTATCAATAATCAAGAGTTTTTTTCTAATGTAATTTAGAATGGAAAAATTTGACTAGATACTAAAATAAGGAAAGCAAGCGTAGAAAAAAGGGAACTAGAAATTGGCGAAATAACAAATTTTAGGGACTATAGGGAGTATTACTTGTCTTGCGAGTCTTCGACACAAGAAAAGGAATTTTAGACATCCTTTTCTTGTGTCGATCTTGTCATTCTTAATTGCATTCGTTAAAAAATCCTATTCTTAGTTTACATATATATTACTGTTTCTATATATATAAGGTTTTAATATATCTATATTAATATATATCGTATATATAATTATTAATTAATAGTATTAATAATAGTTACTTTTTGTAGTTTTATTTATTTTCATTTCAAGTTTGATGAAATACTAAAAAAATAAGAAAAAACTTCTATTAGTATAGAGTATAGACAAAATTTTAATGATAGATCTAATGATAAAAAATATTGTGTCAGTCGGGAAAGCAGAAAAATAAAATTAAGTGACCCCCCCCCCTTTTTTTCGATCTTTGAAAGGTTAATAATAAATACTATATGCTCCTAACCTACTAATCTAATTAAGTTCATTTTTCAAAAACACGATAAAAATTGTTCTGATTATTAGCAGTTCAACGGGACCCCCTCGAATCAGACAAAGAAGGAAGAGTTGGGCCCCGTTGAGTTCTTATGTTTTCACGTCTATAACTCAGTTCATCCAATTTCTACAGGGATGAACCTAATCCTGAATATGAACCATAAAAGAAAATACCTATTAAACCGATCACAAGAATACCAGCTACAGTACCTATTACCCAAAGAGGAATCCTTCCAGTAGTATCAGCCATTTATCCTGCTTCCCTCCACATTTCATCGAGTGGTCATGCTAGAAACATAAACAGTCAGAGATAATTATGATATATAATCCATCCGAATGGGATAAGAAAACGAATGGGATAAGAAAATTACTACTCTTTGTTTTTTATTTTATTATTCTACGCTCTTTTCTTAATTTTACTTAATTTTAATTGAAGAAATAATTTGAAAATAAAACAGCAAGTACAAAAATGAGTAATAACCCCCAATAGAGACTGGTACGATTTAATTCAACATTTTGTTCGTTCGGATTTGATTGTGTCATAGCTCTATAAATAATTGAATTCGGTTTATCGTTGGATGAACTGCATTGCTGATATTGACCCCAAAAAAGAAACGGTAGGTACAGCTAGTCCATGAACAGCCAACCAGCGCACTGTAAAAATTGGATAGGTCCTATCTATAGTCATTGGGTCCTCCTAAAAAGATCTACTAAATTCGTCGAGTTGTTCCAAAGAATCAAAACGGCCTGTTATTAATGGAATGCCCTGTCGGCTCTCTGTAAAATACTCGTTTGGACGAGGGCTCCCAAACACATCGTAAGCTAAACCGGTGCTGACGAATAACCAGCCCGCAATGAATAGGGAAGGTATAGTAATGCTATGAATGACCCAGTATCGAATACTGGTAATAATATCAGCAAAAGAACGTTCTCCTGTGCTTCCAGACATACTGAACTCCAGATATTCTTGTAGGGAATCGATTCTGTAAAAGATGAATCAGTAAATTCAAATTCACTGAGATTACATCTTTGTGAGATCGTCAATAAAGTACCAAGGGTATTTTTAGAGTCTACCGAATCAGTATAGCTATCCTTCTTCTGACACAGCAACGCAATTTGAATTAGTATAGAACTGAAGTGTTAGATAATTTATTTCGTTTTTTTTTTTGCTTGTCGATGTATAACCATGTTCCACTTCTTCAAATTCCTGTATCTGTAATCTATAGGGGCTTTGATCCTTTATTTGTTTTGGACTAGAAAATAAACTAAAGATTAGATAAAATGAAAATTCAAGAGGGTGGTTTCTAATTCTAATAATTCATTAAGGAAATTCTCATATTGTCCCAAGTCAATTGAATCCAAAACCCATCAATTTCATTTTTGTTGCATATGCATAATTGTAGAAGATATTTTTTTGTAATGATAATGTGACCCCCCCCCGTTTTTGCTTTTTATTTCATTTTATTTTAAGGAATTAATTTCCAGTAACAAGAAAAGGAAGAATAGTTTTGGATCAATTCAAAAGAACAACAAATAATAAAAAAATAATAATACTAAATCTTTGGAATGCGTGCATTGTTTTGTTGTATTCAATTTAAAGGTTTTTTCTTTCTTTAACTAACTACAAAGATGATGGGTTTTTCACTCTATTTTCTATATAATATCGAAAGAAAAAAACCTAAAACCTAGAAAGGAAACGATAATAGAAATTGCTAATTACAAGTTTTAAAAATCTAGTTAAAATAAATAACTCTTTTTTTGACTGATCTCGTTCTCCGTGTAGGATACTGCTTTTTGGTTTAATTTCATAGATTAAATGAAGAAAACTGCTCTACTATATTAATCTACTTTATTCAAAATTGAATTTATTTCAATTTGAATAAATGTAGAAGGGGGCATATTCTAGGTTCTAAGGTCACTTAAAAAAAAAAGAATCAAACAACTTTTTTAAAAATTTTTACATGTTTATTCAAAAAAAGTTCTATGTTTTAACTGAAGTTATATAATAGAGTTATTTTTTTATGTATTATTTTTTCTTATTAATTTCTTAAAGAGTTTTTCAATGAATCAGTTACGTGAATTCTGAATCCTGAGATGGTGCAGATGCCAAAGACGATGAATTTCGTTTTTTCTTTTTCTCTATTTTTGTTCATACCACCGATAATGCTTGATAACTCACAAATTTTCAATTTAATTTTTTGATTCTTGGAACTAGTATTTGTATCTATCTTTACTTTAAAAAATTTTTTTATTGAAACTTCGGGAAGTACTTTAGAAACATATGTATAAAAAAACATATTTTATTGAGTCCCTTCATGCCTACTATAACTAGTTATTTCGGTTTTCTACTAGCAGCTTTAACTATAACCTCAGTTCTATTTATTGGTCTAAGCAAAATACGACTTATTTGAAATTAATTGAATGAATCTTTTTTGATCAAAAAAGATTTATATGGTATTTCATATGTTCGATAGTTCCTTACCGTGTTAATTACCCAATTTTGGTCATTGAGATTCGTCGGCAATACAGATTAAGAGCTAGGAATAGATAGTACCTCTCTTTTCTCCCTTTCAAAAATGAAAACAAAATAAAATTGAAATGATTGAAGTTTCTTTATTTGGAATCGTCTTAGGTCTAATTCCTATTACTTTGGCTGGATTATTCGTAACTGCTTATTTACAATACAGACGTGGTGATCAGTTGGACTTTTGATTAATTAACATCTCTTTTTTTTGACTGACCTCCTTCTTGCTTTCATATGCGGGAGGTCGAATTCAGATTGCTGCTCAATTATTTGCGAACAGTGGAATTTTGACACAATCTAATAAACAAGAGTGACATCACGCTCTGTAGGATTTGAACCTACGACATTGGGTTTTGGAGACCCACGTTCTACCGAACTGAACTAAGAGCGCTTTTCTTGTTTTTTATAAAAAAACGAAAAGGCTAGAAAGAGGACATTCTTTAACTCGAATCGATTTTGTACGTATATACTATATCATAGTATATCATAAAATTCAGAATTATATGTATGTCCAATTTTATTAAAAAAAGATAAATCTAAAATGGATTCCTCGTTACTGCTCTTCTGAGCAGTAATTAGGTAGGGATGACAGGATTTGAACCCGTGACATTTTGTACCCAAAACAAACGCGCTACCAAGCTGCGCTACATCCCTTTCGATTGGTTTACAGTGTCATTGTAAACAATTCCTATCTTGTTTTCCACATCCTTCTTTTTTTTTTGTTTCATATCAGATAACAAACATATATATAAGTATAATTAAAAAAATTACTTTTTTTAGGCAAATCCTATCAATTTCAAATTTACATAAAAAGGCGTTTCCATTTGAAAATGGAATCTATAAGATCGTTCTAGTAGACAATATTTCAATTCTAATTTTGAAAATGGGGGGTTACATATACAAATACAAGAACTTCTTAACTACATGTACATCTATAGTTATATATATTACTATATATATTGTAATACAATAAAGAAGAAAGAAGGAGGATTTCAAATGCGAGATCTAAAAACATATCTTTCCGTAGCACCGGTACTAAGTACTCTATGGTTCGTTTCGTTAGCAGGTTTATTAATAGAGATTAATCGTTTATTTCCAGATGCATTAACATTTCCCTTTTTTTAATTCTAGTTATTAACATCAGAAAGGATAAAAAAATTTAGAGATACGATCAACGATCGGGGAATAACCCCCCCTTTTTTTTCTAATTCTTTTTTTAGAATAAATTTTTTTAGAAAAAAAAGAATTAGAAAAAAAAAAGGGGGGCCGAAAGGTCATAAAAACGAGGGTTCAGGATCCAATTAAAAAAAAAGTGTTGCTAGGGAAAGAGTATCCTACGAGATACTTAAAAAAAATACTGTACAAAGATTTGAAATATAGTTTTCAAAAAATCATTATATTACTTATTATTTTCTTTTTATTTAATTACTAATTAATATTCATTGCAACGAAATATTTAAGACATTTTTTTGAGTTAATTAACAGCTTCTATTTTTTTTGTTCTTGTTCTTTATGGACCCTAAAATTAAAATAGAAGATTGGGGGTGAATCATAAATCCAAAGGAGGTTTCATGGCCAAAGGTAAAGATGTTCGAGTAACAATTATTTTGGAATGTACCAGTTGTGTTCGAAATGATATTAAGAAAGAATCGGCTGGAATTTCCAGATATATTACTCAAAAGAATCGGCATAACACTCCTAGTCGATTGGAATTGAGAAAATTCTGTCCCTATTGTTATAAACATACAATTCATGGGGAAATTAAGAAATAGATAAAATTGAGTGCTTGTATGTCAAATTTTATTTTAAGAACAGGAATAATGAGAGTATCTACGTATTATTACATATATATAAATATAAACAAATAAAATAATAGAAAGAAATCAAATCCTATATTCTTAATTCTATATAGAAACTCTATCCTATATAGAAATAGCAATCGTTTTTATTTTGATCCGATCAAAAATAGGATTTTATAGGTAAGGAATAAAAAATTATGAATAAATCTAAGCGACCTTTTACTAAATCCAAGCGATCTTTTCGTCGGCGTTTGCCCCCGATCCAATCGGGGGATCGAATTGATTATAGAAACATGAGTTTAATTAGTCGATTTATTAGTGAACAAGGAAAAATATTATCTAGACGGGTGAATAGAGTAACTTTAAAACAACAACGATTAATTACTATTGCTATAAAACAAGCTCGTATTTTATCTTTGTTACCTTTTCTTAATAATCAGAAACAATTTGAAAGAAGTGAGTCGACCCCTAGAACTACTAGCCTTAGAACCAGAAAAAAATAGACTTATTCTTCAATTGAATAACTAATCTGAAGGAATTAAAAAAGAGGTTAATATTTTGTTCGACAAATCCAATCAAGAATCAAAATTTGATTGTTACGTCTGTTTCTGTCATAAAAAAAAAAAAAAAAGAAAAGAATCGTCGAAAAGAAAAAGAATAAGTCTTTTTTTAGCGACTATATACCCTCGTTTTGTTTTGACGACTTTTTTTATAATACTAATTTCTACTCTACCCTCCCCGAGCTTATTCTACTTAAGAACTCTATTTCAAATATTTTAGTGGATTTCTTCCAATCCCCTCATTTTTTGATCTCATTTGAAATCGTATAAAGACAACTCCTATTTAATAGAGCTATTTGTGCAAGTATTTTTCGATTAAGAAGTAATTGCTTCTTGTACAGATTGTGTATGAATCGGTTATAACTATAGAATACCTCCGTTTCGTGAATTACGGCATTTATTCGAGTGATCCATAAACGACGAAAATCTCTTTTTCTTTTACCCCTATCCCGACGAGCCGAAACTAAAGCTCTTATTCTCTGTTGAGTCATAGTTCGTGTAAGTCGTGAATGAGCCCCTTGAAAGCTTGATGCAAATAAACGAAGTTTTGTTCTACGCCTCCGAGCTATATATCCGCGTTTAATTCTAGTCATTGAATAAATCAAACTTTGATGAATAACTAATTCTTTTTTTAGTTTTAGTTATTCTTTTCCCCTTTACTAGTCATTAATAACCAAACGAATTATTCCAATGTATAAAAAAAAATTCCAATGGCTTTTGCTACTCTAACCTTCCCCACCACTATTTTTGGCTAGGTATTTTCCTTTGCTTTGAAAGGATAAATTGCCTTGGTACTTGATATAAAAAAAATAGAATAACTACAAAAAGTAGTAAATAGAAATGGATAAATAGTGGGTTCCTTCGTTTCTATGGTTACTTCTAAAACGGTGAGGTCCTCTCTATACACCGGAGCTCCTTCTTTTAATTAATCAATACTATTGGTAACTTGTACAATTCACATTCTTTGGCTCTACCCCATTAATATTCCAGTAATAGATCTTTCACAATGAGATCCACTTTATACAGTAACGGTATTTTATTTTAAAATTGATTTGGTCATTTACCCTGTTAGTCCGTTTTTTCTTTCAAGAGTGGAATCTTTTTAATAAAAAATGGGATTTCCCCCGCTTAATTGATAACCATTTGTTATCATTGGGGGTTTTCTAAAAAATGGAGTTGATTGGATTTGCACCAATGTAAACCATAAGTTTCAGACACAATAGAAGATATGAATGATCTATCTTTTTGAAATAATGAATCGAGTTCCTCCATTCTATTTTATTAACAGGTACTGATCCTTGATATTTCAAAAAGAATTTCCTTTTTGTGTTTCAGTCTATGATCTAAACGAGTCGCACATACACCCGAGTACATGTTCCTCGTCGCTGAGGGCATCCCCGAAGCGCTGGGGATTTTGTGACATTTCGGATTGGCTGTCTTGTATTTCTAATAAGTTGTTTAATGGTTGGCATACGGAATCATATAAATAATGGGCTGGTTTAGATGGGTTCTAACCGGCTAATTCTGAATTACTTCTCTTCAAGATTCTCTTCAATATAAAAAAAATATATTGAAGAGAATATGAAACTAAACCTTTAATCTAAAAAGATATAAAATTAGCAGTGGTAGATTTGCATGAAATCGCTCCTATTTTTATTGAACCGCTACAAGATCAACAATGCCATGAGCTTGGGCTTCTGTTGCTGACATAAAAACATCCCTTTCCATGTCTTCGGATACAACCCATATAGGTTTGCCCGTTCTTTGTACATAAACCCTTGTGATGGTTTCGCGAAGTTTTAGTAGTTCTTCCGCTTCCAAGATAACTTCTCCCGTTTGTGCCTCATAAAACGAACTAGCGGGTTGATGGATCATTACCCTGATGATATAATAGAAAAGCTTTTTCTATTTCGCAGAATGAGGCGAGATAACCAAAAAAACAGAGAAATTTGAATAACCGTACAGGCTTTTTTTGTGCGTTGCATACGGCTCTAGAATGGAATTTACGTTTTTGACCTTTCCTTTCGGCGAAAGAAAACAAAATATAGGTTCTATTATACGCGGATCCATAAATGATCCAATTACCATCCTTCTTTTTTGTTTTGTAGGAGTTAAAAAAATACTATGATGGTTCCGTTGCTTTATATATCATTTTTTTTGATCCGTCTATGATTCAGCAATCCCAAAGTGTCTTTTTTTTTGTTTTTGTAAATAAGCTTCCGGTGTGAAAACAAAGTTTGTGACGCTGGGATGTGCCCGAATAGGGAAGATATCATTTTAAATACCCCTTTCTTATCCCATACTACTCTTTCAATATATAATCTAATTTTTTTAATCTAAAAAATTTCATATCGAATTCGAAGTGCCATGCTATTATTACTTAACTAATTCATATTTCCGAGGGCGAAGGCATAGTATTTTTTCTCTAAAATAAAAAAACTCATTGGCGCCAAGCGTGAGGGAATGCTATACGTTTGGTAATTTCTCCTCCGACTAGGATAAAGGATGCTATTGAAGCGGCCAATCCCATGCATATTGTCTGTACATCGGGTCGCACAAATTGCATAGTATCATAAATAGCCATTCCAGATATTACCCATCCACCAGGAGAGTTTATAAACAAATAAAGATCTTTGGTATCCTTTTCTATACTGAGATATATCATAAGACTAATAAGTTGATTCGAGATTTCGGTATCAACCTCTTGGCCTAAAAAAAACAATCTTTCTCGATAAAGTCGGTTGATTAGGATAAAATTTTATTCCTTAGGAGCCGTACAGGCACCTTTTGATGCATACGGTTCAACAAAAATTGTTAAAAAATCAATGTGTCGATTCCAACCCCCCCTTTTTTTTCAGAGAAGGCTTTTCTTTCTAACTTAATAAGGGAAGGGCTTGCTTCCCTTTTAAAAGTAAAAGAAAAAATAAATAAGTTTTGGCCCCTTTTATTTATTAGATATTATAATCCTAATAATAAAATAATAAAACGATTGATTAGGCCTGTCAGACTAACTTGATTCATTGATATTTTTTTTTCATCGAGATTCAGTTGAAATGGGGATGGTTTTTTCTTGTTCCTGAATGGGCTTCTTCCTTTTTTTATTCCGTTTTTTTAGGTTTATGCTCTACTCCGAGTAAAAGGAAAAATTTGCCCGATTTTGATTTGCACATATAGGACAAATGAACCAAATACCGCGTCTTTTTTTTTTTTACTACTCCTTCTTTTTTTTTCAATTCATTTCTTTCACATGTCTTCTGTCAAATAGTCAATAAATTTTTAATTATATTATTTTATTTGATCAACAGTTTTAGATCACCCTGTTTCAATTTTTTGTATTTTTTTTATTTTTTAATAGAATTTTTATCATAATTTTGATATCATATTCATATCATATTAAGTAGTAATTATAAAAATATTATATATTAATTATCAATTGGATTTTTGCTAAACGGAGCCTGGATACTTAATTTTATTAGTCCGATCACGTAAACCATAAAAAATTTTTGATAATCTAATATCAATCTAAATACTCCCTGCATTTAATTCTAATTTATTTTTTGCGCTTCGCGTTACAAATTTTTGATAATTCAATCAATCTTTTTGAGCGAAACAGAGGATATCTCGATCGAGGGAGAAAATGGGGAAATCCCATATAGCCCAATATATCTGACAAGTCGCACTATATGTCAACCCAAGATGTATCTCCTTCTCCAGGACTTCGAAAAGGTACTTTTGGAACGCCAATAGGCATGAAATGAAAAAAAAAGAGAATGAAGTTCTCTATTTCACTTTGATGTGGAAACGTAAGACTGGGGTTTCATTTTTTTTTATCATATTATCCTTTTTTCCTACTTTATTAATATTAATCATATTTAAATTAATCATATTTAATACAGAAGTTGAATAAGCTAAAATAAAATATAAAATAAAAGTAAAGTAAGAGAGAATGAATAAAAATTAAAGGAAACTTTTTACGAACGGGCTTCTGAACAACAATAGCTATCTTGGTTCATATAACATAGGATTCACCCCCATTGCGTATTGGTACTTATCGGATATAGAATAGATCCGCTTCCCTTTTTTCCTATGAATCGAATTGTTCCATTATTACTAACAGAATAGAACAAATATTAATCCTTTCTCCGAAATAATTACCTAAAAAGGGGGGGTCCGTAACATAGTTTTTTCCAATGCAATAAAGTTACATAGTGTCTATTTTTCATTGATAAAGGGGTATTTCCATGGGTTTGCCTTGGTATCGTGTTCATACTGTTGTATTGAATGATCCCGGTCGTTTGCTTTCGGTTCATATAATGCATACTGCTCTGGTTGCTGGTTGGGCCGGTTCCATGGCTCTATATGAATTAGCTGTTTTTGATCCCTCCGACCCTGTTCTTGATCCAATGTGGAGACAAGGTATGTTCGTTATACCTTTCATGACTCGTTTAGGAATAACCAATTCATGGGGCGGTTGGAATATTACAGGAGGGACTATAACGAATCCGGGTCTTTGGAGTTACGAAGGGGTAGCCGCAGCACATATCGTGTTTTCTGGCTTGTGCTTCTTGGCAGCTATTTGGCATTGGGTATATTGGGATCTAGAAATTTTTTGTGATGAACGTACAGGAAAACCTTCTTTGGATTTGCCCAAGATTTTTGGAATTCATTTATTTCTTTCAGGAGTGGCTTGCTTTGGTTTTGGCGCATTTCATGTAACAGGATTATATGGTCCTGGAATATGGGTATCCGACCCTTATGGACTAACCGGAAAGGTCCAACCCGTAAATCCGGCGTGGGGCGTGGAGGGTTTTGACCCTTTTGTTCCGGGAGGAATAGCCTCTCATCATATTGCAGCAGGGACGTTGGGTATATTAGCGGGCTTATTCCATCTTAGTGTTCGTCCGCCTCAACGTCTATACAAAGGATTACGTATGGGAAATATTGAAACCGTCCTTTCCAGTAGTATTGCTGCTGTCTTTTTTGCAGCTTTTATTGTTGCTGGAACTATGTGGTATGGTTCTGCAACTACTCCCATCGAATTATTTGGTCCTACTCGTTATCAATGGGATCAGGGATACTTTCAACAAGAAATATATCGAAGAGTTAGTGCCGGACTAGCTGAAAATCAAAGTGTATCAGAAGCTTGGTCTAAAATTCCTGAAAAATTAGCTTTTTATGATTATATTGGTAATAATCCAGCAAAAGGGGGATTATTCCGAGCGGGTTCAATGGACAATGGGGATGGAATAGCTGTTGGATGGTTAGGACACCCCGTCTTTAGAAATAAAGAAGGGCGTGAACTTTTTGTACGCCGTATGCCTACTTTTTTTGAAACATTTCCGGTTGTTTTGGTAGACGGAGACGGAATTGTTAGAGCCGACGTCCCGTTTAGAAGGGCAGAATCTAAATATAGTGTCGAACAAGTAGGTGTAACTGTTGAGTTTTATGGTGGTGAACTCAATGGAGTAAGTTATAGTGATCCCGCAACTGTGAAAAAATATGCTAGACGGGCTCAATTGGGTGAGATTTTTGAATTAGATCGTGCTATTTTGAAATCCGATGGTGTTTTTCGTAGCAGTCCAAGAGGTTGGTTTACTTTTGGGCATGCTTCGTTTGCTCTACTTTTCTTCTTTGGACACATTTGGCATGGTTCTAGAACCCTCTTCAGAGATGTTTTTGCTGGTATTGATCCAGATTTGGATGCTCAGGTGGAATTTGGGGCATTCCAAAAACTTGGAGATCCAACTACAAAAAGACAAGCAGTCTGATGCAACATTGCTTTTTTCTTTTAGTTTCTGTTTGCGATTTTTTTGATTTCATTTAATAGGTAGGGTACTGTAGGAATCTTGATTTAAATCGCTGCCGTTTCTTTGACTCTTTTTTGTTCTTTATCCGGAGGTATACTCCTTCAGTAAACATAAACAAAACAGGTATGAAAGCTATAATTGTAAACCACGATCAAATTTATGGAAGCATTGGTTTATACATTTCTCTTAGTATCGACTTTAGGGATCATTTTTTTCGCTATTTTTTTTCGGGAACCGCCTACAATTTCAACTAAAAAATGAAATAATTTTTCATTCTCTTCATTGACGTAATCAGCCTCCAACTATTTGGAGGCTGATTACGTCAACTAGTCCCCGTGTTCCTCGAATGGATCTCTTAGTTGTTGAGAGGGTTGCCCAAAGGCAGTATATAGAGCATACCCAGTAAAACTTACAAGTAACCCAGATATAAAGATGGCGACTAGAGTTGCTGTTTCCATTATTATATAATTGAAAGACCACAATGGATCTATGCTAAGATCGTTTATTTACAACGGAATGGTATACAAAGTCAACAGATCGTAATGAATACAAAATAAGATTTATGGCTACACAAACTGTTGAAGATAGTTCTAGATCTGGTCCAAGAAGCACTACTGTAGGGAAGTTATTGAAACCGTTGAATTCTGAATATGGTAAAGTAGCTCCTGGATGGGGAACGACCCCTTTGATGGGTGTTGCAATGGCACTATTTGCGGTATTCCTATCTATTATTTTGGAGATTTATAATTCCTCTGTTCTACTGGATGGAATTTCAATGAATTAGACTGAGAAGAATCTTGAAGTGCTAGCTTTTTGTTCGATACAAAAAAGTAAAGTATGTAGGTCTAAAATTTTGCACCTATTCTCCTTTGGTAGTTCGACCGCGAAATTTTTTTCTGCATTGTATATTTCCGGAATATGAGTGTGTGACTTGTTAGAATTGACCCTATGGATAGTACAGAGAAGGGGGTCTGTCATCTTTATCAAGATGGTTTTATTTCGTCGGATATTCATTCGAGTATCTGGAGCACGAAATAGATCAAATAGATCACAAAGTTTTCGAACTATGATTCATACTTAATACTTAGACCTCGTAGCCGGACTTCTTTCCGTTCTATCTTATAAATTTTCATAAATCAATTTTTTTCTGCTTTTAAACTCTTATTTAGATCAAAGGACAAACGCTTCTTTGTATTTTATGTTTTTAATCATTATAGCTCTTTTTTTTTTTATTGAATAAGTGATGATCCAATGGTTCTCACTCAGTGAACTTTGGACTTTGAAGGTTTCATTGAATTATCGTGGTTTTCGTATGAATCTGAGGTTTCAATTAATAAGTAGGGTCTTAACAAGAAAATTCCTATCAATAATAAAGAAAACAAGAAGAAATCCGTATTCCCATTCCATACAAATACCAACTAAAAAAGACAATAACGGTAGGTAATCTAGAAGATTCAAGAGGCCTGTAACGATCAACACAACATAAAGACGTATGAGCTGACTTGAGTTTTTGGCATTTAACCACAAAGAAGAGCTTTCGCATTTTGACTCTTAAATAATATTGAATGAGAGAGAAGTTTAAAACTTTATATTCCATATCCGTTTCAATCAGTATTTGGGTCTTTTTTTTGTTTGAGCTGTACGAGATGAAATTCTCATATACAGTTCTTGGAGGGGGAGGAACCTTGGTTTACCTATCTCAATAAAGTTTATGATTGGTTCGAAGAACGTCTTGAGATTCAAGCGATTGCAGACGATATAACTAGTAAATATGTTCCTCCGCATGTCAACATATTTTATTGTCTAGGAGGAATTACCCTTACTTGTTTTTTAGTACAAGTAGCTACGGGATTTGCTATGACTTTTTATTACCGTCCAACTGTTACTGAGGCTTTTGCTTCTGTTCAATATATAATGACTGAAGCTAACTTTGGTTGGTTAATCCGATCAGTTCATCGATGGTCGGCAAGTATGATGGTCCTAATGATGATCCTGCACGTATTTCGTGTATACCTCACCGGTGGTTTTAAAAAACCTCGCGAATTAACTTGGGTTACTGGTGTGGTTCTGGGTGTATTGACCGCATCTTTTGGTGTAACAGGTTATTCTTTACCTTGGGATCAAATTGGCTATTGGGCAGTCAAAATTGTAACAGGTGTACCTGACGCTATTCCGGTAATAGGATCGCCTCTTGTAGAATTATTACGCGGAAGTGCTAGTGTTGGACAATCCACTTTGACTCGTTTTTATAGTTTACACACTTTTGTATTACCTCTTCTTACGGCTGTATTTATGTTAATGCATTTCTTAATGATACGTAAGCAAGGTATTTCTGGTCCCTTATAAATAATATAGATTCTAGATATTTTTAATTACTAATTTATCTTATTACTTGGTGAAGGAACAATCGTATTTTATTGCTATAAATATGGATTATTAAAAAAATAAGACATGTATTTGGATATTTCCCTTCAACTCCACAATATTGTATTATTTTTTTGACATAAAAAGTTGAAGGGAATTCTATGAAGAGAAAATGGATTATGGGAGTGTGTGACTTGAACTATTGATCGGGCCGTGCAGAAATATGACTTTATCTGCTACATTGGAATTCACAACCAAATGTGTCTTTGTTCCAACCACTGTGTAAGCCCCATACAGGGGATAGGCTGGTTCACTTGAAGAGAATCTTTTCTATGATCATAATACCCGACGATGTCGTGGATGAGTGGGCTCCGTAAAATCCAAAAATCCAGGAGATTAAGGGATGGAACATAATCAGGATTATGTTTTTAGCTATTTTTTACTAAAAAAGAGCTAAAAAAATAAAAAATTAATAGTATGTAAATGCATTCATTTCCTCTGCATCGACTCGATTTCTGATACTATCGGAGTGAATACAGGATCTAATGAAGAGTAGAGGGTAGACTTCATTAGTAACAAGTAAATCCTTTGTATTTGAAAAATCTCGATATAATTTTTGAGATTAAGGATTAATTGATAAGGTATGAGACGATCCAGAAAGCACTTAATCATGATCAACTTTTAAGCTTACGTGGGTGTTGAGCATTTACCTGTAAGAATGGAATTTATGGTAATCTTTAGTTGCAATAACTTTGGAATCGGATAATTCTTTTTTTACATATTAAATACTTGTGGATAACATATATATTTTTTGTATGTATTAATTTAGTTTGGTTAATTCTTGCTCGAGCCGGATGATGAAAAATTATCATGTCCGGTTCCCTCGGGGGATGGATCCATAAGAATTCACCTATCCCAATAACAAAAAAACCAGATTTGAATGATCCTGTATTACGAGCTAAATTAGCTAAAGGTATGGGTCATAATTATTACGGAGAGCCCGCATGGCCCAATGATCTTTTATATATTTTTCCAGTAGTCATTCTTGGTACCATTGCCTGTAACGTAGGCTTAGCGGTTTTAGAACCATCAATGATTGGTGAACCTGCGGATCCTTTTGCAACTCCTTTGGAAATATTACCTGAATGGTATTTCTTTCCTGTATTTCAAATACTTCGTACAGTGCCTAACAAATTATTGGGTGTTCTTTTAATGGCTTCAGTACCGGCGGGATTATTAACCGTACCCTTTTTGGAAAATGTTAATAAGTTCCAAAATCCATTTCGTCGTCCAGTCGCGACAACCGTCTTTTTGATTGGCACCGTGGTGGCCCTGTGGTTAGGTATTGGAGCAACATTACCAATTGATAAATCTCTAACTTTAGGTCTTTTTTAATTAAATTTATTCAATTGTAAAATAAAAGGCGTGGGTATCTAGGGAGTAGTCATTTCAAAATGAATTCTCCCTAGATACATATCTAAATTAATTTTATTAAGTAAAATAGGTTTGACTGGAAAATCGAAATTACGTTGAAGGTTTAAAATCCATTTCAATTTTAAATTGACTTTTTAGTCAAATTTTTTTTAATGCTTTTTTTATTTTTTTTCTAAAATGTCTAATATCTTTTTTACATCTTCTATGTGAAAATGTTCCATTTTGATAAGGTCTTCTTGACTGTTATTCAAAAGATCCAATAATGTATGTATATTGGACTTTTTGAGACAATTATAGATTCTGGGAGGCAATTCTAATTGGTCAATAAAAATATATTGAAACGCTAGTTCTTTTTTTTTTTTTCTTAGGTTAACTAATCTATTATGAAAAGGAAAAAGGGGTAAAGTAACTTGATGTTGATTGTTCTCTAAATAGAACGTTTCTTCTTCTACATGTAGAAAAGGAATAAATAAATTAATCAAATTCCGGGAGGCTTCATGAAGTGCTTCTTTAGGAGTTAAACTTCCATTTGTCCATATTTCTAGAAAAAGAATCTCTTGTTTTTCATTCCCATTCCCATAAGAATGAATACTATGATTCGCATTTTGAACAGGCATGAATACAGCATCTATAGGATAACTTCGGTCTTCAAAGTTATTTGACATTTTTAAACTATATCCGCGATTCCTCTCGATTTTTAATCCAATACACAAATTTATTGGTTCCGTTAAGGTAGCTATATGCTGTGTATTATCAACGATTTCCACAGAGGGCGGTAAAACTATGTCTCGAGCAGTTATATATCCGGGACCTTGGACACAAATAAGCGCGTTGCGCGTTCCATATAGATTACTTTTTAATACAATCTCGTTCAAATTCATTAAAATTTCATGTACTGATTCTTGAATACCGACTATGTTAGAATAGTCATGTGGTATGTTCTCAGATTTTGCACGTGTAATACATGTTCCTTCTATTTCGCCAAGTAAAGCTCTTCGCATCGCAATGCCTATTGTGTCGGCTTGACCTTTCATAAGTGGAGACAGAATAAAGCGTCCATAATAAAGACGCTTACTGTCTCTTCTTGATTCAACACACTTCCACTGTAGTGTCCGAGTAGATACTTTGACTTTCTCTCGAACCATAGTAATTTTATTTGATCAGATCATTGAATCATTTATTTCTCTTGAAACCCTTTCAGCCTTTATTTAGTTCTATACACGTCGTTTTTTAGGGGGTCTACAACCATTATGTGGCATAGGGGTTACATCTCGTACGAAACTTAAAAGTATACCGCTTCTACGAATAGCTCGTAATGCTGCATCTCTTCCTAGTCCAGGGCCTTTTATCCTTACTTCAGCTCGTTGCATACCTTGATCCACTACTGCTCGAATAGCATTTCCTGCTGCGGTTTGAGCAGCAAAAGGTGTTCCTCTTCTTGTACCCCTGAATCCACAAGTACCCGCGGAGGACCAAGAAATCACCCGACCCCGTACATCTGTAACGGTCACAATGGTATTGTTGAAACTTGCTTGAACATGAATAACTCCCTTTGGTATTCTACGTACATTTTTACGTGAACCACTACGGGTATTTTTACGTGAACCAATTCTTAATATAGGTTTTGCCATATTTTTTCATTTCACAAGAAATATATGGATATATCCATTTCATGTCAAAACGGACCTTTTTTTTACTAGCTCCTTGGAAGTGCCTTTTCCTTTAGTAAGATTATCCTTGTCTTTGTTTATGCCTCGGGTTGGAACAAATTACTATAATTCGTCCCCTCCTACGGATTAGCCGACACTTTTCACAAATTTTACGAACGGAAGCCCTTATTTTCATAGTTGTTATTCCTTAATTCTCTTAATATACTTATTGTTGGACGAAAAAAAGGTTTCTTGATATTTTTGAATCTTGAATTGTATCTTCGTGAAAGGAATGTTGAATTTCAAAAAACCACTGACTTATTTGAATCCTTGTTATGGAGTCTAGAAAGTGGCTGTTCCCCGATTAACTTAATACCTAAGAACTTACTAAAATTTTTACCCCCCTTTTTCTCCTATAGGTATACCTATACAAAAATATGTCGAATCCTTTCAGAAGCATGACCTAAAATAAAAAAAATCTTTAGTATCTAAACAAAATCGAACCATACCGTTCGGAAGTGATTCATAAAGAAAACTTTCATTAATTCATTTTTTTCTTTAATTTCATTCGGGGTAAAAAATTCTAAACTTTTTTAGCAGGGGTGGTATTACACAACCCCCCCCCCTTTTTTTTCACAAATGCTAAGTTCCGGATATCCAATTTTGATATTAGAAGGATTACCATATATAACACAAAATTTCTCCGCCGATTCTTTTTAGTCGAGCTTCTCGATCTGTCATTATACCTTGAGAAGTTGAAAGGATTACAATTCCTATTCCGCCTAAAATTCGTGGAATTCGTTGAGAGTTAGAATAGATTCGTAGACCCGGTCGGCTTATTCTCTTTAAATTTAAAATCGTTTTATAGGATTCTTTCTTATTTCGTCTATGTCTTAGGGTTAAAATCAAAAAATATTGATTGTTTTCGCGATGTTTCCTTACGTTTTCGATAAAACCCTCTCGTAAAAGTATTTTAACAATGCTTTCGGTGATGTTAGTCGATCCTATCCGAACTGTTCCTTTTCTATTCATGTCAGCATTTCGTATAGAGGTTATTATATCAGCAATAGTGTCTTTCCCCATGATAAGTTAAAATTCCTTAATTGTTCTATAATTTTGATATAATCAACATGTTATTTTTCTTTTATTTATATAAAAATAGAGACGAATTATATATTAATATATGAATTCAATTATTAATATATAAAATTATTAAGGGTATATGCGTGATACACAATCTATTAATTATAATTAATTTGATTTCAATACCATTTTTTTAATCCTATCCTATATTAACTATCGATATTTAGGTCTTATAATACTTCAGGAGCTAATGAAACTATTTTAGTAAAGTTTAATTGTCTCAATTCCCGTGGGATCGCCCCAAAAACGCGAGTTCCTTTTGGATTTCCTTCTTGATCAATGACAACTGCGGCATTGTCGTCATATCGTATTATCGTCCCATTCTTACGTTTGAGTTCTTTACAAGTACGTACAATTACAGCTCTGACCACTTCTGATCTTTCTAGAGTAGTATTTGGGATTGCTTCCTTGATTACAGCAACAATAACGTCACCAATATGAGCATAGCGGCGATTACTAGCTCCTATTATTCGAATACACATCAATTTTCGAGCCCCGCTGTTGTCTGCTACATTCAAATAGGTTTGTGGTTGAATCATATTTTTGTATCTCTTCTTTTAGTGCAAAGGACGAAGTAAAAAAAATATTGTTTGTCAAAAAAAACTTAGAATCTTTTTATCCTTAAATGTTATTTAGCTTTTTCATTCTATATTCCTATTCAGAAATAATGAATTGGGTTTTTATAGGCATTTTTGATGCCGCGATTGAAATAGCTTTTCTGGCTATATTTTCTGGTACACCACCCATTTCATAAAGGATTTTACCTGGTTTAACCACAGCTACCCAGTACTCTGGGGATCCTTTCCCAGAACCCATACGCGTTTCCGCGGGTCTTACTGTAACTGGCTTGTCTGGAAATATACGTACCCAAATTTTTCCACCACGTCGTATATTTCGTGTCATTGCTCGTCGCCCTGCTTCTATTTGTCTAGATGTAATCCAAGCGGGTTCAAGTGTTTGAAGAGCATATCTGCCAAAACAAATACGATTCCCACGAGAGGATATTCCTTTTAGTCTTCCTCGATGTTGTTTACGAAATTTGGTTCTTTTTGGGTTATAGTTGATGGGTTTTTTCTAAATGAGAAATTCCATCTCTACTGCAGAACTGGACGTGAGAGTTTCTTCTCATCCAGCTCCTCGCGAATAAAAGGATTAATTAAGATATAGATGTAGTTAATGATTAATCCTATTAATCATGGTATTTTTTTTTTATTTCATCTTATCTCTTCTAAATTTGTGTATGTCTTTTTTGAAATAGAATCAAAGATCAATTTTATTTCGATTTATTTAAAAATAACGTAATATCATCATTACAAATGTAATTTTTATTAGAGTTAGAATATTATAACAAATCCTTATTTTTTTTTCATTGTTTTTTTCATCTTTTATTACTGTTTTTATTTGAAAAAAAAAAACCAATTTTTCGCCGGCGAATATTTACTCTTTCAATATCTATTTAAGTTTGCTGTTTATCCCCCGAGGTCTCAGAATCAAAATCAGAATAGATAATAAAGTTTCTGGTTTATTCCGCCATCCTGTCCAATGAATTACTAAGATTTCTTGTTCACTAGAATCCTATATATTCATGGGTTCCGTCGTTCCCATCGCTTCTTGATTAATCATTAGGCCTGAATTCTACAATGGAGCTTTTACATGAAATTTTGAATTTCTTTTTTTTTTGAGGCAATTTTCTCAGTTTTGATTGTCTCAAGGCTCTTAATTTTTTGTTTTCGGAACAGATTTATCTAATTATTATTATTTATCTAATTATTATGAATGAATCTGTATTGATGCTTTATTACATTGCTTTTCTTACAGTGACCTCATAGATTTTCCAAATTGGAATCATATATCATTAATATTCAATTTTTTCGCTCTTTCTTTCATCCTTCCATTTATCCGCATACTTTTTGATTACCTTTCATAACTTAATAATCATCTTTCTTTATTCTTTTTTTTTAGTCAGTTGCTCCAATGATATGATCAGCCTATCATATCTTGACTAATTTTTTGGATCCAGATAATGCGAAGCAATGAGTTGCTTAGGTTATTTATTAATGCTGTAGTTATTAGTTGGTAAGTTCTTTTTTTTTTTTATCGTAATCTAACCCTAAACCAACGAGTCACACACTAAGCATAGCAATTATATCAAAGGAGTTTTGATGGAAATGTTTATTCAACCTTATAGAATTGCTTATTTTTTTTTCTTAAACATAAAAAAGAAGACTACAAGTTTTTATTTTTATTTCTTTATAGTGTTATACTACATAGTTTTCGTTTTTTATCATTGGATAAAATGTAAAGACAAATAAAGTTTTTTTATTCTTCGTCTACGAATATCCAAATTTTTATTCCTAAAACCCCATAAATAGTTCGAACTGTATAGGAACAATAATCAATTTTAGCTTCAATTGTTTGTAAAGGAACTCTGCCTTCTCTGATCCATTCAACACGTGCAATTTCTTTTCCGTCGATACGTCCTGCAATTTGTACTTGAATTCCTTTTGTATTCGCCTGTTCAGTTAATTCAATAGCTTTTTTCATTGCTTTTCGAAAAGAAACGCGATTTTTTAATTGGCCAGCTATAAATTCTGCAAGAATATTAGGATGCCCATACGGATTGGAAATTCGGGTAATAGCAATGTTGAGTTTTCTATTGACACAATTAAGTTCTTTTTGAACATTCATCTGTAATTCTTCGATTCTTCGGGGTTTATCTTCAATTAATAATTTAGGAAATCCCATATAGATTATGATCTGAATGAGATCGATTCTTTTTTGAATTTCGATACGTGCAATTCCCTCCATACCAGAGGATATTCTTATATTTTTTTGGACATAATTTTTAATACAGTCTCGTATTTTTTTATCTTCTTCTAAACCTTCAGAATACTTTTTTGGTTGTGCAAACCAAATAGAATGATGACTTTGGGTTGTACCAAGTCTGAAACCAAGTGGATTTATTTTTTGTCCCATGGGCCTCCACTACTATATGTATCGTAACATGTTAGATTTATGTTTTCATTGCTGCATCCAGGTTTTTTTAAATACATTAAATATTCTTCATATTGTTGATATAAGGATATATCTTCCAATACGATAGTTATATGACAAGTGGATCTTTTTATTGGGTAACTCCGTCCTCGTGCCCGAGGTTTTAATTTTTTCACCGTATTTCCTTGATTCACTTCAGCTTTACTAATGACTAAATTGGTTTCTTTGAAACCCTTATTATGACTAGCATTTGCTGCTGCAGAATAAACTAATTTAAAAATGGGATAACATCCTCGATACGGCATAAGTTCTAATATCATAAGTGCTTCTTCGTAGGAACGTCCACGGATCTGATCAATAACTCTCCGTGCTTTGTGGGCAGACATAGATATATATTGCCCTAAAGCATATACGGAAGTATATGATTTCTTCTTTTTCTTCTTTATCATAAGGTTTACCTCTCACTAAAAAAAAAAATTATATTCATTATTCATTTTTTTGAATTCATTTAATTAACGACGAGATCTATTATCATTTTTCGCGTGTCCTCTAAAATTTATAGTAGGTGAAAATTCTCCCAATTTATGTCCTACCATAAGGTCGATTATATAAACGGGTAAGTGTTCCCTTCCATTATGTATAGCGATAGTATGGCCAATCATTGTGGGTATAATAGTAGATGCCCGGGACCAAGTTATTATGATTTCTTTTTCCGCCTTTGTATTAAGCTTCTCTATTTTTCTTAATAAATGCTTTGCTACAAAAGGATTTTTTTTTAGTGAACGTGTCACAGTTAATTAACTCCTATTTTTTTTAAGACGAAGAAAGAAATTCGATTTTCTCTCCTATTTACTACGGCGACGAAGAATCAAAGTCTCACTATATTTTTTCCTTTTTCTAGTTCTTCTTCCAAGCGCAGGATAACCCCAGGGGGTTACGGGTTTTTTTCTACCAATTGGAGCCCTCCCTTCACCACCTCCATGGGGGTGGTCGACAGGGTTCATAACTACTCCTCTTACTACAGGACGTTTACCTAGCCAACATTTCGATCCGGCTCTACCCAAACTTTTCTGGTTTACCCCAACATTTCCCACTTGTCCGACTGTTGCTGAGCAGTTTTTGGATATCAAACGGACCTCTCCAGAAGGTAATTTTAATGTGGCCGATTTCCCCTCTTTTGCAATCAGTTTCGCTACAGCACCCGCTGCTCTAGCTAATTGTCCACCCTTTCCAAGTGTGATTTCTATATTATGTATGGCCGTGCCTAAGGGCATATCGGTTGAAGTAGATTCTTCTTTTTGATCAATCAAAACCCCTTCCCAAACTGTACAAGCTTCTTCCAAAGCATACGGCTTTCTGAATGTAGATGATGATATCTATACGGATGGATCTTATCTTATATATATCGTAGAATTCTTCTATATATGGTAGAAGTACCACACGAGTGGATATATAGGAATCAAAATCTGCCGAATAACTTATGTTATGATCTTCTACATCCTAGGTCTTCCCGTTCCGTCATCTGGCTTATGTTCTTCATGTAGCATTCAGACCGAATGACTCTATGAAATTACGTCGATACTTCCACATATTATGGGTAACGTAGGAGACATCTCTATTTTTCCCCGGGGGAATCTTTAGAATTACCACTGCTTAGCTTTCAATTCGCCTCTGACCATCAAATGAAATGTGAATAACCCGTCCTCCTCTCTTTGAAACAAGGGGCGCTTATGGTTCTGTCGGTGCTTGAAACAATTTTGTCTTCTCCATATTACTATATCTCTAGAGTCAATAATTTTATATGAGGAACTACTGAACTCAATCACTTGCTGCCGTTACTCTTCAGTTTTCTGTTGAGGTCTATCCTGCAGAGGTACTCAAATTGGATCAGTGATCGATTTCTAGGTTTTGTCGTAAACCTAATTGGTTACTTCCAATTACGTAAATCAAATAGTTCAAACCGCACTCAAAGGTAGGGCATTTCCCATTTTTATAGGAACTTCTGTACCAGAAACAATGGTATCTCCAATTATAGCCCCTCTGGGATGTAAAATATATCTCTTCTCACCATCCCCATAGTGTATGAGACAAATGTATGCATTTCGATTAGGGTCGTATTCTATGGTTACGATTCTACCATATATGTCTTTTGTATTTCGTCGAAAATCTATTTTACGGTATAGACGCTTATGACCTCCCCCTCTATGCCTTACGGTAATGATTCCTCTGGCATTACGACCTTTACCACAATGATGCTGCCCATAGATCAAATTATTTCGTGGATTGGATTTCACTTGACTGTCTACGGCTCCATTGCGTGTGCTCGGGGTAGAAGTTTTGTATAAATGTATCGCCATGCTATTAAGTATTTTGATTTAAGTTCTTTTCTTTCTAAGAGGTGGAATAGAATAACCCGGTTGAAGCGTAATGATCATACGTCTGTAATGCATTGTATGTCCCAGAATAGGTCCCATTCTTTTAACCTTTCCGGGGAGTCGATGACTATTCATAGCTATTACCTTGACACCAAAGAAGAGTTCGACCCAATGCTTTATTTCTGTCCTAGTTGATCCTGATTCGACATTAAAAGTATATTGATTTTTCCCCAATAACCGAATACTTTTGTCTGTAAATACTGCATATTTGATTCCATCCATAAATCGATTTTCTTCCCTATGAGTTCTAGTCTCAATAAGAATGCTAGTTCTTACTGTTCATATGTTATGTTATGATATGAATATACCACACCAATTCGTTATGTATAGATGATGAGAAGATTCCATTGATACAGAGCCAATTCCAATAGACTTATTGGAGGGTCCCATTGGCGTGCATCCAGTAGGAATTGAACCTACGAATTCGCCAATTATGAGTTGGGCGCTTTAACCATTCAGCCATGGATGCTTAGTGGGGATCCTCGTACATGGTGAATAACCAAATTCCAATTGAAATGAAATCTTTAGGATAAATCAATGCAATTTAGGAGGAATCAATGAAAGGACATCAATTCAAATCCTGGATTTTCGAATTGAGAGAAATAGTGAGAGAGATCAAGAATTCTCACTATTTCTTAGATTCATGGACCCAAATCAATTCAGTGGGATCTTTCATTCATATTTTTTTCCACCAAGAACGTTTTAGAAAACTCTTGGACCCTCGAATTTTTAGTATCCTACTTTTGCGCAATTCACAGGGTTCAACAAGCAATCGATATTTCACGATCAAGGGTGTAGTACTATTTGTAGTAGCGGCCCTTCTATATCGTATTAACAATCGAAATATGGTCGAAAGCAAAAATCTCTATTTGAAAGGGCTTCTTCCTATACCTATGAATTCCATTGGACCCAGAAATGATACATCGGAAGAATCTTTTGGGTCTTCCAATATCAATAGGTTGATTGTTTCGCTCCTGTATTTTACAAAAGGAAAAAAGATCTCTGAGAGCTGTTTCCGGGATCCGAAAGAGAGTACTCGGGTTCTCCCAATAACTAAAAAGTGTATCATGCCTGAATCTAACTGGAGTTCGCGGTGGTGGAGGAACTGGATCGGAAAAAAGAGGGATTTTTGTTGTAAGATATCTAATGAAACCGTCGCTGGAATTGATATCTCATTTAAAGAGAAAGATATCAAATATCTGGAGTTTCTTTTTGTATATTATATGGATGATCCGATCCGCAAGGGCCATGATTGGGAATTGTTTGATCGTCTTTCTCCGAATAAGAGGCGAAACATAATCAACTTGAATTCGGGACAGCTATTCGAAATCTTAGTGAAAGACTGGATTTGTTATCTCATGTTTGCTTTTCGTGAAAAAATACCAATTGAAGTGGAGGGTTTCTTCAAACAACAAGGAGCTGGGTCAACTATTCAATCAAATGATATTGAGCATGTTTCCCATCTCTTCTCGAGAAACAAGCGGGCTATTTCTTTGCAAAATTGTGCTCAATTTCATATGTGGCAATTCCACCAAGATCTCTTCGTTAGTTGGGGGAAGAATCCGCACGAATCGGATTTTTTGAGGAAAATATCGAGAGAGAATTGGATTTGGTTAGACAATGTGTGGTTGGTAAACAAGGATAGATTTTTTAGCAAGGTACGAAATGTATCGTCAAATATTCAATATGATTCTACAAGATCTAGTTTCGTTCAAGTAACGGATTCTAGCCAATTGAACGGATCTTCTGATCAATTCATAGATCCTTTCGATTCCATTAGTAATGAGGATTCGGAATATCACTATCACACATTGATCAATCAAAGAGAGATTCAACAACTAAAAGAAAGATCGATTCTTTGGGATCCTTCCTTTATTCAAACGGAAGGAAGAGAGATAGAATCAGACCGATTCCCTAAATACCTTTCTGGATATTCCTCAATGCCCCGGCTATTCACGGAACGTGAAAAGCGAATGAATAATCATCTGCTTCCGGAAGAAAGCGAAGAATTTTTTTGGAATTCTACAAGAGCCATTCGTTCTTTTTTCTCTGACAGATGGTCAGAACTTCATCTGGGTTCGAATCCTACTGAGAGGTCCACTAGGGATCAGAAATTGTTGAAGAAAGAACAAGATGTTTCTTTTGTCCCTTCCAGGCGATCGGAAAATAAAGAAATAGTTAATATATTCAAGATAATTACGTATTTACAAAATACCGTCTCAATTCATCCTATTTCATCAGATCTGGGATGTGATACGGTTCCGAAGGATGAACTGGATATGGACAGTTCCAATAAGATTTCATTCTTGAACAAAAATCCATTTTTTTATTTATTTCATCTATTCCATGAACGGAAGAGGGGGGGATACACGTTACGCCACGATTTTGAGTCAGAAGAGAGATTTCAAGAAATGGCAGATCTATTCACTCTATCAATAACCGAGCCGGATCTGGTGTATCATAAGGGATTTGCCTTTTCTATTGATTCCTACGGATTGGATCAAAGACAATTCTTGAAGGAGGTTTTCAACTCCAGGGATGAATTGAAAAAGAAATCTTTATTGGTTCTACCTCCTATTTTTTATGAAGAAAATGAATCTTTTTATCGAAGGATCAGAAAAAATTGGGTCCGGATCTCCTGCGGGAATTTTTTTGAAGATCCAAAACCAAAAAGAGTGGTATTTGCTAGCAACAACATAATGGAGGCAGTCAATCAATATAGATTGATCCGAAATCTGATTCAAATCCAATTCCAATATAGTCCCTATGGGTACATAAGAAATGTATTGAATCGATTCTTTTTAATGAAGAGACCTGATCGCAACTTCGAATATGGAATTCAAAGGGATCTAATAGGAAATGATACTCTGAATCATAGAACTATAATGAAAGATACGATCAACCAACATTTATCGAATTTGAAAAAGAGTCAGAAGAAATGGTTCGATCCTCTTATTTTTCTTTCTCGAACCGAGAGATCCATAAATCGGGATCCTAATGCATATAGATACAAATGGTCCAATGGGAGCAAGAATTTCCAGGAGCATTTGAAACATTTCGTTTCTGAGCGGAAGAGCCGTTTTCAAGTAGTGTTCGATCGATTATGTATTAATCAATATTCGATTGATTGGTCTGAGGTTATTGATAAAAAAGATTTGTCTAAGTCACTTCGTTTCTTTTTGTCCAAGTTACTTCGTTTTTTGTCCAAGTTACTTCTCTTTTTGTCTAACTCACTTCCTTTTTTCTTTGTGAGTTTCGAGAATATCCCCATTCATAGGTCTGAGATCCACATCTATGAATTGAAAGGTCCGAACGATCAACCCTGCAATCAGTTGTTAGAATCAATAGGTCTTCAAATCGTTCATTTTAAAAAATTGAAACCCTTTTTATTGGATGATCATAATACTTCTCAAAAATCGAAATTCTTGATCAATGGAGGAACAATATCACCATTTTTGTTCAATAAGATACCAAAGTGGATGATTGACTCATTCCATACTAGAAAGAATCGCAGGAAATCTTTTGATAACACGGATTCCTATTTCTCAATCGTATCCCACGATCAAGACAATTGGCTGAATCCCGTGAAACCATTTCAGAGAAGTTCATTGATATCTTCTTTTTCTAAAGCAAATCGACTTCGATTCTTGAATAATCCACATCACTTCTGCTTCTATTGTAACAAAAGATTCCCTTTTTATGTGGAAAAGGCCCGTCTCAATAATTCTGATTTTACGTATGGACAATTCCTCACTATCTTGTTCATTCACAACAAAATATTTTCTTCGTGTGGTGGTAAAAAAAAACATGCTTTTTTGGAGAGAGATACTATTTCACCTTCGTCAATCGAGTCACAGGTATCTAACATATTCATATCTAACGATTTTCCACAAAGTGGTGACGAAAGGTATAACTTGTACAAATCTTTCCATTTTCCAATTCGATCCGATCCATTAGTTCGTAGAGCTATTTACTCGATTGCAGACATTTCTGGAACACCTCTAATAGAGGGACAAAGAGTAAATTTGGAAAGAACGTATTGTCAAACTCTTTCAGATATGAATCTATCCGATTCAGAAGAGAAGAGCTTGCATCAGTATCTCAATTTCAATTCAAACGTGGGTTTGATTCACACTCCATGTTCTGAGAAATATTTACAGAGGAAAAAACGGAGTCTTTGCCTAAAAAAATGCGTTGACAAAGGGCAGATGGATAGAACCTTTCAACGAGATAGTGCTTTTTCAACTCTCTCAAAATGGAATCTATTCCAAACATATATGCCATGGTTCTTTACTTCGACAGGGTACAAATATCTAAATTTGATATTTTTAGATATTTTTTCAGACCTATTGCGGATACTAAGTAGCAGTCAAAAATTTGTATCCATTTTTCATGATATTATGTATGGATTAGATATATCATGGCGAATTCTTCAGAAAAAATTGTGTCTTCCACAAAGGAATCTGATAAGTGAGATTTCGAGTAAGTCTTTACATAATCTTCTTCTGTCCGAAGAAATGATTCATCGAAATAATGAGTCATCGTTGATATCGACACATCTGAGATCGCCAAATGTTCGTGAGGTCCTCTATTCAATCCTTTTCCTTCTTCTTGTTGCTGGATATATCGTTCGTACACATCTTCTCTTTGTTTCCCGAGCCTATAGTGAGTTACAGACAGAGTTCGAAAAGATCAAATCTTTGATGATTCCATCATACATGATTGAGTTGCGAAAACTTCTGGATAGGTATCCTACATCTGAACAGAATTCTTTCTGGTTAAAGAATCTTTTTCTAGTTGCTCTGGAACAATTAGGAGATTGTCTAGAAGAAATACGGGGTTCTGGCGGCAACATGCTATGGGGTGGTGATCCCGCTTATGGGGTCAAATCAATACGTTCTAAGAAGACAGATTTGAAAATAAACTTCATCGATATCATCGATCTCATAAGTATCATACCAAATCCCATCAATCGAATCACTTTTTCGAGAAATACGAGACATCTAAGTCATACAAGTAAAGACATCTATTCATTGATAAGAAAAAGAAAAAACGTGAGCGGTGATTGGATTGATGATAAAATAGAATCCTGGGTCGCGAACAGTGATTCGATTGATGATAAAGAAAGAGAATTCTTGGTTCAGTTCTCCACCTTAAGGGCAGAAAAAAGGATTGATCAAATTCTATTGAGTCTGACTCATAGTGATCATTTATCAAAGAATGACTCTGGTTATCAAATGATTGAACAACCGGGAACAATTTACTTACGATACTTAGTTGACATTCATAAAAAGTATCTAATGAATTATGAGTTCAATACATCCTGTTTAGCAGAAAGACGGATATTCCTTGCTCATTATCAGACAATCACTTATTCACAAACTTCGTGTGGGGCTAATAGTTTTCATTTCCCGTCTCATGGAAAACCCTTTTCGCTCCGCTTAGCCCTATCCCCCTCTAGGAGTATTTTAGTGATAGGTTCTATAGGAACCGGACGATCCTATTTGGTCAAATACCTAGCGACAAACTCCTATGTTCCTTTCATTACAGTATGTCTGAACAAGTTCCTGGATAACAAGCCGAAAGGTTTTTTTCTTGATGATATCGATATTGATGATAGTGACGATATTGATGCTAGTAACGATATTGATCGTGAACTTGATACGGAGCTGGAGCTTCTAACTATGATGAATGCGCTAACTATGGATATGATGTCGGAAATAGACCGATTTTATATCACCCTTCAATTCGAATTAGCAAAAGCAATGTCTCCTTGCATAATATGGATTCCAAACATTCATGATCTTGATGTGAATGAGTCGAATTACTTAGCCCTCGGTCTCTTGGTGAACTCTCTCTCCAGGGATTGTGAAAGATGTTCGACTAGAAATAGTCTTGTTATTGCTTCGACTCATATTCCCCAAAAAGTGGATCCCGCTCTAATAGCCCCGAATAAATTAAATACATGCATTAAAATAAGAAGGCTTCTTATTCCACAACAACGAAAGCACTTTTTCACTCTTTCCTATACTAGGGGATTTCACTTGGAAAAGAAAATGTTCCATACTAATGGATTCGAGTCCATAACCATGGGTTCCAGTGCACGAGATCTTGTAGCACTTACCAATGAGGCCTTATCAATTAGTATTACACAGAAGAAATCAATTATAGACACTAATACAATTAGATCTGCTCTTCATAGACAAACTTGGGATTTGCGATCCCAGGTAAGATCGGTTCAGGATCATGGGATCCTTTTCTATCAGATAGGAAGGGTTGTTGCACAAAATGTACTTATAAGTAATTGCCCCATAGATCCTATATCTATCTATATGAAGAAGAAATCATGTAACGAAGGGGATTCTTATTTGTACAAATGGTACTTCGAACTTGGAACGAGCATGAAGAAATTCACGATACTTCTTTATCTTTTGAGTTGTTCTGCCGGATCGGTCGCTCAAGACCTTTGGTCTCTACCCGGACCCGATGAAAAAAATAGGATCACTTCTTATGGATTCATTGAGAATGATTCGGATCTATTTCATGGCCTATTAGAAGTGCAAGGCGCTTTGGTGGGATCCTCACGGACAGAAAAAGATTGCAGTCAGTTTGATAATGATCGAGTGACATTGCTTTTTCGCTCCGAACCAAGGGATCCCTTATATATGATGCAAGATGGATCTTGTTCTATCGTTGATCAGAGATTTCTCTATGAAAAATACGAATCGGAGTTTGAAGAAGGGGAAGGAGAAGCAGTCCTCGACCCGGAACAGATAGAGGAGGATTTATTCAATCACATAGTTTGGGCTCCTAGAATATGGCGCCCTCGGGGCTTTCTATTTGATTGTATCGAAAGGCCTAATGAATTGGGATTTCCCTATTTGGCCGGGTCATTTCGGGGCAAGCGGATCATTTATGATGAAAAGTATGAGCTTCAAGAGAATGATTCGGAGTTCTTGCAGAGCGGAACCATGCAGTACCAGAGACGAGATAGGTCTTCCAAAGAACAAGGCTTTTTTAGAATAAGCCAATTCATTTGGGACCCCGCAGATCCACTCTTTTTCCTATTCAAAGATCAGCCCTTTGTCTCTGTGTTTTCACATCGAGAATTCTTTGCAGATGAAGAGATGTCAAAGGGGCTTCTTACTTCCCAAACAGATCCTCCTACATCTATATATAAACGCTGGTTTATCAAGAATACGCAAGAAAAGCACTTCGAATTGTTGATTCAGCGCCAGAGATGGCTTAGAACCAATAGTTCATTATCTAATGGATTTTTCCGTTCTAATACTCTATCCGAGAGTTATCAGTATTTATCAAATCTGTTCCTATCTAACGGAACGCTAGTGGATCGAATGACAAAGACATTGTTGAAAAAAAGATGGCTTTTTCCGGATGAAATGAAAATAGGATTCATGTAATGTAACAGGAGAAAGGTTTCCCATTACTTAGCCGGAAAGATATGTGTCCATGAAATAGGGATTAAGTGGAACGGAATTGACTGGGTGGTAGAGTTGTAGAAACACCTGTTTCTTCCACTTAGCTCCATGGAACAATATGCTACGACGGAAACATGGAAGAATTGAAATCTTAGATCAAAACACTATGTATGGATGGTACGAACTGCCTAAACAAGAATTCTTGAACAGCGAACAACCAGAGCTATTACTCACTACATCAAAAAAATTTCCATTAATGAAGGATGGAAATCCATTGGAAAATCAAAAATACGCATGTCGGATGAAATTGTTGTTGCTATCTGTTCCAATAACGAATCAACTGAATAACTAAATAAAATAGATAGACCTTTCTCTTCGTCTCAGGTCGATAGATCTTCTCAATTGGAAGATCCCCTATATGGATAATACACATTCCAGTTGACCGAGCCTAATTCTAATTGTTTTGTTCCGAAGTAAAGATATCCACGGAGTGGTTCGCCCTATTCAGATATTCACGACCAAGAAGTACTGGATTCTGTTTAGGATAGGTCCTGAAAGGAGAAGGAAGGCTGGAATGCCGCCAGGCGTCTATTATTGAATTCACCCGACCCGATAGTACCAATTTTGGTAACGTCCATCCAGTGCCAAAGTCACTGAATGGGTAAGTCACCAATCCCTAAAACGGACTATGTACTTTATCTGCTGGGTTACGGGGGCATTTTACCAGAGGTTTAGATTGTATCAATCTACCCTTGTGTGATTCCTGTTGAATCATATACTGCGGGGCGCAGGGCGGACGATTTCAAAGCGGACTCCCCCTCCCCATTCATTAGATAGAGAAGATCGCCAAGATTTCGCGATCCGCTGCCGAACTTATTCCATTTCAATATTATGCCTTGAAGAGGACTCGAACCTCCACGCTTTTTAGCACGAGATTTTGAGTCTCGCGTGTCTACCATTTCACCACCAAGGCATCTTGAAAGTGAATCGTATTCCATAAATATGATATCTATCTAGTACGGTGTATTGAATATATGACAAAGGTGGAGTGTTGAAGTATTTCTATTGATCGGTCATGTCATATAGGCCCGAGTCGGACATCTAATTGCTTAGATTTGAATTATCCTTATCCGGAGGATGCCTTATATATATATTAATATTATATCAAAAAGATGGACAATCAAACCTATTTCTCGATTCAATAGAAGTCCAACCAAAGAGGTGAATAGGGTCCCAAATAACGAGAGATATGTAAAAAGTAGGTCAGATTTCGCCTATTCCTAATCCTAAATGGAATGTAACGACGTAGGGATCCCTATGTAAACATAGTATCTATTTAGATACGCTCGAATGACCCCTTCTCATAATGAGAATGTATATAACCTTATTCCGGTCTGGTCCGGTATGGAATGAACTTATAATCATGGAATCGACTCGATCATCAGATTATAGATTATAAGTTCATAACCTTAGTCCATTCCCATTTTGGGCGGAACCGATCTACTAATTCTTTGATTCCAGTTAGTAAGAGGGATCTTGAACTAAGAAATAGATTCTAGAAGCTAAAAAGGGTATCCTGAGCAATCGCAATAATCGGGTTCATTGATATTCCTGGTATAGTAGATGCTATCACACATACAATCATACTCAATTCGATGGAATTGGTTGATCTTAAAGGGGATATTCTATAATTTCGCACGTGAGGGGTTATTTCTTGGTTTCGTCCAGTCATTAATAACTTGATTATTTTTAGATAATAGTAGATAGAAAGAACGCTCGTAAGGAGTCCTATTGAAACCAAGAAATATAGGCCTGCCCGCCATCCACACCAGAATAAATGGAGTTTTCCAAAAAAACCTGCTAGTGGAGGAAGACCTCCTAGGGATAAGAGACATAGAGCTAAAGAGAGAGCCAAAAAAGGATCTTTTGTGTATAATCCTGCATAATCTCGAATGTTATCAGTTCCGGTACGTAGACCAAATAATATAATGCAAGCAAAAGTTCCTAGATTCATGGAGATATAGAACAGCATATAAGTTATCATGCTCGCATATCCACCATTTGAGTCTCCAACAATTATTCCAATAATTACATATCCGATTTGACCTATGGACGAATATGCAAGCATACGTTTCATGCTTGTTTGAGTAATAGCAATGAGATTCCCCAATATCATGCTAAGAATAGCTAGGATTTCCAGAAGAAGATGCCATTCATTTGATGAGAAATAAAAAGGAATATCGAAAATTCGAGTGGCTAAAGCTGAAGCAGCTACTTTCGAAGTAACAGAAAGAAAAGCAACGACTGGAGTGGGAGAGTCAGAGTCGAAAAGAGGATTCCTCACTTCTTTCTCTCATTCAAAACCGTGCATGAGACTTTCATCTCGCACGGCTCCTAAGTGATAAAAGTAAAGAAGAACTCATCTTCTTTCTTTTTTGATTACTTTCCTCGCGTATGTATAAGATCGAATCCTTTCTAAAACGGATTACTAATCCTTAACTTTTCGAGGAATCCTTCATCAGTGGTTGTGAATGACTGATTTTTCTCAATCGTTTCGACCTTGGTTCCGTAGGAGCACGTCCGAAAGATTGAGAAATGGAACCATCTGATTTGATTCGTTCTCAATAGCCATGAGATGATCATCTTAGGGTGATCCTTTTGTCGACGGATGCTCCTATTACACTCGTAGTCTCTGAAGGATGAGAACCAACTATGTAGCATCTACATCGAGAATTCAAGTCTTTCTTGTATACGTCATTAGTCCGATCCTTTGTAGGAACTACCCGTAATAACAAACTTGCAAAATGGATCCGTTTATCATAAAGAGATTCGTTGTTCCTGACCCTGCTTCACATTAATTGTTATTTGAACAAGTCAAAGTTCTGTCTTGGTCTGCGTGGGGATAGCATTTCTCTTCTGCATGTCCATGGAGTTTTGAAAAATCCAAACATCTCAGAGATAGATAGAGAGGTAGGAATTTCTCAAACGAACCGCACTCCTTCGTATACGTCAGGAGTCCATTGATGAGAAGGGGCTAGGGAAAGCTTGAACCCAATTCCTACAGTGATGAATATAAGCGCAATTGAAATTCCTGGGGAGTTATACATTTGTGTATTGATAAGACCATTCACTATTTCTTGAAGCTCAATCTCTCCCCCGGATGAACCATATAGCCAAGAGAAACCATGAACCAGAATAGAAGAGCTTGCCCCACCCATGAGTAAATATTTCATAGTAGCTTCATTAGATCGTACATCTTTCTTGGTATATCCAGATAATAGGTAGGAGCATAAACTGAAACATTCTGGAGCTACAAAGATAGTTATTAAATCGTTAGCACCACATAAAAACATTCCTCCTAGAGTAGCTGTTAATACGAATAACAGAAACTCTGTTATAGCCATTTCTGTACATTCAATGTACTCTACGGATAGAGGAATACAGAGAGTTGAACATAGTAAAATAAGAAATTGAAAGATTTCGTTGAAATTGTTCGTTTGGAAATTTCCTGAAAAGCTAATCATAGGTTCTTCTCTCCATCGGAACAATAGGGCCGTTATGCTCATTACGAAACTTGTTGACGAGATGAAATATAACCAAGGTATATCTTTTTGATCAGAGGTTGAATCGATCATCAGAAGAAGGATTAGGCCAAAAATTAGGATACATTCTGGGAAAATAAAACTTCCATCGAAGAGAAGCAAATGAAAGGCTTTCATAAAAATTCTCGTAGAATCGAGAATGAAATTTTCATTCTGTACATGCCAGATCATGAATTAGTAACTGCATCCAATCTCCAAAAAAAAACCAATTTTTTTTTTTTTGAATGGAATATTTACGGAATCCCCATGAATAGGTTAAAACCTTATTCCATGGTATTTACATGAGATTGCTCTTTCTTATTCTTAAGCAAGTCCCCGAGAGGGCTTAGTTGATCCATGATTTATGTTTCGTCTTTTCTTTCCTTTTCGTTTGTTTCGAGAAAGAGATCGATCAATTCCGATTTTTTCTTTTTCTATTGATTCTTTTCGGATCGAGATGTATGGATCCACGGATCTATGTGTCTATATAGATCCTGTTCATGGATTAACGAAAATGTGCAAACGCTCTATTTGCCTCTGCCATTCTATGAGTCTCTTCCTTTTTGCGTATGGCATCGCCACTCCCTTTGGCAGCATCCACTAATTCGGAACTTAATTTGAAAGCCATATTTCGACCCGGACGTTTTCGGGATGCCCCTAATAACCAACGAATGGCAAGTGCTTTTCCTTGCGTGGATCCTATTTCAATGGGAACTTGATGAGTTGATCCGCCTACACGTCTTGCTTTTACTGCTATATCGGGAGTTACTCCACGTATTGCTTGACGTAAAACAGATAGTGGATTTGTTTCTGTCTTTTGTTGAATCTTTTTCAAGGCTCGATAGATAATTTGATAAGCCAATGATTTTTTTCCGTGTTTCAGAATACGGTTAACCAACATGTTAACTAATCGATTACGATAAATTGGATCGGATTTTGCAGTTTTTTCTTCTGCAGTACCTCGACGTGACATGAGCGTGAAAGGGGTTCAAGAATCTGTTTTCTTTTTATAAGGGCTCAAATCTTTTATTTTGGCTTTTTGACCCCATATTGTAGGGTGGATCTCGAAAGATATGAAAGATCTCCCTCCAAACCGTACATACGACTTTCATCGAATACGGCTTTCCACAGAATTCTATATGTATCTATGAAATCGAGTATGGAATTCTGTTTACTCACTTTTAAATTGAGTATCCGTTTCCCTCCTTTTCCTGCTAGGATTGGAAATCCTGTATTTTACATATCCATACGATTGAGTCCTTGGGTTTCCGAAATAGTGTAAAAATAAGTGCTTCGAATCATTGCTATTTGACCCGGACCTGTTCTAAAAAAGTCGAGGCATTTCGAATTGTTTGTTGACACGGACAAAGTCAGGGAAAACCTCTGAAATTATTTCAATATTGAACCTTGGACATATAAGAGTTCCGAATTGAATCTCTTTTGAAAGAAGATCTTTTGTCTCATGGTAGCCTGCTTCAGTCCCCTTACGAAACTTTCGTTATTGGGTTAGCCATACACTTCACATGTTTCTAGCGATTCACATGGCATCATCAAATGATACAAGTCTTGGATAAGAATCTACAACGCACTAGAACGCCCTTGTTGACGATCCTTTACTCCGACAGCATCTAGGGTTCCTCGAACAATGTGATATCTCACACCGGGTAAATCCTTAACCCTTCCCCCTCTTACTAAGACTACAGAATGTTCTTGTAAATTATGGCCAATACCAGGTATATAAGCAGTGATTTCAAATCCCGAGGTTAATCGTACTCTGGCAACTTTACGTAAGGCAGAGTTTGGTTTTTTGGGGGTGATAGTGGAAAAGTTGACAGATAAGTCACCCTTACTGCCACTCTACAGAACCGGACATGAGATTTTCACCTCATACGGCTCCTCGTTCAATTCTTTCGAAGTCATTGGGTCCCTTTCCTCGTTCGCGAATCTCCTCCGTCCCGAAGAGTAACTAGGATAAACTCGGTCACGTTTTCATGTTCCAATTGAACACTTTCTATTTTTGATTATTCTCAAAGGATAAGATTATTCTTTTTACCAAACATCTGCGGGTCCAATCACACGATCTTATAATAAGAACAAGAGATCTTTCTCGATCAATCTCTTTGCCCCTCATTCTTCGAGAATCAGAAAGAGACTTTTTCAAGTTTGAATTTGTTCATTTGTAATCTGGGTTCTTCTACTTCATTTTTATTTACTTATTATTTCTTTATTTTCCCTCTCTTTTCTTTATTTGATTTCTTTTTTGATTTTATTCCCTTCCATCATTCTTAAGTCCCATAGGTTTGATCCTATAGAATCTGACCCATGTTCTCATTGAGCGAAGGGTACGAAATAAATTCAATCATATTTTTTTTTGATCAAAAAAAAATCACTATGTGAAATCTTCGTTTTTTTTTTTCTCTTTCTCTATCGCTTTCCCATAAGTACAGCACTTGTTGAATCGATAGAGAACCTTTTCTTCTGTATCGATATGAATCCATTATGAATCGATATTATTACATTCCAATTCCTTACCAATATCCCTCAAGGAAAATCCCGAATTGGATCCCAAATTGACGGGTTAGTGTGAGCTTATCCATGCGGTTATGCACTCTTCGAATAGGAATTCATTTTCTGAAAGATCCTGGCTTTCGTGCTTTGGCGGGTCTCCGAGATCCTTTCGACGACCTATGTTGTGTTGAAGGGATATCTAGATGATCCGATCGATTGCGTAAAGCCCGCAGTAGCAACGGAACCGGGGAAAGTATACATAAGTATACAGAAAAGACAGTTCTTTTCTATTATATTAGGATTTTCTATTCTATTAGATTAGTGTTAGTTAGTGATCTTGGCGCAGTGAGTCCTTTCTTCTCGGTCCACAGAGACAAAATGTAGGACTGGTGCCAACAGTTAATCACGGAAGAAAGGAGGCTCAGCGGGAAGAGGATTGTACCATAGAAGCAAGGAGGTCAACCTCTTTCCAATAGATAACATGAATTCTGGCAATGCAATGTAGTTGGGCTTTCATGTTGATCCGAATGAATCATCTTTTTCGCGGAGTGAAATCTTTGCCTGCTAGGCAAGATTATAGGATAGCAAGTTACAAATTCTGTTTCGGTAGGACATGTATTTCTATTACTATGAAATTCATAAATGAAATAGTTAATCGTGGGGTTACCATTCTCTCTTTTTTTTTTTATCTCGCACGTGTTCCTAAGAAAAGGGAATTTGTTAATTTTTCGGGGTCTTAAAGGGGCGTGGAAACACATAAGAACTCTTGAATGGAAATGGAAAAGAGATGTAACTCCAGTTCCTTTGGAAATAGGAAGATCTTTGGCGCAAGAATAAAGGATTAATCCGTATCATCTTGACTTGGTTCTGATTTCTCTATTTTTTGAAGTTTAAGAAAAGAATACCGTTTCTCCTACCCGTATCGAATAGAACATGCTGAGTAAAATCTTCTTCATGTAAAACCGGCTTGATTTAGATCGGGAGAATCGTACGGTTTTATGAAACCATGTGCTATGGCTCGAATCCGTAGTCAATCCTATTTCCGATAGGAGTAGTTGACAATTGAATCCAACTTTTTCCATTATTTTCATTTCATACCCGTAATAGTGCGAAAGGAAAGCCCGGCTCCAATCCAAGTTGTTCAAGAATAGTGGCCTTGAGTTTCTCGACCCTTTGACTTAGGATTAGTCAGTTCTATTTCTTGATGGGGGAAGGGATATAACTCAGCGGTAGAGTGTCACCTTGACGTGGTGGAAGTCATCAGTTCGAGCCTGATTATCCCTAAACCCAATGAATGTGAGTTTTTCTATTTTGACTTGCTCCCTCGCTGTGATCGAATAAGAATGGATAAGAGGCTCGTGGGATTGACGTGAGGGGGTAGGGGTAGCTATATTTCTGGGAGCGAACTCCATGCGAATATGAAGCGCATGGATACAAGTTATGACTTGGAATGAAAGACAATTCCGAATCAGCTTTGTCTACGAAGAAGGAAGCTATAAGTAATGCAACTATGAATCTCATGGAGAGTTCGATCCTGGCTCAGGATGAACGCTGGCGGCATGCTTAACACATGCAAGTCGGACGGGAAGTGGTGTTTCCAGTGGCGGACGGGTGAGTAACGCGTAAGAACCTGCCCTTGGGAGGGGAACAACAGCTGGAAACGGCTGCTAATACCCCGTAGGCTGAGGAGCAAAAGGAGGAATCCGCCCGAGGAGGGGCTCGCGTCTGATTAGCTAGTTGGTGAGGCAATAGCTTACCAAGGCGATGATCAGTAGCTGGTCCGAGAGGATGATCAGCCACACTGGGACTGAGACACGGCCCAGACTCCTACGGGAGGCAGCAGTGGGGAATTTTCCGCAATGGGCGAAAGCCTGACGGAGCAATGCCGCGTGGAGGTAGAAGGCCTACGGGTCCTGAACTTCTTTTCCCAGAGAAGAAGCAATGACGGTATCTGGGGAATAAGCATCGGCTAACTCTGTGCCAGCAGCCGCGGTAATACAGAGGATGCAAGCGTTATCCGGAATGATTGGGCGTAAAGCGTCTGTAGGTGGCTTTTTAAGTCCGCCGTCAAATCCCAGGGCTCAACCCTGGACAGGCGGTGGAAACTACCAAGCTTGAGTACGGTAGGGGCAGAGGGAATTTCCGGTGGAGCGGTGAAATGCGTAGAGATCGGAAAGAACACCAACGGCGAAAGCACTCTGCTGGGCCGACACTGACACTGAGAGACGAAAGCTAGGGGAGCGAATGGGATTAGATACCCCAGTAGTCCTAGCCGTAAACGATGGATACTAGGCGCTGTGCGTATCGACCCGTGCAGTGCTGTAGCTAACGCGTTAAGTATCCCGCCTGGGGAGTACGTTCGCAAGAATGAAACTCAAAGGAATTGACGGGGGCCCGCACAAGCGGTGGAGCATGTGGTTTAATTCGATGCAAAGCGAAGAACCTTACCAGGGCTTGACATGCCGCGAATCCTCTTGAAAGAGAGGGGTGCCTTCGGGAACGCGGACACAGGTGGTGCATGGCTGTCGTCAGCTCGTGCCGTAAGGTGTTGGGTTAAGTCCCGCAACGAGCGCAACCCTCGTGTTTAGTTGCCACCGTTGAGTTTGGAACCCTGAACAGACTGCCGGTGATAAGCCGGAGGAAGGTGAGGATGACGTCAAGTCATCATGCCCCTTATGCCCTGGGCGACACACGTGCTACAATGGCCGGGACAAAGGGTCGCGATCCCGCGAGGGTGAGCTAACTCCAAAAACCCGTCCTCAGTTCGGATTGCAGGCTGCAACTCGCCTGCATGAAGCCGGAATCGCTAGTAATCGCCGGTCAGCCATACGGCGGTGAATTCGTTCCCGGGCCTTGTACACACCGCCCGTCACACTATGGGAGCTGGCCATGCCCGAAGTCGTTACCTTAACCGCAAGGAGGGGGGTGCCGAAGGCAGGGCTAGTGACTGGAGTGAAGTCGTAACAAGGTAGCCGTACTGGAAGGTGCGGCTGGATCACCTCCTTTTCAGGGAGAGCTAATGCTTCTTGGGTATTTAGGTTTGACACAGCTTCAAACCCAAAGCCCATGAGCTTATTATCCTAGGTCGGAACAAGTTGATAGGATCCCCTTTTACGCCCCCATGTCCCTCTCGTGTGGCGGCAGGGGGGCGTAAAAAGGAAAGAGAGGGATGGGGTTTCTCTCGCTTTTGGCTTGGCATAGCGGGCCCCCAGCAGGAGGCCCGCACGACGGGCTATTAGCTCAGTGGTAGAGCGCGCCCCTGATAATTGCGTCGTTGTGCCTGGGCTGTGAGGGCTCTCAGCCACATGGATAGTTCAATGTGCTCATCAGCGCCTGACCCTGAGATGTGGATCATCCAAGGCACATTAGCATGGCGTACTCCTCCTGTTCGAACCGGGGTTTGAAACCAAACTTCTCCTCAGGAGGATAGATGGGGCGATTCAGGTGAGATCCAATGTAGATCCAACTTTCTATTCACTCGTGGGATCCGGGCGGTCCGGAGGGGACCACCACGGCTCCTCTCTTCTCGAGAATCCATACATCCCTTATCAGTGTATGGACAGCTATCTCTCGAGCGCAGGTTTAGGTTCGGCCTCAATGGGAAAATAAAATGGAGCACCTAACAACGTATCTTCACAGACCAAGAACTACGAGATCACCCCTTTCATTCTGGGGTGACGGAGGGATCGTACCGTTCGAGCCTTTTTTTCATGCTTTTCCCAGGGGTCTGGAGAAAGCTGCAATCAATAGGATTTTCCTAATCCTCCCTTCCCGAAAGGAAGAACGTGAAATTCTTTTTCCTTTCCGCCTCGAAATGGGAGCAGGTTTGAAAAAGGATCTTAGAGTGTCTAGGGTTAGGCCAGTAGGGTCTCTTAACGCCCTCTTTTTTCTTCTCATCGAAGTTATTTCACAAATACTTCCTATGGTAAGGAAGAGGGGGGGAACAAGCACACTTGGAGAGCGCAGTACAACGGAGAGTTGTATGCTGCGTTCGGGAAGGATGAATCGCTCCCGAAAAGGAATCTATTGATTCTCTCCCAATTGGTTGGACCATAGGTGCGATGATTTACTTCACGGGCGAGGTCTCTGGTTCAAATCCAGGATGGCCCAGCTGCGCCAAGGAAAAGAATATAAGAAGGATCTGACTCCTTCATGCATGCTCCACTTGGCTCGGGGGGATATAGCTCAGTTGGTAGAGCTCCGCTCTTGCAATTGGGTCGTTGCGATTACGGGTTGGGTGTCTAATTGTCCAGGCGGTAATGATAGTATCTTGTACCTGAACCGGTGGCTCACTTTTTCTAAGTAATGGGGAAAAGGACCGAAACATGCCACTGAAAGACTCTACTGAGACAAAGATGGGCTGTCAAGAACGTAGAGGAGGTAGGATGGTCAGTTGGTCAGATCTAGTATGGATCGTACATGGACGGTAGTTGGAGTCGGCGGCTCTCCTAGGGTTCCCTCGTCTGGGATTGATCCCTGGGGAAGAGGATCAAGTTGGCCCTTGCGAACAGCTTGATGCACTATCTCCCTTCAACCCTTTGAGCGAAATGCGGCAAAAGGAAGGAAAATCCATGGACCGACCCCATCGTCTCCACCCCGTAGGAACTACGAGATCACCCCAAGGACGCCTTCGGTATCCAGGGGTCGCGGACCGACCATAGAACCCTGTTCAATAAGTGGAATGCATTAGCTGTCCGCTCGCAGGTTGGGCAGTAAGGGTCGGAGAAGGGCAATCACTCATTCTTAAAACCAGCATTCGAAAGAGTTGGGGCGGAAAAGGGGGGAAAGCTCTCCGTTCCTGGTTCTCCTGTAGCTGGATCCTCTCGAACCACAAGAATCCTGAGTTGGAATGGGATTCCAACTCATCACCTTTTGAGATTTTGAGAAGAGTTGCTCTTTGGAGAGCACAGTACGATGAAAGTTGTAAGCTGTGTTCGGGGGGGAGTTCTTGTCTATCGTTGGCCTCTATGGTAGAATCAGTCAGGGGCCTGATAGGCGGTGGTTTACCCTGTGGCGGATGTCAGCGGTTCGAGTCCGCTTATCTCCAACTCGTGAACTTAGCCGATACAAAGCTATATGATAGCACCCAATTTTTCCGATTCGGCAGTTCGATCTATTATTTTTCATTCATGGACGTTGATAAGATCTTTCCATTTAGCAGCACCTTAGGATGGCATAGCCTTAAAGTTAAGAGCGAGGTTCAAACGAGGAAAGGCTTACGGTGGATACCTAGGCACCCAGAGACGAGGAAGGGCGTAGTAAGCGACGAAATGCTTCGGGGAGTTGAAAATAAGCGTAGATCCGGAGATTCCCGAATAGGTTAACCTTTTGAACTGCTGCTGAATCCATGGGCAGGCAAGAGACAACCTGGCGAACTGAAACATCTTAGTAGCCAGAGGAAAAGAAAGCAAAAGCGATTCCCGTAGTAGCGGCGAGCGAAATGGGAGCAGCCTAAACCGTGAAAACGGGGTTGTGGGAGAGCAATAAAAGCGTCGTGCTGCTAGGCGAAGCGGTGGAGTGCCGCACCCTAGATGGCGAGAGTCCAGTAGCCGAAAGCATCACTAGCTTATGCTCTGACCCGAGTAGCATGGGGCACGTGGAATCCCGTGTGAATCAGCAAGGACCACCTTGCAAGGCTAAATACTCCTGGGTGACCGATAGCGAAGTAGTACCGTGAGGGAAGGGTGAAAAGAACCCCCATCGGGGAGTGAAATAGAACATGAAACCGTAAGCTCCCAAGCAGTGGGAGGAGCCCTGGGCTCTGACCGCGTGCCTGTTGAAGAATGAGCCGGCGACTCATAGGCAGTGGCTTGGTTAAGGGAACCCACCGGAGCCGTAGCGAAAGCGAGTCTTCATAGGGCAATTGTCACTGCTTATGGACCCGAACCTGGGTGATCTATCTATGACCAGGATGAAGCTTGGGTGAAACTAAGTGGAGGTCCGAACCGACTGATGTTGAAAAATCAGCGGATGAGTTGTGGTTAGGGGTGAAATGCCACTCGAACCCAGAGCTAGCTGGTTCTCCCCGAAATGCGTTGAGGCGCAGCAGTTGACTGGACATCTAGGGGTAAAGCACTGTTTCGGTGCGGGCCGCGAGAGCGGTACCAAATCGAGGCAAACTCTGAATACTAGATATGACCTCAAAATAACTGGGGTCAAGGTCGGCCAGTGAGACGGTGGGGGATAAGCTTCATCGTCGAGAGGGAAACAGCCCGGATCACCAGCTAAGGCCCCTAAATGACCGCTCAGTGATAAAGGAGGTAGGGGTGCAGAGACAGCCAGGAGGTTTGCCTAGAAGCAGCCACCCTTGAAAGAGTGCGTAATAGCTCACTGATCGAGCGCTCTTGCGCCGAAGATGAACGGGGCTAAGCGATCTGCCGAAGCTGTGGGATGTCAAAATGCATCGGTAGGGGAGCGTTCCGCCTTAGGGGGAAGCAACCGCGCGAGCGGCGGTGGACGAAGCGGAAGCGAGAATGTCGGCTTGAGTAACGCAAACATTGGTGAGAATCCAATGCCCCGAAAACCCAAGGGTTCCTCCGCAAGGTTCGTCCACGGAGGGTGAGTCAGGGCCTAAGATCAGGCCGAAAGGCGTAGTCGATGGACAACAGGTGAATATTCCTGTACTACCCCTTGTTGGTCCCGAGGGACGGAGGAGGCTAGGTTAGCCGAAAGATGGTTATCGGTTCAAGAACGCAAGGTGTCCCTGTTTTTTCAGGGTAAGAAGGGGTAGAGAAAATGCCCCGAGCCAATGTTCGAGTACCAGGCGCTACGGCGCTGAAGTAACCCATGCTATACTCCCAGGAAAAGCTCGAACGACCTTCAACAAAAGGGTACCTGTACCCGAAACCGACACAGGTGGGTAGGTAGAGAATACCTAGGGGCGCGAGACAACTCTCTCTAAGGAACTCGGCAAAATAGCCCCGTAACTTCGGGAGAAGGGGTGCCTCCTCACAAAGGGGGTCGCAGTGACCAGGCCCGGGCGACTGTTTACCAAAAACACAGGTCTCCGCAAAGTCGTAAGACCATGTATGGGGGCTGACGCCTGCCCAGTGCCGGAAGGTCAAGGAAGTTGGTGACCTGATGACAGGGGAGCCGGCGACCGAAGCCCCGGTGAACGGCGGCCGTAACTATAACGGTCCTAAGGTAGCGAAATTCCTTGTCGGGTAAGTTCCGACCCGCACGAAAGGCGTAACGATCTGGGCACTGTCTCGGAGAGAGGCTCGGTGAAATAGACATGTCTGTGAAGATGCGGACTACCTGCACCTGGACAGAAAGACCCTATGAAGCTTCACTGTTCCCTGGGATTGGCTTTGGGCTTTTCCTGCGCAGCTTAGGTGGAAGGCGAAGAAGGCCTCCTTCCGGGGGGGCCCGAGCCATCAGTGAGATACCACTCTGGAAGAGCTAGAATTCTAACCTTGTGTCAGGACCTACGGGCCAAGGGACAGTCTCAGGTAGACAGTTTCTATGGGGCGTAGGCCTCCCAAAAGGTAACGGAGGCGTGCAAAGGTTTCCTCGGGCCGGACGGAGATTGGCCCTCGAGTGCAAAGGCAGAAGGGAGCTTGACTGCAAGACCCACCCGTCGAGCAGGGACGAAAGTCGGCCTTAGTGATCCGACGGTGCCGAGTGGAAGGGCCGTCGCTCAACGGATAAAAGTTACTCTAGGGATAACAGGCTGATCTTCCCCAAGAGCTCACATCGACGGGAAGGTTTGGCACCTCGATGTCGGCTCTTCGCCACCTGGGGCTGTAGTATGTTCCAAGGGTTGGGCTGTTCGCCCATTAAAGCGGTACGTGAGCTGGGTTCAGAACGTCGTGAGACAGTTCGGTCCATATCCGGTGTGGGCGTTAGAGCATTGAGAGGACCTTTCCCTAGTACGAGAGGACCGGGAAGGACGCACCTCTGGTGTACCAGTTATCGTGCCCACGGTAAACGCTGGGTAGCCAAGTGCGGAGCGGATAACTGCTGAAAGCATCTAAGTAGTAAGCCCACCCCAAGATGAGTGCTCTCCTATTCCGACTTCCCCAGAGCCTCCGGTAGCACAGCCGAGACAGCAACGGGTTCTCCGCCCCTGCGGGGATGGAGTGACAGAAGTTTTGAGAATTCAAGAGAAGGTCACGGCGAGACGAGCCGTTTATCATTACGATAGGTGTCAAGTGGAAGTGCAGTGATGTATGCAGCTGAGGCATCCTAACAGACCGGTAGACTTGAACCTTGTTCCTACATGACCTGATCAATTCGATCAGGCACTCGCCATCTATTTTCATAGTTCAACTCTTTGACAACACGAAAAAACCATTGTTCAACTCTTTGACAACATGAAAAAACCAAAAATTCTGCCCTTCTATCCAAAGGATGGAGGGGCGGAGGCCTTTGGTGTCCACTCCAGTCAAGAATTGGAGCCTCACAATCACTAGCCAATATGCTTTTCTCGCATGCCTTTCTTCGTTCATGGTTCGATATTCTGGTGTCCTAGGCGTAGAGGAACAACACCAATCCATCCCGAACTTGGTGGTTAAACTCTACTGCGGTGACGATACTGTAGGGGAGGTCCTGCGGAAAAATAGCTCGACGCCAGGATGATGAAAAGCTTAACACCTCTCATTCTTATTACTTTTTCATATTGAAAAAAAATGAAAAATGAAAAGGTTGTCTTATTCAAAACCCCAATTATGAAATCCCTTCTATCCCACTTCACACCCCGGAACGCACCGTTCTTATAGAGAGAAAGGCACTTTCACATCTTCTTAACCCGAAATGGCTGGGGAGAGGAAAGGTTCCTTTTTTTGTAGGGTACTCCTGGGAACAGATCCAGTGGAGACGGGGTGGGGCTTGTAGCTCAGAGGATTAGAGCACGTGGCTACGAACCACGGTGTCGGGGGTTCGAATCCCTCCTCGCCCACAACCGGCCCAAAAGGGAAGGACCTTTCCCTCCGGGGGGTAGGAAAATCATGCTCGGGATAGCGGACTCAAAGCTATGGAACTTGGTTGGGGATGGGTCTTTTGTCGAAATAGAGTGGAGTGGCCTTCTTTTTTATTTGAATTTAGATATATATATCTATTATATCTATCGCTTTTTTTTTACATATAGTATGATTACCGGCCGAATCAGCATATTTTTCGAAGCCCCGTAACTCTTCCTCAGCCAGGCTTGGGCAGAATAGCAGAGCAAGTACAAGTATTAGTAGCATAGAAAAAATGCGTTCCTCATCATTAAGTCATTAAATGAATATGTTTGCGCGCGGTAATTGTGAACTCTCGGGAGAATCGATGACTGCATCAAAGATGCACTTGTTAGTACACCTGCAAATTCTGAATTGGCTAGTTGTAAATAGCCCCAGGACTATGGAATAAAGGATTATCCCGGACCTACACCGAGGTATTGACGGTGATTCTCAAATATCACAGAACAGAATGTGATACGATGAGATAGAATGCAATAGAAACAAAGACACAGGGAACGGGTTACCTACTCTTACATTCTGAATTCTTGAATTCGGAATGAATCAAATCTCCCCAAGTAGGATTCGAACCTACGACCAATCAGTTAACAGCCGACCGCTCTACCACTGAGCTACTGAGGAACAACGGGAGATTAGATCTCCTAGAGTTCAATTCCCGTTCTCAACCCATGACCAATATGAACTCGAAGTTTCCTTCGTAACCCCCGGAACTTCTTCGTAGTGGCTCCGTTCCATGCCTCATTTCATAGGGAACCTCAAAGCGGCTCTATTTCATTATATTCCATCCATATCCCAATTCCATTCATTTAATATCCCTTTGGTGTCATTGACATAAGAGATGTCGTTTCTAGTCTATCTCTTTCTATTTCTATATATGGAAAGTTGCAAAATCATCATATAATAATCCAGAAATTGAAATAGAAAAGAAAAAAGGGAGGTTTGTGATGGTTTTTCAATCTTTTATACTAGGTAATCTAGTATCCTTATGCATGAAGATAATCAATTCGGTCGTTGTGGTCGGACTCTATTATGGATTTCTGACCACATTCTCCATAGGGCCCTCTTATCTCTTCCTTCTCCGAGCTCGGGTTATGGACGAAGGAGAAGAAGGAACCGAGAAGAAAGTATCAGCAACAACTGGTTTTATTGCGGGACAGCTCATGATGTTCATATCGATCTATTATGCGCCTCTGCATTTAGCATTGGGTAGACCTCATACAATAACTGTCCTAGCTCTACCGTATCTTTTGTTTCATTTCTTCTGGAACAATCACAAACACTTTTTTGATTATGGATCTACTACCAGAAATGAAATGCGTAATCTTCGCATTCAATGTGTATTCCTGAATAATTTCATTTTTCAATTATTCAACCATTTCATTTTACCAAGTTCAATGTTAGCCAGATTAGTCAACATTTATATGTTTCGATGCAACAACAAGATGTTATTTGTAACAAGTAGTTTTGTTGGTTGGTTAATTGGTCACATTTTATTCATGAAATGGGTTGGATTGGTATTAGTCTGGATACAGCAAAATAATTCTATTAGGTCTAATGTACTTATTAGATCTAATAAGTATAAGTTCCTTGTGTCAGAATTGAGAAATTCTATGACTCGAATCTTTAGTATTATCTTATTTATTACCTGTGTCTACTATTTAGGCAGAATCCCATCACCCATTTTTACTAAGAAACTAAAAGGAACCTCAGAAACGGGTGGGACTAAACAGGACCAAGAGGTATCCACCGAAGAAGCTCCTTTTCCTTCTCTTTTTTCGGAAGAAAGGGAGGATCTGGACAAAATCGATGAAATGGAAGAAATCGGAGTGAATGGAAAAGACAAAATTAATAAGGATGATGAATTCCACGTTCGAACATACTATAACTATAAAACAGTTTCTGAAAATCGAGATGGAAATAAAGAAAATTCTAATTTAGAATTTTTCAAAATAAAAAAAAAAGAGGATCGTTAAAAAAATCAATACATAGCACAAATACAAGAACAGATAAGAAGAGATGCGACTTCCACCTATATATTTTGTTACTTCTCCTACAAAGAAACTTGTAATACCTACTCCATTTGTAATTCCATCAATGATTCGTTTATCAAAAAAATTCGTTTGTTTTGCTAATTTTCTTATACTTTCAGTTAAAGATTTTTTAAAAAAAGTATCTATGTAACCACGATTATATGACCAATTATATACAAAATTTATTGGTTTTTCCCACCTAATTCTTTTAGAACTCCACTTTTGAAATGAATTAAGTAAAGTTAAATTTAATCTAGATGAATAAAAAGGCTTATATAAACAGTATGCTATAAATATTCCAAACAAAGCTATACTGACTGAAAAAATTGCATTTTTCAAAAATTCATACCAATCTACAAAATTTTCTGAATTGGTATGCAAAAGGTTTATCGACGGCGTTAATAATTTTGATAATATATCAAAGTCTATTCCTTCTTGATTGAAAGGAATTCCTATGGCTCCAATAAACAAAGTAAATAAAAGCAATACAAGCATAGGAAATAGAATAGTATTGTCTGATTCATGGGGATAATAGAAAGTTCTTGTATTAAGTCCAAAATTTTCAACAGTAATAAAAGTTTGATTTCTTACATTATTACTAATTTTATATGTTTTATTGCCAAAAAAAGAAGCTCTTTTCGTATTATTCATTGTTAATAATGGTACTAACCCAAAATTCCTATTAAGTTTTTTATCTTCTTCTTTACCCCATAAAGAAATTGAATAGAAGGAGCTACTTTTTTTTCCACTATAATTTATAAAATAAGTGTTTAAATGGCCTTCAAAAGTAAGTAAATAAATCCGAAACATATAAAATGCGGTTAATCCCGCTGTTGAACAAGCTATTATTGCAAAAATTGGCGAAAATAACAAACTATCATTAAGAATTTCATCTTTAGACCAAAAACAAGCAAGGGGGGGAATACCACAAAGTGAGAGTGTTCCTACTAAAAAGGCAGTTTTTGTAATCGGCACATGTTTTGTCAAACCACCCATAAGAATCATATTCTGACTTTTATCAGGAGAATAGCCAACTATAGCTTCCATTGAATGAATAATGGATCCAGATCCTAAAAACAACAAAGCTTTCGAATAAGCATGAGTAATCAAATGAAATAAAGCGGATCTATAAGACCCCATACCTAGAGCTAACATCATATAACCCAGTTGAGACATTGTAGAATAGGCTAAACCTCTCTTAATATCTTTTTGAGCAAGAGCTAAAGTGGCTCCTAAGAGTACTGTTATTATACCTATCAAAGATATTATATACATTATAGAAGGGATAACTATAAAAAGAGGAAGAAGACGAGCTACAAGAAAAATTCCCGCTGCTACCATAGTAGCAGCATGTATAAGAGCCGAAATAGGAGTAGGGCCCTCCATGGCATCCGGCAACCATACATGAAGAGGAAATTGTGCAGATTTAGCAATAGGACCCACAAATAATAGAAATGCACACAAAGTAAGGAATAAGAGATTTATTCTATTATTTAATATTAAATTATTGAATATTTCGAACAAATCTTGAAATTCGAAACTGCCAGTTATCCAATAAAGACCTAAAATTCCTAATAATAAACCAAAATCCCCTACACGATTGGTTACAAAAGCTTTTTGACAGGCATTCGCTGCAATAGGTCGTGTGAACCAAAAACCTATTAATAAATACGAACACATTCCAACTAATTCCCAAAAAAAATAAACTTGGATCAAATTAGAACTAGTAACTAATCCTAACATTGAAGTATTAAAAAAACCCATATAAGCAAAAAACCTCAGATATCCTTGATCATGAGACATATAATTATCACTATAAATCAGAACCAAAATTCCAACAGTTGTAATTAATATTGACATAATAGAAGTAAGTGGATCAATAAAGTAACCGAACTCAAAAGAAAATTCATTATTTATGGTCCAAGACCATACATTTTGATGAATGCAACTTAGAAAAATTTGTTGAATAGATAGATAGAGCGAAAAGATCATAACTATACTTAACAAAAAAATACTCAGAAACGTCCACATACGTCGAAGGTTTTTTGTTGCTGTCGGAAAAAGTAGAAGTCCAGCTCCGAGTAAAATAGGTACTGGAAGTGGAATGAAAGGGATGATCCATGAATATTGATATGTATGTTCCATAAAATAAAAAACCCTTTTTATTTTATTCTTAAATTTATTATTTCTTATTCACTGGTTTGTATATATATATATTTTTTTCAAAGGGGATAATAAAAAAGCGCATTTTTTCAAACTTAAATAGAAATTTTTTCGAATTAGTATAATCCTTCATAAACCTTTGAAAAGAAATATATTCAAATCAAAAAATTAGAAGTTATTAACTAATATTACTAAGTTACTGTAAAAAAAACGATTTGTCTTTTTTTTTTTTTACTACTAAAAAAAATTTTGATTTTATGCAGATACAGAAAAAGTGAATTATAATTCCGTATTACAATAATTTATATACATATATTAAGAATAGAACAAAGATTTACACGACAAAAAAATACTTAATATTAAGTATAAAAAAAAGTTATTTGGGTTTGATTATTTAGAATTATTATTGAATTATGGAATTTAGTGATTGTCTTCCAGTACACTAAGTGAGCTTTTTTTTTTCAAGAAATATTATTATAATCGATATTTTTTTTTACATATGAAGTGAAGAAATTTAATAAAATTTTTTCTAATATAATCTAAATCTATCAGTATAGATTAATTAAATGAGCACTCTTATACGGATTTAAAACGTTAAATACAAAAAATTTTTCAAGAAAAAGGGAAAAAATAGTTGGGTTTTAAACTTTTGAATGTCTGTTTTGTTTGAAAAATAATATATAATAAAATTTGAAAGAAAAAAATTACTCAATATGGAGTACGAAAGAATAGAATAATAAATGTCTTTGACATCCAATTATACCACTGAAAAACTTTTTTCATTTTTGAATGGCAGTTCCAAAAAAACGTACTTCTATCTCGAAAAAGCGTATTCGTAAAAAAATTTGGAAAAGGAAGGGATATTGGACATCGTTGAAAGCTTTTTCCTTAGGGAAATCGCTTTCTACAGGTAATTCAAAAAGTTTTTTTGTACAACAAAATAAATAAAAAACACTAGAATCATTAGAATTAGCCTAACGTAAAAACCAATTTTTTAGAATACATATAAATTAAAAAAATCTATAGGAACCAAAAAAAAAAAAAAAAAAGATAATATATATATATACGAAATATACGAAAGATTCCTATTGATTTTGTAAAAAAAAGGGGGGGTTATTACTTTCCCCATCAATAAAAAAATAAATAAAGATCTTGTATTTCCTCTTAACTAGGAAATACAAGATCTTTTAGCGAAATCAACAGGTTCTTTAAATTAATTTAAGTCAAAAATTTTTCACTTTATACCTTTAGGAATTATTATTTCTCTTAATTCTTATATTCTTTACTTGGAATCAAAGTTATAAAAGTATCTATCCACGATTAAGTGAATATTAGATACTAATAAGTAATAATATATGATATTTTTTTTAAGCGATCAAAAAATATTATGTTTGTACAATATAAAAAGATGCATGAAAATAGATATTTTGACAATTGTTGTTTTTCATTTTTCTTGAGCAACTTAGGCAAATTTTAGTTAAAATTTCTAAGGATTTTGGAGAAGTTTTAATTTTTAGAAAAAGCATTTTTTTAGTAATAAAATCAATTTTAAATTCCATTAAATTAGCTTCTTTATTTAAAATTTGAATCTCGACGATTGAGTAAAAACTTGTTAGTATTATTTTGAACAAGTTGCCGCTATGGTGAAATTGGTAGACACGCTGCTCTTAGGAAGCAGTGCTAGAGCATCTCGGTTCGAGTCCGAGTAGCGGCATAAGATCTTATAAAAGAGATATTATAAGTTTTATAATCAAATTAATACCCGACTTGTTTTCTAAAATCGGGTAAAACCTAGTATTAATTTATTAATTTTTAACAAATTTTTTATGATTTTTTCAATTTTAGAGCATATATTAACTCATATATCTTTTTCGGTCGTTTCAATTGTACTACTAATTTATTTTTTAACTTTATTAGTTAATTTAGATGAAATCATAGGATTTTTTGATTCATCAGATAAAGGAATCGTAATTACGTTTTTTGGTATAACAGGATTATTATTTACGCGTTGGATTTATTCAGGACATTTTCCATTAAGCAATTTATATGAATCATTAATTTTTCTTTCATGGGCTTTTGCAATTATTCATATAGTTTCCTATTTTAATAAAAAAAAAAAAAATCACTTAAACGCAATAACTGCGCCAAGTGCTATTTTTATTCAGGGTTTTGCTACTTCAGGTCTTTTAAACAACATGCCTCAGTCTGCAATATTAGTACCAGCTCTCCAGTCCCAGTGGTTAATGATGCACGTAAGTATGATGATATTAGGCTATGGCGCTCTGTTATGCGGATCATTATTATCAATAGCTCTTCTAGTCATTACATTTCGCAAGGTCGGATCTACTTTTTGGAAAAAGAATATGAAAAATAAAATGTTATTAAATGAATTATTTTCTTTTGATGTACTTTACTACATAAATGAAAGAAATTCTATTTTACTACAACAAAATATTAATTTTAGTTTTTCTAGAAATTATTATAGGTATCAATTGATTGAACAATTAGATTATTGGAGTTTTCGTATTATTAGTCTCGGATTTATCTTTTTAACCGTCGGCATTCTTTCAGGAGCTGTATGGGCTAATGAAACATGGGGTTCATATTGGAATTGGGATCCGAAAGAAACCTGGGCATTTATTACTTGGACCATCTTCGCAATTTATTTACATATTAAAACAAATAGGAATGCTCGAGGTATAAATTCTGCAATTGTGGCTTCGCTAGGTTTTCTTTTAATTTGGATATGCTATTTTGGCGTCAATCTTTTAGGAATAGGTTTACATAGTTATGGTTCATTTACATCGAATTAACTAAAACATTAACAAAAAAAGAAAAGAATCCAAATAAAAAAAATAGCATCTATATATAACTTCATCTAAGTTAAGAAATCTAATTTAGTTTTAGTAGTAAATCATCAAGAACCTTTTGAATCAAGTAGTACAATGATTCAAAAGGTTCTCACAATACAAAAAGCAAAGACTTCTTATTATAATTCAATTTAATGTTTTTTTTTATTTCCTGAAAACTATCCATAAAAATAATTAGATAAAATGGATTCGACCTTGTCACTTGCTAATGAGAGCACAAAATCAGGATAAATCCCAATACCAATTATGGGTAGAAGAATAGAGATTGAAAGAAATAACTCTCGGGGTCCAGAATCAAAAAAAGAAAAGTTTTTGGCATTAATTAACTTGTATCCATAGAACATTTGACGTGACATAGATAATAAATATATAGGAGTTAATATCATTCCAATTGCCATTACAAAAATAATTAAAATTTTTGAAATTAAGAAATATTTTTGGCTGGTAATTATTCCAAAAAAAACGATTAATTCGGCAACAAAACCACTCATGCCCGGTAATGCAAGGGAAGCCATCGATAAGATAGTGAACATTGTAAATATCTTTGGAATGGAGATAGCCATTCCACCCATTTCATCAAGATAAACAAGCCGGATTCTATCATAACTAGTTCCTGCCAAGAAAAAAAGTGCAGCGCCAATAAATCCATGAGAGATTATTTGTAAAATAGCTCCATTAAGCCCAGGATCCGTTATAGAACCAATACCTATAATTATAAAACCCATATGAGATACAGAAGAATAGGCTATTCTCTTTTTTAAATTACGTTGACCGGGAGATGTTGAAGCTGCATAAATTATTTGGATTGTACCGACTACCATCAACCAAGGAGAAAACATAGAATGAGCGTGAGGTAATAATTCCATATTGATTCGAACCAATCCATATGCTCCCATTTTTAATAAGATTCCAGCGAGAAGCATACAGGTACTGTAATGTGCCTCGCCGTGGGTGTCAGGTAACCAAGTATGTAAAGGTATAATCGGTGATTTGACGGCAAAAGCAATAAGAAATCCAATATAAAAGAGTATTTCGAGTGTGACCGGATAGGCTTGATTCCCTAATAGTTCTAAATTTAATGTTGGTTCGTTCGAACCATATAAACTTATACCTAAAACTCCTATTAATAAAAAAATAGAACTTCCTGCAGTGTATAAAATAAATTTTGTAGCTGAATACAAACGTTTCTTTCCACCCCACATGGATAAAAGGAGATAAACGGGAATTAATTCTAATTCCCACATGATGAAAAAAAGTAAAATATCCCGAGAAGAAAACGATCCTATTTGGCCGCTGTACATTGCTAACATCAGGAAATAGAATAATCGGGAATCCCGAGTAACTGGAAAAGCCGCTAAAGTAGCTAAAGTAGTAATAAATCCGGTCAGTAAAATCGTTCCTATAGAAAGTCCATCTATTCCCAGTCTCCAATAAAAATCAAAAAGATTGATCCATTTATAATCTTCGGACAGTTGAATTAATGGATCGTCCAGTTTAAAATTATAACAAAAAGCGTAAGTCGTTAGAAGAAGTTCTAAGATACAAATGCATATAGTATACCACTTATTAACTTTATTTCCCCTATGCGGGAGAAATAACATTAATGAACCGGCAGATATTGGAAAAACAACAATTATTGTTAACCAAGGAAAATCATTCGTGGTAAAGACAAGATACACCAGGTCCAAAGAACGCGTACTCAAAAAAATATATAAATAAAAAAATATAATTGAACTTTTTTGAGTACGAGTACTTGTCAATAAAAAAAATAAAATGTATTCCAAATTTATTCAAATCAGGTTTTCGGTAACGTATTAATAAGCTAGACCCATGCTTCGAGTTGTTTCATGCCATAAATAAACTCGAACGCTCAAAAAATCCGTTGGACAGGCAGATTCACATCTCTTACAACCAACACAATCCTCGGTTCTTGGGGCAGAAGCTATTTGCTTAGCTTTACATCCATCCCAAGGTATCATTTCTAATACGTCTGTAGGACATGCTCGGACACACTGAGTACATCCTATACAGGTATCATAAATTTTTACTGAATGTGACATAGGATCTATAGTTTTTTTAATGTCATAAATTTTCAATCTAGTAAACTTATAACTAAATGATATATTAAATTAAAATACTAGATGAAGCAATGATTTCTTTTAATAGAATTTTTTAATGAATTCTGGCTCAATTGGTAAAAAATGGGGCTAAAATACTTTGATTTCTTAAATTTTCACAAATTTAATCTAGTAAGTCATAACCTATCATATATGCAAATTTAAACCTATAATTTTTTGATTTATGCTACTTATTTAATAAGGTCGATTGGTTGATGCGAGTTGATTTTCTGTTACGATAAATTGACGAGACTATAGCTAATCCAATAGCTGCTTCAGCGGCTGCAATTGCTATAACAAAAATGCAGAAAATATCCCCTTTTAGTTGGGAATTATCAAAAAAATCAGCAAATGTTACGAGATTCATATTAACTGCATTGAGTATAAGTTCAAGGCACATAAGAGCCCTAACCATATTTCGACTCGTGATCAATCCATAAAGACCAATCAAAAATAAATAGGCACTCAAAACAAGTACATGTTCGAGTATCATTGAGCAACTCCTTATCAATTTTGATTCATTATCAATATGAATAATAAAAACAATTCACCGGATTCAATCAACTAGAATATAACAACAAAGTACGAATAAAAACTATATTAGGGAAAAAATTTCAAATATATATAAAATATAAAAATTTATATTTAAAAAATGAAATAGTATTCAATCAAATTGAATGAACGGAAAAAAATATCATAACATACACAAACACAAAGTTTTCTTTGGTCTTTACTAATTGGAACCTTTTTTATTGACGAGCCACAGAAATTGCACCTATCAAAGCAACTAAAAGAATTATTGAAATGAGTTCAAATGGAAGAAAAAAATCTGTTGATAAATGAATTCCTATTTGTTGACTATTACTTATTAAATCTTGTTCTAAAATCTGGTTTAATCTTGTAGTCCAAATAACCCCGTACCATGACGTATCGAGAATAGTAGAAATTAATGAAAAAAGAATAGTTGTACAAACCACTGAAGTAATCCCATTCCCAACAGTCCACAGATTGAAATCTATGGAATATTCGGAATCATTCATGAACATCACAGCAAATATGATTAAAACATTTATGGCTCCCACGTAAATAAGGAGTTGTGCAGCAGCTACAAAATGGGAATTTGCTAGAATATACAATAAAGATATACAAACAAGAACAAATCCTAAGGAAAAGGCTGAAAATATTGGGTTAGGAAGTAATACCACTCCCAGACCTCCTACTAGAAGACCAGATCCCAGAAAAACTAAAAGAAAATCATGTATTGGTCCAGGCAAATCCATTATATTATTAAAAAAAGAAAAAATAGAAATCCTTTTCATGACCTTATTAATTTAACCGGGGAATTTTTTTTTAATATGTTTCTAATAGAGTGAAATTAGAATCTAATGAATATTAATTGATGTAGATACAATTATTAGACAGTTTCGCTTTTTTATTCTAATATTTTCAACCTATCTATTTCAAGCAAGATAATAATTACGAAAATATTATATTAAAAGGATGAGCCTTAATACTTAATATTATTCTATAAATACAAGTTTTTAATTAAATTCTTTATATAATATAATTCAACAGTTTTGCATTCTTTTTTTAATAAAATTAATGGGTTTACCCATTTTTTGTTTGAGGTGAATTCAAAATTGTTCGAATAGTATAATCGTCAATTACTGACATTGGTAAACGCCCCAAAGCGATTTGATTATAATTCAACTCGTGACGATCATAAGTTGAAAACTCATATTCTTCAGTCATTGACAAACAATTTGTTGGACAATACTCAACACAATTACCACAAAATATACAAATTCCAAAATCAATACTGTAATTAAGCAATCGTTTTTTTCGAATATTAGTTTCCAATTTCCAATCAACAACCGGCAGATCTATAGGACATACTCGAACACATACTTCACAAGCAATGCATTTATCAAATTCGAAATGGATTCGACCGCGGAAACGTTCTGATGTTATTAATTTTTCATAGGGATATTGAATAGTTACAGGTAAACGATTTGTGTGGGATAAGGTAATCATGAAACCCTGACCAATATACCTTGCAGCTCGTAGGGTTTGTTGACCATAATTCATGAACCCGGTTATCATAGGAAGCATATTGTAATTATCTATGAATAATTTGATCTTTGTTTCTTTCTCTTGTTTAAAACAAGTAATGAATATCTTGGATTGATTTTCAATTTAGAGTGAAAAGAGTTGGAAAGAAGTTGTTAATAATAGATTACCAAGGGAAATCGGTAAAAGAAATTTCCATCCAAGATTTAATAGTTGATCCATTCTTAGCCTAGGTAAAGTCCATCTGGTTGCGATAGAAATGAACAAGAACAAATAAGTTTTAGCTAATGTAATAAAGATACCAATTGTTGTTCCAAAAATTTGATCCTTTTCAAATAGCTCCAGCATAGATATATACGGAATAGAAATATTCCAACCGCCTAAGTATAGAACTGTTACAAATAATGAGGAAATTAATAGATTTAGATAAGAAGCAACATAAAATAAACCAAATTTGATACCGGAATATTCAGTTTGATAACCTGCTATTAATTCTTCTTCCGCTTCTGGTAAATCAAAAGGTAACCTCTCGCATTCTGCTAGGGAAGAAATTAGAAAAATGATAAAACCTATAGGTTGACGCCACAAATTCCATCCCCAAAAACCATATTTTGATTGTGCCTCAACTATATCAACTGTACTTAAACTGTTAGATAATCCTAGTCGGTGATAACATTACTATTCTCACCGCTATTACAAAACCGTACATGAGGTTTTCGCCTCATACGGCTCCTCGGGGGCCGTAAATAAATATAAGGACCAGATTAGTATTATTTAGATGGATATGATGTGTTCTAAAATGGATTAAATAGAAATATATCTGGGGTCCCGAATTATACCAATGGAATTCTGTCTGCTCAAATTCTAAAACTAAAAAACGCGCTTCGGAATTCATCTCATCCTTTACAAATTTTAATTTCTATTTGTTGAGTAATAACTTAATCCTTTAATAAAGCACCCCTTGTAAAACTAAACCTAGGTTTTTCAGCCCGTCGTGTTTTTCAATTACGAAAAAGAATTAAACATCCTATTAGTTTCTTATTCATGATAGAAATTCTATTTTATTTTCGAAATCTATAAAAAAAAATATACTTGTTTCGTTCCTATTCTTCTTTCTTTCTTTTTTAGAAAAAAAGTAGGTGGACTTAAAAAAAAATAAAGGATTATTTCGTTTCTGATAGTCATTACATTTATCGGTGGATGGGAGCATACTCTGAATCGGAATCTTGGGGAGTACTGCCTGATAATTTCTACAAATTTCAAGCCCCAATTAACCTTCTTTTTTTGTTATCTTATGTTATGCATAAATATCCTTTTCAATTTGGTTAATCTCTATTACAAATTCTTTGTGTATTTTGGTGTTTCTAACCATCCACGCGTTTTTACCTAATTGCCGATCACTTTGTAATATATGTATATGTATAGTAATTTATATAACTGATAGTGAAAACGTCATACGGTTAATATTTTTTTTAACCCGCTTCAAGCCCGGCTGACTAATCAACCAACCTTGGGGTAAAGCGATTCTTACGCTTACGTTTATTTCCATTTAACCTTTGTACATAGGAAATGAGACTTAATTTTTCTTTTTACTGCTAATTTCTGAGCAGTTTTTTTTCACTCATATATAACTATCAAATTCAAATTCCTTTTATTAAGATAAACCCGAAAGATAAATATATATATTCCGTTTTTTTTTTCATTTTTTTTTTATCTAGAAGAAACGGAATAAACCTTTCTGTTTCAACGAATCGCACGTAGAGATATTGATAAAACACATAGAGTTAATGGTATTTCATAACTAATCGCTTGAGCAGCAGCTCGCAGACCACCTAAAAAAGAATATTTATTATTTGATCCATATCCTGACATAAGAAGTCCGATCGGAGCAACACTTGAGATGGCAATCCATAAAAAAATACCGATATTGAGATCCGCTAAAACAAGGTGATTGCTAAAAGGAATTACTGAATAACTTAGTAAAATAGAGATAACTGCTATAGATGGTCCAATACTAAATAAAGGAGTATTTCCTCTAGATGGACGAAGATCTTCTTTGAAAAGTAGTTTTGTCCCGTCGGCTAAAGCTTGAAGAATTCCTAACGGGCCGGCGTATTCAGGTCCAATACGTTGTTGTATCCCTGCAGATATTTCTCTTTCTAACCACACAATTACTAGTACACCAGTTATGATTCCCAATACAAGAGAAAATATAGGGACAAATATCCATATGAGTCCATAGACCTCTTTTAAAGATTCCAATCTAAGAAAAGAATTTATTGTTTGTACTTCTGTTGCATAAATTATCATTTTAACGATCAACTTCTCCCATAATTATATCTATGCTACCGAGTATCGTCATAATATCAGCCAATTTCATTCTTTTAACTAGTTCAGGAAGAATTTGCAAATTAATAAAACCCGGCGGTCGGATTTTCCATCTCCAAGGAAAACCACTTTGATCTCCTATGAGAAAAATTCCCAATTCCCCTTTTGGAGCTTCAACTCTTACGTAAAGTTCTTGTTTCGATAATTCAAAAGTAGGGGAAGGTTTTTTACTAATGAATCGATATTCAAAATCATTCCACTCTGGATTCCTTTTTTTATCAAAGCCTCTGCTTTCTAAATTTTCATAGGGACCCCCCGGAAGTCCTTCCAGAGCCTGTTGAATAATTTTGATGGATTCTGTCATTTCGCTAAGTCGTACTAAATAACGAGCTAATGAATCTCCTTGTTTTTGCCACTGAATTTCCCATTCAAATTCATCGTAAGACTCATAACGATCAACTTTACGAAGATCCCATGGTATTCCGGATGCGCGTAACATTGGTCCGGATAAACCCCAATTTATTGCTTCTTCCCCACCAATAATCCCAACGCCTTCAACTCGTTCTAAAAAAATAGGATTTCGTGTAATAAGTTTTTGATATTCAACAACCTCTGTTAAAAAATAATCACAAAAATCCAAGCATTTATCTATCCAACCATAAGGTAAATCCGCCGCTATTCCTCCAATACGAAAAAAATTATGCATCATTCTCATACCGGTGGCAGCTTCGAATAGATCATATACAAATTCTCGTTCTCTGAAAATATAGAAAAAGGGAGTCTGTGCCCCAATATCTGCCATAAAAGGGCCAAGCCATAACAGATGAGAAGCTATACGACTCAATTCTAGCATAATTACTCTGATATAGCTGGCTCTTTTAGGAACTTGAATATTTCCTAATTGTTCGGGTCCGTTTACTGTTATTGCTTCTGTAAACATAGTAGCTAAATAATCCCACCGCGTTACATAAGGTAAATATTGTATAATTGCTCGGTTTTCTGCAATTTTTTCCATTCCTCTGTGTAAATAACCCAATATGGGTTCACAATCAACAACATCCTCACCGTCTAGAGTAACAATTAAGCGAAGAACACCGTGCATGGATGGGTGGTGAGGTCCCATATTGACTATCATAAGATCTTTTCCTGTAACTGGTCTCTTCATAAGTTTTTCCTTGATTCGTTCTGGTATGAATTAGATTGCTGAAAAAGAAGTTTATTCAAAAATTCAAGATCTAAAAAATTAACTAATTCACAATTTTGGAATTTAACGAGTTTTTAATTCCCGAATATTCAACTGATTAATTAATTCTTTATAACGTACTCTATTTTTTTTTGACAAATAAGCCAGCAGTCGTTGACGTTTTCCCAGAATTTTTCGTAGACCTCTCTGAGATAAATAATCTTTTCTGTGCAATTCCAAATGTGAAGTAAGTCTTCGTATCTTATTAGTGAAACTGACTACTTGAAATTCAACAGATCCCTTGCTTTCTTCTTTTTTTTCTTGAAATGAAATGAATGTATTTTTTATCATAAAAAGAAATCCTTCCCTTTTTAATATGAATTGAAAGATATGAATTTTACTGATCAGTAATAATAATGGTAGTTTTTTTGTACAAGGATCCGAATTTAATTATCAACTTATTAATTCTTAATTTTATAAAAAAAAAGTTTAAATTTCGATCTAAAAAAGGAGGATTTTAAAAATTTATTTATGAATTCGCTCTGAGTGGTATCTATGTCATTAATTCAATGAATCTCATGTATAAAGATTGAATTAAAAAAAATCCCTCACATTTGTGCATCCAATTGTTTTCATATACCGTAACTTATATTATATATAGTCAAAATATGTTCATATACCGTAACTTAATACTATATATATAGTCAAAATATAGTAAAAAGGATCTACCATTAATGCATTTGAAATCGCGTATACATGTGTATTCTTATCATACTGAAATGATTTCCATTAGTCGTATTAAACCAATAGCGATTCATACAAGCTAAATCTTCTAATCGAAAATTGGGCCAAAGAAAGGATTTTAATTTAATTAGGTTTTTTTTATCCTTATCAAGATCTTTCTTTTTATTCAAAACTGTGGTCAAGTTTTGAATATTTGTATCAAATTTTGAATTTCTATCCCTTGCATTTTTTTTTTTTAAATTGAAACAAATTAGAATTCGAAATTCTTTACGTCGTTTAGGGGATAGAATAGTTTCAGGGACAAAGAAATTTAAACTTTTTTTTTTATAAATATAGCTTTTTTTTTTTGATCTTTTACTTATTTTTTGTTTATTTTTATGAACCAATGAAATCCCGATGGTTCTATATATAATAAGTTGGCCGTCGTTTTTTACAGACAAACGAACAGGTTCAACAATCAATATTCCTTTTTTCATTAATTTTGAAAAAGTGAAATTCTTCTCAATCATTAGAATATCTAAGCTCATCTCTCCTCTTTCAATACAAGATATCGTTATCTCGGTTGGATTTTTTAGTCTAACCAAGAGACAGTATGCTTTTACATTATTGAGGATTTTTTGATTAAAAAAACAATTCCATCGCAATTGAAAACGCGAATACCTTTTGAGAAATAAGTCAAGTTCCGCTTCGGTATTGCTTTTTGGTTTTTTTTTATTTTTACGTTTTTTTATCTTCGATTCTGTATAATTTTCTTCAATATTTTTTTCTTGCTTTGATAGAGCTGATTCCGTATTTATTTTTGTTTCTTTATCTGATTCAAACTCTTCTTGACCTGCTGATTCGTTTTCTTCTTTATTTAGATTAAAAAACCGAAGGAATTTTTTTTCGTTTGATGGTATAAAACCTTTTTTCTTTAGGGTGATCTGTTTATTCACATTTTTTGTTTCATTAAAATTGAAAAGAAGTGATTTAATTGGTATGACCCACGGTTTCATTTTATATGTACTAGAAAATAAGAAAAATTCTGGAAAGAAAAAAAAGTCAAAATTTGTTATACGAGGATTTAGTATTTCTTCATTCATTCCCATCCAATCAAAAAAATTTATTTTTTGATTGGCAAGACTCGTCTTAGTTATTTTATAAATTCTTTTATAATTCTTAACTTTAGTTTTAATATATTTTTTTTTACTCCTAGTATCAAGCTCAATATTTACTTTTTTTCTAAACCAAAAGTTGAGAATTCTCCAATCCAAATATTTTCTATGCAAAATTTCCCCTATACTGCGAATATTATATTTTTCTAGAAAACAAGAGATTAAAAAATTTTCTAGGCCTGTAGACATATCAAAAAATTTTTCTGTAGAATGAATAGATTTGTAGCAAACTGTAGAATGAATAGATTTGTAGCAAAAAAGATTATATATAGAATCCTTTTTGAAATTCTGTTTATGTTTTGGATTGAAAAATAAGTTAGCCTCACAAAAATTTTGTTTTTTGTAATTATCAAAAATTTTTTTTTCGTATGAATCCACTTTGTTTAAACTTGGATTTAGAACTAGAGAGTCTTGGTTTAGTTTTTTTTTCCATTTTTGGGTTACTAATCTAGCCCATGCAATCTGAGGTAAATTATATTGAGAATGACTTCGTAACCAGTTTTTCCATTGATTTATTTCGGAATTTAAAAGGGTTTTATCTTTCCATTCATAATGAAAGATTCCTTGTTCTTTAAAAAAATCTTTTATTTGATTCTTAACAAAAAAGGATGTTATGTATATGTTATATTCAAAAAAAGACTTTAATTTAGAAAAGTTACTAACTTGAATTTGTGATAATTTGTAAAATACATATGCTTGTGATAAAGAGCATAAGTCATAACTAAAAAAATTTTTTTTTGATATGAAATTTTTTATAGTCGAAATAAAATAAATGGTATTTTTTTTTTTTTTTTTTTCTCCATTTTCCTCATTCTTGTAAATATATACATCAAGAATTTTTTTTGTTGAATCAACAAAAAGTTGTGTAGTAATTCTTGGAATATTAATAATACCTAGAAAAATAGAAATAGACAGTTGTTCAACGCAAAATTGAAAAAAAAAAAAAGATTTACGAATTAATCGAGTTTTTTTTTTTTTTAATKTCTGCCAACTTTTTTTTGATGACTCAATTATTTTAGAATCATAACACAGTTTGTTACAACTATTAGTTAGTTTTTCTTTTTCTTTTGAAATTTTTTTCGTTTGATTTCTGATTGTCTTTATTTTATCAATCACATTTTTTATTTTGTTTTCGCTGAGTGAAGAATTTGGCCACTCCGTCGATTTTTTTTGAACAGATAGTTCATGAATCATCTGATTACTCATTATTGAATCTTTTTTAGTTTCATTTAATTCATATATTTCTCTCGGGCCAACTAATGGAATTCTATTTCGTTTTGAAAGGTTTTTTTTTTTTTCTTTTAGAAAAAGCAAGTTTTTTATAATCCAGTTTTTAATTTCTTTTTCGACTTTTAGGAAAATTGTTGCTCTTTCTTTGAAAATCCTTAAAACCGGAAAAGACTTCGTTTTGGATTTTTTGATTCTTTTTTTTAATTCTTTAAAAATAGGTTTAAAAAAAGAAGGCTTTGGTTTGGTAGAACCAAAAGGTAGGTCAGTTTCCAGCCCCCAAACTGTTAAAAAACGAAAATCATTTTTTTCTCCTTTTGTTTTTTTTAGTCGAGCCTTCTGAGATGATTGAAATTTATATTTATGCCAAGGTTTAAGATAAAACGGAAATAGGATTTTTATCTGAATACCATCCGTTAACCAGTTTCGTGGAAATTCTGTTTCGGATAGTTGAACCCCATTATAAGTACATTTAACATGCATTTCACGTTTCCACTCCTTTAAATCCTCTTCCCACTCGGGAAGTTGAAATAATACGATACGGATGCTATTTTTAATTATTATCAATAAAGGTAATATAATATATTTTCTAAGAATAGATTGAGTTACTAAAATAAAACCTCTTATTATTTGAGCAAATAAGAAGCTATCCCAAGTTTCCGCAATTTCTATACGTCTTTGTTCTTCTTTTTTTGATTGTTCTTCTTCGTTTTTATCAAAAAAAATTTTTTTTTTCCACATGAAATTTCTAAGAATTTTTTTTTTTAGCCCCCATATATCAAACGAAAAAAAAAAAAATTTATCTATTCTATCAAAAAAAAGGGGCGAATGTGCTTTTGCTTGCAAAAATTCCCAAATAACAGTTTTACGCCTTTGGGAACGCATGGATCCTTTAATTATCTCTCGACGAAAATCAGATTGTTGTGAATAACGGATCAAAGCCATTTCATCTGTTTGATCAGAATTTTGATTATCTTTGAGATTAGTATAAATCTCGTTATGCGGTTCTTTTAAATCAGTAAAAACCACTACACGTTTTGCTTTTCTTGAACGAATTCCAGGTTCGGTTGGTATATTTTCTTCAGTTTCAGCTTCCAATTCTTCCAATTCACTTGTTAATTTGTATGACCATTGAGGAACTTGTTTATTGATTTCATGGAAATCAATAAAATTTTTTATAAGAGTTTGATCATTATTATAAATTATAACGACATCAAATAAAATTTTGAAAATTTTTATTTCTTCTTCTGACTGAATTTTTTCTTCTTGGGGTTCGGAAAAAAAATAAAGTTTTTTCTCTATTGATAAAGACTTTCTTTTAAATTTTTCTATTGTTTGCTCAAATTTTTGAGAATTAATCTTCAGAAGTATAGCATGAATTTTGTTTATCCAAGATCCTCTTATATTCTTTTTTTTATAGGTTTTGGTTATGATTTGGAACGGAGATAATTTTTTGATTCTTCCGCGCGAAATCCCATGTAAAAATGGATCATAAATTTTAGGTAAATATTCTTTTTGAGTTTCGTTATGACAAAATCGAGTTGTTTTTTCCAGTATATTTTCAATAGACCCTTTTTTATCTAAAGCTTCAATTCTATTTAAAAATTCGTTTTTTAAATTTTCCTTTTTTTCTTCATTGACCAAACTCCAACAAGTATAAACTTGATCCGAGGTTTTTTTTTCTGTTGTAAATGAAGGGATCTTTTTTTGTATCATTTCAAAAAAAGTTGAAAGGTTGGGGGGATATGTAAAAGATATTCGTTCTTTTCCATCACTTTGGCATGTATAAAAAAAATATTGTGACATTTCATTTCTTACAGTATTTTCAATTTTATCATTTTTTATATATCGATTTGGTCTATTCCATCTTTTATAATCGAAAACTAGAGTTACAAAAGGTTTTTCAAACCATAAAAAACGATCCTCTTTTTTTTTTATTTTGAAAAATTCTAAATTAGAATTTTCT

>mitochondrial genome

AGAAGAGTTTGAAACAAAACTAAGGGAAGTAGCTACGATCGCTCGCAGGCCGTTGCTCGCTCCCTCCAAGTGTTGAACAGAGATAGCTACGATAGAACACAGCTAACAACCCATGACAGAATAATATGTATATAAGAAGACGGCTGCTTAGAGGAGTGATCTGTTCATCTAACTCAAAATATTGTAATGTAAGAAGAAGAAGAATCTTACGCCCAAAATTCCCATCTCTTTTTTCTTGGTTGGACCAACCGGCACCAGTCATTTCCGTCTTCCTTAATTGGGAGAGTCAGAATCAGTCTCTCTTTGTTTGGGGGGGGAGCGGAGCAGTCAATGAAGGAACCTTTGCTTTGAAAATGATTGTTCTAAAATGGTTATTCCTCACAATTTCTCCTTGTGATGCAGCGGAACCATGGCAATTAGGATCTCAAGACGCAGCTACACCTATAATGCAAGGAATAATAGACTTACATCACGATATCTTTTTCTTCCTCATTCTGATTTTGGTTTTCGTATTATGGATCTTGGTTCGCGCTTTATGGCATTTCCACTATAAAGAAAATGCAATCCCGCAAAGGATTGTTCATGGAACTACTATCGAGATTCTTCGGACCATCTTTCCTAGTCTCATCTCGATGTTCATTGCTATACCATCATTTGCTCTCTTATACTCAATGGACGAGGTAGTAGTAGATCCAGCCATTACTATCAAAGCTATTGGACATCAATGGTATTGGACTTATGAGTATTCTGACTATAACAGTTCCGATGAGCAGTCACTCACTTTTGACAGTTATATGATTCCAGAAGAAGATCTAGAATTGGGTCAATCACGTTTATTAGAAGTGGACAATAGAGTGGTTGTACCAGCCAAAACTCATCTACGTATTATTGTAACATCTGCTGATGTACCTCATAGTTGGGCTGTACCTTCCTCAGGTGTCAAATGTGATGCTGTACCTGGTCGTTTAAATCAAATCTCTATTTTGGTACAACGAGAAGGAGTTTACTATGGTCAGTGCAGTGAGATTTGTGGAACTAATCATGCCTTTACGCGTGCGCCCGGAAACATAGGCCGACTGTTGAGCCCACTCTGGCTCAGCCGCACCACCCGGGGGTGCGAGCCACCCGAGAAGCAAGCTATTACAGCGAGCGGCTGGAGCTGTAGGGAGCCGAGGAGCAAGGCAGTAGATAAGATAGAAGAAGGGGCCAGGCACCCGGTGGAGCAGAGGGGACGGTTAGGATAACGAACTTGAAACGCGGAGCCCGAGCGGCTGGCGAGCGAGTGGTTAGTGGCCAATAGCGCCCTAGTTGATGGCATTCCTCTCTGCGGCTGGCACTCGAGGAACCACAGGGCACTCCATACAGAGCAAGCAAGTCTTAGGGATGAGACGCCCGCGCAAGGACCTCAATTCTCATTAGGAGGTCGAACCAAGGACCTATGGAAGTCGGGGCTACCCCGTCCCCATGGGCAACGCAATAGTGTCCTGAGGGAGGAGTTTAGAGGCCTTATAGTAGCATGGACTTCTTTATCTTTCTAGGTCATGCGAAGGGGGCCAGTCCAAGATCGTACTGTTCCTCTACAAAGATAATAGACGCTCTCAACGGCTAGGCGCCACTCTCTTTCTGAGTTATTCCAGCTTCTTCATGATTTCGTGCCGCGGTGAACAAACAAAAAAAGAAGGCCGTCTCAGCGGAAGGAGAAGGACCTGCAACGGCAGAGACTACTGACCCTCTTCTTGTTCTTAGCCGTCTATTACGAGTCCGGGAAGCCTGGAATCATAAATATGAGGGATCCAGAAGGGTGGGCAGGGCGTTAGCAAGGTTTTTTATCCCCTCTTCCCGGTCAAGATAGATGGGAAAGGAGTCCTATCAAGTAAAGGCCATAACCAGCCTCTTTTTTTATGTACACCTTTCTTTGTTTCAGGCTCTTCAAGACCTTCCTCCTGCTAATCCGGCCATTTCCGAACCTGTCTTTCCCACCCTTCTCAATTCTTGCGATTCCTAGCCAGCCCCCTTAGCTTATTTTGCTTTATAAAACCACTTTTCCCTTTTTTCAGCTTGCTGCTCGCTTTCTCGCTTCCGAGAGGTGCTTTAGCAACTCGACTGAAAGGAGAGGGCCGAAGGCGCCTGACTTACGGTTTCAAAGCCTGGCGCGAAGCGAAGGGATTGGATTTCACCTATGATCAGATTAGTGGGCAACCATTGTTGACTTTTTTCGTGGTGTTGTTGACGTTGTTGAAAGCTAATTTCGAAGTAGGCCTTGCTTTTCTCCGCTGGGAGCAGCTCACACTATGGTGGAGGAGGTGCCGTGAAGATCTAGGAGTGTGAGCAGTACGAGCTGAAAGGCTCCCATACTGTTTGGAGGGCAGGGGGCATAGATGCCAAAGAAAGCTGACCCCTATCTATCGTCGTAGAAGCTGTTCCTAGTCTCATCTCGATGTTCGTTCCTATACCATCATTTGCTGTCGCAGCAATGGACGCCCCCCTCCTGGTTGAGTCAATGCTCCCATCCCCAAATCGGTCCTCGTCTGAGGATTCATTTGGGCTGAGAGTCCTTTGCGAACCATGGCCTATTATCCCCGATCTGGGATTAGAGTCTTCCATTGTAAATCGAATTCGAGTACTGGAAGCCGCTAATTCCCCCTTTCTGCTTGGAAAGGAAAAAGGGGAATATTGGGCAGAGATAAAAGAATCTCTAAGAAATTCTTCTTACCAAAGGGAGTATTATAGGGGTCTTGATTTCGAAAATCGAGATCTACTAATACGGGAACGTAAACACTCATGTTATGAAGTGTTTCGGGAAATCCTTTTAAGGAACCCTTCTTTGGAAGAAGCGGCGGCCTACCCTCCTCAAGAAAACTTTATTTCCTTCTTGAACGAGAAGCGGGACGCCCTAGACGTTTCCCACCCCGGGCACAGCCCGGCGGAAGTAGACCGTCTGGAGATCTTATTTCTAAAGGAGGTGGAAAAAGACCTTGTCAAAAATGGGAGCGGATCGATCCATATCATTGGAAATTTGAATTGAGATCATTTTTTTCGGCAGATCAAAATATGGTGCAGAAAAACCCAAATCGATATGAATGGAAGATGCCTCTGGAACTAGTATATCGGTCAGTCGATGGAACAATCACAACGTTACGCCCCAAAATCAAACTATATGGAGCCGTTACGAATGACCATAATGGAGTCTACTACACACTAGCCAAGAAAGAGTGTATTTTACTCACTTCGCCCGTTGATGATCCAATTCCATCCATTCATTCATTCATTATTATTATACGAAATATCACTTTTTCAGGCTAGTTTGTTCCGAGTAGGCATTCCATCCCGACGAGGTATTGTTAGCTAGGAGACGGGGATTCCTTATCTGTCTGTCAAGTAGAGTTCCCCTTCTCCGTCTGCAAGGTTTGTTTGTTCCTTGATGTGTTAGTTAGCAAGCGAAGCGATCTTCCTAAGTAAGCAAGCTGTAGTGAGCGAGTGCCTACCGCGTTGTGCGTATGCATCTACTGAGGTAAGGCACGATTGAGCGGCTAAGGGTTGCTGCTAGCGCATTCTAAAGAGTATTCCTTCTCTTTGTTCGGAGTAGCTTGATGAGAATAGGCTGCTAGCGAAGCGTCCTTATGCGAGCAAGCTGTAGAGAAGGAGTGGCTGTCGGGGGCAGTTACGACTGATCGGCTAGCTGTCTTGTTGCTAGAGCGCAGTAAAGAATACCTCTCCTGCCGGAATCATAAACTCCAGGTTCTTCGGCTGTTTCAGGAGCTGCAGAAAGGGATGCTTTAGCTGTTGAGGCGAAGAAGGGAATTGAGGTAAGGCTGGCATAAGAGAATGGAGTTACTGAGCTTACTGAGCTAATGGCCTAGAAGTAAGTGGAGTTAAGACAGCTCGTAACAGTATCGGAATACAATAGAATCCCTTCTTTACTTACCTATAGGCGAGTGACCACTGAACGATAAGAACAAAGCCACTTTCAGCTATAACCGGAGTGAAATGAGTTCTATAGGCCTTAGCCGGAACACTTTCTAACTAACTGTTAGCCAGTGAAGTGATTGCTAGGAGTGATCACGAACGAAGGATGAATAGAGGCGTAGTCTATAGCCGGCATAAGCTGTAACAGGAGTTCGAGTTTGAGTTACTCCAAGAACTACAGGGAATGCCATAGTAGAGCTAAAGCCAATAGCCGTAGCAGTGAAAGCTGTCTATCATAAGGTAGGAAAGGTCCTCTTGCTCTAGCAGTCAACGAATCGGAATCGGAAAGGGACGATCTTGTTACTGGTTCAATACCATAAAGGGAATAAGTTGAGGAATCTACATATGAAATAGTTCCAATATCGGCACAAAGATAAGGCGTTTCTCGGCAGGAAATGGTGTAACATCAGAGTGAATAGTTTCTGTAGCTGGTAACTCATTCACCTCTCCTTTCAGTCGAGTCACTCCGTGCCTCTCCAGCTTCTCCGAGCTATCTATCTCAAATTCATTTACTTTACTTACGAAGCCAGTCTCCTCTATAAGACTTTCATTCCCTAGACCTCGTAGGCTGATGATTGCCTTGATTGGCCAACAGTATCAGTTCAATCTGGCATTTCATCCTTGGCCTTGGTAAGGGTGTACACTATCAATGAACCAGTAGTTGCATTTACAGCAGAAGATAGATGGCATACTTCAAAAGCGGGAGTAGAAGCAATTGTATTTTATGCTACTAGGGCGGCTACTGAATCAATTGATGGAGTTATATGGGAATAAAAAATCCTCCTTAGAAGTGAAGGGCTTCCCCGCGGTTATGAGAATTTCTGGACCGCTTTCACTTTCTGTTCAGCATTTTAGGTATCTATAGACTTTCCTTCTCTGAGGAAGATCTACTTCTAATGATGTAAGTGGTTGATACCAGGGACAGTGGAAGTGGAAATGCGATCCCGGGGCATTTGAGTGTCTAAGGGCTGTTGGAAATCTGAAGCTATGACGGTAGGGGATAACGGCTAGTTGTGCGTACTAGGTACCTAAAGCGCTTGGGGCAAGCCTTCTAACATAATAATAGAAGTTCCCGGGTCTACTGAATCATTTACAAAGTCGACAGTTGAATTAGACAGGAAAAGAACCATCTGAAGCTGTTTCGGCTCTAAATGAAAGAGTAGGACTTTCTCTAGTAGCTTCAAGCATTCCAGTCGAGTTACTAAAGTACCTCAAATCACTTGCTTGGAGAGTTCCCAGCGAAGAGTTTTTCCTTTAATCAGCAGGTCTTGTCAGAGACTTTCTTTAGATGACATTTCTCTCACTACCAATAGTCTCCGCTTCAAAGAAAAGCTTTGAAGGGCTTACCTGCTTTCTGTTCGAGAGTAGGAATCGACTCTAAGAGCAGAGTCCGCGGCAAACTAGAAGAGTGAAAACTAGAAGATTAAGATGGAACAAGTCTTTCTGAAGGCCTGACCTATCAATAAATAGGCTTTTTTTTTCAGAGTGGGATATGGAAACTTTTCCTCTGTCTATTAGACAAAGCGGAATCCAATACGAATTAAGTGAAAATGATGGCCAATTGAATCAATAGTGACATCCACGCACATAGAATAGAAAGCATGGACTAAGAAGGTCTGCAAGCCTTCTTACTCTGCTTCTCAACATTAGCACGACTACTCCCACTACACAATACAACGGGTAGAGCCTTTCCAACATAAGCACTTATACCACCGGGCTCACCTACACCCCTTGAGGTGATTCAAGTCGACTTACGACTCTTCAATCTATCCGATACCTTCGTATCGTCAGATGAAGTACGCGACCACCATTCCCGAGGACCAGGGAATGCCGGTGCTTTTGTGGATCTGAACCTACATGATAGATCGGTTTCGGGGGATGCTCATTCAGGGGATGAGAGTCCTTTCCTCGAATTCATAGCTCATTGGGATTCGAACCCAACCACATACATATTTGACTGGAGTGAGACGATCCTTCTTTTTTCCTTTCGATAAGAAAAGCTAGGAGTGAGACCAAAGCAGCTTGTCTGCAACAAATAGACTCAGAAGTGAGTGATCCATACCTTGACTGAAAAGCTACCAATTAGGTCAGTAGCTGGCCGAGTCGCTGATAGACCAAATAAGCACAGTAGCAGTTTAGAGCCGAAGGAGCTCTATTTGGGATTCTATAGCCTACGCGCTTGCCTTTACTTTAGGGGATCGAAGTTGGATGAATCTCCAGTGGTTCCTTCCATCGGCGTCATCGAACCACGTTAGCAATCCAGAATAACTGGAAGCTCGAAACGACCGGTCAAAGGAATTGATCCGTTTAGTTCTGTTCTTCTCCTAGTTTTATAGCACACCCATGTAGAGGGTTTCCTACGACATTCCACTTGCGGAATGGAATAAAGGCTGGTGCTAGTCTAAAAGACGAATCCGAACAGAATCCCCTTAGTAAGAGGCGTTCCTCTCAGTCGAGAGACTGGCGTTCCTCGAGAACCAGACAGACTAGCTATCTATCGATTGTAAATGGTATGCGGATACAGGTCGAAGAAGATGAACTATGAGTTGACTGCGTTAATTAGCTGCCTGCTTCTTGCTTTCGTCCGGTAAGGATTTCCCGCTTTGCCCTATGGACACTAAGGTTAGATACATCAAAGTTTGAATGCCTGTATAGCTAAGAAATCTGCACTCTGAAACAAAGTTTTTGATGAATGAAACTTTCAGGGAAGGGTAAGAAGATGCTAGAGTAGTTCCGAAGGCTTCTATTCTAATAGGGCTGCTTCCTCTTTCTCCCGTTGAGTTGGGCTACCAGTTGTAGAGAACGTAGGACTTCCCGGTAAGCGTATCCCCGAAGTGTAGCGCAGGGCTGTCCGAGTGATTAGATTTCTAATTGAATCAAAGCGTCGGCTAAGTCAACGGTGCAGCTAAAGCAGTGATGTCAGTCTTTCATTCCTCTGTTTGTTTATGCGGAATGGGAAGATGCTGCTAGAAGTCTCGTTAGGCCTTTGCTAGTTCCTCTTCTTGTTTGGTTTGTTGTTCCAGCGAGCGTGGAGAGTGGCTCGTACTCCTGCGTTTGTAGCTCCTGTCAGTCTAGAATATCTCTTCTCTTCGTTTGAGCTATCCTCCTGTTGTGAATCTCTATCCTTGCGGTGTATGGATTCCTCTGTAAGTGCTGCTTCCTATCTGCCATTACCTGTATTCTATCGATAGGAATGGTTGTGGTATGAGTTAGTGTGCGTAGCTGCCTTGTTTCTGTGTATGCACAGCAGTTGTTTCTGTGTGAAGCATGTATGGAGGTAGTGAGGAACGGCCTGCGAACTACCGTAGCTAAGCGGCACTAGTTCCTGTTGCTTGCAGCTTCAGTATAATTCATATCCCTTGTTGGTATGTGAGTAAGTTGTTGCATGAATGGTCAGGTAGGTAAGCATAGCAGTATTCCTGTTTATGCATGTATCCCTGCGGGTTGTCTTCCCTATTGAATGAGGGAAGTGAGCTGGTTAATCGCAGCTTACTTCTGGAGTGGTCCCTAGGCGGGTGTAGCTATTGTTGCTTGTAGCTGCTTAGATCCCTTGATTTAGGCTTCGATCCCCACTTTTCAGGCATCTGCTTCAGGAAAGGCAGTTGACTTGGTCGAATCCCGATTACGATCAAAAGGAAAATCTTGACTTGCAGTGGAATAACGGCTTTGCGGAGGAAGACCATCGAAGGCTGTTGCTGGGAGAGGTCCATTAGTCCGGGTCGATTGCTTCCATCCTCAATTGCTTCTTTTGAAGGTTACATGCCTGCTGTTGAAAGCCTGGGACGAGACAGAGTTAGTAGGCTTTGATCCTGCGGTTGAAGGTGATATACCAACAACATGAGCTAGCATGGTTTCGAGTGCGATAACATGGGATATGGATATGTTTTCTTCGATGCCTAGGCAGGCTGGGATTGGAACTGTTCGTGATTAGACCTTCTATATGACTGAACTTCCCCGCAACAAGATAGAAAGAAGAGTCTGATTCGGAGAAAGATTAAGATCGTCAGTGATCTACGGATAGCTACCGACGTGAGAGGCCTAGAGTGAAGCTCTGGCAGAGTGGCTCCCTTGTTAAGTGTAGGGCCCCTTGGCTTTGGACAACTCACCAAGCCATAGAGACTAAAGAAAGAGCTAAATCAGTCCTTGCTGCAACTGAAGGAACGAAAGAAATCTACTTCCGAACTGAGCGCTAAAGGTACATACGGAGAGACCTACGATTTCCATTCTGTTTAGGCTATGAAAACCTCCCCGGATGAAGGAATGTAATACATTGAAGACTTCGAACTGCTTACGTAATGGAAAAAGGGGCTAGATGGCTTACGGGATATAGGGTTTTGAGGAGTTCCTCTGACTCACTTTTTGACAGAATCAAGGTCCTATCATTTTCTTCTTTTCTGAGTGAAATCAAAGCAAGACCATAAAGGGAAGAAGGTTGGTTACCTGCTATACTAAGAGTAAGCGAGCTAACGAGGCCTAATCCTGGGTTCACGGCATTCCATTCCTACCTATTCCATTTCGAGGGGTAGCAGTAGATCCTGTCGATTCAAAACCTTGTTCGAACTCTCTTTGGCATCTCACAAATGTTTCAAAATATATAGTACAGATTGGATCCTGAGCTCAAGCTAAAACTACCTACAAGAGAATCCATACTGAATCTATTATCATAGATCTTGCTTTCGTCGGATACGTCTCTTTCTCCCCAATTTGGATGGATCCTCTCATTCTAATTCATCGCTACTAACTAGAAGAAGTTCCTGGACTAAGAAGAGCTCCCCTTTCTAGGGAGATGTGGACGAAGGCTACTTGAGCTCATTCTCACATTTCGGGGTAAGAGGCTAGGCTAGGAATAGGGTACAGAGGCGTAGGGGGAATGAAAGAATTTGCTTAGTCATAATAGTGCTTTCAAAGTGAAAGGAGATAAAGTAATGAATTCCTCTGCTTGCAAGCTACCTATGATTTTCTTCGTCACCAACCATTTCCGCTGGCTCAAGCATAGCATCTTTTCCCCAAGCCCATTTCCTCCAGGATCGGTAGGCCCATCCACCTCACTATTATATTATCCAACCTATCGCTTATTCAATTCTAAAGATCGATTTCCTGCTTCTGCAAAGGGCGGAAAAGTCTCATCTGCTCTCTTCGGTTAAGGTGTCCACGATACGAATACCTATTTCCTCTAAATATCTATTTACCTTTCCCAGTCTTTTGTGCTGTAACAGCAGTCGCATCTGGAATATAGAATCTAGCTCTTAGCTGGTCTAGTCCAACCCCTCTTCTGTCTGATGACAATCGGGAATACCTTGCTAGCGAAGTAAGCAAGAAAAGGGATGCCATTAGTTTGATTCAAAGGCATATCTATAGGATAGCCTCTGTTTCCGACGGCAGTTTAGGAAACTGGAATTCCTTTAGTAAGATAGCTCATGAATGAAGCTTTCAAACCACTTCCTAATTTCAGGAGTTTAGGAGTACATATGAGTTCATGAGTGCATGAGTCAATGTGCCCGCTGTGGTGTATGAGCAAGTGATCTGATCCTATGCGCCTTTCTCCTGGTTATCCCATTCCTTGGCTTACATGGGAAAGACTAACCTCCCATTCTGACTTTCCTTCCCCTTTCCTCGATTGAAGATTGTTTCATCACAAAGTCTGACCCCTGTCAAACAATTCCAACATTCACAACAGGCAACATTCAGTGATACGTTATGCGTTAGTTGAAAGTACTCACTCTCCCCCAACTCCCAACATTAGCAACATTGAGATTGTTAACATGGGAATTCACAACATCGTTCCGGGCTGACCTCAGCTACAGTCTTTCCCTAACATTAGCATTAGCAACAGGGGAATTCTCTACACTAAACAGATAACAAGGGTTCTCCTAATTAGCCAGATTATGTCGGAGTCAAGCAAAGATTGTATGAAAGCAGAGAAGTATTAAGTAGTGACGAAGGACAGGGATAAGTACTAGAACAACAAACAGAAGTAGCAGAAAGATCTATTCAAAAAGGCAGATAGGAATGATTGAAGGTAGGCGTTCCTCGGAAGGTTAGGGAAGGGATAGCAGTATACAAAGTTCCTATAGCTGTGAAACGGTTATCCTCCAAGGAGGAACCTAGCTTCTTCAGCGAAGCTGGAGGAACCCCTATATTGTCCAGGGATGTAAATCAAGTTGGCTGGTTAAAAAACATTGGTCCAGCTTAAGCTTGACGTTGAATGGGAATTTAGAGCTCCAGTCCAGCTAGAAGAAGCGTTGTTAGAAGCGGCAAAAGCTATGAGATTCGAATCATAGGCAAGGAGTTGTGTCAAGAAAAAACAAAAGACAATCGAAATGGACAATCGGACGGATGGCAAAAAGATCCAATAACTGAGAAAGACAAAACAAAGATTCTTTTCGAGAAAATAGAAATAAGACAAGATATATGAGCAAGAAATCTGCCAAAGGTCTTCGAGATCTTGTATTTATATGTCGCTCTATTAACAGAGCCGTGTATACCGGAAAAAAAAGAAAAGCGTTATCGGGTTTGAACAACTGAGTTGTTACAATGTTCTTTTTCATTTTAATAAAGTGACTCCTCTTCTTTCAGTCACTCCGTACCTCTTCTTATGAAAGATTCAATTTGCATTTCATTTCTTCACCGGGCTTAGACCATGTCTCCCGAACAATCTCAGTACATATGGCGCAAGACGATTCCACATATCGAGGTCGGAATGGGATCGGGTGTTTTCACGTCTCACCGTAGTGCCCGGTTTGTCTTGATTTCCGATTGCTGAACAAGAAGTCAAATGGAACGATTGTATTTTGTTGTGACCCGCGCCCGCTACGCGCCTTTGAGTCGAGTGCATTTCCATCGGGAAGGATTCAATCCAGCCACAGGTTCCCCTACGGCTACCTTGTTACGACTTCACCCCAGTCGAAGACCCCACCGTGGTATGCGTCAATAAGACCACCAAAAGTCTTTGTGTGACTAGTGTTACACAGAAGTGCTGGGTGATCATTGTTCCGATGCTTCGGGCGAAACCAATTCCCAGGGTGTGACGGGCGGTGTGTACAGGGCCCGGGTACATATTCACCGCGGCATGCTGATCCGCGATTACTAGCGATTCCAACTTCATGTTCCCGAGTTGCAGAGAACAATCCGAACTGAGGCAATCTTTCCGGATTCGCTCCGCCTTACAGCCTTGCTTCCCATTGTAATTGCCATTGTAGCACGTGTGTGGCCCAGCCCATAAGGGCCATGCGGACTTGACGTCATCCCCACCTTCCTCCAGTATATCACTGGCAGTCCCTCGTGAGTGCGTCACGCACCTTTTTGTTTGTTTCGGAGCGGTTTTGTCAGGGCGTACTAAACCCACTTCCTTCGTCCCACACCACCGTTCGGCTCACCTGAATGCCGAGTCTTTCTCCGCCGACTAGTTAGGGCTTGGAAAGGCGCCGGAGGAACCCAGCTTCTCCCCTAAAGGAGGAACCCCTTGTTACTGAGAGCAGAGCTAGTTGCTGTCACTCAATTCCTAGGTCTGGCACATCACTCGGCTACTTGGCTTACTTCGGTTTGCACAACCTTTCTCCTTAGGCGCATGTCTGAGCAACACAAGGCGAGGGTTTCGCTCGTTATAGGACTTAACCAAACATCTCACGACACGAGCTGACGACAGCCATGCAGCACCTGTATGAAAGTCAGTACCATCCCGTTAAGGATAGGGTTTCTCGTTCATATGTCAAGGGCTGGTAAGGTTCTGCGCGTTGTATCGAATTAAACCACATGCTCCACCGCTTGTGCAGGCCCCCGTCAATTCCTTTGAGTTTCGGTCTTGCGACCGTACTCCCCAGGCGGAGTGTTTAACGCGTTAGCTGAGCCCCAGATCAGAATAGACCAAGGGCGAACACTCATCGTTTACGGCATGGACTACCAGGGTATCTAATCCCGTTCGCTCCCCATGCTTTCGCACACCAGCGTCGGTAGAGACCCAGAGAGCTGCCTTCGCTTTTGGCGTTCCTTCGTAGATCCGTAGATTTCACCCCTCCACACGAAATTCCACTCTCCTCTGTCTCACTCAAGTTCAGTGGTTTCGAAAGCATTCCGCCACTTTTTGACGACTTTCACTTTCAACCCGATGAACCGCCTACGTGCCCTTTACGCCTAGTCATTCCGAAGAACACTTGCCCCCCCCGTTTTACCGCGGCTGCTGGCACGGAGTTAGCCGGGGCTTCTTCCTCGAGTCATGTCATGATCGCGCACTCGACGAAAGAGCTTTACGAGCGGAATTGCCCTTCTTCACTCACGCAATATTGCTGGATCAGGCTTGCGCCCATTGTCCAAGATTCCCCACTGCTGCCCCCCGTGGGAGTCCGGGCCGTGTCTCAGTCCCGGTGTGGCTGATCGTCCGAAAAGACCAGCTAAGCATCATCGGCTTGGTCAGCCTTTACCTAACCAACTACCTAATACTACGCAGGCTCATCAAACAGCGCTTTCTAGCTTTCTTCGGGATTTAGCCCGAACTGTTCGGCAGATTCCCACGCCTTACGCACCCGTTCGCCACTTTGTTCACAACTCTTCCCACCTCTTGGGTGAGACAAGCTACCTTGAGCTAGGAGCCTCCATTCCTTCTGACTAGCTCCCAGGGAACAACGTTCGACTTGCATGTGTTAAGCATATAGCTAGCGTTCCTTCTGAGCCAGGATCAAACTCTTCTTTTGACTATGATTGGGCCCTGCAGTGGTAGAACCTCGTGAACCGGGCGTACTGACTTCCCAACCTTCTGTGGACCTTTCTTCTCTTATGAGATTTCACTTTCGAGAGACTGAACCTTCGGGTAGAGAAAGCTAACTCAGCTAAGAAGAACAAGGCTTTTTCTAGTTTGCTTTCAAACTTTCTGTGGGCCTTTCTTCTCTTATGATATTTCACTTTGTTTAGTTTATTCTATCTATCTCTGTCCCTACCTTATACTATCCTTCCTATCTGCCATTTACCTGTATTCTATCGATAGGAATGGTTGCTCTACCCGAACTGAGTCTTATTCCTCGGCTTTGACTTCGAAGATACTCAAGTCTACTCTTTAGCGAGGGACTACTTTCTTTGCTTGTTCGGCTGGGAATAGGTTTCATGACTAGTTCTGCTATTGTCCGTAGGTCTTTGTTGCTGAGGTCTCATGCATGTCGTAAGCGAGTCAAGCTCTCCTAGGTCCCTGGGCTCTCCCTTGTTTCGATGGTTGGGAATTGTCATGAGTCATTCAGTAAGTAGTGTTATCTGCCTTGCTTTTGTGTATGCACAGGATTCCTTGAAGTGGAGCACGCCCCAAGGGTAGCTGTCAGTCTAGTAAATATCCCTTTCCTTGGAATGAGTAGGCATGTTATAGGCGGTAAGTAGGTCGATAGCCGCCCTGTTCCCGTTGATACATGGGATGTGTTCTCTATCCGTTCTGTCCCGCCTCGTCTAGATCCCTGCAGCTAATGTGGAGAAGGTGAGCTCCCTCCGAAAGTGTAGATCGCGAGTGGTCTATTGGGGGTGGGCCCCGGAGTGATCGGCTAAGGTACGTGAGCTCTTCTTCTGTCATTACTGAATCTCTCCTCTCTTCGTTCGATGTTTGGGAAGTTCTCCCGTTTGCTTCCCCCCGGTCATTACCGGGTACCCTTCCACAGTCCAACTGAGCAAATTAGGAAAACTTTTGACTCGAAAGAACTCGACTAAAAGGAATTCATTGGACTTTCCGATACAGCAACTCAAAAAGAACAGCCGTCCTTACCGGTGAAGCCTACTGTCTGACTCTTAGCCTAGCTACTTAGATCAGACTGAGCGAAACTCTGCAGCTGCTTAGTCAAGTTCTGCTACTGACTCTCTTTCAGTTGAGGTGGAATCAAACCCTTCTTTGGCTTAGGGTTATGCCCACCTCTACTTGTTAGTCTTGCTTTTTTCAATTCCTTTGGTCGAGGAGTAAGCCTCACCCCAAACCGACACTCTTTTACTTCATTACCTTTCTTCCGGCGGGGCATTTTCTTTCCTTAAGTGACACCTCTAAGGACCAGTCAGTTACCAGCCTATTTATTTACTATTATATGACATCCTATCTGTCTAGTGGCTAATTGATTGAATTGGTCGAAGGATTGACTCGGACTCAGACTCAGGTCGGGAAGTCCAGCCCACAAAATGCAAAAGCCAATACATAGAGCCGGCTATAGAAGATTCAAGATTGGCGCTAAGACTAACTTCGGAAGGGATTACGAGTACAAAAGTCAGAATGATTCGGAGAAAACCGAAAGCCTTACAGGAATGGAGGATTGGCAGTTAGGCTCAAGTCTTTCATTAAGATATACCGGCTTGGCAATCCCTTGTCTGTTGATATGAAAGAATGCGCTTTCGGGCGAACTAAGGCCGAGGTGTCTCGAGACTTCCAGGCGGAATTTTCAATAAGGCGATAGAAGGCATTGGGATCAATCCAGACTTTGAAAAACTGAATTGACTTGAGTCCTTTACCGCGGGGGGGCGGCCTCATCTCATCATTAAGTCATTAAGATAAGAACTCCTTGATTGAATGTTGAATGGCATAGCAGCGGGGCAAGATCGGGTTACCTCAATGGCAAAGACTGGCTGAGACTTCCTAAAATGCTGTATTCGTAAAGGCAAGCAAGAGCCTAGACGATTTCCACACATCGAAGAAAGAAAGTGAATCGTTTGTCTGATTACAATGGGACCTAAGATAGCCCTAACTCGTCTTTTGCCTAGAGAAAGTTTAGAATCGATATCCATCTATCTGCTCCTATGCTGCTTCTCCTTCTGCTGGATGAGTGAGCAAACTCTGACGTTGGGGCGGATGGCGGCTTCATGCGCGCATTCTGTTGTGATGGGGGCTCTGTTCTCCATTTACCAAGGAATAAGAGATAGGTAGAAGCGGGCTCCATGGAATGTAGATGAGAAGCGAGCAGACCTGTGTGAGAAGCTAAGAACCTAACAGTCACCCTCAATTTATCGGTAGTTCTCTCTCTTAACCTTCTTTATGGTCCGATCCTAAAAGCTAGAGGAGCACACTAGTAGAAGTTACGTCTTAGCGCTGTAGCTTTCTTTCAATGTCGAGATGAGGAGCGTAGCCTATCTTCTTTTTCTCTCTGCGCGTAGGAACCACTTTCTTTCTGAAGAGATGGGCGTGATCGTCTTTGGAATAGGCATAAGCGCCGTTCTATTAAATAGATTAATAGAAGGGCAAAGCCTACCATTCATTAGTTGAACCGGTTTCTCTTGGATAAGAACGAGAGTCCAGAAAGAAGGCAGAGTTAACTACTAAGACGAGAGAAGTCGGAACTAGTGCAAGTGAGGGAATTCGCTCTGCTACTGCTAGGTCGAAGCAAAGAAGGTTAAGAGAGAATAAGTAGTTCCGAGGGATTTAGGCTTCGTCTGCTCTCAAGCCAGCTCCTAACTCTCGGTCTTCTGGCGAACAAACTTACCGGATTCAAGTGATTGAAACACAGGACCCTTTTCCACTTGGGTGACGACATACATAAGGTTTGACGAGCATTCTCGGGTGGTAGAATGGTGTTGGGACTATGGAAACTGTCGATCTCCAACTTGAAGGTGGGCGGGAGTGCTTGAATCGGGAGGAGTAGATTCTCTTACCTGTGGTTCTCTGCTAGTGGATACTTCTCTCTAGCGGTGTAACGGGTGTTGGCACTTCGGTTGTTGCCAGTCCTGTCATTTCCTAGAATATGTGTTAGTATAGGCTGGAATATGTAAGTATTTTATATAAGTTCCCCGTTGGTCTCTTACTAGTCTGCTCTTCTTTGTTCCAGTGAGTTCTGTCATGAAGTGTAGCTCATGTTAGGATTACCCGGGGAGCGAGCTTGTCGACGACGACGGGTAAGCCGTGTGAGCGGTTAAGGGTAGCTTGTTGCTCAGCTGTCTTTAGTCAATATCTTATGAATGTTCCGGTCCATAGAATAATGTTAATGTAGCTAGAGTGTTCCTAAGGTGCATACTAGGGCTTCTTCCTCTAGTTACCGTTGTAGCTGAACCAGCACTTGCAGCTGTATATTCTGGGCGAGGAGCGTAAGCGATATGGCATATTTGTGAAAGAATCTCCTTTCTCTTCGTTCGGAATGTTGCTAGAAGTATTCCCTGTGGTTTGTACTAGTTGTAACTTATGTTTCCCGGTGTTAGAAGGTTGTAGCTGTTCAGCGGGTGCAGCTAAAGGAGGACCTCCAGGTCCGTAGAACTCCTATCTTTGCTTTGTTCGTACGGGAATAGGTGTCATGAAGTTTCGTCTATTGTCCCTAGGTCTCTGTTATGTATTGTTAAGTAGTTCTGGCGGGCTTCTTTCCTTGTTTTGCTTGAGCGGATGCGCGTAATGTTGCTGAATGTGCGTCTGTTCTCTAGGGTTTCCTTGTGCTTTTGGGCGTGACTAACAAGTGAAGCTGTTTTCTTTCGTTGTTGTTATGGGAATATTGCGCTTGGGTCCCCTTTACCTAACCAACAACTTCATCTTCCTGTGTCTGCTCAAACAGTTGTTTTCGCTTTGCTTGATTAATAGTTGTGGGAATGCGTCCTTTGTCCGCTTCCCTTTCCTTGGTTGTGTCTGTATTACGGGCTGCTTTCTCGATACTTCTATTGTTGCGTGGAAGTGCTGCTTTCTTCCTGATAGTTGTATCCCCGAATCCAACTATGATTTGCTGAGCTGTTGCAGCTCCTTTCCTTTCTTCTTTGTGGGCGGACTGAGAAGGGCCCGTATACCGTACGTATCCCTCCGGTGATTGTCGTTCTGTCTTCCCTTTCCTTCCGGGTTGTCCGGGACTGGCTTCAATGTTCGGGTAGCATTACCTTTGTTCTGCTCTCCTCTCTTTGAAGTGTACTTTCCGGCCGAGAAGTAGCTTTCTTTCTAATATAGGTTTTTCTCTGACGACTAACCTATATTAGTTCCTGCTGCTACTGTATGTTGTGTACCGAGTGCTGGCTGTTATAGTATGGTTATAGCGTTGATGGATTGTTTGTTATCTCCTTACTGCTATTAGCTGTTGTTATAGGGCTATATTCCTACTGTTGTTATCTGTTAATATGCTGTTGTTATCTGTTAATATGCTGTTGTTATCTGTTCTCTGTCCTTACTGTTGTTAGGTGGGAATAGGTTGTTAGCGTAGCTTCTGTTCCTATGCTTGAATGTGTAGTGAAGGAATGGAGGGAAGCTCTTATTAACGGCCGGAGCTAAACTCCGCTTAGCCCGGACAGGGACAACCAAAGCAGGAACTTAGAGACTCCTAGTAATACATCATACCTACTAAATCAGTCTATTGGGGCTATAGGTGTTCCCCCCGGGAAAATAACCAATGGAGTTGATTACGTTGTGAGTAAACGAGGAAAGGCGTAACATTCCATAGTTAAGGGTCGTTCATTAGCCCTACTAGTCAAGCACGGGAAGCTAACTTTCTATCAACAAAGCTAGCGGGGTTTCCGGGCCTTGTTTTGGTCCAATGTGAAAAGCTACATCCGAGGAATTGTATGATTCAGATACCTGACGAAATAAAGAGAGAAAAGATCGGGCGGGATAGCTGGGATGACCTATTTCAAGATCAGATGATCCACCAGCTTGTCTGCTTGTGAGGTTGAGGATTTGACTGTTATGATCTCTTGGCCCGTGCCTCCGTAGTGTAGTGTAAGTTCTTAGACCAAGATTCGAAACCTTGTCTTTCCCGCTTCCCTTCGCAGGAAATTTTTGACAGAAAGTAGCGACACTGATACCTTAGAACTAACAAGTACCAGGCCTAGACCGGGGAGTCGGAACTAATAACAGCTTTTATAAACTCCAAATCCAACTCTTCCAACTCCAAGTTGGACGCAGGAAACTCTACCTACAACCCTCAATTCCGGAGGTTGGCAGCTCATCAACTACGAGAAAAATGTGATCGCAATCAAGCACATAACATTGAATCTCGTGCAAGACATTCTTTAGCTAAGTCTATAGCAGCTGATTCAATAGCGGATGGTCTTGTTGAAGGCTTTTTGACTTCTACTGAGAGCAAAGTCCTTACTTACCAGACCGGGCTACTTGAATAGCAGAAAGAAAAAATGTCACGAACCCAACTCCTAGGTCACTAGGAAGAAGAATGGAGGGAAATAGAAGAGGTCCTGTAAAGAAGCTTTCATTCGATGCTTCTGATTCAACTCCCAGACAAACAAGGAAGTATGCGCGAAGATAGGGTTAGTCCCTGCGGCAGGCATTATCCGGGAACGATTATCCAGGAACTTACAGAAGGTTGGCGTGACACGCCTAAATCCACAACGCTTCAGGCGAAGAGATATATCTTTCCGCTTTAGCGGCTTGAACCCTTCGATCAAGAGATACCTTAAATAAAATCAACTAAGTAGCTGACCTCTTAGACGTTCGTGGGTATTCAAATTCATACATAATCTTCTCCGACATGAGATAGAGTGAGAGGGCCTGGCCTATTCCATAGAACAAACTCAGCAAATCTTGATCCGTATTCCACCTCAGATGGTAGAAGGGGCAACTCAGCCTTGTCTTTTTCTTTGGGCTAGGAACCCAGGAGAATGTTCAAAGCGATAAGAGAACAGGTCTTTTCAGAGAAGCTCTTGCCACTCTTCTGTTAGAGCTCTTTAGTCCCATTCCTAACAACAGGGCACTAGCAACTGGGAACAAGGGAAACACCAACTCCCAACTTCCCTAGCCCAACCTTCCCAACCACCTCGGAACAAATATCATGCATACATAGGAACCCGGCTTCGCTAACCTTTCTTACTTAGTCGTGAAACAACTGTTCCCATCGAACGGAGAGAGATAGTCAAATGACAAAGGAAAGCAGACCTCGGAACGCGCTGCACAGCACGGAACAGCCATTACATCAGTGATCGGCAAACAAAGATAGATACTTAACAGCAGCTACAAGCAACAATAGCTACACCCGCCTAGGGACCACTCCAGAAGTAAGCTGCGATTAACCAGCTCACTTCCCTCATTCAATAGGGAAGACAACCCGCAGGGATACATGCATAAACAGGAATACTGCTATGCTTACCTACCTAGAGGAGAATTTCATCCTATGTGCTCTACATATTTTCTATTTCTTTCTTATCTATCCCATTTAGCGATAAGATACTCGGGTTATGTTTGGTGAACTGGGCCGTGTTTTATATCCTCCTTCCCCACGGGAAAACGTTTGCAGATGCGATCATGAATCGAACCAGATCGAAACATTCAGCTGTCGACGGACAAACTTTGCCGAAACGTCGACCGCAAACGAAGGATGGCCAGGCCCCGGTTCCCAGGGTTATAGTATTCCCTATTATGAGCCTACTCAAATCAGAACTAGTTGATTCTCTTTCGCCTATCGGCCGGCCGGCTTGTTGCAACCTTCCGAGGAACGCCTGCTGAGATCCCAAGTCTCCAAGTGGGCCCTCTTGGCCACCCGCGACCTTGGCTTTTTAGAGAATCCCGCTCCTGAGTCGTGGAACTTGTAGCTCGGCAGTCCACCGGGTGGGTTTGTTTATCCTTCCAGTTTCGAGTGTCTTCTTGGATAGTTATAGCGGCCCATAGGCGCGAGATGTACCTTGTGGGGGGGGGGCGGCGGTCCCCTGGACATAGTCGTTTCAGGCAGTGGCCGTTTAGTCCATGGTCCATTAGATGGGAGGTGCAAGGCCAGAAAATTGAACACATTGATTCCGCTCGTTCCCGTCCTTCGCTTCAGGGCCTGCCCCTCGGTGTGGTCAGTACTCCATACTGTCGGGCAGCGAAGCTTACACTTGTTCACTAATTATGACGGTTCGCCAGGGCCTCTTTCCTCCTCCCTTTTCTGCTCACTCGTAGGGGTCCGGACCCCCACAAAGGGGGAGGGAGTCGACTGAACATCTCAGCCATTGGCGGAAATTTCGCCCGCATCCGATCCCCAATTCTTGTTCACCCCGGATGATCGTGTTGGGTGAATTGTGACCTCGTACGATCGTGTTGGGTGAGCAACAGCCGCTTCGTCACAGTACTTACTTATGGGCTAACGGGTCACACTTTGGCCAAGTATCCTACAAAGAGACTCCCGAGAGCCAGAAGTATTGAAGGAATGGCCATAGGAATGGGCGCATCATGACATCGTGAGATGTCTCGCCCGAACGAATTAGTTGGTACTAGAAATGTGAGAAAAAGTAAACGAAAAGAGTAATAAGAAGTGAAAAGGACAGAAATACTTCCCAACCAGAAAGCAAAGTTCCCACTGATGGTATACTTAGTGTAAGCGAGCTCTAAGATCACATCTTTGGAATAAAATCCAGTTAGAAAAGGAAATCCAATTAGAGATAAGCTGCCTATGAGCATCATGGCATAGGTCAAAGGGAAGGAGGAGGCAAGCCCCCCCATCTTCCGCATATCTTGCTCATCCGACATGGCATGAATCACCGAACCAGCACTCAGGAATAGTAATGCTTTGAAAAAGGCGTGATTCATTAAGTGAAAGACGCTAACCGAATAGTTAGAGATGCCGCAAGCAAAGATCATATAGCCTAATTGACTACAAGTTGAATAAGCGATGACCCTCTTTAGATCGTTCTGTAATATTCCAGTGGTTGCCGCAAGGAATGACGTCGTAGCTCCTGCAGAAGTAATAACAATCAAAGCCGTAGGTGGGTATTCAAATAAAGGGGAGCACCTTGCTATCATGAAAACGCCAGCTGTTACCATAGTAGCTGCATGAATCGAAGCGGATACTGGAGTGGGACCCTCCATAGCATCGGGTGACCAAGTATGCGATCCTATCTGTGCAGATTTCCCAACAGCACCAATAAGAAGTAAAATACAAATAAGACTTATGGCATTAAATCTCATATTGCAAGAAATCCAAGAATTTCTGGGGGCACTAGCACGAGCAAAAATGGTTGAAAAGTCTACTGTTTGAAAGAGAGTAAAACGACCCGAAATCCCAGGAGCTAATCCAAAATCACCTACTCGATTGACAAGCATAGCTTTTGTAGCTGCTTTATCTGCCTGAAGTCGTGTAAACCAAAAATGAATTAACAAATATGAAGCAAGACCTACTCCCTCCCATCCCAGGAATAATTGAAGAGAGTTATCTCCAGTCACCAACATTGGCATAAAAAAAGTAAGAATGGATAAATAACACATAAATCGAGGGCTATGCGGATCCTCGGACATATATGAAATGGAATAAAGATGGACCAAGCTACTTATGGATGTAACCACAATTAACATCACTACGGTCGGGCTATCGAACACAGAGTCAGAAGTGAATTACGAGTCGGACCTATTTGCGAATCGAGCGAGCTCCCCTTGCATGCAATGATGTGGTGGTGAACCTCTCATTCTAATTCAGTGCTCTCCGAACCGTGCGGGAAGGTTTCCCATCACACGGCTCACCAACTTGATCTTCCGCGGGAACCGTATGTCCAAACAGGCCTAGAAAAACAGGTACGATCTCGCTTTCCTTTGCCACTCAAGTGTACGAATCAAATCCGTGCTTAGGCCCCTTCTTCCCTTCCAAGAGTCCACCACCCACCTTAGTAGTCTCAAATAGGGCGTGCAGGCCTCCCTATTTTTTGAGTAGGTGATTCACTACCGAAGCGAAGAAAAGGCTGGATCAAGAAAAGGGGGTACTACGAGCCCTCTGCCCCACGCATCTAACCAGCTCGCGTGGTTCACCGGTTCCACCGACTAGACCAAAAGAGTGATTCAGTCGATACAGAGGTGCGCTTGAAGTGGGGGGTGTGCTGTCCCTATTGGAGGAACCCCTTCCCCATAAGGCCCCACCGTCGGGGCATAAGCGCCCTCTTGCTACCCATATGCGAGGCGCCGTCTTAGCCTTCCCTGACCAGGATCGCTCCCACACCTGTAGCGTTCGTGATCGGCCTACTCAACTGTGTATCGATTGAAGGGCAGGAATTTCCCACAACCAAAGGGAGTGGTTACGTCCAGTATGTCCCCCCTTCTTCCCGACATGCTATGGTGCCCCGGGGTGGGTAGGAGCGGGTCGAGTCCGTATCGCCGCGGAGCAACAGCCGCGTCCGGATCAGATCTATCTACTCGGCAATTCATCCGGTGACTTCACGGTCGCCAAAGAAGCCCCAAGAAGCATCAAACATTTCCGATGAGATCCATGGAGCAATTCTTAGATAGCAAGCACTAGCTCCCGGTGCGACTTCATAAAAAGCAATCAAAGATAAGATCGAAGAGAATGAAACGCACGTAGTGGTCAGAATAGCGCTTCCTTCTGATCCTAGAAAACGTCCGAAAAAACCTGCTACGGAACTACCGAGCAGGGGTAAAAATACGATAAGTAGATACATAATTTCGAGTGTGATCAGAAACCTAAAATCAGACAATGACAGAGCGGCCAGCGATTGAGTGATAGATTTCTCGACGTCCGGAGAACGCTCGACCGAAGAAATGAGTAACTAACAAGGAAGATTTTGTTCCCCAACTTGCTTTATTAACGGGCATTTTCGGGGACTAGCCCGCTTCCCATTACTCAAGAGGGCAATTCCTCGCACATAATCAAATTAAGGGAGCCATTGAAAGGTGACTAAAAGACCAGAAACAGGGACTACCCGAGCTAATGATAGAGGCAAGAACACTTTCCGGCCAAGTCCCATTAATTGATCATAACGATATCGTGGAAATGCTGCACGGACCCATATATATAGGAACAGAAAGAGAAGAACCTTGATACTAAACCAGATCGAGCCCGGGATCTTCTTGAAAATGGGAAGATCTAGGATAGGCGGCCAACCTCCTGGAAAGAACAATGTACATGGACCAGGTGAGTAAGGATGCAGCTCCGTGGACCGCTCGTCGGGCCTGATAGGTGGTGGTATCACACCCTTCTCAAAGAAACCGTACGTGACACTCTCGCGTCATACGGCTCCGCCCCGGAATCATGCTTACGCCCTCTCCTTTCAGTCGAGTCACTCCGAGGAACGCCTTTGACCAAGGGGTCCTCGAACCAACCTGTCCTCCCTTTCTATTCCTAGTCAGGCGTTTTCATTCATTCATTGATTGATTCAAGGTACGAAGTGACAAGTCCAAGCGATAGCGGTAAAAGCTAGTCGCCGGAAGCGAACTTCCGGGCCGGGAAGGGGCCAAAAAAACGTGAGCGCCCCTGCAATCTTTCTAAAGAAACAAGCGAGAAACTTGACTTTGAGAAATACCTTATTATATATAATTAGTAAAGGCGTGTTAGCCTATCTATAGTAAGGGGCCTTTTCTTGATCGTTAGCGCTTTACTAATAACAAGGACTTTTCTCGCTTGTTTAGTATTGCTTTGGCTTCGCTGTCCGTATCTTGCTGGCGCGGAAGCTACCGCAACTAAAAGAAAATGAATGAAGGAAGAAGGCATTAGAAAGACTACCGAGGCATTCCGGGCCGACTACAATACAAGTCATGAGCGATAGCGAAGCCAAGCCGGATAGGCTTTTTTATGTCAAAAGCCCCACCCCAAAACTAGCTCTATCTTATAGCAGACAACTCAGGCAAGTCTACTCAACTAATCTCATAAGTAAACGCCTGTTCGCATCGCAACTAATAGAAAAAAACGACTACTAGACTAGACTAGTAGTTGAGTGTTCCTTCTTGTTCAGGTCTTGACCGGGTCCGAGCTTCCCAAGCTCTATGCTGTTGGGGAACTCTGCAAGGATCTTACCACCTTCTTGATTTACAATATTTGAGTCTTTGGAGTACTTGGGGATTATATTCCGCGCCGAGGATTTGTGCTTGTGGGCTAGGGTGAATATTGCAGACCAGCGAATCTGGTGGTCGACAATCGTTCGGACTTGGTAAAGGTTGTCGCGGCACCTGTAGTAGGACAGAGGACTTATCGCGATGCCCGCGGACCAATTTACGATGTCTTCGTCGCTGACGTTCGTCAAGCAGGCCACGTGGATTGGCCAGGGTCTTCTTCGGCTAATGATACCTCGATCCCGAAGCCTTCGGAGTATCTTTTTTATAGGTGCCTCTATCTTTACGGGGAATTCGCTGCTGATAGATCCCGCCCAGTGTCCTCCTCCCTCCCCCGCCGCCTTCCGACCCGGGGGAGTATACAATGACAACTTACGGGCATGAGTGCCCGATCGTGAGACTACCTGTTGAACGTCCGATGACGCGTTGCTCCGACCTGAGCTATGCAAGAACGAGATCCCCCGAGGAACGCCTTGCCGGATGTGCTTGACGGTCCCCCATAATACGCTAACTTGGGGACTTCTGACTCCAGCTGTTCCAAGAGTCTCCGCTAGTTGAACCCCGTCCTGTAGACTCCCTGTTTTGCTCATCCCCTTCGTCAGCTGTTTGATCGGGATACTATCACCTAGATTCCTAAACTTTGAATGGATGGCGGAGCGTAGGTGGCAAGCAGTTATATGGATACGGTGCTTTACCCGTAGACGCTTTTCCAGCTCTCGCAAAAATTGTATGGGAGTCGTCCTCGGAGGGACTTCCCGAATGACCGTACCGAGGAATTCTACCGTACTCCGTGCAGCTATTGTTGTTGATCCTGCGGAGCCTACCCAAAGGTTCAGGCCGGATTGTAGGAAATGGGCGATACGTTTTTGTATTTCTATGAGAAGCTCTACGGCACCCACGATTCCCAGTAGTAAGTCGTCGGCATATCGCGCGTAACAAATCCTTATTAAGTAATGGGTTTTTAAGGGGGCCTGCTTACGGGCCAGGCCTCTCTCTGACCTGATAACTAGTATCGCCTCCCCGCCCAGCTCTATCAGCAGGCCCTTTCTTTTGCAATACTTAAGAAGGTCTCTCATGGCCCAATTATTATTACAGCGTTCTCCACCATAGAATTCGGCCTTCGGGGTCAACCCGGCGGCTTCTATGAGGAAGGCGGCGCAAAGAAGGCTCGAGGGCTTGTTAAGGAAGACGGCAAGGGCCGACGAAGGGGGGAAAACGAAAGGCCTTTTCTGGTCCCCCCTGAGCCGGAGGGTGCTTGTGGGGGGGGTGTGCCACGACGAAACAAGGGAATGAAAGGCCGCTTTGCGTTGCATGCTCTTAACGCTCCCCACAATGATGGCTCTGTTGTCTTGGGGAGCGTTGAAGCTTGCTTCTTCTCCAGGGTTTTCTTGGTCATCAATACGACGACCTGTCCTTAATAGAACCGATCTGACTCTCTGAACAATCGGAATTTCATACTTCTGTCGGATCCTCCCTATCTCCTGATCGAGCTTGTGTAGGTAGATGTTGCCTGGTAGGGCCGATAGTAGTACACTGTGTGGGACGGAGTAAGGGCCCCTCTCAACTCCTACGAGTCGTCCGGCGGAAAAGACTTTATGAATGGAGTAAAAGAACTTGGGATCGTCGATCTCTTCCTTCAAAATTTGGATGAGTCGATGTCGATCGATGGTGTGAAAACACTTCCTGATGTCGAATTCTAAAAACCAGCGAGAGATTCCCCACTCTTCTTTGATCCGTCTTAGGACCGAGTGGCAGCCTTGACCCGAGCGGAAATGCGATGTGTCTGGAAACTCGGGATCGTAAATGGATTCGAGTACCATTCTGATCGCCTCTTTCATGATCTTTTCTATAGGTAGAACTACTGTGAGCGGTCTAAACTTCAACCCTTCTTTCTTTCTTCATATTGTAAAGGGGGGAAAAGCTCGTTTTTTGCTCCCCTTTTTGATAATCAAGGGAGGAACCCGTCGTTTCAGTGACTCATAGGGCTTCCCTCAGTTCAGTCTTTTTGGTTCTTGAAAATGGTCGCCACCTCTCTTCTTCTTTCAGTCGAGTTGCTAAAGCACCTCTCCCAGGACCGGAATGATTATCCCTGCCCGATGGGTCTACATCCATCCCTGAATGTCGTCGGGTACTGTTCACTTCCCCGCCATTCTTGTAACCGTCACCTGTAATCCGCGCAGGTGTGTCCGCACCCCCCTGAGTGGACGAGAAAGAAAGGATTTCTCGGAGCAACCCCCTAGGTTCCAGACCCAGGAGTCAACTTTCCCGTATGAGCATTCGGTACATGTATCAGTCCGTGGGAAGAGTGAAAGGGTCACCACTACTGAGGATCTCCCCCCCTAATCTTAGATAGGTCGTCTGAGGGTTCGCCGCGGTTCATTGCTGTGCTTACACACAAGGCTACCCTTCTCCGAAAGCTACGCGGGACCACCTACCACTAGTCTTCGGCCGGAGGGGTTTATTGCACAAAAACGCCGGGACGCAGGCTCCCGAAGAGGGAAGCCCAACGAATGTCAGATGCAAAGTTCCGCACCTCATTAAGATCATATTGGCATACTCTCCCAAAAAAAAAAGAGCAGACCCCATTGAAGACGAGAGTAAGGGGTTCCTCCAAGGCCATTTCTGTCCACCGCCCTTCTCACGGAACCGTACGTGGACGTTACCGCTCATACAGCTCCCAGCCAGCAAGCAGTTAGCCTTCCTCTACAAGAAATGGAAATGTGGATGAATCGAATGAAATCGAGGAATTCGGTTTTTCTTTTCAGACATGATAAGTAAGAGCATCCCTTTCACAAAAACCTACGGATCCCCCGAGGAACGCCTGCCACTTCAGCACCTTGTGATCTTTGAGAAGATCATTACGAGCCCTTTCCTAGAATGTTTTTGTAGATTCCGAAAATTCCAGAGTGGATCAGGACGAGAATGAATCCGGGAATCCAGGGCATACTCATCCTGGAGCTTCCGCTCTCCTCTGGGAAGGGCTTTTTTATGGATAGAGAGATGAGGAGAGGTATTTCTTACTCGACTAAAAGGAGAGGGTAGCCTCCCGTAAAATAAGAAAGTATGAAAAACTGGATTAAGAGACGAAAATTCTTCAAAGAAAGCCATAGAAGTTGTATCATCTATTATATTCCAGCGTGACTGCTCACGTCGGGATAATCTTGATTTTAGAATACCTTTAATATACTTTGAAATATAAGCTCCACCCCCTTCTTCACTGCGTATGCTCTCTTCTCTCACTGCGAATGATGCCAAAAAGAGTTCAGCTCGCAAGCGGAGTCTCATTGGCCGTGAATCAGAAGCTACCATGTTCTAACTTGCACTTAGTCAATTGGGGGGAAGATTAACCACAACAAATCGTAAACATCAGATCGGACCTTTAATAACAAGAATTTGATTAGCGTTCTTTCTTTTAGTTTGAACAACAAAGTCCGGGCCAACAATATCCCAAGTTAATCTGTAAAAGTCAGCTGGAAAACCATCCGAGCCAGGGGATTTGTCGAGCGGCATGCTGAAAAGACAATTTGGAATCTCCTCCGTAGTAACCTGTTGCAGAAGATTCGCTTGCATCTCGTTGGAGCATCTGAAGTCGATAAAAGAGCGAAGGGATGAGATAGAGGCTCGAGTGTGGTTCACCGGTGAAAACTGAAACAGGTTGGAGAAGTAACGAATGGCTTCTTCTTGGATATCACTTGTTCGAAATAGATCCACACCTTGATCTGATGTAATTCTGCGGATTGCATTAGCAGATTTTCTGGCTTCGGCAACTCCATGAAAGATCGCCGATGAAAAAAAAGGCAATATCCACTCGTAGATCGGCTTGGTTATATTTATATAAGGGCTATTTTGCATATTTTGAATGTTTATAGGCAGTAGGGTAAGGAAAGGATCATAAATCTATGATAATTTCCGAGTTGCAAAATCCAAATACAACCTGACTTGAATCCTTACTGGACTACTTCATATGCCTTCCGCACACAGGACAGAGGAATTCACTCCACACACGAGCCAACTCCCCTAAAAGGCCTTTCTTTCATGCAATTGGCGACGCCATCGCCTATATCTTTCTTGTTAAGATCTTAAGCTTCCTGGACGTCTACAAATCTGTTAGGCAGGAAATGTTTACATTGGGGAAGAGTTCATGCTATCATGTCGCTAAAAAGTGGGCTCGGGGAACGCCTACTACTCGACTAAAAGGAGAGGAACGCCTTTGACTTCTTTCTCTTTCGAAAAAAAAAGGAATGTAGTTAGCCTAATCACTGGGATGATGATTGGATCAGCAACTTCTCTTGAAGCTGGACATGGTAGGCTGGTTTTGTCAACTCATTTGCTTCCGCGCTCTCTTCTGTAAACGACCTGCTTACTTCTCCACTGACCCACTCATAGACTTTCTCTTATAGACTACGCATGAATGCTTTCCTATATCAAGAAGAAAGCGAGAAGTCCGTTGGGATGCTAGATAGACTGATTCCCCTGGGTCCATAGCGTCACACCCTCATAAAGACAGTGACGTCCTATCCTATCATGCTCTCACAGGCGAGAAAGTTCTTCCTTCTAATTGAATGTCCTATTCCCCTGCCCGAGTCACTCTTCTTCCAGGCTTAAGGGGCAGCCAGGACATCAGAAGTCAATTCAAGACTTAACAGGATCGGTATATGGTTTTGCATCAAAGGCAATTCGCGGTGAAAGAAAGTAGTCTCTGTTAGCTCTTGCTTCAACCTTGACATCAGTCAATGAGGTTCCTAGGAAAGTCAATGATTTTGTTGCAAAAGCTCTTCTTCTAGCTACAAGCATTCCCTCTTCAGTGGAATGAACTACTTGTCAATGAAAGGGCCGTCAATTCCCCATCAACTGGATAATTAGCATCGGAATCAGACTCAGAAAGGGAAGCGGATGCCACAACTACTGTAAAGTCAAGAGTTTCATAGGGAAGGTGACGCATCAAATGCTACTATAGTCGACTCAGTTACTGGTTTTGCTGTATTAGTAGCGAAAGGCTGAGGCGTATAATAGAACAAGGTATGTTCCGGTAAAAGATTATCTGGTTGGGATAGGGATCTCTCCTTCTTCATTTCTCAAGGCCGGTAAGAAATCAAGAAAAGTGGTTGGGAAGTGCCCGGCCATCTACTAAGAGCTACTAGGAGCTAACTAGCTATCTTGGAAATGCTCTAAACCGTAGAATCACAACAGCCCTTCTCTTAGGGCAACAGGGGAACAAGGAACCAGGGAAAGACCAACACAACTTCCTTGACTTCCTAGGCTTAGAAACCCATTCCTATAAACGGGAAAAGCTTACTAACCTTACAACTCTGGAAGGGATTACTTTCTTACCAACTGACATGGCTTACCTGGCTTACCTTACTAACAACTAACAAACATTCCTGGGATTACTTCCTGACAGGGCTTAGCTTACTTACTAACCTTCCTTACACGGAATTACAGGGAAGCCAACACCCGGGATGTTCCGAGTGAAAGTAGAAGTTTAACCGGAACTGTCGAAAGATTACTACAGAAGAACCCATAAGATGTCCCTTACATATACCGATGTAAACAAAGATCTGAACGATTGAGAGAAGGAAGATAGAGGAAAGCAGGAGAAACACAAACTGACCGAGCTAGCGATGTTCCTATTAGGCTTGGAGAACTACTCGGAGGATGAGACAGGGAACAAAGCACAGTATACATCCTACTAGAGCTGCTAACCGGTATTCCTCGGTGGTTCCGTCCTTTAAGCCAGGGAAGCGAAAAACACAATGAACCAAGCGGAACAGACGGGATACAGTCACATCAGTACAAACAAGGAATACTAATTAAACAGAAGCTACACTGGGTACGTACTTAGCTACGGTCGCTCGGAGGCCGTTCTTCGAGTAAGAGACTAAGCAGGCTTCGCTCCTTCGTTTCGTACAGGTGCTGAAAGTCTAGACAGCGGGCCCTCTCCTTTCCGACTGGGCTAACTCGGGGAAGGGTCAATCTCCTCCGGAGCATGCTGCACAACCACTCATTCGTCCTGACTAAATAGTAGAATCTATCTCTCAGCGATTCTTTTCTCCGCCAATAACCCCTTCCCAACCAGCCCGCCCGATCGATTTTGTAAGATCGGCTAATTTAAAGGGGTTAATCTGGTCCGGAGTTGTCGGAACGAATCGTTTGCTTTATCAAAAAAAGCTTTATTAGGGGGTCTTGGTTGGCCCCCCTATTTTCAACCATTATAGCTGGCGTCGACCCGCTTTGCGATACGGACGGACACGAAGAAAAAGGCAGGCAGCGAAAATGCAAAAGAGAAGACCAATGATCCCCGCCCCGGAGCATGTTATTGACTCAACCACTAATCTGTTGAATGAAGGTAGCTTGCTTACTCTCAGCCACTAGTTAGAAGGAAATCCCTCTCCTTTTAGTCGAGTTACTAATTGATAGTACCTCTCCAATGGCCATTGCTGGGGTGGGGCATTCTATCAAGTTAGAGCCGGTGCTTAAGTTGCCTGAGTTTGGGTTGAAACTTATGGCAGAGACCTTGCAGCTGAGGAAAGGGTTGGCTAACATAGCTCGCCCTATAGTAATATGTCGACCGCGCCACATTAGGTATAACTGGTAAGCTAAAGGAAAAGGGAGCTGTTGTCAACCAACGGGAACTAGTTATCCTGTCTATTCATTCTCTTTCTTCTAGTTAGTCAACGGGCGTAGCAAGAAAGTAACCTAACCTATTTATCGGGATGTAGTAACAAAGCCAAGCACTTGTTTTACCGTGGAAAATCTCTCTTTACATAGAAGAGAGCGCAGCAACTCCCCCATTAATTAGGAACCGGTGGAACTGGAGCACTTGTTTATTTACCATCTCCTTATACTTGAAGACTCTCGCCCGAATTCATTAGTATTAGTTGGTACTAGAAATGTGAGAAAAGTGAACGAAAGCGCTAGCGCGTAAGCAAATCCCTTTAGCCCGTCCACTACCGAAAGAGAAAGAAAAGAAAGAGCAAGAAAAGAGAAATGCCAACCCGTGGGATGAGAAGTTGCAGAGTAGCTGATAGTGGCCTCCCTCTCCGAGTAAGAAATACCTCTCCCAAGGGTCAAGTCACTCCGTACCTTTTTAGTCAAGTAAGAAATACCTCTCCTAACATGACTTCGAGAATTGGGCGGAGCTGGAACATTTGATGCAATAGCTGACAATCCAATGGGAATTATCCAATCGAAGGAAGAATGATTCATGACTACTCTTGCTGCTTTGAAAGAACTCTCTGAGCTTGGTTTTCAGTCAACTTTATCTCTCTCTCTTTATGAGCGGTGGTCATGCTCTGTACATTCTAGTATATGTTGATGATCTTAGAATCACATGGTCCAATCCTCTGAATATTGATGACATAGTATGCATCAAAAATGCCATCTTTTCTATAAAGTATCTGGCTCAATCTTGGTCAAAAAGTGAGTAGAACTTCTGCTTCTATCTTTCTCAAAACATTACTGATCTATTGGTCAAGGCTGGCATGGAAAGAACCTCTCCCTTCTCCCATAGTCACTTCTGGGTCCTCTTTGTCTGCTCTGCTGTTGGAAGTTCCACTTTGCTAATCCTCATCTCTATCGTAGCATTCTTGGTGGTTGCTAGCATAACCAGACCTGACATAGCTTACTCAGTGAACTTTCACAATTTCTGATGAGACCTTAGATAGAAAAGAAGGTTTAGATTCCTGTTTCTATTCCTTCCTATTAAACGCTTTCAAAAGATTGTTGAGATATCTTCTCTGAATTGTCTGCACTTGACACCTTCCTCTCAACTTGCTCTTGTTTTAGTGATGCTGATTGGCCTTCGTGGGAATTTCTAGATCACTCTCAGGATATTGGCTTTTTCTTGGGAGATCCTACGGGAAAAGAAGGAAATAAGTAAAAATAGGAGAGTCAGAGTATGGGTCCATGTCCGCCACCGCAAGTGACTTAGTCGGTCTCCTTACTGATCTGAATCTTTCAATCTCCATGTCTTGTGAAAAGAAGCACAAGATCTCGATGAATCAACCTCAGGAAGAAACATTTGACACGGGATTCTCACTAGATTCGGTATACAGTTCAAGAATCAGGGAGACTGATCATCTCATATCTTGCTCACCACAATTAGCTGAGAAATCACGTCCATCAACATAGATTTTGATCCTCCAAGTCCTTGTGAATTGAGTTGGGAGAGTGATGAACTAGCTATCGGGGGTATGGATACATAAGCAATAGTCCTTCTTTTTCGGCTCTACGTTCTCAATGCTTCGCTTCGTCAGTCTGCGACTCACCTCGCCTCTCTCCTCTTCATCCCTATTCTATGGCCCCTAGGTTAGAAGTGAGTTTCAGTTCTCTTAGGTCTCTTTCCACATATACAAACTGAGAGTTCAGTCTCATATAGCATGAGGGGACATGGCTTGGTAATCGATTCAAATTCATTCCCTCTGATCGGCAATGACAACATCATCACCGAGTACCGCGTACGATGTAAAGCGTACACCAGGCAGGGTCAGGATGCACCTGTTTCGCACACCACCACACTAATATATGGTGTGATAGCGCGAAAGTAGGCCAAGAACCGTGATATCCCCAATGGCTGCCCTGCCACAAAGCATACTTGAGAGAACCTTCGTTTAAGTTTAACAAAAGGCACCTCAAAGATATTGCATGCAAATGCAGAATTAACCACACTTGAGGCAAAGTAGCGGTCAAATAGGTACTGCACCACCTCAAACAGAAAGACTAAAGGCCAACGATCAGTGGCCGACTTTAAGTCAAAGGAGAAAGAGTGCCTACTGCCAACCAGTCGATCAAAAGGCTGTGTTTGGTTAAAAGTCCGGGGAGACGTCTTCTCCGCCCCCCCAAACATGGAAGGGGTGTAACAACCTATGATTAACGTAGTTCCCAATGGCGAATAGAAGGCTTTTGCCACCTCCCTCAACAACCTGACCTAGTCGGCCCATCCTCCGAGGTTTTTGTTCTGCTGATAGCTCTTTTAAAGAAGGGGTCTCAACGAGCCTCCTGAATTTCATTTCATCCATACAGGAGAATGCATCATTCTTATCGCAGAATGGAAACAAACCGGATTGGATTCGCTCTGGCCATAATAGACCTTGATTGAGTAACCGGGTATCCACTGGTACACATCACCTGGATCATGTAAGAGAAAGAATCCACTTTCAAAGCGAGAGACGGAAAACATGACCTAGCTTTCGCCAGCGCCTTCGGAGGACCCCTCCTGTTGACAAATTGTTCAACCGGAACAATCGTTTTTTGGGGCACCTACGACATCCTACTAGGCACTCCTTGGTTGATAACACAAAAATTGTTCCCTACAGTCTTCATTCTGTCTTTCTGCAACAGTAAGAAAGCAAATCGTAGATAGCAGAATACCGGGAATATCAATACTATTGGGATAAGAGCAATATTGCATATTGCCTAGATGTTAGCAGTTGCTATCTCTTAGCAGTTTAATGCGGTTGAATGCGATCCCCGTTAACTCTGGTTTTTAGAGCTATGAGTTGAAGAGTGAGATAGACGTCTAAGTTTGAATGTTTACCGAAGGGCGGAATGATTGCCCATTTTCAGACTTACCGAGTGGGAATAGCAGTCAATCTGAGATTGCTTAAATCTCATAAAGTAGTAAGACTACCTCTTTTTTTGTAAGTGAACGCAAGGCTAACATAAGCTAGAGTTGAACGATCTACTGAAATAGCCTGATGATGAATGGAACCTCTTGAAGACATTACCTTGGCTTTTGATTTTAGGTCTTGAATTCTTTCAGATCCTGCCCCGCTTACTTTCGCTAGGCATAATAGGCCTCTCTCACTCCTCACCGAAACTGCGAAGGGACGGTGAGCGGCTTGGCTTACCGCAAGTCCCTACACAAGAGCTTGTTGACCATAGCAGAGACACGGCCGTACTGGTTGGGCGATGGCACAGTACGCGGGACCCTCGTATGAAACCCCACTGAGCTTCCGGTGGAACGGACTATTATCGGATGGGACACGCCTATAGCTAAAGGAACGTACAGCGAGCCGTAGCGGGAGGGACGTCAGGATACCACGGCCCACGGGCAAGCCAAGCCAGCAACCTTCAACTCCCGCAGGTCGTACGGGGTCTTTCGCCGGAGTTCACGGCGGTCTATTCTCTTTCCAGATTGAGTGGATAGGGGCTTTTGGCTAACGTACGTTCAGGGGTCTGCCAGTCAACTACCTTCTCATCTAATGAGGTTTTCAATACCACGAGTCCGTCGGTCGTTGTGTGGGCGCCCTCTACTTCTACTTTTAACTAAACGCGAACAGCCGGCATTGCAAGCAAATAGAGAGCCCCCGCCCGTTTGAATCGTTGCGAGCCGGAAAGCGTACCGGCAGTTTGAGTCGCGAAAGGACCGCTTGCTTGATTTTATTATTATTCTAAATAATAGATATATAAGATTATCTATCTATAAACAATAAGAAAATCATTATTTTATCTAATTGGGCACTCCTGGGAAGAGGAAGAAGCAGATAGAGCAAAGGCCTCCCCTTTGCTTGCGTCCGCTCTTCCCGAAGTGAGCAAATTGCATGTAGAGATCCGTAGGGGCTTATAGTTTAATTGGTTGAAACGTACCGCTCATAACGGTTATATTGTAGGTTCGAGCCCTACTAAGCCTACCACCCCTTACTCTTCACCCGAAATAAGGCAGTCGAAGTCGGCACAAGAAGTAGGCGGAGTAGAGGTCCAACGGAACTCGACTGAAAGGAGAGGCGTTCCTCGGTTGCGGGTTCAGGTGCGGCTAAATCAATGGGGTTGGCGGAGTTGGTAGGCGTTCCTCGGTTGGCACAGTTCTGTAAATAAGAGATGTCTCATCCGGTTGAGCTGTCGCAATCAGTCTTTCTCTTCCTGGTAGCAGAACGAATAGGAGATCCAACATGACTGTATTAAGCCTGTCGCAATCAGGGTTAAGCTAGCTCATTGCCAGAAAACAAACAGGAGCTCTTATTTAGATCAGAGGACGCTCATCCCAGGTGCTTTAGCAACTCGACTGAAAAGGAGAGGTATTTCTTACTCGAGCACTTGTCTAGAGAGGAACGAGAGTATTTCTAAAAGGAGAGGGCGTAAGCATGATTCCGGGGCGGAGCCATATGACGCGAGAGTGTAGACTCTGGAACTCAGGGAGCAAGACCCTAAAGAAAGTTCAAGAAGCTATGAAGAGTGGTAAACTCTAAAAGGAAAGATGGAAACTGGGGAGTTGGCTGATAAAGATGGACAGTAACGATTGCGTAATATAAATTTATCGGCCTCGTCATCGAAAGCGGCTTCCAATTGCTCGGAAATTTTCAGCTATATGGGGGGCTTGGATGGTGAGCAAAAACTATTGATCAAGAAGTTGGTCAATTTTCGCATGAAAGAAGGTAAAAGAACGAGAGTTCGTGCTATTGTTTATCAAACTTTTCATCGCCCAGCTCGAACTGAACGCGATGTAATCAAACTTATGGTTGACGCCGTAGAGAATATAAAGCCCATATGCGAAGTAGCAAAAGTAGGAGTAGCGGGTACTATTTATGATGTCCCTGGGATTGTAGCCAGGGATCGTCAACAAACCTTAGCTATTCGTTGGATCCTTGAAGCAGCTTTCAAACGACGTATAAGCTACAGGATAAGCTTAGAGAAATGTTCATTTGCTGAGATACTGGATGCTTACCAAAAGAGGGGAAGTGCACGTAGGAAAAGGGAGAATCTTCATGGACTGGCTTCCACCAATCGAAGTTTCGCGCATTTCAGATGGTGGTAAAGTGAGACCACATAAAGAGCTCTTCGTCATTCAGTCAGATTATTAAGTAAGATATGGTTTGACCCTTTTCCTTTTTGTTTTCATTTTCATCTAGAAAGCCGGCCTTCCTCATACTCCTCCCTTCATTCATTGAGTTAGAGGAATCCATAGGAGGCCCACCCGTTATGCATTGCATGAAATAACCTTTCTTTTTTGTATGACTGAGAGGAACGCCTCAGGTCGAATGAATACGAAAGGGAGATCAATCAAAGAAACAAAAAAGGCCATGAATGAAGAAGTTGGGCCTTTCACCCTCTTTCTAGTTACTCTGGGAGCTGATCTGATAAATGCACTTCAAAGGGAGGGAAGGCTAGGTCTTTCCCATGTTGGTATGGCCGAGCATAAAAGATTTGAAAATGAAGTCAAAATAAGAAAAGGTAGAGAGAAAACTGAACGAAAGCGGTAGCCCCCGTCAGGGCAAGTGGTAAACTCTAAAAGGAAAGATGGAAACTGGGGAGTTGGCTGATAAAGATGGACAGTAACGATTGCGTAATATAAATCTACTTTTTTCCAGTTCCACCAATTTGACAGATAATGACGTTCTGCCAGGAGTTCCAGTTCTCGGCATGTTTCCTAATACGTCATTAGACGCCATAACGGAACACCCTTTATACTGTTCAGGTACAGTCGCTCTAAAAGAGGAATTAGTGGATAATCCCATTATTATGGGGAGTTTAACCCGGTTGAATCACTTCTTAATTAACATGCGCTGGGATTTTCAAAAGGGAGTTATTCAGTCCGAATATATTCTAAACCTTCAGCGAGAATTGGACCACACTCCTGCAGAACTCCTCAGCGATAAGCTGAACTTTATTTATTTTCGGGAATCCCTCAATTTATGGACTAGAGTGAATGAGTGGTATCTGCAGAATTTAGGCGTTCCTGGCCCTGCTAATTTTCTTCAAGAATATGAAGAAAAATGTTATTCGAACTATGTTAAAGTTATGGAAATTCCCACACCGCTGGAAGAATGGAATTTTAAATTTTTATTTAGTATTCTAGCTATCGGTATTTTCTGCCTATTCCTATTCTGTTGGATGAAACCTTACTTGCCTACCAGTCTTGAGCAGCAATCATCTTTGCTTATGCGGACGAAAGTCTTTCCACAGCACTCCGACAGAAGTCTCATATGATGACTCTGTTTACTCAAGAGCCTTCTTTCTCGATTAAATGCCTGCCCACTACCCCAAGCAAGCCGCATAGGTCTTTGTTAGCAGCTAGGCTTCTGTTTCTTTCCATAAGCGACTTCTCCTGTAGATGCCCAAAATAAAGCTTTCGCTCAAGGGATATCGCTTCATTTACTGCGTTAGGGTGGTGCCAAGCCCTTCATCTCTGGCTAAGGCTCAGTCAGATAGGCTCAATATCGGGCTTATCCGGCGAAGTCTCAAACTCTGTTGTGCCAGGGATATCAGGCTTGAGTTGGGACAGGTGCTTATTTCGATACCGCTTCTGTTGTTTTTGTGATGCTAGTGATCACATTTACAGTTAGAAGTAGGCCATGATTGATTAGCTAGAGTAGAGCTAGGTTGGTGCTGCTTTGCTTGGCAATCCTTGATCTAATTGAATCAGAATCCGCAAGATAAGCAGCCGAACGAGCAGAGCAGTAGAAGGACAGGTCATCTAGAATCCTATTCTTCCGATGCTTTGATCGGTTCCTTCTGATATGTAAGAGTTGACTACCCAGCACATCTTTGTTTGCTCGACTCGGTAAGTACAGCCGCTTCCTTTATTCTTTTCTTTGAACCATTACTCTCAAGGGACTACTCCGTTGGCCCAGTGACCCTTGGTCTTTTTTTTGTTGCTGTTCCCCGTTCCCTTTATCAGCTCTTTACCAGCTATCCGATTACCAATGAACAATAGCTATGACCGATAACCAAGGTTCGTAGAAACTCTATAAGGAGAGGCATAGACCGGTGGTCCCTTCCAAGTGCCTCTTTGTTTTGTACGAGCTGTTTCCAAGTGTTTTGATTAGTCGAGTCTTCTTCTGAGTCCAGCACTTTTCCTTTGTCCGATCGTCCGTCTCTTCCTCTCCTCTTGGTCGAGTTGCTAAAGCACCTATCTTTCTGATCCGAAGCCAAGGGATGTGCGATGTTTCTTTCTTTTGTTATTGTGCTAATTGCACTTTAGATTGAGAAGATAGGACTGATCGGTATCCATTGAAGATTCCCTTGCTTATCGGCATTCCTGCCCTGTGCACTGCTTCCTCAGCTTCGAAGCTTGGTTTGTTTGTCTCGAAAATTGACGACTTATCGGCTGTTGATTGACCGGTATCTGGTATGCTTCTTCTTCCTTACAGTCGACTGTTGCTACTTCGGACTACTACCATTCACTGCTGAGAAGAAGCTGTTGTTTAAGGAGTGGCACCGATCAGTGCCTTGTTCCTGTTACCAGTGTCTTGTCTTGGTTCCTTGTGCCCATTGTCTCCTAGTGTTTTGTTGAGTTTACTGTGTCGATAAAGTCCTTTTTATTCGAGGAAGTGTAGCCTTTTCAATTACAATTCTTTTATTCGTCCGAGTTTACTGTGCCATTTCGAGACTGTCCTTCATCCGAGGTATCGTGTTTATTGTGCCATTTATAGACATTGAGGACATTACTGTGTTGTACCAAGCACCAATAGTGTGAGGATTAAGCCGTTATTCTATCTTCCTTCCTTCGCTCTAACGGGTGGATTCGCTCTAACAGGAGGATTCCGCTCACTTGGGGTGGAGTAGCGTATGCACCTCTCTTTCTTTGTTGCCTCCGGTGTGCTACCGAGCCTTTCCCAGGTATTTACTTACTGCCGGTTTACTCCACAGCATTGCCATCTTCTTTGTTTTGTTGGTATGAGCTGATCGGCCCTCTCAAGCGTAAAGTAGTCCTCTGATTGTGTTGTATTGGCTCCTTAAGCAACATTTGTTTACTTATGACCGGGTGCTGCTTATGCCTAGACATCTTCCCCAGGACTTCTTCTCGAAAGACGTTGACCTATGGTAGGTGCTGAGTCCAGCACAGTTGTTCCTTTGTTACTGTGCCATTTCGAGACCTTCGTCCGATCATTGTTCCTTTGAAGACCTGTCTTTTATTCCTTCTCTTTCCTTGCAGTAATTAACTCACATTTATGGTAATGCTGATGCTTGGCAGCTTCACTGTTCCTTCTTGTTGTGCGTAGGCTTGGCGTTGTTTACTCCACCGTGCTATCTTTGTGCTGCTAATCAGTATAGTATATTCTTTGCATACTGCTATCTTTGTGCTAATCCATTTCAGTAAACGGGAAAGTGGAGGAGTAATGATTGGTTCCATCAATCTTATTATTTCCTTTGTTTTGGCTTGGGTTTCTTTCATACGAGTAATGCTATTTATCAATCTTTGTGTTCATCAGTAGACTCTTCAGCTCCTGGCTCCTGATCAATCTTCCACTCTGCGCCTGCTGCTGATCCATGTCATTCAGATGTTCCTCGTCCTGTTGATAAAGCACCTTTGCACCTTTTCCTCGAGGTTGGGGTTTCGGTATGCTTACAGGGGTTTTAGGGCGGAGTATGAGCGTGTTGTTTATTCATCGGCACCTTGACCTTGACTAATCGACGTAGAGCGTCTTGGCTTCTCGTACGGCTTGGTGCTGTAATCTTTCTTTCCTTGGTCAGTGGATTATGGTTCAGTACAGATAGTGATAATGCTCCAATGGTTACTGCACCTGTGTAGGGAATCTTATTTCTTTTTCTTTCTTTCGTAATTAAGTCCATTTTTTCAGTCGGGGAAGGGAGGGAGAGTCCTGCTTCGGACCTGTGACCTGCTTCGGGGCATAAGTGTATTAGTGCTTCTGTGTACGATTGTGCTGATCAGTCGTTCGTGCTTTTGCGATAATGCGATAACTAAAATAAAGAAGAATGTGAAAGTATGAGTTTTGTACTTGAGTGATGTGCTACTGATCAGTTGTGTGAGGGCTTTTGTTTCCATAGAGCGTGTGCGAAGTGTGGAGTTTCGTACTTGACTCCATCCCGTGATCAATGTGCTAATCATTTCAATATTGAGACTCCATCCTGCTTCTGCTCCTGATCCTTTGTGCTGCTAATCAGTTTTGTATGCCAAACTGCTCCTTTGTGCTAATCATCAGTAGAAAGTGGAGGAGTAATGCTTGGTCAAAGTGCTGTAGATTTGTTTCGGTACGAGCACTGCCTTTGTGCTAATCATCATTTCAGTATATTCTTTCTTTCGTGTTCCATCCTGATTAATCTGTAATCTATGTTTTGTACAGCTGGCTTGGGAATCATCAGTAGAGTCTTTCCGCTCCTGATCTGTCAACTTTCTTTCGTACGGCTGCTTGGCGTGCTGTCATCTTTGCTACTCCGCTTCGTACGGTTTTGGGTGAAGGTTCAATCTTTTGTTTCGTACGAGTCGGATATGTTTGTGCGAGAAGAAGGACAGCTTTCGTAGAGAGTCCGATCAATCTTCCACTCTTGCACTTGCATTCATTCGGTTCTCCTCTCAGTTCCGTCTAAATCCCCTCGTCCTGACTAAAGCACCTCTCCGTGTTCCGTCGATCACCTAGATAAATTAAGTATTAGTAATAAGAATGAAAAGTATGGCTTAGCGTGTTGGATCAATCAGTCATTGCTTTAATCTTGAATTAAGTATATAGAAAAGAAGTGTTTGTTGTCTGTGCCAAGTCTAGAAGTGGTAGTATTAGCGATCCTATTGAGGTGTTTATCGGGTTCGCTGCTCGAATTTCAATAAAGTCAAGTTTCCTCAATCCCGTTTCTGTTGAGTTGCCAAGATTTCTCAATCCTTGTATTAGTTGATCCTTTTGTTTATTCGAAAGATCGGCGGGATGCTACTTAAGGTAGGGTACGGGCGCTCTATCATTGTCTGATTTTAGGTTTCTGATCGCTAGCCTGCCGGGCCGCCCACGCGATCAAACTATCAATCTCATAAGAGAAGAAATCTCTATGCCCCCTTTTTCTTGGTTTTCTCCCATGCTTTTGTTGGTCAACAACCAACCACAACTTTCTATAGTTCTTCACTACTCCTAGAGGCTTGACGGAGTGAAGCTGTCTGGAGGGAATCATTTTCAATTAATCTAATCATGCCTCAACTGGATAAATTCACTTATTTTTCACAATTCTTCTGGTTATGCCTTTTCTTCTTTACTTTCTATATTTTCATATGCAATGATGGAGATGGAGTACTTGGGATCAGCAGAATTCTAAAACTACGGAACCAACTGCTTTCACACTGGGGTAAGACCATCCAGAGCAAGCTAAAGCTTGGTGGAAAAGATCGTACAAGTAAGTTCGGGGTCTTAGCGTTCGCCACGCGCTATTTCCTCATGTTCGTGGTCCCAAAAATGCGGCTAGCTATATATCTAATATATGGTTTGAATTTTATTTTTGGGATTAAATGGGGGTTGCTAGGAAATGAGATATTTCAGTTCGGCGTCGGACCAGATGGCGTCGCGCCCCCAGCTCTAGATCTCAACGAGCGCCCGCCACTGCATCTTTTGTACGCGGATGTTGAGAGTTCCGACTCTCAACAAGCGCGGAATGCTGATATGCTAGCGCATATTAGCCGAGTGCAAGAGATAACCCGTGACCTAGAGGGTGAGCATGATATCGCGCGGCGTCAAGCCCTCGTCGATATCATGAAGTGGGAGGTCAGGAGCTTGGATCACCACTTCCGGGTCTTTCGGTACCTAGACCGTCTGCGAGATTCGAAGAGAGCCAAGGTGAACGAAATACTCGATCTATTTCGATGAAGGGGCGGAGCCGTATGACGCGAGAGTGTCACGTACGGTTTCTTTGAGAAGGGTGTGATACCACCACCTATCAGGCCCGCCTTACGTTACTAAATTCGTGGCTCAAGGTGATCATCCATACACGGAATCACCAACCCCAAAAAACTAAAGCTTTCGTTCTCTTCTTTTTTTTTTTACTTGATTGGCAGGTGTACAACAACCTTAACCGCGCAAGCCTGGGCTGGGCCTACCTCCATCCTTAGAGGAGCCGTATGAGGCGGAAGCCCCACGTACGGTTTTGAAGCCGAGCCTTTCCAGCAATGGGGCTTAGGGACCGATATGATGATTGGTTTAGGTAGGGCGGCCGGCCTACTACGGGCACCCGCCTGTAGGGATTAGTGCGTGAGACCGCGATCCACAAACTGACGCATGGGACTCACCCTTTACTTGAGAATAAAGAGGGGAAAGATAGCATGTCCCAAGAGCGAGGCGAGTTTTGGAACCCTACAGCGAGAGGGACGCCTCGCGAGCCGGGCTTCCAGAGATGAGGCCTTTTGGCGAAGCCAAGTTTCTTTCGGGCCACCAAACCCTGCAACTGATGAGAAAGAAGGCCCTATGGGGTAAAGGGAAAAGCGTGTACGTTGTCACACTCCCTGCCTTCCAAAGGTGCCTAGAGGACGGGCCAGACGCAGCAGAGCGACAACCCGGGAGCAGATTCCCCACCGGCAGGGGGACAGGAGACGGCCATCTCAAGGCACATCACGACCTACAGGTAAGACCGGCGAGACCAGGGAAGGCAACCCGATTGGGAGTCAGAGGATCCATAGTACCTGCAGCCCCACGGACTTAATATTCCTCATTTTAGAAAGCGGAGGGAAGGGATCTCTTTTCTGCAACGGAAAAAAAACGGAGCAGATTTGACTCGGCACAACCTAACGATACATCCAATACCAATGATCTGTGCCTAGAATGCGTTGCTAGATCTCTGTTCTAAAAAGCTATACAGGGCAACAAGAAAGTTGACCGGGGGCGGGTCTTCCCTTTCATTACTTATTTCACGACGAGGGAACCGCGCCCGCATCAGTCGAAGTGGTGCCCTCCTCTAAGAAAGAAGGTTTCAGTCTCATTCAACCAACTCTCTTTTTGAAGCAGATAGTTCACGAGATAATTGAGTAAGTTAAGATAGATGTCACAATTTGAACCAAAGGCCTTTTTAGTGATTAGTCTGCTAGTTTCTTTGATCTTAGTCGGTGTTAATTATTCCTTTTTTTTTTCTTTTTTTTTTCTCAAGTCAACCCAGAAGGAGAAAAGACCCTAATTTTTCTCTTATCCATCGTCTTCTTGGTAGCAAGTTTTTTTGTCTTTGTTGTTAGTCACCAAGTTATTTACCCAGAAATAGATAAGGGGGATTTAGTAAATCCACTTCAGGTGCTGGCCCAGCTGTCGTGTCTATTGGTTCTCTTTAGCGGCTCAAGAGCCGCTTACGCAGGCGCCAGCACCGGCTGAAGTTGCCCATCCTGTTCCTAACCAATCTCCTCTTGGGGGGGACGGGGGAAATCGCTAGATCCCTCATGGGATCAAAAACAACCTATCACCGAAGTTCCACCTATTCAAAAAAATGGTGCCTGAACCGACGGGAAGCTCTTCTTCGCAGGGTCAGGGCTGCCGCGCCTTGCCGCAGCAGTGGGGCCTACGGTCGGACAGGTGGGACGTGTCTGTTGTGAGGCTTGCGGGGAAGCTTGCCTGGATATCGCCCGTGCCTGTTGTCAGGAATGCGCGCGTGTCTCCCTAACCTAAATCTCAAGGACTTCAGGTGTTCGGTCGAGCTTGTCATCTAATAACATTTGAATTTCTCTTAGATTACACGTTCCCGAAATGGATCCTATCAAATATTTCACATTTTCTATGATTATTTCTATTTTAGGTATTCGGGGAATCCTCCTTAATAGACGAAATATTCCTATTATGTCAATGCCAATTGAATCAATGTTATTAGCTGTGAATTCGAACTTTTTGGTATTTTCCGTTTCTTCGGATGATATGATGGGTCAAGTATTTGCTTCATTGGTTCCAACGGTGGCAGCTGCGGAATCCGCTATTGGGTTAGCCATTTTCGTTATAACTTTCCGAGTCCGAGGGACTATTGCTGTAGAATTTATTAATAGCATTCAAGGTTAACCATTCCTCTCCTTTCAGTCGAGACTTCTCCTGGTGGACCTTACAGTCGAGTCACTTTGTACCTCTTCTCAAATTAGGAAAATACGAAGTTCGAGGAACGCCTTTCGTTCTCTTTTTTTCTTGGTCGGCAGGGTCAGGGCCTTTCTCGCTGGGCGAGCGCATCCGATTCAAAAGTCCTTTCCTAAACCACTTCCCGTTCAGTTGCTGAAAAAGAAATGGGGATAAGCTTTCTAAATGAGATTGAGTTCGACGAATATGGATGCTAGAAAGATGTTATTTGCTGCTATTCTATCTATTTGTGCATTAAGTTCGAAGAAGATCTCAATCTATAATGAAGAAATGATAGTAGCTCTTTGTTTTATAGGCTTTATCATATTCAGTCGTAAGAGTTTAGGTACGACTTTCAAAGCGACTCTCGACGGGAGAATCCAGGCTATTCAGGAAGAATCGCAGCAATTCCCCAATCCTAACGAAGTAGTTCCTCCGGAATCCAATGAACAACAACGATTACTTAGGATCAGCTTGCGAATTTGTGGCACCGTAGTAGAATCATTACCAATGGCACGCTGTGCGCCTAAGTGCGAAAAGACAGTGCAAGCTTTGTTATGCCGAAACCTAAATGTTAAGTCAGCAACACTTACAAATGCCACTTCTTCCCGTCGCATCCGTTTTCAGGACGATCTAGTCACAAAGTTTTACACCTTAGTGGGTAATCAATTTGCCTACTCTTCTATCTCGAAAGCAGAAAGAGTAGAATTCATTCGAGAGAGCTTGGTGGTCTTAAGAATGGTTCGGGGGGGGGGTTTCTCTTAAGAATAAAGAAGAGGAATTGAATCTAATTGATGTTCATGCTCTCAGAAGAGCGGATCCAATACCAAGACTACTTCTTTCTCAGGAAGTGCAGCAAGTACTTGAGGAATTTTGGGATATCATGCCCCACGAATTAGTTGCCAAGTGCGCCCTAGTAGGGCAGTCTACCACAAGATCGAGTTGGAGCCTGGAGCCAAACCCCCAACTATTTAGACTGGGGCTGGGTTAAGCGGATGGCCCCCCAACTAGCATCTATTAGTTAAGGGGATTGGTTGAACTCAGGAATCAATTTCAGGAACTCATGGAGGCCTATTTTGATTCCAGCCTCCAAGGCAGTTTTTCCCTATATTGATTGCTGCTGGGAAAAGGTTGGGGAGCCCCTTTCCAGAAAAAGCGGGAGCCTTGTGCATTGATTATCGGGCGCTCAACAAGGTAACCTAACTATCAAAAACAAGTATCCACTTTTATTGCAGACTTCTTCGCCCGCGCGAGATACTTCTCGAAGTTGGATCTACGGCGGGATACTACCAGGTGCGTATCGCGGAAGGAGACGAGCCAAAGACGACCTGTGTGACAAGCAAGCAACCTTACTAATAAAGGGGCCTTTTCTTGCTCGTTAGCGCCCCCCTACCCCTATTATATTAGTTAGCCCTCCTTCCTTTACTCTTGGTACCTAGACACTTTGAATCAAGGCTACGCCCCAGGTCCCTTAGTTCAAGCTCCATCCCACATCCATTTGCCCAAACGACGAATAAGCTGCGAAAGAATGAGCTCCTGTCGCAATTGAAGAAGAATATGCTGCTAGTCTTTTAGCCACAGACGCTCTTGAGTCAATCTCAGCTGTGGTCCTATCAGCTCAACCAGTTGCCGGTCTTGTTTGCGTAGTAAATGGTTTTGGCGGAATACGGTGTTTGAGAGTAAGCTGCTATTCAAAAATATGGTCTGTCTGTAAGCAATTACCTTTCCTGGCTTGCTTTAGTCGGTGACTCTATGCCCTCTGCTTTTCAGTCGAGTTGCTAAAGCACCTCTCGCCAAGACGTCTTTTCCAGTGTCGATCATCTTGCTTTCCCGAATTCGTTAGATTGCCGCCAGTTCCAGGATTGGGAGAACATAGGATTGGTAGTTGGGCTCTGCTCGCTTGGTTGGTCATTCCCTTCTGTCTTGGTTGATGGATAGGTGAGCGCATGCGGTTACTTCCGAACGAACCGGTCGATGGCAAGCTTTAGCTTAGCTGGCTAGCGGAACAGAAGATGGGTTAATGGGCTCTTAAGGCTTGGGATTTGATCCACTCGATAGGAAACGATCCTCAAAGAAGACACCTCTCCCGCTTCTGTGACTCAGCTTCATCGTACAAGGGACTTTTAGCTCATCATTCCACTTCGACTGGTAGGGGTTTTCTATTAGTGCACTTCGGTGGGAATAAGTCTTCCTCTTTTCTTTCGGAAGGAGAAAAAGATGCTTCATTTGGTTCCCTCGGATCGGTAAATCCGAATTCGTGACCCAAACTCTATCTAGTTTAGTTACATCAAAGGCGGCTGCCGAATAAAAGGGGAAACTTTGCCTGCCAGATTGCCTTCGGTAACCCTAACCCAGTTATCAAAAGAAGGCGCCAGTTCGGCTTTCAAGAGAGGGGAGCGAGCTACACATAGTGAAGCGAGTATTCGATGAACACGAAGGCTTTGATGAAAAGGAAGATTTACATTTAAAGTAGGTAAAGTAGGGCGCTTAGGCTGGCTGAAAACAGATAAAGGAGACATGTGCTTGGACGAGTTCCTCCGAGGAACGCCTTAGCGGGGTCTACTTTCTTTTGTTGACGCAGATTGGGCAGGTTGTCCTGACACTCGGCGCTCTACCTCTGGCTATGCTATTTTTCTTGGTCCCAATCTTATTTCATGGCGCGCCAAGAAAGAACCTACAGTCTCTCGTTCTAGTGCTGAGTCTGAATACCGCTCGCTTGCCTTTGCTGTTGCTGAGTCTCACTGGATTACTCAGCTACTTCGCGAGCTTTGTCTCTTTCTTCCTCACCCTGTTCAGATCTATTGTGATAACATCAGAAAACTTATATGACTGCCAATCCTGTTCATCATGCTCGCTCGAAACATATTGAAATTGACTACCATTTTGTACGGGAAAAGGTTGTCAAAGGTGACATTATTGTGCAATATATTCCCACATCCGAGCAACTTGCTGATATCTTCACGAAGGGCCTCCCCTCTGCCCAATTTCACTACCTTCGCTCCAATCTTCGCATCCTTCCACCTTGCTCCGATTGAGGGGGAGTCTTAGTGTATCTAGGTTATACTGTTAATTGTGTCGTATGTATTAGCCCAAGGGCTTAATTGTAATTTGACTTGAAGAGATCATCTATATAAATAGACATCTGATGCCCTAATTAGGGCTAAGCACTGAAATTTGTCCTAAACCAATTTCAGAAACATTTTGTTATGGTGGGGAGTGAAAACACCAATGCAGCAGAGAACGACCGTATTAATCGGAATCTACTTATATTAAGACAGACGACCGAGAAAGAAAAGAAAGGCTCCACGTTCTCCAAGTGCTTAATCTTAGCGTGCTAGCCGCTGCTTCATTTCGTTTTCGTATAGTGAGAACGCTTTCTCTTTCTTAGCGCTCCGCACCCTTGTATAGTAAGGGGAACTCTGGTTGCGCGGCTTTCTCTTATAGGGGTCTATTTTCTCTGACTTGACTCAGCTTGACCTACTTGACTCAGCGGTTAGAGTATCGCTTTCATACGGCGAGAGTCATTGGTTCAAATCCAATAGTAGGTAAAACCGGCCGAAACCCCTGCTTTTTCCAGCATGACAGCAAGCACGGACTGGCAGCAAGGATGAAACTGAATGACCAAAGCATCGGTTGCTTCCACTTGTGGCCTTCTTCGACCGCCTTGACACCTTACTTAATATCAAGGCAGCCCACATACATGAAGAGAAGAATTGGGTTTAGCGGTAATGGCATAAGCCAAAGCTAGATCACCGGAGTTTAGGCAAATGGGTACGAGAGAAAAAGGTTTTGAAATGCATCTTCCGAAACCAGATAATCAACCGAAGGAGATTCCCCTGCAAAAAGGAGATGGCTCTAGTTTCATACTAATCTTCGATCTTCGAGATTCCCCCACGGAGCGGTAAGGCATCCCAAGGAGCGAAGCGGCTCAAAGGATTGGCAATGAAGCTCAGTCTGGTCTGAGGGGATGGGCTAGGCTACTCTCCCCATACATAGGAAGAGGGGGAAGTCAAGTACCTAGATCCAAGATCACGATATCCGCTTTGGTGGTAATATCCGGCATAAAAGAAAGCACCAAAGCAAACAAAGTCGATTCTTTGTTGAGTCAACTGGCAACTGGGATTGCAGCGGATCTAGTGGTTCTCCTATTCTATGGTCTTGCTGCTGAGAGAGTAAGAATAGAAAGCAGAAGCTCGCCCGCGGGCGAAAGACAAGGGATGATTCTTCTAAGATACGAACTTGCGGAAGGAATGAAAAGGTATTCGACTAAGACGGGGACGGGTATTCCCGAAGATGCTTGACCTAAGAGATAGAGACGAGAACGAGACTAAGAAAATCAGATATTGGAGAGGAAAGAAGCAGTGCGAGTAGAGTAGAGCAGCTTGGTAGCTCGCAAGGAGCGAAGCCTGCTTGACCAACGGGAGAAGCCAACTCCGTTCCTCCAGCTTCGCTGAAGAAGCTAGGTTCCTCCTTGATGTCGTAGGTTCAAATCCTATCTCCGCACTAAGTAAGGGTTTCATTCTGCATCACTCTCCCCGTCGTTCTCGACCTCGCAAGGTTTTTGAAGCGGCCGAAGCGGGAAGTGACAATACCGCTTTTCTTCAGCACATTTTGGATGATTTGAGCGAAAACGGAGTACAAAGTTCAGCCTTTAAGGAGGCTATGAATCAAATAGGGCTGGTGGCGCAGTCCCCACTTGACCAATTTGAGATTGTCCCATTGATTCCTATGAATATCGGAAACTTCTATTTCTCATTCACAAATCCATCTTTGTTCATGCTGCTAACTCTGAGTTTTTTCCTACTTCTGATTCATTTTATTACTAAAAAGGGAGGAGGAAACTTAGTCCCAAATGCTTGGCAATCCTTGGTAGAGCTTCTTTATGATTTCGTGCTGAACCTGGTAAAGGAACAAATAGGTGGTCTTTCCGGAAATGTGAAACAAATGTTTTTCCCTTGCATCTTGGTCACTTTTCTTTTTTTGTTATTTTGTAATCTTCAGGGTATGATACCTTATAGCTTCACAGTGACAAGTCATTTTCTCATTACTTTGGCTCTCTCATTTTCTATTTTTATTGGCATTACTATAGTGGGATTTCAAAGACATGGGCTTCATTTTTTCAGCTTTTTATTACCCGCAGGAGTCCCACTGCCGTTAGCACCTTTTTTAGTACTCCTTGAGCTAATTTCTTATTGTTTTCGCGCATTAAGCTTAGGAATACGTTTATTTGCTAATATGATGGCCGGTCATAGTTTAGTAAAGATTTTAAGTGGGTTCGCTTGGACTATGCTATGTATGAATGAGATTTTCTATTTTATAGGGGCTCTTGGTCCTTTATTTATAGTTCTTGCATTAACCGGTCTGGAATTAGGTGTAGCTATATTACAAGCTTATGTTTTTACGATCTTAATCTGTATTTACTTGAATGATGCTATAAATCTCCATTAAAGTTCTTCTTTCTTTTATTTAGATTTATAATTGAACAAAAGCGAGGGATGAGAGTAGTGTTATTTAGAGCAGTTACACAGCCCCTCTCCTTGCAGTCGAGTGACTTCGCCCCTGAATGTCTTAGATAGCTGTAAGTGAAAGAAGGGTACTAAGTAGCTGGGAATGCGGCTAGCTAGTACTTACTTGTTTGTACTCCCCAGAAGCTCCAAGCCTTAACTACAATCTTCTTCGGTGCTCTTTTTTTCTTTTTAGAAAGCTTACCGGGGCTTGAGAGTCTTTATATTTATAAATGGATACACGGGTTAGACCTTCTTCTTTTCATTCTTGGTCTTATTGTTTTCGTTGAGAATGAGAAGTCATAGAAAAAAGGTCTTAGTCCTAACTCGCACAGAGAAGGATTCTAAAAAGCAAAAGAAGCGTGAGTCAAATTAATTGATATGGAGTTGATTTCCGAGGGAGGGAAAGGCAAGTAGTCTCAGAAGCAGGAAGCATAGGAAGCAGCAGTAAAAGCAGTCCGAAAAGCAGTGAAGGATGGCAAGGGGAGAATAAAGACTATGAATGAATGTTGAATGGGAACTGGGAGCTCCTGAGTGATTCTGCTTCACTAAAGGGTCAGAAACTCGATATCAGTGTTTCATTGTCGCGGTCCGGGATCCCCCGAATCATTCCCAAGATCCATCGTCATAGAATGAAGATGCGGGGTGATAGTTTGACTATCTTGTCAAACTCTATCTCTCTTGGATTTTTAACAAAGGAAAGCCCCCTACCCTAATGCCATTTGATTGATTAAAGTTCCGTGCTGAGGCGTTCCTCGGCATCGTCGTCGAGCTGCCGAGAGAGATCCTCTATAAGTCAGTCTCTCTACGTAACCCCAATCGGGAGCTCTCAACTAACATCCATCTTACCATACCAATCAATCTGATAACAGCTCACCGGAAAACCTTCCGGAAAGCCTAACCAGACTGAGAAAGGCATAGGTTTTGATTCCTGGAGAGAGAGCGACAAGAATGAAACCAGTGACGATTGGTTTTTGGGTTAATGTTACAAGGCTGACAGGAATACCTCCGGTAACCAGCCCCTCTTAGTCTCAAATAGGTCTGAATGTTATCACACCCTATTTGTTTGCTTCATCGCCGGCATTAGAGCCACAGCGAACCGACCTACTGCTATCACTAAACTAGACAAAATTACGTAGTAATTCTTCGGGCACTACACAGTATTGAGGTTCCGGCTGAGTTCCACCAATGGACGCCCCAGTTAGCTGATCTACGACCACCCTAGTGTTGCGCAGTATGCGTATTGGTTGATCTCTTGAAGGATATGCCCAATCGTATTTGGCTGGGGATGGGAAAGTTTGGGCGCTGGCAACGAATCATTTACGAAAATCCCCCGATCTAAATCCTTCTTGTCGTATGAGAGGCTACAGCCTAGATGAATGCAACGCCAAACCTGTTTTAAATAAAGGCAAAGAACCTGTTAATTAGCACGGAACTACAACAATCCACAATGGTTTCTTCCAAGTCAACTCAGAAGTTGTTACTCACTCATCAAACCAAGAAAGCGTGTATAGCTCATCAAACCAAGAAAGCGAGTATAAGCTCATCAAACCAAGAAAGCTAGGCTCTTTCTTTGTTGCAGTTGGGCAAGCTTATCTGTTTCAAGCAAACTAGGAGGAAACTGTTATCAAACAGAGAGGGGTGGGCGTTCTTCTTAGCAAGAATTATTGCAGTAGGATAATGGCTAGGAGGATTTGAAAGGCATTATGGCATTAAGATTTCCAAGGCTTAGCTCAGGGACTGCTGTCAGCCGCTTCCCCTCATCAACTAATTAATGAGAGCTTCCCCTGTAGTCGTCAGCTCGTTCTTGACAGATCCGATCGGGTGTTCATAATCTGGAGTAAAAGGATTCGAACCTTTGCATGCCGGTACCAAAAACCGGTGCCTTACCACTTGGCTATACTCCATACGGCCTCTGAGTTTTGGACGGTAAGGGGGAAGGGAGAAGCAGGGCGGGGTTGCGCGCGAGCCATGCAAGAACGCGGGAATATAGCAAGTAAGCAGCAAGGTTCTTTAGGACCGACTACAACAAGCTCGCGACTCTCATAGAAAGAAAGGGTAAGCTCTCTGCTCTATTTGCTCGCAATGCTATACCTATCAAAGTTGGCGAACGCTTCTGTAACCTTAGACTCGTTACGCTCTTCGACTGAACTCGTTGGCTCCGCGTCCACCGAAAGTCGGTAACCTTCGTCACCGTCAGCATTTGGTACTCTCTCGATAGCTTCAATAGTTCACTGAAAGCCTTTGGTGTAATGAACCGCAAGCCCTTCTGAAAGAAAGAATAAGAAAAAGGCTTGGTTGCGGGAACCGACGGTGACGAAGCGGGCCGATGCGGCCGTTTCCCGGGAACCGGAAGGTTGCAAGGTTCCCGGGAAACGACGTTCCCTTTGTAAGGTAGGCCTTTTGATAACTAATTAGAGTTGACATGAAATGGATCACGGGAAAAGACGTATCCGATGAATGAATGATTCAATCCCTTCCCACTCACACGGGTTCTTCTATTGAAGGGCTTCCCCTTCTTCTATGTGAGGTGGGGAAGGTCAGAGCGGAACCTAGATAGAACGCGGAGCGCCGGCCCCTTAGCCGATACAGTTACCATGCTTACCAGCTCTTACCATTGCCGCTTCTAGATGGGAGGTAGGCGAAGGTAGCTTGCTCGAGGAACGCCTAGGCTACCTCCACTCCACATGTTATTCGCGAAGAAAAGGGAGGAACCCGGTTCTTCGCTTAGTAGAGCTACCGGCCGGCGTACGACGACCGCTTCTTGTTCTCGCCCAGCCGCTTCTTTTGATTTGATTATTATTTGAAATCCTAGACTAAAGAAGAAGCTCCAGAATCCGCGCGGGGCAAAATCTGCAGCAGGAGAAGAAAGAGGGTCCAGGTCAATTTGATAATGAAGCGAAGGTCCCCCTTACTCCCTATTCCCTCTTAAAGCTTTATAAAGCTGGGCGGTTGGTTAAGAACTACTGACTGTAAACCATAGATAGCAGTTACAGTCACTCAATTGAAATGTTCGCCTTTGGAATGAAGATGGATGAGCAGGGGAGTTTTCACTCCTACGTGGTATTCTCTTCCAACAAGGGTTACGGCTAAAACACCGCTTACTTTTCAAAGACAAACTGCTCCTAAACCAAGCCTGAACACGTTTCCAGTTGTTGATCGACGCGTGTCGTGATCACCAGCATAGTCGGCGTCACAATAGCCAACTATCTTACACTGTTCTCCTTTCTTATACAGAAGACCATAGTCAAGGAGGAACCCCTTTCATGTACCTCAATATTCGTCGAAAGTGAGGTTTCTTTGGATTTTGCATGAATCGACTAACCACTCCAACTGCATATGCGATGTAGGGCCTTGTCAGGGTTAGCGCTTGTGCTAAGCGATAAGAATTCGTTCTGGAATGCCGGCGCGGACTGAAGGATGATGTTTATTAGCCAGACAAATCCTTAATTCGAAAGACGACAATCAAAGCATCACGACTCGTGGAGCATTCATTCCTAAAAGGTGTAAACCTGTGAGATCGGTAGACAACCCGTAAAAGGAAGCCGCTAGGCATCAAGCCCTATTATCTTACACCTAGGGAGGGTGGCCGTTGTTGGGTTTTTCTCAATTAGAGTCGCAAGGAGTTTGCTGCTCGTTGGTAGTGGAATGCTGACTAACCGTCTCCGTCCCCCTATGATGAGAAAAAGCTTTCCGAGTGGACGAGGACCGATCTTCTCTTTCTTTAGTCTTGCTCGTCTTATGGCATTAGCCTTTGCGGATTCCTTAATCCGCGGGACAGCTGTCTCGGCACATGTCAATCCTTATGTCCCCAGCGAGCAGGTCAAGGTACTCTATGTTGCTGCTTGCCTTTATCTTCCAGTCTGCCAAGCAAGCTTGTTTATTCTATGTTATTGATAAATCGCTATTTCCCCCAAACCGCTAGTTTTCTCATACGACTCAGAACTTCTCACGTTGTTCTGTCTCCAAACAGAGAGCTCATAGGCCTTATTCTTTGGATAAAAGTAGCGACGAGCCGACTACTACGACCACATGCGCATCTAGCGCAGTGGCTTGTCACTTCGTACCTTGACCATCTTCCGAAGTTCTAAATAATCTACTGATCAAACGCTGTAGGGGCGGACTGCTCTACATTCAGCCACGCCACAGTGACCCCCCGAAGCGATCTGCCTCATTGCAGGACGAAATCCGGCAGCCAATTGCTGGCTCTGAATAACCAGCCCAGCAAGAAGTTCAATTCTTCCATAACATAACGGGGCGGGGTTGCGCGCGAGCCGAGTGACGTAGATGCACAAGAGTACTTCGCGCCACAACCATTTCTTTTTTATACGTTCTACGGACCGATGCCTGCTGCTTCATCTGGTAGAAAAGAATCATAGATATGCCGGTCATTAGAAGGAAGAACCACCATAAAAAGATTCCTCGTGTATCATCTGTAGCAAAACTATGAACGGGAGCTAGCAATCCGGACCGTATTGAAAAGGTTCCTAAGACACAGCATGGAAAAGTCACAATATTAAGAAACGAGGTCCAAGAATGAAGAAGGGGTAAAATTACAGAATGAATACGAGCTGTGGCTAATACCCGAGGCATAAAAGAAGCATTTTCTACGGGATCCCGAAACCACCAGCCACCCCGACCTAATTCATGATAAGCCCACCAACTTCCTGGCAAGATGCCTACGGTTAAAAACCACCAACATGTCAAGATCCAAATTCGAATTGGTTCCTGGTCCTGATCAGAGACCACTGTGTTCGCGCCGGCGGTCCAACAAAGAGGCGAAGTAGTGGTCTCTTTCTTTCCATTACGAACGACACGCTTCGCCTGCTCCCTCCCCGTGTCCACTAGCGCTCCTGCCCAGAGCAAAGAAACTACCTTTTTCGTAGATGAATTAGGTTCAAGTTGATGTTTTTCACAACTCTTTCATAACATGAAGTAGATCTTATGTCCGGGCAGTTCAAAATTCTCTGCAGTGACCTGACAGAGAGAAAGGCTGTACGCGAATCGGGGCTAAAGGCGGCCCACTTAATGGCTTCCCTTTGTTCCAAAATGGAGGACGAAAGGTAGCCATCGCTACGTAGAGCGTCCTGTAGCTTCACTTCAATATCAAATTGAAGATCCGGTATTCAAAAGTCTTCCTTGGTTCCACCCTCACATTCATGAAAGAGATTGCCGAAAAGGGCTTTATGCAAGCAGAAAAACGACCTTTTTGACAATTCACAAGTTGTAGCTTGACCCCGGTTCGGGACTTCCCAAGTCACTCCTATAAAGATCCCCGGTTCTACCTCCTTCTGCAAGAGTGATCTATCCCGTCAATCCCACCCGCGAGCTATACATTCTAACCTTCTTTGCCTAGCAAGATACGTCTAGTTCTCCCTCCGCTTGCCTATCTCGGTAGGAAATTTGAACAGTCTCCGATTTCAAAGGAATTCCTCGGCAAGAGCTATACCTTCAGGAACCGGAGCAATAACTGCTATAAAGGAAGGCGTTATAGGACAGAGGTGGCGATGAAGATCATGGAATCATAACAGCACAGAAAGCTCCGAACTACTTCCGCTTGAGGTTGGTCGTAGATGAACCCAATAGATTGACTATCTTCAAAAGTAGCCGCTAGAGCTATTAACTATCGGTATTACAGAAGAAGCTAAAGAAGAGCTCTGATAAAAATAGAATTAAGAGTAAGATCAAAGCTCCCGGGGATCAAATCTCCTAGGTAGGTGTGGCTCGTTGAGACCTGAAGGTTCCCGTCGGCCAAGGGCGTAAGAGGAGCATGCTTTTCTTCGTCGTCAGCTAGCGGGCGCAAGCAGAGTGGGACCTTCTCCGATAGTCTCTTTCATCATAATATGTGAAGGCTTTCCCGCTCGATGCTTTCTTAATGTCATAGTTTATATCAAGATGAAAGGGAAAGACAATAAGAAAGATTCAAAGGTTTTTTTTACCTAGATAGTCTTTTATGTAGTAGACTTTCTCGAGGAACGCCAGTCTCTCGACTGAGAGGAACGCCTGGTAGGTGGTGAAGGACGAGAAAGATACCTGGACGGAGATGTGAATTAGATATAAACCTTCTCATCACTAAATCGATCAAGTACTTACCTGAGAGTGAGAGAGGTCATATTAGTATTCTCTGTATATTCTCATAGTGACTTTGAAAGCACTCTAAGTCCCGTGCGACTAAGAATAAGAAGAGGTGATAGCCCGACTGCTTGATTCCGATTTACTACAAGTTTGCAACTAAAGCCACAGAGGAAAGGTTAATTCCTTGTAGGGAAGTTGATAGGCATTTTCTATCTTCCTGAGCATACGCTCATTCTTTCTCTTATATACGCTAATTCTTTCAAAGGCCTATTTGATAGCTTCAAACGCTTGTTCTTATCTTTATCATACGAAGAAATATCGATCGTCAAAATGGCATAACTATCTCTTTCTCTTTACTAGGAGCTGCTTGCCCAGTCATAAAGCTAGGATCAGTCTCTGTCCACTCTAAGGAAGCCCCGTCTCAGGCGAAGGTGGGAAATAAACAAACAGTTGTTACGTCGCCTTCGATTAAATCGGTAAACAAACAGGGGGAACTCTGATTCTCGTACAATCAACATTTTCTTGTTTAATTCCGTCACAAAACCCCGTGTTTTTTCGATGAATCACTAGTAACAGGAAGATTAGGTAGTTGGTTAGGGGGAGCTAATTGGCAATGAACATAGGAATGGCAACCGGGGAAGAACCCCACATTACTACCTATCACATGCACGAACGGGGGCAGGGCAGCAGAGCAACACTTCACGCACTATCATGACAACTTGTTCCCAGCGAACGAAGAGAAGGAATACTTATTAGTAATGACAGGAATGACAGCAAGCAAACAGCCGGGAACTTGAAGAACGCCTCCGCTGCTAACGCCCTTACCTCATCATGTCACTTATTCCCGTCGAACAAACAGGGATATTCTTTAGAGAATGCGCAGAGCAGCTTAGCCGCTCCATCGTGCCTTACCTCAGTACACGCTACGCGCAACGCGGCAGGCACTAACTCACTACAATACATGCAGGAACAGCAATCACAACTAAAAGCAACTAGCTGGCTTGCTCATGCAACCTATTATTCCCATCCGAACAGAGAGAGGAAGAGTGACTCTTATAATGACAGGAATGAAAGGAGTGACTTAGCTGCACTTGCAAATACAGCAGTACTCCGCAGGCAAACAGCTGACCTATCATGCCTATTACGAACAAGGAGAGGTACTTTAGTAACTCGATTGAAAGGAGAGGTACGGATCGAGCGTAGCTTTTGAAAGCAAAAAGGATTCAATCCGCCTTGCAGCAAATACACAGATGTTCTTCCCTCTCCGTCGAGTACCTTCCGTCCTTCATCAAATACTTAATGAGTTCGCCCAGAAAGAGGGGGAAGCTTGTTCTGTCGACCTTTGCATCAATTCCCTCTTTTAGTGTTAGCAAGTCTTCTCCCATGCTGTTCATTGACTCCCTTTGTCGTGTTCCTCTTGCTATCGCAGGCTGAGGTGGACTGGAGTTTTTTCGTGAACCAACGAGTTCAGTCGTGTTGCGTAACGAGTCTAAGGGAGTGGAGTATACTTTTTCTATGATATAAAACAAATAATATACTAGGTGAATCAGCAGTTCTCCTCGCCTACTCTATAGTTCTAGTCCGCCTACCGAACGGAGGAAACAGAGATTCTACCACATTGCTTCGAGACTGGAAGGAATGTCAGTCATAAATAATGAAATCCATATAACTCTACTCCGGGTTGTGAGAACATCGTGCCTGTTGCGATTGTGCGGCTTTCCGCATGGCTGGAGACCCCCTATGAACAAACAGTAGGGTGGTTGTAGGAGAACCCCGACTCCCTAATGCAAGATAGAGCTCTTAGGGTGCGTTTCGTTCCAGCAACAGTAGTATACGGTTCAAAACGCCTTGTTCCATCCGGCGCGAATCCCCTCCATCACTAGTAAAGTGGAGGTGTTATTTGTATTGCAATAATGAATAAATGTACGAACACAAATAATGAGCAGACCTGCCCACGTTTATATGTGGATTCATTTAATAATATATTATTTTTTTAATAAAGAAGCGCTTTGAGGCAGGTTGTGTTTCTCGGTTGATGGATGGAGTTCCATTGTCCCATGAGAACTCTTAGCTACTGAGATACGATAAGAGAAAATGCACTTTTCCATCTATATAAAGAGAAAGTTTTGGATTCCTACTGTCCTCTTACGAAGGAAAATTGGATTTATTACTTTTCCATCTATATAAAATGAAAAGAAAGATTTGTGTTTAGCTCGCCTACACAAGCCAGGGATTCTACTTGACTTTTCAGATGTTGCTTATCCAGGCTTGGTGGTAAGGGGATGGGTTAAGCCAGCTCGTGACAAAAGTACCTCTCGGACGAGTTTTCTCGATTACAAGATCGAGTTCTCAAAAATGAATAAATGCGTACAGATATAGATAAAGTGTGCAGTGAGGGATCTTTATAGGTAACCAGTCTTTACTTAACTCGACCCAAGTAATGCCCAAAGACTCCCATGCTTTTCTTGGTTGGACCAACCGGCACCGGCAATTTCCGACGAGTCTTTCAGAATTTGAAGAGCAAGAAGCGGAACTACAAGAAAGCTTTCTTTATCTTTATGGATAACCAATCCATTTTCAAATATAGTTGGGAGACTTTACCCAAGAAATGGGTCAAAAAAATGGAAAGATCGGAACATGGGAATAGATCTGATACCAATACGGACTACCTATTTCAATTGTTGTGCTTTCTCAAATTGCATACCTATACAAGGGTTCAAGTTTCGATCGATATTTGCGGAGTTGATCATCCCTCCCGAAAACGAAGATTTGAAGTGGTCTATAATTTACTGAGTACTCGGTATAACTCACGCATTCGTGTACAAACCAGTGCAGACGAAGTAACACGAATATCTCCGGTAGTAAGTCTATTTCCATCAGCCGGCCGGTGGGAGCGAGAAGTTTGGGATATGTTTGGTGTTTCTTTCATCAATCATCCGGATCTACGCCGTATATCAACAGATTATGGTTTCGAGGGTCATCCATTACGAAAAGACCTTCCTCTGAGTGGATATGTGGAAGTACGCTATGATGATCCAGAGAAACGTGTGGTTTCTGAACCCATTGAGATGACCCAAGAATTTCGCTATTTCGATTTTGCTAGTCCTTGGGAACAGCGTAGCGACGGATAATTCAGAATCAGAATAGGTCCACCAGTCCAGGGGACAAATCAATAGGAAATGCTATTTGCTTTGTAAGAAGACTTCTATGAAAGTCTTTCAAAAGAGAATTGCTTCTATCAAGATAGCCGCCTTGTCAGAAGTCCTTCTCCAAGCAGTTTATGCGCTTAAACCCTCGCCGTAGAATCCTTTAACCGAGATGAATTGCTAAGCTTCTCATATTCGTTCGATAAAGTGAATCCACAGAATCTATCCTCACCTATCCTTATGCTTTTCTCACAGATTCAACAACAAAATGTTTCTCTTCGGGGATCGATGAAAGGCTAATCCGATTTCTCTTTCCCGGCCGCCCTTGCTCTTTCTTTCTTGAAAGGTGTCTGGTGCTTGTTCTAGCAGCTGACAATCAAGGACTGAGCATGGTTTAGGACCAAGGGGGAAATTTAGAGACTGAGAGCAAGGATTCTTTTTTAGTAGGAACAAGAAGAACATCACTAACTGGTATTCTACCTTCTGTGGTAGCAAGAGTTGTTGAACCGACATGAGAAATAGGAAGATAAGCACCATCTCCAACCATACTTCTGAGCCATTGTAAACTAGAGACTGTTGTCATTTTTGAACTGAATCCGTGACATGAGTGGAGGAGAAAATGCTCTACTGAGCTAACGGTGGAGGAAGGGCAGCTAGAAGCCCGGTAGCAAAGCAAGGCAAAGCAGATCCAAAAGAAGAAGGAAGTCGAACTTTCGTTCGAACTACAGTATTTATTATTATATTTAGGTAATTAGAAATATCGTAAGATAAGAAAGAATTGACAAGCGGATAAGTTTTCTAGTTGGCGCAGTTGGAAGTTGCTTGGGAGGATCTGTGCTGGTTGGTAGACTTGCTAGAGTGGTTGCTTTTGGTTGACATAGATCCATAGGTATGTGCTCGCAGTCGTCTCATAGAGCACTGCGGAAATCGAAGTCAAGGGGTAAGGTGTAGTCAGCTTCCCCGCTCACGTTTCGGGTTGCTAAGGTCAGATTCTTGAAACAACAGTCCTAGGAGGGCCGATTGATTGACCGAGATGGGAATCAATGCCTAGTCAAATCAAAGTCAAGTAGGGGCGAAGCCGTTGCTTGTTTGCTCCTTCGTATAGGTTCCGTGTAGGCGTCTTTCCATACGATCAATTGAATATTGGAAAAATCAAATCCGGTGGGTTTTTATCGTGAGCTCCTTTCGCGAGTCGAGGAACGCCAGTTAGTCGACTAAAAGGTCTGCAAGCCTTGTCTCGCCATTATTATAATGGCGGGAACCATATTCCTTTTCCCTTTCAAACTCAAAAGGAAAAAGATGCTATCTATTCCATTATGTGGCAATACCGCCGCGAGGAGTAGAGTGAGTCCATGCTTCCTAATTTTCTGACTGAAAAGTCTTTTGGCTCGATGCGCTTAACGAAAGGGAACTCTCTAGATTAAGAATGAATTTCCTTCTCCATTAAGCCGGACCGGCGACGAATAATAAATAGGTACGTACCCCCACCCCAGGCAGAGTTAGTGAGCCGTGTAATAGGCGACCATTTCGCGCGGTTCGGGGGGCACTTGAGTAAGCCGCCGGCGCCCGACTGCGTCTTGACCCCTATCCAATTTTTGGGCCAATTCCCCCCTCGTACTACCAAAAAATGAGATTCTTGCCGAATCCGAGTTTGCTGCTCCAACCATTACCAAACTAATACCTATTCTGTTTAGTACTTCAGGTGCTTTTGTTGCGTATAATGTAAATCCCGTAGCGGATCAATTCCAACGAGCCTTTCAAACTAGTACTTTTTGTAATCGACTCTATAGCTTCTTCAATAAACGCTGGTTCTTCGATCAAGTTTTGAATGACTTTCTAGTCAGATCGTTCCTGCGTTTCGGATATGAAGTCTCATTCGAAGCTTTAGACAAAGGTGCTATTGAGATATTGGGCCCTTATGGTATCTCGTACACATTCCGACGATTGGCCGAGCGAATAAGTCAACTTCAAAGTGGATTTGTTGTGCGAAGAGTGCGTTATGACCCGTGGCCGCCTGCCTGGTGGGGGGCGGCTCCTCCGTTGTGGGTAAACGGGAAACCCGACTCTACGAACCCGAGGAAAGGCTGCACAGCAGTAGTAAGGGCGTTAAGACCGGAGCTTTTTGTAGTGCTAGCAGGAATGCAAGTGAATGAATCCCATCCCCTAGCGAGTGAAGTGCTTACGCTGCCTTAGAGATAGGGGCGAGCAAAAGAGTCCGTTCTTCAATTTTGAGATCTTTAGATTCGTAAGATCCTGATAGAGTCCCCTTCCCTTTACTAATATAATAGAGAGGGGGCTGAAAAGCAGTTGCTTGCTGTCTACTTCGCGAGCCTTCACTGCACCGTTCTGTAACTTGCCTTCTTTCGTCCGTCCACGAGGCTGTAAAAAGAGAGATAGGGGCGGCTATCTAGCGGGAGTCGTTTCGGTTGTATGCCACGAGGTCCCTATGGACAAGGGGACAAGTGAATCATCGCTTTTGGGCGCAGGCATCCCTCTACCATCCATCCCATAGCATTCCATCGTCTCGTATTGTACTGTACCGTATCAGACCAGATAGATCTATTGAGTGAGAGAAAGGTAGTGTAACTAATTAGATATTCTTCATATTCTTATTGATAGGGATCCCCATTCATTCCTAGATTCCCGTCATTACGGATGCCCCTACCTAACTACATGGTAGTGGTCTAGGGAGCGCAATTGCTAGAGCACGGGAGAATGAAGAGTAATGATTTCAGCAAGAGCAGCCGGACGGACTATTCTAGTGAGTCTAGTGACTACTAGAGTAGAGTTGAGTCAAAAGGTATGGTATAGCAGCCTTTCCGAGCGAGCCTCTGGGATCTCCTGTAAACCCCCATGATGTGGTAAAGGGAGGATATTAGGGGAAGCAGTGAGTGGAGATTCCCCTGCGGAGAGCCGGATGAGGGGAGACCTTCACGTCCGGTTCGGAGGGCGGGGATATCCCGACCCTACTATCATTATGCCTTTGCAATGTTACTTGGTTCAACTCTATTTGTGACCTTTTCTCGTATGTGGGACTCTCTATCTTCTTGGGTAGATAATCGATCGTCTTTCATTTTGATAGTGAGTAGTTTTTATACAAAGTCAAGTCAAGAATAATAATCGAACTGGAGGAGCTTGCCAGGATGGCTTGGTAAGCAAGCAACCAGGCGCCCATTCCCAGTTCTTTCTCTTCTCTCTTTTTTTAGTTTAGTGACAGCTCATCAAAACAATTGTCCAAGATTGCTAGATCGAAGACGCGACGAAAAGTAAGAAATTCCGAGTATTTTGACCAATTCAAGGCGCGTAGCGGGGTTGCTCCTTTAGCAAAACTTGGATAAAGTTGAGCCAAAAGATCCCGATTCAAGAGAAATTCATGAGGAAGTAGTATTTCTTTAGGATCTACTCCAGCATTTAGAAATTGGTTAATCGGTAAAGATACATGTACTGGATTGTCCTGCCCAAGAAAGGGACCCAAGTCCTAGTAGCCCTGCTAAATGGTGATTCAACATAGATTCTACATCTTGGAACCAAGCCAATTTTGGAGCTGCTTTGTGATAATGGAACCAACCAGCAAAAAGCATTAAGGCTGCGAAGACCAATGCGCCAATTGCGGTACAATAAAGTTGTAATTCACTAGTTATTCCAGATGCTCGCCAAAGCTGAAAAAAGCCAGAGGTTATTTGTATTCCTCGGAAGCCTCCGCCCACATCTCCATTCAGGATTTCTTGGCCCACTATTGGCCAAACCACCTGAGCACTAGGTCCAATGTGAGTAGGATCGCTCAGACATGCTTCATAATTGGAAAAACGAGCACCATGGAAATACATGCCACTCAGCCAAAGAAAGATGATAGAGAGTTGGCCAAAATGGGCGCTAAATACTTTTTCGAGAGATTTCCTCCAAATCACTGGTATGACTCTGTGCATCGTTCATACTTCCGATGTGTGGAGGGAACCATTGCAGCTATCCCTTGAAAGTGCAGCTAAAGGAGGACCTCCAGGTCCGTAGAAGAACTACTTGCTTGTTTGTCCTGGTATTGGAAGAGGTATCTAGAGGCGTTCTTCGGGGTCTTTTCTAGTCTTGACTTCCTTCTCTGACCCGTTGTGGGTGCCCGGTCTCCCTTCCTATTCTATCTTTGTTGTTTGTGAATGAGTACCTATCCCTTATGGGCGAACGACGGGAATTGAACCCGCGCATGGTGGATTCACAATCCACTGCCTTGATCCACTTGGCTACATCCGCCCGCTACGCGCAACGGGCTCCCCGCTCCTAGTCACTAAGTAGTGCTCTTCTCATTTCGCCCGCCTGTTTCACTCCCGCGCCGGAGGGAATGGGATTCGAACCCATGATACAATCTTCTTGTATGTCGATTTAGCAAACCAATGCCTTAAGCCACTCAGCCATCCCTCCAAGTTGTTGATCGGAATTTTTTAAAGAGTCAAGTCTTACTTATTTAAGCTGGAATGAAAATCTTCTTCTCTTATGATATTTTATAGAGTTTCGTTTCTTTTATCTATCTCCCCATTCGAACGAGAGAATTGAGTTGATAGCTTCAGTAGTGGTTGCTTGATGGTGTGTAGTGGGATGACCTGGTAGCGATCATATTAGTATGTATATGAGCAGGACTTTCAAGATCTGATATCTTTCAAGAAAGTTGGACCATTTATCACATCAAGGTAACTACGATAGGCAAAAGAAGACTCTTAGGTCGTTGATTCAAATTCTACCTATATTGCATTCACTTAATGGTCAAACCTACAATGGAATTGACTCTAACATCCGATCCGGCCGGAGGAAAGACTTACTACTACAAGGGCTTGTGCCAACCAACAAGAAGGGGGACGGAGCAAAGACATCTCTTGGCCAGAACCGGACATTGCCCTTTACCATCTCACCACTAGGAGACTGGGACTCGAGGCGTGGTTAAGTATTCCCTTCCGCTCTGGAAGTCAATTTATTTCCTATCTTTCGGTTCCAAATCTTTTTTTTCTTATAAAGAGTGTATTGTATGGTCCAGTTGGTGTGGAGAGATGGGTCACTCTTGCGCATCTCGCGCTGTGGTGATTGGTAGAGGAGCCGAGCGTACGCAAGAATCAAGTGCCTCAGGGCTTCCACTAGGCAATCGGACCACTACATCGAATTAAATAAACTGGTTTCACGCTGCCTGAGGTCAAGTCTTCCCCGCTATTCTAACTCTAAATCCCGGTCGCACTCTTCGCACAACAAATCCACTTTGAGGCGTCGGCTGAACCTTCGATCCTTTAGGAAATGACCAGCAACAGGAGGATGAGATGCCGAGGAATTTTGGTGCTAATCCCAGGACCAATCCCGTTGTTTGGGCCAGCCGAGAAGACAGACAGCGCACTCAGGACTAGGCCCGTATCCAGAACGAATAGAAGGAGGATAATTGCCATTTTTATGCTAACAGGCTGCTCAACTTTTGGAATGAGAACCAAAAGAGACCGATTAATCTCCGCGTACTGTCTCGGATCACTCATGGGAACGGGAGTGGGTGGTCCCTAGGAACGGCGGAAAGGAAGTACTGGTTCAACCAGATACGAGTGACGGAACCGGGGTTGATCAAAGACATGCGCCGAGTGCCAGGAATGACTATAAGCCGATTCGTTGTGGAACTAATGCTTGCATCGGTTTCTTTATCTTGTGGTCGACATTGAAAGATTCCTGTTCTAGGATTGGGCGGTGTCCGTCCACTAATGTAAAGATTGGGCGTGGGAGAGGTTTGACGAAAGAAACTTTCCGGGCGATCATCTTCTGAGCTCTGATCGTAGACCAACCTCATCCGGCGGACAGATTCCTGTTCTGCTGATCCTTCTTCTTCCGGACCTGAATAAGACAGAATGAATCTTTATCTGGGAAGGTGAGAGCTATCTCGAAATCTAATAAATTACCTCCTGGCCCCTGTTAGAAGGTTATCCGCCTTGCTCCAATGCATGTCGAAGGTTTAGCTGGGGAGCACTACGGTATGCTGCATCTATCTCTCTTCGTGGGACTAAGTAGAAGGTTTCGGGTAGAAGGCTTTCCCGTTTATTCTAGGTGGTCCATCCTAACATTTAACCAACGAGGAGAACACTCCACGAATGAAGAGGATAATCTCCTATCCTTTTTTTCGAGTAGTTTTGGCTATCGATCTAGTAATATAGTCAAAGTAATCAAGGTCCGAAACTCAAATAGCAAATCAAATCTTTCGATATAAGGGTCTTAGAGCCTCCCCTATAGATAATCCAATCTAACCCCTTCTGGAAAGCCAGACCATAATATCGAGCTAGCAGCTTAAGAAGCAAAGGGACAGTGGGGTGGTGAAAGTGACAACAGATGCTGCAGCCGCTTCAGAACTGATGCTAAATGACGGACAAAGCTACTCCGCTACGGCATAGGAGAAGAGGCCTACGCACGTTGAAGTAGGTGTCTAGGTGGAAAAGCAGAATCAAAAATAGCCAGTTGTTAGAGTAAGATGAATTCATTACCTTATGGTGCTTACAGAGCATCAAGAAGAAAGTAGCTTAAGACTATTCCCCTTATAGGTTATAGGCACGCTTACTGCTATGATATTTTAGGGCTTCGGTACGGTAGAGTCTTCCCTATAGCTTTCCTTAGATGCGGGCTGGAAGGTCAGCTAGTTAATCAATTGTGCCAATCCTTCTTCTCTCTTTGACGACCGATAAAGAAGAAAAGCAAATACCTATTGTGGGATGTAGTACTCAATCTGATATCAGTACTTTCCGGGGGTGCAAAAGCAGATTCTCAAAATGCCATTCCCCTACCTCTTTTAACTGCTGCTGCTATCAGAGGTTTCTGGTACGGAAGCGGATTAGCTGATATGTCTTGTTCCAGTGCAAGCGCTTGTGAGTTCCCTAGGTTACCAATGGGTGAGTGGAGGAACCCCTGGGAAAGCATCTTGATCAGATCTAGACGAATTAGCCAGATAGGCAGACACCCAATGGAAAGATGAGTGCTGTTCAATTATTTGAGTTCAGTCTAATTTTCGACCTAACAAGAGCAACGGAATCACGCTCTGTAGGATTTGAACCTACGACATTGGGTTTTGGAGACCCACGTTCTACCGAACTGAACTAAGAGCGCTTTCTTATCACAATTAATAAGACTGTAAAGACGAGGATTCTTTTTTTTTTTTTTATAACCCCAATAAATTTTCCACGCCTATACTATTATATATAATATGAGAAATTGAAAGATTATCTATGTCCAATTTGAATCGATCTCAAATTGATCCCTCCTTACTGCTCAGAGGAGAAAGAATAGGTAGGGATGACAGGAGAAGTCTCAGATGATCCTATCATTTGAGGATTCGAACCTGTGACATTTTTGACTCAAAACAAAGGCGCTACCAAGCTGCGCTACATCCCTTTCAATTGGTCTACGGTCTCATTGTAGAGAATCCCTGCCCTCTTTTCCACATCCGAGGAACGCCTCCATCGATATCAAATTTCTCTTGCCATTTTTTCTTTTTGCTTTTGATTTATATACTCATCATAATCCCGCCCCTCCTACAAACGCTAACTTACTTATGAGAAAAAAGGCTGATAAATAATCAGCCCTTTGAATTGAATAAGCGAGGCTTTCCCGATAGAAATGCTTGCATGCGAGAGAGATCCCAATTCCGTCTATCCGAAAAAAAAAAAGCAAGCCCTGCCGCCAGCTTCCACCCCGACAAAAAAAAACATGAGCGCCTAGCGCGAAAGGTTGCTTTACTAAATAATATAAGGCGCGTTAGCGCTTTAGTTTTCAATAAGGTATTTAGTCACTCAGCCTTTAGGCACTTTAGTGACTCGAGAACTTGTTTCGAGGCACCCTAGTGACTCGACTGAAAAGGAGAGGTTGTGAAACAAACTCGACTAAAAGGAGAGGTATTGATTACTCGAAGCGTAGCGAGAGGTTTCAATAACTCGAGTGAAACGAGAGGAGAGGGGGGAGAAAGTCCAAGATTGCAGTAAGTTTGATAATGATCGAGTGACATTGCTTTTTCGGTCCGAACCAAGGAATCCCTTATATATGATGAAAAATGGATCTTGTTCTATCGTTGATCAGAGATTTCTCTATGAAACGAATCGGAGTTTGAAGAAGGGGAAGGAGAAGGAGTCCTCGACCCGGAACAGATAGAGGAGGATTTATTCAATCACATAGTTTGGGCTCCTAGAATATGGCGCCCTCGGGGCTTTCTATTTGATTGTATCGAAAGGCCTAATGAATTGGGATTTCCCTATTTGGCCGGGTCATTTCGGGGCAAGCGGATCATTTATGATGAAAAGTATGAGCTTCAAGAGAATGATTCGGAGTTCTTGCAGAGCGGAACCATGCAGTACCAGAGACGAGATAGGTCTTCCAAAGAACAAGGCTTTTTTAGAATAAGCCAATTCATTTGGGACCCCGCAGATCCACTCTTTTTCCTATTCAAAGATCAGCCCTTTGTCTCTGTGTTTTCACATCGAGAATTCTTTGCAGATGAAGAGATGTCAAAGGGGCTTCTTACTTCCCAAACAGATCCTCCTACATCTATATATAAACGCTGGTTTATCAAGAATACGCAAGAAAAGCACTTCGAATTGTTGATTCAGCGCCAGAGATGGCTTAGAACCAATAGTTCATTATCTAATGGATTTTTCCGTTCTAATACTCTATCCGAGAGTTATCAGTATTTATCAAATCTGTTCCTATCTAACGGAACGCTAGTGGATCGAATGACAAAGACATTGTTGAAAAAAAGATGGCTTTTTCCGGATGAAATGAAAATAGGATTCATGTAATGTAACAGGAGAAAGGTTTCCCATTACTTAGCCGGAAAGATATGTGTCCATGAAATAGGGATTAAGTGGAACGGAATTGACTGGGTGGTAGAGTTGTAGAAACACCTGTTTCTTCCACTTAGCTCCATGGAACAATATGCTACGACGGAAACATGGAAGAATTGAAATCTTAGATCAAAACACTATGTATGGATGGTACGAACTGCCTAAACAAGAATTCTTGAACAGCGAACAACCAGAGCTATTACTCACTACATCAAAAAAATTTCCATTAATGAAGGATGGAAATCCATTGGAAAATCAAAAATACGCATGTCGGATGAAATTGTTGTTGCTATCTGTTCCAATAACGAATCAACTGAATAACTAAATAAAATAGATAGACCTTTCTCTTCGTCTCAGGTCGATAGATCTTCTCAATTGGAAGATCCCCTATATGGATAATACACATTCCAGTTGACCGAGCCTAATTCTAATTGTTTTGTTCCGAAGTAAAGATATCCACGGAGTGGTTCGCCCTATTCAGATATTCACGACCAAGAAGTACTGGATTCTGTTTAGGATAGGTCCTGAAAGGAGAAGGAAGGCTGGAATGCCGCCAGGCGTCTATTATTGAATTCACCCGACCCGATAGTACCAATTTTGGTAACGTCCATCCAGTGCCAAAGTCACTGAATGGGTAAGTCACCAATCCCTAAAACGGACTATGTACTTTATCTGCTGGGTTACGGGGGCATTTTACCAGAGGTTTAGATTGTATCAATCTACCCTTGTGTGATTCCTGTTGAATCATATACTGCGGGGCGCAGGGCGGACGATTTCAAAGCGGACTCCCCCTCCCCATTCATTAGATAGAGAAGATCGCCAAGATTTCGCGATCCGCTGCCGAACTTATTCCATTTCAATATTATGCCTTGAAGAGGCGTTCCTCGAACCTCCACGCTCTTTAGCACGAGATTTTGAGTCTCGCGTGTCTACCATTTCACCACCAAGGCGTTCATCGGAGTGAATCGTATTCCATAAATATGATATCTATCTAGTATGGCCAATTGGACTGAATGTGGTATGGCAGCAGTAGCAAAGGGGCACATTAATTAGTAAGGCAAGCTGTAAGAATAGCACTCGGAAATCTCTCTAAAACCAAAGCCCGGGAGACAACTGAGTGACTGTGGTCAAGTAGCTGTTAGTAAGAAGCTCCGGTTCACAAAAGGAAAGAGTCACTGACCGAGTGCAGTGGTAATACCGACAGAGAGAGCTCCCAACGGAAAGAGGTCCCTAATAGGAGAGAAAAAAATTCCAAAGGTATGAAATGACTCGATAAGAGGTCCTTTGGAAGAATGCGGAAGCACCACCAATGGCCAGGCACAAGGCTACGTCTGGCACTCTTGCAAGTAGGGAGTCCTATGCCTTTTTATATAAACAGATAAAAGCTCGAGAAGATCTTTCCTCATGTGGATTCAAAAAGCAAGGATCTGTCCAATGGATTTGCCTTGAAAACAAAGTCGTATTTGAGTTGGGAAGTTCTAGGAAAAAGGTTTGCCTACGGAGAAGACTTAATCAATTCACTTCGTGGATAAACTTCATGGTGTTATTCTTTTGGAATTTCCGGTGGGAAAGACCAGGGGAAGCATTGACACTATGGAAAGAATCTATCTCTGGCAAGGGCAAAAGCGATAGGCCAGATTAACGAGGAAAGGGGGACTGGTTCAACCTCAGGAATGAGAGCAAGCAAGTCCAGATTTGTTGTGCAAGTCACTGTCTTTATCCGATCCGATAAGATAGATCTGATGCATATATAGTAAAGAAAAGCTCTTCCGCAAGCCATTTGGGGAAATTCCCTAACTTTGAACGAAAGAATAGTAAGCTTCTTTCCTTGCTACTTGAAAGAATGCCTTGGCACACTCATACTAGAAGTCAAAGAAAGAACTCGGGTCTAAGCTCTCAACTTCAAGGAAGGCGTTCCTCGAGAGCAGAAATGTCATCTACCTTTCCAACGGAATCTTTGACCGACTAACGTGAGTTCTAGGAAAAGAGAGAGACTTTAAGCTAAAGAAACCACTTTTTTTTACCTAGACATTCTTCTAAGAGTTCAGGGCTAATAGGCCCAGTCTGATAGTTATTGGCTGAGAACATAGAGCGGGGAAAGTTCTGACTTTGTGGTTCCCTAAGAGCAGTTACCTCCTGTCCCGGAAAAGCCTAACCTGTCTAGCCGGAAAATGCCTCCTTTTTATTACCTCCAATAGATAAGTTCTAACACTGCAAGGTGTTATTCTGGGAGTGTTGCATGCCCAATAGAAATCTTTCTTAGACTTTACCCCTTACAGAGAGAATAATACAAACTTGAATACCCGAAACTCCAACGGAAGAACTCCGGCAAAGACTTCCACTATGAGCTCGATTGTTCCGCTTAGGGCCCGTAGGAATCTGAAGCCTTAGACTTCTTACACCGGGACAAATGAAACTACTATATCCGGAAAACTTCTACTCTGTCACTGTTTCCCGCGGCTTTTTTTCAATGGAATTACAGCGAAGTCGAGTATGGACAGAGAGGGTAGCTCCTTTTCGTCGAAAGCTTTGAAGCAGTAGCCACTCTCTGCGCATGTTCAATAAGATAGCAGCTCGATCGAAAGATATATCTCCGATCACTTTTTTTAAGCGAGCACCCTGCACACTGGATCGTAGGCAGCTGTGGCCGACCTGTAGGTCTTTTGTTCGGATTCTGATGTTAGAGACTCAAGGGGTTCCTCCAGTTTCGCTGAAGAAGCTAGGTTCCTCCTGTGACAGGCTTATTTCTCTTATGGTAGGGCAGTAGCAAGAAGCCTCCATAGGGGGTGCGACTCCTATGACTACTCAATCGATTAGCATAAACTAAAGGAAGAGGTGGGATTTCAAGGGTTAGCCGAGGAATTCAGTGAAGTTTCCCAATCTACAATCCTAGCCTCCCTAGCAGGTAACCATTCTCTACTCACTTCTTGGGGCACTTGGTATAAAAACCCCTCTCCTTTCAGTCGAGTGACTTCGCCCCACTCGGGGAAGGAATCGAAAGATAGTGAATGACAAAACAAGTGAGGGTAGTGAGACTTCAAATCTTCGAGTAGAATCGGTTTGTTTGCAAATCCCCAAGCTGTGGCACTGATGACTTTGTTACCTTTTCCCGATTCTTTGAATATTGATTATGCCTTTTTCCTTTCCATCTCCCTTTCCTTTCCAAAAATGATTGATTTGCAATTGGGTAGTAAAAGTGACTTGTTTCCAAAGATAGGGAGGGATAGGGGTCGCAAGGAATAAGTCGTTTTCTCAGGATCAGGTCCCTTCCCCCTTTATTTTCAGAAGTGTAGTACCTGTCTAATGCGGTAACTGATCTCATATCTCACATCTCGATCTCTCTCAATGGAAGCTATCTTGCTCAGTTTAGGGCGTAGGTAGGGGGATGCACTCATTGCTGCTCTCCGACCTTAACTTAAAAGAGAAGGTAGCTCAAGCAGAATCCGAATCGGCTAGCTCTCATTTATTATATGGTGGAAAACAGCCTGTAAGGAGGAACGGAGTTGGCTTCTCCCGTTGGTCAAGCAGGCTTCGCTCCTTGTGATTCATCTCGCGTCCTATGTACACACGTACTTGTCTCGTTTCGAGTGCTAGGACCAAAGAAATCCGAAGAATTCTACCAATAAGATCTTAACCAGCCATTGAGTAGTAGCTTTTGTTTATTCAACGGATCGACCAGAGAGGCCATTCTGCAACCTGAAAAGCCTTATTTGATTCTTTCTACCTATTCTATGCGTGCGTCTCGATAATCTTTTAGAAGAGCTTCCATGCCCCATCTTAGGTCCCAATTCAACTGCACCCGCTATAGCATTTCAAATAAGTGGTTATTGAGTTCACGGAACACAAACAGAATCTTGAATTTCGTATAGAAAGAAAAGATTCACTTCTATTCTCGGAGCTGAGGTATATGAAGAATGGCTTTTTTTTTTTTTTTTGTCCCTTTCGTCTAGTGGTTCGGACATCGTCTTTTCATGTCGAAGACACGGGTTCGATTCCCGTAGGGGATATCTACTCATTCTCGGCCGCTTTCAGTTAGTGTTCATTGATCGGGTGGATCTATATGCTTCGGGGAGTGAGACGAGGTGTAGCGCAGTCTGGTCAGCGCATCTGTTTTGGGTACAGAGGGCCATAGGTTCGAATCCTGTCACCTTGATGTCTTTTTTTTCGCTATGCCGCTCCGCCCGCCACAGCATATATATAAAAAAGAGGGAAGAAAGAACAACCGTTTGACTTTGGCACATGAGGTGGCGGGTTTGGCTAGGTAACATAATGGCAATGTATCGGACTGCAAATCCTGGAATGACGGTTCGACCCCGTCCTTGGCCTCTCCTTTCAGTCGAGTTGCTAAAGCACCTCTCTAGACAAGTGCTCGAGTCACTCCGAGGAACGCCTTTTCAGTCGAGTTGCTAAAGCACCTCTCCTTTCAGTCGAGTTGCTAAAGCACCTCTCCTTTCAGTCGAGTTGCTAAAGCACCTCTCCTTTGCTGTTCGAGTAAACAAGAAATGCTCGAGTTACTAAATACCCCTAACGGGGCCCCTCTCTGATAAAGAAAAAAACGAAAAAATCTCAAATTTATGAAAAATTATGTTATTAACAATCTAGTTCGACTATTGTTACTCCTGCTCGGTAGTTCCGTAGCATTCAAACGTTTTCTAGGATCAGAAGGAAGCGCTATTCTGACCACTACGTGCGTTTCATTCTTCGCACTGGTGGGCTTCCTATTTTTATTTCGAATTTATTACTTTCGTTTGAAAGGACCACTGAGGGAGATTCTCAATCTCTTCTTGGTCTTTTCCGCTGGGTTCGTGGGATCTTTGATACGGATCGAAGTCATCCACCTAGTGAGTCCGGCTTTGCCCCTGTTGGGGCCCTTTCTATGGTATGCTGTGACGGGTTCGTCTGGGGAGGTAGTGAATCACCAAACCGAGGCCTCTTCTCAGTGGTTTACGTATACTTCCGACATGCTGGAAGATTCGGCCAGTTCCGGGCGTAGCTCGTCGGTCAATCAACCGATTCAGAGGGAACAGGCTGGGCCATCCAATGCCTTTCCCGCCCCCCAACCCACCGCTGCCCCAGCAGCTCAACAGCAGAATCACCTAGATCAACCAGCTGGGGAAAGGGAGGCTAGGGCGCAAGAGCACGCCCGCATCTCTGCGGAGATCGAAAGGATTATGGCGGCCTGCGAGAATGAGGAGGCCGCCATGATACGCAAAGCCCACAACCTCTTGCATCAACTTGGCATAACTCTCGAGGATGCAGAGGATGTCAAGCGTGCTCTCCAGTTGGCTCTCCATGACGACTGGGAGCACGATATCGATGACCGTAAGAGGCATTTCACGGTGCTCAGACGCAACTTCGGAACAGCTCGCTGTGAAAGATGGAACTCTTTCATTGAAGAGCTCCGGGGTTTGGGTAACCATCAGGTAAATGCCCGGCATTATCTAGACTGACATATCACGGTGGGATCTTCGACTGGGGTGAAGTCGTAACAAGGTAGCCGTAGGGGAACCTGCGGCTGGATTGAATCCTTCTATAGATAGAAAAGTTCTTAAGCAAAGACTGGAGAGGCCCCCGGTATGTAGTAAGTAGGTCTCCAATACTGGGAGACTGAGGAGAATGGGAGTTTGTGGGTTTAGGGTGGCATGCTCCCCAGGGCCTTTTTTTGGTAGGTAGGGTTCTTCAATATGTGATCTAGCAGTGTAAACCTGAAATTTGTCAGAGTTGGCTTCGATTGAGATTAGAAAGCGTAACGAGACTAAAAGAGTAACCTCGTCAATGCAGCGCACTGCGTTTCGAAAGATCCGAGGGCCCATAAGCCGGGTGTCAGGTACAATTTTCAATCTAACGTACGACTCCGCTTGCCCTCTCCATTCCTTCGTTTTAGAGCGCAACTCTAGTTTCAGCTGATTGCAGAACCATAAACCTCGCTCGCTCTATCGAGACCAAGCCCAGCCTGTCTTTTTTTTCAATACTGTTTTCTTGCATAACATAGGGGTTCCCGTAACGCTTAAGATAGGAATTGGGCCTCGACAGAAAGATCCCATCCGCCTTTGGGTGTGACTTCTTGTAGTCTGTCCATTATTTCGAGGATTCTTCTTCTACCTTAAGGAGCGAAAGGGCACTCTCCTCCTTTCGTGTTCCTTGTTCTTGTCAAAGCTTTCTGTGGTGATTAGGCGAAGGAACTAAGTAGCCGATAGAGGAGAAGGATCTTCGAGATCTGCCCAAAACTTCATTGCTGACTCAGCACACTCCTTTCCGTCGTTCTCTTTCTCACTCCTTTTTAGCAAAGCATCAATTAGCAAAGCAAAGAAAGCTGACGGTCCCTCGGCTACCCGTTACAGGTTCTGAACCCTTCTCTCCCTTGCTTTTGGGCGATGATTCGATTCTTCTGGGCTTGCTTGTGGCCACTTAAGAAAGAGTTCTGCCTTCTAGCCAAGCCTTTGAGTTTGAGACCAAGTAAAGCTTCCCGGCAGTCAACCAAGAAAGACTCTTTCTCCCCTACTGAAGAAATGGAAGTCAGATCTTTTTCCATACCATAACGTATATAGAATCGATTTTCTTTTCTGATCGCTAGCCTGCCGGGCCGCCCCCGCGATCAAACTATCAATCTCATAAGAGAAGAAATCTCTATGCCCCCTGTTCTTGGTTTTCTCCCATGCTTTTGTTGGTCAACAACCAACCACAACTTTCTATAGTTCTTCACTACTCCTAGAGGCTTGACGGAGTGAAGCTGTCTGGAGGGAATCATTTTGTTGAAATCAATTAATCTAATCATGCCTCAACTGGATAAATTCACTTATTTTTCACAATTCTTCTGGTTATGCCTTTTCTTCTTTACTTTCTATATTTTCATATGCAATGATGGAGATGGAGTACTTGGGATCAGCAGAATTCTAAAACTACGGAACCAACTGCTTTCACACCGGGGGAAGACCATCCAGAGCAAGGACCCCAACAGTTTGGAAGATCTCTTGAGAAAAGGTTTTAGCACTGGTGTATCCTATATGTATGCTAGTTTATTCGAAGTATCCCAATGGTGTAAGGCCGTCGACTTATTGGGAAAAAGGAGGAAAATCACTTTGATCTCTTGTTTCGGAGAAATAAGTGGCTCACGAGGAATGGAAAGAAACATATTATATAATATATCGAAGTCCTCTCCTTCAAATACTGGAAGGTGGATCACTTGTAGGAATTGTAGGAATGACATAATGCTAATCCATGTTGTACATGGCCAAGGAAGCATAAAATGATTCTTTCATTCTATAGATACCTCTGGTAGGTAAAGCACTCTACTGTGCTTTATTGAAAGTTCCCATCGCGGGGGCGAGGATACTTGCCTTCGCGGTTCGACTTTCTTTTCAGGCTTGACTCATTATTTTCCGGTCCTCTCACACCCCTTTAGAGCTCTTTATGATGCCCACTGAGTAAGATTCGGGGGCTTCCCGGCGCAGAAGCTCATTCTGAACCGCGGGAACCTTCGTCTCTTCGACACAAACGTTTTATGAAGAGGCTGATGGTGATGAGGATCCATGCGCACTCAAATAAAAGTAGCTTGCGTATTGGGTTATCCACGGTGTTAGGTGCTCCATTGCACCCTCATGTGGAGGGTAGGATAATTTGGACTCTTTTGGAGCACTATACTCCGAAAGATCCTATCCTTCCTCCTCTTCTTTCAGTCGAGACTTCCTCCTAGTGAGGTGTTGCCTATCCAGGTATGGAAGTATTCAATGAATACACTCTGTACCATGGGTGGATGAAGCTTTATCTGGAGTATCAAAGATGGAATTGTATGCTAAGGCTTAGTGCCAGTATAGAGCTTGAAGTCTTTATGGATGTGCCGATCTTTATCCGTACATATTACCGACCTGCGAACATGGATGATTCGTTCAGGTACGGGATAATATTTAGGATGTACGATTTGGCTGTCCAGTTACAGTGGGGCAATGTCATGAAATTGCTACCAGTAACTAAGACTCAAGTGGAGACAAAAAAAAAAGCACTCAATTTGAATGTAAGTAAAACACGGGATGGAAGAGGAGGCCTGAACCAACAGGGATGAAACAGTATAGAATTCCCCTGGGCGAAGCAAGTCACCGATTAGTAACCTAAGAAAGAGTTGTCAACGGGCGAGTGTCCTGCTATAAGGTAAACTCCTACTATTTCAGCTTCTTTTCCCGTGGTCGAAAGCTAAATATCTCAAGATGAGATTTTCCAAACTCTCGACAAGCAGCAAATAGAAAATGAAAAGAAGATTTTGAAGGATATGATTTCATCTAAGGAGTGGCGGTTAACAGAAGGCCATTTCAACTTATGCTCTAAAAAGATAGAAAGCATTGTTGATAAAGCCCTCTCTTTATATAAAGAGGGCTTTAGCGCCTTCGGATAAAGGAAGATTCCGATATTCACATAGGTATTTAAGCCTACTTCGGGGATCTGAACAGCTTCCCCTCAAATGGGAGCCGTTTAAGTCATCTTAAAAGAGTCCTCGACTGTATTGGAAAAACAAAAAGTCCAATATGGCGGGAGATCTCTAATTTGATTGAGAGCTTTCAATCAAATTAGATTGTAAAGTAGTAGTCCTGTGTAAAAAAAAGCTGGTGGGGCGGGGTCCAAGCAAGCGTAAGGGGAGGGGGACTAGGGTGGAAGGGTCGTCGAAGGAGATGCATTTCTGGTACAAGTGGTATTGGACAAGATCTCAGGGAATCATCTCTTTCAGATTTCTGCCTTTCTTTCCCATGACGACTAGGAAAAGGCAAATCAAAAATTTTACTTCGAATTTTGGACCTCAACATCCTGCTGCTCATGGTGTTTCACGATTAGTATTGGAAATGAACGGAGAAGTGGTGGAACGTGCGGAACCACATATTGGATCACTCCAGTGCGGCACGAAGCCGCTGACGCCGAGTCGGCTCCTATGCCGCTAGCTATGCCCTGCTTGGTCCCCCGGCACGGTGGAGGTTCCGTAGCGGGTCATGAGCACCGGGCTAAGGGGCGAAGTCACTCGACTGAAAGGAGAGGGCGTAAGCATGACTCGAGCACTTGTCTAGAGAGGGGCGGTTGAGCAACTCAAGCGAACCGCCCTACCTTACTACAACATAGGGACAGAGGGGAGAAGGTTGTGAAGGTGGCCTCGTTATCCACACCTCTGGTCGGATGAATGGAGGACCGCCCGACCCGGGTTTCATGAGCGTTGGCGGGTCCTGGAGTGCCTGTCAAGGGCGCTAGCGCATACCCCGGGGTGATCATCATCACCTGCACCTCACATCTCGGCGTAGTGGAACGTGTAACCCGCCTGCTGTCTCATTCAACTACATTTGTTCCTGTAATCTATAGCCTAACAGAAGGCAGCGTCGAGGGGCTTTAGCAACTCGACTGAAAGGAGAGGAGAGAAATTCCCATACAGCCAGCGGGGAGGATGGCACTACAGGCAAAGACCGTCTGGCGAAAACGCCGCAGGCGCGAAGCGTGGTAGGCCTGCGCCGGGTGAGCATAGGGGGAAAGGGATCCCGGACGGTGGAAGAGCCAGGGGAGGCCGGGTCATTTGACGGAAATGGAAGGAGGAACCCCTCTTATAGAAAGCCCTATGAAGTTAAGGGAAGTGAATGAATTCTTGGAAAAATAGGGAGCGAGCCTATATATAAAATGTAAGAAAGTCAATTATTCAATGAATAGATGATAAAGTCAACCATACGACAGACAGCGCTGCCTACACGCGAATTAGCTTCCGAGGTCGAGCAGTCTCAATTTCACTACAGGATTTGCGAATGAATGCTGGGCTGGGCCACCTCGAATGGCGTGAGCCGCATGCGGGGAGACCCGCACGTACGGTTTTTAGGGGGATCTGGCCGAAAGACCGGCCGGCGCCCACCCGACTAGAGGGACTGAGAAATTAATAGAGTACAAAACTTATCTTCAAGCTTTACCTTATTCTGATCGTTCAGAGGGCGATCGCGGAGTCACTGAATGAAGTCCTCCGTTTCTTTCGGAGGTGCTGACCCGCAGCGAGGCAGAGATGACTAAGTGACATATGGAATATGGCGACAACAACAGCATGTCGTAGAAGGAGAGAACAGGTGGAGCCAACGACCCACGTTGACTAACGTATCTACAACTACATCCCCGAGCGGCAGTCAAACGGAGGCGTGAATGCAAGATGCCAGCGGAATGATCGGCCGGACAGAGGCTAGGGCGAAGGAACCCCTTCCCACCGCGTCCTTCCCTGTGTATCGGAGATATAAAGCGAGTGCACCGGAAAAGAACGGGAACTGGGTCGATCTATTGCGAAGCATCCGAAGCATAACTGCACACTCACACAATCTTTGCCGAGAGATAGGAGCATTCGGTGGAACCGGTGAACTACACTTGCTTCTGGATAGATGTGTGGGACAGAGGGCTCGTGGTACCTTCTGCCCACCCTTCCTCCTCTGCTTTGAGAACTGTGTGAACGGAGAGTGGGCAGAAGGGAAGGAGGTCCTCATACAGAGAAAATCAGGGAATGGGTCGAGATAGATGACAGCGCCGAGGAACGCCGAGGAACGCCTTGCCGGGAGGGCAATCTTCTCTTATGGTCTTCACCTCCCGCCCGGCCTGGAAATTGAATCCAGCCCCCCCCTCTTTCTGATCCATTCATTTCTGCAAGCCCAGAGCGTTGCCTCCCTTCTATTGCATAACCTAAAAAGCTATAAGCAAAGTACCAAAAGCGCGCTCCGCCCGGTGACTAAGAAAGAAATTGGTTTGCGCAACAATTGAAGTGATGAGGCGTTCCTCTCCTTTCAGTCGAGTAAGAAATACCTCGGGAAGTAGGGCTTCTATTGAAAGGCTTTCCCTCCCTCAAAAGGGGACTAGCTTTCAATACTAGTTCTTACGTTACGCTGCCATTTTTCCAATATCATTGAATAGCATGGCCTGGGGCTAAAATAACTCAAGTGGGAGAGCCGTGTTATGGGTGACCTTATTGCACGGTTCAGAGAGCACTTGTGTATGTGATGCAAGTGAACGTGTACGAAAAAGCTGTCGTAAAGTTTCGTTTTTCGTTCCGTTTTCGACCCTATCTATGTTTCTATGATGGCCCAAGAACACGCTCATTCTTCAGCTGTAGAGAAACTTTTGAATTGCGAGGTACCATTACGAGCTCAATATATACGAGTGTTATTCCGTGAAATAACTCGAATTTCAAATCATTCACTTGCTTTAACTACTCATGCTATGGATGTGGGAGCATTAACTCCGTTCCTGTGGGCTTTTGAGGAGCGGGAGAAATTGTTGGAATTCTATGAAAGAGTCTCGGGAGCCAGGATGCATGCCAGTTTCATACGACCAGGTGGAGTGGCACAAGATCTGCCTCTTGGCTTATGTCGAGATATTGATTCCTTCACACAACAATTTGCTTCTCGTATCGATGAATTAGAAGAGATGTCAACCGGCAACCGTATCTGGAAACAACGATTAGTGGATATTGGTACTGTCACTGCACAGCAAGCAAAGGATTGGGGATTCAGTGGTGTAATGTTAAGAGGTCGTGCGACATGAAGACATTGATAGCAATATGGGGGAAGTTCCCATCAGGCAACAATGGTTCCGCCTGACTCTACTTAAGCATGCATATTATGTAAGTGAAGACTTGGTGTGAAGCCTTGGAGCTTACGTTAGAAGAGCAAAAGGCCCGGGGCTAGGGTGAGCTGAGGGGGGACAGCGTAAGTGAGCGAATGTGTGTAAGCCCAGTCAAAGATGACTGTTCTAGGCGGGGGGAGCCACCCACCTTTGAATGGTGTTGGTCCTACAGACCGTGAACGGATTTCGCCTCTGGCCTCTGGGCACGTCGGAACCGCGCGAGTTCACCGGGGTGGAGCACGGTCCGCCAAAACCGGCATAGATTAGGTGCTATTGATGGAACATGGTAAGCCTATCTTTCTCCATATGGAAGTGCTGCGAGCACTTAGAGATGCGGGTAGAGGAAGCCTCAAAAAGCGAAGGCCGAGCTGTAGGTCACGTGACCTGCACCGAGTTGGTGGCTGACTGGGCTTTTTCCTTGATCAAAGCAGATCAACTCGCCTTCTTTCTTGTTACCCAAAACTAAAGTCGGTCGAATGGTTTTTTTCCTGCCCCGGAACGTCGAATGAAATAGGGGGCCGGGTTCTCTTTCTACAACCCTTTTGATATGATAGGCCCGGCTACCTTTCCCCTATCCCTTATGATTAGGGGGCTGTTAAGCCCAAGAACGACCAGTCTTGTGGTGGTACGGAAGGAACGGACTCCGCGAACGTCCCGCGCCCCGGAAAGAAAGTCTCAACCAGAACCACATTCCTTTTGCGTGCGGATGTAGCTAAGTGTCTGACTCTATTGGTCATAGTTTCCTGCTGTTGCGGCTGGTGCTCGTTTGCGCGCGCGTGAACCAACTCAACAAAGAAGGAAAGGATGCCCGGGGAGGCATCTGAGAATGATTCGAGCCGTATGAAGGGAAACTCTCACGTACAGTTTGTTTTTTTTGGGGGGGCAGGAGCCCGACAGGGTCCCCCACTGACTTGGCCCGGGCCTAAGTGAAAGTGAAGTGGTGGGCCTACCCATCCCAACCAGGGGTATGCTGGGATTCGCGAAGAGCAGCACCTTACGATGTTCATGACCAATCGGATCCTGACGTACCAGTAGGTACCAGAGGAGATCGCTATGATCGTTACTGTATCCGTATCGAAGAGATGCGACAAAGTCTTCGGATCATTGTGCAATGTCTTAATCAAATGCCTAGCGGCATGATCAAAGCCGATGATCGTAAGCTATGTCCTCCATCACGATGTCGAATGAAACTATCCATGGAATCGTGCGTCGTGTGAAACGTAGATCATCGCCGTTCTTAACCGAGACTCAGGTTAAGCTCCGTCTCGGAACCTTGTGGGTTAGGAGTAAAGCATCCCGAGGTTGACGCATCTCATTGGGCGTAGAGAAGCATTGGGAACCCCAATTTCTTTCTTCGGAGCCGTTTCTTTTCCCGTCCCCCCACCCCGGCATAGCGCTTCGCTTCCGGTTCTTCGGAAGAATCAACTTACTTCTACCTTCTTCATTGATCTGGGGGAAAAGGAACCGTCTACCAGTTGGGAAGCTAGACATCAAGTAAGTGGCTTGATGAGGATAACTAAGCTGACACGCCGGAGTTGGCTGCTGGCACAACAGGGTGGTGCCTTACCGCGCCGCAGGCGAACGCGCGGTAGCGTTCGTGGTGGTGCTTCAGGATTCCAATGTACTGCGTCCAAGATCAGAACGAGCTTGCCGGCGGACCACTGCCGTCCCATTCTTGAGTGAGCTGGAGCACAGCCATCTTATCCACTGAACTAGCTAGAAGCTATCGCTTCGGGTCGAAGCACTAAAAGAAAAGAACCGGGAAACGCGGCGGCATAGGAACCACGGGACCCCCACCCTACTAGTAAAGGGAAAACGGAAGTGCGCTCCTGCGCACCAGCTGAAAAAGCCCTTTCCCCTTTCTCTGATAATAAGGAAAGCTTCATAGCTCCAACCTATACAAGGGGTTTTTATGTCCTTTTTATAGGTTGGGTTGTTGGATACGGGATCCTCGTAGTAGGCTGGACCAACATCCAGCCGAGAGAGGGCAGCCTCTAGAAGCAACAGGTTGGGAAACCAAGAGAACGCTTCGCCTTTTCTTATCTTCTTCCTGCCCTAGGAGTAGAAGTAGCACAAAAAAAGAGGGATTAGCATTATTGACCCAATGATAAACCACTAACACCTTCCTCGTTGGGGCCCCGCGCACTGGGAAAACGCCGACGCTACGGGAAACCGGCCACTAGTTACAAAGCTCCAATAAGGCGTTCCTCGAGAGGGCTATCACAGTCAGGTGCGGAGCAATTACCCCTATTTTTAAAGTACCCTTCTTCCTATTTAGGGGGTTGAGGCGAGAAATGGCTTGATGAATCGTTCCGTTCGCCATGCACCGGCCCCATTCACTTGATTCTCGTAGAGGCTGTAAGTACACAGTGCCCCACAACTATCAATAGTATAGTGGGGTTGAAAGACGAGAGTGCCCGCCCTTTTCTTTCAAGTGGGCCACTTTTTTTTCCCGAACGCAGTCCGGGATCACCGTGGCCGTGTATATATATACAAATATCTTCGATGCTGTCATTTCGAAATGTCCGCTTCAACCCCGAGGAACGCCTCCCAAAAAAAGCAAAGTTGGCTTGACGAGCGCAGATGTGAGGAAGCGGGAGCAATAAAACAAAAAATCTCTTTCTTGTCCTTCTACTTAAGGGGCAAAGAGAAGCGCTTTTGCTACTGAGAAAGCGAACGGTCAGCGCGAAGGTTCAAGACTTTTCTGAGCGTTAGCGAAGCTAGATTCTCATAGCGAGGCGCTTCGAGTTAGCGAAGCGCTGTAGTAGCGCCGAAGCCCTATGTGCTATAATGCTGAGCCAAGGACACTCCGCCTTATCTATAGAAGCAGTCAACTGAGTTCTGAACGAATTAGATCCTTGGTAATGGCTCAATCTATAGATAGAAAGCCTTATGATGGGAAACTACCACGTTAGGTTTGGAGAGAGATGGGACCGGTTATATAATAGAGGGAGCAGATGCAAGCTTTTTTCTTTCAATAGCCGGCCAAATGACTACAGGATCATCGGTCTACTCTACCTCAATTCACCATTTCGAACTTTATACAGAAGGTTTTTCCGTACCAGCTTCTTCTACCTATACCGCAGTTGAAGCACCTAAAGGAGAATTTGGTGTCTTTCTGGTCAGTAATGGAAGCAATCGTCCCTACCGTCGTAAAATAAGAGCACCCGGCTTTGCCCATTCACAAGGACTCGATTCTATGTCCAAACATCACATGCCAGCAGATGTGGTCACCATCATAGGTACTCAAGATATTGTGTTTGGAGAGGTGGATAGATAGGACGACTAGTTGCCAGGACCTTAGCTTTATTGCGAGCCCAGAAGTCTCTCTTTTTTTTTCGGCCTTCAGGAACAGCCTTTAAGTCAAATCCAACCTAATATCATTCATATCCTTCTACATAGAAGAAAGACACTCTAAGATCCTTTTTCAAACCTGCTCCCATTTAGAGTCAAGAGATAGATAAATAGACACGTCCCATTGCCACTTGATGGGGGGGGCGTTCGTTGTATGTTGAAGCAGAGATGAATAGGGTGACTGTGAAGAGAGTGGTGGTTGATCCTGACTCCACAGTCAATCTCATACCTATGTCCACTAGGCATTCAAAAAGAAAAGATTGGAGGAGCACTTCTCGAGGTATATAATTTGAGTTCGAATAATTGTCAAATCAATTTTCTAGGAATGGTATGGTCAGAGTCAATTGCAACGTTGGACCTTTTCAGAGTCCGATAGAGTTCCAGGTTGTTTTTGCACCGACGACATATTACGCTCTTCTAGGCAGACTCTAGATTCATAAAAACCAAGCGGTCCCATTGACATACCATCAATGTATTAAAGGTATAATCAAAGGAAAATAAGTACTCGTACCAGCGGTGAGCACTCCCTTCGAAAGATCTGAAATCCATTTTGCTGACGCTATACATTATTCGGAGTTTGCCGAGGATGGAGAGCTTTCATTGAAAGAGGTCCAGGGAGCTGTTGGCTTACTCGTTGGGAGGCGTTCCTCGAGAACTAGATAGCTAGTCTGTCTGGTTCTGTTCTCGAGGAACGCCAGTCTCTCGACTGAGAGGAACGCCTCTTACTAAAGTAGTTCAAATAGATAATCCACCTAAAATGGAACATTCGCTTCTAGATCGCTTGCTATAAGTAAGGAAACTCCTGAGTACGGAATTGAGACCGAAGAAAGGAGATTCCGTAAATTGACTCTGAAACAAGAGGCAGGAACTCTTCTGCTGCTGTTTGCAAGCTCTTTCTTTAGCATTAGTTAGCATTAGAATCCCGCCCTTCCTGACGGAGCTCTCCTACCCTTCTCAATGCAGGAATTCGCATACAATCGACCGGTACCATAGCTATATTCAATCCGGTGGGTCAAAGGAAGGAGAGCCGGATTGCAGATCGGGTTAAATAAAGCGGGTGAACACTAGCAACCAGTACAAAACTACCCCGTCAAAGACGGAAAATTGGTATAAACCAAGATAGCAAATGGAATGATTAACTAGTGCCACCCCAAACCCACAAACAATCTAACCACAGGTATGGATTGACACCGCGAAGGAAGTCAATACAGGAGGGTTGGGAGTGAATGGAATATCGAGATAGATATCAAGATATCCCATCAACCCAAAACCCTTAACAAAGCTAGAATGCTTATGTTGCGTATAGTAGCCAGGGGGCCGAGCAAGAGACCTCCAATAAGGTCAAACCAACGGGCAAATCAGTCCCAAGAGTGAAAGGGAGTTTGAACTGTGAACATATAGGATTCCGCTAATCCTTATGCTCTAACTAGTCTTAAACGAAAGAGATATCGATTCCTTTCCCTGGTGTACTAGACTTGAATTCCTTTGCAGTCTTAGGCTTTTGAGCTTTTTTCATTCAAAGCGGGATGCTTGCTAGACTGATAAGCCAGAGAGAGGAAAGGAAGGAATCAATTATTGAGTGGGAGTAGGGTCTATGTCTATGTTGAACAGATTTGGGGTACAGTCAATTTTCGTGCCATGATCTTATCCATAATGTCCCCTTTTAGAGAGCAGTAGAATTAAGAGCTTTGAGATCGTCAACACAAAATCCGATCAAATTAGGATAAAATGTACTGGCTGGAGGGACAGACTCCAGAAAAAACCGCGGTCATACTTGAATCAAACCATTTACTGTTCTGAAACTCCATAGCCTGGAATTTAGATAGGCGAGAGGTGCTCCCTGTAGTTAGTGAGTCGTCCCGGCCTTTAGTCTTGATATTATCCGAAATGGGGAGAGTTTATACGAACCAGTAAGAATAGCCCGTTCCAGTCTAACGAGATTCACTCTCTAGTAAATCAAATCATATATTTTGACCGTCTCCTGTATTTGTAGGATTCTTAGGTGTGGGTTCAGGTTTAGCAACAAAGTGAGGCAGAAGTTCTTTCATTCCTCATTCACTTCTAGGACTTGGGTTAGAGAGGGGGGAATATAGTAAATAAATAAAGTAGAAGTGCAGTTCCTATTTCTCAGCTCAGATTAGAGCACCAGCTGTTACGCTGACAACCCCCGTTACGCCGCGCTTGAAATCCTAGCCCTAAGACGGAGTAGTCTTAGTAGCAGTAGGATTACCGGATTCCGCTTTATGTCTTTCTTTCTTACTTTCCCTAAGCACTCATTGTATTTGAATTTGACCCGCTCAGAAGAGCTATGTATCCGGTCTTGGGAGTCAAATAAGGGCATGGCTTAAACCAGCTCCCTAGCCTAGGGTGAGGTCTCATAATAGAGTAAAGTAGGACAGAAGGATTAGCTTAGCAGTGAACAAAAATCATTCAATCAGCTCAAGAGCTGGGAGTTCTCCTTCTAGGTTTCGGAATAGAAGGAAGGAGGCTCCCAGGAGGTAAGTCCTGCTTGATTGCTTTCACCAGGTTTATAAAGACTGGTTCGAAAGGACCAGGTCGGGATAGGCTGGGAGTCGATTAGCCCGAGTTGTGTTAAGATAAGTGGCACTTGAATTTGAAGTAGACATCGGCCAGGTCTTGGATGCTGAATTATCGGTCTTACCACTTGTATCGATAGACCCACTTGAATTGAATGCTGAGGAGATTGATTTTCAATCCAATCCTGCCTGATCTTCGAACTTTCTCTCTGCAAAAGGCTTCTGGCAAAGCTTGAACATCCGTATCGTTAACTCCTTTTAAAAAGGAAGACAGAAGAGCTGGTAGGACGGACTGGTAAGGAATTCAATTACTTATTACTTATGAGTGGAGGGCTTCGCTTTTTTTCTATAACTGTCACTGGAACAGAAGGCCTAGTACTCCAATTGGCTTAAGTGCACTCCCAATGATATTCTAGTCTTCTTTCTTAAGAAAATATAGATATTATCGTGGAAAGAAATCTATCTCTTGAAGGAAGTCCAACTTAATTCAGTTTGATCTCCCAAGAGACGGTATGGAACGCTCGTATAGAACCCCATCCAATAGAACTCAAACCGCTGGGAACGCAACGGGATGGATGTCAACTGGTAAGAGTAGCCTTGGTCTGTAGATCTTTACAACAGGAGATCAGGAGTTAGCTCGCTTGGACGGAGAGCACGAGAAAGAAGGGGCAGTCTAATTGAACTCCACTCGTTTAGAAGAAGCCTCGCCAGTCAGGGGGATAGGACAAATTTCTTCACAGCATGAAGAAAAGAGAGGGATTCAAAAAAGGCTGGCTCCTTAGAAGGGATTTGAGTGCTTTAGACTAAGAAGTGAGATTAGATAGGCTTGTCTTAGGATTTGATGCACATTCAAGGGCAACTTCTGCTTATGGGTAGGCACCTAGTGCTACAATGGCTGTAACAGATTCAGAGATTACATTTGCCCTTGGATACACACCACACATTGATTGGATTTGAAAGGTACGAAGGGTCCATAACGGACAGGAACTACAACTATTGATACTCGGTCTAGACTAGGGGGGGGAAGGCACTGCAGGAAACGGGACTGCTGTACTGTAAAGGGGTTACCGTCGAAGGTAAGGACAGAGACTGATTAAATGGGGGAGGACAGATATAGGCAGACACTTCCAAAAGGCGGAAGCATTGGCTTTTAGGACGCACTCAAACTTCCATTAAAACTCCAGGTGGCAAAACACAACTCCATCCAATAAGAAAGAGGAATGTGCTAACGCCAATAAGCGTATAAGAAAAGGACTAGAACTGGCTGAAAGCACTTACCACTAGAAATAAATTGGCCTGACAGAAAAAGTCAAACCCCTCCTCTCCCTCTCGCAATAAATGCTCGAGTTCCCGAGGAACGCCTCTCCTTTCAGTCGAGTCATGACTTCGCCCTCAGGTGGGAGGAGAAACTGTACAGGACATAGCTTTCCGTCTCTTTCTTTCACAAAAAAATGAACCACCAAAAACTGTAGATTTCTTTTTTGAAAAGGCCACAAAACTCTTTTCAAAAAAGAAAGAAGTGCTACTCAAAATGGCGGAACTAGATCCTTACGGATCGTGGATTCAGAGTGGCGCTCGCTTTATTAAAACCGGAAAGACTTTATCGTAGAAGCGGGACTTAGAAAAGTCCTCAAATCTCTTTCTGACCATGGTGTCAATAGCCCATACTTTCAGTCTTTTTTAAAAAGACAACGTGAAGAGGAATAGATCAACAAAACAAAGAAAGGACAGGAACAGGGGTTGTTGCAGAATTGGGTTCGAGTCCCAGGGACGCAATGTGGCTGCTTAAAAAACTGATTCAACGAGATATAGATATGTCCCCATTAAGATTTCAAACTTGTCGTCTACTTTCAGGAAATGTTCGGAACAGAGAACTGACAATAATACAACGTCGCATTCTCCGAAGATTGAGGAACAGGAAGAGATCTATTAAGAAGAGAAAGATTTATCCGAAAAAATATCTTACCAGTTACATACAATTACAAACTACACGAAAGTTGCCCCTTTTTCATGGGGATTTACCCATCACAGAGATGCACAGAGGAACAAAACGAACTTCATATATCCCTTTTCCACTCAATCCAGAAACAAGATTTGACGTTATTCCGCTTCGTCTCCATTTTCTTGAAACTATTCCTCAAGCAAGGCAGCCGATAAGTCATCGAAGGGTTTGTGTGAATAAAGGAATGGTAAGCATTACTCATTTGAAACTTTCCCACGGTGATATAATATCTTTTCAAGAAAATAACGCGATAATACGCGGTGAAGAAATAAGGAGATCTTTCTATAAAGAAATTTCAGTTGAAAAAATCATAGGCAAATTACTGCATCAACCGCTAAGAATGTGGAGAAGAAGCAAAACTGAATGGTTCCACCTACTCAAAACTAAGAGGGGATGCCGCCTACTACTAAAATCCCGGTTTTTGCAACAGTTGCGTTCTTCTATGCAAGAAGAAGACTTAGAAAGAACAAAGAAGTTTGGATCCGAAAAAGTATGCTTAGGAAGTTCCTTCGCTGAGCACAAGAGAATGAAGAGGAATTTGTTAAAATCCCTATTCTTATCGAAGAGAAGGAAGGAGAAAAACCTAAATCTTCCTACTCGAACAATCAGTCCTATAGTTTACAACTCTTCTTTATCTTTATATAGTAATTCGACCTATTGCTTCGCATCCCCCCATAAGTTGACTATGAAGAGAAGAATCAAAAGGATCGAACTACCTACTCATTATTCGGAGGTTAATCATAGAACACCAAAAGCTGTGGTATCTTATGGACCTAACATAGGTCATATCCCTCACGACATAAGATTAAAAGATCCAAACCTTCCTCTTCGGAGCAGAAACGGACGTGGCCAAAACATATAAAGATCGGCGTAGTCACTCGTAGTAGGAGTCAAGATATTGCGTATAAATATATATAAAGAGAGAGTCTCTTGAGCGGCCGAGAATCTTATGTCAAAAGGACCAAGGACGATCTTTTCGGAAAGGAGGAGTCAGTCTTCTTTGAAGATCGAGGCGCGTAGCGGACAATTATGCCATTCGGAAGAAGTCTTCTACAGAGGGAAAGCCTGTATCGAGTAAGTGGAGAGGAAAGATCCCCGGAGATTCTGATATTATTCCATTCCAGTGGCTCGACCAGTAACCAATGGAGAAAACTCAAAAATCCTTTGTTTCCCGGTAGAACGGAGCCAGATAGCTTTACCAGTTGGGAGTTGTCCGTGACGAATTCCGATTCAGTAGCTATCAGTTCAGAGTAGATCTGTCCAGAGGTCGATTCATCTACACGTCTACATAAAAGCTTGAATTTCACAAGGTTCACGATAGGATTTTCTTTTAGTGGGTTGACCTAAGCCTTGAGCTTCACTAGGGGGAGGCCAGCTATCTATGGTATCCTCGGTATGAGGATGAATGTCTCTAACCTAGCTATTGCCCTGTTTTGATCCTATTCAATCGTATAGAAAGCTACTATAGACCCTAGCTATTCCGCCCCTTTCTTTTCTACATTCCATTTTTTATTGAGCCGAATCACCATCATTATATTATATATTCATTGTTGGTTTGACTGCTGGTCTAGCGATCCTTCCTAGCCGTCGCCTAGAGTGCAGCCTGCCCGCTGAAGACCGTAACGTAAGTGACTCAGTGCTCCATACGGGGGAAATGAAAGCAGAATTCGTTCGGATCCTCCCACATGTTCAATCTTTTTTTAGCGGTTTCCCCAGAGATCTTTATCATTAATGCAACCTCCATTTTGCTCATTCATGGAGTTGTATTTAGTACCTCTAAGAAATATGATTATCCGCCGTTAGCCAGTAATGTGGGTTGGCTTGGATTACTTAGTGTTGCGCGCCTAGGAGGGCAGCGCGCTTGGGGATGCAGAGGAGCTATTATCGCCCAGCCCCCTACCCTAACCTAATGCGGCACCGGATTCGGACCGCAGGGAACCGTAGCATGGGGGGGCGTCTAATCCTTTTGCCGCCGCAAGGCTGGCTAATCGTACGCAGCAGGCTCGAAGACCCCTGGTTCTGGAAGGCACATGAGTCCGAACGCTATGTTGAATGTGCGACCGACACTACGTAGGTACCAGTGCAGGTGAGGCGTCGGTCGGTCCTAGAAACGGCGGCAACGGCGCGAGGAGTTAACGACCGGACGTGCTGCAACCTAGGGATCACCAGGCAGCTCTTTCGCTCTATAGGGATCGGGGGGGCATAGCACAATTCCTCTTGGAGGGGGGTTGGTTTGCCCGGGTGACTGATCCTGCCATAATGTACTCCTACCTATAGCACCGGCAACCGAAGTCAAGCATACATAGGACGATCTTCATGCGTGAATAGCCGGGAGGAAATAAAGGGCGGTAGATGATAATGAAAAAGGGCGCCGCGTCTGTACGGGCGGGCGGAATGGATGAGCAAAAGGTGCCATGGGGTGAGGAATATCCCGAGTCTCATGTAAGACAACTAAATGCACCCCGCCGAGGTGCTGCCGAGGAACTGGGAGACCTTCTCGAGCACAGGATAGGCAATTGAAAGACAAAGCTAGCCTATTCGTTATGGGGAATCAATGCTCCGGGCCGAATAAAGCTTACCTCCGGCACCTGGCTTTCCCCGGCGCATATTAGCTTAGCTGTGCTTAATAGGTCGGTTTGTTTGGCTTCCTCTCTTAGCCCATTCCTAACCAGTGGAGAAAAGGTTCGCTCATGCTGGAGGGAGCATCCCCACTTAGTGATCGGTCTGCTAGTTCACGCAAATCCGAAGAAGTTATCACGGACGAGCCACATGCAGGGAAACTTGCACGTGTGGTTCTGGCCGGGCTTTCCTGAGGTATCTAATAACCTTGCTTCTGCTCGCCGCTGGCGCACCTCTCCTAACTATTGCCCATTTATTCTGGAATAATCTTTTTAGGAGGGACAATTTTACATATTTCTGCCAAATCTTTCTATTATTAAGTACGGCTGGTACCATTTCGATGTGTTTCGATTCTTCCGACCAAGAGAGGTTTGATGCTTTTGAATTCATTGTATTAATTCCACTTCCTACTTGCGGTATGCTCTTTATGATCTCGGCTCATGATTTAATTGCCATGTATTTAGCTATTGAGCCTCAAAGTTTATGTTTTTATGTAATCGCAGCATCAAAAAGAAAGTCTGAATTTTCCACGGAAGCCGGCTTGAAATATTTGATCTTAGGTGCATTTTCCTCTGGAATATTATTGTTTGGGTACGACCGGACAACTACCGATATCAATTAATATCTTTTTTTAGAATGTTGTTGTTAAATAGATAAATATCTATCTATATTGTAAACTATCGGATCGGGTATTACTTAGATGTGAAACTTTAAGACCTCATTAGTTGTGATATTGATCTTACGGGGGGGGGAACGAAATCAAAGAATATATAGACTTGTAAAGACCCTCTATGTAGTTGTCTATCTAAAGGCGATCGATCTACATCTTTCTCCATAGCCCTTGGGCTGTGTCTCGATCTCCTACGTGCGAAATCAGAGGGACCTGTTTCTATGGAGATTCCCCTTGGTCTAATTCCACGCCTTATGACGAAAGGAGAGTCGGCGTGGCATCAAGTGAAGATTTAGGGAAGTATAGAAAAATTCCCTTCTGGGGTATTCCGAAGGTGTAACCTAGGCAACGAAAAGGGGCCCGATACTTCAACTAGAGGAGCGACCAGTTGGTTCACCAAACCCCGACCCAGCGTCACACGTTCTCCAGGTCCGAAGGGATCCAGTGCCCAACTACCGACTCCTCCCGGAATTTCGTTATCGGAGCCGAGCCTGGAACGGCCGTTAGTGGACACCCACTACTTTTCACCGGTTAGAGAGGCCCTCTTTAATACATTAAGAAAAGATGTTCACAGGGGCCAGAAAGACTCGGTCATAGGAACTTAGACCACCTACGCGCTGGTAAACGAAAACCACCTCGACCGGATCAGAGTGAAACAACAATGTCGAAATCAGGCCGCCCCTTGCCTTGAAAGAATTCACACGCGGCCACCTGCTTTCCCAAAAGAAGGGGAGATCCGCTGCTTACTGCTCACGGAAACATCCGACCTATACTATAGTCAAGTTGTCCGCCTATCTTTCTTCTTTCTTTTTCTTCTATTCACATCAGATTTTATACTTTCACCAATTCAAGGTGAAAAGGGGGAAAGAAGAGAGCTTTGGGATTCGCTCACTTCTGAATTTGGAGTTATCGAGATTCTCAATGTGGGGAGGCGTTCCTCGGGAATGGCGGTTCTCAGACAAGGGAATCGTTCCTCTCTAAATCAAAATTGGCTAGAATGTTTCTTATCAATAGAGCTTTAACGTCTGCGCATAGAATCGCTTTTTTTGCTCGATAAAGTCCGTTCCTCACAGCAGGACCAGTGTAAACAGGTTCTAGGTCAAGCGATCCTATAGGCAAGTTTGAAAGTCGTTACCCCCCCAATTAAATAGTATTCCACAGAAATTGTTAGTTATTCTCCTGCATTAGATATTTTCTAGCAATCTTAAGGTTAAGGCGAGCAATCTGTAGGTATTCCTCCGCTGCCCTTTATAGCAGGGGATTCCAGTCGAGGCTTCCCCTTTCGAATGATTCAGTGCTCTCTTCAACCGGCGAGTTTAGCCTTGTCAGTGCTCTCAGAAACCAGATCAATTAGGTCTGTGGATCGGCTCACTTTCCCAATCGAAATGAAGGGCTGGCAGTCACTCATTGACAGCTTCTTGGAAGTCAAAGGGAATCCCCAAATACCGGACAGGAAGCTTTGCAGGTTTGAGACCGGTCTGGGTTTCAATCAGTTCAAGATCTTCCGCAGGAATGCAAGAGGCTTGTCTTTTGTATGTTGATGGCAAGACCCGACATCTGTTTGAACTGATCCAGAATGGAGAGTACACCAGCCACTGAACGGGGAGAACCTTCGGCAAATATAAGCAGATCATCCGCGAAACAAAGGTGTGTCAATCTGAGGCGCGAACAGCGGTGATGATATCCAAACTGGTTTTCCTCGGCACCTCGATTCAGCATATGACCCAGAACATTCATAGCAATAACAAATAAGTAGGGTTAAAGCGATGAAAGTATAGCTGTGCTCCAGTTTTTCGAGTCAAAGTTCTATGGTGAAGAGCCCGGTCAAGGTGACCCTATGAAAGTCAAGAGGTAATGAAGACCTATAAGATCTCCCACATTCCGGCTGGCTTCATGCCGAAAGTATGGACTATTTTGTTTGATATGATTGACCCTGTTTGGTAAAAGAAAGTTCGTGCTATCGGTCATTCACGCAATGAACAGTCTAAGCTCAAATTTCACCGATTTAGTTTACCCCATAACCTGAAGCATCCGAGCCAGCAGGAGAAGCACAAGCAGGTATTTATTACTCGCAAGGGCAGGGAAGTCAAAGCAAAGACGACGAAACCGGTAGCCGAACCACCCCCTTTTTGGTTAAAGCGGACTCATCAAAGGAATCCCCAGCAAGAGGCTCTCCATCCGAAAGAACTGCTTAAACTGGCATCTCCAGGTCGAGTACCAAGATCGGAAGAAAGAAGAGGTGAGGCGTTCCTCGAGCTTACCCGAAGAGAAAAGACAGAAGTCTAAGGGAAGGCACCTATTAGCTTTAGTAGCCCCTTACGGAATGAAAGGAAGGTGCCAAGCCGAAGTGTACAAATCTCATAGGTCTATATCTGCTCAGTCTCTGTTAGCAGCTTCAGTAGGATTCTGGTCTTTTCTTTCCTGCGAGTGTTGAAGTGGGGAAGTCCGGATCAATCCGTCGAAAAAGACGCCATCTCAGTCTAAATGGAAGTAGACCAAGTAGTTTCATAGGAAGAAATGTGAAAGTAGGGGCTGCTAAGCGCCCAGACCCATTGGAGGTGAGGTGAGATAGTCAATGAATGGGGTGGTAGATTCTGGCGCAGTGTCAATTGAGAAAGCGATTGTGACTCTGACCTTCTTATCGGCTGCTGGTCTTGAAGTCTCGCTAACTACTTGGATACCTCCTCTCATTCCTCGGAACGAAAAAGCGAGAGTACATTCTCCTTTTTAGTGCATTTCAGCTGCGTCAGCAGTTTATTCTTTCTTATTCCCTTTCCTGTGTTCATTTTTCAAGTGGTGGATAGGATGAACTTACTGGCCTAAGGTCCTTCTCTTCTCTTTCTTATTAGTAATCTTGTTCAAGTGAGAGGTCCAACGGAACTCGACTGAAAGGAGAGGGACTGACATCTTCTTTTCATTTCTTTCATATCTCCCTTGATTCTGTTTCAGTTTGAAGTAATAGGGAGGAGGCTTGTAGACCTTACAATTGTCTTTGTTAAATACTTATGACCGACTGACTTGAATCTGCTTGCCATTGGTCCTTATCTTTAAGATAATGGTAAGAGTGGTAATGTAGGGCTAGGGGTATAGAATGGAGAGCCTTTGTAGGCCGATAGGTTGATTGAGTCAGTACACCTGACGTATGCTTAAAGAAAAAGAATGAGATTCAGCTCTGATCACCACCACCGGATGCATTTTTCTTGATCTTCTCGGACTCCAGTAAAGTTTATGCGTTGGGCCTAGAAAGCGGTAAACGTCAGCAATGAGTGCTTCGATTGTATTAAATTCTTCTTCTCGATTGGGGAAAGTCCGTTCCATGCTAAGACGCACCAGATCTATGATCTCTATTTTCTACAGAATCGTCTAGCGAGAAAACTCTTTCACTAATCTTTCTTCTTGGCACTTTCGGTCTTCTGCAGAATAGTAGCATTGCATCGTCAATTCAGAATTGATGTGGAGAGCTTGTTCAAAGAACTCGAGTCAACACGCTACTCTGGACCTGGCTCTCTTCTTCATACTTACTGCACGCAGGATAAGATCTACTTGTCCTCCTCCCTTCCCTTTACCTTTAAATGAAAGCTTTCACCCTCTCCTTTTAGTCGAGTAAGAAATACCTCGGGAGATATCGATCTTGCTTCAAGCATGCAATCTTCTTCACTAGTAGGAGAAAGAATCTACATATTCAAGTACAGGTGATTCAATAGCAGCAGACCTCGGTGGAGAGAGGTCTTACTAGCCTTCCCGCACGACTGAGCACAAGAAATGTGTTTTGGTTGTGTTGCCAAAGGAGGCACCACAGTAGTTGGTTTAGGCCACAATAAATGACTACCTTAAAGGGGGAGTAATTGGGTATGATGCCTCCTATTCAAGGAGAAATCCGGTGTTCGTCACACTGGACGCCAACCATCGGACCTGGTTCGTTAGAAGCTCCTGGTGGCCTGAGAGGAAAGAGAATTTCGCCCGAGGAACGCCTTTGGAGGAACCCCTGACTTGTCAAACCTGGTACTAGAAACTTTATTGTCTCTTCTTCACTGATCTGTTAAGATAGCTACTTAATTGAGACGTGGATTAGGATTAATCAAGGGGGGTCAAACCTTCTATTATTAGGGTGGGTTCGTCCCGACTATCTTAACCTATATGGTCTTTATTGACAGCGAAAGCTTTGTATGACTGTTCCGGTAGAGTAATGGGGATAAGGGCGAACTATCTCTTCTCTTCTGTTCGTTGCAATGGAACTGAACGTTCCAAGTGCATCCAGCAGGAATCGAACCTACTTTCCTCTTTATTAGGTTGGGCGCTTTAACCATTCAGCCATGGATGCAAGCGAGTGAACTAGGTTTTGGTATTCCTTCTCGAGCTTCAATTTCTTCCGTTATAGGAGATTCGAGAAAAGATGGAATAGGAAGACGTGTTTCCAGAACAAACAAGATACGGGTTGAGAAGGGGAAGTTAGCAAAGTTAGACAAGATTGGAATGGGCATAGGAACATGTATTGATGTACCAGATCGGCTAATGCTCCCAGGTTGATGCGATGTATTCCACCAGTTGACTGAAGACTTTATTATTGGTATATCGATCGGTCCAGCACGAATTGAAATAGAAGCCGGTTCGACAGGAAGCTTTTGAAAACGCAGTGCACCCAGGTAAATAAGAAACGAGATGAATACAGAGGTCAAACGAGCATCCCACACCCAAAAGGTCCCCCACATTGGTCTTCCCCGAAACCCCCCAGTAACTAAGGTAAACAACGTAAAAAAAGCACCCATTTCTATACCGGTTCCGGAAGAGCGAAGATAAAGGGGATGTTTTGTTAATAGGAACAAGAAAGTGTTTATAGCCGTGGCGATATAAACAATAATACTCATCCGAGCCGCAGGAACATGTACATAGAGAATACGAGAATTTCCACCTTGTTGAAGATCTAGTGGTGCTACCCCAAGACTTAAATGAATAGCCATCGCTGTTAAGAACAACCAAGACCCAATGAGAATTTGCGCGTAGCTTCTGGTCTTTGACATCAAAAAAGAAGGTTGTAATAACGGAACGGACATGTGCAAATTTTGTCCTAGCTAGTGGAACAAGAAAGACATGGATCTATAATACGCAATCAAAGGATTTCCCCCAACGAATACCCTGCTTTGTTTGTTTTCGCTCTGCTTGTGCGTGGCACTCCTTGCTGGCGTAGCGGCGCATAGCGAAAAAAATAAAAAAGGAAACTCACCACCAAAAGAAAAAGGTAGGTTAACTAACGAAACTAAAAACTACATTTGAATTCTTGCTCACCGATAATCCTTTAGAAAAAATCTTATACAACTGTATAGAGGATTATTGGTATAGCTGTCCACTAGCCGAAAATCGGACACCAAATTATTGTGGACTGAATGAAGTAATTGCATAGCTGCCGGGTCTGCCGTATTTGGCAATACGGCATCCTGTAGAAGTTGAGCGCTCACCTTGCTCAAGAAACTTGTCGTTTCTATATAAGGGTGACTTCGGAGGGAACTCGGACATGAAATTTTTTTTATATTTGTCTAGATACTTTTCCACTTCCTCAACTACAAGATTTCTCAAGAAATCCTCCTTCGCCTTTCTTTTATCCTTTCTTTTATTTCTTGAAGAAGAAGAAGAAGAAGAAGAAGCGGGCGCACGAGAAAGATTTAGCATCATGTGGATGACGGAGAGGGGGAAAATTCCATAAAGCGCGAACCAAGATCCATGATACGAAAACCAAAATCAGAAGTAGGACAAAACTCAGAGTTAGCAGCATGAACAAAGAACTAGGACATACCAGGTGAGAAGTTCGGCTAAGCCGGAAAGATAGATCTCTTCGATCCTTGATTGATTTGAACAAAAAGAAAGAAAGAAGCCGGTAGCAAAGGAAATGAAATGGCAGCTGTGCTTTATATAACATAATAGCTTTTTGGTCTTCGTTGATCACTTCTGCTTACCCCACTGGAAGACAGGAAGAGCTAGCATCTCTCTTTCCTAAGGCATAAGGCGTTCCTCGAGGAGTACAAGCCAAAAGGTGCCATAGTCGTCGCAAAGGTTCAAGCAAGGAGGAAGTAGGTCTCAAAAGAAAAAGGGGGAGGTTTCAGAATTCCCAAGCGCCTAGTCAGTTGAAGGGTCAGTTCAAGGTTAGCGTCCGATTGTGCGCAATGAATAAATCAATGTATTTGCGGAAAATGGGACGCTGAAGGGCAGGGGTAGCCCATGTTAGCAACTTCTTAACCTGGCTGCACCACCTTCTTTTTAGTGAAAGGTGAGCTACGGTGCCCCAAACCACAAGCAAAGGTTCTAGGAGTAGAGATCTCGTCTCTGCAGGTTCGGAGCTCCTATCTCCGTACCGAGCAAAGCGCAATGTGGTAATAAATCAAAGTCTGGGTTCGGTCTTCCGGTGGACGGCATCAATCACCGGTGGTTTGTTTGGGTTTCAACTAGAAAAGTGGATTCTGGCTTCAATTCTTAGCATGAAGAAGATTATCGTGGACCGCTTGCCCCGGTGTGAACTCATGAGATTGAGAGAGATGGGGAAGTAGGCGAAGCTGTTTAAGTAAGAGTTACCTAATTGATTGAATACCAGTCAATTTCCCACAAGAGTATACTCATTTCCGCAACAAAATGGAATTGCCTTTGATGCTTCGAGGAACGCCTATGCAATCACAGCTGAGTAATGGACTGGCGAACTAAAATGGATAGCTGCTGGGAGAATTGCGACTGATGAATCGCGATCAGCCGCTGATTCCCTTGCCGTTGGATTCGTGCCTCAATACTCTGCCACTGGGACTCGGTCGGAAGAATCTACTGCGGCCTTCTCTACCCACCTTTATGAATTCTAACCCCAACCAGCCATGACAAGGCTTAATTTATTAGAACCCGTTGTTGTTTAGAAAGATGTGTTATTCCTGTGAAAGTCTCGTTTTTGACTCCGTTTTTTTGAGCATTGTACACGGGCTTTGCTGCGACGAGGATGCCTTTTTTAATAAGGCCAACGTCAAGACCTGAAACAGAGTCCTACCACGTTAAGGGAGAGCACCCAACTTGCTTGTTGACACGTGAGGGAAAATAGGAGTCTGAGGTGGCAGGATTTACCACTATAACCCTATGGTAGTGATGGTTGGTCCCAGTACTCCTTCGAGTTCCTTTTGCGCTTAGCGTCGGAAAGAGGAGTTGCTTGCCTTACCACACCAACCACCTTAGCACTGGAAGTTCTAGCAATGGGCAGCTAGGCAAAGGAAAATTAGCTATCTAATGAATATTCATTAGATAATAGATTGTGGATCTGTTCTTAGCTCGTGCAATCAATAAAAGCGGTCCTTCGCCTTAGTAAGCTAGCTTTCTAAGCCCGGGCGTGGATCGATCGTGACGAAAATGGGACTAATCCTCTGTACTGTAATAGAAGAGTTTGAAACAAAACTAAGGGAAGTAGCTACGATCGCTCGCAGGCCGTTGCTCGCTCCCTCCAAGTGTTGAACAGAGATAGCTACGATAGAACACAGCTAACAACCCATGACAGAATAATATGTATATAAGAAGACGGCTGCTTAGAGGAGTGATCTGTTCATCTAACTCAAAATATTGTAATGTAAGAAGAAGAAGAATCTTACGCCCAAAATTCCCATCTCTTTTTTCTTGGTTGGACCAACCGGCACCAGTCATTTCCGTCTTCCTTAATTGGGAGAGTCAGAATCAGTCTCTCTTTGTTTGGGGGGGGAGCGGAGCAGTCAATGAAGGAACCTTTGCTTTGAAAATGATTGTTCTAAAATGGTTATTCCTCACAATTTCTCCTTGTGATGCAGCGGAACCATGGCAATTAGGATCTCAAGACGCAGCTACACCTATAATGCAAGGAATAATAGACTTACATCACGATATCTTTTTCTTCCTCATTCTGATTTTGGTTTTCGTATTATGGATCTTGGTTCGCGCTTTATGGCATTTCCACTATAAAGAAAATGCAATCCCGCAAAGGATTGTTCATGGAACTACTATCGAGATTCTTCGGACCATCTTTCCTAGTCTCATCTCGATGTTCATTGCTATACCATCATTTGCTCTCTTATACTCAATGGACGAGGTAGTAGTAGATCCAGCCATTACTATCAAAGCTATTGGACATCAATGGTATTGGACTTATGAGTATTCTGACTATAACAGTTCCGATGAGCAGTCACTCACTTTTGACAGTTATATGATTCCAGAAGAAGATCTAGAATTGGGTCAATCACGTTTATTAGAAGTGGACAATAGAGTGGTTGTACCAGCCAAAACTCATCTACGTATTATTGTAACATCTGCTGATGTACCTCATAGTTGGGCTGTACCTTCCTCAGGTGTCAAATGTGATGCTGTACCTGGTCGTTTAAATCAAATCTCTATTTTGGTACAACGAGAAGGAGTTTACTATGGTCAGTGCAGTGAGATTTGTGGAACTAATCATGCCTTTACGCGTGCGCCCGGAAACATAGGCCGACTGTTGAGCCCACTCTGGCTCAGCCGCACCACCCGGGGGTGCGAGCCACCCGAGAAGCAAGCTATTACAGCGAGCGGCTGGAGCTGTAGGGAGCCGAGGAGCAAGGCAGTAGATAAGATAGAAGAAGGGGCCAGGCACCCGGTGGAGCAGAGGGGACGGTTAGGATAACGAACTTGAAACGCGGAGCCCGAGCGGCTGGCGAGCGAGTGGTTAGTGGCCAATAGCGCCCTAGTTGATGGCATTCCTCTCTGCGGCTGGCACTCGAGGAACCACAGGGCACTCCATACAGAGCAAGCAAGTCTTAGGGATGAGACGCCCGCGCAAGGACCTCAATTCTCATTAGGAGGTCGAACCAAGGACCTATGGAAGTCGGGGCTACCCCGTCCCCATGGGCAACGCAATAGTGTCCTGAGGGAGGAGTTTAGAGGCCTTATAGTAGCATGGACTTCTTTATCTTTCTAGGTCATGCGAAGGGGGCCAGTCCAAGATCGTACTGTTCCTCTACAAAGATAATAGACGCTCTCAACGGCTAGGCGCCACTCTCTTTCTGAGTTATTCCAGCTTCTTCATGATTTCGTGCCGCGGTGAACAAACAAAAAAAGAAGGCCGTCTCAGCGGAAGGAGAAGGACCTGCAACGGCAGAGACTACTGACCCTCTTCTTGTTCTTAGCCGTCTATTACGAGTCCGGGAAGCCTGGAATCATAAATATGAGGGATCCAGAAGGGTGGGCAGGGCGTTAGCAAGGTTTTTTATCCCCTCTTCCCGGTCAAGATAGATGGGAAAGGAGTCCTATCAAGTAAAGGCCATAACCAGCCTCTTTTTTTATGTACACCTTTCTTTGTTTCAGGCTCTTCAAGACCTTCCTCCTGCTAATCCGGCCATTTCCGAACCTGTCTTTCCCACCCTTCTCAATTCTTGCGATTCCTAGCCAGCCCCCTTAGCTTATTTTGCTTTATAAAACCACTTTTCCCTTTTTTCAGCTTGCTGCTCGCTTTCTCGCTTCCGAGAGGTGCTTTAGCAACTCGACTGAAAGGAGAGGGCCGAAGGCGCCTGACTTACGGTTTCAAAGCCTGGCGCGAAGCGAAGGGATTGGATTTCACCTATGATCAGATTAGTGGGCAACCATTGTTGACTTTTTTCGTGGTGTTGTTGACGTTGTTGAAAGCTAATTTCGAAGTAGGCCTTGCTTTTCTCCGCTGGGAGCAGCTCACACTATGGTGGAGGAGGTGCCGTGAAGATCTAGGAGTGTGAGCAGTACGAGCTGAAAGGCTCCCATACTGTTTGGAGGGCAGGGGGCATAGATGCCAAAGAAAGCTGACCCCTATCTATCGTCGTAGAAGCTGTTCCTAGGAAAGATTATGGTTCTCGGGTATCCAATCAATTAATCCCCCAAACCGGGGAAGCTTAAGCGGAAATTGAAGAGTAAGGTGAGGGAGGGGGAAGAGCTATCAAACTTTAGAGGCTTAACTCGCTCGCTCTAACGCTCGTTTAGTAGACAGCTCGTCAGTCAAGTACGTAACGAGCCTTGTCTACGAAGCTCCGCTCCCTCGTCTTGCTTGCTTCTGGCGAAGCTTCTAGCACTAGAAAAAATAGGCTTGTATGGCAGGAATACCACTTTTGAGGCCGATCACTACATAGGAGCGATAGCGAAGCCAAGCCGTATAAAGGCGAGCAGCCCTTATAGCAATAGCAAACGGCCTACTTATAGCCTTTTCCCCTTTATTCGGGGACTTCAACGGGTCTTCCAAGCTCATAAACTGTAAATTCTTCACGTTTTCTTCAGACAGTGGGAATGAATTCTATTACTTGATTGACATTGACAGATATGTGTATCACACCGAACAAGCAATATGCCGATATGCTTGATGTACCAGCCAAGCCAGCTCGACCGTATACAGTATAAGGGATTCGCCTTTGCCTCAGACAGAAGAAGAACAGAAGTACTACCGGGAAGGGAAGCCTGACATGGGTAGATATTTATTTGAACAAAGGAACTCAAACCGGTACCAGAAAGCAAAGAAGAGATGGAATTCCTGATTGAACATATGACCGACCTACAAGAAGACGAAAATCAAATTCCTAATTCCATTCTCTGAACTAAAGGGATGTCTCTAAGCAGCCAAGGCCAAGAGCAAGCAGGAGTGGTCCTATCCGCCCATTCATTTTTAAGTGACTTATAAGACGTGAGAGATACTCTAAAGTCATAACGGGGAAGGCCAGAGACTTCGTTCAAATGGGGGGAGGTTTTCTCTTCAATAAAATGAAAGGCAGGTATTTTTATACGAAAATTGAGAATTCAATAATAAATGTTCACTTGAGATTAGGTTCGCGAAGAAGAAGATCACAAATGAGATCTTGAGCTTGAAGCTTACTCTCCAGAATCGAAAGATCTCTTATCCCCGAGGCGAGGATGGCTTCATAAACCTTTTTTTTCCGAAGGAAAGATAGATAAGGGGTTTCATCCATATTTTTCTCCCATTTACGTATCACGTTCAGAGATTTTCATTCGTTGTCCGCTCTTTCCGTTTTTTTTTCGTACAAAACAAAAGTAATTAGAAAAAAATAATCTCAAAAAAAGGTCTGACTCTTAGAGGTCTCGATTTTTATTTTATACATGGCGGTCGGTAACGTTGGTGAATTAGGTAAAGGTACGGAAAGAGAGGGATTCGAACCCCCGGTAAACAAAAGCCTACATAGCAGTTCCAATGCTACGCCTTAAACCACTCGGCCATCTCTCCTACATTATGACCCAGAAACCTCGAGTGAATAGCGAGTCATTTATATTCTTGCAGTAAAGGTAGTTCTATATTTTTTTTCTTTCTTTATCAGAGGGGGCCCCTTAGGCCTTGGGGGGCGGGGCCTCTTTTTTGATTTTTTAATAAAAAAGGGGGAGAGGGACCCTTTTTGATTTTTGAAGTACAGCCCTACCCTATAGTGGAGTTATCTTTTCCCTATCTAAGCTATATCAACATAGCAAACTAGAAAACTTATCCGCTTGTCAATTCTTGGTGTAATATGTAAATGATTGGTGTCAAAATTTTTCACTGATCAGGTGTGATCAGTCTCATCCGTGTAAGAATTAGGAATTCCTCTTCCAACTCGTCCCAGAATGGGCGTTATGGCAAAGAACAAGAAGAAAACCAAAGGAGAAATTTGTCCAATAGTAACAAATGGTGCTTCCACAGGTTGACATCCGATCCAACCTAGTAGTAAGCAATCCGCCAAAAGCAACCAAAACATTCCTTGGTGAATCGGTCGAAAACTTGAACTACGCACATACATACTTTTAAAAAAAGGTAAAGCCAAGAGACATATAAAAACTGGTGCTATTGCGGCTACACCTCCCGCTTTGTCAGGTATACTACGAAGAATGGCATGGATCGGTAGGAAATACCATTCCGGCACAATATGAGGCGGGGTGGACATCGGATTAGCAGGTATATAATTGTCGGGATGTCCCAAAACATTAGGAGCATAAAAAATCCAAATAGAAAAAAAGATAGCAAAAGCTACCCAACCAACTAGATCCTTGACATAAAAATAAGGGTAAAAAGCTATTTTATCCATCTCAGAATGTACACCCAATGGATTATTTGATCCATATTGATGCAATGCGGCCAGATGAAGAAGACTGGCGCCTACTAAAATAAAGGGGAGTAAATGATGAAGACTAAAAAAACGATTTAAGGTGGCATTGTCCACGGAGAAACCACCCCAAAGCCAAGTCACTATGGTATCTCCTACTACAGGTATGGCGCTAGCTAAGCTTGTAATTACTGTAGCTCCCCAAAAGCTCATCTGACCCCAAGGTAGTACATATCCTATAAAAGCTGTCACAATCATTAATAGGAAGATTACAACTCCAAGACACCAAACAAATTCCCTAGGACTGCTATAACTCGCATGATATAGACCACGAAAAATATGAAGGTAAACCACAATAAGAAACATACTTGCCCCATTAGCATGCATATAACGGAGCAACCAGCCCCCTTCAACATCTCTCATAATGTGTTCTACGCTGTTGAAAGCTAAATCCACATGAGGTGTGTAATGCATAGCTAAAAAAACGCCAGTCACTATCTGAATGACTAAACAAATACCAGCTAACGGACCGAACCCCCACCAATAACTAAGATTGCTCGGGGTTGGATAATCTACTAAATGCTGATTAAGTGTGGAGGATATAGGTTGTTTAAGAAGAGAGAATCGTTGGTTCCTTATAGTCATTTCTCTTTCCTATCGTGACAACTCTTGTTCACCCACTTTTTTTTGCGTTCTAGGGCCCTAAAATATCCTAAAATATATATATTGATATTTGAGCCTTTCTTTGACTTTTGAAAGCATTCCTCGGGGGCGAATTAAGCGTCGACGTTTTTAGAATTCTTTCTCCTACACACCTTTGCCCTCTTTCACCGAAGAGGAAAGAAGAATCTTTCAAGCGGGCAGAGACCTAAATTTCTTAGATTCATTCCTAAGCTTGCTTTGTCGCAGCAAGATGGATTGGATGATCAGTCCGAGAGTGCTGTAGAGAAGAGAAAGCGGTCAAAACTTCTCTGATTAGGTCACCGAGCAGTCGGACAACCCTTCAGTAACCCAGGATGACCCCGAGAGAAGATCTCGTCCACCAAGCGGGACAGCGGAGTGCGATCCTTAAGCAATTGGAGGTTCTCGCTCATCTCTTGGGATCCATATCATCTGAATACTTTTCCTGTTACATTAGCATAGCTAAATTTGAATAGGATGACTCAGCGTCAGGAAAAGTAAGCCCCCCTTTGCCTTGATTTAATTTGGCTTCGCTGCGCTCTGAATCAATCAATAAGCTTATTGATTGGATTCATCCTATTTCATTTGGGCGAGAGGTCCGAAGGAACAAAGAAGCTCGACTGTAAGGAGAGGTGCTTTAGCAACTCGACTGAAAAGGCGTTCCTCGAGAGTGAAACGAGAGGGAGGCTGGGCTTATCCATGGGACTTGCCTTGGCGGTTAGACTTTATTTTAACACATCCTCGAATTCGTCTCGTCGGTTGGTTGATTCTCGAAATAACCTATTGAGAAAAAAAAAGGTCCATCAGGTTTTGCCCTGCCATCTCCGGCGAGGCCCCATATCCGTGAGACTGGATCACTGTAATTCACGGAAGTCCATTTGGACCTCTCCTTTTAGTCGAGTCACTCCGTGCCTCTCGGGAGTAGGGCTTTGTTCTGGGGGGGCCGACTTGCGCTGCTTTATAAGGGAGAGAATTTCATAAAGTAACTCTCTCTATCTATCCTTAGAAGTAGGGCGATAGAAAGTCAATATGAGATTTGCGTTGGTTTTTCCAACGAAACAAAGCATTATCATTCGGAAGGCTCAGTCCCAACTCGGACTTCCATAATGGGTTCATCCCTCCGCACTACGGCGCTCCAATGAACAAGAGTTCTCCGCCTTAGTATATTTAGAAGAGTAGTCGGGAGTTACATTCGTAACTTAATGTTATGTTCACGCATTATATCTTTCTTTCCAAGAGTACTACCTGTACGTAGTCTATGTCTGGGGAGCATACTTGACAGGAAATAGACACAAGCGGTAGAGAGGTCCCCCACTTCGATTCCCGCCCCGTTCGCATCTCCGATCCAAGATAAGGGATTACTCTGTTAGGTCTTTTCGGTCCGGAGCCGGCTGGTAATGGACTTACTTACCTTGCTTGTGGACCAGCTGCGGAGCTAACCTTTCTTTGTTCTATTGGTTTGGTGGTTGCTACCAAGACGATTTCTTTATGCCCATCAAAGAACCTCGAGATGCTAATCCACGAAAAACGATACGATAAATTCGAAAGAACTCAGATACAGAACGAGGGCGACCCGTGGAAATACATCGGTTTCTTACTCGTGCAAAGGAACTATTTCTTGGCAACTTGGACAACTTATAACGATGTTTGTCCCGCATATCACTAGGAAGATCGGGATCTTTACAAAAGGCTTTATAAAGCTTTCGTCTCAATTCAAATTTAGCCGCGAGCAATCTACGTTTGTGATCTCTACTATTTTGCTTCTCCGACATCTTACTGAGTTTCCCCCTCATCTTTTTGCAAAAAGCCGCTCCACAGTGGTAAAGTCTCATCTTGTGTGTTGGCCGAAGTGACAATAGTCACATTGAACCCTCTAATATGTTCGAAGATCTCGAAATGATCTTCCAGTTCCGGGGAGAATTCGCAAAACTCCGTTTCCATCGAGAATTGAATGGAGTTTTTCCGTATTTCGACCGGAAAATCTAACAGAGACATTACTGTCGAGATTCTGACCGAAAAATTAGACATTCCATGCCCTCGGAGAGTGCTTTGTCGTGCTAGGTCACTTACATATCCTTTGTCTTTATTTGACCCCAAGAATGGATTAGATCGAAAGGACTTTCCTGTCGAACCCCTTTGTGTCTGTATGAATTTCTGACCGCGCGGAATCTCCATAGCCAATTTTCCATTTTTTATTATGAAATTATAGGGTGCCTTTGGTACTACTCTTATTTCACACGATCCAGGAACTTCCATAACGTTGGCGTGATTCGGTTTGAGCAACGGATCTTGACGTGATACATCTTCGTAATGAAAATTGAGTGGAAACATGAGTTGGCTTTTACAGTATCTATAGAATAAGCTGACTCCTCCTACTCCTCCTTTCCGAAAAGATCATCCTTGGTCCTTTTGACATAAGATTCTCGGCCCGCTTTCTCCCCATTAACCAATTACGTTACGACCACTGAACAAACTTGGTTGACGAACATGGTTTATGAGCCGCTAATGTAGCGGCTTGTCGAGCATTTGCCAAACTCACACCATCCATTTCAAATGGGATTTGTCCCGTGGACACACGAGCAATCCAACCCGTAGGATTTCCTTTTCCTCTTCCCATTCTTACTTCTGTAGGTTTCCCGGTAATAGGGAGATCCGCGAAAACTCTTACCCATATCTTACCATTTCTTCGGAATTGTCCGCTCATAGCACGATGGAAGTGTCCGATTATAGCCCGACGCGCTGCTTCAATGGCTCGATATGAAAGACGACCAGCTTTACAACTTTTAGTGCCATATCTTCCAAAACCCAGTTTTGTACCATCCGGTTTGCAACCCCTACTACATCTGCCTTTACGATATTTACTATATTTCGTACGTTTCGGATATAGCACGTCCCCCTTTTTTTTGACTATATGAAATCCACACTTTGACACCTGAGATTCCGTAACGAGTAGATACTTCCGCAGGAGCATAATCGATTTTCTGGTTAAATACATTACGAGAAGTTTTTCTATGCTTATAGCATTTAGTTTGAGCTTTTTCTGCTGCGTCTTTTAATCGACCGGAAAAACATATACGGATTCCCTCCACCCTTTTTGGAATCTCCTTCACTATTTTAGCAAAAATGGAATGAAATGATCTTCTTTTGTTCTTCAGTTGAAAAGAGATGTCTTGAGCAATCAGAGAAGCGCTTTGATAAACAGATTTTATTTTGACTGACTCAATTAAGGTCTTAGTGTTTGTTCTATTAGACAAGAAAGATCGCATTTTTTTTACTTCGTAAAAATAAGAGTTGTACCTATACGGAATTCCTCTCTTTCTCAGTATGATTTCTATCATCATATCTATTACCTTTATCAATTCTTCTATCCCACCTAGGTTTTTGAATTTCTCTATCAATTCCATTACCTTATCCTTACCCAAAAGGTTCCAACATTTGACCCTTAATTGATTGAGTAGTTGTTCCCGGGCATCAAGGTTATTATACACCCCAACCCCATCTCTTAGAAAGAAAAAGGTAGCACCGAAGAATGGAAAGAGCGAGATGGTGGTTTTAGTTGTTCCCGCGAAGCGAAGATCATTCGCCAAGCGAATGAAGTGGGTCAACCTATTTTTGGCTTCGGCCAAACACTTTTTCTCGCTGGTCGAGCTTTCTACAAAAAAAGCGATACGAGAACGTATTCTCTTCTGTAAGCTTCTTCCCTGTGCATTCGCTCTCCCCATCGTAGATGGTTCAGCCGCGCCCGGTGCCACGAAATGATTGAGAACTACGACGGGGTCGAAATTCATTTGGTTCTTTGTATTAAAAAAGTATTGCATGACCAAAAAATTCAAGGAGGGGCGCACTGCAGGGAGGGTCCGTAGATAACTCGTCGGGCCGTCGGAGCGGGACTTCTTTGGGAAAAAGAACTTGAATAACTTCGTTTTTCTGAAGGAGTCGTCATTTTCTATCAGGAACGCTATGTCATTTACAACCCCGGCGTATTTCGGATGCTTGAAGGCCCCGCTGATCCGAAGTGATTTAGAAAGATTCTTCTTTATCGATGGTGATCGGTCATGGTATCCATAGCGTTGTTTTTTTTTCGGCCACCCCCGGATTTCGTTTTGCTTCTTTCGGTCGTCGAGCCTGATCGACTCGACTCTTTTCCTTGCCCCCCGGCCTCTCACTTCGTTTCGTTCTTCTTCTGTATCGTCGCGAAGACACCCGATCGGCCCGGCTTTCCCGAATGTCGTCCACCACCGGCCCTTCTCCTTTCCGGGTCTAGTTTTTTCACGTCGTTTCAGTCGTCGTGGTCGACGGGGAAGAAAGAAATGAATGAATGTTCTTTTAGGAAAATGTAGAAGAATACACCTACCGAGACGAAAGCCAAAGGTGAGTCTCGTAGGTGGACGTATCGAACCGAAATAAGATCTCAGATTGACATCTTGATACACAAATTTACCATAATAATAAATAGAGTCAATAGTGACCCCACCGTAGAAAGAGCCGCTTCTTTTTTGCTTGATAGGGGGGCTTCCATTCACCAACAAAGACTAAAAGTGCTCTCCGAACCGTGCTGGATAGTCACCCATCACACGGCTCTCAAACCCAACCTGTGGTGGATCCCGGGAGACAAAGTCAAAGCGCTTGATCCTTTGCCCCATACTTTGAGATGCTCCTCCCCGCGCAGCGACGCTGCTCGTGAGAGGCTTAGCTTTAAGCTGCTATTGTTCGGTTCGGATAAGTCAAGTCCCTTTGATCGGTTCGCTCAGGTGGTCTTATCTTCCCTAACGTTCAGAGTCGTTTTGGTTTGAGAAAGGAGCACCGGCCGAGCGCCCTGAATGAATAAGAAATGGACAGGAGAGAGAATCAATGATTCTTATTCAACCGAGTTGAAGCTAGCAACATGTTGATTACACACCGGAGGTTAGTAAGAACTGAGGGGTTCTTCCTCCTAACCTAAGTAGGCTACCCGCGCTGCGAAGCAACTGGTACTTGATTGGGGCGGGGGGGGCGGTTTGGTTTCGTGCAAGGCCCTCGCAACAGGAAGAGGCAGTTGTCATTTGAAAGGAAAAGCCCTTTGTTGTGTATAGGGTTCTAAACCAGCGCACCCTAATTAACAAACAAGCCCTTTCGCACTTCTTAGTTAAGCCTAAACTTATTAAAAACGAAAGTGCTAACGCGTCTTTATAATATTCAAAAACCTTTCGCCTTTATTGATATAAAACAAGCTTACTTACCGGCAACAAGCACCTTTTTTTCTCAAAGTCAAGTTTCTCGCTTGTTTCTTTAGAAAGATTGCAGTCTTCGCGAAACAACTTATCCTTTTCTACGAATCTTCCCTTTATTGAGCAAAACTCTATCTATATTTCTTCCCGGGATATTTTCTATAATTTCAATTCTATCATTTGTTTGACAGCTTAAACTAGGCTTCATTTTAGATAGGTTTTCGGTCTTAATCAAAATCGGTCAGGGGTGCGTCGGAACGGTCGGTGGCCGCTTAGTCTCATCCGAGAGTCTAATATCTTTGTACTGCTTTTTTTCTTTGAAAAAAAGAAGGAAGGGGTCGATTTATAGGCTCCTTCCCTTCCACTGCATAGCTGCGCTAACAGGTACTACGAGCCCTCTGTCCCACACATCTAACCAGCTCGCGTGGTTCACCGGTTCCACCGAAAACTCTCATTTGTTAAAACGGAGCATAGTGCGCTTTAGGCGCCGAGCGAGAAACCTCTCTTCTTTCGTTTCGGTTTATTCACTATCTGAAACTTAGCACTTGTTTGATTTTTATTATTGGGCGGCGGCGTTCTTTTTTGAAGTCTATCCGAACCGAATTAGCACTTCTCAGATCAGACTAGGCCTCCCCCGCAGAGCTGGGCGCCGGGGACCTGGATGCGCTAGCGATCGGCGCATAGGGGGGGTTGGGTTGCCTGGGTTTCTCGGCCTGGTATCCTACACCTCGCGTTAATGATATCTACATTCAACTGTGCCCCGGAGACACGGTCGAAGCACGCCCATCCAGTGTGCTTCTTTCACGACATGCTCTGGTTCCGGGTGTTTTCCAGTGGTCTTCTAGCGTTAGAAGAAGTCGTGTCCGTCCCGGACATCTGCAACGTCCTCCCACGCTTTTTTTGAAAATATAGGATAAACTGAAGGAAAAGACTTACCCATTCGGTGACTTTCGCGGTCGCCCTCACTGAACCGACTTGAATCTGAACTACGATTTTTTCCAAGTCTTACCGAAATCGGATTTCCTTTTCGTGCCATATTTTTTGACTTTATGGATTTCTGTCCCTTTTTTCTTCCCGGTCCAATATTTGTTCTCGAAAGTCTGAGTTTCCGTGTACTCTCCAAATTTATGACCAACCTTCCCCTCAGTGATCTTAGAACGCTACTACGCATCTTTACTATATAGTGGTAAGGTAGGTTTGGGTATAGCAGGACTTGAACCTGCGACCATTAGGTTAAAAGCCCAATGCTCTACCAACTGAGCTATACACCCAAATAAAGATGTAGTAGTCAATTAAGATTGGTGCGGAAAAGAGAAGAGGTCTCAACGAGCCTTCAGCATCTGAAACGGGAGCTTTAACCTGCATATCATACGGGAGTCTTAGTGGAGAGCGTTCTCTGGTTCTCATCCCTAGTAAAATTTGAAGTGACGGAGGAACCCCTATTCGATAAAGAAGGGAACGGTGCAAATAAAGCTATTTCGAGTCCTCTATCAACAACCAACCAAACCACCCGCTTGTCGGGCTTAAAGCGGAACTGAGCTTCTCACTAACAAAATCAATCGTAAGCTTCATGTCCGAGACTCGGAAAGAGTGGCGTTAAAGAGGGCGTCAGAGCCTCTCGAAGTCTCCGATCTCACAGATGGTTAGGCTGCAATTCCTTCGCTTGTGAAGAAGCTTGGCTAGTTCTCCTTACTCGGACATTCTATTCATTCGACCACCGCCCCCTCCTTACGAATGGTTACGGGACTAGAGCGAGTCTCTTTTTATTTGCAAGAATAGGTCTGAGCCTGATGACATTCCTGCTTTCCTTGCCATGTCCAACTAAAGTGAAAGTGAATCAACTGCAAGGAGGAATCGACCTTTTTCTACTATTCATTTGCGCCGGAAACCCCTTCATCATTTGCCCTGAACTGGTGAAAGGAATAGGAGGACTTTTCCCTCGGTGGAAGTAGGGATTTAAGCACAGTTGTGATTCCGCTTTCATGTCTTGGATTGAGCTTTCTCACTCCGAGAAACGCCTCTTCCTTGTCGGCGTCCTTTGTTCGCTACTGCTTTAAAGAATGGGATGAGGCTACCTGATCGTCGAGTCGACTTCCATCAATCAATTGGATTGGATCTTATTATCCATGGTCATAAATCTGATTCTTCACCCAGTGGGAACACAGTCTTCATGGTGGTACCAGTGCGTATATATGTAGATGGGGCCTTCGGCTCCCACCCTCGATTTTCCATCCAATCTTCCTATCTTCTTTTCCCTGCTTTGGAAGCATTCGATCTCTGCTTCGCCCTCGTCAGAGAAAGGGGGCGAGAAGCCTGCGTGCGATTCCTGGGTTGGATATCCCTGGCTCTCTTCTCTCTGAGTCCCCGCTAGCTTTCAAGCGTTCATTCAATCTCCCTCCCAACGCAAGGAAACCGATCGATGCACTTGGCTTTAGGTTTCGATCGATAATAATTTATATTATATATAAAACGCTTCTCGCTGGAGACTCGGGTAGGGCGCTTGCTCTCCTCTTGGCAGCCTTCTTCAAGTAGGCTTCTTTCTTCGCTAACGCTTAGGAATTCCTAGAGACTGAGAAACTAAGTTGAATTCTATTTCTAGCTTTTTTTTATAGCTATATATTTCGTAACTGATGAGAGATTAATTGATCGATACATCAGATCCTAGGTCGGGAATGACGGGGCTCGAACCCGCAGCTTCCGCCTTGACAGGGCGGTGCTCTGACCGATTGAACTACAATCCCGGCTATACTGAACACTAACATGATATACTGAACACTAACATACTCGAGCACTCGTTGCGAGAGGAACCCCAGTTACTCGACTGAAAAGGAGAGGTTGTGAACACAAACTCGACTGAAAGGCGTTCATCGGCTTTCACCCTTCCATTGCACTTTCATGAGTCAACCATCACTCATACGAAATTGAGATTGCCGAGTCAACCTTCTTCTCATACGAAATTGAGATTGCCGAGTCAACCTTCTTCTTTCAGGCTTTGAATGGTCTCCTGATGGAAAAGGTCTTGTTGGTTTCAATTGTTTATTGAAGTAATCCTTGCATTCAGCTGCTGATTGATTCCAGTCAGTGCTTCAGATTGTCTTGGGGCACTGTCAATATACTGACTTACAGGTAAAGTCTCCGTTTCCCTATCTAACTCCTCTTGGAATCTCTTGTTTTCAACAAGGAAAATTCCATTTTCTGCTTGAAGCAGTGGCTAACTCATACAAGACTTCTGCCCGAACGTACTTTTCGTTTTCAAATCCAAGAAGGGACAAGAATGGAATGATTTTCCTTTTGTCAATATCTCGTCCGATACTGGTCCTATCGGCCTCTTTGAGATTAAAATCGCCTTGACTTCTGCCAATAAGAAAAGCAAATCAAAGCTATGGTTGAGAGTTCCAAGTTCTTTCCAGATTAGATTCTAGCTAGGATTTGTTGGCTCAATCAGTCGTAGAATTCCTTCTCCCCCCGGCATCTACTCCTAAGATCTCTGACCTGATCGATGTCTCAGGAATTAGTCCCGGATCATATTCCAGTGCGTCCACAGAAGAGGAAATCGCAATGCATCTTGCTCAGCAGGCTTAGCTTGGAGTGGGACAGGGGTTCCTCACTCCGTGCCTCTCACATTGGGAACTTCGCTCACTTCCTAAAACACTGGAGACTGAGGCGCATTCGCAACAATACATCTATGACTAGAGAATTAGGTATAGGAAGAATAGATCTAGCAATACAATCACCAAACTATTAACACTTCCAATACCCATACCATCAACTCAAAGGTTGTCACCACGGAGCATAACTGCAACTAAAACTATTACCAGAAAGACAACTATACTGGCATGCCACCTATACGGGCACGCGTACTCTTTCCAAAGCTAAAAGCACCTCCTACACTTGAGAACTACTACGCATTGGAAACGAAGCAGATAGTCACCATTACCATAAGACCAGCGACCAACTAATGGTTCTTTTCTTACTTAAGACTCAGAAAAAGAAGAAGCCAACCCAGATCCTTATTCGCGGTAGCAGCTACTTCTTCTGCTTCTTCGGTTGTTGCCGCCTAATTAGCTACGATCAATGAAAATCTCTACAGAGAAGAAAGCTGGTCCCACTAATTTACCTAAGCAGGTCTCCTTAAGTTAAGTAAGTGCATGTCCTTTCTTTTGAAGGTTGGGGAACCTACCGATGAAGATTCTCTCTACCATACTGGGCAATATAGATGAACAAGCCATGCCCACCCCGCCTCAGATCAGCAGGAGTCAGACACTGCATGGCTCATCCATCAGGTAAAGAAAGGCCAACAATAGAATACATGTTATTTATGGCTGTTTGTTGCAGGAAGAGTTTCATCAAGAGAACAAGGCAATAAGGTATCTGCAGAAATAGGAGTAGTAGCAGTAGTCGCGGGTGTCGAAAGAATAAGGGAATCAATGCTGTCAATCGGAACAACTGGTTGGGCTACTCCTTGTTTGAGGAGTTAGTTTCTTGATTCTTTATCTCCGGTATCTGCTCTAGAAAGAGTTTCAGTGCAAACTCTTCTCATACTATCAAGCCGGAATAACTCGACTGTAAGGAGAGGTTTAAATAACTCGTGGAACCTTCTACTCTCCTTTATCAGTATCATTTCGTTGGACCTCTTCTTTTTAAGAGATAGGGGCTACATTTTCTATTCCCGCCGCTTTTGTTGTATCGCTAAATAAGCAGGCTTCCGTCAAGCGAGGTCAGCCAAAGGGAAAGAAGAAAGAGTGAGCGAGAAAGCGGTGAGCTTACTCTTTAAGAATTGATTACCGTTGATGGCGGGGAGCGGAAACCTTTCCCTTTCTAGTTGTGAATTCCGTATTTTTGAAAAAAAAAAGTGCATAATATTGGATTCAAACCGACGGCCCACCCTTTCTTTGAACCTTGTCAATGATCGAACTTAAAGATATGAACTGAGTGCCATTTGATGAGTAACTGAGAACAAAGGAGAGGGGTATAGCAAGGATGAAGTAATGAAAAAGGAAGTCATTGCTAGTAGTAACGACTTATTACGATCCATTGGTTCATATAGAATCCATGTCCTAGGTGTATCAAAAAACATTCTTTTCACTAAGCGTATATAATAAAATAGAGTGAAAGGGTCCACCCCGAAACCAAGGGGATACTAACTAACAGCTGAGTCCTCTCCAAACCGCAGGAGATAGTTGCCCATCATACGGCTCACCAACTTGCCTCTATGGGAGGCTCACTCCGGGCAGGTTCGGATCACTTATAATACAAAGCTCGGAGAAGGAGGGAGTTGGGTTAGGAACGCAGTAACTCGACCCCTCATCAACTAATTAATGAGACCTTATCCTTGGTGGGAGAGGGCCGAAGGCACTCGACTAAAAGGTTCAAAGATCTCGACTGAAAGGTTCAAAGATCTCGAGCGTAGCGAGAGGTGCTTTAGCAACTCGACTGAAAAGGCGTTCCTCGGAGTCATGACTCGAGCACTTGTCTAGAGAGGCACGGAGTGACAAAGGAGCTCGACTGTAAGGCGTTCCTCGAGAGTGAAACGAGAGGAGAGGTGAGGTTTCAGTTTTCAATATGATTCTTTTTTCGTATTTGTTCGATGAGAAATGCGAGTCAGTTTTGTCCATTTTTTCTATCCCCCTCTATCAAACAAAATGATCAAAAAGGAAGAAGTTTACTGGCTTCTTATTCTCGTCTTTGATCTCTTCCATCTCTGCCTCGCTCGTTGTCACCTATATATATATTGAAGAAAGAAACCCTGTAGAGAATGAAGAGGGGCCTAGGATCTTCTTCTCAACAGTGCTTCTCGGGGCTCCCCCCTGAATAAGTAAGGCCCCGTTAGCCTGGGCGAAGATGGGGATAAGGAATAAGGATTGAAGCCCCTTAGCTCTGCCAGGCACTGGACAGGGGTTAGCTCGGTAAATGTGTAGAGCCAAGTGTAGTATGGTGTAGTAGTAGGCACTTCTAGGCCCCTTCCCTATACTGGATAACTCCAGTGCTTTGGGTACTACGGACCCTCTGCCATCCATTGCAGCAGAGCCGTTTCATGAGCGGGGGGGGCTAAGCGCAGTTCTTTGAATCAAACGTTGAATGAAATCGATTCTTTTTTAGATATCAAAATGGAAATCGGATAGGATAGATGGATGGATCTATCTTTCCATTTATATATTACTAAAGGATTTATATAGTTAAGTAGCGTAGCAAGAAGCCCCAAATCCTTGATTTGGCCAGGAAAGACTGCACTGCTTTGGGCCCAGGATGCGAAGGGAATGAGCTCGGCTGCTTCTCCTCCACACTGATTTTTCTCCGTGCCTGCTCCGCATGCGCTTCGCGCGCCATTGGCGCTTTGCTCTCCTCTTATTCTTCATTGGACGGTTCGGTTCGGATGGACTTCGCCGTTCTTTCCCAACTAAAAAAGAAAAGGCTGTATCACATCGAGATGTCGATTCGTTTTCCGCCCCCAATGAGATGGGGAATTTGTAACCCCCTATTTATACTTTGGGGCCCTTCATCTTTCTGAATCCAGGCCCGTCCCGGCTCGCGTCGTTCCAACAACCGGCGGGGAGCACCTCAGTATACGATCGCGCGCAGTAACTGGGAGTCCTATTACACCTAAGGCGAACTTCAATTCACCAAACCAAGGTTCATCTCGTGTAGTGATTGTGGACTCTACTAAGGATATTGAGTAGACGGTTGATGTATCAGACTCGACCCTATCTTTCGTAGCATGCATTCCCATCGGTGTCGCAACTGATTCGGTAAGCTACGTGTCCGGTGCACGGAGAACTGCCTTCGGTCCTCCAAACTGACTTATTCGTGGCAACCTTCCGGCCGCCCAACGACCTATAACGCTAGTCACTACTCCCACTGGGGCTAGGAAGTAAGCCCCACAACCCAAAGCGGCGAAGAACAAATAGAATTTGCTACAAAAGCCGGCTAACGGGGGTATTCCTGCGTATGAGAACATAGTAATGGAGAAGGTAATAGCCGAAATAGGATTCGTTTTGGCTAGAGCGCCCAAATCCGCTATATATTTGACACGGGTTTGCCGTAATGCTGAAACTATGGCGAATGCATCCATCGTCATTAATGCATAAATAAAGATACCAATTAGTAGTGATTGAATTCCTTCTATGGTTCCACATGAGAAACCAGTACGAATATAACCTACATGTCCAATTGAACTATGAGCTAGAGGTCTTTTGACTTTCGTTTGGGCCATGGCGGCCAGTGCTCCTAAGATCATAGAAGCAATGCTGCAGAAAAAGAAGATTTGTTGCAATGTAGCTCCATAGGAACCATAAATAGAAACACGTAAAATATTAGCAGAAATAGAGATTTTAGGCGCAATAGAAAGGAATGCTGTAACCGGGGTGGGTGAACCCTCATAGATATCTGGTGCCCACATATATAGAGTCAAAAGGAATATGGAGTCCGAGGGGGAGGGGGTTTTCTTCGGGGCTCGAAAGATTGAATAGACCCACAAGTTTGAAATTCGCCACTGGGGAATTCACACGAAAGGGAACGAGGAATTCTCAACGAAAAAAGTGCTCTCTGAACCGAACGTGAAAGTTTTTTTCCATCAGACGGCTGTTCCTGTAACTCCACTCTCGACTTGGATTATATATCCAAAAAGGTCCGCTCTCGGCCATTCCACACGCTGCCTAGCCGAGGTGTGGTTATCTGAAGTGGATTCTCTCTTTTCTAGTAGATGCCGAACCTGCTGCCCCATTGACTAAGAAGAGGGGCGGGCGCAGCAGCTACATGGACCCCTTCCTTTCTGTTGTTGCCAGCGCTTTCCCGGGCCGAGCCGGAATTTTTGATTATATTCTAATGGGCAGGTAAAGCCCGCAGGCTGCTATCCCAATAGGCCAGCGGGCGGAACGAGGAGAGGCTCCGGCAATCATAGCACCGCGGTCGAAAAAGCGTGCTCCCTTCAGATAACACGCACCGTAGGCCATCCTCGGCCAAGAAAGAAAATCTCTCGATCTCCTAAAGCCTTTTCCTTCCCCCGCCGCATCTCCCTCCCTTCGCCGAGCCTTTCCGGGGCTCGTCGAGACCTTATCAGAACTTGGCGGGCATTCTTTCCAGCCCGGGGGATGGGTTTTGTTTGAACATAGGCTCAAACATGATTTGAAGGTCCTAGCTACGCCATGCGTTCAATACAAAATCTGGAAAGCTGCAGAGATGACAAAAAGAGGGCGCTTTCCTATGTTGCACTGAAAAAAGAAGGACCTAAGAGAAAACGCTCTTAGGTTTTTTCCTCCCGCGCCCGCCGCCGCCTGGCCCCCGTTAGAGGGGAAGGGGAAGATTAGCAAAGCGAAAAAAGACAGAGGGAGGGGGAGCAGCATCTTATTCTCTTCGCGAGAACTTCCGGATCGAAGAAGCATTCGGAACGGGCTCCGCGTTCATTTCGTTGGCGGAAACGATCCAATCCATTCGGGGCTTCGGCCAAAAACGAATTCGACTTGATTCATAGATAGAATCAATGATAAATAAACAAAAGATAGATGAACGAGATATCTTTTCTTTCTATAAAAAAAAGAGGAATAGAATAGACATCCCTTTCTTTAGATGTCTATCTATCCTTCCGATTTTATATCGTTATATCTGCTCTCTCAATTTTTTTTAAGAGAGAGCAGATAGATCCTATCCCCTATATCGAACACTAATTCCTATCTATTGATAGGAAGATCTTCGTCTAATAGCGGACTTTGCCTTTTTTTAGGAATTTATCATATCCAAGGCAGCTTACCACAAGAACCCCACCCCATACGACTAGTTGGGGGGGCTGTTCGCCTTTTGAATCAAACAAAGTAAGTTGTAGGTAGGGGCTTCATAGCTACTTTCATTCTAAAGGAAAGCGAAGAACCAATCTTTAGTCAATAGGAGCCCTACTTCCCGAGGTATTTCTTACTCGACTAAAAGGAGAGGTTGTGAACACAAACTCGACTGAAAGGAGAGGTTGTGAACACAAACTCGACTGAAAGGAGAGGTTGTGAACACAAACTCGACTGAAAGGAGAGGTTGTGAACACAAACTCGACTGAAAGGAGAGGTTGTGAACACAAACTCGACTGAAAGGAGAGGGACAAGGGCGGTCTTGCTTGGCGCGAAGGCTGCTGGTTGGGGGTACGGTACTAAAGGTCCTCGGACTTCCAGGCGGTTTTGATTTTGGGCAGCTGTTCACCGTTGGATCTCGCCAATACAGCCCCCTATGGTTTTTGTTACCGAGATATCTTTTTTTTTTCATTGTTCCCAGGGATTTTTTGGGTAATCTGCTCCCATGCTGCAAACAGTCAAATCAGAACTCAACCTTGTCGCTCTTCTTTCTTTCCTCGCGGGCAGGAAGCACACCAGCAGCGTGCGTTGCGTGATTCTACTGTGTTTTTTGCACTTGACTGGGTGGACAGTTGACCGGAAGAGAACTTCGATTCGCCCGGCCAGCAGGCGGGCGGCGTGCTTATCTTGTGTGACAACACTACAGAGCGAGTGGCTCTTCTAGGCGGGCAGCTGTATGACAACACTACAAGGTTTTTTTTCCTCGTGTAACATGCTATGGTCTCAATGCCCTTACGAGGTAGTGATGAGTTTCACTCGCTCTAAGCCCGGCCCGCGCGCGGTTAGGAAGATTGGCCGCAATCTGAGCGGTACCACCCACCCTACCCTACCACCTATAGGCGGCCGTCCGTCCTACAGCCGCCCGAAAAGGAACTGCAGTGATCTTGAATAGGAATCCTACAGCGATAGATAGAATCCCCATAAAAATACCACTAGATCGAGCACCAGTGATTTCGTATCCGGTCAAAATCTTGGCTAATTGATCGAAGTGGGTAGCTCCAGTAGACCCATAGATCATGGAACAAATAGGGTGGGTAGGCCCAACCACCACACTACACGTATAGACGCGAACCCCCCTCGTTACCGTACGTGCGACTCTCACCGCATACGGCTCGCACAAAGACTCCTAAATCCATCCCGAGCCTTTTCTTCCCACCTCTCCTTTCAGTCGAGTTACTAAACTCTCCAATCCTCGATCAAACTTGCCCAGGCGCTATTAAATAAATGGGGGTCTTTCCCTTCGCCTATCGTATATTGTATATTGTATGTATCGGCTTGGCTTCGTCCAAAACGAAAACAAGGAGGCATTCCGGCGGGCGCGTGCGTGTAGGAAGGCCGACCATTACATAAGCTAAAAGACTAGGACTACAAGCCAGCCGGAAGCGACTCGCTTCTATTCCCCGTTGGGATTGGATATAGATGGGGAATCTATTGATCGTAGTAGTTCGCCAACCTCTCTAACTAACATACGATCAAATTTTCGGCATGGCCAAAAAAAGAAAGAGCTTCGCTCGGTGGGCCTTCCTACGCTGACGAATGCCTCCTTTGTCTTCTCTTCAGTCTACAACAAGTGGGAGAGGCAGGATTCGAACCTACGTAGAAAAACTTCAACAGATTTACAGTCTGCCGCTTTTGACCACTCGGCCACTCTCCCCTTCCCGGGCCGAGGCCCCCTCAATGGGTTCTAAGAAGGAGGTTTTTTCCCTGAATCAATAAAAAAAAAGAAACTTATTGATTTGATTGAAGGGAACTAATACTATTTCTTAGCTTAGCTAGCGTTCTGGGAGAATCTAGTCATTTAGTCAATTCATAACAATCTCTGTAAAGCAAGGGGCAAAGCTTCTACGACAGCTTCCCCCTCATGTAGTCGTCGGCCTTCTTCCCCAGCCCCCTTCGTCGCTTCTGGCGAAGCTGCGAGCCTACCCTTCTCGAGCCTACTTAAATAGCTTACGCTTACCAAGCCTAGTCTACTAAAAGAGGGCTACAGTAAGCAAGCTTTCCCCCTTTGTTTTTGTTGTGGGTCGCGCCTCACCGCAGGCACTGAATGAATGAAATGAGTGGAATGGTTAACCTTCCCCCTTTTGAATTACGAAGAACTTCGTTGCCACTAGTGGCGAAGCAAATTTCAACCATCCCATCCGAAAACCACTCGGAGCCTAAAGAGAGTTCAGTCTACAAGAAGCTGGCAGAGCTTTAGCCATTGGACTACATCGCACTATCCTACCTAAACCCTTTTCTTGTTTGTTCGGATTCTTTTCTTTTTCGAATGAGGCTAGTGGAGACAAATTCCTTCCGACTAATGAAGAGATACTACTCCAGTCTTCCCATTCATTCCCAGTTGATCCTTCTTCTTCCTAAGAATTTAACGAACCAAGTTCACCTATACGAGAGTGAGGAATCAAAACCAACAATCAACTTCCCACTTTTTACACGATTCAGCTAGTTTAGAAACAAAGGAGAAGGACAGGGGCCGTTAGGTCAGAAAAGCGATAACTAACAAATCTCCCCAAGTAGGATTCGAACCTACGACCAATCAGTTAACAGCCGACCGCTCTACCACTGAGCTACTGAGGAACAACGGGAGATTAGATCTCCTAGAGTTCAATTCCCGTTCTCAACCCATGACCAATATGAACTCGAAGTTTCCTTCGTAACCCCCGGAACTTCTTCGTAGTGGCTCCGTTCCATGCCTCATTTCATAGGGAACCTCAAAGCGGCTCTATTTCATTATATTCCATCCATATCCCAATTCCATTCATTTAATATCCCTTTGGTGTCATTGACATAAGAGATGTCGTTTCTAGTCTATCTCTTTCTATTTCTATATATGGAAAGTTGCAAAATCATCATATAATAATCCAGAAATTGAAATAGAAAAGAAAAAAGGGAGGTTTGTGATGGTTTTTCAATCTTTTATACTAGGTAATCTAGTATCCTTATGCATGAAGATAATCAATTCGGTCGTTGTGGTCGGACTCTATTATGGATTTCTGACCACATTCTCCATAGGGCCCTCTTATCTCTTCCTTCTCCGAGCTCGGGTTATGGACGAAGGAGAAGAAGGAACCGAGAAGAAAGTATCAGCAACAACTGGTTTTATTGCGGGACAGCTCATGATGTTCATATCGATCTATTATGCGCCTCTGCATTTAGCATTGGGTAGACCTCATACAATAACTGTCCTAGCTCTACCGTATCTTTTGTTTCATTTCTTCTGGAACAATCACAAACACTTTTTTGATTATGGATCTACTACCAGAAATGAAATGCGTAATCTTCGCATTCAATGTGTATTCCTGAATAATTTCATTTTTCAATTATTCAACCATTTCATTTTACCAAGTTCAATGTTAGCCAGATTAGTCAACATTTATATGTTTCGATGCAACAACAAGATGTTATTTGTAACAAGTAGTTTTGTTGGTTGGTTAATTGGTCACATTTTATTCATGAAATGGGTTGGATTGGTATTAGTCTGGATACAGCAAAATAATTCTATTAGGTCTAATGTACTTATTAGATCTAATAAGTATAAGTTCCTTGTGTCAGAATTGAGAAATTCTATGGCTCGAATCTTTAGTATTCTCTTATTTATTACCTGTGTCTACTATTTAGGCAGAATACCATCACCCATTTTGACTAAGAAACTAAAAGGAGAGGGAAAGAGCTAGCCTTCGTCTCAAGGTTCCAAACAAGAGGGTAATCAGTTGTGACTACTGAACCTACTAGCTTGCAGAAGTAGCTATCTGAGTAAAAGCTGCTTTCAAAAGAAGAATAAGGAAGTCTAGCAAGCATTCAAGCAAGTCTCGCTGATCCGCATTGTATTGCAAGCTAACCAAAAGGAAAGAGAAGAGTCACCTTTTGAAGCTATCAGTTCAGCTATCAGTTCAATCATTCACTCGCTAGGAAGAGGGCGTCTCGCATAACTAGGACAGTGAAATTATTTCTTTTCGGAATCTGCAATTGGTCAGCCTTTCTCGAGGAACGCCTTTGGTCCGTCTATCTCCTACGAGGAACTACGGCCAAGCTAGTTCTTAGTGAGCTTTCTCTCTTCGGGACACAGAACTCCCGGAACTTCCTTTCTTGATTCACCCAGAACGGAACTTGCTCTAATCCTTCTCGTTCGATCCATTCTTCCTAGCGCTCCTCAATCAGATCCATAACTGCTGTTGCTTGTGCAGGAGTAACTGGAGAACTTCTAATTTCAATGCAGTTGTCAACTAAGAAACACCGGAAGTCCATGCGAGCCGGGCTGACATTAACCAATCAGATACCCTCCTCGAAAGGGCCTGAAATCAAACACCCAGCCCTATTAGATATTAGATTAGAGAAGGGATAAAGTCGAGAATGACATTCAAATTGAACAACTGTGTGGTGTGGGCTTCGCCCCTTTTGTAAGGGTTTACGAGGTGAACTCGGAAGCGTGGGCGTCAAGCATCAAGTGGAGGCGCCGAATCGTTGCAAGAGGGGGTACTAAGCGGGATCGACCGGTCAGAAGCCAAGCTGGCCAAAAGGCATAGGGGTTCCTCTGAGCTGGCAGGCTCATCTCACTGATTGTGTGCTTTTATTGGATAACTCCCATCAAGGGAAAGTGCACTTTAGCTCTATTCCGTTTCCACAACGTGTTGATTTCCCGCGTTGACCCCTAAACACTATGGTATGGCATCAAGGTCGGCAACCCAAAAGGGTCGGGCCTCAACCGATTGACTTATTTATAAGGGGAAATCCACGAATATATATATAACAAGACAAGCACTTGCATACAGAAAGACCCCTTGCGCCTAGACCGATAGTCCACGTCTTCCACGTAAGTAGTTGAGTTAACGCCCCTTTCTAGACAAGTGCTCGAGTCATGACTTCGCCCTCTCCTTTCAGTCGAGTTGCTAAAGCACCTCTCCTTTGCTGTTCGAGTAAACAAGAAATGCTCGAGTTGCTAAATACCCCTAACGGGGCCCCTCTCTGATAAGGAAAAAAACGAAAAAATCTCAAATTTATGAAAAATCTGGTTCGATGGCTGTTCTCCACAAACCACAAGGATATAGGGACTCTCTATTTCATTTTCGGTGCCATTGCTGGAGTGATGGGCACATGCTTCTCAGTACTTATTCGTATGGAATTAGCACGACCCGGCGATCAAATTCTTGGTGGGAATCATCAACTTTATAATGTTTTAATAACAGCTCATGCTTTTTTAATGATCTTTTTTATGGTTATGCCGGCGATGATAGGTGGATTTGGTAATTGGTTTGTTCCGATTCTGATAGGTGCACCTGACATGGCATTTCCACGATTAAATAATATTTCATTCTGGTTGTTGCCACCAAGTCTCTTGCTCCTATTAAGCTCAGCCTTAGTAGAAGTAGGTAGCGGCACTGGGTGGACGGTCTATCCGCCCTTAAGTGGTATTACCAGTCATTCTGGAGGAGCAGTTGATTTAGCAATTTTTAGTCTTCATCTATCTGGTGTTTCATCCATTTTAGGTTCTATCAATTTTATAACAACTATCTTCAACATGCGTGGACCTGGAATGACTATGCATAGATTACCCCTATTTGTGTGGTCCGTTCTAGTGACAGCATTCCTACTTTTATTATCACTCCCGGTACTGGCAGGGGCAATTACCATGTTATTAACCGATCGAAACTTTAATACAACCTTTTTTGATCCCGCTGGAGGGGGAGACCCAATTTTATACCAGCATCTCTTTTGGTTCTTCGGTCATCCAGAGGTGTATATTCTCATTCTGCCTGGATTCGGTATCATAAGTCATATCGTTTCTACTTTTTCGGGAAAACCGGTCTTCGGGTATCTAGGCATGGTTTATGCCATGATCAGTATTGGTGTCTTAGGATTTCTTGTTTGGGCTCATCATATGTTTACTGTGGGCTTAGACGTAGATACCCGTGCCTACTTCACCGCAGCTACCATGATCATAGCTGTCCCCACTGGAATCAAAATCTTTAGTTGGATCGCTACCATGTGGGGGGGTTCGATACAATACAAAACACCCATGTTATTTGCTGTAGGATTTATCTTTTTGTTCACCATAGGAGGACTCACTGGAATAGTCCTGGCAAATTCAGGGCTAGACATTGCTCTACATGATACTTATTATGTGGTTGCACATTTCCATTATGTACTTTCTATGGGAGCCGTTTTTGCTTTATTTGCAGGATTTTACTATTGGGTGGGTAAAATCTTTGGTCGGACATACCCTGAAACTTTAGGTCAAATCCATTTTTGGATCACTTTTTTCGGGGTTAATTTGACCTTCTTTCCTATGCATTTCTTAGGGCTTTCAGGTATGCCACGTCGTATTCCAGATTATCCAGATGCTTACGCTGGATGGAATGCCCTTTCCAGTTTTGGCTCTTATATATCCGTAGTTGGGATTTGTTGTTTCTTCGTGGTCGTAACAATCACTTTAAGCAGTGGAAATAACAAAAGATGTGCTCCAAGTCCTTGGGCTCTTGAACTTAATTCAACTACACTGGAATGGATGGTACAAAGTCCTCCTGCTTTTCATACTTTTGGAGAACTTCCAGCTATCAAGGAGACGAAAAGCTATGTGAAGTAAAAGAAGAAAAGGTCGCCGACTGCTACTAAGAACCTAACAGAACTTTTATAAATTTCTGCATTGAGATTCCGTAAGTAACTCAGTGAGTGCTTTCTAAGAAAGAAGGGCAAGGAAGAAGAAAATGAAATAGGAACAACCGCGCTGGTCGTAATAGATCGACTTTCATGCTGAAGCAAGAACTAGCATGAAAGTTCCATTTCAGGGAAGGACGACGTACCCATGATACTTTCTGTTTTGTCGAGCCCTGCTTTAGTCTTTGGTTTATTGGTTACACGTGCTATAAATTTTGTACATTCCGTTTTGTTTCCCATCCCAGTCTTTTGCAGCATAGAAACCTTTTTCACTTACTTTTCGTCATTTCCTATTATAAGGAAATTGTCAATGAAATGGCAATTCCTTTGGTTTTCTATTTTCAAATTCATTTTTTCCATTATTATGATAAAATTATTATTTTCAGTGGGTTACTTATGCTTCGACGATTTGACTCGTGCCATTTCTCAATTCTACCCGCCGATATCAGGATTTATGGGGGGGGGAAATACGCCTATGCCCCCTACAAACCCATTCGACGGTTTTCTTAGCAGCTATTTTGATAATGAGCGCTCTAACGATCAGCGGAGGGGTTCACCTTCCTGGTCGGAACAGTTGCCTGCGGAAAGTGGCCTTTACTTGAACCTCGATGTAGAAGATCAAAACAAAGATCCCATCGAGGAGCAAGTAGAAGCAGAGGGTTTAAGATGCGACAGAATAAAAGAAAAAATTATAGAAAAAACCCATTCTCTATTAATAAGCAAAGGGTACCATATTCCAGAAAAAGAAGATATACGCCGCGTTATCGAAATTGTTATGTTTCACGAGGAATCCGTTGATATTGATCATCGCAAAAGAAGATTCTACTACCTTTACTCCTGCTTAGGAAAAGAGGAAACTCCATTCTGGCGGGAGATTCTTAAATTACTTGCTGAGTATAACATCACTCTCTTCGATACCCGTTGTGAGCACTAAGCGACACTTTAGGTTTACTTCTTTTGTTAGGTCTCGACTTCTTCGCTATGATCTTTCCAGTAGTTCATATAGGAGCTATAGCCGTTTCATTCCTTTTCGTTGTTATGATGTTCCATATTCAAATAGCGGAGATTCACGAAGAAGTATTGCGCTATTTACCAGTGAGTGGGATTATTGGACTGATGTAGTAGGGGTTGCAAACCGGATGGTACAAAACTGGGTTTTGGAAGATATGGCACTAAAAGTTGTTCAGTGGTCGTAACGTAATTGGTTAATGGGGAGAAAGCGGGCCGAGAATCTTATGTCAAAAGGACCAAGGATGATCTTTTCGGAAAGGAGGAGTAGGAGGAGTCAGCTTGTGTGAAGGAACAGAAAGCGGAATGGCATATTAATTACAACCTACATACTACTGAAACTAAGAAAGAGTCAACTGTGAAATAGGGAAGGAACGGAGGAGGAAGAGAGACCGAAGGGATCGAGGGAGTTACCCTACGCAAAGGGAGGGAACGTAGCTGCAAGGCGTCCGCCCCACTAGCGGACCAACGGGCCTGGTTCGTTAGAAGCTCCTGGGCCCTAAGACAATCCGTCCCGGCCGAGCAAACTTGTTTGGTCGAGTTAGCCGGGCACCTTGATTGGGTAGTAGTGAGCCGATACCAGGAGGCGAACTATCTATTAAAGAACGTGTGTGTACGTGTGTGCGCATGTACTAATAACCAAGGAAGTAAGCCAATGAACCATAGGAAGAGGAAGACCTATAACTATCACCATAGGAAGCCAAGGAAGGATGGAAGGAAGGTTCGTAGGATGTTAAGCAACATTTGACCTACGCCACACTTTCGATTCCCCGAAAGTTCTAATCGAAGGCATTGAAGGTCGGCAAAAAGCAAGCTAATTCGGGTTATATCAAGGAAACGGCTCTCTTAAAAATTCGAAGACAGAAGGAATTTGGCTATACCACACAAGATGACAGGCAGTGAATATCCCCGAGTGCCTCTTTTCCGCGAACTCTAAATAGACTTTAAGTAAGAATGTGGAACTCTTATTCGTTACTTTTGATCAAGACGAGGAGTAGCTCCGAAAGAAAAAAGAGCCAGACTGTCGTGTACTTTAAGGTGTCCTTCGATTCGGGGGGCGGCTTTTCTTTGAATATATAAGTCAAGAGTCAAAGATCCCGATTGGACTACACGGCGGAGCAGATTCTTTTAATAGCGGTTGCTAGTCAAAATCATAAGCCGAGCACAAGCCTACCTGCGGACTAGATTCTAATTATATCAAAACCCAAGGGTTGTTCGGACTGGTACGATCTTGACTTCGACTGCGACCCCCGAAGTCAGGTATGACGCTCCTATAATCATATGATGAATGTTTTAGAGGATCTGCAGGTCGTGAAGGGGATAAGAAGGACTCTTGGTGAAAGGCTTTTTTCTGACCCTTCAAATCCTCATCAACTAACTAGTTAATGAGACTTCTCCAGAAGAGTCTTCTTTTGAATGCCTTGAGGAGCTCCTCTTTTGATCAATCGTAACAAAGCAGAAATGATTTCGTCTTGTAGGGACGACCAACTTGGAATTGGAATGCAATGTTGGCATTTACATTCCCTTTTTCAACTTCAGGGCTTTAGCGTATCAGTTGAGTTCCCTCCGGATTGGAGTCCTCAGACAACATCCCTTCCCGGCTAATGCAGCTTGTAGCTCCAGTTTCAAGATTCTCTCCTCTCCCTGTTCATAGGTATCATGAATGTTATATGGACCCCGCCTCCCTAAAGGGACTAGGTTTACCTTACCCGGCTATAACCATTGAACCTGTCTGTATCAGGCTTAAGGGTAGAGGAATTGGTCGAGGTGATAGGCACATTTGCTGAAGTGCCGGTTCTTCCAAGATTCCTTTGCATTTCTCGTGGACGAACTACTTTCTTTTAGTTTTCGCTTTGACTTCACACTTCTCCATATTTAGTTTATACTCAAATATTAGGTAGGCTCTCTCTCTTGATTAAGGGCTGATAGTTGGTTCATTAGGAAATCTTTCTAAAAGACGGATATCCTTCTATCAAGGAGAGATTGATATATGAAATGATTAGAATTAGAATGATTCCAGTTACTCACAGCGGTGCTACGATCAGCAGGTCTTGTCTCTCCCCTCGCATCTGTGTCAACTGTCCGTTGCTGTATCAGTAGCGGAAAGCTTTACATTGAACCAGAGGAATTCCGCCTCCTCCGACTCAAACATTGGATTAGCTTCAGGCATTTCAGGATATTCTCGAGAAGACCAAGTTTAGTAAAGATATTTTTATTGTAGAACCGGTCCCTCCCGTTAGATCGAAAAAATGTATTACAAGCTCCCATGGAAAGTTTTTTATCAATTCATTCCACCAGCACCTTCTCTGCTCTTCAAACCCGCTCCTAGGCAAGGTCCTGATTGTGTTCCAATAGGGGAGTCGGGCGGAGTGCTCAACTAGTTCGGATACTCTAGCGGCGAAGTCAGCAGCTAAGGATCACCAAGATGCGAGACCCTCCCTTTCTTTTATAACTCTCTAGTAGCTCAGTTACGAATAAACATCCTCTTATTAATGCTTGCATGAGCGGATAGATCAGATATTGATGAAAGTAGTGAAAGAGGCGCGCCAACAAAAAGGATTGTTTTTGTTAATTTTCCCGCATAATCCTATGTGAGGAAAATAGAAAGGGAATCCGTCTATATTGTCCTTCCCGCTTCATTCTCTTCATCTTCCCTTATCTTATAATACACTGGCTAGCGCCTTTATTCGATTAGACTGGGATTAAGACTCCTCGTCACCGGGCTAGCTCTTCTCCTCCGCTTGTCCTCTTTTGCCCTACTCGAATGGGGGGAGCTACTCCCATAAGAGAGCTATGATCGGGCAAGTAGATACAGTAGAGCTCGCGCTTCTTTGATTCGATAGTCGGAACTCTTGGCCTCTGAGTCCATATTAGGTGAGGTCTAACTCAAGAAAGATTAGTATTTGTACTTTTCTTTCTCTTTTCCCTGGGACAGCTAATCAAAACAAATATGGCCCCTTCTCAAAGAGACTGCCCTTAGGACTCTATTAAGTGAAGTCATTTAACAGCGGATAGACACAAGTTATGACAACCCTCACACGAGGGAGGCTTTCGTTAAGATAATTCGCCCATACAATAGAACAAGGAGAGCAATCAACGCTATGGCTACTTTAGGACAATTCCCGCCTTAGGGACCCCTACTGTGACAACAGCTACTACGCATCCCACATCAGATAATGCTATCGACGAAACAACCCTTTATCCAATCCAGCATATCACCATGACCTGGCACAGAACCAATCTTCACTTTTCGACATCCGTAACGAAGCGTTCTAACAGCAGTTACACAGCCCCTCTCCTTGCAGTCGAGTGACTTCGCCCCTGAATGTCTTAGATAGCTGTAAGTGAAATAGGGCTATTAAGTAGTAACTAGGAATGCGGCTAGCTCAGCTAGTATACTTACTTGCTCGTTATAAGGGGAGGCTAAAGCAGGGTAGCGAAACTAGGAACGGAGTTGGCTTCAAAGATTAAGAGGAAAGGACAGAACAAAGAGAAGGGTAGTGGGAAGGACAGCAGGATAACTGCTTACTAACGAACCTAACCTACAATCCATCCTCCCGGACTAAGAAAAGAGAAAGGATAACAGTTACCGAGTAGGAGTACATTAGATATCCTCACATAAAAATAGATATATAAGAAGATTCTCATTCCAGTTAGAAAGCAATCGATAAACCACCGCCCAAAGTTGCTTAGCCAAGAGTAGTCCTATATGGGCTTCGCGAGCCCATATAGGACTACTCTAAGCTAGACAAAATCCCTTCTTTCAAAGTCACTAGAAAGATAATAAAAGAATCAAAAGGTCTAATGAGCTAACCAATCCTCCTATCCTATGGGAATGCTTTCCTAAACTCTGATTGCTCTTGCCGACTCGGAACGAAGAGTTAGCGGTGAAAATAAGTCATTCTTTCTTATTGGCTTCTCTCCAGGTGGGTCACCCAGAGCGAGGGATGTTAATAAGTCACTAACTACAGTGCCAAAGAAAGAGGCTTGGATGCGCGGGATAGCTTGAAGAACGAGTACCGGGAGAAAGGACCTTCTTTTGAGGAATGAGGATTAGGAAGCTTACTCGTAGAAAAGAAAGTTGGCACCTACTAAACCAAGTGCCTGAAAAGGGGTAGTGAAAAAGGCGAAGGCAATCTCGGATTGTTCAGACACTGCCTTTGGTTCATTACCCGTTGAGAAGGCAGTAGAAGAGGTAGCATTCGCAGACCAAAGCAAAGACGGCTGGGGATTCTTCATAGCATAGCAAGAAGGAAGGGCGTGCTTAATACATAAGGACTAGTAGAGGCAGTAGAGGTCCTTCACCATGAAGATTGTATGCTTTCCTTTTCACTCAACAATGAAAGCTAAACGAAACCGATTCCAATCTCTTTTCCATTCAGAAAGAGAATCGATTATGTAGCTCACAGAAGGGGTGATCAAAGAAGTTGACTAAGTGAATGGGAATCCGATAGCTCTACCAGCCTTGGAAGAGTAGTCAGAGGCAATTCGCTAATATTGAACCTTTTCTATCATCTTTCTTTCCTCAAGCATGAAACGGAGTTGAGCGGTAGGCATTCCTTTCCATTACAGTGGGAAAGTGGATGCGAGTCATAGAGTTCTTCCAGTAAGGTTTGAGGTCACAGGTACCAATGCAGTGGAAAAGGAAAACTAGGATATAAACAAGACCCATTTGTTTCAAGTTTAGGGCCCCCTAGTGTTACAAGCTACTGCTTGCGAAGGAAAGAAAGAGTTGCTAAAGCCAGCCCTCTTATTGGTAAGATAGGCTATGAGCTGTAGGACTGCACTGAGATGGCAGATGAGAGATAGTGGAAAACGAGTGACTCTTTTGGGGCTAGCATGAACAGAGCCTTAGAGTCAGGGGGTGGGAAGAGTATTTATTATAGGAAACAACTATATAAAAGAGCGCCTACTCCGTCCCATTCACGACTGGAGTATGGAAGGAGTAGTCTTGAATGCGATGGTACGTTCAATCAGGTCAAACCGTTAGCAAAGCTTCGCGGTCATTGGAACCTTTGGTCCTTTTGACCTCAAATCTCCTACTGATAGGTGGCCTCGGACGGTCATCTACCAGGTTGTTAAGAACCTCTTTGATGTGCCGACGGCGGCCGCGGTCATTGACGGGTCCTTCTTATCAACAGCAGAAGCAATCCTATGGGAGTCTCTTGGGTTTTCCTCTATGATTGACTTGGGCATGTATACTTTTGATTCTCTCTGGAGATATGGTTCTCGTATGTCTACAGCGTTAGAACTCGAGTTTTGCTCCGCATCACAAACAACTCATCACGTGGGTATTATTGTGCATCTTCCAAGCGTCATTCTCAATTTTTTTTTACATTTACATGGGGGCTACCCCTCATATACATATAGAGAGGCGCAATCAGTATAGTAAATATTGGAGGCTATCTGAAGAACTAGATTCATTTGGTAACTACTTACCACCTGACTATATCCCCTCGCCAACTCCCAGCTCTCCGGAAACTTCCCTTATACCCCGCCCTCAACACTGAGAACCCTTTCTTGTCATCTCCGAGCACTGAACCCTCTGGACTCTCCGAAAATGCTGAAACCTACTCTTATTCCTCTTCGGACACTTTGGACTATGAATTTCTTCCAATAAAGTTTTGATCCCTCCGCTCCTCTCCTTTCATTCGAGTAATCAATTACGTTGGACCTCTCCTTTTAGTCGAGTAATTTCATCAATCCCTTGACTTTGTTTATGCCCTTATGCAGTAAAGAAAGCAAGTGAGGCGGGCTAAGATTCCATTCGAAGTAAGGTAAGAGGATTCAATGTTGCACCATCTTCAACTCTTCTCGGTATCGGGAGTTTTTCAAGAAAAGGTCCCATCCTTCAATATCATGATTGGGTCGACCAGGCCAGATCATGAGTAAATAAAAAATCGAAAACGTACATAGCTGTTCCAGCTGAAATACTTGGAATAATTCTACCACTTCTACTAGGAGTAGCCTTTTTAGTGCTAGCTGAACGTAAAGTAATGGCTTTTGTGCAACGTAGAAAGGGTCCTGATGTAGTGGGATCGTTCGGATTGTTACAACCTCTAGCAGATGGTTCGAAATTGATTCTAAAAGAACCTATTTCACCAAGTAGTGCTAATTTTTTCCTTTTTAGAATGGCTCCAGTGGCTACATTTATGTTAAGTCTGGTCGCTCGGGCCGTTGTACCTTTTGATTATGGTATGGTATTGTCAGATCCGAACATAGGGCTACTTTATTTGTTTGCCATATCTTCGCTAGGTGTTTATGGAATTATTATAGCGGGTCGGTCTAGTAATTAGGGGGCGGCCGTTCGGTCGCCTATGAGACTAGGACCAATAGGTCAAAAATTGGTTTGTGCCGCAGGTGTTGAACGATCTACTCTACACAGGTGTGGGCTTACAGGGCTAGGGCTCAGAAACCCTTTCTTTCATTCATCAAGAGGGCTAGACGCGCCTTTCGAACCAGTCTTTATAAACCTGGTCGCTCACTTTTCCGGGCCGGGATCGATAAGTAGAAGTCATAAAAAGATATGTTTCTCTTCGCACCACTGATAGACTACTAAAGATTCAATTAAAAAGGCCCTACTTAGTTTCGCAAGCCTTTGTCATTGTCAGTCAACTAAGTAGGTCGTTCCGCCCCCTGCGAATCCGTAAATCTGAGGAGCATGCCGCAACAAAAGGATGGTCCCCTATGCATTTCATTCTTTCCAGAAAGGAAAATTAGAAGTACCCCCATCATGGTGAACCTCTCCTTGTGATCGGGATGAGGTAAATGCCTCCCAGCCGGGGGGCGGATCGAATCGGAGTTTCCTTAGGTAGCCACCGACCTACAGTTATCCTTAAACTTCCGTGCTTGGTGGAGAAGAAGCGAACAAAGGTACGCTCGCTTGCTGTCTTGTTCTCTGCCGCGAACTGGGATCGCTCGCCAGCTAGGTCAGATTGAAGCAACATTTTTTGAGAACATATTACCCATCTTCGGGGACAAGGGGCGGAACGACCTCTCGATCTACTTACAGCAGCCCAGGAATAAAAACGTCGTCTAGGCGTTCCCTCTTGCTCCGATCTACCCTACGCCTAGGACGTTGTCTGGGCCAAGAGTCATAGTTAGTTGCTGTTTCCATTTGGTTGTTTTTTTCTCGTTGTTGATACCGGCAAGACCCAGCCAGATGATGTCTGCTGGTTGGTAGTGAGAGGACTCTTAGTATCGGCATACCCAAAAGGAGGCGCTAATTAAAAAACAAGAATTTGATTTTCTTTCTTGCTCTCCCTTAGCAGCGGGAAAGGAGTCTATCTATCTGCCTAGCTTTGGTAGATTTCCCCCAACGCAATTCACAGAAATTCCACGAATCCCTTGGTGGATAAGTCCGACGACTCAGCAGCAGTGCGGAATTGAGTTTCTCGTCTGGTGGATCTGATCTATAGTTATGGGGCAATACATACCAAAAGGTTCGAAGAAAGAGTCGTTAAGCCGAGAAAGCTCAAAATTTGCCTTCTCATCAAAAATAAAGAAGGTTCTTCTCAAGACCTTAAGAGCAGCAAGAAGAAAAGTCAGAAGAAAGCCCAGGGGGTAGTGGCCTTTCTTCTCAGAAGCAAAGGTAAGTCCATTCTGTACCAATTTTTGTTGCTGGAATATATTCGGACTAAACACGGAGTATAAACAAACAGGCTGAGATTAGAAAGCTGATCAGACAGAATAACCTCTCTCTTTTTGCCCTAGCTAACAAGTAAACGAGTGAATATTGCGAAAGTGAACGGAATGCTGTTGGCATCTTCGAGCCAACCTGGAGTTCTTCTTCCAGTAAGACGCGTTGTAGTTCGTAGGCTAAGTGAAAACGGGGAGCTACGGCTGATCGAAAAGCGAAGATAGGCGCATAGTTCAGTGATCTATCCACGAAAGAGGGAACTCAAAGAGGCGTTCCTCGATCCGCTTCTTTTAACAAGATAGGACAAGAAAACGAGAACTACAGTTCGAGTGAGCGGCTAAGTAAGCAGAGCTCGTAACCTCGAGTTATGTATATAGAACTAATAATCTTTCTTATTGTCTTTAAAACACCTAAGTCCTAACAAAGCTACCAACCTTCTTGTACTTCCAGACAAGTAAGGGGTTCCTCCAGGTGCTGGTGTTGAGTTAAACAAACAAACGGGAGTCAGATAATAAGAAACGAAAACAAAGACTTTTCGGACCGAAGGGATATATCAGGATAAAGAGTTTGTAACTCACTTGCTTGCTAAAGGTAGCGCTAGCTAAAGTAAACTTGAGCTACGGACTTAGGAAAGAAAGGCAGCTAGTCGCTTAGCGGAGCAAGTTCTATGGACAAGCTTGAGTTAGGCATCGATTGAAGAGCAAAGAAGAAGGCCAGACTGTTGCCCGGCGGACAAGCTGGCTTTGAATACTCCAGTATCTCCGGTTTCGGGATAAGATAGGCTTCTTTACCCATCCGAGGAGTTGACTTCTTGACCGATAGGGGAACGGCAGCATATTCGACCATTGATCGGAGAATAGGCACTGATCGGAGCCAGTTCACAACCTGGGATAGCTAACGCTACCTTGCCTGAAGACATGGGATTGCCGTAGAAGTAACATGCCAAGCAAGAACTTGCTCCTTTCACTTCCCTAGCTGTAGATCGAGCAGGGCGTACGAACGAACTAGCGTATGCGAGCTAGCGATTCCCAAGTAGTCGTTAATAGATAGAAGAGGTTGCTTTTGTTTTCCCGGGAAAGATGCCAATACCGAAGACATCGCATGCTAGAAAGAATGTGCTTTCTTCGCGTCATTACGGTTACCTGTTCACTAGTAATCTGGTCGAAAAAGATCCAACGTCGGTAACAATCGCGATCAATTAGAGGAACGAGACTGGAAGGTCCCCACAATAAGCAACTATATAACTCTAAGGATCGTGTCATACTGCCTTTCGCTGTGCAACTTCCACCAGCAGTATGACCTTCTGAATGCTGCTAGGTTCGGGACCTATTCGATTCCTTTTCTAAAGGAACTTTTATATAAAAGGAAGCCGAACCCACCTTCGATGATCTTAGACTTCACGTTCTCTTCAAATTCCAAATATCAATAAAGCCGACTGTCATTTTGAAAGCTGAGTCTTCGAGAGGTCTACCGTCTACCGCATTCACAGACCCTCCTCCGCTCCGCCCCTCCTATGGGTCCGCGGATTGCTGCCTGGGGTTCCTCCTTCCTCGCCTTACTACTGACTCCCTCCTCTCTACGTTCTACGTAGGTTGGTTTGCTAGGTTGTTAGCGTAGCTGCTCTTCCTAAGTAAGCAAGCTGTAGAGAAGGAGTTGCCTGCCGTGTTGCGCGTAGCGTGTACTGAGGGCGGGCACGACTGATCGGCTAGCTGCTCTGTTGCTATCGCACAGTAATAAGTATTCTTTCTCTCCGTTCGGAATGAGTTCCTTGAATTGATCGGTTTCTGGCTTAGCTCTATTCCTATGCATGCAAGTGTAGCTCGTGCGTGCCTTCCGAGTAGGAGTTGGCATCCCTACGAGGAACAGTCAGTGTAGTGAGCGTAAAACTAATGGTGAGAGGCGTTAGCCGGGCCATTAGTCTGAGCGAGCGGCTAAAGTCCACTAGGGCTGTTGCTTGTTGTTTCTGTCATTTAAAGAGTATTCTTTCTCTCCGTTCGGAATGGGAACTCCAAGTTGTTTAGCTCTCTTTCTTCTGATTCCGGTATTTCTCCAGCTACTAATACAGCAAGACCAACTAGAGAATATCCTGTAGTTTATGATTCTGATTCCTCGGAGTTAAGTTCCCTTTCATTCTTATCTTTCGCTGGATTGCCCATTTAGTTCCATTTAAATGAGAGAAAAGAGGGAAAACCTATACTATGATGGGGGTATTTGATTGGGCCTTCCAACGCTGACGAATGCATCCTTTCTCTTCTCAAGCTACAAGAAGTACGTACTTTCAGATGGTAAGGATTGAACTCTAACTCAGAAGTGGAATTCACAGCTATTACTACTATAGTAGTCTTTCATTAACAGTTCCTCCTGTAACTCAATAATTGTAAGTTGATTCATAACCACGCTAGTAAAGTTGCCTTTGGAATTGGAAGTTGAGTCCGATCTTGGTACTCTCGCTAAAGTTTGTTGACATTTCGCCCTCTAGAATAGATTGATTTACTGGAGTCCCTATCCGAGTAATGAACTATAGAATTGACTTCTCCCCAGACCCTTCACTATCTTGTCCAGGTGTTTCATTGGCTTCGACGCCTGGACTTTCATCTTTAGATACTCTTTCCGGCTTCATTTACGGATCCTGCAACTATAGAATGACCAGGATTTGATCCCGTTTCACCTTCTACTTCTATAGATAATTGCCAAGCCCAGCTGGAACTATTGACTTTACACCCTCTACCGCAGCTTTACTTTTCAGCTAGGGCGGCTATCGAACGTGGCCTTCTACGCCCGAGGAACGCCTTCTATTTGACATCCCTTTCACCTGGCGGCTAAACCATCCGTTGATGAGGGAACTATTGAAGCTTCTAAAGCAGCCCTTTCCGCTGCTCCTACATCTGATGGAAAGGAAGGAAGTCCACTACGGTATGGAGGAAGTAGTTGAATAAACTGATAGAGCAGGCCAATCAAAGCAATCATCTGCCGCTTTCGTAAGTCAATTCCTCAGCTACGGCTATTATTGAATTTCATTTACTGGGCTTTCGGATGGAATAGTTTACCTTGACTTTGAACCGTAAGGTATTGAATAGACTTCCCCGTATTCATTTTTTTGAATATCTCTTCCATTGCTTGTTAGCATTGGCTATCAGTCAATTCCGAGGAACGAAGTCACTCGACTAATAGGTCTGCAAGCCTCCGGTGGCCTTACTTTGCTTTCAGGTCAATAAACGAATCTAGCTTACGCTTGGACTCCTGAATTGATATAACTCAAATTGCATGTGTTAAGCGTTCGAATAGCTGGCTTTATCAAATGTCTTCCTTTTTGGGAGAAGTAGGAATTGTTAGCCAAAGACTTCCGTCAATGAACATGAGAAAGAGGCACTCCTTCTCTTGTCGAAGATGAGGCCTGCAGACTAGTCTTACATATATTACCTCATTTATCAAATAGCGCTCACTGCCCTCCGCTCAGAGTTGTCTCAACTTTCACCCAAGATTCAGTCTTTACTGAAAAATTGAGGGATAGTGGAACTGTTTGACTAAGGCCAATAAGAATTGGATAACTCCAGTGGGCAAGAACTCCTGACTCTGGAGAGGAAGGTGGGTCTGGTTCTCTGTTTAGTAAAGAAGAAGGTGGCTCTGATACCATATGAAACTCTAAGAAAACAGAGAGGAGAAGAGGTTTTTCTCTGAATCGATTTTTTGATTTATTTTTCGGCTTAATTACAAAGTTCCCCTTTCTCTAAGCTCTTATACCCGTGAGCAAGGGAACAGAAACCCTAGGGTTACTAATCCAAATCCGACACGCGTCCCTCTAGTCACTCGCACTCATGGCCGTCTACGACTACACCATCCCCGAGGAACGCCTACGCTCACTACTATTATAGTACATGCAAACACAGGAAAGCAGCTACGCCCGAGGAACCCCTTTCTAAGACAACAGATCGAATTCCTATACTCCCTTCGTTCGAAGACCACTTTTTCCTTACCTATGCCCCTTAGCCAATCAAGCCTGACTTTCCCTTCGCTTCGCGCCTCGGTCTTTCTTTGGTCTATTTCTGCGGGTCACTAAACTAACCTTCTCTGGTCCGCTTTGGCTATTGAACTAATATTCTTCCCCCCCAGGCCCCAAGCCAGTCAGTTTGACCAGGAATGCCTGCGAACGGTATAATCATGAATAAGAAGAGCAAGCGGTAAACGAGTGCGAAGGGAGCGAAGCGAGTTTTTCTTTTTAAGGAAGTCTAGTCTCCTTCAGGCGAGTTGAACGAACGGTCTACTCAAGCTCCTGAGAGCGAGCGGAATAGCCTGGTCTTTCAAATCAAATAAGTAGTTAACCCGCGATTGACCTTTATCTTGTCTACCTCTGTCTCGCATGAGAAATCGAATTCGGGGTGATCAAAACTAGCCAACGAACTCAGCTGGCGAGTGTGAGGCTATAGATATAGTCTATAAAAAAAAAGAATTGAGCTGACAAAAGGGAAGAAAGAGTTGTTACGCCGCTGGATTAAGACGACTTAGCTACTTGTCTGAAAGCGGAAAGAGAATAGGTCTCAACGAGCATTCGGTAAAGCTAGAAAGCAACCGAAAGAGGCACATCAAAAGGTCTGAAATCAGTCTAGCCTGCCAAGGCAAACTAGGGATGGATCGGAAGTCTGATAAGGCTTTCAGAGATAGGGACGTAAGCGGTAAGAAAGAGTCACTCAGTAAATCGGTGGATCACACACTTGTTGGAGACAGGGACACGCCTGACTATTTAGAAAGTATACAAGTGTTGAAAGAGTGAAGTCTGGGGACAACGGTAAACGTGAGGAATGGGATCTCCAAAGCTACCCGCCCTACTAAAGAAGTTCGCAAGCAAGGGACATGCCGAAGACCTACCTTTCCAACAAAGTTCAAGGCGAGGACTTTTAATTGACGATGCGTTCCGTCGTCAGCAAGCGGTGGCCGACAAGGCCCTTTTCCCTAAGGGAAGCGAAGAGGACTGGTTTGAAACTCGTTCGGTAAGATTCTCCTGCATTTACCCAAGGCACTGATATTCTGAGTCTCTCAATTAGTTGTGTTTTAGGTAGGGGGTTTTCGTCTTATTCTTATGAGTGGCCCATGTGAATATGAGCCAAGGCAGGAGCAAGGATTCCTGAAAAAGAAATTAGATATAAAAAAAAGTGCAAGTCGACGTTTCTGATATGAAAGGTTAAAGTGTAGTGAGAGCCGGCTCTAAGCAGGATCCAAAGACGTTCAAATCCAGTTCCAATCTGGATATCAAAGTGCAGTTCAATCGTCGAAAGTGATCTCTCTTTTAGTCCGGTCTTCTTTTCTCTTTTGAGTCAGGTTCTTAAGACAGGGCAGGGAATCGGGTTTTATGAATATCTCTCTTCCGGCCGTTAGTTCGAGATCAAAACTTTACCTGAGAGAGACGTCACTGATAGTAACAGTAGGTGCGCCTTCAGTTGAGGAGAGCTCTTTTCACAAGCAGTGGTTATGAGCTCTTTCTTGTTTCGATTCAGAAGAGGTAATGCACTAGTGGATTGAATCACCTTTTTTTTAGTGCCAACAAAGTGCATGTGTTCTTGGTTAATAAAAACACGAATTTCGATAGGCTATAGGACTGATACTATAAGTAGGATAAACGCCTTAAAAGCAAAATGAAAGGTTTCAACTTATCCAAGGAATCTCTTTCTTGGCATTTGTATTTGAGAGAGAGCTATGCTTAGGGTCAGTTCCTTCAGTAAACTGATTTGGAAAGTGAGAAGTTGACTTTAGGTAAGTAAGTAGTTCCGTATGAAATTTAGAAAACTTTCATATCTGTCCGGAGGTCAATCTTAAGTGTTGGAAGCTACTGCTAAGGTACTCCCCTTCATCTATAAAGGATAGGCAATTGAGAGAATAGGGCGATAGCCTGGTTTTGATTGAATATCCTTTCCTGTGCTTATCTTGTATGAGGTATACCGAAGATCTGAGTCAAAGTAGGTAAGAGAAGGGGATGGATGTCTGAGCGGTTGAAAGAGTCGGTCTTGAAAACCGAAGTATTTCTAGGAATACCGGGGGTTCGAATCCCTCTCCATCCGCGAAGTCATAAGTTCTCTCTTGCCGCCTGATAAGAACGAATCGGATCGACTCGACTGATATGATAGATGGAATGGGTACCTTGTGTTATGATTTTGTTAGGACTTTGTCTCCCTTTCGTTATCTTCTCCCGGTTGGGGGTGGATCACCTTACCCACAAACAAAAGGAATAACAGAGAATGCCCAGTCAATAATAATTAATAATCTAATGGCAACTCGAATTGAATACAGGTAAAAGGCAGATAGGAATGATTGAAGATAGGAATCTATTTATCAAACGGCTTGCGCCCCTGCACGTGCACCTTATCGGATGTCGCCCTTGGAACTGGCAGAACTTAGGAAGCAGTTGACAGACTTGCTGGATGCAGGCTTTATCCAGCCTTCCAAGGCACCATATGGGGCCCCTGTGCTGTTCCAGAAGAAGCAAGATGGCTCTTTGCGCATGTGTGTGGACTATCGTGCACTGAACAAAGTGAGAGTGAAGAACAAGTATCCTGTCCCTTTGGTGGCAGATCTCTTTGACCGATTGTCCAAAGCCTCTTTCTTCACAAAGCTGGACCTTCGTTTGGGCTATTGGAAAGTCAGAATTGCAGAAGGAGACGAGCCTAAAACCACTTACTTCTGTTACCAGATATGGCTTTACGAGTTCCTTGTCATGCTGATAAGAGGCCAGTCGATGAGGCTCTTGAACAAGTTCCCGGCGAAGTTAGCCCTATAAGTTAACCGCCTGCCATGTCTACCCCGAAGGTATCTTTGGTGCTGTTGTTCCTAAAAGTCTCTTCTTTCACTTTGTTTCCACACTTCCCCTGCTTTGTTGTTTCAGAATTGAGAGGCACGAGATAGCCCCCAAAAAAAGGCGAAATCCACGAGAAGGCTTCTTTGACTAAAGAGAATCGCTCTTTTCCACCTCCCTAATGGTCTGAAAGCGGAGAGAGGAAGACCTCGATCGGGGAAAGCCCTTGCTATTTTCAAGTTCAAGGTTTGATCAAAGGATCAAGGTACGAGCAGACCAAGAAGCGAGGACTTACGAGATGCTTTAGTAACAACCTGAAAGCGCTAAACTGGTCATAGTTGCGATGTAGAGGAGAACATTCTCTACCAGAACAATACCAGAGATAGAGGAAATGGCACTAATAGTAACTAGTAAGCCAGCTTTCTACTAATAACAAGACAGGGAAGCGATCTCTTCATGTCGAAAGGAGGATGTCGTTTTCCTCGAGTCTCCACTGGGAATACTACTATACTATAAAGATTGATCAGCATTAGCCGGCCTTCTCTTGAATAAAAGCGAGAACCTGGTAAGGGGAAGGGCGATCGGCAAGTGTTGCTAGTTGAGCGATTAGCCTCGTTAGCTCTCTTTGCTGTCTAGTTTCTTGACATTCCTAACTATAACTTAAGATTTGATCTACAACTCATTTGTTCAAACCCGATAACGCTTTTCTTCCGATCTCACAGTTCCTTTGTCCCTTGTAAAGCATTCTATCTGTGGCCACGTGCTACTGTGGAGCTTTTGAATCGACGTCAACCAGAAGAAAGAAGAGAGCCAGAGCGCCATACCCTTCTCGGAAGGAACCCGCCCTTGGAAGAGCTACTAGGGCATCGCATCTATGCTGAAAGAGCACAATCCTTGATCTAGAGGGAGCCATTCCTTACCTTAGAGGAAAAGACTTAGCCCAGTCCTCAGTTTCCACTTACAGAATAAATGAATCACTTTGCCACTCGACGAGGCAGATAGCTCCTCTGGTGCTACTATAGCCTTTGTGGTTGCTTCGCTAGGAAGAGCAGCACTTATATTGCTTGGATTATCTATTCAGCACAAGCACACCGCTGTGCTGTGGAAGAGTAAACGAACTACTATCGATGTGTTACCTAAGAGATTAGCCTAAACGAGAAAAGGGAGTGTGCTGGACTGACCTTTGCATTTCTTTCATATCTCCATTCTCATAATATATCCATTCTCATAATATAATAGGGAGGCGCTGACCTTACAATTGTAAGAGTTAAATGACCGACTGACTTGAATCTGTTTGCCGTTGGTCCTTATCTTTAAGATAATGTTAAGAGTGGTAAGGGCTATGGGTATAGAGCCTTGGCCAAGAGGTTGATTGAGTCAGTAGACCTAACGTACGCTTAAAGAAAAAGACAGCTCTGATCACCCTTTATCTTTCTCGGACTCCAGTAATATGTGTGGGGCAAGTAAACGTCAGCAACGAGGACTTCTTATTGGGCTTACTGCCTCTGCCTGTCTTAGGTACCCAAGGGGAGTCCTACTAATAGCCATGCTGAGCTGTCCTTATCGTAGTCATCTTTTCCTATTATTGAACAGTTTCAACTTGCATGTGTTAAGCATAAGCTTAAATCTTTCGAAGCTCCACTGATTCATCAAAATGCATCCAAAAATCCCGGGATATTAGCACTTTTTCAGGCTGTTTTGACCTCGTCAATCAAAAACAATATCTTACAACATTCCTAAAATCGAGGATCTGAAACCGGGTCTTCACGTACTGATGAGGCTGAAAACACGGGATATAAGGAATTGATTCAAGTCGAAAAACCCTAAAAATGCGGATTTTCGTGCTGCTTTTATTCAACATTTCAGGGGATTAACGTTTTGTCTGGATGTGCTGGAAACAGATGAATGAAAACAGATGAACGGATTCCCCACCCAACCAATACAAATTATGCTTCGCTAACAGAAGATCAAGAAAAGTCAAAGATGGAAATGCCAGAAATCATCCATCCATATCATATGCACTATCGATCGACCATCACTCCTCTGTGCTGGATATTAATTTCTCACCCAATCGACTCGTTTCATCTGCTAAGTCTGTCTGGCGATGGTTGGTCCAAGGACCCGCTTTTATATAGTATAGAGATATAGCCCCTCTTCTTTCCTGTTGAGGCAATTATTCCTCAATAGTAAAAGAATCATCCATTGAATGCGCTCTTATTGGCGTAAAGAAAGAAGAAGTCGAAGAGTCGTCTCGAGTGAAGGGATGTGCTGCTTTGCTAGCGAAGTAAGCTAGGCGTGATTGTCGTATTCAATGACTCTGGTAGCTAAGATGCGGTTGGAAGCGAGGCTGCAGATCTGGGTGCGCTTAGATGCCCCGAAGCAATGACCAACTCAATTCTCTCCGCTAAGTAACAAAGATGAGAAGAAGCGCACTGATTTCCCTCAGCTAAATCTTGGGCATTCTTGTTAGCCTTGTACTTGGCGGAGCTCTTTCTTTTTCTCGTCTATCAGCTTCTCTGCTGACGCCATTTCCGAATGTCTTTAACCCCCTAGCAAAGCAACCAGCTCTTTCTCGTCCCCGCTTTCTAAGCAAGGAGAAGAGTCGTCTCAGTTCATCATAGCTCGAGAGTATGTCCTGACCTCTCTAGCACCATTAATAACTGTGCTTGTGCTTCAGAACGGAGAAAGCTGCTCTTAGATAAGAGAAGCAAAAGAAGTCTCTAGCCTTCCCGCCTTTCTTCAATCGGTCATGTAATACAGATCAATCCGCGAGGATTTCTTTCCCTTGAAGGTCTAGCGCAGCAATTTGCCTTATATTTTCTTTGCTGCTATGCTGTGTCCAGTGAATAGAAAAGACCTTTACCGGGAATAGACCGCCTTTTCGACCGATGTGCTGTACCATTCTTCTTTGCTTTTCGGATGCTTTGAAGCACACCTCTTCCTTGCCTTTAGTAATCTACGGAATATTCATCTGTGCTAGCACACTCCTAGCGAAGCAACTTTTGAGCCAACTGTTGCCGATGCCGGAGCTGCTAAAGAATTAGATGGGCACAGACATCTTCCTCGGTGCTTTCTCTTCATTTCTTTTGTTTGCCTAAGCTAGAAATGTGCCTAGGACCCCTATTTAGCTGGACGCCCAGAAGGAGTTATAAGGGAGATTTCAAATCGGGCAACAAACAAGATCAGATCGTAGAATAGAAAAGGATTTGCTGCCGCTACTGAATCAGAGTCCACGGTGCAACAATTTCTCATCTGCTCGCGAATTGGATTCGAACCAATCAAGCTACTTGCCCTTTAGTAGATCGTGAGTGGGTCAGTCGTCCTCCTCATTATAGTCCTCCTAAAATCAATAGCATTTCGTCGGAATACATCCTGTCTTTTCACCTTAGTAGTCCTATGCATAGTCAGTACTATAGCCCCAATCATGGCTACTAATAAAATAAGACTAGGAACCAAAAACCAGACAGAATAGTAGGTATAAAGTAAATTTCCCAATGTTTCCAAATTAGTCCAACTTCGTACCTTTCCGGCATAAACCGTATATCTAAGAGAGGTCGTATTTCTTTGGGTTGGTAGTAATGGAATGCTTTCATTATCTAAAATGAAAAACATTTCCCACCAAAAGATCAGTCCAATAATCCCACTCACTGGTAAATAGCGCAATACTTCTTCGTGAATCTCCGCTATTTGAATATGGAACATCATAACAACGAAAAGGAATGAAACGGCTATAGCTCCTATATGAACTACTGGGAAGATCATAGCGAAGAAGTCGAGACCTAACAAAAGAAGTAAACCTGAAGTGTCGCGAAAGACTGGGATGGGAAACAAAACGGAATGTACCGGATTTTTAGCACGTGCAACCATCAAACCAGAGACCAAAGCAGGGCTCGACAAAACAGAAAGTATCATGGGTACGTCGTCCTTCCCTGAAATGGAACTTTCATGCTAGTTCTTGCTTCAGCATGAAAGTCGATCTATTACGACCAGCGCGGTTGTTCCTATTTCATTTTCTTCTTCCTTGCCCTTCTTTCTTAGAAAGCACTCACTGAGTTACTTACGGAATCTCAATGCATCCATTTAATGCATTCTTTTCGATCTTGTACCCACGGAGCGGTAGACTGAACACCACAAAAATCTCGATTCAAAAAAAGGTACAGGCAACTAAACCTGTGAACTCAGATAGCCTTGTGGTATGGTAGCGAGACCCAATCTTGAGTGAAAAAGATACCGCGGCTTCGATCTTTTTCAACATAATCATCGCGCAGTAAATCAACAAAGCCCAAAGTTTACTCTCTATCTCCTTCAAGTTTACCTACTACTGTTCCCGCGTGAATATGATCTCCACCAGATAGACGTAAAGCTTTAGCTAGTACACGGAAGTGCATACCATGATTCTTCTGTCTATCAATAACAGCATGCATTGTACGGTGGATGTGAAGAAGTGGGCCATTATCTCGGCAATAATGAGCCAAACTAGTATTTGCGGTGAATCCCCCTGTTAAGTAGTCATGCATTACGATAGGAACTCCCAATTCTCTGGCAAATACAGCTCTTTTGATCATTTCTTCGCATGTACCCGCAGTAGCATTCAAATAATGCCCTTTGATTTCACCTGTTTCAGCCTGTGCTTTATAAATAGCTTCGGCACAAAATAAGAAACGGTCTCTCCAACGCATAAATGGTTGGGAGTTCACATTCTCATCATCTTTGGTAAAATCAAGTCCACCACGTAGACATTCATAAACTGCTCTACCACAGTTCTTCGCGGATAACCCCAATTTAGGTTTAATAGTACATCCTAATAGGGGACGTCCATACTTGTTCAATTTCTCTCTTTCAACTTGGATACCATGAGGTGGTCCCTGGAAAGTTTTAGTATAAGCCGGAGGGATTCGCAGATCCTCTAGACGTAGAGCAGCCAGGGCTTTGAACCCAAATACGTTACCCACAATTGAGGTAAACATGTTAGTAACAGACCCTTCTTCAAAAAGGTCTAATGGGTAAGCTACATACGCAATAAATTGAGTTTCTTCTCCTGGAACGGGCTCGATGTGGTAGCATCGTCCTTTGTAACGGTCAAGGCTGGTAAGCCCATCGGTCCACACAGTTGTCCATGTACCAGTAGAAGATTCAGCAGCTACCGCAGCCCCTGCTTCTTCAGGTGGAACTCCGGGTTGAGGAGTTACTCGGAATGCTGCCAAGATATCAGTATCCTTGGTTTCATATTCAGGAGTATAATAATTCAATTTATACTCTTTAACACCAGCTTTGAATCCAACACTTGCTTTAGTCTCTGTTTGTGGTGACATAAGTCCCTCCCTACAAGTCATGAATTTAGAATTCTTGCAATAAAACAAAACAACAAGGTCTACTCGACATAAATTAGGAATAGATTTAATTAACCTTTTTCACAGGAATCTTTCACAAAATTATCAACTAATCAGAATGATTCTTTATTAGACCATGATATTTGATTCGCCAACTACATCATTATTGTATATTCTTTCATATTATGTATAGCGCAACCTAATACTTGTTTTTCAAGTTTAGAATCTTCCTCCGTACTACTTGATTTTAGTGTTAGGCCATTCTCTCTAGCTGCTGCAGTAGCAGGCACGTATCGAAGGGGAAGAGGAGTCAGCGTAAAGTTACGTAGCCTAACTGTTCGGTTATTCAATCATATCCGATTTGGTCCATTTCGCATAGACCTTTGTTGTTCACGTCTTTGCTACGAGCGTGGGATATGGCAAGGCTGAGCATTTACCATGGCAAGTGAAGGTGAAGACGGCATGAATGACCACCTATTCGGGTTCACTTCTTCTCCTCCTGCTTCTGATGGATCTTGAAAAGACGCATCCACCTCCACCTCTTATAGGCGTCGGGTCCCTCATTCGGATTGAGATTCATCCAAGTCTACCGTAACGAAGCTCCGCTCCCTCCCTATTTCACTTTTACACCTTGAAAGGGCCTGGCCTGTTTTTCCATCTATTTGAAATCCTGTCTTGTTCAAGCTATGGAAACTAAGTTTTCAGTATAGCGAATCCACAACTGCCGCAGAAAGAACAGGAGCTGGTACCGAAAGACTGGCAGGAATGTGATATATATTTTCAACAACAATAGCAAGGGTAGAACTTCATGATTTATTCGGTGTTAGCGGTCTTGGGTCAAGGGCGGAGACCCTTACCTAGTGAAGGAAAAAGTTGAGTGAAAGCCTTCGCTAAAAAAATGGTTTTGATAAAAAAAATGGCCAAGCCAACCCAATCCCAGTATTGGAATAAGCAAAGCAAATAAATTGTTAACCGCCCTGACCAAGTCGTCTTCAAAAATATTCCAACAGTGATAATAAAAGGGAATCTATCAATTGAAAGGATAGGGGCTTTGCCGCCAACCACTCCCGAAATTACACTTAACAAGGAGTGGCCTATGATCTGATCGACCCCTTGCTAAATGAAATGTGTAACATGCGTCGCTGGATATCTATGAGACCACATCTCGTTCACCAACACTCTATCTAACTTTTTCCATACCCTACCATTAGTCCACAGGGGATCCCGTAAAAGGCAATGAATTCAAACCACACTGGATCCCACCCTGAGCTAAGAGCCATAGCAAGAGATATAGCGTTGGCTGTAGGCTTAGTGAGCTCATAAACTGGCAGAACTATCATGCCTATTACGAACGAAGAGAAGGAGCAACTAGCAGAGAAAAAGCAACTAGAACGAGAACCACAGGGCATTCGATGCAGCAGACATCTAGGCAAATACTTCCATGTTACGGCACATACTAAGACATATTCTAGAAATGACAGACGGACTAGCAGCAATACGAGCCGTTCACTACGCTCACTGAAACTACACCGGTAACCAGAGTAACGAACTCAAAGAGACCAACGGGATACAACGGGATACTTTGAGACATATACTCCACATTCCGGAATTCCAAGGAATAGATCAAATGAACGAGAAGATATATTCGAGAAAGACAGAAAGCATTGTTTGATCAGCCAGAAGAAAGGCTGTTCCGAAGGTTCAGTTGCACATGTGGTCTAAAAAAAAAAGGTTACAGGTAGTGATGAGCAGCCTGCTCCTAAAGAACGGAAGGAGACTTGTTTCTTCTCTGCGATGTTCTTGGAAACCGCGGCTTTTGCCCGCCAGCTGCGAATCATTCATTCAGAACCGGATGGTATTTTCAGTCCAGGTATTCTCTTTCAACCTGGGTATACTTTGTACCCGTTTGATACGAAATTTAGTACCTGGGCAGCTAATGAAGGGTTGAGGTTTTATGAGGCTTGGCCTGGCTTGGTGTTTGATTCCCTCGCCATGGGCTTCGACCGTTCTTCTCAACCTCTTTATTCCGGACGTTTGGCTCAGTCCTTAGAGGGTGCCGGAAAGCGGCGTTTGTTTGTAATCGGTAACTGGTTTAAACAACGTCTGTTATACCCCGTGCATGTGTGGGGTATGTCTGTTCTGAGGCGTATTCCACAAGACGGTACCTTCCATCAAGAAGGTCCTATCCATCGTCTGGCAAAGAGACGCCCAAGATTTATAGCAAGTTTTGACTTGAGCGCTGCCACCGATCGGTGGCCCGTTCCAGTCATATATGAGCTTATGGCGTGCCTTTTCGGTCAAACGATGGCATCGTGCATTGTCAATGGTGCCTTAGCACTCAACTCATGCTCTTTGAAGTCCGTTACTGGACGGCATGACGAAGTTGTGTTTGTTGCAGGTCAACCTTTGGGTTATTACGGCTCCTGGGCTCTATTCGCGTTGTCCCACCATGCTATTGTGTGGTTGGCAGCATTACGAGCATATCCTCATCAGACGAGACCATTTCTCGACTACGCTTTGTTAGGTGATGACATCGTCATTGCCGATCGTAGTGTGGCTAAGGAGTACCGTTCTCTACTTGATGCTTTGCAAGTAGATATATCTGATGCCAAGTCTATTGTATCTGAGACGGGTTGTCTTGAGTTCGCCAAGCGGTTCTGGGTTAAGATAATGTCGAAGGACTTATCCCCAGTATCTGCGAAAGCTGTGCTTGAGAGCTACTTCCTCGTTGGTACTCAGCAGTTGGCCTATAAGTACAAGTTAAGTCCTAAGACTTGTTTAAGGTTAAATAAAGCTGGATACCGCGTGCTAGGACAAATGGACACTAAGGCCCTATCCCGGCGTTTTAGTCGGATTCAGGCGTTATCTAGTAAGCTTTTAGTCGGTCCTGATCGGTTACCACTCGAGTGGTGGATTGGAAGAGGATTACCTCTCAGTCCCTATCTTCGTGGTGTGTTAATTGCAAGGATTCGGGATGGGATGAAGCTTAAGCAGCTCACGCCCCCTCCCTTGGAGATGTTCCTTTGTGGAGAGCCAGAAGCACATATCGCAGAAAACACCGCTTATAGACATTGGATGAACAATGGTGTGAGTACGTGGTATGGTTTGACTTCGTTCCTCGTCGTGCCCCTTCCCACCTAGGAAGAGATACTAACCCGCAGCTGCACTTGGTGCAATCACAGCAGAAAACCCTTCTCCTAGACATTCCTCGGGGGCAGAGGCAGACCACACCACCTCGGAAACAAACAAACAGCACATGCAGCAACAGAGACAGGCGTTCATCGCTCACTTACCTGCATACATCATGACAACTTGTTCCCATCTCACCATGCAGAGGCGCGTAGCGAGTAACAATCCGGTCCATGTGCTGGCTTGGCATGGAAGGCAACCCTTTCTTTTTCGCAGCGCTACGCGCCTCCACATCCAGTGAAGAGATCCTGGACAGGGAAGTCAGAAGGTCTGCAAGCCTAATTCGGATTCTTTTTCTAACGTCCTACCTACAGGCGCATAGGAGCTAGTTTTCCAACCGCGGAACAATATCTTACACTTAGATTCCACCATAGTGCTAGATTAACTAGGCCTTCCCTCCACTAAACAGATTCCATCGTAGTGGATAGAAGTCTTTAGACTAAGGGCAGACCGCAAGGGCAAATCTTAGGCATTGGTTCCGAGGACGGTAGCCTACTGAGAATGAAGGAAGAGAAGCGATTTTGAATACTTTCTAAGATATTTCCACGATCAATACCGACTTCCTTATCTTTTCTCTTGGAAGTCGAACCTTTATCTGTATGGAAAAGAGGGCTTCTACCTATACTTGGTTGAGAAAAGGCTGAATGGTCCAGGACAAGAAAAAGCCACTAAAAAGAACTGTAGGTCATACAGGGTAGTCCACTTTATCTTTATCCGTGACTCTGAGTACAAGATTTCCGGGATCAAGAAAGTCGCAAATAAATATGCTGGGCTGGACTCTTTTCTCGTATGCCCGGAAAACGTTGTTTCGATAGAACTCAGCTTGCTGCATAGCTCGTCTAAACAAAACACTCCAGATCCGCACTTCCCTTTAAATATACTCCAAAGGGACTAAGCTTGACTAAGAAGGGCTTTGCTCCGCATGAGACAAAGTTATCAAAAAGTACTTATTCCAATTTAGATAACCCTGATTAGAAACACACTTGACCGTTTTATAGCTTGAACCAGAACAAACATTAAACGCCCGGAACAACTGTTAGAATTTCGATCGAACAAGTATTGGCATTCTGTCAGCATTTCCCCCGCGGTCAGTGATAGTTAGGAAAGGAAGAAAGAAAGGGTGATACACGAGAAGCTAGGTCAGGTATATATAGCGATTGCAGATGGTGCACTAGTTTCGTATACATAGGCCCTGGTTGGTAAGGTGATAAGATCGACACATTATATGCGATCTACGTTCATAGGTTCTACCTTGAAGCTCTTCTCCTTTCCTTACTCTAACAAGTAAGCAAATAGGAATTACCTTGTGAAGCAACGTAAATTTAGTGAGTTTTGGGAGGTTAGCCTTAACACATCCAAATCTTCCGAAGTCAATGCGGTAGCAGCCACTGTCCCGACCTTTTCTGTCACTCCTTCCTTTGAAAGGAGCTTTTCGCCGTTAAACTCAGCTCTTTTTCCGGCTTTACCGATTAGATCAGTTTAGACCTTACCTTGGGATTGGTTTTTTTCCTTTAAGGCTGACCCTTCTTATAGAAAACTGGCATCTAGAAAACTGGCATCCAAAGGTCCTCAACCAGGGAGAAATCGACAAATCTAGGATAAAGTCCACTTGAATCGACCAAAGGGGAAGGAAGTATGAAAATCCAATTCTTTGACGGAAACTAAAGGCTAACCTACAAGCTAGCCTCGTCGAATCGACTCACTCTTTTAACCTCAAAGGGAATCTGAGAAACAAGTAGATCAAGGATTCCCATGTTGCCTATGGTGTCTTGCGAATTCCTAGGAATGTTAGGAATGGGAGAACCAGTAATCCCAGTAATCCCAGGAATAGGAGGTAAGGAGATTAGTTGGTTAGGAGGGAATCCCAGCAATATTAGCAATGAGGTGATGAGATTAGGTTTGTTTTGGTTAGGGATTAGAGGAATGGGAGAGGTAAGCCAAGTAAGGTAAGGAGTTTTTGTTTGTAGAAAGTTCCATTTCAATCTGCCTATCTGTTTGAATCAGTTTATCCCAAGGAAGTGTTTTACTCTTTTAATTGGTAGTTGGTTAAGGAATCCCTGGAATGTTAGCAATGGGAAGAGTTCACCTTGCTCCCTCTCCTTTTAGTCGAGTAAGAAATCCCTCTCGCAGAAAGCTCAAGTATGTAAACAACCTCTCCTTTTAGTCGAGTTACGTCAGTACCTCTCGCAACAAGTACTCGAGTCACAAAACTAAAGGTCGAGTCATGACTCCTCCCACCTGAGGGCGAAGTCATTACTCGACTGAAAGGAGAGGCGTTCCTCGGTATGCTTCACCCTTTGAATAGCAGGAATTCGTAAGTGGAAAGACCTCGCACTTCACACTATACATAGGCTGCTAGGAAGAAAGAACTTCGTACCTTTTAAGCTCCTCACAGAAAAACGGGACTAACCAAGGACGGCGAATGAGCAATACGTAGAGGTTTTTATTTTCCTATGGATTAGCGGTACGATTACGATGCTTGCTCCGAATGGAAGGGGGACCCTTTTCTTTACCCGGAGCAGTGAGCTTAATACCTAAGCCTGTAATTGACGGCTGGAGGGAGCTATCCTACTCTGTTAGGAGCTGGCTAGGAAGTGCCGTACGTGACATTTCTTTCCTAGGACTATTTAGGGTAGGGAATTTCTCTATTTGATTGAAGTGAAGGTCTGCAAGCGTGTACGGGAATTGAAGTGATTAGAACAGAAAGAACTGAGCTTGCCAGCCAGGAATGAAGAAGCAACGGACGCTATTCGTGGACAGAGCAACGGACAGATAGAGGTTTTGTTCCCTTCGGACCCTAACGTGATACGTCTTCGCCATGTGCTATTACTACTCTTGCTTGTAAAGTCCTCTAAATGAGTGTTGAGTCTACTAACAGCACGTCAACAATCTCTTTCTTCATATACTATACCGTCCGGTTTGATATCCGAAACAAGAGATATGACACAAGAGAGAGAAAGATAGATATTCCCCTGATAGGAAGAAGACAGAGTGCAGTTAGCAACTCAGAACACAGATTCGGATTAGTGCAAATAGAACTAGCTAAATTCAAGCGGTGAGTAATCCGAGTCCTTCTTGCTTGAAAGGAGTTGGCTTCTCTAGAAGCCTACTAATAATAGTATGAGGTTAAGTACCAGCTAGCATTCCGGAGATTGCCGGGGGTATTTGACCGCGGCGGGATAGAGTCTTCCTTCTACGATTCCGAACGGCTGCTAATGCCAAACCAAGAGAGCTACTAGCTAGGCAACAGGCGCTACCGGTGCTAGAAGCGCTTAATGGCAGGAAAGACTCAGGTTGTGGCATTTTTTTATTGAGATTAGATTAGTGATTAGTCACACCTACCGTAACCTATATTGGTAAGGTTAAGCAGCTGACTTAGCCCCCGTCTTCTTAAGGAGGTCTTTCTCACAGGACGGAGATAGGTTATACAATTATGACTTATTCAAGAGGTCCTCCCTTCCCTACTGGAGCGCTAACTCCTATTTTTTTGTAGAGAATCCTTGTGTAGTGTATTCTACTGGTATAGAATCTTTGTGCGCTGACTCCTACGAATATACCGAACTAGTCCATCCATTTTGATAAGATTCTTCTGCTGCCGCTTCAAACACTCGAACACCTCTCTACTTACCGATCCACTCTTCTCCTTCACCGCCTTCCCTAGCATCTATGTTAGCCGCTTCTCTCAGGCACAGCAACTACGTACCACCAATATATTCTCATTTCGTTCCTGCTGGGCGTATTTCAGTGTCTCAAAAGAGAATTGCTTCTATCAAGATAGCCTTGTAAGAAGTCCTGCTCCAAGCAGCTAAGCTTCTCCTTGAAGAAGACAAGATGAGAATCCTTTCAATTGCTTATCAATTGCTTATTCTCTCCGTTGATTACTATTTTCTTGATCCGCTTCTGAATAGAAGAGATTTCACCTACTTTCATTCACTTCCTTATAGGAGGTCGGGAAAGTTAGCTTCCTACAAGAAAGGATTGCTTCTAGTTCCGTAACACTCATTGATTGGGAATGGAATGGGAATGCTGATTAGGGGGAATAAGAATACATTGACCGGAGGCGTTCCTCGAATCTTAAGCTTTCTGTTCTGGGAGGTTTACAAGTAGTGAATGAGATTGCATTGGATCGGACAGCTTTTAGGCGCTAGGAGGGCTTTCAAGCTTTGATGTAACCGTTTGTTGGTTGTTGGAATTGATGAGCCAAACAAAGCCGTTCTCAGCCTTCTCCTTCTATAGTATCCGTTTGAATACGAGACAAAGGAATCTATAGGCCTATCTCTCACTTTCTAATACTACTTATTGCTAGCTAAGGAGCTAGAGTATACTAGTCTAGAAAAGACTCCCACATATATAGCTGGCTGTCTGACTCTAATACCAATGCGAAAAAACTAATGAATTCATTGATGAGGGCGAAGAAACTTTCAGCTTTAGAGAATCCAGTTCCAGCGGAAGACTCAGTACGATAGCAAGTCAAATCAATCTCCTCCAACAGAAGGCTCTGACCTGACCACAGTCAATCAGTCTATTGTTTTGGTTCTCGAGAGTCTAACCGAGAAGAAATCGTCTCATCGCTTTGAGCCTGGTTTAGGTGTGGCGCACCCGCCCATCTAGTAGCCCAGCCCTTCCTCCAAGAGTAGTTATCGCAAGGAACAATGGGCATCCCTGCCAGGAAAACCAAGAGTAGAGTGACGCGAAGGACCTGACGCAACTCTACTACCGCCCTTCCTACCAAAAAGCTAACCCAACCGAAGGAAATGACATAAGGTCTGGTAAGGGCTATAAAGAATCAAAATCTTTCCTCCCTTTCGTTCTACTTAGGTGGAGCAATCCGAACTCTCGACAGAATAGAAAAGAGGTTTTGATTCCTGTGTAGACACCTCAACGAACTCATGTGGACACTCCTCAGCCCGAGGACCTCAAATACACATTAAGATGTTGACCCGTTTTTCTTTTCTTTGCTGATTCCTCTCAATGAAATTTGCCATGTTGCACTAAGTTACTTACGGATGTATGCATGCAGTCCGGGAACACTTTGGGGTGAACACCCATCCGAACGAGTAGAGTCAATAGTTCAGCATTTAGGCTGTAACATTTAGCAACAAAAAAAGTCTTTAACCCAACAAGTGCTCTCCGAACCAAGCTAGATAGTCTCCTATCACTAGGCTCACCAACCTACCTGGACTTTGATTCTTATTATTCCTACCGGATATCAAAACCATAAGGATTGTTTCCAGCCATGAGTTCCCATATGACATAAGCGCTATTGGGTGGGCCATTCCATCAAATCGTTGAGAAGGGGCCCCCAATCTCGTATTTGATGGAATGGCGTGGCGATAGACTTCGCTTCTACGACTGCCTCCCCAGCCGTTGCAAACACTGAGCGAGAACGGTAAAGGGCGCACAAACTATGTCGTTGCGCGGGGATTCACCAAGCTGGGGCAAACTAAGCCGTTCGTGGTTGGTGGGGCGGATTAGGAATGCGAAGGCCTTTACTTCGAAAGGAGACCCGCCCACTATTACCACGAAAAAAGCCCTGCCCTTTTCCTTCGCTACTAGGAAAGTAAGCAACCTCTATTAGCGCCGGACCTTGAGTCGAATTGATCGTGTCATGTGCAACGTCCATCAATGATGGTTCATTAATGTCCATAGATTTAGACTCCTTCCCCCTTCCCTACTACGAAAAAGATCGAGAGGAGCCCGAACCAAACCAAGAACGAAAACGGAAGCCTTTCGATTCGCTCCCCATTCTCTCTTAAATAAAGCTCGCCCTCGAGCCCTGGGCAGGTCGGCCGGGTCTTTCCCTGTTGTTCTGTTCCTGCCACGAGAAAGACGCACGGAAGAGGTATGCCCAGCGCGCGGAGGATCTGTTAAGAGTTTCTCTTGTCTCGGTAGGGAACTGTACGAGCTTTTCCCCTATTATATTGAATCAATAAAAAAAGAGGTCAGTGCTACGGCCCCTATTGCTTGTTTTGTTTGATCCAATATTGACCGGGGACGAGCCCCGACTTCCATAGGTCCTGGGTTTGACCTCCCGTAGTGGTCCTTGCTTTTTATAAAGCGGAGCGGGGCCAATTTCTCGTACTTGCTCCGTGTGCCCCCCTTTCGTCGAGTGCCCACTGGACTGTTGGCCTACCAAGTACTTGCCTGCTGCTCCTGCCGCCCGCTTGACGGGCGGAGCACTCGTTATCCTTACGGTTCTGTTCTCTCTGCCGGGGCCCCTCCCATTGCTCTAGCCATAAGCTATGGCAGCTCCGGCTCGCAACCACGGGGGCCCGCCCCGCGGGGCCAGAGTGGGCTCAGCAGTCAGCCTTCATTCGAATGGAGCTAGGGGGTCTTTCGCTTTTGCCGGATCTAGAGAGATACTACGAGTCCTCCGTCCCAGATTCCATCGCCGCGGCCATCTGGTTCACCGGTACTACCAAAAAGTCTCATGCTTACATTACTGTTATTGATGGTTTGATTATTTTGGACTACGAAGACCTCGGTCGTGCTTCTGGTTTCCATTCCCATTAGAATATTGATGATAAATCTCCTCGTGCTCTGCCATAAGAACTTGGAGTCCGTATCATGGTGAAAGTAAGGTAACGCTGTGATTCTTGCTCAAAAAACAGACCGAACGCGTTCGAGTTATCGGCTCGTCCGACCCGGCAGCATTCATGAGTCGCTCGTTCAACTGTCCCTCGGAGACAGGGTCGAGAAGTACCATGCATGGTTGCCCTCTGTCCTTTATTTCTCTCGGTCCCACCCAGAAAGAGACGGCTTAGCAAAAAAAAAAAGTTAGCGCCCCTGCGCGAGCCTTTATTAGTAAAGTCAAGCTTTGGCTCCCTAAAGACTCAAAAAATGAATCAAAAAAAGGGGGACTTATGCAACGGGCTATGCCACCCAAACCAACTGAGGGAAGTCGCATCCGTCCCATCGCAAGCACCTACAATGTCATGATCACATAGGTTTACCCTTTTTCTTTGGTAGTTCAACGGACGGTCGCCCCTCCAACAAGAAAAGGTATAAATATGGAAACTTCTCTGCCATTTGAATCGGAGAATTTATGGAGGAAATCGGGTTTTAAATTTCCAGAAACCACACGATTATATAGCCAAAGGGAATAGGCCGCACCTAAAATCATCCCAAGCGCTGCTAATGTGGCTACTAAGCTATTTCTTTGGAAAGCTCCTACTAAGATGGGAAATTCCCCGATAAAGCTGCTAGTACCAGGTGAACTCATATTGGCCAAAGTAAAAGAAAAGAAAATGGTAGAGAGATTCGGCATGGTGCTCACTAAACCTCCGTAATATCTAACAAGTCGAGTCTTATGTCGGTCATATAGAACACCAACACATAGAAAAAGGGCTGAAGGAACCAGTCCATGACTTAACATCGGTAGAATACTACCTCCAATTCCCTGTATGTTCGATAGAGCGAAAGATTTCTCCCGGAGTACGCCGATTACCCCCCGAACCGTACAAGATAGTTACCACCTATCATACGGCTTTCTAACATCACTTATTTTTCTGGTCGTTCCGCTCTGTCGATCGATGGTAGTATAAGAGATCTGTAACCCCCCCCGAAATTATTTACATAAGGGTTCCTGCTTCCTACCCATAGTTAGCATGTATCTCCCCGGGTCATACTTGAGTATCACATCGTAGACCCCCGCCTAACTCTATCTTCCTTATCAGTGAACCGGAACTTAGTGCGTAGGTACTCTTCAATGCAGCGTGGACGAGACGACGTCACATACTACGATCACGTACAAAAGTCTTGCCCTTCAGCTCGGCAATATGGAACCTATCACTCCCGCCGTTCCTTTATGAGGTCTTATCGGGATATAGGTAGGCCCAAGCCTTTCCTTTCCCACCGACCGAGCGGTAGTTCCTCACGGCTGACAAATAGGAGTGACGGCCGTAAAAGAAGGGCACCGGCCCCATTCCCCCCTCCCCCCATCCTCTTTCTTTCCTTCTCGAGAAAGAGAAAGAAGAGAAGAAAGGATTGATTCTCTTCTTTCATGTGAGAACGGCCCTTTCTTGTATCGAAAGGTTTTTCGTGCTATACACCACGTTGAGAAAGTCCAACCTCCTATGGTACCGTACCTTTTTTTTTTTTTACCTCGAAAGGTCTGCAAGCCTTATGATGCTACAGTCGGCTGTCCGCAGTGCAAGGGGTAAACTCGGACTCGGATGGGATAGGATTGTGCGTTCGGGCCCTTCGGGTGTCTCATATTTTGGCCGAGTTTACCGTACCCCCCGCCCTTATGTTAATGTTAGGCGACCGTAATCTTTTGTTACGTTCCACCGCTGTTACGCCAGAAAGAAAAGGTTCCACACGAGCTCTCGTTCTGCGGCGTGGTGGCAGGACCAATAGGGGATTGGCTCCGGGATAGGGGTTCCTCCGCAGGGGTCATGCAAATACATAGGCCCCTTCCCTTATCTTTTGGATAAATGGGGTGCTTTCCGATCTACGGTCCCTCGGAGCGAAGGGTTAGGCAGGTAGTATGGGCCCTCTGACTTCCAGCTATGTGCTGTGCGGCTCCCGCGAAGCGAATGAAGGGAAGCTCTTCGAGCTTACGGGTGAGCTTACGTTTACTCTCTGCCTCTTTGATTGATAGGCGGGCGGTCAAAGCCCCCTACTTAAGATTCCATTCATAAGGGTAATTTTTTGTTGTCGTGACGCTTTGGTTAGGCCGACTTATAAGGCAAGTTCTAGCTTCTATAGTGGCCCTTCCATTTCCACTTTGTTGTAGTTTGTATTTTGACTGAATGAATATATCAAACCAAACAAAGGCGAGCAGTATAAGCCCTTCTCTGGCCCTGGGAAAAGCAGCGAACTAAGGTTTTGAACCCTTGCAACTATGATAGAAAGCCGTTTGCTTGTTGCCAAACCCCTTCGCAATCAAAGTAGGCCGCGACTCTTGTATTTTAGTCGGGCGGGGGAAGCTACCGCTTATACGACAGCTCCGCTTCCTCGTCTTGCTTCTGGCAAAGCTTCTACTTTGCTTGCGCGAAAGTGCTTTTCGCTAGGTCAAAAAGCTTCCGGCAAAGCGAAAGAAAAGATCTGATCCAACAGAAATCCGCATATTGAGCGCAGCTGATATGCGGCTACAGCTAGGCGATCATATCATGCCATAAAAGACTTTGATATGTATAAAGAGATCCCCTATATATACAGATAGAATAGATGTTTATGGACAGACATTCCATTGATTCTGAGTTTTGGGGCTGAAAAAGCTCGTCGATCCAAACAAATGAATCATTCTTCTGGTATCTCTGCGCCCCCCGCCCCGAAACTGACCCACAGCACAGCGCCCATTCGCTTTGCGCGCGGGGAGGGGTTCCTCCAGCTTCGCTGAAGAAGCTAGGTTCCTCCGTGAAGTTCATGAGACCGCGGCGGAGTGGAGCAGCCGCGACACTCTTTTTCCTTAGCTGGATCTCGCTGGTGCCACCTAAACGGTAAGTAGGCGGCGGATGTGTGACGTTTGGAGTTGTCGTTCGTGCACCTGTCCCCAATGGAAGAATGAGTGATCCGTTCCCCTGCAGATCATGGCCTTACCACTCGGTCCCCGATGGCCAGCGGGGTATTCGGTATAGCAGCTCACGTTCGTTTGCGCCTTTCCGCAGGACTGGGCACATGTTAGCTGTAGGAAGTATGGTTCCGAAAGTGACTTCAGTTCGCCAGTTCAGGCTCAACTCCTCGCTTTACGTTAGCGGACCTATGGCTCGGGCCGGGGCTCCGCGCTCTTTCTTTCTGTGTGGGACGATGCCGGGGAGAGAAGGGAATGCGTCGAGGCGGCGAACAACAACAAGACAACTACATTTTGGCTTTTCCCTCCATGAGATGAGCCCAGTGCATTGGACCGATGGATAAGAGAACTGAGCTGGCCCAAAAAGCGCTGGGGCGCTCTACGTACGATGTAGACTCCATCAGATACATATCGATTTCTTTCTGATGGATCAAGAATAGATAGTTGTCTCATCAATAGATAGAAATAGGCTCAAAGACCTTATTATTGATTCCCTCTCTATTCTCTATCTATTTCTACTCTTTAGATCTATCAGAACAGATCAGGCTATCTATCAATCGATTTGAAAATAGCACGTTCATCTCGCTTTTCGTTCATTTTCGCCACGCCAACATAGCCGCCATAATAAGAAGAAGCAGGCGAAGCAGCTAGAAGCGAGCACTTCTAGCCCTTTGATTTTGATTTACGAATTAATTGGGAAAGCCAACAGAAAGATTCGATTCAGACTTCGTGGAGCCGGTGCTGCTGTTGCCGTCTAACGACCGTCCCCCCCACTCCCGTCCCGGGGCGCTAAGGGACTGAAGGGAACAGACGGCAGTGTTTTGCTGACGCCTCGAATCAAGCTTTTGTTGCGACATGCTATGTTTTCTCCCCCATTGCTAGTAGGTTGATGGGTCGCACGCCCGACACACATGTTTTGGCCTTGTGTGTCCATAACTCAAAATAGGTGACCTAACGGCCGCCGCCCGACTAAACATACCAATAGTCACCAGATTCATATGGGCTACTGAGGAGTAAGCAATGATCTTCTTTAGATCGATCTGTCTTGAAGTGGTCAAGGAAGTATATATTATAGCAATCGCGCTTAAAGTATAAATGAAAGGAGTAGAACAAAGTGTCGCTTCGGGAAACATGGGTATTGAAAATCTTAAAAACCCGTGGGTTCCAAATTTTAAAGGAATTCCTGCCAAGATGACGGATCCTGCCGTAGGTGCCTCTACATGAGCTTCGGGTAACCAAATATGAACTGGTACCATAGGCACTTTGACGGCGAAAGAGGCGAAAGAAGCAATCCATAGAAAGATTTGGCGCCGCTCACTAAATTCTGTGGTTAATGATATTTGTAAATCGGTGGTTCCTGTTTGGAAAAGAATCAACAGAATAGCTAATAGCATAAAAAGAGATCCAAGTAAAGTATAAAGGAAAAACTGATATGCTGCCTTGATCTTTCTTTGTCTCGAACCCCATACTCCTATAATAATGGTAGAGTAAGGGGTGGCCCCAAAGCGGAATTGACCGGGGGTGCTTGGTCGAAGCTCCAGTAGGTAGCCACTCCCTTCTCAGGGAACCGTACGTGAGACTTCCGCATCATACGGCTCCGTCCCGAGCTTCCGTCGTCGGCCCTTGTCATTAGACCACTATCTATGCATGTATTTTTAGCCTGGACTCTCTAATCTTTTACTTTTCGGGTGGTGGCGAACTATGCAGCTTTCGTTGCGGGAGATGCCCGCCCGTAGGGCCCGGCCCCTGCTTCTCGCCCGAAAGAACCGCTCACTAGCTAGCCGGACTAGTGGGGCGCCCTATCTGGAGACATACTGAAAACCATACCTTTCGAACCGAACGCAACGCCCGTCTTCAAAATCTAAAGATAAATGGGCTCGTTAAGAAGAAAGATGGAGTGCGGCCTGTAGAGCACATGTAACGCACAGTCGGGTTGCTCACGCAGCGGATCTCTCTTTCTTATAGTACGAGAGAAAGAGAGATGTGAAAGTAATAGCCTTACCTTATGTTATGGCCGCCCCCCCGACTTACGGCCTTTACGCTACTACTGTGATGTGAGCGGTTCTTTCGGGTTTGATCTTTCCCAATTATCCTGGCCGGAACTTATACGCAGCACTTATACGGAGGTGCATGCATAAAGGTTTGGAACTCTTTTTTTATCTGAGAATCTGAAGGGCAGGACCCTATTCTCGAACCCTCTGCCGAGCTTAACCCTGCCCGAGGAACGCCTTTTGATTTGACGGGGGCCAAAGAAATGTCTCCACCAGCTGTCAACAGTCTTTCTTCGGAATTCTACTGAACGGGTCGATCCTCTGCTCCCCTTTGTTTTAGCAACTTATCCTAAGGTCTCCTCTCCTTTCAGTCGAGTGACAAAAGTACCTCGCCAAGAGCTATCCGGCGACGACAGAGGAACCAACCCGCTTGCTGTGGCGGGGCGGCTTTTTTTGTTTCACAATAAGACCTGGACGATTATCCCTAACCTCGGTAGCTTACGAGTTTACGTCCATCCCTGGTCGGGTACAGTTTCCTTTTGATTTCTCCCTTGTAGCCGTTACCTGAATTCGCGCAAGTGTGTCCACACCCCCCAAACAGACTTGACGAGGAATCCATTCTCGCGAACAAACACAACCCCTACACACACCTAGGAGCACCCCTTCCTGCATATCCATACAAGACCCGAGTAGGCGGGTCGAGGTCCTACGAGGTACCCACTACTCGGAGTACCCTCCCAAAAGGAAAAAGATGTGTTTGTCAGGTTCGGTTCTTCTTAGTTGGATTGGGCGGGTTCGACCAGAAATGCTATGCTTACACACAAGACTACCCCTCTTCCCGAAAGCTTCGCGGGGACTACTTTACGACGACGGACGACCGCCCGTAGGGGGTTTACTGCACAAGGCCCCTGCAGAGGAAAAGCTTTCTCCCAGCGAATATAAGATACTCCGCTCCGCACAACATAGGGATTGGCACGCTTTCGGGAAAAACATAGAATAGTAGAAGATCCAGCATGCGGAACACGGCGATCATTAGAAATTCACGAATTAAAAATGCTGTAATATACTCTTTCCCATAACTTCTCATACCAGACCAACCCACTGAAATGCAAATAGGGATCAGAAATGTGGTCAATATCACGAAGAATAAAGAGATACCGTCTATACCCAAATAAAAATTGATGTTTTCATAAGGAAGCCATCGAAGGCTTTCCACAAATTGAGATTTGGCCGTAGAAGAGTCGAATTGTATCCGAGGAACAGGAGAATACAAAAAAGTAATAAGAGAGGCACACAGACCAATTAATCGTATCGGTCGTATTCTTGAATTTGGAATGAAAAGAGGAGTAATGCTTCCTAGCACAGGACACAGAATAAGACCACTTAGATTAGAATAGCATTCACAGAAATGTTCTAACATAGAGTAGAATCGAACATTGAAGAGATTCGTTGACAACAGTCAAAAGATGGGCGCTAAATTCCAATACAATAGGGGTCTATTAGTTCAAGTCAGCTGAACACCGTGATTTATGAGTGGGTAGGTGGGTTAGGCGCGCCTCTCGCCCACTAAGAAGTAATGAGGCTAGTCTCCCCCTGGAAGTAGTGGTGCCCCCTTCCCGTGTATGCGCCTTTATTTATATTCTTTCTATTTTTGCATTCTTTATCCAATGGATAATCTTTCTAAATCAATCTCTCGGGCAAAACGCAATTCGTCGATTTCAATCCCAAGGTGCACCTTGCCTTTTTTCAGGTAGCTTTGTTACGCCTATTCAGCTATTGGTGCATTGGTCATAGCCGAAATTCGTCGAAGAAAATGAAGTTTTGTCTTTCTTTCAATCCAAATAAGAGAACAAAGAAACCATTAAAAAAGAAATCCAGGATGACAGCTTTCAAAGAGTAAGAGACAGGGAGGGGGAGGCTTTCGAAACCAAACGAGACTAGAAGGAACATGGGAATTAGCACCATTCCTATGAGTGTTTTTGTCTTGAAGAGCCAACAAAGGGAGGCGCTATTGCCCTCATCAATGAATTAATGAGTTTCTTCGCCCTCTTTCTCTATCTCGGTCTCGGTTTAGCATCATCATATATCGATCCGTATAAAATTTGGATTGGTTGTAGCGCGGGAGCAGCTACTCCATGCTGTGGCTGTGGGGGTGGGGGCAGCCTAAGCCTGATAGAGGCAGAAGCCGGGGCCACCGGAGCTATCTGCGTCGAGTGTGTCGATTGAAAGTTTTGGGGAGACAACTCAAATTGCCAGCGCAAAAATCGAAAGAGTCGTGCCGTTCAAAGTGGAATTCTTCTAAGATCCATCTATTGCTCGATTTTGCATGCTCTGCTCCCCCCCCAAGAGAGACTTGTTCTCAAAATGAAGGAAAAGGGAAATCGACGGTTGGTTGTTGACCAACGGAAAGGCATGGGAGTTGGAATGTAAAAAAATCAGAATAAATGGGATTTGATCATTCAGCGCGGTGCAGCCCATATAAGGTAAGGAGCTTCAGCTGTTCACGTTACAGCTACTTTGTTGGCTCTTACTATACTACGATATTTTCATTGATCGTAGCTAATTCTGATTCCATTGCGAAACGAAAACCGCTCCTTCTGTAACAACTGATTCTACACAAGAGCAAAAGAGCCTTTTTCTTCCTAAGGATCTATTCATTTTTCCATTAACAGTCGAACCAACCGGAGAGAGATTGCTTTTATCACAGACCAGAAAGCCAGGAGCGAAGCCTGCTTGACCAACGGGAGAAGCCAACTCGGTAAAGCTAGTTTACGGTTCCTTTTCTTGCTTGACGGTACGCTTTTAACCAAGCCTAATCCATATAAGAAAAGCAAACAACTATGAAAACAGGGCAAGAAGATGCAGACGGACGGTACGCAATGAGACTGGAGTACCTGGCAGGGGGCCTAAACAGACAGTAAGGGGGTGGGTCTAGGAGAAGAGAGAAGAACGAGGGTGAAACGCACTTTAGTGACTAGAGAAAGAAGTTCTCTAGGAGAAGCAGTCATTGAAAAAATAAGAAAAGAAGGAGCTGATCCGATGGATGAAGATAACGATCAACGGAAACTATCGGCTATGAAGCTAGATGGCATAGAAGAAGGTTATGCCAATATGGATGGCTTGCCTGGAAACTAGCCATGTATGTACTGGCTTCCGCACTACTACTTGGTCGGATCAATCTCACTCTCGAAATTGGGAAGAATCAACTCAAAATGTGAGGGAAGCACGAAGCATAACATTTGAATGAGGGGCCCTCATCAATGAATTAATGAGTTTCTTCGCCCTCCGAAGGACTCGTTTTCAGCTCCTGGAGGAACCTAGCTTCTTCAGCGAAGCTGGAGGAGAACCCCTTATATATTATATTCAAAAAAAAGTCAAAAAATCAAATGATTACACAGCCCACTTCGCTCCGCTGCTCTGCTCGCTCCGACGATTCTACATACCGGCCGAAAGAGACTGAGCCCGGGCGAAGCCAATCACATTGAGTTGTAGATTGACATAGTTAACCTTCAGTGCACTTATATATAATATATAGTCAGAGTTGAAGCTGAGCGTTCACCTTAGCGGCACCTCTGACCTCAGATGCATGTGTTAAGCATATAACTAGCGAGCGAACCATACTTCGGATATCGCCCGGAGCAAGGCCAGTCGCTGGATTAAGACTAACAGGACCATTCCTTATGGAAGACCCAGCAAGAGACGTCACTACCTACCTAGGCCTCAGGATGAACTCCGGTGTTGAGGCCCGAAGACTGGAAATTCAACCCTAATAACAAAGTTCCTCTCCAACCAGTCGAGTTACTACGTTCCTTGGTTCACTTCAGTAAATTCATTAACACCGTATTTGATTCCATCTTGTTTTTCTGTGCTAGTTACTATCTGTAGTAGTGGACTAACTGGGCTGGGCACAACTCATTTGAAAAGGAATGTCAAAGCATTGCGGATTCTTCCTTCGACGTCTTATTGCCCTTGATATTAAAAGAGGTGCAAGCCTTACTTACCTATACCTTTGATATCTTCAGCTCTCACGGATATTCGCCTGCTTTGTGCCTCTGGTGGCCCTTGACTGACCCGTCTCGTATCGTTTAGATAGTAAGTCCATGGGTTCTACAATCTTTTGTTAACCTTTAGGGCTCCACGGAGAAAGTTGTCGGATAGCAGCGATTCCTATTGCTATTCTATGGTCTTTTCTGTCTTTTTCCCTTTACCCGGTCCTATCTCGAGCAGGATCACTCTGCTCTTTGGTGCTCGGGATTCCCATTCAAGAACCAGTAACTCACCGTCTGATTCACGCGGATCAGAGCCATCGAGTCGATAATAACTGTTCTTCGGTGCAGCTCTGACCCTACAGCAAGATCACGCCGCCGTTAACTAGATAGTTCAGAGGACGAGCCTCTTAATTCGATCTTATTATTTGATTATTAAGCCCGCTTTTGCGTTGCCGAATTCTTCTTGTATTTGCCATCAGGTGAAGGAAGGAGGAACCCCTTTGCCATAGACCAATTACCTGAGATTGGCTAGCATGAGGCGTTCCTCGGAGGTTAACCCTAGTTGATTCTGCACTCCCTAAAGAGAACAATCCATTCCTTTAGCATAGCTAACGAATAAGGAAAACTGAACTACGGCGCTCACCGAATGTATCACTCATCCCGCTCCTAAGGTAAGGAGTCCTCTGTGTGTTATAATCTTTATTGGAATTCATTGATAGTTACTTTGCCAGGTTCTAGGGGGGGCTACTCTTCTTTTTTTTCTCGATCGAGCCGCTCTCCCTCATTCCACTCGTCCAGCCCTCTTCACGAACTTGTACAATCGATGCCACAAAAATAGCCAACTCTATTATCAAAGAAATAAGGAAACGGGCGACGATTTGGCACCAGATATCCGGAGGTGTGGAAAGAGCAGCTGTGAGAAGCGGAAAAACCATCAAAAAACGACGATTGTTCGTGAAGGTTTCCAAAGAAAGACCCCTTGGTTCTGGCAAACAGATCACAATTACAGGTACCTGGGAGCATACCGATGGAATGAACGAAATACGAACAGTTAACATAATATGGTCATAGATCTTAGGTTGTAACTTGATCATGAGCGAATTTGTTGATGTTGCACCCACGAAGTATGGAAAGTGCCAAACATTGGGAACGACCCGGGGAGGAGTTAGGAACAGGAACAAGAAGAAGCGAGAACCACTTAAATGGAGGAATCGATTGTATTTCGTCCTTTGTTCTCCATAGCAACTGGGGATCAAAAAGCACCAAATTTGATAACTTATTAAAGGAAAGACGAAATAAGAGCATGCTATTGAAGACGTTGCAATAAATGTCGAAAAGGCCTCCGTTAATTGTGTACAAACAAAATACGAGTCAAAAGGCAGGGTAAGAAAGGGTGACGCTAATGGAGATATTAACTCTTCCGGGAACCAGTAACACGTAAACCATGTCAAACCAAGACCGATCAATATCCGAACGGAACGGATTCGAACTTCTCCTAGAATCGTTTCCGATGCGAAATGAAATTCATAGGATATATAAGTAATTCAAAGCTAAAAGTGAAGGATTTTATAGGGTTCATGTACCCATGTTCCTATACGTATATCGGCTAATGGTATGCAATTTCCTATTTGATAATTTATCTCAAGTATTTCGTATCTATAAGCAGGGGGCGTGGCCAAGAAGCCTACACTCCAGCGGACTGCCCCGTTCTTCACACAGTGAATAAGGGCTTAGGATGGTCATTCTGGGCGGATTGCGAGAAGGAGCTGGTCGAAGGTTTGGACCAATCGCAATTCATCACCATTTTGCCTGCTTCTAATTGATGACTGGCTATTATATAAGTGTTGACCTAAGCCCCTGTGCAGGGGCTCGGCTCCCGAACCGTACGTGAGCTGCCTCGTACGGCTCTTCCTAAGAACTTGAAGCCCCCTCTCCAGGAATCAAACTTTTTTAAAAAGCCTTCGCCCTCCGGGGCTATTAGAGAAGCAGCTTCGTTTCTTGACTTCTTTGCTCAGTCAAGCTTCCTTGTTCCTTAGTGTAGTCTCGGTCCATAGTTTAAGACTCCGTTCCTTATAGTATAGTCTATATCCACAGCTGACGGTGACTCCGTACCGACGCCTTTCCCTTTGTAGACGGCTAAGTGACTTCGTCACTTTAACGTACTTTTTTTCTTACTCTATCATAGGCCTTCGTCGGGCTGGCTTTCATCCCCAAGCCTATAGGCGAAGGCTTGGTTTCGATATCGCTCATGACTTGTTATAGAGTCCGTGTAGTGGTCGGCCTTCCCATCAGAAATGGGAAATGATAGAGTGGTCGGCCTTTTGCTTGCCTCCTTCCAGCTCAGAATGCCTAATGCCTATTATAAGTTCTAGAAGCTAACCGCCAGAAGCTCACCCTTCGGGTCTCGCTTCCAGCGCAGGAGGCCAAGCATTCTGCCAGACGTCCCGCCTTGGGAGCCCCGTTCGCCTTTGTTCAGTAGATCTTCAAAAAAAATAAAGATTTCAATGCAACCTTAACTACAACTACATTACGCAAGCTCCACCAATACCTACCTATCGAGTCAAAAAAAGTACCAGTGACGGTGACTTTCTAGGTTTATGGATTCAGTCAGCGAAAGTCCTCAATATCAACAAGATGTCGTGACCGGTGTAGCTAAGTTTCAAAAGGTGAACAGCGCCCTTTATAGTCGTAAAATGCTTCTCTGAAAAGTAAGGAAGGAGGCAAGCAAATGAAGGGAGGCATTACGGGCCGACTACAAGAAGGAAAGCTTCGTAGACTCGACAAACCGCTTCTATAATGCCGACTACTAAGTAAAGACTTTCCCCTTCCCTTATTAAATAAAGTCAAGGAGCGAAGCCTGCTTGACCAACGGGAGAAGCCAACTCCGTTCCTCCAGCTTCGCTGAAGAAGCTAGGTTCCTCCGAAGCGAAGCTTAGCGAGCTGGCCGATCACTACATAAGCCGCTGGCAGAGAAAGCTCGAGGAGAAATATTCATTCGTTGCTAAGCCAAGGTCCCAGGTCTCCGAGTCAGCCCGCGCTTTTTCGAAGAATCCTGCACCCATGGGCATACGGCCTGGCAGGCCTTCCCTTTCCGCCGGAGCTTCATGGGTGTTGCCCCGTTCCCCAATCAGATATGATATTTGGATGTGAGCTATACTCCGCGAGGAGCCAAACGGGCGTATGGCCTTGCCATTGGACCTCTCTTCCCGTGTGACTAGAAATGTTCAGTGACAACCTCTCAATTGTCATTGCCTGGCCTCTCTTGCTACCGCGGTAGCACCTAGTGTGGTCAGCACCAATCGTGTTCGGGCAACGAAGCTTACACTCATTCACATTGGTTCATCCTGCTATGCCCCGGGCCTTTTGCTCTCCGTAAAAAAAGGTGGCCGGCCTTTCATCAAAGCCCAGGAATCCGCTGGACATCTCAGCGGCGGGATTTGATACCCGCATCTGATCGAAGTTTCATTTTATTAGTGCCCCCAGATAAAGGAGAGCTCTTTCTAGGTGGGTCGCTTCGCGCAAGACATTGCTTAGGACCAACGGGTCACACGACAGGTGCACGATCGACTTTGCACGCTCCATCCTTCGCCCTCAAACCCCGGAGAGCTTTGAGTTGCTTCGCCCTTTCGCCTATCGGCTTGGCAGGCAAGCTACCTTGATCCGTCTTCGGCTTCGATTTGTTATGCCCAGCAGCTCCAACAAGCCCTAAAGTATCTTGCCGCCTAAAACTCTGCCAAGAAAGCCCTGCTTTACCTTTGATCCTATGTGCCCAGAGAATGCTATGCGTTCTCCACTTTCGGACCTCGCAAAGAGAGAACGTGCTTTTTCCTCTCACTTTCTGTCTCATTCGCGGAGCGAAGAAAGCGGTAGGAACCCCAGCTACCGCTATCCTTGGGAAACCAAAAGAGCTAGCGAAGGCAAGGGATGCAGTCTCTCTCTTGGCCTTTGGAGAGGAGAAGGCAGAGAAGAAGACGTCCTTCACTAAAGTTTTTGTGCTTCCTGCGCCCTTACTAGCAAAGGCGCTCTTATACGAAGACAAAGGAGGCATTCCGGTAGGAAGGCCGACCACTACATAAGCGGCCATCAGTCCAGGAGAGAAGCAAGCTACCTTTCTTTGATCCACCTTCCCGGGCAGGAAAGAGAACGAAAAGAGGCCGCTGATGGTGTTCGTGGTAGGTTCGAGGATCTTGCGCGGCGGAGCTAACTCCTCGATCGTGTTCATTTTTTTCTGGCAGCCCCCCTTGATCCATCGTACTGGAGCGATCTGAGAAGAACGATTAGGGTCATATTCTATACTCTCTACAATGCCCATAGAGGAAGTGCTTCGTTTCAGATCAATTCTTCGCAGCAATCGCTTCGAGCCACCCCCTCGGTGAAAAACCGTAATACGCCCTGAGGAATTCCTACCAGCAGACTTTCCTGTACTCAAAGTGAATTGTCTAAGTGCTCTTGCTCTCCCTGGTCTCATTGTCTATCTCGTAATCATTTGATTCCGTCTGACTCCTCCTACTCCTCCTTCTCCTCTTTCATCGTCGTTAGTCCTTTTGACATAAGATTCTCGGCCACCTCTGGTCCGGGTGCTCAACTTGACTTTTAGTAGATCGTGACTATGATTTTAGATCGCTGAGTTATTTAAGCAATTCTTTCTATATTCTATATATTATTATTTATATTATTTTTGAGTCTTTCTTTTGATAAAAAAAAATGGATTCCGAACAGAAGAAGACAAGACTTCTTCCTGTATTAGTAGTGAAGGAAGAATTAGTGGTTGGTTTGATTGTTGACCAACAAAAGCATGGGAGAAAACCAAGAAAAAGGGGGCATAGAGATTTCTTCTCTTATGAGATTGATAGTTTGATCGCGGGGGCGGCCCGGCAGGCTAGCGATCAGAAAAGAAAATCGATTCTATATACGTTATGGTATGGAAAAAGATCTGACTTCGTTAGAAGAAGATGAGCTGCCTAGATCTTTGTATGTCCAGGTCTCGGTAATTGAAGAAAGAATGCCCAGGTGATTAAGCCCGGTAAGACCCCGTGATAAGACATCTTCCGCAATACAAAATAACAACGGGGGATAAAGGATCCCCCTGTCTCACACCGCCTAACATGAGAAATATCCTTTTGGAGAACCATTAACCAAGACAGAGAGCTTGGCAGCGTCGAGGTGCTTTAGCAACTCGACTGAAAGGAGAGGAACCACACCCATGACTAGCTTGTTTCTGCTCGTTCGTTCGCTTGTAGTTTGCTACGCTGTTTTGCGAGTCATTCCATTCTCTTCCCTTAAACTACTATGTGAGGATCGGGGGGTCGTTACGAGATCCCCTTCTTCTTATCTTAATAGGAACGTAGGGCGTGTTATGGCTTGAAATCCTTTCGAAAAGGTCAGGGCGAAGGGTATCCAACCAAGCAACAAGGAAAATTACGAGAAGCAGAACAAAAGAAGATAGGGGATGGGATTCTAATGGATATTATCCTGTAGGGTGGGCATCCAAGACAAGATTGAATTCGTTGTCAAATCGTTCCAGCCAGGCATCCGTGCAGGTGTCACAGGTTCCTCCTGAAAGGCTGCTCTCTCCCCTAGCGAGGAACCGGAACCAACTAATGGATCCATGGAACCCCATATATTGCATCTCCAACGGAAGGTCAGATGCTCCATCCGAGGGAAGAGAAGCCATCTCCGAGACCATTCCAGAGAATGAACTCATCCCTTTCCTTTTATCGAGACCCCATCTCCTGCAGCCAGAGAAGCGTAGGTGCAGCAGCTCTCTAGCTGCATTGAACCCTCGAAGTTCATACTGTCCTGACCCATACCCATGAGTCATTATCTCCCTAAAGAGATTGGATAAGAGTCACTAAGATAAGAATTCTGTCTGACCATATGATATGATTTAACGAAAAGAGGTTTCTAGTAAGTGTGCCTCTAATTGGCATATCTCTCTGCTGTCGATTAGATACCTTCCTTGCCCTGCTTAGGGCTATGTGATAGCACCCAAAAAGGGGACTGATTCTATTTGAACAAGACCACAGGAACCATAGTCACACGGTCAAGCGAACCAAAGCCAAAGCGAAATCTCTTTCCGGCCTTTGAAGGCGCGTAGCGGGCTCTATACCCAAATCCTTCTAGTGTACGAGCGCTGCTGCTTCTTGGCCTCGCTCTTGCTTTGCAAAGATGGGTCGCAGCCTGGCACTCTCAGTCCCAGAACTAACAATCTCAGAACTCAGAATCAAAGAATCCCAGAACTTGGAATTCAAGAACTAGGGAGATAAGTGGTGAGTTGGGAAGGAATGTTAGGAACCTAGACCCTTCCCTTACCCAGACCCAGAACTAGGAATCTTATAACTAGGAATGTGAGTAACCCTGACCCTAAGCCAGAGAAGTTGTTCTGGACGAGAGAGTAGGAATAGGTACCTGCTTCAACCATAGAAAGTCAACAGAGGGGTGATCCTGCTTAGGTTTGAAAACCTAACTCGGAATGACGAGGTTAAGAAGAGGAAGATAGAAGGGAAGGGGTAGCTGATTAACCAACTGTAATACAAAAGGTACGATGATCCTCCTAGGCTTGCAGACCTACTCGGAACTGTGGAAAGAAAGCAAAGATATTTAAAAACGGGGCTTGCGCCCCTGCTTTAGCTGCACTTGCAGAGGTAGCTACAGTCGCGAGCAGGTCCTTGCTCGCTCCCTCCAAGTGTTGGTTAGCTACGATAGAACACGGATAATAAACAGATAATTGCAGTGAAGGAAGAATGACAGAGAAATACGTATATGATAAGAAAGCTGCTTACAGGAGTTAGCCAGCTCACTTCCCTCGTTCGAATAGGGAGATAGATAAAAGAAACGAAACTCTATAAAATATCATTAAGAGAAGAAGATTTTCATTCCAGCTTAAATAAGTAAGACTTGACTCTTTGTTCACTTTGTTTTCTCGCTCCTTGCTGGCGAAGAAGCGGCATATCAAAAAATAAAGAAAGAAGCAAAATCTCATTCAATTTGAAATATAAGAGATCTCTATGCCCCCTGTTCTTGGTTTTCTCCCATGCTTTTGTTGGTCAACAACCAACCACAACTTTCTATAGTTCTTCACTACTCCTAGAGGCTTGACGGAGTGAAGCTGTCTGGAGGGAATCATTTTGTTGAAATCAATTAATCTAATCATGTCTCAACTGGGTGACTCCATTTCACTAGGCATTTTAGGGGCGTTAATCTTTTCCTCACTCGGACTCGGACTAGTGGGTTATAAATTCTTTTTATTTTAAAAAGAGATTCCTACAACTACCTTGCTGTTTAAAATGAAAAAGCAACAGCAAGGTAGTTGTAGGAATCTCTTTTGGAAGTGGAATTCCTACTCTCATCTCTTTTTACTAAATATAAGGAACAACACCCTTATGATGACATTCCGTCTCTAACGGGTATAAATATACCTAAAGTTTTAAATCTTTTTTTTTATTATTGTTGATACTCCTGCAGAGAATCTCTTAGGAGCGAACGCGATCTATTTAGACCTGATCTATCAGGGCACGAATAGTCGCTACTTTGCCCAAGCTCTGGAGTTAGTGCTTTCGTTCAGTTCATAAGGTTGACTTTTTTTTTCTAAAGCGGTATTCCTCCTTGCCTCTGAATTCAAAAATGGAATTATGGAATTATCTCCCAGAGCTGCGGAACTAACGAATCTATTCGAAAGTCGAATTAGGAACTTTTACGCGAATTTTCAAGTGGATGAGATCGGTCGAGTGGTCTCAGTTGGAGATGGGATTGCACAAGTTTATGGATTGAACGAGATTCAAGCTGGGGAAATGGTTCTTTTTGCCAACGGTGTGAAAGGAATGGCCTTGAATCTTGAGAATGAGAATGTCGGGATTGTTGTCTTTGGTGGTGATACCGCTATAAAAGAAGGAGATCTTGTCAAGCGCACTGGATCTATTGTGGATGTTCCCGCGGGAAAGGCTATGCTAGGGCGTGTGGTCGACGCGATGGGAGTACCTATTGATGGAAGAGGGGCTCTAAGCGATCACGAGCAAAGACGTGTCGAAGTGAAAGCCCCTGGGATTCTTGAACGTAAATCAGTGCACGAGCCTATGCAAACAGGGTTAAAAGCGGTAGATAGCCTGGTTCCTATAGGCCGTGGTCAACGAGAACTTCTAATCGGGGACCGACAAACTGGAAAAACGACTATTGCTATCGATACCATATTAAACCAAAAGCAAATAAACTCAAGGGCCACCTCTGAGAGTGAGACAATGTATTGTGTCTATGTAGCGATTGGACAGAAACGCTCGACTGTGGGACAATTAATTCAAACTCTTGAAGAAGCGAATGCTTTGGAATATTCCATTCTTGTAGCAGCCACCGCTTCGGATCCTGCTCCTCTGCAATTTTTGGCCCCATATTCCGGGTGTGCCATGGGGGAATATTTCCGCGATAATGGAATGCACGCATTAATAATCTATGATGATCTTAGTAAACAGGCGGTGGCATATCGACAAATGTCATTATTGTTACGCCGACCACCAGGCCGTGAGGCTTTCCCAGGTGATGTTTTCTATTTACATTCCCGTCTCTTAGAAAGAGCGGCTAAACGATCGGACCAGACAGGTGCAGGTAGCTTGACCGCCTTACCCGTCATTGAAACACAAGCTGGAGACGTATCGGCCTATATTCCCACCAATGTGATCTCCATTACTGATGGACAAATCTGTTTGGAAACAGAGCTCTTTTATCGCGGAATTAGACCTGCTATTAACGTCGGCTTATCTGTCAGTCGCGTCGGGTCTGCCGCTCAGTTGAAAGCTATGAAACAAGTATGCGGTAGTTCAAAACTGGAATTGGCACAATATCGCGAAGTGGCCGCCTTTGCTCAATTTGGCTCAGACCTTGATGCTGCGACTCAGGCATTACTCAATAGAGGTGCAAGGCTGACAGAAGTACCGAAACAACCACAATATGCACCACTTCCAATTGAAAAACAAATACTAGTCATTTATGCAGCTGTCAATGGATTCTGTGATCGAATGCCACTAGACAGAATCTCTCAATATGAGAAAGCCATTCCAAATAGTGTCAAACCTGAATTACTACAAGCCCTTAAAGGTGGATTAACTAACGAAAGAAAAATGGAACCAGATGCTTTCTTAAAAGAAAGAGCTTTAGCTTTAATTTAGCTTTCATCACATTAGGAATCCTCAATCAGTAAGAAAGAAAAGTTCTTAAGCAAAGACTGGAGAGGCCCCCGGTCGAGATGTAGTAAGTAGGTCTCCAATACTGGGAGACTGAGGAGAATGGGAGTTTGTGGGTTGAGGGTGGCATGCTCCCCAGGGCCCTCTTTTTTTTAGATAGGTAGGGTAATGCATTTCAGTATGAATTTGATCTAGCAGTGTAAACCTGAGATTTTCAAGAGTTGGCTTCCGCTGATTTTGATTATTAAGCGTAACGAGACTAAAAGAGTCGTGAAGCGAGTCGGAAAGAAAGAGGCGAATCTACAAGAGTCTTATTTTTTATATGTTAACGGGCGGAGATTAAGAGGTAGGCAAGTTGGTAGGCTCCGGAGAATAGAATGCAATGGAGGGCCTAACGCCTAGTAAGACGGGGAAGGAGTGTTATGAAAGGGAGGAAGAGGAGGAACCTAGCTTCTTCAGCGAAGCTGGAGGAACCCCTCTTATACAAGTCGAATCTCTTTCTCTCTCTCTTTGCTCCAAGCCAACTGGTAATTGGCCACATCCTCTTGTTAGCGATGCTCTTGATTTCCGAGTTGTTTGCTAGGCTAGTTGATCAAGGATTTGTGCTCTCTGAGCTAGGAGTTTTCTTTCTTGGCTTTGTGGACCCTTCTAAACCAGCTTTAGTAGCTCGGGGAACAAACAGGTGTCTTTGCCCCGCTCACACAGATTTGATCCTGGCTCAGAAGATCCGAATAACCGACGACAAACAATAACAACAAACGAATGGAGCTGCAAAAGATCAGGAATAAAAGGAAGATTACAGCGAAGGAAGAGACGAATAGTATGATGTACGGTAAGGGATAGCTGTTTAACCAACTGTAATGCAATAGAAGGTTGATCCTCCATGGTTTGCAAACCAACTCGGAACGAAGAAGAGAGAGAGGAGAACGAAAAGGGACAGAGAAACAACAGTTGATGAAAGGTGGGATTGAACACACTAGAAAGGATGTTTCTCGTTGGGGTTGAACCAACTCTCAGGCCAAGAGAACATACCTCGCCTAATAGACTGGAGGAGGTGTCCCGTACCCTTAGAATCGTCTAATAAAAAAAAATGTGCTTACCTTCATGAGAGCTGACATTACTTCTTTTAATATCGATGCCTTAGTCAGGCTAACTATATGCGATGCGTCAAACATAAAATGAAATTCATCCTACCGAAACCAGAGAAAAGAGGATCTCCGGAGCTTAACCATCGAATCGGGAAAGCACTCTTTCCAGCTTAAAACAGAGCACAGAAAGGACACTCAAAGCAGGTCCGTCTGTATTTCATTATCGTAGAGATCCCCTTCTCTTGATCAATTAGACAAGAACTTTACTTTGTTAAAGACATAAAAGGAGAGGTCCCACTATGACTATTAGCGGTTCCTAGCGGGCATGGGAGAAGAGCAGGAAGAGAAAGAAAAAAGAGACAAAGATAGACTAGCCACTTCCTTATTATACGAAAAGTTTTTGATTGATGAGTTCTCACCTTCTCTCATGGAGTAGGTAGATGAGACTAAAGGGGCGTAGGGGTCACTCCGCTACGCCCCTTTAGTTGAACTGGTTGCTATATAGGTCAATTGAATCTTAGCCAGTTTCTTCTTACTCCTTTTGTTCTCATTCCCGGCCAACCGTGCCATGAAGAGTCGAGCAAGAGCAATCGCAGCTTTATCTCTGCCTTTGCAGCTCCCATTCCTGCCTTATAAAAGCTTCAATCTACAAAAGTAGAACTACTGATGACAGGTCTGGCCTACAACTGACTTGCTTTCAGACTTACTGGCTGAAGAGCAAAGAGATATCTATTTTACAGTAAATTCCTCTTAGAATAGCATCCCGCTCTTTATAGGTTTAGTAATGCTACCTTGAGCCCAACGAGTGTAATACCTGGAGCGAGTAATAGAGAGGACAGAAGATATAGACTTTTTCTTTCCTTTCTGGCTTGACCATGAGGCCATGACTATTCAAATCTTACAGGAAGCAACCGCTGTACCCCGCCTTCTAGCTCTTAGACTTTTAGAAAGGGGTTCCTCCGGCGCCTTGTTCCGTTAGTTTACTTTTCATTTTCAGTCTTGTAAGTTAGTTATGTCTTTCAAGGGGTATCACCATCCCCCGCACCTCTTGTTGGTGGAAGCGCGGGGCGTTGCAACCATCATCCTTGGACCCGGAATATAGAGCTCTTCCCTGGTACCTGGAGGATGGAGAAGAGAAGGCATTGTCTTTCCTTGATGGAAAACTCTATGGTATAGCTATCATTAACGGAGGAAATTCTCCTTCCGCACCTTGATCTTTCTAGGGCTATTTTCACTAAGCCGAATCTGTGGACTGAGTGGCTAACTATTCTAGGAGTGGGGCTCTTCTTTGTATCGAGAAAATCTTTTGTTAACAGTTCAAACTCCCTGGCACCATCCCCTGAATAAGGGGCTGGGGGAGGTTTGACACCCTCCCAGATCACTTGAGCACAAAGCTTTAAAAGGAGCAATAGTGGTCCTGCTAACGAGGTACTATGCCCGGTTAGTGGACTTTCCTTACCAAAGTGGCAACACAGAGGTTCAGTCAGAAAGGGTACTAGTCTCTTAGTTAGCCAGTCAAGGTGTCAAACGAGCAAAGCAAGGGCTAGGTCAAGGTCAAAGAAAAAAAGGGGGTTCTTTCTCGTAAAGCACTCTTGCTATCATTCCTCGCCTTACTACCAAGCGTAATAGCTAAAGAGCTAAGAGTGCTAGTGTCATTCCGTCTAGCGATAGTGGGCCAGTAGTACCGGTATGTATCTATTGTACCAGTAGTTGCGATTTTCATTCATAATTTAATCGTCCTATGCATTTCCAGTTCTTTTCTTTCTCCCTTTACGCGAGTTGAAGTTTAAGTTGAAGATTTCCTTCCCTTCCTGCCGAACTCTCTCTGAGTTCCCTGCCAGCTAATGCTAAAAGGCAGTCCTAATCTACTTCTTTTTCTCTTCCAGCTAGTTCCGGTTCTCTTGATTGAAGATGGGTTGATATGGGTTCATGCGAGCTCCTAGTCATTTCAAGGTACCTTGGATGGGGTCAGAGCTAACAGCTATGGAATTCCCGGGGCTGGTAGCAGTCTCAATTCTCGTATGCTTAACACGTGGTGGAGCTAAGGATTTCATACCTTCTTTCTTTTCTATCTTTCTTCTCGCCGACCAAGGCGAGGCGCTCTTAAGCCGTTTCAGGAAGGGAAGGGGCCATCTACTACAAAATCATACCTTTCGGACGTTGCCACCGCTCAATCCACCCCTGCAGAAAGTTGGTATTCAAGAGACGGAAACTATGTCAAGAAGGTGGAACTAGGAATAAAAACAGGGATCCACGTCGAGCCATCCTGACGGACGAGCAGCATCAATTGAATCGGCAGACACAAAGACGAAGACAGAGACGAGCTAACATGTAAAGTAGTGGAATGGAAAAGGATGGTAGCCCACCCAGGACAGCACATAGAGTAAGGTTGGCTCTATGCGAGAGAGGAAAACCGCATGGACACGGCTCCTTTTGGATAGATCGTCAAGCAATTTACCCATATAGTTATTATCCCGAGGTTGAAATACAATCCATTACATCTGGATTGAAACTTGAGAACCTCGAGAACCAAAGATATAAACTGAAAGATAGAAGCTATCTAACAATTTGAATATGAATAGGAACAGCCTTCATAGAGAAGAGACATGGACAATGTATCCTTAACAGTATGGCAATCTCTTCCTCTTTCGATTGGCAAAGAAGCAAAAATAGTAAAGCGATAAAAGAAGCAACTCCTTGAGCGGCTCTATCAAGGAGCAGAGGCAAAGGATCTTCTCCAGTGGTCATTAGTAATGCACTAAGTGGTCCTTATTGGGTGCTGGCGAAGCATAAGAGGAGATTCTTAACGTCAGGAATATCGGCCTTAGCATCTTTCTTATAAAAAATGATCTTTCTCTACTTGAGAAGGTTCTTACCAGGCATACTACTCATCGATTATTTTGCCCTTTGGGATCGAAACAACCTATTAAAGGTTGTCGCCTACATAACCTACCTCCCAGACTAAGGGAAGAAGGTCTCAGATCACTGTAGTTCGAGCCCTTTTGTTAAAGCAGTTCCTGTACTTTGAATGAATTCAAGCTAGTAAAGTAGTCTGCTTGAAGGGGCCCTCCTCACCACTCCCCTTCCCCTTATATCATGGAAGCAAGCCAAACGAAAGCAAACTAAGCATGATACAAGGGCCTGCCTTTCGGTACGTGAAAACGGCAGTTACACTCTTTCACTGTCTTACTTTGGCCCCTCTCTAGCCAAATCAAAAATAGGAACACCCCCTGAGACACCGAAAAAATGCTATATAACAGATAGACCATGTCAGCCTCCACCTTCATCACTAAGCAACAAAAATGACGCGATCCTTACTGTCCATCTTTCTATAAGCCAACTCCAAAGTTTAAACCTATCCATATTCTTGTCATTTCTTTTCCTCGCCGCACACAAATAGAAGAATTCCCAATAAAAGAAGAAAGAGGTCGAGTCCCTCGATCCACCCTTCTAGCCATTCCAGGTTTGGAAAGTAAGGAAGAAAAAAGAGCAATGCTCCAAAGAGTAAGGGGACCAAAGGTTGTCGTTCCGCCCGGATATTCCTTTGAAAAAGGAATAAACCCCAAAAAATAAATAAATGACAGGCTAGAAAAACAAAGGGAATGGGGCATGATATGTTCTCTCCTCCCGGCGCCGAAGCTATCGAAAAAACCCCTATACCAGTACTCGATAGAAAATAAATGAAAAACGGAAAAAAAAAAGCAAGTTTTCCTCGTCTCTTAAAGCAAAGGAAAATGATGCAAGCACCCGACACCATGGCGGTTAGCCGCGCCGGTTCGCGCAGAAAATCTTCACCATAATAGCCTTTTCCTAGAAACCAAAAATAAACACTCAGAATGATAAAGGGACAAAGGGATTTTTGGGGGCGTTCCTCCAAAAAGAAGCAAAAAAGATAGTATGTTATTAGCTGACAAAGAAGAAAAAAGGCCCATTGCGCGAGGTCCCTTTTCCAATGAATAGAAGATAAGAATCGCACAAGGAAGAACGTCCGATGGAAAAAATCAAATTAATAAACAAAAAAGAGGAAACATAATGAAGACAATACCCTCGATACTTACGACGTTTTTTAGAAACAGAGTCGATACGACAGAAAAAAAAAAGGTTAGTAATAAAAGCAATTTTGCCTCTTTTTCTTCTTTAGTTGTAAAGAGGCGAAAATTCAAAAGAAACTGATAAAGTTGTAATACAACTTGCAAAGCCCTTGTATAATTCTCTTCCTCGAAATCGGGAAAGATGTGTAAATAAAAAATGGAAAAAAAGACATAGAAAGATGTCAGGTATCCCACCTTTACAAAGACCAAGAATCAATTATTATTCATTAAATAGAACGAGGAAACTGAATGATAAGAATGCCTCTGAGATTCAATCATAAACCACTTTGTCTCGGTTGTATGTAAACCCCCACCCCTTCACCCCCCAACGGGCTCTTTCTCTTTTGGGGGTCTAATTTTCTTTCTATCTGACAGGACAAACTAATAGGAAGGGATGGTTCTTTCATTGCATTGATAGAAGTCTAACTAGAAAAGGATCTCTCTATTACTTTGAGAAGAGAATCGTTGGTTTGACCGACGGAAAGCATGGGAGAAAGAAAGATGCACAAACAGACGAAAAGAAAGAAGAGAATGAGTCACAAGATAAAATGAAAATGAGAGAACGACGGGGAGAGTGATGCAGAATGAAACCCTTACTTAGTGCGGGGACAGGATTCGAACCTGTAATCTTCAGGTCATGAGCCTGATGAGTTGACCAATTCCTCTACCCCGCTTCTTCCCCGAGGTATTTATTACTTCTGCTCATAAGGCTTTCAGACCTCAACTAGTGCTTTGGTTCAGAATCTGAACATAGCGCCCTTACTTAATTCATTTCGAGAAAGATCACTCCACAAAGCAGCCTTCTTCTTATATACGTATTATTCTATCAATCAATAAGCACGGGTGGGGTTCCGTTCCGTAGTCGAACTCGAGGTGAGACTGATCTCGTAGTCAGCTAGTGCGCTTATCAAAGTAAATCTGTCGTGCTTGAGGGGAGGCACGGAGTGATTTGTTCAACGGATTTCCCTTCATAAACTGACTTGATTGATGCTACGAATGAAAGCCTTTGGGATATAGCACAGAGAAAGAAGAACCTATTCTATTACAAATGCAGTTATGTACAAGGATAGGTGATAGGTTGGTCCATAGTAGAGTAGCAATGACAACTGCTACTAGGAGAGCTACCTTCTCAGACCAGGGGAGCTCAATAACTAGCGTCTGAACGCCAGAGACTATTGAAAGACCGATACCGGGATAACTAGATAGGGCAGCACTTAGACCAAGGACTTACTCAAAGAAGGGCGTTCCTAGGGACCACCCACTCCCGTTCCTTGGTTGTTGGTGTATTGGCTCTTTGGTTCCCGGTCAAAGCAGGTCGTTCACCGTGAAAGCAAGATAGGGTGAATCTGTATCGTAATAGGGGTATGAGTATAGTATATGTACAAAGAGGTAATTTACTCCTTCGACTGGTCCTTGTTTGGTTTAATGAATGATGTCCGTGAAATCAAAAAATTATAGAGGAAGATGAAATACCAGCTGATTCCCCTATTGATTAACCTCCACCCAAATATTAGCAACGCGGATAAGATCCCAGCTCCTTCTCCAACGAAAGAGATTTATTAACTAGACAGGCAAAGACATCGTCATTCTATTCTATAGCATAGGGAAGCATGGAATCGGACGCTAGCTTCTCTTAAATGGTGCTTAACACATGTAGATTCCCTGACTTTATTCATCTATGGGATTCCAACTTAAGAGAAAAACTGTCGGAGCTGCGCCCTTTCCCTTCTCGTCGACGGAAGTGAGTGTTCCTAAGGCGGGAAAAGGGAAGAATAATATTCTAAAGGTCTTTAAACTTAGAATTGTGTAAAGAATAGTTCTTTCTTTCGAAGCGAGCCCCATACCTACTACGGCCAGGAATTGATGCACAGAATCCGCATTGAAGGAATTACCGGCTTTCCTAAGCTTGGCACAGATGTAATGTAATAAGCTCTTTTCCCAGCGATAAAGAGAAAGATAATGTGCATTTTTCTTACTGACTCTTATCTAGTGCGCCTAGGACTAATTCCACTCACGGCATTAGAGGTCTTACCCGCTACCCTTTCAAAAAACTTTGTGGGATCACACCCGTCAATCTCCGATGAATCAAGTAAGGGGGGTAGAATCCGCTGCTTGTATGGATGGTATCGAATCAGCTGTAGGAGGAATATCCGCTGTGGGACTTCACAAGCTCATGCCATAAAAAGTAAGCTCTTTCGGAAGAGAAGGAGTTGCATATATTATATAAAGGCTGTTAGCAAGTCAATTCCTTTACCTAGGGAGAAGGGCCATTTCGCCTAGTAGGAGTACTAGTCTTAGCATGTTAGCAATTTGAATTGGACAGCTTTCGCTTTCTATCTAAGCTGGAATGTAGCCTTTCACTAATAGACATCGTACATCTTAGCTAGGGAAGCTTCTTTGTCTAGCTAATCTTCGGGACTGTAAGCAAAGGATGTCACTATAACTAACCTTCCCAAGTAACAGTACCTAATTTGACCAATTTTGATTTTATAAGAAAACAAGGCGTTCCTCGACTAGCCTATTCCCAATTCCCAAGCCAACCAAACCAGGGAAGCTAGGTCCAACCATTACCCTTTTCGAGCAGCTCTTTCAACTGCCAATCAATCCCCAGAATAAAGCCAAGGTAGGGAAGGGATCGGATGGCCTGACTGGCTCAACTAATTAGTAGAGGAGGTTGCTTTAGCAACTCGACCACTCGGAAGAGGAACGAAGGTACTCAGCCTCCTGGGGCAAGTGGGAGCGACACACTGAATGAACCAACTCCAATGGAGCAGGATCAAGAAGAAGTGAAGCTACGTAAAAGTGAGCGTGGTAGAATCTCTCGTCGTCGATTTGAGATTGAGGGAGATCCGGGGAATCTTATTCTGTCACCCAAAACGACCACTCCTTCTGTCGGAAAAAGGACTTGCCCTATATTGAAATGAAATCGAAACGAATGGAACGCGACAGAGCACTCCCTATCATCAGGTAGTGCGCGCCATTCAGAACTATGATATCGTCTTCTAGTCTATTCGGCAGGTCCAAGTTGTTTTGGTCTTATAAGCTCAGGTTCTTGAGTTTTGGGAATTTCCTTCCATTGGCTGGTTCAAATTCAATACGGATGGTGCTTCCAAGAGGAACCTTTTCTTGCATGGTGCTGCAACGGGACATTCTGGTAAGTCGGGTGCTGGTGGCCTTCTCCGAGACTGTTCAGGAACATGGATCTATGGGTATACCTGCAAAATAGCTTTCTCTACGAGCCTACAAGCTTCACTTTGGTTTAGCTTCCAACTAAGGCTAAGGGAGTTGTTTCCCCGGGATTGAGTGAACGGGTTCCTCCTCCACCCGTACCACAAAAGCGCTAATTTATATCAAAAAGACCGGGAAAACGTGAATCTATCCCGAACATTTCCTAAAATACTGGATAAACTGACTCAGATCGGTCTCCCTCAATCGAGAGGCTCGCTTGCTTCACTTCAACTTCATAGAAGAGGGAAGGATCTATACACTAATAGGCGGCCTACTGGCGTACCTCGAAGTACTCCGGTATATACACCAGGGCAACTTTGAAAGACAAGTTAGCAACCGTACTGCTGGTATGAATTGAGTATTGCAGACTAGTGCTAAGCCCATAGCAACAAGACTGCTATTATCAGATAGCTAGGACTTATTATACAGCTCTTTAACACAAGGGAACGTATGGGGATATCTCAAGAGAAAGTTTCTAGTTCTGACTTTCTGCCTTAAAAGCAGATTCAAAGAGACAAGATCCAAATCCTTCAGCCTATTCACAGCGGATGAAGTAATAATAGTTTTGTACAATTAGAACTTCATACCTTTTTCTTGCTTTCTTGTTCTTAAGCCAGTTACATACTTTATTAGCCCGAGGGGTTCCTCCTTTTAGTCGAGTATGTAAACAACCTGAAACTAATGCTTTTTTCCCTTTCTAGCTTTCCACCATTAAGACTTGTTGACTTCCTACGCTATTCGATTCGCTGGCATGTCCGGTCTCATACGAAGGAAAGGTTGAATGGTCAGTCACTCATGAATTCCTTCTCTAATCACTACTGGAATATAGCTTATAGAAAGAGCAGCTTGAAGGAAGTGGCAATGCTAGAAGGGCATTTCTTTCCCGCCTACTTGAGTAGCGCGTTGGATCTATGCTTAGGCAGCTTAATCGCGACTTCATATACGTTGCCATGTCTGTATCCGACCAAAGACCTTCCTGATGTTCTTAATAGGCATTCTGAGAATAGGTTGCTGGCGTAGCTTCTGTTCCTATGGTTGAATGTGTAGGGATGGAGTACCTGCCGGGGAAGCCTGTCGTAGACGGCTGGCAGGTACGACTGAGTGGCTAGCCGGGCTGCTTCTTCTGCGCATTACTCAAGATTCTTCCTGAATCAACGAGTAGCCATCAGCTGAGAAAGATTGATACCAAGTCGGAGCGTATCCCGTTCGAGGGTCCACCTATTCGGTGTCCAGTACGAGAGTCAATTCCCGGGAAAGCTCAATAACTGTATTTTAGAGTACAAGTCAGACGATGCAAACGCAACGCTATACCTAGAGTTTGATCTAAAGTCAAGCTACCATCCGATTTCAAACCGTGAGTACTCTGTTTCACTCTCATTCCGCCTTTGACTGAGCTTTCGGGTTCCTCTTTGCCCACGGATTGCCATTTAGATTATGCCATTTTTCCGGTCTTTCATGCCCGGGAAAATGAACAGTTGCACTTTTCTGTCATATCGGATATATTCAAAAACTCTCCTCTTTGTCTACGCTCGTTCCTGCCTTATGTGAAGCTCCCCCTGTTCTGGAGAAAAGGGGATAGGAAGCCAATAGGGCAGGCAAGAGTGGCATAGGTCGCCATAACCATCCAGCTCAAGGAATATCACATAGAATAGCTAAGGTGCGAGTCTTCTATTCAATATGGGTAGGTACTTTCCCACGGAAGAGCTCTGCTATCACCTAAAAGACTGCAATTAGCCTATCGGATAGAACTCTTCCTTCCTATTTCCCCATTCGAACGAGGGAAGTGAGCCCGTCTTGCATCGTAAGCTGTCACTATTTCTTCCTTGGAGCAAAGCAAGTGAAGCAAGTCCAGTGAATAAAGGCGCGTAGCGGGGTTGCTTCGTTTCCACCAGGAAAACTTCGAAGTTTGTTCCGCTGTTGTGGGAATAGAATACTGGCAGGGGTAGAAGCCATCTTCTTTATTTATTCAATTCTCGCGTGGTAAAGACTATAAAAGTCATCAATCGTGCCATTTGATCCTTTCTTCGGTATTTATGGTACAATACCTCTCTCGCAGCTAATTCAATAGCTTTTTTTGCTAATGAACCCCAGAAGTCGAGCAAGAACTTGCATCTCATTCCATGAAGTGAGAAGCTCTCTTTATCTTCTCCGTCTTTATGCTTTAGTTTGTTTTAACACCGGTGGGCAAAGAAGGAAAAGACTGAGATTCATTCCCATACGGAGGTTACTAACATAACAACCCGCCTTATCCTTTCCTTTGAACAGAATTATGGGGCGCGGATGCCCTTCCTGGCTAAAAGAATGGCTGTATAATAGAAAATGAAAATCTGGGCTATCGATCTTGATTGAGTAATGACTACACATTGACTGCGGAAAAGACTACGGTTTTTCCTTTTCAGGTACTACTATCCATCCGTCGAAATCGAAGCAGTTCTCGGGAGCTTGAAGCAAAGCAAGCAGCGAAGGAACGGCTGCTTTTCTTTCAATACTAGCTACTAGTGAACACTCTTCTTGTTCACCTTTCAGGTATAGTTGAAATCATGTAAGTTAGTTAGCCTTAGCCTCTCTGTTCTCCTTTCGAAGCGGATTAGAAAGCGCAGCAGCTTAAACAAAGCCGCACAATCAAAGGTGAATTCCTCTCGTATATCAGAATAGGGTGACAATTCCATATTTGAAAAAGACCCCCCACCATTATGAGTACCCTTTGACTATTGTTATCACAACTTTATTTCCCGCGTTTTAGTACGCTACATCGGTGTAAAAGATTGAATTGCAGGGTACAACCTTTCCTCCTTACTCTGGCCTGCTTGCTTTCTTTACCCCGGGCCCTTTCTCTTCTATTCTATGGAACACATTTCAAGACTGACTGACCCACTAGAGGGAAGAATTCATCCTATTGACCGAAGGCTAAAGGACTCCTATTCTTTTTTCACAATCGCCGATCAGGTGTTAGGAATCAATTGCAGCGAGAAGCCTCTCTTGGCCAAGAAGCAAGAGTTCCGGTGCGGGAGTAATACAATCTGTCTATTAGTGAAATCAAAAAAAAGAAATATGAATCTCGTATAAATGAAGGGAAATTTTATGGCTTAAAGGAGCTTTTAAGCCACTCTTCTAATTCTTCTTGGGTTATTTCCGATCGGATCTTGCCAGGGGTTGGTGCATTGATGCCGAGAATACGCTGTTTGAAAGGTAACTTGATCTAGATGCGGAGTCTCGTTTGCACTTTATGGTACAACTTATTATCCTTTAAATAAGAGGAGGTGGTCAAGACCTTAGGCAGCTTGACGCATCTAGTTTCGGTCTTGCTAACGGGCGACAAAAGGATGTTGGCCTTCCCATTTGAAGCGTAGAAGGGGTCTCACAGAGATCTACCCGGCCTTGCCAGCCAGTCTATCCAGCAGTCTACTGAATTGTCCATCTTTGGTATTCTGCAAGCTTCTGGGGCAGGCCGCCTCTTATTTCATCGTATATATAAAATGGAATATATAGTCTCGTTGGGGGTTGATCGCTAGTTTTTGAATCCACAAAAACCTCCTATATACCTACCTCGGCATCTTTCATTGAAAAATGCATATGGTAAGTAAGAATAAGAATGAACGGAAGGCGTTCCTCGGGCTTCTAACGAGTTCCTCCTCTACACATCCACTCCTTTATGCCTTCTTTCCAAAGATTCATTCCTTTGTTCACAACAATTGCCCGCAACAAGTTTCCCTCGTAGTTGGATGATCTAACTTCGAGTACTTGTGTTAAGCAAAGCACAATCAAGATAAGAAGATCAGCTTTTTCCAATAGTCAGATAAGATCTTAGACACCGTTGACCTGCTTCACGGGATCGCCTATAAGGACCTGATCCACTCATGGCAAAGCTTATTTGGGCGGGGCTTTATTGATCCAATTTCTCACTTTTGAGAGTCAACCTGATCAACCATCATATCTTTTTTTACTTAATTCAACACAAAGCACCAGTCTTAAGAGAAGCAGAGAAGCAACCTTCCTTGGCTCACTTAGAGGGAACTGACCGGAAGCAACTAACTGACCAGCAGTAAGCAACCCTACCTGTACCTTAGCAGGCTTCGCTCCTCTGGTTGTGAGAAAAGGTATTGCATTGCCCTTCTCAATCCGACAGAGAGCTCATCCCTGTCTCACTCACCTGGAACCCATCCTAGGTAAGCAAGACTTGATTTCCTTTCATTTTCTTTTCTTCCAAGTGGTTTCGTTTAGTACGAGATGGCTCCACACCTCGCGGTGCTTCCACCTCTCGCCTATCGAAGTTCTGTTCTAAAACCTGTTCCGAGAACTTGTATAGAGAAGGATTTCCCGCTAAGCAGCAGTTCTTCCATACCAACTTAGCTTCCCGGCGCTGCTATTGTCATAACAACCGGTACACCATAGGTTGGCCCAACCCAGTCCTCTCGTACTAGGGTTGGCTCCTCGCAGTTCTCGCTTTAACACCAACGGTAGATAGGAACCGAACTGTCTCACGACGTTCTAAACCCAACTCACGTACCACTTGAATCGGCGAACAACCGAACCCTTGGGACCTTCTTCAACCCCAGGATGTGATGAGTCGACATCGAGGTGCCAAACGACTCCGTCGATAAGAGCTCTTGGGAGTCATCAGCCTGTTATCCCCGGCGTACCTTTGATCCGTTGAGCGAGAGCCCTTCCACACGGGACTCCCGGATCACTATGGCCGACTTTCGTCTCTGTTCGACCAGTCGGTCTCACAGTCAGGCAGGCTTATACCATTACGCTCACGAGCAGAATCTTTGCTTGAGCCTACCTTCGCACACCTCCGTTACTCTTTAGGAGGCATCCGCCCCAGATAAACTACCCACCTCGCAGTGTCCCGCCTCCCCCCGAATTCTCGGTGCGGCGGTTAGGCACCCTTAGACGAAAGAGTGGTCTTTCAGGATTGGTCTTTCTATGTGAGGACCTCCCACCTATCCTACACATTCGATCAAGGTTGTCACTGCGAAGCTATAGTGAAGGTGCACGGGGTCTTACCGTCTAGCCGTTGGTACTCCGCATCTTCACGGAGAATTCAATTTCACTGGGTCCATGTCGGAGACAGCGGGGCAGTCGTTACACCATTCGTGCAGGTCGCTACTTATGCGACAAGGAATTTCGCTACCTTAGGACAGTTAGAGTTACTGCCGCCGTTTACCGGGGCTTCCATTCAAAGCTTATAACACTTCTCCTTTTGACTTTCCAGCACCGGGCAGGTGTCAGACTCTATACATCGTGTTACCACTTAGCAGAGTCCTGTGTTTTTAATAAACAGTCGCTACCCCCTGGTATGTGCCGCTTTCCTAATCAAAAGATAGGAGAGCACCCCTTCTCCCGAAGTTACGGGGTCATTTTGCCGAGTTCCTTCGACATGGTTCTCTCAAGCGCCCTAGTATACTCTACTTGTTCACCTGTGTCGGTTTGGGGTACGGTCAGTTCACCGGGAGGATCGCCCTCCCAATTCGAAGTTTTTTCCTGGAAGTTTCAACCTTCTTGACTATGACAAGAGTCGCGACTATAAACAGACTCGTGACTATGGCAGGTCGGTACGCTCTGCTCTCTCGCGACCCCTACTCTAATCAAAAGACTAAAGGCCCCTACTGAAGTTCGCCAAACTACGACGAGACTTTGGCCTTTTGAAGCGCCAGTAGCGTAGGTCGACCGTGCCAGGCCGAGTCAGAAAGGCTTTGATGACTCAAGGTTCATATTAGGGAAAGGAGAGTGAGGGGTTCCTCCGCTCGACTAAAAGGAGAGTCGTCCCGATCATCCAATTCGCTCCAAGAGAGAGGGATGGTTTTGTAGTCAAAGCAACTTCGTCACTTTCGTGTACCCATCGGACGGCAGCCCTTTCGGGGTTTCCTTAGGGACCGATTCACTCTGCGTAGATTGACTGAACGCAGAAAGCCTTCCACTGGCAGGCGATCTTGTTTTTCACAGGATTTTTCGTTACTCATGTCAGCATTCTCACTTCTGATATCTCCAGGTCTTGTCACCAAAAACCTTCTCCGATTGACAGAACGTTCCGCTACTGACACTTGAAAAGCAGCTTTGCAAGGTCTCGTCGCTTCGGTGAATCACTTGAGCCCTGATACATTTTCGGTGCCATGGAGCTAGACCAGTGAGCTATTACGCTTTCTTCAAAGGATGGCTGCTTCCAAGCCCACCTCCTGGTTGTCATCGCTCGATCACTTCCTTTTCCACTAAGTGATTGCTTAGGGACCTTAGCGTACGATCTGGGCTGTTTCCCTCTCGACTTTGGATCTTAGCACCCCAAAAGTCTGTCTGTACAAACGAGAACGGCCTGTATTCGGAGTTTCCCTGGGGTTGGTAAGGCGAAATGGGGCCACCCTAGCCCATTGAGTGCTCTACCTCGGGCCATCGACATCATACGCTCTACTGAAATAGATTTCGCGGAAAACCAGCTATATCCGATCTTGGTTGGCCTTTCACCCCTAGCCACAAGTCATCCCCGTATTTTGCCACATACGTGGGTTCGGTCCTCCAAGGCCTGTTAGAGCTCTCTTCAACCTGCTCATGGCTAGATCGATCGGTTTCGGGTCAAATAGGAAGAACTAGAAGATTCCACCTTTGGAAAGCGCCTACACCTAATGGCTTAAGCCGCTCTTCCCATTTCCTCGCTGACCCATCATGCAAAAGGTACGCCGTTAGAGTGAGTGCGCTTGACTACTCCTTCGACTGATTGTTTGCATCGGATCTCAGGTTCTCTATTGCACTCCCGTCATAGGGTTCTTTTCACCTTTCCCTCACGGTACTTGTACGCTATCGGTCATTGAGGAATACTTAGGCTTAGAGGGTGGTCCCCCTTTCTCGCGTAAAAGCGATCAGAATTCGAACACGCCGCGTTTTACTGGGAAGGATCGAACCATAGGAACGAATCTACAGGGCTATCACCTTCTTTGGCCAGATCTTCCAACCTTTTCACAATTACAGTTCACAGCGCCCTTTAGGAATCTGAAAGAAGAGGTACGAAGTAACTCGACTGAAAGAAGAGGGGCTTGCTGGTTTTTCCATCATCCAATCCACAAGAAATCGAATGAAACCTGGCGAAAAAGAAGTGAACACTTTGGAACGAAGCTTCGTCTTTCTTTTTCTTCAAATCCCAAATCCGCTCTCGCTCGCCGCTACTAACGGGGTCTCGGTTGATTTCCCTTCCTTTAGCTACTTAGATGTTTCAGTTCGCTAAGTTTTCAAAGTCCAAAGAGCGCAGACTAGCCACGGAGCTTGGATACGGTTTCCCGATCGGAGATCCATGGATCACAGACGGTATCTCCCCATGGCCTTTCGCCTCTGAAAGCGTCCTTCCTTCTCAATGCCCGGGCATCCATCCAATGCATTCTTTTCGATCTTGTACTCAGGGTACACTGAACACCACAAAAATCTCGATGAAACTCTGAGAACTAGAAGCAAGGGAATCAAAAAAGTCTATTCTTGACGTGAACGGCCGCTTTGTCATTAGAGTGTGAGAACCTTAAGGAGAAAGCTTTTTGATTGATTTGTTTACTCAACTCGTAAAGGCTCATCAAACAGCGCTTTCTAGCTTTCTTCAGGATTTAGCCTGAACCGATGCCAGAGCTCTCAGTGGTTTGTGGACTCGAACCACAGTAGGAATTTACAGTTCCGGCTCCCCAGCGGAGCGTAACCACTTTCTTACTCGTAAAGGCAAAGAAAAAAGGGATCCGCCTCGAATCAAAACGTTCTTTCTTTTCTAAAAACGATCTTTCTTCTCTTATGAAATTGATAATTTAAAAAAACGATCTTTCTTCTCTTATGAAATTGATAGTTTGTGAGAGGAATGCAATAACTCGACTATTTACATATTTGATTTCCAGCAACTGATCTTTCTATCTTTCTGCTCCATCCTTCTTCTAAAACTGCTATCCTTTCAGATTGACTCCCTACTCATAAGAATAAAAAGGTCAATCAGACCATTTGTAAAATCTCAGACCATTTGTAAAATCTGTCCTTTGGCTAAACAAAAACACTTACCTTTTCAATCTCATAATAACATTTGTGATGAACCTTTTGATCTGATTCACATTGACATTTTGGGCCCTTTTTCAGTTGAAACCACAGAACAGAAGGTTTTCGATATTTTTGAACCATAATGGATGATCATTCAAGGGCTACTTGGGTCTATCTTCTCAAAAGTCAAAGAGAGTTTCTTCCCGAAGGGGGATGGAAAAATATAGCAACTCGTACTACCTAATATTCCGCTCTAGGCCTAGTGCTACTCGCATTAGATTCAACCATCTGCCTAGATCGGAAAGAGATAGCTCTAAGAGCAGCTTTCCCCGTTCTGACCGGTGCATCCAGAGCAGAGCAACCTCCCCTCTCAGATTTTTTTGAGTTAGCCATAGGTAAGAAAGATGCCCACTCTTTTGACCTGCCTTACCCTTCAAGGCAGTTCTTCCTTATGAAGGTGGGACTGTAAAAGAACCGGTTGAAGTACCAACTCCAACTACTGCTTTTTCTACTGCAGCTACGGCTGCTGATCGATCTAGTCCTTTGATCTAGTCTAATCTTTTTCCCGGTAGCCGGTAGAAGTCTAATGTCCTATAGCCGAAAGGCATTAAGCTAATCATGGTTCCCGCTCTTAGCCGTTGGGAGTCCGATGCGACGGGCAGCGCAGCCAAGCTTGATATCTTCGGTTCTCCTTCTTCTCTCTATTGAATGATTCCTGGGATGGGAGAGAGTAGGTCTCTCGTCATTCTCGGTGAACTGCAGCGTCTTCCCACGATTGGAGAGTTGCCTTATACATTGCTCGAAATAACAATAAGCCGGATTTAATAATCCAATTCCACTTCAGCTGGCCACTTGGATTTCTTCTTGCTTCCCCGCCAACTGCCCTTGCTCATGAAAGAGATAAGTGGCTTTACTGTTTGTTATCTTGTTCTTTGCCATATCTTTCCTTGCTGGGCTAATAAGATTAGGGAAAAATAAGATATAGACTATGTATATCCATTTCTTCCTCTCTTTCTTACTTCGATTCTGGTCGTAGCCCTTATAAGACGGGTAGGATAGGATTAATATCCGTAGTAGAAATCGAGGTTTCAGTGATCTCGTGCTAGTGCCCTTATCATTTCACTAATCAAGAGAAGCGCTGGCTTCTTTAAGTTCAAAAGTGCAGAAAGTGCTCTTCACCCGCTCCGCTCTCTTCACTTGCTTCCCCTCACTTCGTTTGAGGACTTGTTCTGTAGCTTTCTCACCTAAAAGATTAGCTCCTTTATCGCCGAAAAGATGCTTTCGAAATGCATTCCCTATGGACTGCAAAAATATTGACACTCGGGCACCACCATCTGGAAATTTGATGACTGGCATAAGATTCACGTCAAGTTCAGAAAAGTATTATTATCGCTTAGCGCTTGTGCTAAGGTAGCTTTTTCTAAAAAATACCCTACCTTAACCTATTTAGGTGTAGAACCCGTTTAGTGGCTTACAGAGAGATGAAGATTTTTACTTGGTTTAGCTGTCATAGTGGAGCGAAGGGACCCAGACTCCTACGGGAGGCAGCAGTGGGGAATTTTCCGCAATACATTTTTCCGTGGTCGAACGTGTCTTTCTATGGTACGCTCGGTACGCCTTGTTTCTATTGTTCTCCGATAGCTCTATCAGTGATTATTTCAAGTAGTGCTTTTTGTCTGTTACCGGATATGTATAGAAGTTTCCTTATGGTGTTCCTCTCCGGTGTTGCTGAATACACGTCACCGGTTCGACTCCTATTTATATTTAGTGGTCCATTTATTGATTGATGGGCGAATGGAGCCCGGGATGCTGAAAATGTAAAGGCCAAGGCCTCTCTCGGTTGTTGCTTTACCTGTCCTATTAGACTAGCTAGGCCCTCCTTCGGTATGTCTTGTTCCTTGGCTTCTCCTTGTTGGAATTCATGGTTGATTGTAACCTAAGGGTTGTTATATCACTGTCCTTCTGTGGGGGAGAAAGAGGGGGTGTTGCTAGCTCGGTAGGCGCACAGGCGCGGGTTCCGGTTGAAGAGTCAAATTCTCCTTCTTGTTTTTATTGTGAGAATCTTTCTCTATACTCTATATAAGATGCCATCTGTTTTCTTTACTCGTTTCCTCCTTAGCTTTGAATCTATCTGTGACTGTTCTCCTTCTTCTCTAAGGTCTGTTCCTTTGTTCCTTCTTTGTGCCTTCATTTGCTTTCTTTTGTTTGACTGTTAACTTAAGTATCTACCTTCTTCCTTTGGTTTCTTGTAGTTCTATCAGTTAGTTCTTCAATGGTTAGCTCTGCCTGAGCTGGAGTTGTCCCTCCAGCGAGAGTTGCTTTCTTCCTTGCTTGATCGGTAGGAGTTGCCGAGATAGCAGGTATTAGCTGTTAGCCGTCGGACTCATTTAAAGGAGTCCTATCGGGCTACACTAGTCTAAGGGCAGTTGAGTAACCGATTCCTACGCTTCCTCCGCTTACCTCCGTCGCTTAACTATTAATACGTCGCTTACCCGCCTCCGCTTACCCGCCTCCGTCGCTTGTTATCTGCTTCCCTTAGCTAAAACGTTAGGCGCTAGTGAAAGCCTTCACTCTCTTATATTTACGTACACGTGGGAAGAAGACCTAGTAATTGAGTGACAGCATTCCTGGACCAATTCTATAAACAGCCAATCAGGCATATCATGGTACAGGTGAAAGAAACTTATCCAATACAGATCTTATCACCTGCCCATCAACGAAAAACCGTCTGCTCCTCTCGTGCGTGTAGGAATAATCAAAAGAGGAGACAGCATGCCTATTATTAAGAACCCAATTCTACTAAAAACGATAACAACGATTCGAGGTTCTCAATCAAGGCAGAAAGCAAGAAACCCATCCAGCTAGGAGGAAAGCAAATCAAGCTATGGAGACCAATCAGACATCTAGATTCTATAGTACGCGTATGTGTACGTACCCAGGCTAAAACAGCAAATCAAACTTGGCTATCGTTTGAGAGCAACCTATCGCTAAACCGAGACCGAGATAGAGAAAGAGACTTTATCGGCCTCTTTGGGCTTTCCGGAAGACGGTTGAGTGGAGAGAGATTTTGAAGGTGGAACTTTATGCAATCCGCCATACGGGGGAGATGGCTTCGCGAATGGGCATTCGGAACCTTTGGGTAGAGTCTGACTCATTATATGCAGTCAAGATGATTAACTCCAAGCCCCATAAGCGCGAAATATGCTGCTGAGCTTCACTGAAAATGCCAGAGCAGTTTGTTTTTTTAGAGTCACACATTGTTGGAGGGAGAGCAATAGAGCCGCCGACTTGATTTCGGGATGTCGAGTTATGTCTTAATGTACCCAAGAAACCCCCCTTAGCCCTTAATTACATTATTAGAGAGGATATGCAGGAACTTCTTTATGTACGTGTATGAGATATTATGTACCATCTTTCTAATATTATGTATTATATAATATAAGACAAGAATAATATTTATTATTCTAATATATAACTGCAAGACCAATACAGGATGCAAAAAAACAAAAGCCCTTCTTTCTCGAGGAACGCCTAGATACATGGCTTACATACCCGGCTCAACATTCTTGGAAAACTAAAGAATCGAGGGTCCAGAAGAGAATTGGCCATTTAATCTTGTAAACTAATCGAGACCGAAATTGGAAAAAGAGATACGAACAGAGAGGAATAACCAATCGATGAAAGAACATGAAACCATTCTGTTTCAATAGAGGTACGAGAAACGGTTAGGGGCAATAAAGTAGGTGAAGTGGTTGGATTTTGCGAGCTGTTCCAACCACTGCTGGATGTGATTCCAAGAGCCGAACGAGAATGAATACCACACAGAAGAGTCAAGACCAGGCTCCCTAATGGAATGTTTAACCGATCCATTCCGGATCGACCGAATTGGTACGAAAGTTGTAACATGGGAAAACCACAGAAAACACGACTTATTTGAATAACCCGGTGACCCACCAATTGTAGAAGTAGGATCTTTGGCAAGCAATAAGTACTTAAATAATACAATTCGAGTGTACCATCTTCTTTCTCATTTCGAGGAAAAGGTGCGGGAGGAAAAGGAAACAACGGAGGGATCCGAATCGGACCTAAATGGGAATGACATGAAAAGTCTTTTTCAAAACCTAGCATTAAGGGCGTTACGACGATATACGAGAGGAATGGAGAAAAACTCGTGATTGGTGTGGAGGGGAAGATCAGTTTATGATATAGTTCAAGAAAAAGTCGTCTCATTTCCTTACTTCTTCTTTCTAATTCATTCACTTCAAGGCTGGTTCTGAACGCCGCGTAACATCATCTTCAACCAACCTCCACCGTACGTACCTTTCGGTGCAGCCACTAAAGAATTGCTTATACACTAAACAGCTGTTTATATACGCTTACTAAAAGACTTACCGATAACTAGTTCCAGAGGCATCTTCCATTCATATCGATTTGGGTTTTTCTGCACCATATTTTGATCTGCCTCTTCTTCGACCCGGAATTCCCATCAAATCCTTTACTCCTCGAATACAATGGGATTTTACACCTGGCGAATCTTTCACTCTACCTCCTCTTATTAACACCTGCGAATGTTCCTGCGAATTATGACCTTCGCCCGGAATGTGAGCAAATATATCATGTCGATTGCTCAACCGTACTTTGGCTATCTTACGTGGAGCGGAATTCGGTTTTTTCGGTGTTCTCGTTGAAACACGCGGGCATGCTCCTAGCTTCTGGGGACATTTATCCAAAGCTCGAGTACGGTCCGTGCGCCGTTTTTCTTCTCTACCATGACGAATCAATTGATTAAACGTAGGCATTATTCTCTTTCCTTTCTTTTCCCCCCCCCATTTTTTGCCCTATCACTTTACTCCCGATCCGAAGCACCCCTTTTCCATTCATAGAGAAATCCAATCGTCAAAATAAATAAAAAGGCCATCATGGACCAAAATCCAAACAAATCAATCTTGTTGGGAGGTACTGCCCAAGGAAAGAAAAAGGTGACTTCCAGATCAGGGATTAAAAATAAAATTGAAACAAGATAAAATCGTATATCGAAACGACTTCTGGCATCACCGGAAGGATCGAAACCACATTCGTAGGCCGACAATTTTTCTGGGTAGGTAGAACTATTGGAAGCAAATGGAAAAGGAACACCGAGTAGGATCAAAGAAACTAGCAGACTAATCACTAAATAGATAGAAATTGGTGCAAATTCTGACATCATTACAGCCCACTTTGTTTTCTCGCTCCTTGCTGGCGAAGAAGCGGCATATCGAAAAATAAAGAAAGAAGCAAAAGCCCATCCCGAAAGGTAGCTTGCTTACTTATGCGATAGAGTCCCCCCCAGGGCCGCCTTTCTTGAGGAACACTCTATTTTTGAGTTCCTTAAATTCTGGGGCCTCTTTACCGCGCTCATTTAGAGCACTTAACAGGCGGTATAACTGCTCCAAGGAGTGCTCTCCCGTGGCGGTACGGGGATTGTCGAGGGCCCGAGCTCCCCGCCCCATCCAATCCCCCGTTGGATCAAGGCCAGCCATTACCTTCACAATCTCTACCTTGACCTCGAAGAGGTCTTCGGCTTGAATGTGGGCTAATTGGATGTCCTCGTATGAGGGATAAGCAAATTGAGCCAGAAGGCGGCGTTCTATCGCCTCCACGCTATCCCCCCCAATCATTTCATCCCTTCTGTAGGGATAGGGGACAGATGGCCCAGCTTCGTCCCCCCCGGAAGGGACTGGATTAGCTGGAAGCGCAGGCCCAGCTTCCTCGGGACCCCGCCGATTTACCGAGGGTTCACCCTCTTCCGTTTGGGGTGCCTCGAGACTACGTCGATTTACCGAGGGTTCACTCTCTTCCGTCTGGGACTCCCCTGCCGGGGGGGAGTCCGAGAATGATTCCTCGAGAACTCGAAGTTCAAACGGATCCTCCTTCCACGTGGATGAGCTCTCCGCCCCGTTAGGGCCCGTAGAAGGAAGTGCTCCCCCTCCTACTGCAGCCCATATAATGGGCTCCAACAGGGGCAAAGCCTGACCACCCAGTAGGTGGATGACTTTGATCCGTATCAAAGATATTACCACGGCGATGAAAAAGACCAAGAAGAGATTGAGAATCTCCCTCAGTGGTCCTTTCAAACGAAAGTAATAAATTCGAAATAAAAATAGGAAGCCCACCAGTGCGAAGAATGAAACGCACGTAGTGGTCAGAATAACGCTTCCTTCTGATCCTAGAAAACGTCCGAAAAAACCTGCTACGGAACTACCGAGCAGGGGTAAAAATACGATAAGTAGATACATAATTTCGAGTGTGATCAGAAACCTAAAATCAGACAATGACAGAGCGGCCAGCGATTGAGTGATAGATTTCTCGACGTCCGGAGAACGCTCGACCGAAGAAATGAGTAACTAACAAGGAAGATTTTGTTCCCCAACTTGCTTTATTAACGGGCATTTTCGGGGACTAGCCCGCTTCTTCATTACTCAAGAGGGCGATTGCCCGGTCCTCTTTCATGTGGAATCATTTTAGATCGTATCCAAGCCTGTCTCCACTTTCTTATGTGAAAGAGGTGCTTGCTTTATGAGACTGAATGAAACCTTCTTTCTTCGATCAGAATACGAATAAGATCAAAAAGGCCATCATTGGGGCAAACAATGCAATAGCTTCGGTTAGAGCAAAGCCCAAAATGGCATAACCAAATGATTGTTTAGCCAATGATGGATTTCGCGCCACAGAATGGATCAAAGAACTGAAGACGTTTCCAATACCGATAGCAGCTCCCGCTGAAGCAATTGTAGCAGCTCCGGCACCTATTGATTTTGCACCTTCTAACATCTCGGGTTGATAATTCTCCTCACGCTTTGTCATTCACTGATCTTCTATCTTATTGATTCCTCGTCGATTCTTACCCTCGTTCCTTTAATCTTGGACATCTCACTTGGAATGCATTCTTTTCGATCTTGTACCCACGGAGTGGTAGACTGAAGACCAACTGAATCTCGACAAAGAGAAGTACAAGCAAGTAGATCATTCGACGTTAGAGGGACTGACTCGTAGCTAGGGTCCCATATCTCTTCGATTATTCAATTTGATCCGTCTTCGTCTTCTCTTATGAAATTGATAGTTTGTGAGATCGATTACTCCTAAATGCTGCCCTTCTCTTATATACGAGAGCTACTGCACGATTGGAGGATTAAGAAGGCCCAGTCTTTCGTAATGAAGTGCGGGACTTTTCCGCTATAATTCAAAGGCGTTCCTCGAGAAATGAAGAGCATAGTCCTGTAAGCGTAAAGCATAAGTAAAGAAAGAGAGAGAGCATGTACTTAGCACAAGTACTCAAGGATCAATGGCGTAGCTGAGCATACCGCGGCGGGTCTAATTGTATCAATAAGGAAGGGATCTTTGAATCCCGATGAGTTTTGATTAAAGTCTTTCAAGCTTTTCCTATTCTTTGGGTTAAAGAGTATCCTATCTTTGGAATTCATACTGATCCCGCGGTATGAGAAAGCAGCCCCTTGCACTTCCTTAGTCTAGTTCCGTGCTGTTCTCATTAAAAAGAAGAATCGCAGGAAGAGCGGTTAAGCTCCGTCAAAACCTTTTAAGGGTAGCACTGGCGCGAGGTCATTTCATTAGGTGAGGGTTCTCTTTCGTATAAAGGTTTAGCTCATCGATGAATCGGTCGAGCGGAAAAAAGAAAGAAAAGGGCCAGGGATTTGCCGACTGAGGCTCTTTTCAAGTTTGCCTACGTATCTGCCGCACTTGGCCAGGATCAAGTCAGCACTTTAGAATGAAGAAGACAATGCGCACATAGAATAGAAAGCTGCTGTTTGCAAGCTCTTTCTTTAGCATTATAACCCGCCCTTCCTGTTAAGGAGTGACGGAGCTCTCCTAAGCAAGAGCAAGGAAAGGTAAACTTGTTTTGAACAAAATTCCATAGAAGGTGCAGATGAATAAGCGGTATTGGAGGAGGGAACAGCCATAGTTGATGCATCGAATGCTAGTGCAATAAGACTTGATGAAGGCGGGATAACGGCACTTGCCTTCAAGCTTGATGAAACTTCTTGCCCCGAGGAACGCCTGAAAGCTTAGTGAGGAAAATCCTCGACAGAGAGGAAGTTTTTCTTTTAATGAGAGTGACCTTTCAAAGAGACTTTTTGCGATAAGGCCCTTCTTAAAGGGATGGCTCACTCTCCTTTAAAGGTGTCCCTGCTGCACTCTAAGGATGGCATATGATTCACTAGCGCAGGAGAGAGAGTTAGCCTTGGCTTTGGCATTTCCTCGCTCAATCTCATAAGAGAAGAAATCTCTATGCCCCCTGTTCTTGGTTTTCTCCCATGCTTTTGTTGGTCAACAACCAACCACAACTTTCTATAGTTCTTCACTACTCCTAGAGGCTTGACGGAGTGAAGCTGTCCCCTGTCATTCAGTGAAAATCTAGTGAGTAAGGTAAGGGGGTACGGAAACGTCCTCTTATCACAGCTTGAACGGGATTCATTTAAGGGAAGAAGACGAAGTCGATTCAATCCAACTCCAACCGCTCCAACGTTATAATCCTGGAAAGATATTGAGTCAATCTAGCAGGGCTAAGGAAAGGCACAAGCCCCAGTCAGCTCCGTCGGAGGTAAATCTAGCAGGGGAAGGTCATATGACCCTTGGGTGAAGGTGGGTTCCGACCTGGAAGTCATTACCAACATTCAAACAAACAAATGCCAGAATTACAATTGGTTTTATGAATAATAGCCAGAGAGCAAGGAATTGGTTTGCCTTTCAAGGGTAGTATCCACTCTTGGTATCGATCTGATAAAACTTTCACTGGCAAAGGTGTGAATTAGGAAGGATTGGTCGCCGAAATATTAACTGGAGACTGAATCTTAATATACCTCAGAACAATATATTTTTGTTACCACGAGATATATTAGCAGCTGCCGATCATTTGATTGGGATGAAATTTGGAATGGGTACACTTGATGATATGAATCATTTGAAAAATAAACGTATTCGCTCTGTAGCGGATCTTTTACAAGACCAGCTCGGGTTGGCTCTGGCTCGTTTAGAAAATGTAGTTAAGGGAACTATAGGCGGAGCAATTAGGCATAAATTGATACCTACTCCTCAGAATTTGGTAACTTCAACTCCGTTAACAACTACTTATGAATCCTTTTTCGGATTACATCCATTATCTCAAGTTTTGGATCGCACTAATCCATTGACACAAATCGTTCATGGGAGAAAGTTGAGTTATTTGGGCCCTGGCGGATTAACAGGGCGAACTGCTAATTTTCGAATACGAGATATCCATCCTAGTCACTACGGGCGTATTTGCCCCATTGACACGTCTGAAGGAATCAATGTGGGACTTATTGGATCTTTATCAATTCATGCCAGGATTGGTGATTGGGGGTCGTTAGAAAGTCCGTTTTATGAACTCGTTGAGAAATCAAAAAAGGCGCGGATACGGATGCTTTTTTTATCACCAAGTCAAGATGAATATTATATGATAGCGGCAGGAAATTCTTTGGCTCTTAATCGGGGCATTCAAGAAGAACAGGCTGTACCAGCTCGATACCGCCAAGAATTTTTGACTATCGCATGGGAAGAGGTTCATCTTCGAAGCATTTTTCCTTTCCAATATTTTTCCATTGGAGCTTCCCTAATTCCTTTTATCGAACATAATGATGCGAATCGAGCTTTAATGAGTTCTAATATGCAACGTCAAGCAGTTCCACTTTCTCGGTCCGAAAAGTGCATTGTTGGAACTGGATTGGAACGCCAAGTGGCTTTAGATTCGGGGGTTCCCGCTATAGCCGAACACGAGGGAAAAATCCTTTATACTGACACTGAGAAGATAATTTTATCGGGAAATGAGAATACTTTAAGTATTCCATTAATTATGTATCAACGCTCAAACAAAAATACTTGTATGCATCAAAAACCTCAGGTTCGCCGGGGTAAATGTATTAAAAAGGGACAGATTTTAGCAGATGGTGCTGCTACAGTTGGTGGGGAACTCGCCTTGGGGAAAAATATATTAGTGGCTTATATGCCATGGGAAGGATACAATTTTGAAGATGCGGTACTCATTAGTGAGTGTCTAGTATATGGTGATATTTATACTTCTTTCCACATACGGAAATATGAAATTCAGACGCATGTGACAACCCAAGGTCCTGAAAGGATCACTAAGGAAATACCGCATCTAGAGGGCCGTTTACTCCGAAATTTAGACAAAAATGGAATTGTGATGCTAGGATCGTGGGTTGAAACGGGTGATATTTTAGTAGGTAAATTAACGCCTCAGATGGCGAAAGAATCCTCGTATGCTCCGGAAGATAGATTATTACGGGCCATACTTGGCATTCAGGTATCGACTTCAAAAGAAACTTGTTTAAAATTGCCCATAGGTGGTAGAGGTCGAGTTATTGATGTGAGATGGGTTCAGAAAAAGGGGGGTTCAAGTTATAACCCCGAAATAATTCGTGTATATATTTCACAGAAACGTGAAATCAAAGTAGGTGATAAAGTAGCCGGAAGACATGGAAATAAAGGTATCATTTCCAAAATTTTGCCTAGACAGGATATGCCTTATTTGCAAGACGGGAGACCCGTGGATATGGTCTTCAACCCATTAGGAGTACCCTCACGCATGAATGTAGGACAGATATTTGAATGCTCGCTTGGGTTAGCGGGAAGTTTGCTAGATAGACATTATCGAATAGCCCCTTTTGATGAGAGATATGAACAAGAGGCTTCGAGAAAACTCGTATTTTCTGAATTATATGAAGCCAGTAAGCAAACAGCCAATCCATGGGTATTTGAACCCGAGTATCCAGGAAAAAGCCGAATTTTTGATGGAAGAACAGGAGATCCTTTTGAACAGCCTGTGATAATAGGAAAGCCCTATATCTTGAAATTCATTCATCAGGTTGATGATAAAATACACGGACGTTCTAGTGGACATTATGCACTTGTTATACAACAACCCCTTAGAGGCCGTTCTAAGCATGCGTCCCAACGAATACCAACAAGTGGAGTTAATGCTATATCTCATCCTTTCCTTAGTAGGGTTTCTCCCTAAACAAAATACCGATTCAAGCCCCTTTACTAGGTTGGGCAAGCTTGGATGCCCTTACTCCCAACAAGTTGCTGGTCGGGATCGTGAAACTCTCGCTCGAAAAGTAAGCTGGTCCTTGTTTGGTCATAAGGATAATCGTTCTTATTAGGTCAGGGGCGGCCGGTCCGGGGGTTACCCGCGATTGGTAATGGCATAAGCAGAAGAGGGGTAGGGGGGACAAGGTTACGCGCTGGGTAGCGCAGCGCATCTGTTTCGGGACAGAGGCGACGAGGGCGAAGCAAGCCACCCAACCCAACAGGTCAGGGTCGGGCCGGCCATAGGTTCGAATCCTGCCACCTTTCTTGTGGATCATCCTGTGGTTACCGGATGATGGGAATAACAAAGCAGAAATCTTGAAATGAGCACGAAATGTCAATATATGAATTCTTTCATTATTCGTTATTTCCGGGTCTTTTCGTTGCATTCACTTACAACAAGAAACAACCACCAGCGTTTGGTGCAGCACCTGCATTTTGGTGCATTCTTCTTTCTTTCCTTGGTCTTTCGTTCTGTCATATTCCTAATAACTTATCCAATTACAACGTATTAACCGCTAATGCACCTTTCTTTTATCAAATCTCAGGAACATGGTCTAATCATGAGGGTAGTATTTTATTATGGTGTCGGATCCCAAATTTTTATGGATTTTTTCTTTGTTACCGGGGTCGACCCCAAAGCCATAATGTCTTAAAACAAGGAGGCCATAGGGAAAGTCTTTTTTTTTTTTTTTTCTCGAACTTCGTGAAGAACTCAATTTTATCTCTCCCTCGTTACGAACAAGAAAGTGGTTTGAAAAATCAGTTGTACACTCCCTTCGTTCTACGAACCCTTGTTGATTCTGAACTTCGTTTGCGAAGGAACCGGACTTTTGACGGACCAGCCCTTTTTTATGCGCCGCTTGATCCTGAAAGGAAAATGAGCTTTGCTCCTCTGGACGCTTGGCGCTCCCGTGGTTCGCGAGAAGGAAAAAGGACTCATCCTTTGTTGCATCTGGCACGAGATGATAAAGAGAGAGCTTCGTCTATCGATGAACAGCGAATTGACGGAGCTCTTGGCATTGCTTTGTTTTTCTCTCCTTTCCTATCAGCGAGTTCCGATCCTTTTGTTCGAAATTTCTTTGTTTGTACCGAACCGCTTGCAGAATCAAATCCTGTTCCACAAGATCCTATATCAGCTATACATCCTCCTTGCATTTATGCCGGGGACGTCGCCAGTGCGGAGGGCTTTGGCTTATGTAGATCAAAAATGATGAATGGGATTGTGGCACTCCACTCGCCGCCAATGCGGAAGGATGCCGCCGAAAAGAATGGAACGCTGCTTCGCTCTGCTGGATGCGTCGGATCCCGTATAACAAGCGAGCTTTTTACCCTCAAATTCAAACATGTGGGCGCAAAATGCTATCCTGCTCTATTGTTACGTAGCAATAGAAGCCCGCTCATGCTGCTTCGGCGGCGCTTTTTCGCCTTCTCTTCGTTCTGGACAGGAGCGAGAAGCCACTCGACTAAAAGGTACTGACGTAACTCGACTAAAAGGAGAGGAGCGCTAGTGGACACGGGGAGGGAAAGAAGACTGGAACCGTGCATCTTTGCTTAGCATGGTTCGACAAAAGAAAGAGGTGGAAAATGGACCGAGAAAATAATGCTTTGTGAACCCAATTGCTTTGACAAAAATATATGAAGAATCAGTGCTATTGAGGAACATTTTATAGAAAGAAAAGAAAAAGAAGCAATAGTAAAGGAGGGCTTTCCCAGTGCATGAAGGGAGGGTGAAGCAGGGTAAGTCATAAGAATCCGCTTTCTTACAAAGACCTCCTGCTATGCTAATGAGGGGTCTTAAGCAAACAAAGTACCAAGAACTTTGGATATTATCCGTTTTTCTATTATATCCCATTTTATCCTTCCGCTTTAGGATTAGCCCAGCTTTTTCGAAACGGACGGAAGGCCTAACTAGAAGCTATTTGGCGCCTTCCCCTCGATGAATACTTGGAAATTTGTCTTGCATCGTAAGCTGTCAGAAGAAAGTAAGACTTAGAAAGAAGACAGTAAGTAAGAGAACTACGATTTACTTGATTCCCAAAGTGGTACGTAGGCAGCCAAGGACGAATCCTTATCCAGTTCTTTGTTAGTAAGTGAGGAAAAGATATCAAACTTTTTTTTGAAAAAAGTTCGTAGTTAATCTACCTCGGACGTACCCATGGCGTGTGGGGTTCGCGTGGGGAACCGAGTAACCAAAGTCACGATCAGTCTAAGGTTGAAATCTGGATGGTCTTTTTGCCAGACGCGCTGGTTCGAGTCCAGCTCGTGACAAAAGGCTACCCTTTCTCTTAAGAGAATCTCGATATCCTCAAAAATGAGAAATGGAAAGCAGCTTCACATCACAAGTGAGGAAGCCACCGGTGACCGGCTCAATGATCGTATACGCTCAATAGAATATGTACGCGCGGGCGAAAGAATTAATGCTGCTTATGCCCGCCCCCGAGGATCACCAAGCAGTTGTATCTATTTCCGAGGCGGTCAGTCCCATCCCGCTGAGGATGAAAAAAGGAAGTGCATGGATTGTTTCTTCGAAGTTGTAGGGAGGGAGTTCTCCCGCGTGAGCGAAGTTGGAGAACGAGTTCCCGGCTCCTGTTGCTCTGTTGCTTGTCCTGTCATTCTGTGCAAGGGCGTAAGCCCGTAAATAACCCCCTCTTCTGTTGTTTATATTGTCATGAAGGTATTAGTTAAGTGAGCGAAGCGGAGGAACCCCTGCTGATACATGGGCTGTGTTTGCTTGTGCGTGCATTCCTGCCATGATAGAATCCCTCTTCTTTGTTCTTATTGGGAATGGTTTGTCAGCGAGAAGTTAGGAGTTGGGGCATCTGCCTGATTCCTATGGCTAGCTGTGCTTTCCCGTTTAGCAAGTGAAGCTAGGGCCCGCCCACTCCTGCTGCTGGCTGTTTCGGTATAGAATAGCTCTTCCTACTGTTGTTTACTGGGAATGAGTTGCATGAGGAGTCCCGGTAAGAGAGCGAAGCGTTGCTCCTGAGGTGTGATGTGCATCTATCCCTGAAGTTGTGACTGGTGTATCCTTCTTATGTGTGGAGGGAACCATTGCTGCCCCCCCTCGCACTTGTAGCTGAAGTAGGCCCGCTAGTTCCGTAGGCGAAGTACTATTTGTTTGTGCGTTTATTGGAGGAAGTATCTAGAAGTCCCCAAGGTTTCTTCTAGTCTTCTCCTCCCGACCGTTGCGTTTGCCGTGTCTGCTTTCCTTTGTCTTTCCTTTCGCTAATCCCTCTAGGTCTGCTCATCAGACAACTATCTATTTCTTTGTGTCGTCTGGGAACATTGTTGGATGAGCAAGGAACGGAGTTGTGCGAGCCGAGTTCCTGTTGTGTTAAGCATGAGACTGTCCGAGGAACGGAGTGGTCTCTCCGAATGTTGAGCAAGTGCAGCTCTTCCCCTTCGTTCCTTTGCTGACCAACTAGCAAATACTCCGCAGGCTCACCAAACAATTGTTGTTTAATGGGAATTGTTGTTCATGAGCAAGCCAGGCGTTCCTCTCCTATTAGTCGAGTAATTGGCGTTCCTCGGGGTTGACGTCGTCGTTAAGGTTCAGAGTTGGAGTGATGTCATGAACTTCCATTCCCGGGGAAGAGGCTATAAGGCTATCTGGGGAATAAGATCGGTTGGCAACTAATAATTATCTTATCTTTCTTTCCCACCTCAACAGAATAAGCCGAAACCAGGGAAAGGGCATACCAAGATCGGATCGCATAAAAGGATTGGAAATGCTGCTCCGAGCGAAGGTAGCCGGCTAATTAGAATAGGAATACTCGGACTCGGCGAAAAGGGTTAGGAGGAACCCCTTTTCTTCCGCTAGGTAAGTTGGGGAAGTCCAACATAAGGCGGGTCAATTACATCGAACCTCAATTCCATCGAGCCTTGCTCGAGGAACGCCTATGGTTGATAGTGAACAGCAGATATGCTACATCAAGCACTCACTTTCTGTCTATTGGCCTTAGAACACTCCTTTTTATGTTGTTTTCAATGAAGGGGAAGTGATTCCTGAGTTCCTTCGCACAGGGTTTGAATTGAATTAAGTAAGTTTTGTCTACATCTTCTCTTCAATTACACCAGGAAAGAAAGCAGATACTGATGCTGAATGCACTTAACAAATCCCATGTCTGAATGCCTTAAACTTCGATTCAAAAGAAAGCAGAACTAGACTGCCAACGATCTGACGATAGAGAGTCTTCACCTTGTGGACCTGCTTTCGCCTAATGGTAATCTTTGTCTTTCCTTCTGTCTTTCGGGGATGTGATTGGGAAAAGAGCCATTGGAAGGTGACTAAAAGACCAGAAACAGGGGCTACCCGAGCTAATGATAGAGGCAAGAACACTTTCCGGCCAGGCCTGACTATAACCAACCTTACAGATCCCACTCAACCTTTTACACCGATGTATCGTACTCTCTCGACCAGGTCATCATAAGAAAATACTGCTAAATCTTTCCAAAGTTATAGAAGGAGGGAAGGCGCGCTACAACTAAATAACAGCCAGCTGAAAAATGCTAGCTAGTTAGGCTAGCGCGCAATGGCTTTCTCTGCTCTGATAGGGGCTTGCTTCTTCAAGCTCTGCCCAACCTATACAAGGTGCTGCTCGCTTTCTCTCAGCCACTAGTGGCTTATGTGGTGGTCGACATTCCTACGCGCTCCTCCTTGCCCCCCTACTTCAGAATCCAAAGGTCAACTTTGGGTGTTGTCGTGACGCTTTGGTTACGAAGGTTACCGGGGTCTAGGTTTATGGATGGAGTCAGTCAGCGAAAGTCCTCAATCAATATCAACAAGATGTCGTGACCGCTTAGACTTGGTGGATTTTGTCAGAAAAGAAAGTTGGTTTCGGGGGTTCATTGATTAGTACACCTCTGGTGCTGGTAAGGTCGGAACCGTGGTCGTCCGTCTCCGGTTTGCCGGCCGATAAGATCTCTGAGATTGATCAGACAGCTGGGTTGGTTTGGCTATTCCTTTCTATTGAAAAGGAGTCAGCTGTCTGCGCGAGTCACCTTCTTTTAGGCTCCGCTGAACGACACGGCATTCTCGTTCAAATATAGGCCGGCCGTACTTGTGATGGTTCCCAAGGATCCAATATGACCACTTAGGTCTACGTTGCCGCGATATTTTTCTATGGAAGCGGGCGGGCTGTTGTTAGAAGTTCGGCCCGGTTTTTTTTGGTGTCGTAGGGGAGGGATTGACCTTTCTAACGCATATTCAGTCGTACCGGCATGGCCCCACTGATTTGTTTTCGATCGGAGCATCAAGCAGCGCAACCCGGTTCTACTTGACTAAGCCCCGTGCTCGTCCAAGGAGGGAGTTTGCTTTACCCAACTTCTTTTTTGATAACAAGGCAGAAACCTCCGACCCGACATTCAAAAGTTTCGAATGAAGAATGAAGAGTGCCCTGCCCCAGCAAGCACCTACTCAATTCTAAATAAAAAAGCGTGACTTTACTAAACAAGCAAGAAAAGCCCTTTCGCACTTCTTAGTAAAGCCTAAGCGCCCTTGTTGCTAAAGGTAAGGCCGGCTTTCTTCGCATGCTTGAGCGCATTACGCATTAATATAATACATATATATAAACCTTTCCTTGAGTTTTCAATAAGGGGCATCTATAGTAAGGGGCCTTTTCAATAAGACTCGCGCTAGGCGATCACGTTTTTTGTCTTCCCAAAAATCGAATATTTTTGAGTTGGTAAAGACCCACCCCTAGTTTCAGTCAGAATGAGTCCCCGGGACCCCGGGACATTGGCTTCCGCCAACAGTGGACTATTAAGGATCGCATCCCGCGCATATTCTACATTATAGCCTGCAACTGATTCAGCTTCCGCTTCTGGGAGATCAAACGGAGCTCGATTAGTTTCTGCTAGACGAGAAATAAGGAACATAACCAATACAGGGAACAGGGGAATACCAGACCATATCTGCTTTTGCGCCATGACAATCTCACTCGAATTACGGGGACCTACACATATTAGTACAGTATACCGGGGTGTCCCCGGCCAGAACCACACGTGCAAGTTTCCCTGCATGTGGCTCGTCCGTGCTTTCCGAGGCGCTGCCTGTGACTCAGCATGAGAGAAGGGGGCGGAACTGCACACTCTCGTGTTCAGAGCATTGTCCGAGTGAGTAGGCAGCGCCTACCAAGCAAAGCTTTCTTGAAGAGGCTGGGCTGCTTCCTTTTCGGCGCCCGCCTTTATTCATCTATATTAAAGCCACACACGACCCAATAGGAACGATTTCATCGCCCCTCTCTAGATAAGAAGCAAAACGCGCCATCACCTACAGCCCTTTCCTCTGCCGGGGACTTCCATGAAATGAAAACGGGCGGCATCGTTGTATGCTCGACATTTGTTGCCCTGGTTTATATCCCGGAGTACTCCTATGGCCGATCTGTCACCCAACCTACTTAAACAACCTAAAGGAGGAACCAAGCTTCTTCAGCGAAGCTGGAGGAACCCCTGTCCCGCTTTTAGAATGACCCTTATGGCACAGCTTAGTCAGTTATTCTCGCAGTTCAGATAAGATTCGGACCGCCACTTCTCTGAAAGCATGGTGCTTGCCTCCTCCCTCCTTGGAGCTCGTCCATTCGTTGGGTGATCCCTTGCTCTCACGTTCGAGTGTTTTCTCGTCGTTTAGGCCGGTGAGTGAGATTTCTGCTCATTGCAGTCACCTCCGGGGTTCTTCGCACCTGGGATAGTACCAGGACCCTTGTGCCCCGGCTCTTGATCAGATAAAGCTGCCCGCCCGAAGCCCGACCTATGACCCACAACTAAAAAAAATGAGCCTTGCGACGAGACGCGGACATTACCCATGCTGCGGTTCCCTCCGGTCCGTTCCCGGGTTGGGTTAGGGGAACATCCGAGCGATGATTGCCTTCGCGGATACAAACGCGCTCACAAGGCGCACAATAAGAATAAGACCAATAGAGACTTCATAAGAGACCATTTGAGCTGCAGATCGTAATGCTCCTAGAAAGGCATATTTCGAATATAGAGGAGTTCAAGTTACCAACAATCAACGAGTTGATCATACCCTTCTATGAACCGTACGAGCACGTCCTCTCTCACACCCCACCGCGCTTACGGCTCTATACCCCAACCCTACTTGCTCTGGACCCCCCCGGTCCTCCCTTTCTATTCCTCGGTAAGCGGGCCGTGGGAGCATGGCGGGGGGCGCTATCCCCTTTTGACTGATAGAAAGCATCTCTCTTTTGTCTCTCCTGACCGAACCCGCCAAAAAAATCACAAAAAATCACAATAGAATAAGTTGTTCTTGCCGGGAGAGTAGCGTCTGAGACATCTTTATCTGAGGCGTTCCTCGGCTTCGTCGTCCGGCTCATCCTCGGAATCCGAAAATCAAACTGAGACAGAACGGATTGTTTCAGTGACATATCTAATCAGACTGAATAGGATTTGAAGAGCTGTCACCATCATTCAATTCACATCAATTTAAGCAGGCGTTCCTCGGCGCGGAAGTTACCGTTTATAGAATGCAAGCAAGGTAGCTTGCCTGCCAAGCCGATAGGCGAAACGGCGAACTGATCGACTTGCATGCATGTGTTAAGCATATAGCTAGCGTTCCTTAGTCTCAATCACAGACCTTTTTCCATTTTAGGTGAACGAGGGTTACCGGCTCTACGACCTTGCAAGGCACTACAGTAGAGCTCGAGGAACGCCTGCCACTTTCTCAATCGAAAATGTCAACTGTGAAGATTCTCGCGTTTCTTTATGTAATTTCAGGATTTCTTTTCGTTGATCGGATCGAGCACATAGTAAGAAACCCTAAGTCAATTCATTCTCTTTGGCTGAAGTCAATCTTCATCAAGATGGCTTACCGGTAATCTCCCATTCCCGCCGTCGAGAGACTTTAATAACTATAGCATGCCAGAAAAGGGGAGTTTAGGTGCTTAGACCTATAGCCCGAAATCTTCTCTTATAAGCCGACATAACATATTCAACCACGGATTAAGTCGACATAACATCCTCACGTTCAGTCAGGGTTTTCCAATTGGATTACCTTGCCGGAATTGGATAGAAGTAGGGCTAGACGCGCCGGAGGAACCCCTTCTGCTCGAGCTCGCCTTAGGAAACTTTCCCCAAGGTTTCAAGATCCATCTTTCAGGTTCCTTTCAAGCTGTAAGGCTCGCTCTGTTCTCAAGGCTTGGTAGCTCTTCACAAAGTCCTCCTTTTTCCACGAGTTGGAACCGTGCCGACACATTTTCATATCGTCCCCCAGTAATAGAGTGGTAGGTTTCTTCCCTTTCTATTATGTATGACGCCTGAATCCTTCAGCTACATCTAAAGAATCCCAACCATTACACTAGAAGAAGATAGGCAACTACCTTGACCAACCAAAAAACAAATGAAGAAAGGAAGGGACGAAAGTGACTCGAGCAAAGCGAGAGGGATGAAATGAATAATGAATAAAGGGTTTGGATTCCGATTTGGAAGCTTGAATAGTGATAGATAAGACAACTCAACTAGATTTCACGTCTTTTCTTGAGAGTGCCTTTCCATCGCAAGTAACTGAGCTATCTAGGGTTCAAAACCTGGGGAAGTCACTTCAACAGAAGCCTTACCAAAGTCAAGAGATTGGGCTCCTATAGCTATAGTTGGAGCTTTTACGAACAGGTTTAGAGTAGGCAATTCCAGTTCAAGTTTCAGCTTGATAGCTATATCTTTCCTTTCTTAACTGGGTTTTTGAACTTCACATATAGCCCATCAGCTTGTGACTTCGCAATCTCAGTTTCGAGATTGGGAATCGACAGAACTGCCCTTTCTTAACTTAATTAAAAAGGGTAGCTTAGCTGGTTAGATTTACTGAAGGTAATTCAATCGCTGAACGTTGATAATAGCTTTCGAGAAGAGATCCTGACTCTCCAGCTAGTTCAAACTTCCCCGATTAGTAATGCTCTCACCCTGGGGACATATCTCACCTTTGTCAGCAAAACAGTAGAACCATCTTCATTTCTCAGTCTCACACTTCCTATTCCCTTCACTGTTGACATGGTGTCATTTGCCATCTTTACTTGTCCACTACCAGCATCTGATAAACTCACAAACCATTCTCTTCTTCCGAAAGAACAACCTGTGTCAAGTATCCAGACTTCCCTTTGTTAGGAATCCCTAATTGAAAGTGAGAAGGGACCTACACTAGTATATAAAGCTTTCACCCTCTCCACTCATTGCCAATTGGTTTTGAGTTGGAAGCCCATGAACACTCACATGGTATCAAGAGCCATGGAGGGTTCTCTTGAGTCTTATCCTTTTCCTTCCAATATCAATATTACTAGTTGTGTCTCCATTAAACTCAACGATCGTAACTATCTTCTATACGACGAAGGAAGGGTGCGATTGAGTCCGCCGAACTTGGGCGAGAGATGTGACTTCGAGGAACGCCAGAGCTCTGGTGTCTACGAGAAGCTTCATGAGTCAAACAATTGAGGAAGCCGATACTTATACTTTGGTAGAGAATCTCGCACAAAGCAATGGGAGTAATGGCTCGGATTATGATCGGACTACAAGGAAAAACAATGATGAATCTCATCATGCCATCCAAGAGCTGAATGCAAAAATGGATAAGATATTGAAGCGTGACCAAAAGATGACGGTAAACTCTTGTGAAGAGTATGGTGGATATCAAGGGTACCAAGACTTTGGTGTTGAAGGGTATGAGGAGCCACAAGAAGAGTTGAACTATGTTGGAGGTCAAGGATTCCAACCAAGACCTTTCAACCAAAACTATAGGAACCACCCGAGGAACGCCTTTATAACCAAATGAAACTCTTGGAGAAGTAGAATCCATCTAAGTAGCCTCGGTTTCGCGTCCTTCTTTGCCAATAAGTATCTCAAAGCCGCGTGGTCCGTGTGCACTATTACTTTGGAGCCCACCAAGTAAGACCTAAACTTCTCAAAGGCGAAAACTACGGCGAGGAGCGTAAGCGCGGTGGTGGCATAGTTGACTTGCGCGTCGTCCAAGGTTCTACTAGCATAGTAGATGGCGCTCAACTTCCTGTCCTTCCTTTGCCCCAAAACGGCTCCAACCGCGTAGTCACTTGCATCGCACATGACCTCAAATGGTAAATTCCAATCCGGTGGTTGGACTATGGGCGCGGTCACTAGAGCCTCTTTAAGCATCAAGAATGCCTCACTCAAGGCATTTCTCATCAAACACAAAAGCCACATCTTTGCATAAGAGTTGTGTGAGTGGCCTAGCAATCTTCGAGAAATCCTTGAGAAATCTCCTATAGAACCCTAATGTCCTAAGAAGCTTCGAATACCCTTTACCGAGTTTGGTGGTTGCAAACTTGTCATCACCTCAATCTTCGCTTGGTCAACGAATTTATTACTTGTTTTGAACAGTATTCTAAAGACACGTCTTACGAAAAGCAGATTTCATTTTTTTAGGAAATTATTAAGTGGGATTCTGAAACGTATAATTACAGCTTGCACTTTTAACGGCGAACTCTCGCAATAAGCTCTACTTTTTTTCTTTCTAGTTTTGGTGTTCCGATATCTTTCGTAGGATGAGTTCGATCCATTAGGTTCTTCAATTTGTTTAGAAAAGAAACAAGAAACAAGAAAACATCTTTGATTACGATTAAAAGGAACAATCTCAAATAGACCAAGATCAAATTTCGGGTCATTTCTTGGTGAACATGATCTGCCATAATCTCTTCGAGGAACGCCTTCATTTATGTGCCAGAATAAAGAGAGATTTGGTAGGAAAGTGGAAGAGACTTTATTGTATATGATAATCAAAGGGAGTGGGAAAGCTGCAGTAATTCTTTGGAAAAGCCCGGTTCTCTTTGTCTTCGAGCTTTCATTTTTTGAATCCACTCTTTCGTTCCTTCATATACCTCCCCACCAATAGATAGAGACAAATAGGAATAACCAAACCACGTCTACAAAATGCCAGTACCATGCAGCTGCTTCAAAGCCAACGTGATGCTCCTTCGTCAGATGACCAAGATATTGCCGAATACCACATATAATCAAGAAAAGAGTACCTATAATCACATGAAAACCATGAAAGCCTGTTGCTAAGAAAAAGGTAGAACCATAAATACTATCCGAAATAGTGAAGGGTGCTTGATAATATTCCATTCCTTGAAAGCCAGTAAATACTAGAGCCAGTAAAACGGTAGCTACTAAAGCATAAACTGCTCGTTTTTCCTTCCCCGCGAGTATAGCATGATGAGCCCAAGTTACGGCAGCTCCGGATGAAGGGAGAATAGGGGTATTAAGAAAAGGGATTTCCCAAGGATCTAAAACCCCAATCCCTTTTGGGGGCCAAATACCTCCGATCTCTACCGCAGGTGCCAAAGAAGAATGAGAAGAAGCCCAAAAAAAAGCAAAAAAGAACATAACCTCCGATACGATGAACAGAATAGAACCATATCGAGGTCCTAATTGTACGACTTTGGTATGATGTCCTTCCAACGTGGATTCACGTAGAACATCGCGCCACCATACGAACATGGTATATAGGAGAAATATGAGGCCCAAACTTAGAAGTCTTGCACCCCCTTGAAATGGGTGCATGTACATCACACCTCCTACGGTGGTTGCCAAAGCTCCGAGTGAACCCGAAATAGGCCATGGACTTGGATCTACCAAATGATAAGAATGCCTCTGAGATTCAATCATAAACCACTTTGTCTCGGTTGTATGTAAACCCCCACCCTTCACCCCCCAACGGGCTCTTTCTCTTTGGGGTCTAATTTTCTTTCTATCTGACAGGACAAACTAATAGGAAGGGATGGTTCTTTCATTGCATTGATAGAAGTCTAACTAGAAAAGGATCTCTCTATTACTTTGAGAAGAGAATCGTTGGTTTGACCGACGGAAAGCATGGGAGAAAGAAAGATGCACAAACAGACGAAAAGAAAGAAGAGAATGAGTCACAAGATAAAATGAAAATGAGTTCTCACTCTTATCATTTTCATAACACTGATTGACGATGGCCCGAATCCGAATCTCACCTCAGATGTTCATCTGGCCCTCGTTCTCATTCTTTGTGTTATCACGGGCCCGCTTTAAGAACTTCTTTTTATTTATAAAATCGGTAATCTCCCCATTGGGGTCGTTTTCCCTTCTGGTCTGGCCTTCTCATTGTTCCCAATTCGGTTTATTAGCTCTTCCACACATTACCGGTGGCGAAGGCACTCCCCTTATGGGCGGAAATTGCAGCCCCTCTTTCGCTTGGTCAATTAGGGAGCCAATCAGTTCGGCTAACTCCATTTTGATCTTTCTTACTTCTTCATATTTTTTTTTTAGATTAAACTATTCCTGTTGTTCTGGGCAGGTCGAGACGCGGTCGCTTCGAAGAGCTGGCACCGCTTCCATCGCCTCCGACGGAATGAGAACAGCCATCCATAAAAGACATGAAAAAAGAATATAATGGTACAACAATGAAAGCGGCTGCCCAAGTCAGGCTCAACTCCATAAGGGGGCGCTTCATCAACAAGAGAAGGATATTTAGAAAGGCTAGCATAATAAAAGAAAAAAATGAGCTTTCTTATTTATTTTCGCAGAAACATGAGTTGGCTTTTACAGTATCTATAGAATGTAAGAATCATTTTATGCGAGGAATTCTTCGTTGGTGGTAAGTTAGCCGTGAATGGACTCTAACTCTAATGGCTGAGCTAGGTATAGATGCTTACCAACCTCAACATCCCGTGCAATTGAGATTTTTACGTTCCATATAGTTTGAGGAGACACAAGGTTCTAGAGGACCATATCAAAAGAAGAAATTCAGATAAAATCTATCATCGGCTTGGCTTGCTTCCTAGGGGTTTGGGGAAGTTTTGGAAGTTGTGAAGGTCCTCTAAGGTAACTCTAGGGCTCGGGGTCCAGGCCAAGCGGAGGTCAACAGAAGGTCAACTCGCTTCAGAAGGCTGGAGGTAACCTAAGATTCCTAAATGCAGGGGTCTCATGCATTCCAAGTAGCTGAAAAGTGGCAACTTGGATGTTTGAAAGACCCGTCGGGTTCGCTCGGAGACTAACTCACTCGTCTCGTTCATGTATTTACTCGCAGCATTCGCAGCAAACTCGCAGGCAGGAGAGACCTTGAGTCCAGGCTCAGGAATCGACTGGTTCCGAGCGGACTCGGGCAGCTGCTGCTTGGATTATCGTAGAATAAGAGGAGCCGTCTTAGGAAAGGAAATGACTCAACTTCAAAAAAAAAGACAGATGCCGCCTACCTACTCGTGCTCGTAGCTCGATCGGAGGTCAAGAGCCGTGGTCCGGCGGAAAAACGGAAGTCGTCCGTATCAAGTAATACGTGAATTGCTCAGTAGTGCTTCGCCACTACCGTAGCTGGCGGAAATAGCTTAATGGTAGAGCATAGCCTTGCCAAGGCTAAGGTTGAGGGTTCAAGTCCCTCCTTCCGCTCCTGGTGTTCGAACTAGTCATTAATGGTCGGCTTCATTGGTATCCTTTCGGTATGCCTTGCGAACATTTTCATTTTTAGCGCCTTCTCTTCTTTAGAGAAGCCAAACTCGAACGGATAGAGCAGATGGTCCAACTACATAACTTTTTCTTTTTTATTCTTTTTATGGTCGTGCCTTGTGGCACGGCAGCACCCGTACTATTGAAATGGTTCGTCAGTAGAGATGTTCCCACGGGTGCCCCTTTTTCCAATGGTACTATAATTCCTATTCCTATCTCTTCATTCCCTCTTTTGGTCTATCTACATTCCAGGAAAATCATACGCTCCATGGACGGAGCAAAAAGTGGAGTCTTGGTCAGAGCAAGTCGCCCTATTCTATTACCAGACATAATTGGGAGAAGCTCATCCGAAACTAGAGCTAGAAAGGCCTTATTTTTTTTCGTTCCCGTTCTTCATTTCCGTCTTCTCGAATCCAAGGGGGACTTCTCATATTTAGAATCTTTCTGCGGTGTGCTCTGTTTACTATTCTTTCGTACTTTCCTCTTTTTAGCACGCGATAGGTCAGCGAAGCGTGAGCGGGCGCGGAGAAGGAAAGGCCAAACACTTCGGCCGAACGGGAATGAGCAACGACGAAATGACAAGATGAGGTGCTCCGGGCACCCCCATTTAGATTTAGAAAGAAGGGTCGAAGGTTTTGGGCCTCTAGCTTTCCCCGTCCCCCCTGAGTTGGGTGGTGCTTGTGTGGGGGGCGTGCCACCAGAAATCGGGCTTGAAGCTCTCGCCTTACCAAGGAGCCGACAGCTGATGGCTATGGCTGTTGGTCACGACTACTACCAAAAGGTTCCAATGAAGATGAATATTTCACATGGAGGAGTGTGCATCTGTATGTTGGGTGTTCTTCTGTCGTGCGACCCGGCGGCTTATGTGCGACCTGTGGCCCACGCCTCCTATTTGTTCAGGGCGGGCGGCGTGAACTCTGATTCGATCCGGGTATTCAATCCCGCCGCTGAGATGCTCAGTTGACTCCTTAACCTTGATAGGAAGATGGCTTATTCAAAAATTCGTGCATAAGGGTAAGGAACTTTGGATGAACTAATGCGAATGGGTGTAAGCTTCGCTGCTCGGAAACACCCAGTGCTGACCACACTGAGAGACACGAAAGCGCGGGTAACGCTAGTTGGCGAAGTGGCGTTAAGCATCCCTAGCGGTACGAAAAGAGAGGTCGTGATGATATCATCTACGTCCGTACCGCTCCTCGTGGAGTAGATCCCGCATCCAACCAAGTCTTTGACCAGGGAACGGGATAATTCCCACTACCGCTGGCAGGCCAGCCGGGCCATGAGCGCGGTGGGAACGGGCTTCCCAAAAAGCCAGCCCCGGGCCGGGGTCAGCATAGAATGAAGGGGACGGCCCTAATGTTGTGTTGGCAAAGCCAACTTCTTAGGTTGCGGGCGGAGAAGAGCGGACGTGGGGACTCGGGTCGCGGGGTGCAGCGTAACTAAGAGAGCCATTCCATTTAGGGCGAGACAGAATGGGCGGGCACGAGCGGTCTGGTGTCCGAGTCAATTGGTCAGACGATGACTACTTCACTTATTAGATTAGTATTAGCCGCCTATCCCGGAATGAAATGAGATAAAATAGAAAGAACGCGAAGCGCTAGCGCTATAGGATTGGTTTTTTGCGGGGGATAAGCTCGCTTCTTCACAAGCTTACCCCCGCCCCGACCGGCAGCTGCTGGGTCTCCCCATCTCTCCTAAATTTCCCCCGGTCTTCGGCCCGAGCTGTATGAGGCAGAAACTCGTCCCACGTACGGTTCGGAGGCCGAGCCCCACCCCAGCAGTAATGGTGCGGCTTAGGTCAACTAACACAAAGAAGATACAGTTCACTCAACGATTGCCTTTGGGTTCCGAACTCCATATGGGGAAGGAGCGTTGTTGTTTGCGAGGTCTCGATCATTTACATGGACCCACTTCTCATTCCATTTGTGGGAATTTGATGATCTATAAACCGTCCCTAACGAACGATCGGCTCATGTTTGAGCATGATGAATCACTTCATGCCGACCTCTTGCTAATAAACTTTCCGGCCTCATATAAGAATGGAAAACTTGAGCATTTTCTGCATTGGTGGATGAAGAATCGCAAACATAATAATTTTTGGTTAACCATGTTCCCAGAAAAAAGATACTTTCGAGAAAGGACGAGCACGGCTGAAGTGGCTATACATACAAATCTATTTACGGATCTATATGCTTCGATTGGAACTGGAAGTTCCAGAACAGGAGGTTGGTATACCACCATAATGAAACTGCCTTTTATTTTTTTTATTCGGATAGGATTTATGTTGGCTTCGTTGGGAGGCTTGCCTAGTTTGTTACGTCAGCTCCAAAAGGATAAGTTGCGTTGGAATCGAGAAAGTTCCGTGGAGTTCATAATTGCATAAAAGGAGTCAAAGTAGTGGCTGCGGCGCGTCAAGGCACTTCTTCGGCGGTCTCCGTCTCGCCCGCCTGCCATCAGTAATAGGAAAGCTTTCAATCAATAGAAATCGTATTCGTGAATAAATCCCCTTTGTTTGAATCCAATATTATGCACTTTTTTTCGGTAGCAGGACTCTCTTCTGTCCGTTTGAACTCTTGAGCTGGAGCAGGAGAACGGAATAGGCATCCATTAGCGGAGGAACCCCTTCGCACGACATCTATGAAAGCAGCAGTGGGCGAACCGGCGGTGAACAGGAAGAGGGAGGACCGCCCGATCGCTAGGAAGAAGCAAGCCTATTCCCCGCGCGGATGTCATTGCTCACTAGCTCCTTTCGTCCCTTCCAACGCACCATCAACAAAAAGAAAGAAGAATTCTGAATAAAATGGACTACTAGAGCCGCGGGAGCGAGCCATAAGCGAGCCTTGTCGTAGTATTAGGAGCGATGGAGCTTTCCCAGTGAAAGGAATACGTAGCGAATAAGGGGAAGAATAGAAAAGCACTCTTCGGGGCTCACTATTGCCTCTATTACATAACCTTTCTCGAGGAACGCCTGGTCACTTAACCTATTAGCCTGGCTTAGGTCGAAAGGGCTTCCCTTTTTCCTGCTTGACCTTCGTAGCGCATCAAAGAGACTTCACTAAATCACTTTTTTCTTTCCAAGTAGTCAATTAGACTAGAGCCAACTATTTGATTCGGCTACACTTCTTGACTTCATGAATTTACAGATTGAAAATGAAAAAAGGCAATCATTTAGAGGGGGATTTGCAAGTCTTATTCACCACTTTCAACTGACTCCTTATCCAATCAACAGAAAGCTACTACAGAAGTCTCGGCTTACTATCTTAACCAGCAAGAAAAGGTGAATGGAAAATTGCCTCAGATATCTTTCTTTCAAAGCCGTGCTATCTAGCTCATCTTTATGCAGGAAAGGGATGCGCGTGACTAAGGCTATACGCTGCCCCAATTGCTTCTTCGCTCGTTCTCAAGAATTCGATCTTTTCACATCGCCAAGCTTCTACGGTACCAAAGAAGAAGGAAAACTTTCTGTTGATTCTCTGTTCACATTCTCTTTCAATTCCTCTGCATAATAGACACACATTCCCAGCAGACTATCTAGCTTCTTTGAACAATCTTCTTGCTGTCTCTGAACCTTCTTCTCAGAAAAAGAATGAGACCAAAGAGCCCATCCTTAGTTGTTTTGTTTTCTAAGTTCCGTTTCCTTTTATTAGTCTGAGTTCATCTTTTCCGCTTTCAAGCGTGTACACACACTGAAAAGGGAGAATAATATCCCTTTCATTAAATAGGTAGCTCACTGGAGCAAGCAACGAAGTGCCATAGGGTTCAGGATAAACTTATACTGAAGTAGGTACAATCTCTTTGTGGATCAATCTATAGAGTTCATAGAGAAGTAAAAACCTGAGATTAGAGTTGGAGAAAACCGCATAGAATACGAAAAGCAAAGGCGAAGGTAACTTTACATCTCAGTCGAAAGCGGTGAATCCGGATCCATTTCATAAGTGGACTGGGGAATCTCAATCTCAAAGGTCTTCGCTCGGTTCCATAGTTCAATCTAAAACCTGTGAGGCTGGAAACTTCTACAACAATATTACATATTCCCATCACCAGTAAACGATACAGTAAACCTTTAAATAGGTTCACTCTCGTCCATGCTCAAGGGGTTCCTCCAGCCGTCCTTCTGAAAGGTCCTCTTCTACGGCTCAAGGTTCAAGCTAAATAAATAAAGTTCCTTTTCTCACTTAAGCGGATACTCCAAGTCAAATCAATACTTTAAGGAAAGACTTCCCAGCGCAGTCAAGCAAGATAGGATTCTCAACAGGATAAGTTCCATGGACAAGGCGAGCTAGCAGCGGTGCTTTCCTTCCGTGCCGTAGGTCAATGAAATGATCTTTTTCAGCACGAGAAGTCTCATTAACTAGTTGATGAGGAGAAGTCTCAAATGCTCTCATTTGAGGAGAAGGAGAGAGTTACAGGATCCGAAGGAGATGGAAGAACCTGGCCAAAGTAAGTGTTGTGTTCAACCGTTAGTAAGTAATATGGGTTGGCTGGTTGGAAGGACAGCTTCAGTATCTTACGGCCTGACATTACATATGCTGTGAATCGTTTAAGTCAATTTGTTGCTGCTCCTCGAGTTCCACATATGCAGGCTGCCACTCTAATTCTCCATTACCTCAAGAATTCTCCTAGACAAGGTCTGATGTATTATGCAGATTCAGAGATTCAACTTAAAAGCTTTGCAGACTCTGATTGGTTTGGATACCCTGATACCCAGACGATCGATCACCGGTTTTGCTGTGTTCTTGGGTCAAAATCTGATCTCCTGGAAGTAAAAAAGTAGTATGTATGAGTTGAAGTGAAGGGCGAGGTTCAAACGAGGAAAGGCTTACGGTGGATACCAAGGCACCCAGAGACGAGGAAGAGCGTAGTAAGCGACGAAAATGCTTCGGGGAGTTGAAAATAAGCGTAGATCCGGAGATTCCGGAATAGGTTAACCTTTTGAACTGCTGCTGAATCCATGGGCAGGCAAGAGACAACCTGGCGAACTGAAACATCTTACTAGCCAGAGGAAAAGAAAGCAAAGGTTGAATCTAGTTCTCGGAGTTGCGTCTGCTCTATCATATCTGCATTCAGAATGTGAGAGAGAAAGAATTCATAGGGACGTTAGTTAAGACTTGAAATATAATGCTTGATGCAGAATTCAATGCCAAGCTAGGAGATTTAGGCAGAAGTCTATGAACATAGTTCTATAACCAGGGAAGCTACAATACCAGCTGGGAAAATGGGAGATCTTGCTCCTTAATATGTTTATTATGGTGTTCCATCACAGAAATGTTTACAGCTTCGGTCTAGTGGTGCTAGAGGTAGCTACAGGGAAAAGGCCTGTCGGATGATGCTGGTACGGTGCTTGTTGATTGGTATGGAGCTACTGGGAGAAAGGGAAACTGATTGAGGCAGTTGATCCAAAGTTGAAAGGGACGTTTAGTTTCATTTAATGCAGTGGAGATGCAGAGAATGCTTATGGTGGGACTTTACTGTGTTCACACAAATCATATAATGAGAAGAGGCGTTCCTCGAGGCGTTCCTCGAGAGGGAACCTCTTCTTAGGCTTGCTCCCATATTCTCCATGGTTGTGGGGGTTGCTTCTTTATTCTTTCTTGGGTGGTCTTTCTCAACGTTATTCGTCATGGCTGATTCTAACTCAAATAAATCCGCCTCCAACACTTCATCTGATCAGGTCAGTATGCAGATTACCTCTGTAAAGTTGAATGGTCATAACTACTTGTTATGGGCACAAGCCATTAAAGTAGCTCTCGGAGCTAGAAAATCAAATGGAAGTTTCTCTTGAAAGATCCTCCTTCCATATCTGATAGGCATTGTGCAAAGGAAGATTTTTTATGTCTGGTTCTATCTGTCTGTGCTTGGTCTGGACCCTTAGTTCTTGCAGGTGTAAGCGTGCTCCCCATGTAGTTTTCTTCGTTCCCGTGTATCAGGTGCGAGTGTAACGAGTTTGTTTGAAATAAGTCCAATGAGATGATACGAAAACAAAGAGTGAAAAGCTGGCTTAGAAGAAAGTATAGTGGGGCATGGTTTAGAAGGTAGGTTACCTGCTCGTAAGAGGCAGTCTAACTTAGTTAGAGGAAAAGCTTGCTCATCAGAAGGATAAGCAAGGTTTGATGAATTCTCCTAGAAGGAAGATCAAAAGTCAAGGTCTTCAATAGTAGTATAGTAGGGAACATATCACAACCAGCAAGCGTATTCACGTCAACATTTTAGCAATCGAAGTCGGAGTGAGCCGATTCAATGTCTACAGGGCGTCTGTGTAACACATATCAAAGCGTCTGTTGCCAAGCCTAATATCTGATATCTGTGTTAGGAGGAACGGAGTTGGCTTCTCCCGTTGGTCAAGCGCCTTGCCCCTAACAGCTGATTCACTAGCTAGTCCTCCAACTACTCTTACTGGAACAGAAAAGACTAGCACTGGATACGTGAACATTTCTAAGAGCTGGTACGTGAACTGCGGGTACGCTTAGAGCTTCTCTTCCCCCAGGACTGGATACTCTCACGCCGCTTACTCAAGCACTAGCAGCTTATTTGTCGACTTCTCTTCTTTGCTTTCTTACACTCTGATTTGCAGCGAATCAAGCGTCTCCCCAGTGAGTTTTCTTTCCCGCTTTCTTTCTCTGTATTATAGAATAGAAATAAATTCCTCCCTTACAGGAAGTTTGTTCGATAGGACAAGTACACCCGAAGGGAGAGGGTTCTACGCTACTTTTACGATCTTTCCTTTCAGTCGAGTAACTAAAGTCCTGTAAGGGGAAACCTATTCCCAAGCTAACCAAACCAGGGAAGCTAGCTCATGGGATTGGTTGGCTAACAACAGGCGTTCCTCGGAGTGAATAGATGGGGGGATAAAAGAGAGGATCAGGGAGTCATCGGATCGGAGGGTTTCCCACCAAGGAATTCATTTCACGATTCGTATGTCGATCACTGTCTTCGATGATAAAGGGCCATGTGATAAGCTAAGAACCAGGAGCTGCGCCCTTGCTATTGAATCTGCTCTCACTGTTCTCGAAGAAGTTGCTATTGAATCAATCAGCTGTGAACGAGTCGGATGTTGCAAGAAGAAGGGCTTTGGTTCTATCTGTAGAAAAAAAGAACCTATTCACCAACAACCTCTGATGAGAATATAAGAAGTTGCAAAGATCGCTTGCTACATTAGATAGGATACAAAAGATAGGATAGAAAGCAATTCCATTACAAAATCCGGTGCTAGAAAGATAGGTTGAACTCGGCAAGGCAAGAACACAACACTTTTGATTTTCTCACAAGAAGAAGCTGCTAAGATAGGTTGTTCAAGGTGAACCAAAGATAGAAGGTTAGCGGCATTGCACACTAGCCCAAACTGATTGAGATAGAACCTCTTCTTCAAGAAGATAGGTGGAATCAAAGAATTCCGACAACGAATCTCCCGAGGACTCAAGACCGATCCTTTTTCGGCGGATCTGTCCTTGCCTCTTTCGTAGTAGATAGAGCCATGTAAGTTGGTTGTTCTCAAGACAGAAGTCTATTCCGGTTTGGTTTGGAAGGAATGAAAAAGAAAGTGGTTGTAGCTCACTTCAAACAGTAGGGTTGGATCGGAGGAAGAGAGGTCTGACAGAGAAGAGGTCTCCAGAGCCTGGGCATAGGTGATATGTAATAGTTTACACCAAGCCAGGTTTATTGCAACTGAAGTAAAGCAGTTAAGTACCTGGGAGTTGGAACTGTTAGAGTTATACCCGCTTGATATTATTGGGAAAAGCAGAATCAAATGTATCTATTAATTAGGCCTGTCAAGCTAACCAGAATATCAAATATAGTACCAGACCAGTGGGAACATCAGCACCCTTAACTTAAGAGAAACTGAAGCAGCTTAGAGTTCCAATCATAGTTCTCCATTTGACGTGACGCGAATAATAAGCGTGTAATCATTGTCAAAAGCTAATACCTGTTGTGGGATATGCCCTGTAGGCAGGAGTAGGAAGGTCAGCTAGGAAATCAACAGATAACAACAACAACGGAGGGAATGGGTTCCTCCGGCATCTAGGTAGAACCTTCCAGAAAACCGGGAAAGTAGGGCATCATATAGTAAAGCAGGACTAAAGAAAGGATCTTTGACTGTCCTAGAGAGCTGAAATCGATCGTACATTTCACCAACTTCCATTTCAATAGCTGCCAGAGAGAAGTAAAGTGAGAATATAGGTATTGCATTGGTCTGGGAAAGCCCTTCCTAGGGACCGGGGAAGCTTCATGGCATTTATCCTACTCTGTTGCCGGGGAGGATAGCTCCTTCATTGACGAAAGGTGCTTCAGTGACCAATGTTGGTGCCATTTCTTCTCTTGGAGGAATGGTAGTTTTGTAGTGTGCTTTTAAACTCCAAAGCTCACACGGTGTGCCTTCCCATATATGCTGTCTTCACAGAAATAAAGAGATGACACCTTCTTACCATCAATGTACCCTTTTTTCACCAGCAGTTCTAGCCCTTTTTGACTCATGTGGCCTAACCTGCTGTGCCACAGTGAAGTTTCATCCTTCTTGTCCTCAGTTATGAGAGCTTCTGTCTCAACAACTTTGCCTTGTAGGAAGTATAGACTGTCGTACTGTGAACAACTCTGCAACCCTTTAGCACTTTCAAGATTCAATTTTCACCTCTATATGCACAACCCTGTGAATCCAGTGTTCCTAGTGAGATAAGGTTTCTCTTTAAATCTGGTACATACCTCACTTGTGTCAGAATCACCACTGTTCCATCCTCATTTAGGATCTTTATACTTCCTATTCCTTTCACTGATGATGTTGTGTCATTTCCCATCTTGATTATCCCTGTGATCTCTTCATTCAATTCATCAAACCACTCTTTCTTGGGTGTCATGTGGTAAGAACAACCTGTGTCCATGATCCACTCTTGTTCAACCAGATCAGTAATATTCAAGGCTTCAGCTGCATATAGGACATCCACACGCTTGACCTCTGTTCCTTTTACCATAGCAGACTCTCCTCTTACTGTGTTCTGGTCTTTTGTCTTATCATGATAAGCCTTTCTACTGGGACAATTCTTCTTAAAATGTCCTTCTTCCCCGCATGAACAGCATCCCTTCTTTCCCTTTGACTTTGATCTATTTCTGTTCTTGTTTTTGTTTGAACCTTGATCTCGGCATATGAGATTGATTTCATCGAAGTTGAGTAAGAGTCACTCCGTACCTCGGCGAGCTACACACAAATCTTTCTTTTCATTTTATATAGATGCAAAAGTTAGCAATCCAATTTTCCTTCGTAAGAGCACATCAGAAATCCAAAACTTTCTCTTTATATAGCGAGGATTTGATGAAACCCTATTTTAGCTCTACGGAGCACATCAGGAATCCAAAACTTTCTCTTTATATAGATGGAAAAGTAAGAAAAAAGGAAAAAAG

>sca1

AAATTATCAATTTCATAAGAGAAGAAAGATCGTTTTTTTAAATTATCAATTTCATAAGAGAAGAAAGATCGTTTTTAGAAAAGAAAGAACGTTTTGATTCGAGGCGGATCCCTTTTTTCTTTGCCTTTACGAGTAAGAAAGTGGTTACGCTCCGCTGGGGAGCCGGAACTGTAAATTCCTACTGTGGTTCGAGTCCACAAACCACTGAGAGCTCTGGCATCGGTTCAGGCTAAATCCTGAAGAAAGCTAGAAAGCGCTGTTTGATGAGCCTGCGTCCGATTTGTGAGTTGGTTAGGTAAAGGCTGACCAAGCCAATGATGCTTAGCTGCCCAGTCGTGCCGAGGAATTCCCATCCGACCAGAGGAGGGAAGAGAGTGACTAAGCAGCAGCTCCACTTGTCGCGTACTTCTTTAGCTGCACCGTCATCGCCGGCCGGACAAACCCTTCCAACAAATGGGGAGTGGGCCAAAGGAACACGACACATCCAGTGAAGAGATCCCGGACAGGGAAGTCAGAAGGTCTGCAAGCCTCGTCTTCGCCCCTGCTTTAGCTGCACTTGGTGCGTAAGAGGGAGACCCGGTCGCTCAGATACAGACAGCAGCTCATTTTACGCTTAAACACATCCCAGGGAATTAAAGGAATACGGGCTCCGCCTAGCTACCTGATCCCATCATGAATAGGAATTACCAGCGAACCAAGAGAGATAGATTCAACAGAGGAGAAGGGGTGGCGTGCTTTAGCGACACCTTCAAGACATCCAGGAGTGATCTAGACGAGGTGGGACATGCCGGCTAGAGTACATGCTTCAACTGGAACATCGCTTCGCTACTCTTCCCTCCCTACCCCTACGGTCGTACACTCTGTTGATAGTTCGCCAATGGCTCACAACCAGACAATCATAGTATGTTCGTTCGTTCACTTCGTTCTCTCTCTCCCCTTCTCCCGTTAGCTTGTTCCCCTCTTCCTTTCTCAACCTTCTTAGCCCTTCCTCCAAGAGTAGTTACGCCTTTTCCGTCAGTGGAGAGAGCGAGCAACGACCTGTGAGCGACCGTAGCTCCCTCTGCAAGTGGTAAGGCGCAGTTCACCCCCTATCGAACGGAGGAAGATCGCTGTTGATGTCATGACTAACTTCTTGATTCTTTGATGAGAGAGGCGTGCGTGTGAAATGTCTGATTCATGGCTTGGTTAACGAGATGTCCCAACTGGTACTGGAGCACACCCGTCTGGTTCGTACTATCATTGGCTTGTTCATCCTTTACCTAAGCCTAATCTTCTTCTTCATAGCACACTCTGCTACAGCACACTACGCTTCTGTGCTGCTCAGTAGTGACTGCTAGGGAGTTAATCACACCAGGCGGGCTTGCTTCGCTAGAAAAGAATTCCACTAGTGTGTGCTGCTGAGAGTGAACTATGTTACTCGGAACAATATCTGAAAGGAAGCTTAAGGAAAGGCCTTAGTATTCTTTCTTCAAAGGCGTTCCCTGAACAACTACAGCTCGATAGAGACAACTCGGAAACTAGCACTGAAAAAGGGCATACTATCAATATGATCAATATGATATTACCTCGGTGTGCTAATCAAAGGTAAAGAACTGAATACTTAGATTTCTCTTATCCTTCCTATTCTATCTTTGTTGTTTGTGAATGAGTACCTATCCCTTATGGGCGAACGACGGGAATTGAACCCGCGCATGGTGGATTCACAATCCACTGCCTTGATCCACTTGGCTACATCCGCCCGCTACGCGCAACGGGCTCCTCGCCCTTCATTTTCTTTCACTGTCATAAAACCTATTAAACAACTCATTTACCTTTGTATCTTAGCACCAAACTCAAATGAAATTATTCTCTGCCTAATCATTAATGAAGGGGCCCGCCTCACCACTCCACTTCCCCTCATCAACTAATTAATGAGAGCTTCCCCTTGTTATGGAAGTAACCAAACTCACCATGACAAGGGCCAGCGCTCTTACAGCGAAAGGCTTCTTTTCTCTAGCCAAAAACATTATGCCATCAAGCCAAGCCAGCTCCCGAATGTCTTTAATCGAGAGAGGTACAGAATAGTCGAAGAAAGTAAGCAACTGCGACAAGAAAGGACTTATAAGATAACATGGAAAATGAGATTTCACTTCTAGTTATCCGCCAAGGAAATGCCGGAGGAATTCTATATATAGCTGAGCGATGACCCTTTCGTTGTGCATCTTGGATACAGTACCGAAGGGTCCGCTCTACTAAAACTTTGTCATAATGAGAGCGGGGATCATGAAGATTGAAACTATTCATGTTGTTTTCTATAAACTCCAAGGCTTCTGGTCTTATGGCCCAGGGTGATTGAGATCTTCTTTTCTTCTCTTACGATATTTCGAGTTGGATGAACGGATCGCTCCTAAAGGTTTAATAAGACATTAGATTTTCATTCATCAATACCTCGACTTCCAGCTTCTCACATGGAAGGGTCCGGTCCAGAAGCAGAAGCCTAAGTTCTTGGAAAAGAAGAAATAGGGATGTGCGGAGTAACAGTAGGAACCGCAACACTAGTATCAGATAAGTGAGGCAAGAGCCAATCATCATCAATGTCGGGTGTGACCCGATCGGAAGTGCATCGACCGATTGATGCGTAGAATCGATCGAACGAGATGAGGAAAACGGAAATTCCTCTTCGCAGAAAACAACATCACGTGAAAAGGAGATGGTGCCACTCTCAAGATCATAAACCCTCCAGCCCTTCTTTCCAGAGGGCGACGAAAACGCATCTTTTGCTTCGACTCCCTTGCCCCTGTCCTTGCAGCCTGCCTATGGTCAGCTCAGAACTTTTGGGTGTCTCTGCTATGGTTCTACATCATCCAAGCAACGTCATAAGTTCCAACCACGCTCTAGAGCCTGTGTATTTCTGGGGTACCCCTCTGGTGTTAAAGGGTATAAGCTCATGGACTTGGCTAGTCACACTATCTACATCTCTAGACATGTGGCATGAAGACATCTACCCAATGGCTTTTGCTCATGATGCTCAAGGTACTACTTCTTCATACCATTAGATTCTTTGTCCTCAGAAACTCACATCTCTCCATCATCTCTCTCTCCTCCATCAACACACTCTATCACCACTCTACTAAGCGGGCTTCTAAACCTCCTGCACACTTGAGTGACTATCATTGCTATTCATTGCGTGATGATATTCCTTATCCTCTTTCTTCTTATCTATCTTACTCACTCTCCTTCCTACATGATATACATCAATAATATCACAAACCAATCCCTCATTCTTTTTCTGAGGCAAAGACTTCTAAAGAGTGGTGTGATGCTGTGGATAGAGAGATTGGTGCTATGGAATCTACAGAGACTTGGGAAGTTACCAGTCTCCCTCCAGGGAAGAAGGCAGTGGTAAATGGGTTTTGACTGTTAAATATCTCTCTGATGGCAGTGTGGAGAGATACAAAGCTCGTTTGGTTGCAAAGGGTTATACTCAAAAAGCTGGTTTGGACTACACGGAGACCTTTTCTCCAGTTGCTAAGATGGCTACTGTTAAGTTGCTCCTTAAGGTCTCTGCATCTAAGGGTTTCTCACTCAACTTGACATCTCCAATTTCTCAATGGTGAGTTAGAAGAAGAGATTTATATGAAACTGCCAGAAGGGTATGCTGAGAGTAAGGGGGACTCTTTGCCAAGGAATGCTGTTTTACGTCTCAAGAAGTCCATTTATGGCCTAGGCTTCTAGGCAATGTTGAAGTTTTCTGCAGTTTTGTTGTTGGTTTTGTTAAAGGTCATGGACACTTTGTTTGTTAAGTGTACTGATGGTAATTTCATTGCAGTCCTGGTGTATGTGGATGATATAGTGATTGCCAGCTCCTCAGAACTTGGTGCTGCTGGTTTGACTGCTGCTTTGAAAGAGAAAAACTCCGGGGGCTCTCTGAAATACTTTCTTGGCCTTGAGGTGGCCAGAACTTCTGCTGGTATTTCTTTGTGTCAACGGAACTTTGGAACTTCTATCCTCCACTGGTATGCTTGCTTCTAAGCCCTCCAGTGTTCCAATGACTCCAAATCTGAAGCTCTCTAATACTGATGGTGATCTGCTTCCAGATAGAGAGGCTTATAGGAGTTTAGTTGGCCGGTTGATGTATCTCTGCATCACCAGACCAGACATCACCTTTGCTGTGAATAAGTTGTGCCAGTTTTCCTCTGCTCCTCGCACAACACATCTCAAGGCTGTTCACAAGGTACTTCAGTATTTGAAGGGGACTATTGGCCAAGGTCTCTTATATTCAGCTGATGCAGACCTTACCCTCACTGTGCTGATGCTGACTGGGCATCATGTCAGGATAGTCGGAGGTCCACTACTGGTTTTGCTATGTTTCTTGGTTCCTCTTTGATCACTTGGCGATCTAAGAAACAGGCCACTGTCTCTCGCTCATCTGCAGAGGCAGAATACAGAGCTTTGGCTCTTGCTTCTTGTGAGCTCCTATGGTTATCTTCTCTCTGACTTACACATTACGCTCCCATCTCTACCGGTTCTGTTTTCAGATAGTACTGCAGCCATCTCCATCGCTACAAAGCCTGTTTTTCACGAACAAACACGTGGAGATTGATTACTGTTCGAGATTGAGAAAGGTTTGCTCCGGATGCTTCATGTGCTGACAATCAGGTTGCAGATATTTTGACAAAAGCTTTGTTCCCTCATCAGTTTCATCATTTACAATCCAAGATGAGTCTTCGAAATCTCTTCGTCTCATCTTGAGGGGGACTAATAGGAGATGGTTCATCCCGGTTCGATGGTTTCATCTCTGATCTATTGGTTAAGGACCGGTTTGATATCAATTTTGACCGTTTGTATAAATACCCTCGTACAGTGTTCATTTAATTAATGAGAAAGTAATCTTTGACAACAAACACTTTCTCTTCTTCCTCTCGACCTCACTAATAAACGTCTACTTCTTTTCCCACTCTGAAGCAGACGAAGCGAGCTGAAGGAAAGAGAAGATGCCTCCTAATCTACTTATGGATGTCAGAGTTGATGGTCGAGCATAGGTCCATTAGCGGATGGAAAGAATGCTTAGCGACACAGCAGATACTAATGAGAAGGCATTGAAGGAAGTTCGTTTTTTTAGGCTTTCAGGGTTTAGTTAAGACTTTGCCGTCTTTTCCTCTCGCTTCGTAAAAAGTCTGTAATACTAAGATTAATGAGATTTTCCGTTTCCCAGTCGACTATGTGAACAGCACAAAGCAAAGTTTGACCCTTTAGTGTCCAATGATATCAATGCTTTCTGTTTCAAACAGGAGGGAAGCTTTATGGTTTCGTAGCTTCTACTTTTTCTTCTACTTTAGGGTTTGATAGAATCGTCCGCTATTACAGACTTTGATTATCTGTGGGGCAGTGCGTTAGACGAAGGAAGTAGCATCTAATCTAAGGAAGATGAAGACCGGATATTCAAAGGAATATTTGACAAAGCAGAAGCATTACAAGCATACATAAAATAGGAAGCCTACACACACCGTAGACCAGCCACACACCATTAAAAAGGAAACTAAGAACATAGTACATAAACCAAAGACATAGAGCAAGCATACAAGACTTTGGGTCGACGGACTCCTTATTTTCCATTTTAGCGATATAGGAAGTTGGCGGACTCTTCTATTCTAGTCTCCCCGGCGACCGTGGGTGACAGCCTGTATTATTAGGCCTTCCTGCTCCTTCAGGAGAAAAGAAATCCTGTGTTTCAGACCGCGAATGCGGTCCCGTAAAAGTTGCATCCTTCTGCCCACGACCTCCGGATCGAGAGCAGGCGGATGCTCCCAGAACAGACCAAGCATTTGCTCACATTGGAGCTTTTCGTTTTCCACGGTTTGTATTTCTTGTTGAATGCTCGCCAGTCTGTTCGCGATATCCATGTCTACTCACTTTCTTGCCGACTTCTAAGTGCCCTCTTTGCCAAATAGAGACTGAAAGAAGCTTCTATTTATAGGCAGGCCCTAAACCCTTTCTTATTAGTCATAATTCTTGTCTTAGTTCCGTAACATGGTTCAACCCCGGCTTTTCATGAATATGGAACCCCCTTAACTAGATTTTAGTGCGCTGAAGAATTCTCCCCTGGATAAAGGCCCCGCCCCTCAAAATCAAAGAGTCCTTTTTGGCGGGTATTGCCACGCGGCTTAATTACGACCACTCCCTCGGGAATAAGAGGCAATAATAGAACGCACTGTATAGAGTACTCCCCCGCTTTTCTCGGGTTTCCAAAGGCCCGTCACTCCTTACTCGACAGCTCAAAGCTGACCAGAGTCCTCGTTTACCCTACGTGCACTATTTAGCTCTGCCTACTCAGATCACGCTTTTATTTGTCTATTTTTTATTGGCTGATTCCCAGGTAACTGACTTCGGCCAATCCTTACTCGACAGCTCCGTCCTTTGGTGAGCACCGAAGCTAGCTCTCTTGAATTTCCCCGGAAAGACGGAACCTTTCCAGCTCACTCTGTCAGTCCACTAAGACGCGTATAGAGTAAATGAGCACAATAACTTTTTCAGTACAATAAGTCCCCTTCTCCACACTTTTGTTTTTGGGCTTTCTTGGATTCGTTTCCCCAGTAATTCCATTTTGGACTCGGGCGTGGGCTTTTCCATTGGGCTCAGTATCATAATTATCCCAAAAGCCCCCTCTCCTCTTCCTGCTTGTCAATGGGCTTAGATGGATCAAAGCATTAGATAGAATGCTAAGTCCAATTCCTCAATATTCTTCTTCTTCAGTGGCCCAAGCAAGATCATCTATCAGAGAGGATAGGGTCCAAGGTCTGACTCTTAGATATATCCCCTACTACGAGTTACTCCGTACCTCTGCCTATTACTTCACAATTACCTAATGTGTTAAGCAAGAGACAAGTGGAGCTTTTTCAGCAAGCTTTACATCAAAAAGTAACGAAAAAAGACGGGTTTGAGCAACTTTTTCATACAGTTTTGCCCTCTGATATAGAATGAACTAACTTACGCAAATAGTAAATGAGAGGACCTTAGCACTTTTGGTTATGGAGTGATTCATCTCAAATTCCCGGGGTTTAGTGAATGAATTCAACTAGAAAATCTCAATTGCACGGGATTCGCGATTCGGTGTAAGTTCCCTTCGGTCGTCCTTTCTCGTTCCGGATTCAAATCCTTTTTTGGTTCCCGGCT

>sca2

AAAAGCAGAGGAGTGTGCTGACTTCAGTAGCTAGTCAACCAACTTCTGCTCAACCCTAGGAACAAGGAAAGTATCAGCCGATAACTCTGTTCACTTGGTGTGGGATTCCTAAAAGTTTACTATTATCGCACGCCAAAGAGTGTGCTGAAAAGGGAATAGTTGGTTAACTACCGAAGCTAGAAACAAATACCTGCTAAAACCAAGGGAAGCAAGAGTAGGAACTACCCACCCAGGAATCAGCATTCTAAACTGCAGCACAAGCACAACCCTGCTTTTCCATTCCTTCATAGCCCACCTCTTCATTGGCCATAAAGCCTTGATTTCCTTGGACCACGTACACTGATGATTGGTCGGGCTGGGCTACTCTATGGGCGTATGCGCCTAAACCAGGCTCAAAGCGATGAGACTTCTCGGTTAGACTCAAACAATAGATCAGGTCAGTTTTGAGGAGCTTTATTTGACTTAAGAAGAAAGGGCAGCCCTCTGATATATCTTTCGACTTCACCCTCGGCCAAAGCACAGTAAGTTAGAGACTCCTAGTCTCTACTAAATCAATCTATAGCTTCAACACTCTCCTAGCGCAGCAACCTTCTCTCTTTCTC

>sca3

ATTTGCTCTTCCGTACAAGGAGGGTCTCAGATCTGTTAGGTTCTTCTTTTGGAGTGTGCAGGGCATTCTCTGTGATTCCTCTTGTATGATGGAATACCCGTCCTCCGCCAGTCTTTCATTCGCCTATCTGCAATACCGCTTCTACTCACTGGATTTCTTTCGATCTGCCGCATCCTATCGTATGACCTTCCATCTGGCTGAGAGTAGCATGGGGTAATTGCCTATCGTTGTTTTTCTGCTCATTGGAAGAGACTAATTGTTTTGATAGGGAGGCTCCTCACGCACAATCCACAAGAGGGTCGGTATCTCTTTTCATTGATTGATGAGTAAGGGCTCGATAGAATGCTTGATCGATGTTTTAAGGCCTTGCCTTAACCAAGAGTCATCGCATTCGTCGCACGAATCATAGATAGAACCCGATCCTTGGATAGAATCCATAATCAGAAGTTATCGGTACCCTTAGCATCTTACCTTTGATTAGAATGTGAAGGTCCTTGGTTCGGGCAAGCAGCGTATTTAGAAGCCTGCTACCGAAATGGAATTCCTCGGTCGAGAACTTTCATGATAAAGCAGAATTCCGTTGGACCAATAAGTAAGAGAGTACCTTCTCCTAGAGAACTTCTTTCTCTAGTCACTAAAGTGCGTTTCAGTCGAGTCACTGAGCGCCCACCGGGTTGATTGAAAGTGCCCCTACGGACTGGGACACCTTCTTCCAGAGCATTCTCACCATCTGTACTAGCCTTGCATGAAAAAAGGATGAACCATGAGGAATTCTCCACTCTATGAATTCATAGCGCTCTTGGGATGGGCATGTGTCTTGCAGTAGGTCAAAGTGAAAGAATAAATAAGCACGAGGAGATGCTAGGCTGATCAAAAGAAGAAGGATTAGCGAGAAGTATATCTAACTTTATTCTCCAATTGCAATTGACTGATTATAGATTTTCTATATTCTTTTTCTATTTTCCTAGCGAACTAGCAGCTCCCCCGCCCTACACAGGATTGGATACTGAACCTGATCAGATAGACCGATCGGTGCGTAAGATGATAGTGACGCACTAGGTTTGATCTCGCTACCTACTATGTATACTGTTATAGCTGTTCTTCTACCTCTATCTACTGAGGATATTGGAGCACTAACCCGCAACTTTCATCTACGCTAACAGCTCTCTTCTCTTAATCCGCCTTCGATTATTCATTAGCGCTCCGCCGGGTCCTCCTGCTACTCAAACAGCTTTCTTTAGGAAATGCCGACATCTACTACTAATGGATTGACATTCCAGAACAGGAAGTCATTTGAATGCCCGAAGTCTGCATCTTCTATTACATCGCCGAAAGAGTGAAAAGTCCCTTCTTGCCTGATGAGTTCTTCTTTCTTTTCTTGCTCCTCACAGTCAAGATCCCGCAAATTCTGACTGACTTGAAATTAATAGTCGTTTTATCTGATGATATGAGATGACGAGCGTCAGACAGAGAAAGAGAAAAAAAGTCTAGTCAAGAAGCTTAACGGTGAAGCTTTACATCAAAAAGTAACGAAAAAAGACGGGTTTGAGCAACTTTTTCATACAGTTTTGCCCTCTG

>sca4

TTTCTCCCCTTGCGTTGCTAATTCCTCATAAAGCCCCTCCCTCCATACGTCCAGTTGATCATCCCGAACCGCATCCCGGGTTTGACAGAGGGAAATTTTGCGCTCCAGCTCGCGCACCTCCCGATCTATGCGCTCCGCCCCTTCCCGACGACGGTCGGGATCCCTCTCGTTCTGGAGGTTCCGTTCCATTTCATGGATTCGCTCCAGATCCTCCCCCAATTGGAGATTCCGCGCTCGTAAAGAGTCGGAACTC

>sca5

GTGAGAGGAATGCAATAACTCGACTGTAAGGTCCACCAGGTCCGTAGGAGAGTCAGTCTTTCTCTAAGCAAGCTGTTTTCTGGCCTATACGGAAAGAGTTTTCAAGGAGGAACCCAGCTTATCCCCTCTCAGCGGAGAAGGAGGAACCCAGCTTCTCTTCTCTCAGAGGAGGAACCCCTTTCCTTCCGTCAGTCTCCAATTTTCGTTAGTTCTCTCTCTCTCTTAACAGAGCGGAATTCT
