# Supplementary material for: A large-scale population based organelle pan-genomes construction and phylogeny analysis reveal the genetic diversity and the evolutionary origins of chloroplast and mitochondrion in Brassica napus L
Source: BMC Genomics. 2022 Apr 30;23:339. doi: 10.1186/s12864-022-08573-x (PMC9063048; doi:10.1186/s12864-022-08573-x)
Supplement: Supplementary file 2 — Additional file 2. Supplemetary figures. [file 12864_2022_8573_MOESM2_ESM.docx]

**Supplementary Figures:**

**
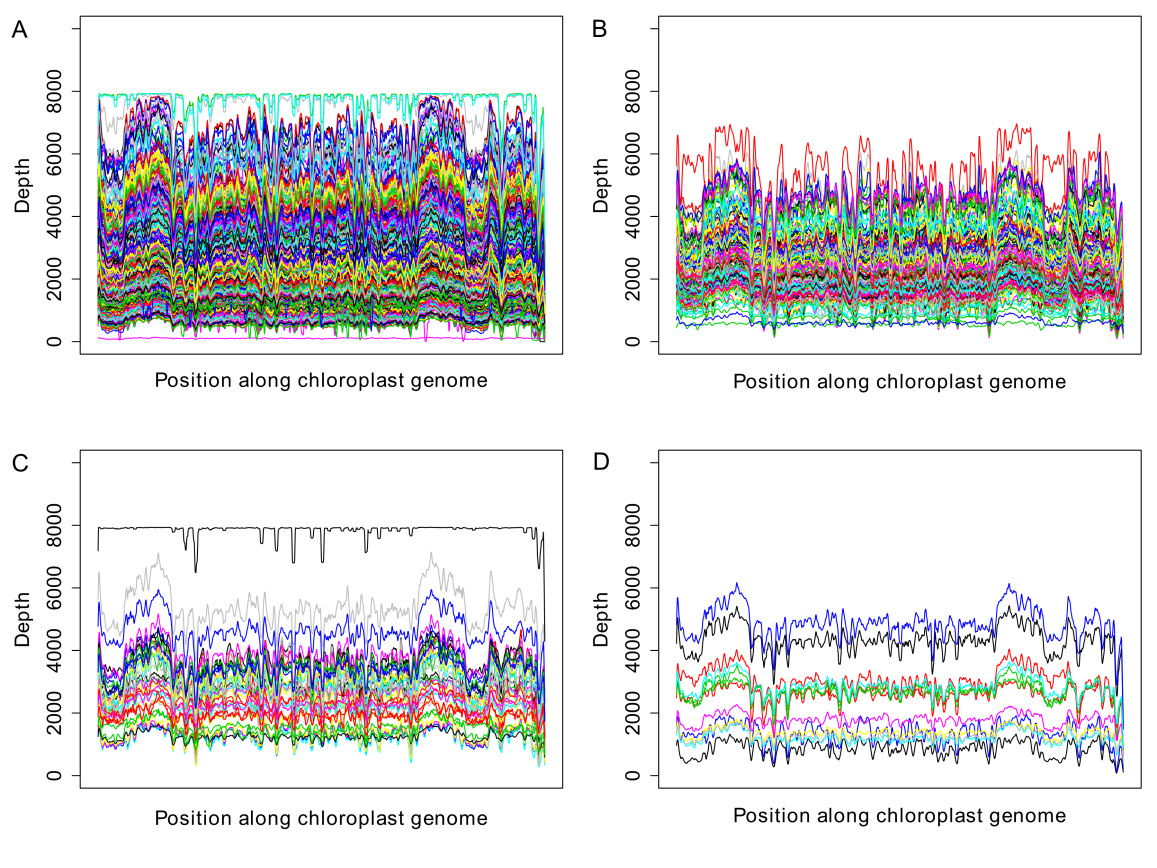
**

**Figure S1.** Genome coverage along assembled cpDNAs for different cytoplasm types. (A) Accessions corresponding *nap*-type. (B) Accessions corresponding *cam*-type. (C) Accessions corresponding *pol*-type. (D) Accessions corresponding *ole*-type.


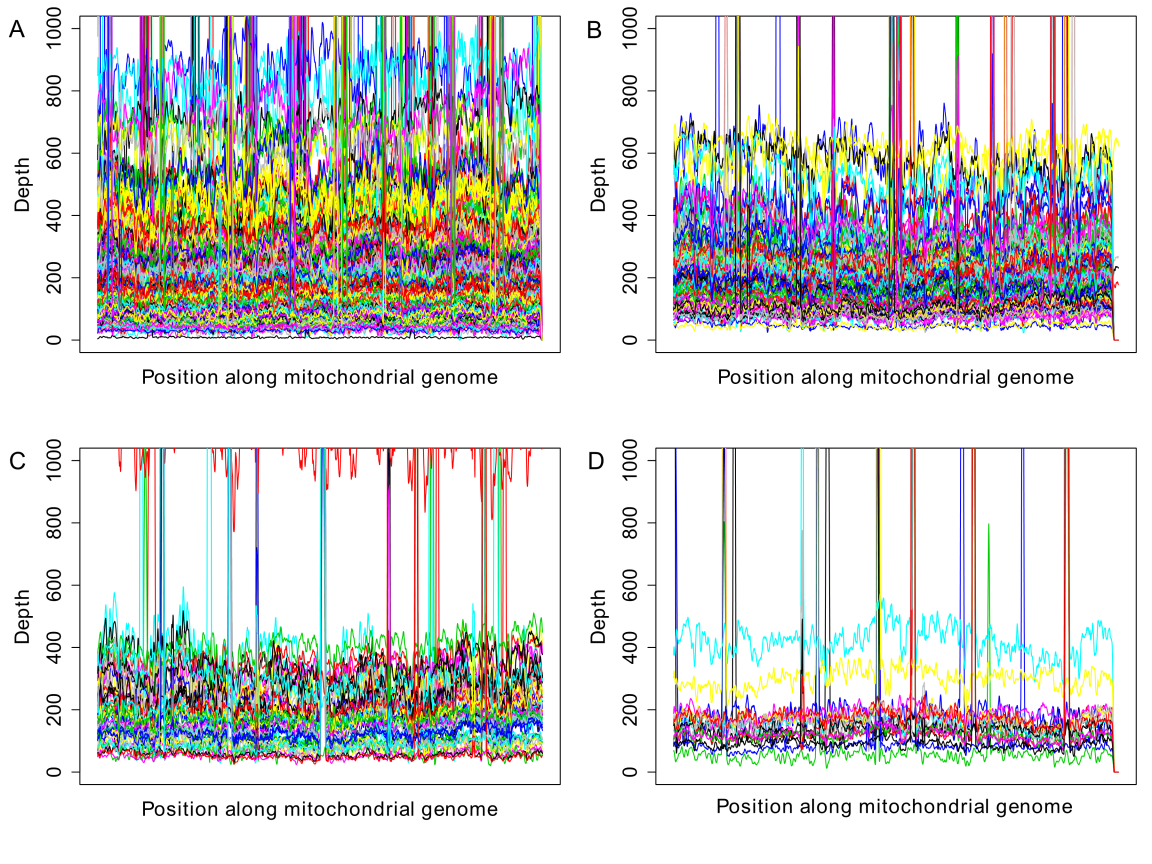


**Figure S2.** Genome coverage along assembled mtDNAs for different cytoplasm types. (A) Accessions corresponding *nap*-type. (B) Accessions corresponding *cam*-type. (C) Accessions corresponding *pol*-type. (D) Accessions corresponding *ole*-type.


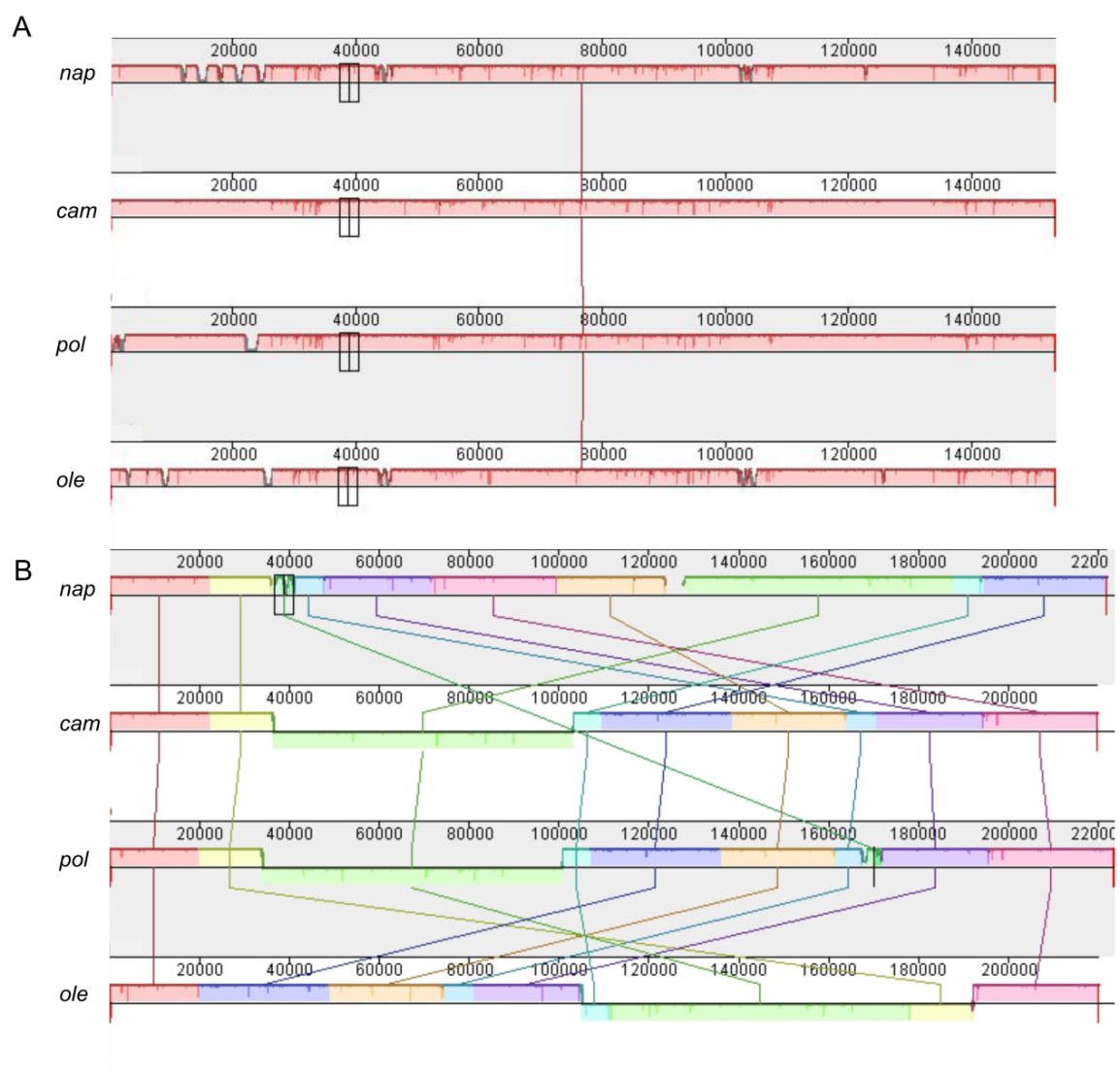


**Figure S3.** Collinear block sequences identified among the four cytoplasm haplotypes. (**A**) Four cp genomes (**B**) Four mt genomes. The collinear blocks were exhibited by mauve visualization. Contiguously colored region is a locally collinear block (LCB) region without rearrangement of homologous sequence.


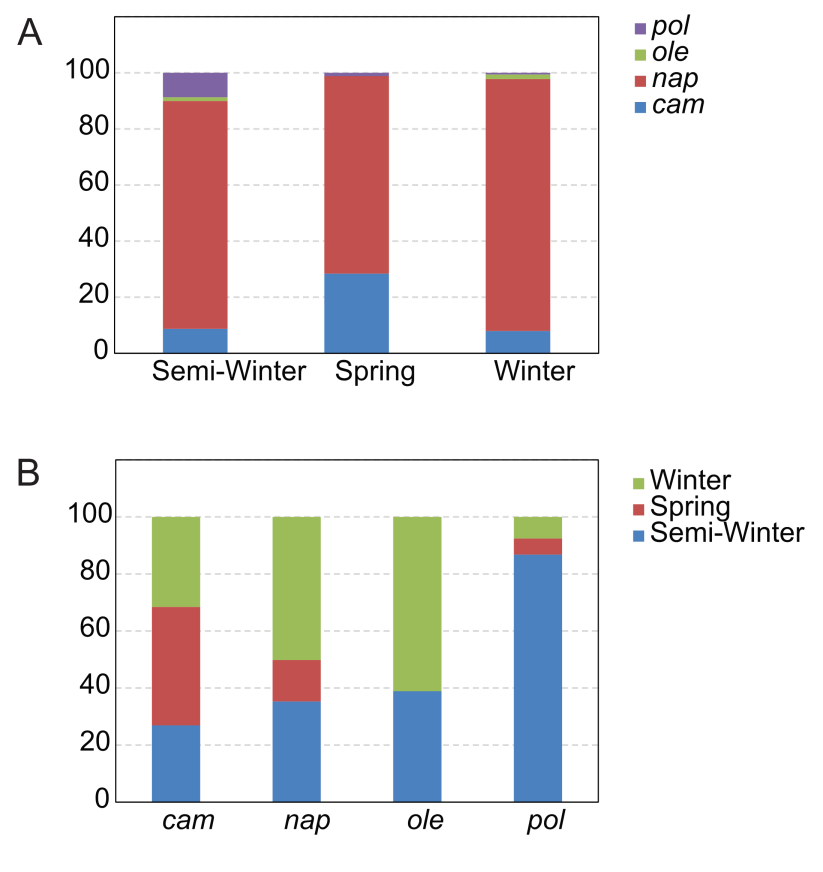


**Figure S4.** Distribution between ecotypes and mitotypes. (A) Distribution of different cytoplasm in three ecotypes. (B) Distribution of different ecotype in four cytoplasm types.


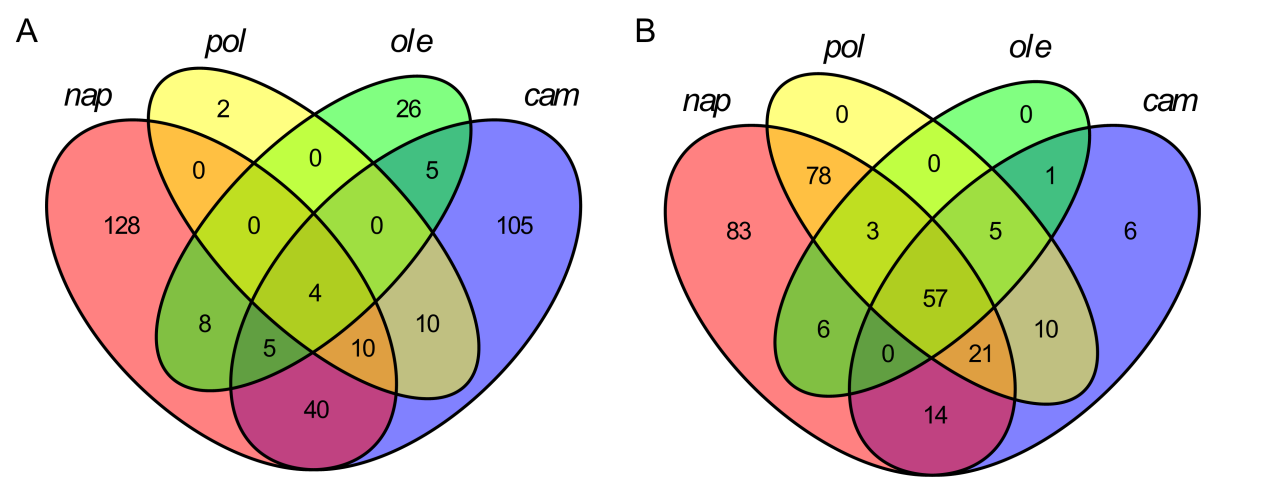


**Figure S5.** Venn plot of variants for four cytoplasm types. (A) Variants in cp genomes. (B) Variants in mt genomes.


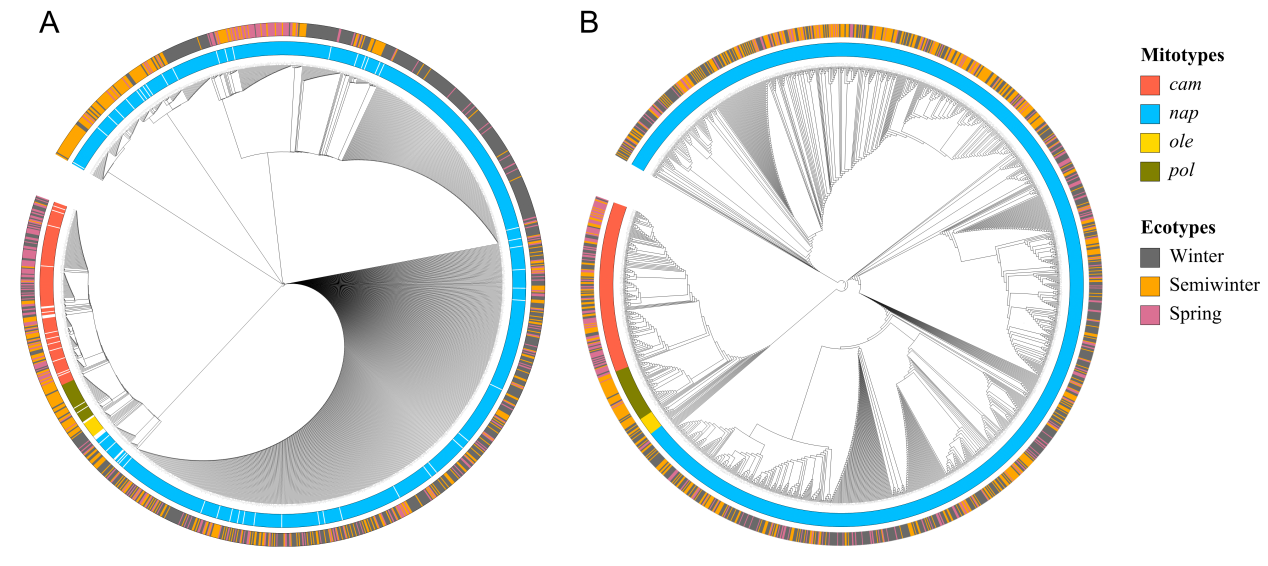


**Figure S6.** Phylogenetic ML tree of assembled rapeseed organelle genomes. phylogenetic tree were constructed for (A) cp and (B) mt respectively, using SNPs with minor allele frequencies (MAFs) >0.5% and missing calls <20%. The layer rings indicate the group name of each clade. Samples in different cytoplasm types and ecotypes in tree were highlighted by different colors.


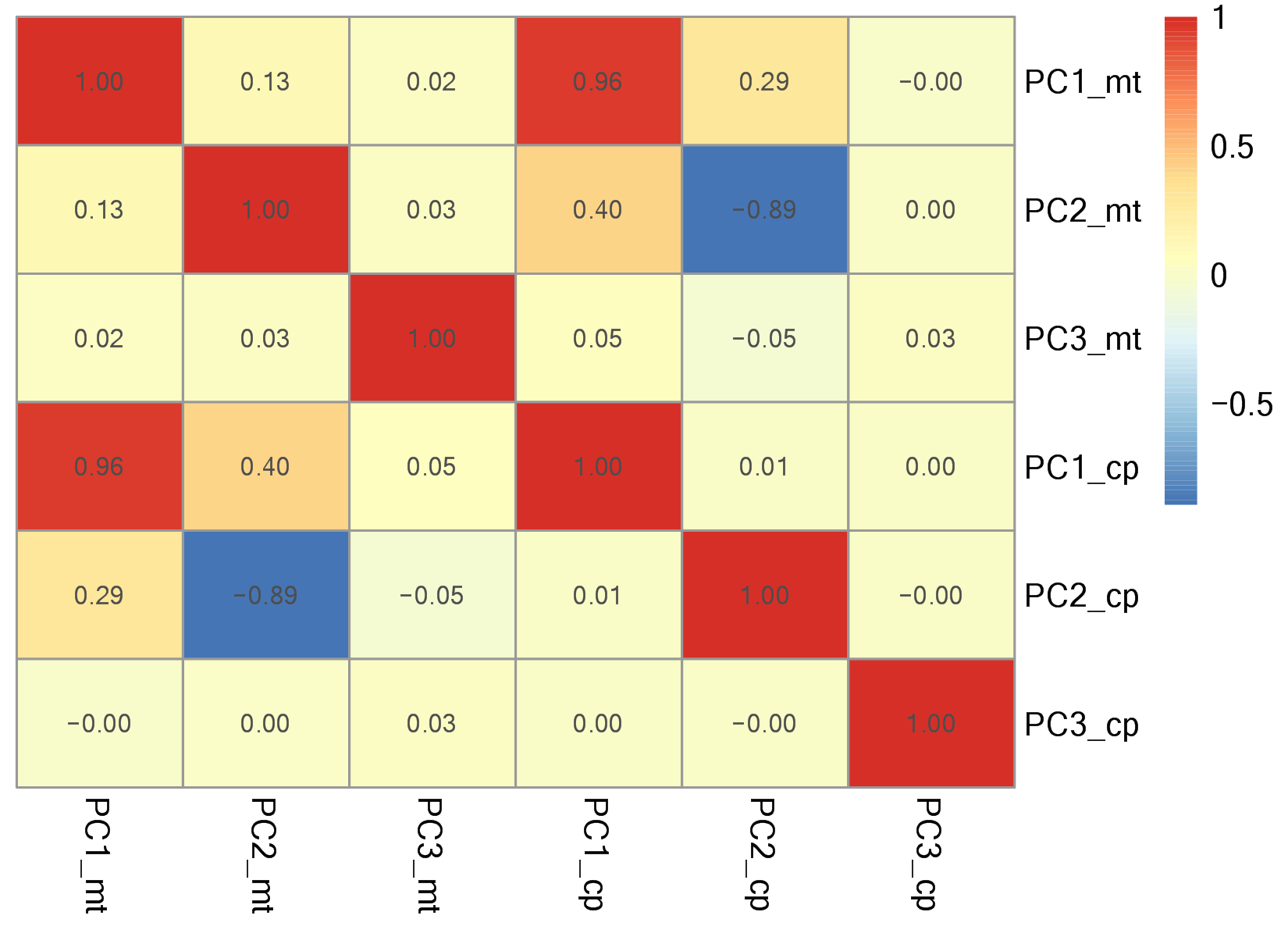


**Figure S7.** The correlation coefficient of top 3 Principal Component derived from cpDNA and mtDNA.


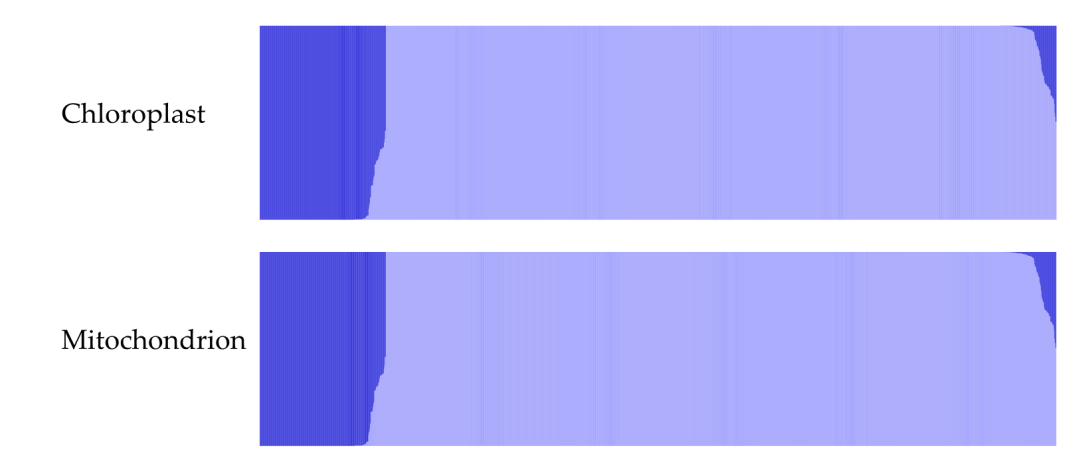


**Figure S8.** Model-based Bayesian clustering of assembled *B. napus* accessions performed using STRUCTURE with the number of ancestry kinships (K) set to 2.


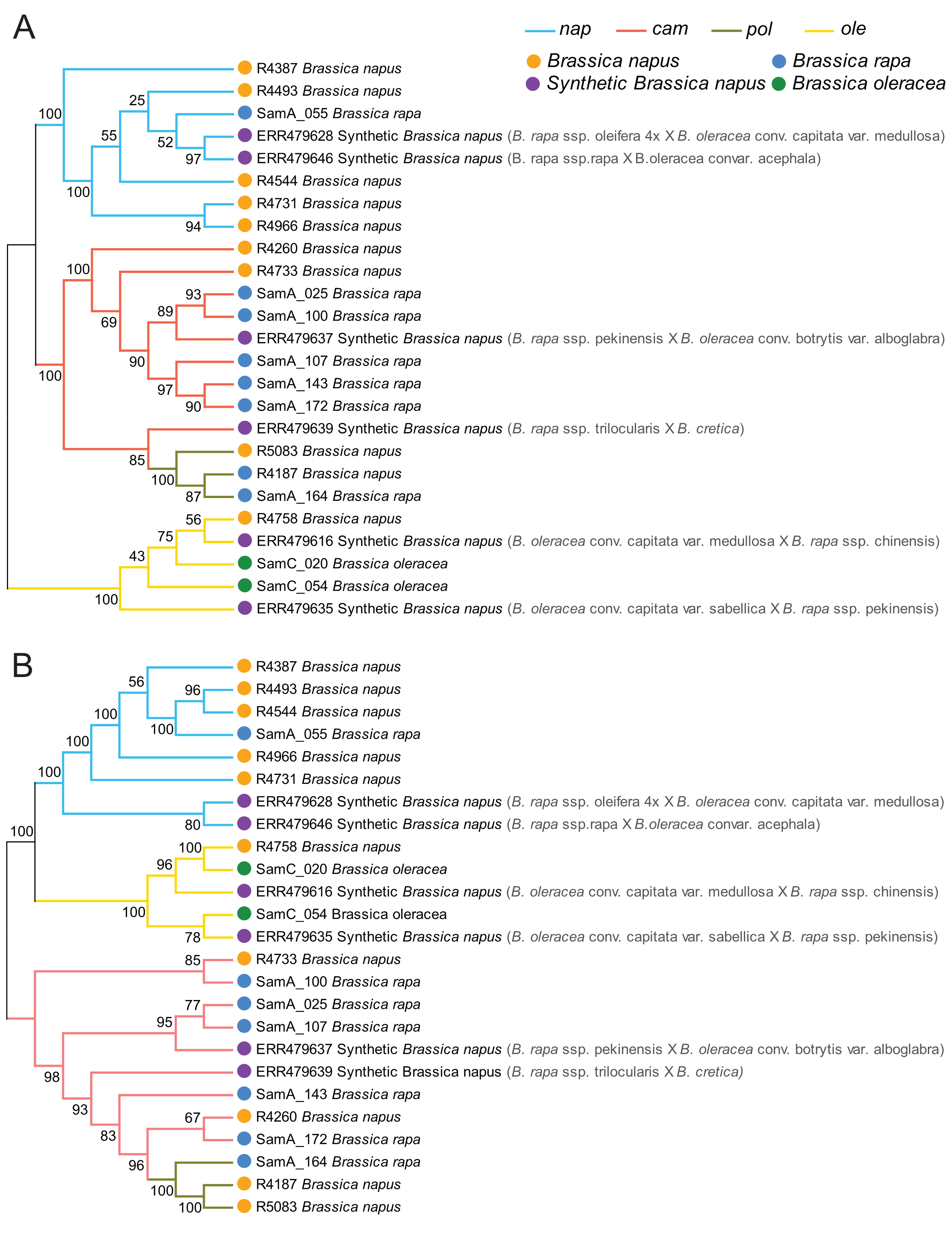


**Figure S9.** (A) Chloroplast phylogeny and (B) Mitochondria phylogeny of four cytoplasm haplotypes based on samples selected form different clades of all accessions.
